# Integrative proteome analysis implicates aberrant RNA splicing in impaired developmental potential of aged mouse oocytes

**DOI:** 10.1111/acel.13482

**Published:** 2021-09-28

**Authors:** Mingrui Li, Chao Ren, Shuai Zhou, Yuanlin He, Yueshuai Guo, Hao Zhang, Lu Liu, Qiqi Cao, Congjing Wang, Jie Huang, Yue Hu, Xue Bai, Xuejiang Guo, Wenjie Shu, Ran Huo

**Affiliations:** ^1^ State Key Laboratory of Reproductive Medicine, Department of Histology and Embryology Suzhou Affiliated Hospital of Nanjing Medical University, Suzhou Municipal Hospital, Gusu School, Nanjing Medical University Nanjing China; ^2^ Department of Clinical Nursing, School of Nursing Nanjing Medical University Nanjing China; ^3^ Department of Biotechnology Beijing Institute of Radiation Medicine Beijing China

**Keywords:** Cdk9, DNA damage, oocyte aging, oocyte quality, preimplantation embryo, proteomics, PUF60, splicing

## Abstract

Aging has many effects on the female reproductive system, among which decreased oocyte quality and impaired embryo developmental potential are the most important factors affecting female fertility. However, the mechanisms underlying oocyte aging are not yet fully understood. Here, we selected normal reproductively aging female mice and constructed a protein expression profile of metaphase II (MII) oocytes from three age groups. A total of 187 differentially expressed (DE) proteins were identified, and bioinformatics analyses showed that these DE proteins were highly enriched in RNA splicing. Next, RNA‐seq was performed on 2‐cell embryos from these three age groups, and splicing analysis showed that a large number of splicing events and genes were discovered at this stage. Differentially spliced genes (DSGs) in the two reproductively aging groups versus the younger group were enriched in biological processes related to DNA damage repair/response. Binding motif analysis suggested that PUF60 might be one of the core splicing factors causing a decline in DNA repair capacity in the subsequent development of oocytes from reproductively aging mice, and changing the splicing pattern of its potential downstream DSG *Cdk9* could partially mimic phenotypes in the reproductively aging groups. Taken together, our study suggested that the abnormal expression of splicing regulation proteins in aged MII oocytes would affect the splicing of nascent RNA after zygotic genome activation in 2‐cell embryos, leading to the production of abnormally spliced transcripts of some key genes associated with DNA damage repair/response, thus affecting the developmental potential of aged oocytes.

## Graphical abstract

In this study, we constructed the dynamic proteomics of mouse MII oocytes during female aging. Analysis of differentially expressed proteins pointed out that a large number of splicing proteins were down‐regulated in aged mouse oocytes, suggested RNA splicing as a novel mechanism to regulate mouse oocyte aging. Dysregulation of key splicing factors, such as PUF60, relates to the impaired developmental potential of aged mouse oocytes.

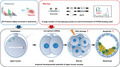

## 1 INTRODUCTION

Fertility decline has increasingly become a public health concern in recent decades. Infertility currently affects 10–15% of couples worldwide (Sharma et al., 2013), and a decline in female fertility is one of the leading causes of infertility (Vander Borght & Wyns, 2018). Female fertility refers to the ability of a woman to produce mature oocytes, fertilize, and give birth to a healthy child. Many factors influence female fertility, among which age is an important physiological and uncontrollable factor (Sauer, 2015). Female fertility declines with age: the incidence of infertility is less than 15% in women younger than 35 years, rising to about 30% in women aged 35–40 years and about 64% in women older than 40 years (Crawford & Steiner, 2015). However, in modern society, women in many populations tend to postpone childbearing, and childbearing by women of an advanced maternal age has become an urgent global issue (Chen, 2019).

The main reasons for the irreversible fertility decline observed in women with age are decreased ovarian function and endometrial receptivity, of which decreased oocyte quality is a determining factor (Tatone, 2008). Oocytes increase in size during their growth phase and accumulate maternal mRNAs and proteins (Chermuła et al., 2018). These maternal factors stored in mature oocytes are of great significance in determining the developmental potential of oocytes and guiding the subsequent early embryonic development (Li et al., 2010). Changes in maternal factors in aged oocytes influence not only its own quality but also the health of embryos after fertilization. There is by now increasing evidence that cohesion fatigue is a main factor in predisposition to aneuploidy that greatly restricts developmental potential of oocytes as women age (Capalbo et al., 2017; Gruhn et al., 2019). Previous studies have found that advanced maternal age can influence oocyte quality by affecting nuclear and cytoplasmic maturation during oocyte development (Conti & Franciosi, 2018). Some genes/proteins have also been confirmed to be related to the quality of aged oocytes *in vivo* or *in vitro*. For example, alterations in proteome of structural maintenance of chromosomes and spindle assembly checkpoint (SAC) in aged oocytes (Schwarzer et al., 2014). The expression of proteins involved in the SAC, such as BUB1, MAD2, and BUBR1, decreased in DNA‐damaged metaphase I (MI) oocytes of aged mice, leading to the generation of chromosomal anomalies in metaphase II (MII) oocytes (Marangos et al., 2015). The substantial reduction in HDAC3 protein in oocytes from aged mice is associated with the failure of meiotic apparatus assembly (He et al., 2019). Defects in the expression of members of the sirtuin family can lead to abnormal oocyte maturation and early embryonic development in aged mice (Qiu et al., 2018; Tamura et al., 2017; Zeng et al., 2018). However, due to the complexity and multifactorial nature of the age‐related decline in oocyte quality, the mechanisms have not been fully resolved, and currently, there is no effective approach to improve oocyte quality and pregnancy outcome.

It has been reported that the reproductive aging pattern of C57BL/6 female mice is similar to that of human females: The ovarian reserve and the litter size decrease with age, and natural infertility occurs in about half of the survival age (Boot et al., 1956; Coxworth & Hawkes, 2010). Aged female mice also exhibit many physiological changes similar to menopausal women, including irregular oestrus cycles (Boot et al., 1956; Nelson et al., 1981), making it an ideal *in vivo* model for studying reproductive aging. Herein, we used C57BL/6 female mice in three different age groups of 8–10 weeks (8–10 w), 6–8 months (6–8 m), and 10–12 months (10–12 m) as models to evaluate the quality of oocytes to identify the phenotypes of the decline in the quality and developmental potential of aged oocytes. We constructed a protein expression profile of MII oocytes of three age groups and found that a large number of splicing regulatory proteins were downregulated in MII oocytes of reproductively aging mice, which affected the splicing events and led to the impaired quality of preimplantation embryos. Our results uncover a novel functional role of splicing during mouse oocyte aging and provide direct coupling evidence about splicing proteins and the developmental potential of aged oocytes.

## 2 RESULTS

### 2.1 Declined quality and developmental potential in aged oocytes

**FIGURE 1:**
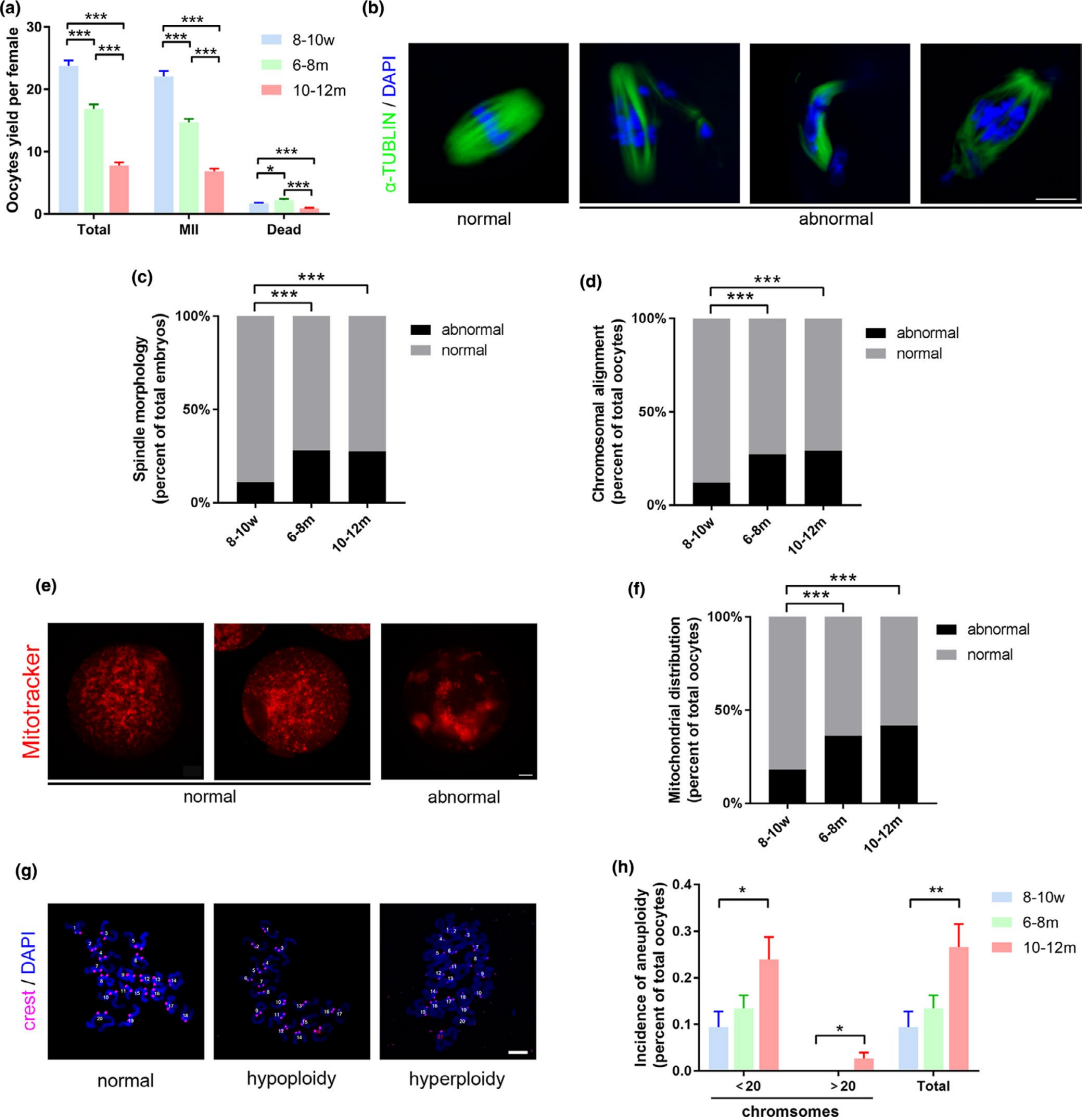
Decreased quantity and quality in aged oocytes. (a) Yield and morphology of oocytes obtained after superovulation of 8–10 w (117 independent experiments), 6–8 m (101 independent experiments) and 10–12 m (110 independent experiments) mice. T‐test (two‐tailed) was used for statistical analysis. (b) Representative examples of spindles in MII oocytes from the mice of three age groups, after labelling with α‐tubulin antibody (green) and counterstaining of DNA with Hoechst (blue). (c, d) Incidence of spindle abnormalities (c) and chromosomal misalignment (d) in MII oocytes of 8–10 w (*n* = 201 from eight independent experiments), 6–8 m (*n* = 174 from six independent experiments), and 10–12 m (*n* = 127 from four independent experiments) mice. Chi‐square test was used for statistical analysis. (e) Representative mitochondrial distribution in MII oocytes from 8–10 w, 6–8 m, and 10–12 m mice (MitoTracker staining shown in red). (f) Incidence of abnormal mitochondrial aggregation in MII oocytes from 8–10 w (*n* = 209 from eight independent experiments), 6–8 m (*n* = 185 from six independent experiments), and 10–12 m (*n* = 134 from four independent experiments) mice. Chi‐square test was used for statistical analysis. (g) Example of typical normal, hypoploidy and hyperploidy chromosome spread of MII oocyte (Hoechst staining of DNA shown in blue). (h) Incidence of hyperploidy and hypoploidy (and total chromosomal defects from these two endpoints combined) in MII oocytes of 8–10 w (*n* = 182 from nine independent experiments), 6–8 m (*n* = 106 from five independent experiments), and 10–12 m (*n* = 117 from seven independent experiments) mice. Hyperploidy was not detected in MII oocytes from 8–10 w and 6–8 m mice. T‐test (two‐tailed) was used for statistical analysis. Data are presented as mean ±SEM. *p < 0.05, **p < 0.01, ***p < 0.001. Scale bar, 10 μm

**FIGURE 2:**
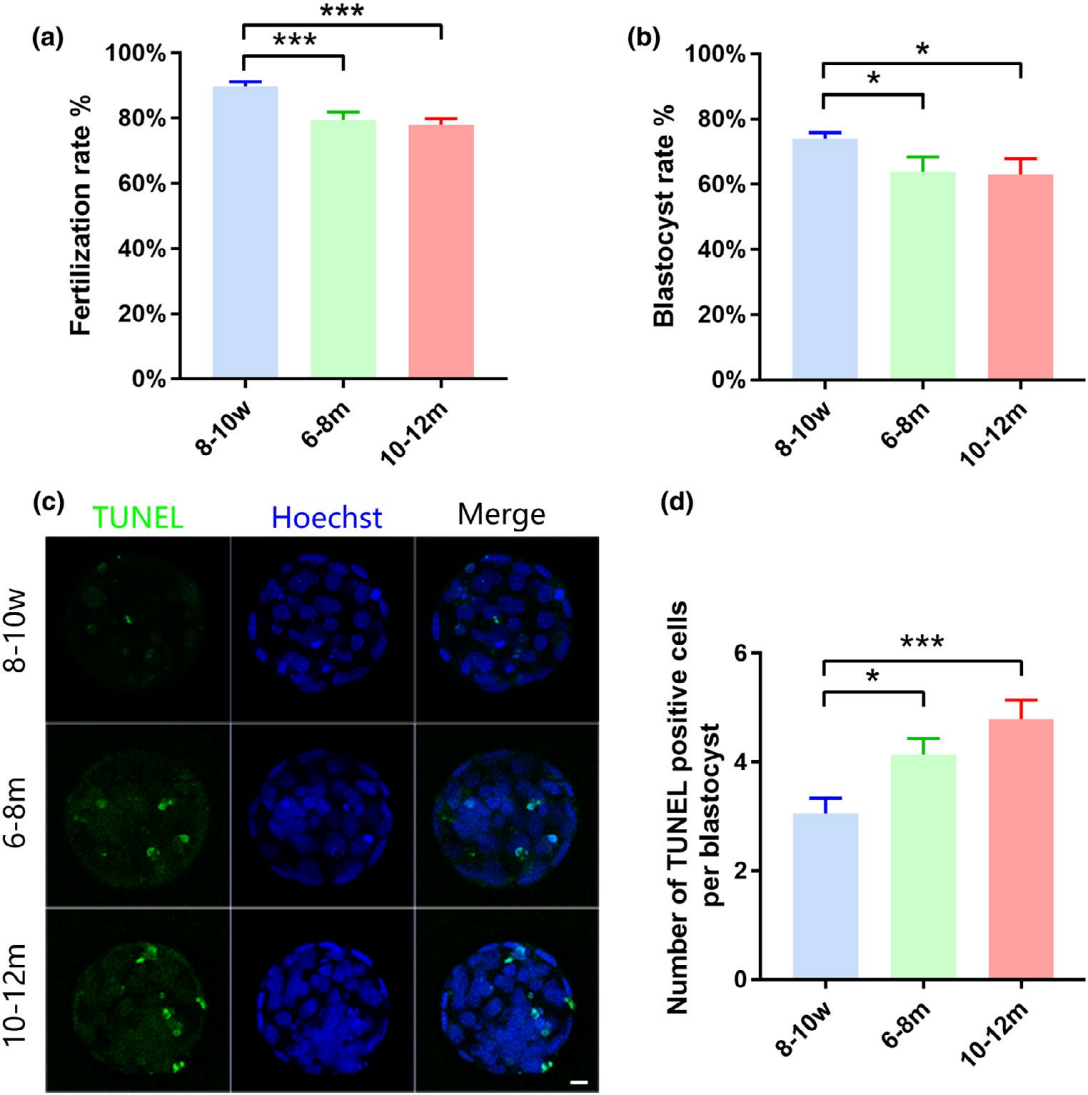
Declined developmental potential in aged oocytes. (a) Fertilization rate of MII oocytes rate from 8–10 w, 6–8 m, and 10–12 m groups (45 independent experiments). (b) Blastocyst rate in 8–10 w, 6–8 m, and 10–12 m groups (16 independent experiments). (c) The nuclei in the blastocyst were stained by Hoechst (blue), and apoptotic cells were detected by TUNEL assay (green). (d) Apoptotic cell number per blastocyst in 8–10 w (*n* = 95), 6–8 m (*n* = 81), and 10–12 m (*n* = 124) groups (from four independent experiments). A two‐tailed t‐test was used for statistical analysis. Data are presented as mean ±SEM. *p < 0.05, ***p < 0.001. Scale bar, 10 μm

First, the quantity and quality of MII oocytes were evaluated among the three groups. We found that the number of oocytes after superovulation decreased dramatically in the 6–8 m and 10–12 m group, especially in the latter (Figure 1a). Fluorescence staining for the spindle and chromosomes showed that normal MII oocytes presented a typical barrel‐shaped spindle and well‐aligned chromosomes on the metaphase plate, while a higher proportion of abnormal spindle morphologies (such as irregular, unipolar, or multipolar spindles) and chromosome alignment (such as misaligned, scattered, and even moved to poles of spindle) were observed in the two reproductively aging groups (6–8 m and 10–12 m) (Figure 1b–d). As described previously (Zeng et al., 2018), there are mainly three different patterns of mitochondrial distribution in MII oocytes (Figure 1e), including polarized distribution, homogeneous distribution, and clustering distribution. MitoTracker was used to stain the mitochondria of MII oocytes from the three age groups. We observed that the proportion of abnormal mitochondrial aggregation in oocytes increased significantly with aging (Figure 1f). Chromosome spreads of MII oocytes were also performed, and the chromosome numbers were determined (Figure 1g). The results showed that the incidences of hypoploidy markedly increased in the 10‐12m group (Figure 1h). The quality of the aged oocytes decreased, and the developmental potential was also decreased, which was manifested by the decreased fertilization rate and blastocyst formation rate after *in vitro* fertilization (IVF) (Figure 2a, b), and a significantly increased number of apoptotic cells in blastocysts (Figure 2c, d).

### 2.2 Identification of DE proteins during mouse oocyte aging

**FIGURE 3:**
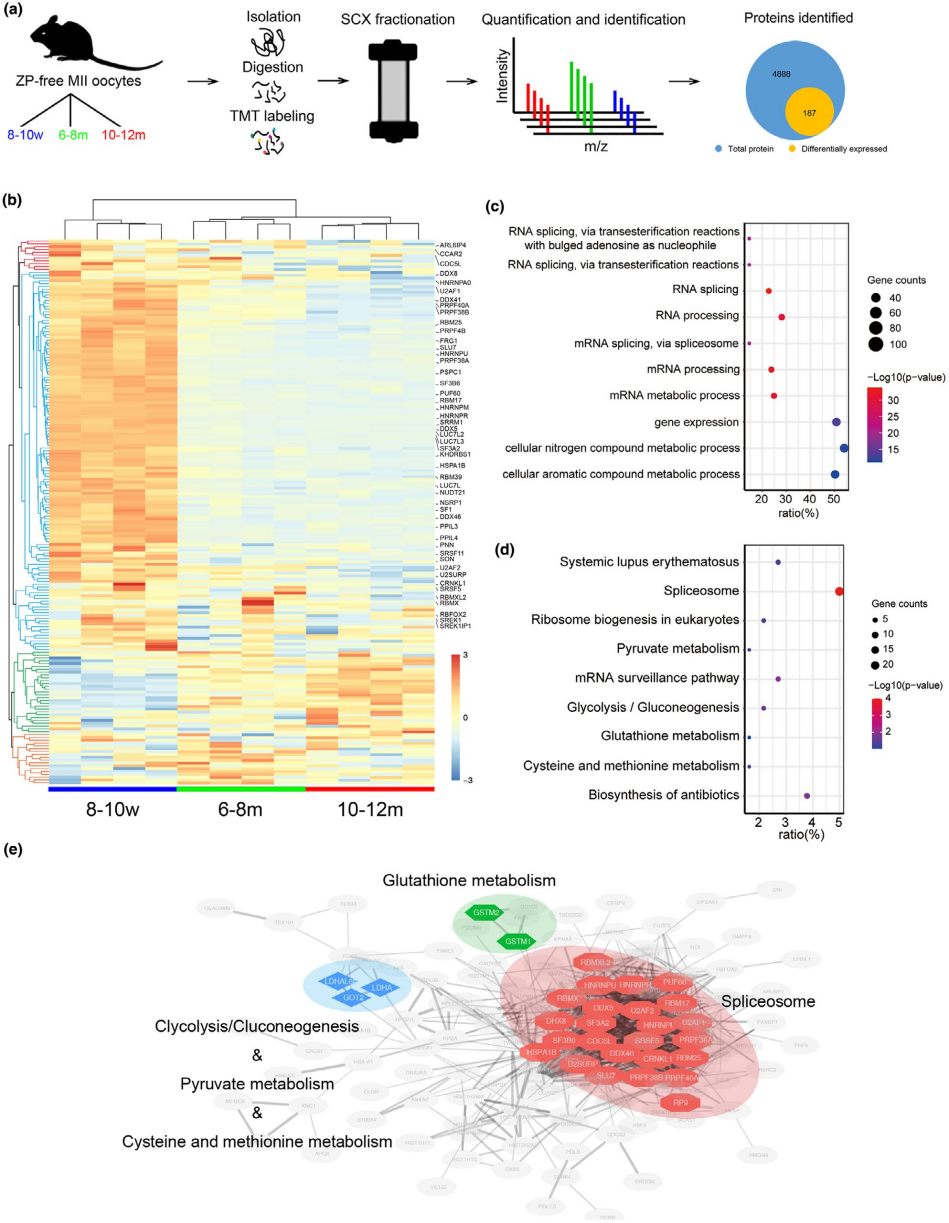
Proteomics of MII oocytes from three age groups and bioinformatic analysis. (a) Schematic of the experimental design for proteomics, with assay results for MII oocytes from 8–10 w, 6–8 m, and 10–12 m mice. (b) Heatmap and hierarchical clusters of the 187 DE proteins. The raw values are normalized using z‐score. Fifty proteins identified as splicing related are labelled. (c) Gene ontology enrichment analysis of the 187 DE proteins using DAVID. The ratio is identified as the involved genes/total genes. (d) KEGG pathway enrichment analysis of the 187 DE proteins using DAVID. The ratio is identified as the involved genes/total genes. (e) Protein‒protein interaction networks of DE proteins was performed using STRING and functional protein interaction networks were visualized using Cytoscape

To better understand the molecular mechanisms that lead to the decline of the developmental potential of aged oocytes, we collected MII oocytes after zona pellucida removal from three age groups and constructed the proteome profile. A total of 4888 quantitative proteins were identified, of which 187 were differentially expressed (DE) (P‐value less than 0.05 and fold change greater than 1.5) (Figure 3a, Table S1). The results of principal component analysis (PCA) showed good intra‐group repeatability, and clearly distinguished the reproductively aging groups from the younger groups (Figure S1a). The heatmap showed that the DE proteins were divided into four clusters, according to their expression patterns, among which proteins downregulated with age constituted the largest proportion (Figure 3b).

### 2.3 DE proteins are highly enriched in RNA splicing

GO enrichment analysis showed that DE proteins were enriched in the biological process of RNA splicing (Figure 3c, Table S2). Similarly, the KEGG pathway analysis of DE proteins showed the most significant enrichment for the "spliceosome" pathway (Figure 3d, Table S3). We constructed a putative protein interaction network using the STRING database (Szklarczyk et al., 2017) and found that spliceosome‐related DE proteins were most enriched in pathway enrichment and formed the largest and densest network (Figure 3e). These results demonstrated that DE proteins were highly enriched in RNA splicing. For further refinement, we found that up to 50 DE proteins (50/187) were splicing‐related proteins based on RNA splicing or spliceosome‐associated GO terms (Ashburner et al., 2000; The Gene Ontology, 2019), spliceosome pathway from KEGG database (Kanehisa et al., 2016) and mRNA splicing pathway from the Reactome database (Fabregat et al., 2018). By mapping the 50 splicing‐related proteins to the mRNA splicing pathway of the Reactome database (Fabregat et al., 2018) and the Spliceosome database (Cvitkovic & Jurica, 2013), we found that these splicing‐related DE proteins were mainly involved in the formation of core complexes during spliceosome assembly (Figure S1b). Notably, most of these splicing‐related proteins (48/50) were downregulated during oocyte aging (Figure 3b).

### 2.4 Abnormal splicing of 2‐cell embryos and increased DNA damage in morulae in reproductively aging mice

**FIGURE 4:**
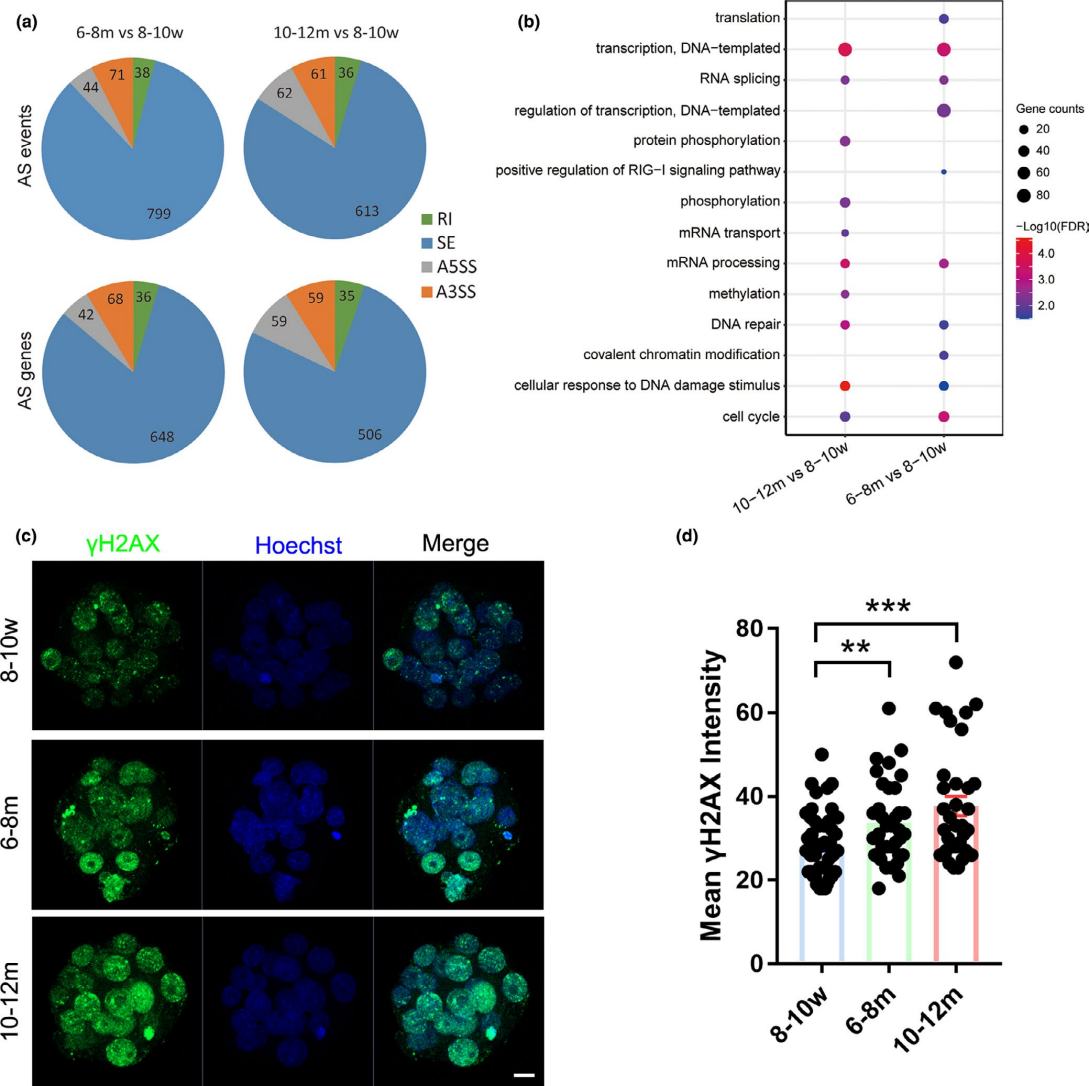
Splicing analysis of 2‐cell embryos in three age groups and increased DNA damage in morulae of reproductively aging mice. (a) Pie chart illustrating the alternative splicing events and involved genes. (b) Gene ontology enrichment analysis of the DSGs between reproductively aging and younger groups. (c) Immunofluorescence staining of γH2AX in morulae from 8–10 w, 6–8 m, and 10–12 m groups. (d) The γH2AX fluorescence intensity of each morula from 8–10 w, 6–8 m, and 10–12 m mice (8–10 w, *n* = 50; 6–8 m, *n* = 39; 10–12 m, *n* = 35; from three independent experiments). A two‐tailed *t*‐test was used for statistical analysis. Data are presented as mean ±SEM. **p < 0.01, ***p < 0.001. Scale bar, 10 μm

It is widely accepted that splicing and transcription are temporally coupled (Naftelberg et al., 2015). Since MII oocytes are in a special transcriptional silencing state, re‐transcriptional activation does not occur until the early embryo (L. Li et al., 2013) (Figure S2a). Therefore, we speculated that changes in the expression of a large number of splicing‐related proteins in the MII oocytes of reproductively aging mice might lead to large‐scale RNA splicing abnormalities in early embryos. It has been reported that mRNAs transcribed at the zygote stage are mostly non‐functional, and the splicing machinery may not be fully formed or function inefficiently, while effective splicing occurs in the 2‐cell stage (Abe et al., 2015), we collected 2‐cell embryos 24–34 h post‐insemination (hpi) for EU incorporation and found that transcriptional activity was strongest at approximately 28 hpi (Figure S2b, c). To better understand the changes in alternative splicing (AS) events during this period, we collected 2‐cell embryos of 28 hpi from three age groups for RNA‐seq. By using rMATS based on the standard protocol, as previously reported (Shen et al., 2014), we found a large number of splicing events and genes at this stage. AS events were divided into four types according to their AS patterns: exon skipping (SE), alternative 5’ splice site (A5SS), alternative 3’ splice site (A3SS), and intron retention (RI). The highest proportion of AS pattern was SE in the 10–12 m/8–10 w and the 6–8 m/8–10 w, which account for about 80% of all AS events (Figure 4a; Tables S4, S5). The results of GO analysis of DSGs in the reproductively aging groups versus the younger group suggested that DSGs were enriched in biological processes related to DNA damage repair/response (Figure 4b). Then, we selected a few DSGs related to DNA damage repair/response to verify the AS changes by RT‐PCR. The validation results were consistent with those of RNA‐seq analysis (Figure S3). We speculated that the dysfunction of DNA damage repair/response‐related genes caused by abnormal splicing would lead to increased DNA damage in early embryos in reproductively aging groups. Therefore, we compared the DNA damage levels of morulae in three age groups by immunofluorescence staining of the classic DNA damage marker γH2AX (Li et al., 2020). By comparing the overall fluorescence intensity in the nucleus of the morulae, we found that the DNA damage level of the morulae from 6–8 m and 10–12 m mice was both significantly higher than that from 8–10 w mice (Figure 4c, d).

### 2.5 DE protein PUF60 is a potential core splicing factor responsible for the decline of oocyte developmental potential in reproductively aging mice

**FIGURE 5:**
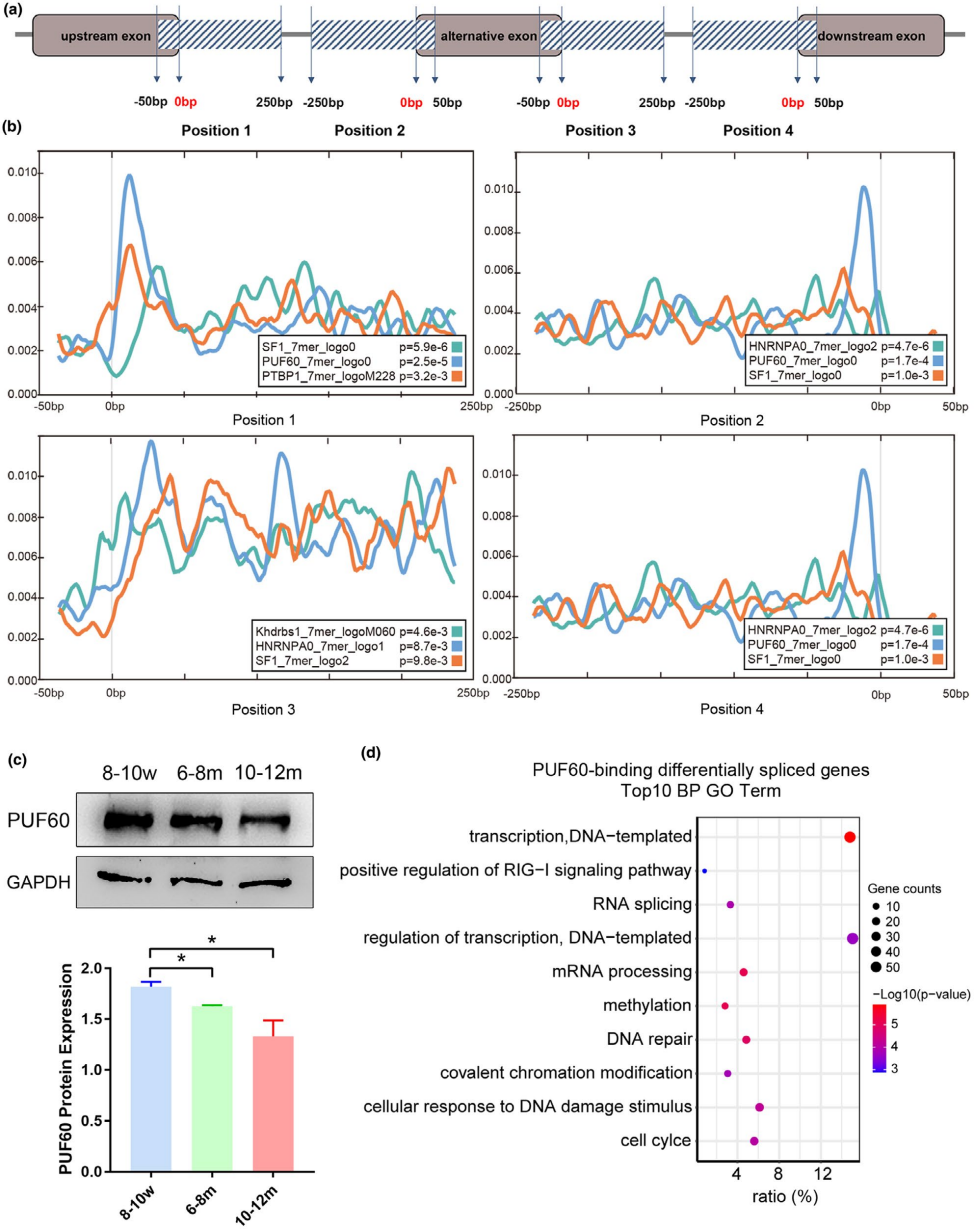
PUF60 is a potential core splicing factor for the developmental potential of aged oocytes. (a) Schematic diagram of scan range of splicing sites in DSGs by binding motif of DE proteins. Schematic diagram of scan range in binding motif analysis. Gray‐rounded rectangles represent exons, straight line represents introns, and the scan range is covered by rectangles with shadows. (b) Density of the binding sites of the splicing factors in four alternative splicing sites. (c) Western blot analysis and quantification of the expression of PUF60 protein and the internal control GAPDH in MII oocytes at different age stages. Data are presented as mean ±SEM from three independent experiments, *T*‐test (two‐tailed), *p < 0.05. (d) GO term enrichment analysis for biological process (BP) of PUF60‐binding DSGs. The ratio was identified as the involved genes/total genes

In the process of AS regulation, a considerable number of RNA‐binding proteins (RBPs) are used as splicing regulatory factors, which can combine with splicing regulatory elements on pre‐mRNA through specific RNA‐binding motifs to regulate alternative splicing. If a splicing factor is a key regulator of DSEs, its RNA‐binding motif should be significantly enriched in AS event regions (Dong et al., 2018). To identify the key splicing regulators in the DE proteins, we collected known motifs from RBP‐motif databases (RBPDB, SpliceAid, and oRNAment) (Benoit Bouvrette et al., 2020; Cook et al., 2011; Giulietti et al., 2013) and performed motif scan on the 300‐bp region of each alternative exon, including a 250‐bp region located on the intron side and a 50‐bp region located on the exon side (Figure 5a) using an analysis tool, CentriMo (Bailey & Machanick, 2012). The DSGs of the reproductively aging groups versus the younger group were scanned, and the results showed that the binding motifs of some DE proteins were significantly enriched, in which the enrichment of PUF60‐binding motif showed a highly matched specific and significant enrichment (Figure 5b). PUF60 protein expression was examined in the MII oocytes of three age groups by western blotting and was in agreement with the tandem mass tag (TMT) quantification (Figure 5c). We further searched for DSGs with the PUF60‐binding motif as potential PUF60‐target genes (Table S6). GO analysis of PUF60‐target DSGs of the reproductively aging groups versus the younger group showed that ‘cellular response to DNA damage stimulus’ and ‘DNA repair’ were present in the top 10 GO terms, suggesting that PUF60 might be the core splicing factor causing decline in DNA repair capacity and possibly thereby also the decreased developmental potential of oocytes in reproductively aging mice (Figure 5d).

### 2.6 Altered splicing of DSG *Cdk9* mimics the phenotype of impaired early embryo quality in reproductively aging mice

**FIGURE 6:**
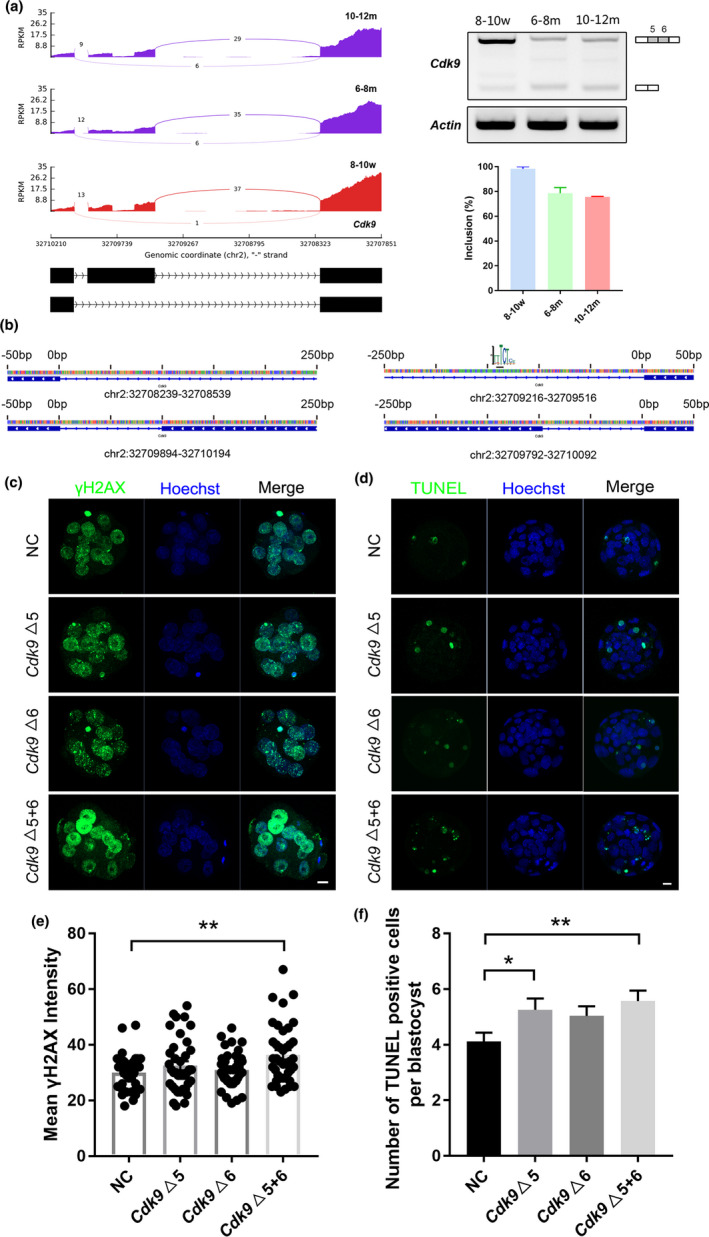
Altered splicing of DSG *Cdk9* impairs early embryo quality. (a) Sashimi plot and RT‐PCR validation of *Cdk9* gene in MII oocytes of three age groups. The alternative exons are marked in gray. The exon inclusion level (Inclusion%) was predicted by RNA‐Seq data. (b) Prediction of the binding sites of PUF60 in *Cdk9* alternative splicing sites. (c) Immunofluorescence staining of γH2AX in morulae from different morpholino‐injected groups. (d) The nuclei in the blastocyst were stained by Hoechst (blue), and apoptotic cells were detected by TUNEL assay (green). (e) The γH2AX fluorescence intensity of each morula in different morpholino‐injected groups (NC, *n* = 33; *Cdk9*△5, *n* = 38; *Cdk9*△6, *n* = 40; *Cdk9*△5+6, *n* = 40; from three independent experiments). (f) Apoptotic cell number per blastocyst in different morpholino‐injected groups (NC, *n* = 94; *Cdk9*△5, *n* = 74; *Cdk9*△6, *n* = 108; *Cdk9*△5+6, *n* = 82; from three independent experiments). A two‐tailed *t*‐test was used for statistical analysis. Data are presented as mean ±SEM. *p < 0.05, **p < 0.01. Scale bar, 10 μm

Among the target genes of PUF60, DSG *Cdk9* is a key molecule in the DNA damage repair/response pathway (Cress, 2017). Exons 5 and 6 of *Cdk9* were skipped in the reproductively aging groups (Figure 6a). The binding motif of PUF60 was scanned near the splicing sites of the skipped exons of *Cdk9*, and the binding sequence of PUF60 was found on the *Cdk9* transcript (Figure 6b). To further investigate the effect of splicing changes of *Cdk9* on preimplantation embryos, we designed morpholinos to block splicing at the splicing sites of exons 5 and 6 of *Cdk9*. The zygotes were separately (△5, △6) or mixed (△5+6) injected with two *Cdk9* morpholinos, then 2‐cell embryos of 28 hpi were collected from each morpholino‐injected group for splicing verification. After mixed injection, exons 5 and 6 of *Cdk9* were skipped and successfully mimicked the splicing changes in the reproductively aging groups (Figure S4a). The morpholino‐injected zygotes of each group were cultured *in vitro* to the blastocyst stage, and we found that the blastocyst development rate of the *Cdk9* △5+6 group was slightly reduced, but there was no significant difference compared with the control group (Figure S4b). Next, we collected morulae from each group for γH2AX staining and found that the DNA damage level of the morulae in the *Cdk9* △5+6 group was significantly higher than that in the control group (Figure 6c, e). TUNEL staining was also performed on blastocysts from each group. We found that the number of apoptotic cells in blastocysts in the *Cdk9* △5+6 group was significantly higher than that in the control group (Figure 6d, f).

## 3 DISCUSSION

Aging is the main risk factor for chronic diseases and an overall decline in health (López‐Otín et al., 2013). The female reproductive system is special because its aging occurs earlier than other tissues and organs, where the earliest manifestation is the decline in the number and quality of oocytes (Crawford & Steiner, 2015). In addition to a decline in oocyte quality, advanced maternal age is also a risk factor for adverse pregnancy outcomes, such as miscarriage, embryo abnormalities, and stillbirths (Sauer, 2015). In recent years, socio‐cultural factors have led to the postponement of women's childbearing age worldwide. As a result, the decline of female fertility caused by reproductive aging is becoming a public health problem worthy of attention (Johnson & Tough, 2012).

Studies of women of advanced maternal age receiving young donor oocytes have shown that the decline in the quality of oocytes is the main driver of age‐related decline in fertility (Navot, 1991; Sauer et al., 1995). In this study, we verified the phenotype of oocyte quality decline in reproductively aging mice at two key time points of oocyte aging. In addition to the decreased oocyte quality, we also focused on the preimplantation embryo development of reproductively aging mice. We found that the developmental potential of aged oocytes was decreased, manifested by a decreased fertilization rate and blastocyst formation rate after IVF, and low‐quality blastocysts characterized by increased apoptosis. These results were consistent with those reported in previous murine and human studies (Hunt & Hassold, 2008; Tamura et al., 2017). The mRNA/protein stored in mature oocytes is key in determining the developmental potential of oocytes and guiding the subsequent early embryonic development (L. Li et al., 2010). Therefore, RNA‐seq and proteomics were used to analyze the gene/protein expression profiles of mature oocytes in mice of different ages to investigate the mechanisms of the decline in the developmental potential of oocytes of reproductively aging mice. Our results showed that only 63 genes were differentially expressed (DEGs) among MII oocytes from 8–10 w, 6–8 m, 10–12 m mice (a total of 63 DEGs, 28 DEGs from 8–10 w versus 6–8 m, 46 DEGs from 8–10 w versus 10–12 m, and 8 DEGs from 6–8 m versus 10–12 m, respectively; Table S7), while 187 proteins were significantly differentially expressed. A similar phenomenon has been reported in a previous study on the transcriptome and proteome of aged mouse oocytes, where the proportion of differential changes in the proteome (3%) was higher than that of the transcriptome (0.05%), but with no correlation (Schwarzer et al., 2014). The inconsistency between transcriptome and proteome also suggests that the effect of aging on oocytes may occur mainly at the post‐transcriptional level (Demant et al., 2015; Trapphoff et al., 2016).

The bioinformatic analysis of the DE proteins of oocytes in the three age groups suggested high enrichment of splicing‐related proteins. In recent years, a series of studies have found that aging leads to changes in alternative splicing in different species and tissues, and these changes are associated with age‐related diseases (Deschênes & Chabot, 2017). Studies have also shown that the expression of splicing factors changes with age in populations and senescent cell models, and similar changes have been reported in other cell systems and aging animal models (Harries et al., 2011), indicating that splicing may be closely related to aging. To our knowledge, no study has reported on the role of alternative splicing in oocyte aging.

It is worth noting that many of the 50 splicing‐related DE proteins identified in our study have been identified as components of the spliceosome, involved in the formation of core complexes during spliceosome assembly, most of which were downregulated in aged oocytes. It has been reported that the downregulation of some splicing factors inhibits the formation of blastocysts (Jumaa et al., 1999; Maserati et al., 2014), where splicing changes in key genes in preimplantation embryos can damage their developmental potential and result in cell death (Perumalsamy et al., 2010). This is consistent with the phenotypes observed in reproductively aging mice with a reduced blastocyst rate and increased apoptotic cells in blastocysts. Recently, evidence has suggested that alternative splicing may be closely related to preimplantation embryonic development, suggesting that splicing may play a key role in regulating early embryonic development (Gómez‐Redondo et al., 2020; Xing et al., 2020).

In the process of alternative splicing regulation, a considerable number of RBPs are used as splicing regulatory factors, which can bind to splicing regulatory elements on mRNA by specific RNA‐binding motifs (Fu & Ares, 2014). In our study, we found that 40 DE proteins were RBPs among the 50 splicing‐related DE proteins. By binding motif analysis, PUF60 was found to be one of the core splicing factors that might critically alter the splicing patterns of some key genes and thus lead to the decreased developmental potential of oocytes in reproductively aging mice. It has been reported that PUF60, as a splicing factor, can bind to uridine (U)‐rich tracts, which is crucial for the recognition of the 3’ splice sites, and promotes the binding of U2 small nuclear ribonucleoprotein particles to the transcript, where PUF60 knockdown in cells alters the splicing pattern of endogenous transcripts (Hastings et al., 2007; Královicová et al., 2018). We also observed an enrichment of the PUF60‐binding motif on introns near splice sites of alternative exons. However, whether the decreased PUF60 expression in the MII stage could mimic the splicing patterns of genes at the 2‐cell stage of the reproductively aging group will require further experimental verification. The GO analysis results of DSGs and PUF60‐target DSGs indicated that DNA damage repair/response‐related genes were highly enriched. We found abnormal splicing changes in key DNA damage repair/response genes (such as *Cdk9*, *Fanca*, *Ube2d3*, and *Mnat1*) in the 2‐cell stage and an increase in the level of DNA damage in morulae in the reproductively aging group. The reduced DNA repair capacity caused by abnormal changes in DNA damage repair/response key gene splicing might contribute to the accumulation of DNA damage, thereby might be an important reason for the increased apoptosis and poor quality in blastocysts in the early embryos of reproductively aging mice. Indeed, we observed increased DNA damage and apoptosis in early embryos after altering the splicing pattern of *Cdk9* in young mice. The rate of blastocyst formation decreased after the splicing pattern of *Cdk9* was changed, but there was no significant difference, indicating that *Cdk9* might not be the main reason for the decrease in blastocyst formation in reproductively aging mice. As a potential target gene of PUF60, *Cdk9* has binding sites of PUF60 on its transcript. However, whether the downregulation of PUF60 in oocytes leads to splicing changes in *Cdk9* and phenotypes with decreased developmental potential of oocytes will need to be confirmed through further experiments. In addition, the effects of splicing changes of *Cdk9* on its transcription, protein, and function remain to be fully elucidated.

In this study, PUF60 was demonstrated as one of the core splicing factors those alterations lead to decrease developmental potential of oocytes in reproductively aging mice. This is one of the possible mechanisms affecting the developmental potential of aged mouse oocytes. However, oocyte aging is a complex physiological process affected by various factors, in which many proteins and molecular pathways are involved. The expression and subcellular localization of the RNA‐binding protein KHDRBS1 (SAM68), one of the splicing‐related proteins in our DE proteomics, suggest that it may play a role in the development of oocytes and early embryos (Paronetto et al., 2008). Splicing factors such as PTBP1, SLU7, SRSF11, SRSF5, hnRNPM, and hnRNPA0 have also been reported to show similar age‐related changes in expression in other tissues (Bhadra et al., 2019). However, their role in the process of oocyte aging requires further research. In this study, we observed impaired blastocyst quality in reproductively aging female mice, but have yet to assess any potential follow‐up effects. Studies have shown that poor pregnancy outcomes and decreased implantation and live birth rates in women of an advanced maternal age may be due to a decrease in blastocyst quality (McCallie et al., 2019). Blastocysts from female mice at 34–39 weeks were transferred to young mothers in another study (Velazquez et al., 2016). As a result, compared with the offspring from young female mice blastocysts, the offspring from old female mice blastocysts showed abnormalities in body weight, blood pressure, glucose metabolism, and organ allometry. Since all embryos were transferred to the young mother during pregnancy to normalize the maternal *in vivo* environment, it is suggested that adverse programming via advanced maternal age is already established at the blastocyst stage.

In summary, we collected MII oocytes from three age groups to construct the proteomic expression profiles of oocyte aging after determining the phenotypes of the decline in the quality and developmental potential of aged oocytes. Bioinformatics analysis showed that the DE proteins were highly enriched in the biological process of RNA splicing, and the expression of most DE proteins was downregulated in aged oocytes. Next, we selected 2‐cell embryos from the three age groups for RNA‐seq, and splicing analysis indicated that large‐scale splicing differences in the 2‐cell stage between the reproductively aging and the younger groups. GO analysis of the DSGs in the reproductively aging groups versus the younger group suggested enrichment of biological processes related to DNA damage repair/response. Further analysis revealed that the DE protein PUF60 might be the core splicing regulatory factor causing a decline in DNA repair capacity and possibly thereby also decreased oocyte developmental potential in reproductively aging mice. The quality of early embryos was impaired after changing the splicing pattern of its potential downstream DSG *Cdk9*, which was consistent with the phenotypes of the reproductively aging groups. Our results revealed that the abnormal regulation of alternative splicing caused by changes in splicing‐related protein expression in the process of oocyte aging was one of the important reasons for the decline in the developmental potential of aged oocytes. A deeper understanding of this phenomenon is expected to provide a new perspective on the mechanism of oocyte aging, opening up a theoretical basis for establishing practical intervention measures to improve the quality of oocytes and embryos of women at advanced maternal ages to maintain female reproductive health.

## 4 EXPERIMENTAL PROCEDURES

### 4.1 Mice

C57BL/6 mice were purchased from Vital River Laboratories (Beijing, China). Previous studies (Boot et al., 1956; Finch, 2014; Nelson et al., 1981) have reported that the fertility of C57BL/6 female mice begins to decline at about 6 months of age, and the oestrus cycle is irregular after 10 months, similar to menopause in humans. Therefore, three age groups of adult female mice were used in our study: 8–10 w, 6–8 m and 10–12 m, corresponding to young maternal age, advanced maternal age, and menopause in women, respectively. Aged mice were ex‐breeders, while young controls were virgin females. The mice had free access to food and water (an ad libitum diet) throughout the period of the study. All animal care and experimental procedures were approved by Nanjing Medical University, Institutional Animal Care and Use Committee, and conducted according to the Guide for the Care and Use of Laboratory Animals.

### 4.2 Proteolytic digestion and labelling

Oocytes were lysed using urea lysis buffer (8 M urea, 75 mM NaCl, 50 mM Tris, pH 8.2, 1% (v/v) EDTA‐free protease inhibitor, 1 mM NaF, 1 mM β‐glycerophosphate, 1 mM sodium orthovanadate, 10 mM sodium pyrophosphate, 1 mM PMSF). The protein concentration was measured using the Bradford method. Proteins were reduced with DTT of 5 mM final concentration at 56℃ for 25 min and alkylated in 14 mM iodoacetamide for 30 min in the dark at room temperature. Then, the protein mixtures were diluted in 25 mM Tris‐HCl (pH 8.2) to reduce the concentration of urea to 1.6 M. Trypsin was added at a minimum concentration of 4–5 ng/μL. After digestion, the peptide mixtures were purified using an OASIS HLB 1cc Vac cartridge (Waters) and lyophilized for subsequent experiments. The 6‐plex tandem mass tag (TMT^6^) labelling was performed according to our published paper (Liu et al., 2019). Peptides from two replicates of three stages of oocytes (375 oocytes for one replicate of each stage) were labelled with respective isobaric tags and mixed together. Two labelling experiments were performed for the four replicates of oocytes at each stage.

### 4.3 High‐pH reversed phase (Hp‐RP) fractionation

TMT^6^‐labeled peptides were fractionated using a BEH C18 Column (300 μm × 150 mm, 1.7 μm; Waters) with the ACQUITY® UPLC M‐class system (Waters). Buffer A (20 mM ammonium formate, pH 10) and buffer B (100% ACN) were employed under a 128 min gradient (3% buffer B for 14 min, 3–8% B for 1 min, 8–29% B for 71 min, 29–41% B for 12 min, 41–100% B for 1 min, 100% buffer B for 8 min, 100–3% B for 1 min, followed by 20 min at 3% B). A total of 30 fractions were generated using a nonadjacent pooling scheme for each experiment and then dried with a SpeedVac concentrator.

### 4.4 LC‐MS/MS analysis

The 60 fractions from the two experiments were sequentially reconstituted in 0.1% FA and subjected to low‐pH RPLC‐MS. A trap column (75 μm × 2 cm) (Acclaim® PepMap100 C18 column, 3 μm, 100 Å; Thermo Fisher Scientific) and an analytical column (75 μm × 25 cm) (Acclaim® PepMap RSLC C18 column, 2 μm, 100 Å; Thermo Fisher Scientific) with a flow rate of 300 nl/min were operated on an Easy‐nLC 1000 system, which was coupled to a LTQ Orbitrap Velos mass spectrometer (Thermo Finnigan, San Jose, CA). MS acquisition was performed in a data‐dependent mode. The parameter settings for MS can be found in previously published papers (Castaneda et al., 2017).

### 4.5 Data processing and analysis

MaxQuant software (version 1.2.2.5) was used to search the raw files against the UniProt mouse proteome database (59,066 sequences, 2017/03). The false discovery rate (FDR) was set to 1% for both peptides and proteins. Precursor mass tolerance was set to 20 ppm and product ions were searched with a mass tolerance of 0.5 Da. Searches were performed using Trypsin/P enzyme specificity while allowing up to two missed cleavages. TMT tags on lysine residues and peptide N‐termini (+229.1629 Da) and carbamidomethylation of cysteine residues (+57.0215 Da) were set as static modifications. The oxidation of methionines and acetylation of protein N termini were investigated as variable modifications. The relative expression values for each protein were calculated by combining the MaxQuant identification results with a local modified Libra algorithm (Guo et al., 2018). One‐way analysis of variance (ANOVA) was performed using Perseus (Tyanova et al., 2016) to calculate significant differences in abundances among groups. A *p*‐value less than 0.05 and fold change greater than 1.5 were considered significant.

### 4.6 Bioinformatic analysis

The quantification values of DE proteins were normalized by z‐score. Gene ontology and KEGG pathway enrichment analysis were performed using DAVID (Huang et al., 2009). A P‐value less than 0.05 was controlled for significant enrichment. STRING was used to construct the interaction network and Cytoscape (software environment for integrated models of biomolecular interaction networks) (Shannon et al., 2003) was used to illustrate the network.

### 4.7 cDNA library construction and sequencing

The 2‐cell embryo library construction and sequencing were carried out using a total of six samples from female mice of three age groups in two separate experiments; each sample contained approximately fifty 2‐cell embryos. The MII oocyte library construction and sequencing were carried out using a total of nine samples from female mice of three age groups; each sample contained approximately fifty MII oocytes. The libraries for the MII oocytes and 2‐cell embryos were generated based on Smart‐Seq2 (Picelli et al., 2014). The KAPA HyperPlus Prep Kit (KK8514) was used to generate sequence libraries according to the manufacturer's instructions. All samples were sequenced using a HiSeq X Ten (Illumina) with paired‐end 150‐bp sequencing.

### 4.8 Identification of differentially expressed genes

The quality control of the raw RNA‐seq data is performed using FastQC and Cutadapt (Martin, 2011). Then the clean reads were mapped to the mm10 genome using STAR (v2.7.a) (Dobin et al., 2013) with default parameters. The read counts matrix is generated using HT‐seq count (Anders et al., 2015). DESeq2 (Love et al., 2014) was used to perform the differential expressed analysis between groups. The differential expressed genes are defined as genes with abs (Log2FoldChange) >1 and adjusted *p*‐value <0.05.

### 4.9 Identification of differential alternative splicing events

Raw RNA‐seq data were trimmed using Cutadapt (Martin, 2011) to remove low‐quality reads and adaptors. rMATS (v4.0.2) (Shen et al., 2014) was used to identify the differential alternative splicing events between groups ab initio. Differential alternative splicing events with FDR <0.05 were retained in the following analysis process.

### 4.10 Binding motif analysis

To identify potential splicing factors that regulate the differential alternative splicing events of skipped exons, 300‐bp flanking regions around the junction sites of exons were extracted. Each 300‐bp region included a 250‐bp region located on the intron side and a 50‐bp region located on the exon side. The 300‐bp regions were further classified into four sub‐groups based on the location: upstream exon group, upstream skipped exon group, downstream skipped exon group, and downstream exon group. Splicing factors and their motifs were collected from three databases: RBPDB (Cook et al., 2011), SpliceAid (Giulietti et al., 2013) and oRNAment (Benoit Bouvrette et al., 2020). We used CentriMo (v5.1.0) (Bailey & Machanick, 2012) to identify the enriched splicing factors in each sub‐group of 300‐bp regions with a control of random 300‐bp regions of the same number, which were generated using bedtools (Quinlan, 2014). MAST was further used to search and sort the best combined matches of the splicing factors in the splicing regions (combining evidence using *p*‐values: application to sequence homology searches).

### 4.11 Statistical analysis

All experiments were repeated at least three times. The results within each experiment are described as the mean ±SEM (standard error of mean). Statistical comparisons were performed using two‐tailed Student's *t*‐test or Chi‐square test. Statistical significance was determined as indicated in the figure legends. P‐values <0.05 were considered significant (*p < 0.05; **p < 0.01; ***p < 0.001). More details of the experimental statistics (such as sample size [*n*], description of sample collection, and the number of replicates) are indicated in the corresponding figure legends.

Other experimental procedures are detailed in Supplementary Materials.

## CONFLICT OF INTEREST

The authors declare that they have no conflict of interest.

## AUTHOR CONTRIBUTIONS

R.H., W.S., X.G., and M.L. designed the study. M.L. and C.R. performed most of the experiments and prepared the figures. S.Z., Y.L.H., Y.G., L.L., Q.C., C.W., J.H., Y.H., and X.B. performed some of the experiments. M.L., C.R., and H.Z. analyzed the data. M.L., C.R., and Y.G. wrote the manuscript. All authors have read and approved the final version of this manuscript.

## Supporting information

Figure S1

Figure S2

Figure S3

Figure S4

Table S1

Table S2

Table S3

Table S4

Table S5

Table S6

Table S7

Supplementary Material

## Data Availability

The RNA‐seq data have been deposited in the NCBI Gene Expression Omnibus database. GEO accession number: GSE 163724. The mass spectrometry proteomics data have been deposited to the ProteomeXchange Consortium via the PRIDE (Perez‐Riverol et al., 2019) partner repository with the dataset identifier PXD023603. All other data that support the findings of this study are available from the corresponding authors upon reasonable request.
