# Supplementary figures and images for: Integrative proteome analysis implicates aberrant RNA splicing in impaired developmental potential of aged mouse oocytes
Source: Aging Cell. 2021 Sep 28;20(10):e13482. doi: 10.1111/acel.13482 (PMC8520726; doi:10.1111/acel.13482)

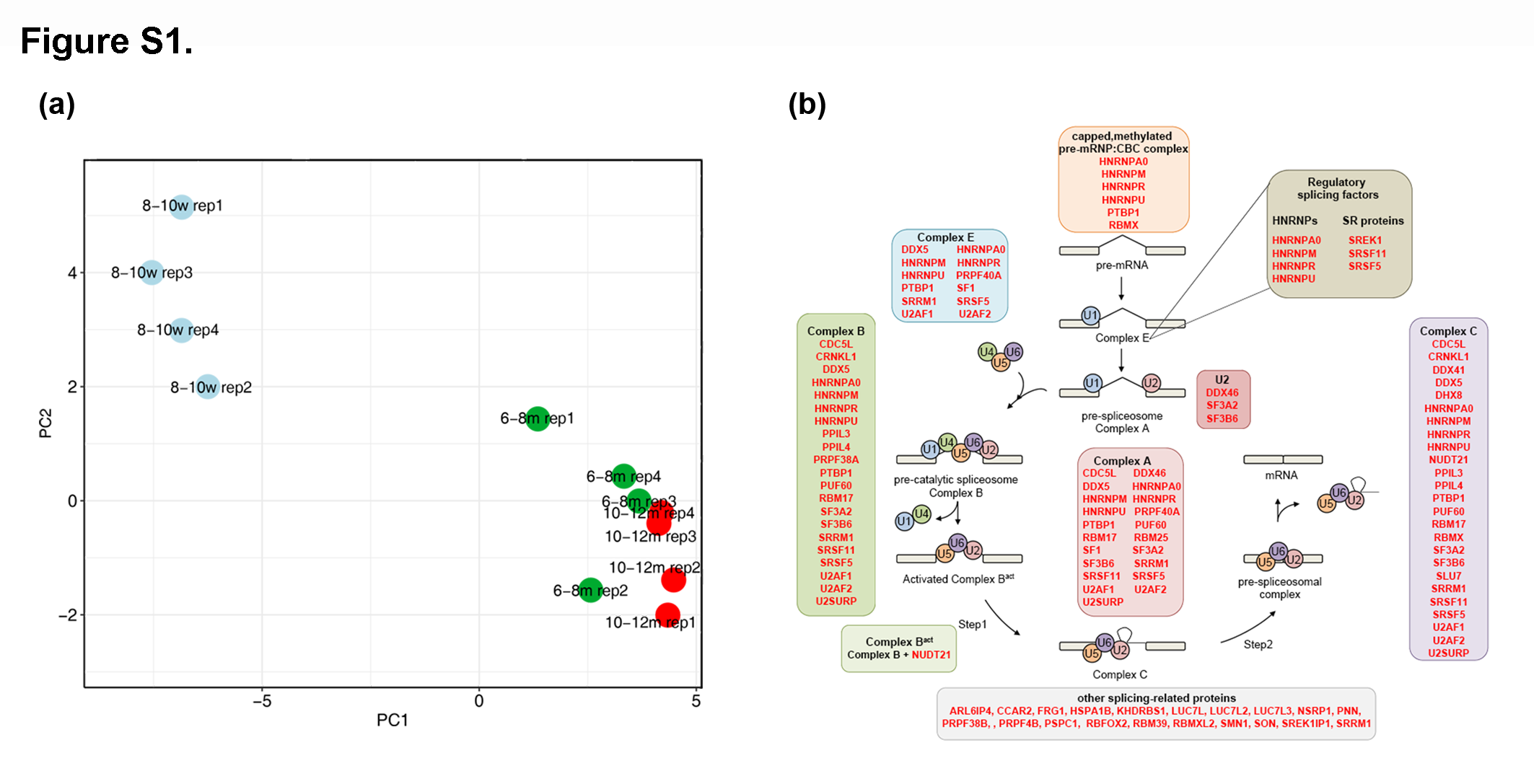

Supplement: Supplementary file 1 — Figure S1 [file ACEL-20-e13482-s001.tif]

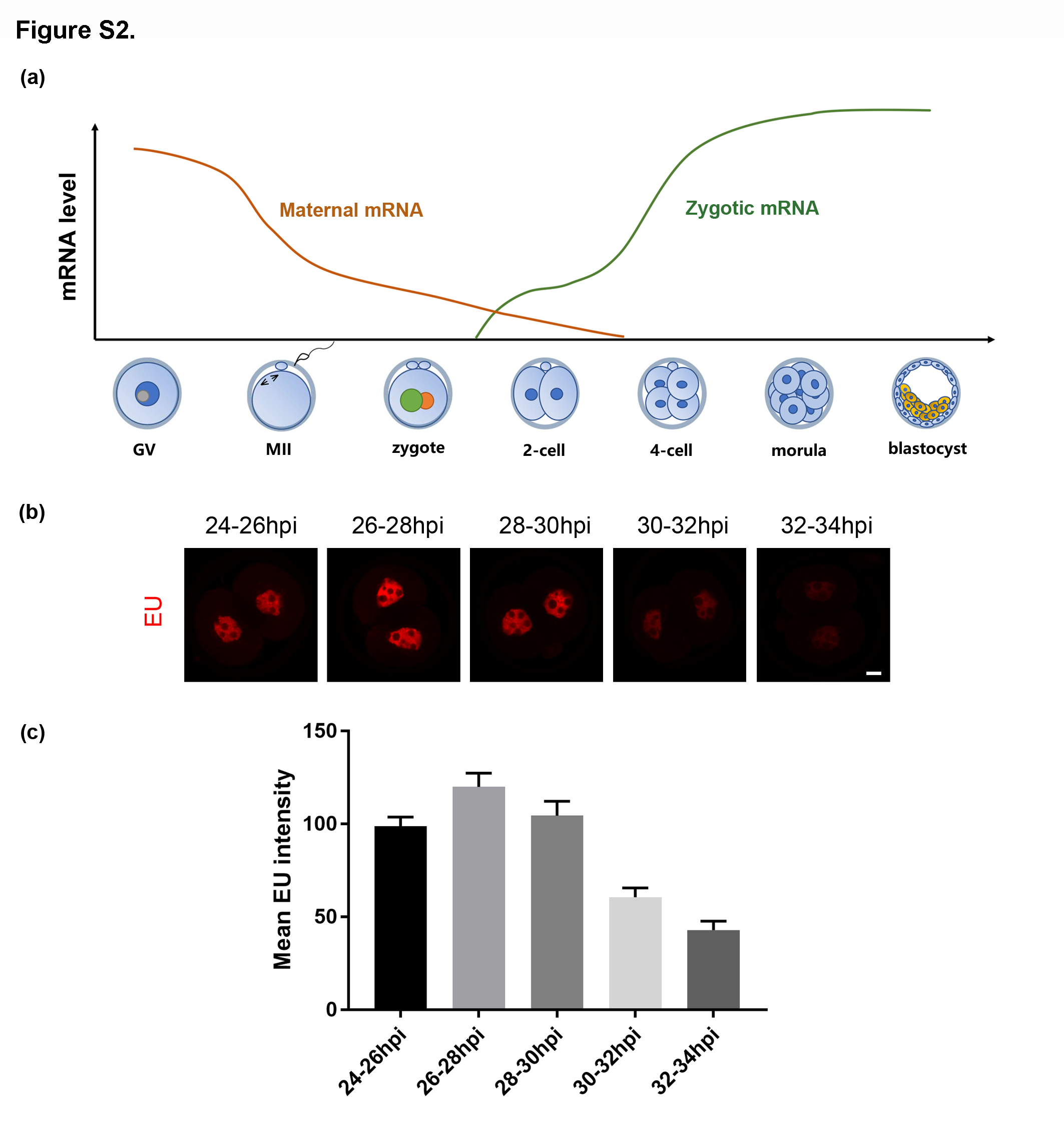

Supplement: Supplementary file 2 — Figure S2 [file ACEL-20-e13482-s012.tif]

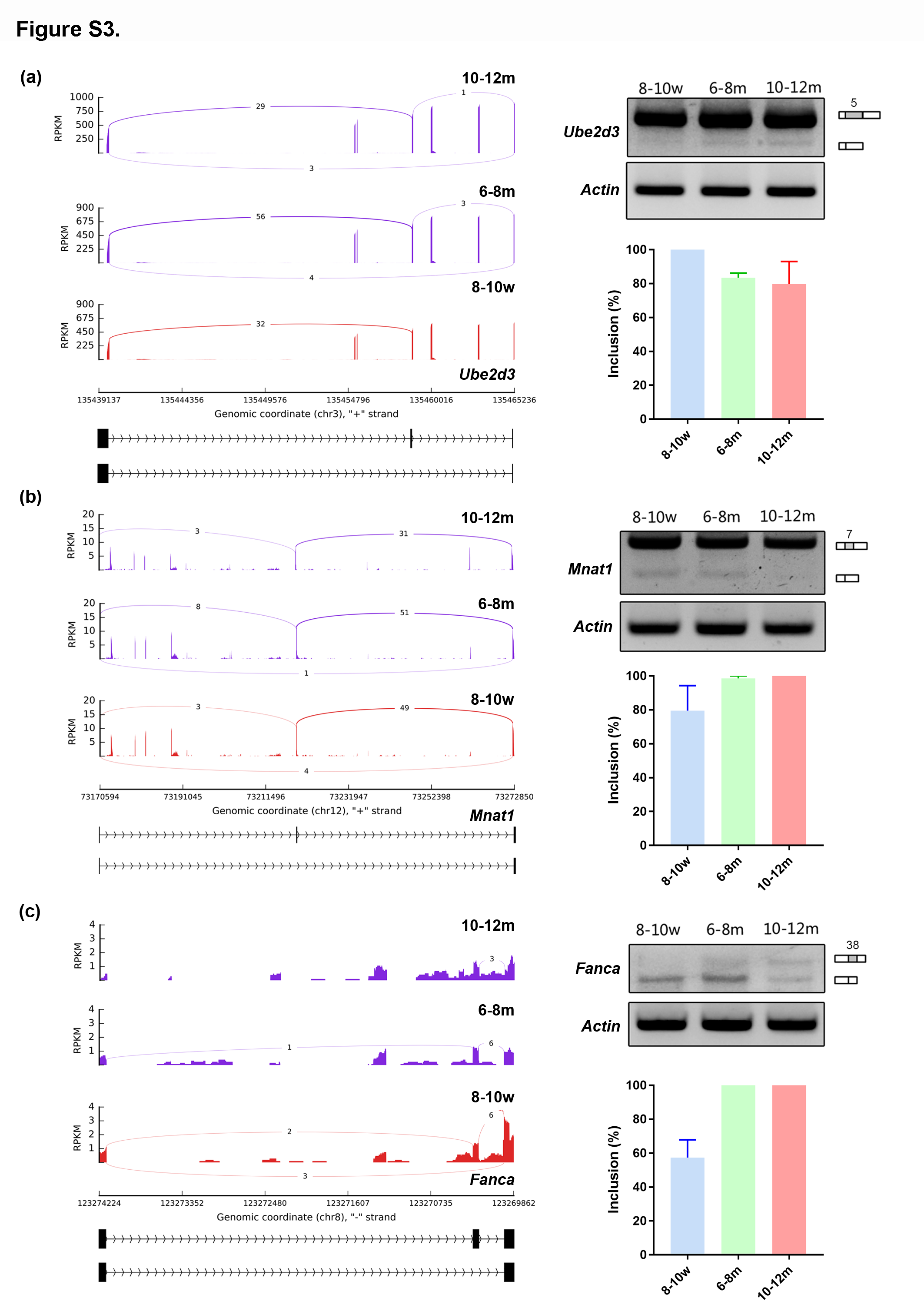

Supplement: Supplementary file 3 — Figure S3 [file ACEL-20-e13482-s003.tif]

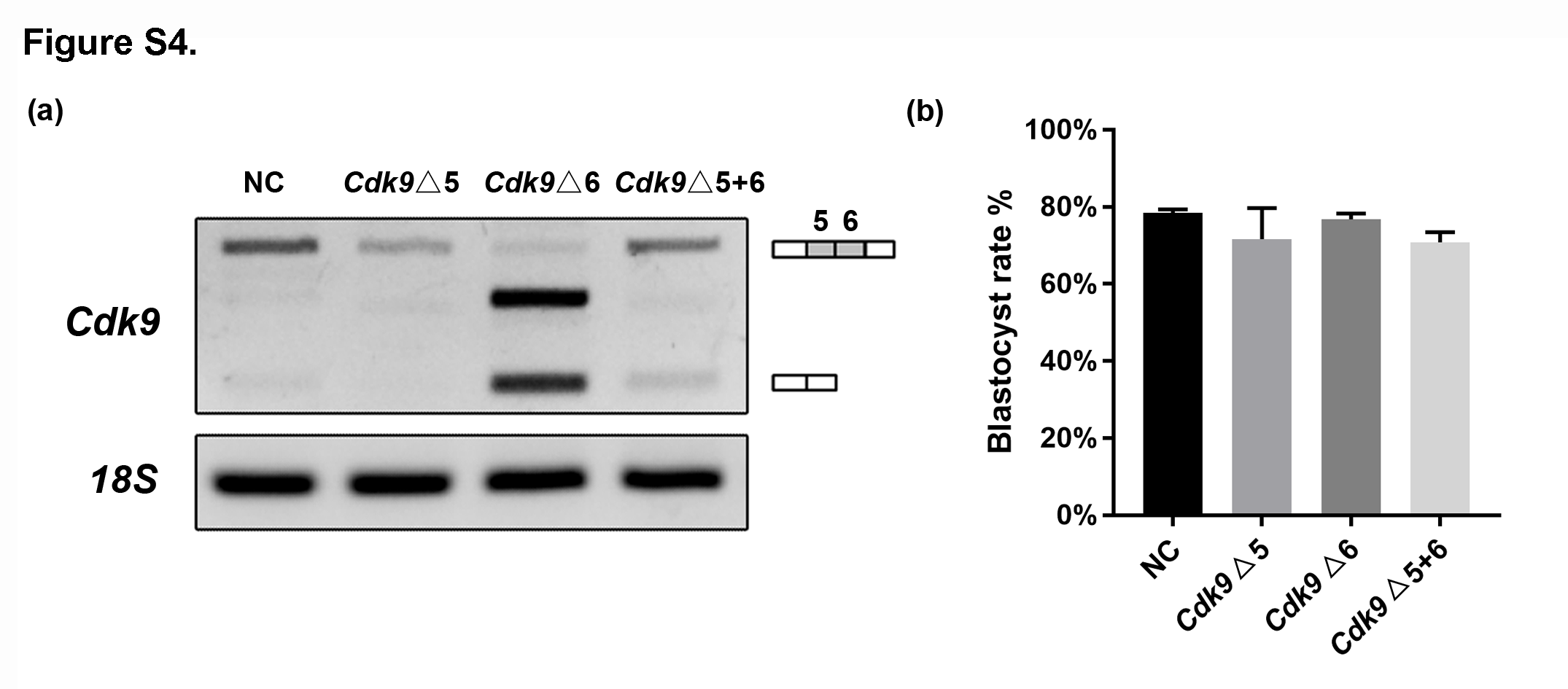

Supplement: Supplementary file 4 — Figure S4 [file ACEL-20-e13482-s009.tif]
