# Supplementary material for: Integrative proteome analysis implicates aberrant RNA splicing in impaired developmental potential of aged mouse oocytes
Source: Aging Cell. 2021 Sep 28;20(10):e13482. doi: 10.1111/acel.13482 (PMC8520726; doi:10.1111/acel.13482)
Supplement: Supplementary file 5 — Table S1 [file ACEL-20-e13482-s011.pdf]

Supplementary Table S1. List of 187 proteins that were differentially expressed in MII oocytes of three different age groups.

DE proteins	Ensembl ID	8-10w	6-8m	10-12m	Fold change
SRRM1	ENSMUSG00000028809	1.66446625	0.717138475	0.618395325	2.691589316
GOT2	ENSMUSG00000031672	1.3088345	0.998042675	0.69312265	1.88831587
ANXA2	ENSMUSG00000032231	1.5152515	0.805449675	0.679298825	2.230611101
RPSA	ENSMUSG00000032518	1.28103775	0.80731695	0.911645225	1.586784162
HSPB1	ENSMUSG00000004951	1.392276	0.8934553	0.71426865	1.949232967
SERPINH1	ENSMUSG00000070436	1.57779475	0.754806975	0.6673981	2.364098354
H2AFX	ENSMUSG00000049932	1.4121045	0.8011738	0.786721475	1.794923038
FBL	ENSMUSG00000046865	1.289542	0.900682675	0.80977535	1.592468825
HIST1H4J	ENSMUSG00000067455	1.41444725	0.80150465	0.784048125	1.804031162
HIST1H2BA	ENSMUSG00000050799	1.6117315	0.730902025	0.6573665	2.45180048
FUBP3	ENSMUSG00000026843	1.29310025	0.861465825	0.8454337	1.529511125
PUF60	ENSMUSG00000002524	1.23607	0.951499325	0.812430525	1.521447019
CBX5	ENSMUSG00000009575	1.3778955	0.8177862	0.80431825	1.713122262
HIST2H2AB	ENSMUSG00000063689	1.4358105	0.72767715	0.8365125	1.97314221
CDC5L	ENSMUSG00000023932	1.54213975	0.804985675	0.6528745	2.362076862
U2SURP	ENSMUSG00000032407	1.5312865	0.77457985	0.6941336	2.206040019
GRWD1	ENSMUSG00000053801	1.3135875	0.8255862	0.86082625	1.591096726
ERCC6L	ENSMUSG00000051220	1.33263325	0.805133975	0.862232825	1.655169564
DDX5	ENSMUSG00000020719	1.33849175	0.840319775	0.8211886	1.629944388
ACAA1B	ENSMUSG00000010651	1.37533475	0.810495725	0.8141697	1.696905619
AK6	ENSMUSG00000078941	1.265704	0.98696515	0.74733115	1.693632067
BASP1	ENSMUSG00000045763	1.3443949	1.196896975	0.458708075	2.930828937
GTPBP4	ENSMUSG00000021149	1.5037265	0.77427575	0.72199775	2.082730175
CENPV	ENSMUSG00000018509	1.40935	0.844502675	0.746147175	1.888836475
H13	ENSMUSG00000019188	1.3432585	0.718546675	0.938194825	1.869410223
CDC20	ENSMUSG00000006398	1.1987395	1.008272425	0.79298835	1.511673532
GKAP1	ENSMUSG00000021552	1.50127175	0.7900721	0.708656175	2.118476919
HDGFL3	ENSMUSG00000025104	1.3838115	0.8279945	0.78819405	1.755673619
IK	ENSMUSG00000024474	1.40575675	0.776130775	0.818112675	1.811236966
EIF2AK1	ENSMUSG00000029613	1.3886825	0.79619885	0.81511855	1.744140299
NUDT21	ENSMUSG00000031754	1.61805725	0.70930895	0.67263405	2.405553584
STK24	ENSMUSG00000063410	1.2749272	0.877227325	0.847845675	1.503725545
PIWIL1	ENSMUSG00000029423	1.28953075	0.860861175	0.849608225	1.517794569
PHF6	ENSMUSG00000025626	1.56984575	0.6769667	0.753187625	2.318940873
RBFOX2	ENSMUSG00000033565	1.320371	0.93784765	0.741781	1.780001105
CWF19L2	ENSMUSG00000025898	1.53068825	0.7642356	0.7050762	2.170954359
POLB	ENSMUSG00000031536	1.31019975	0.847482475	0.84231775	1.555469714
NCK2	ENSMUSG00000066877	1.221299	1.0980535	0.68064785	1.794318457
MATR3	ENSMUSG00000037236	1.4204745	0.782502	0.79702365	1.815298236
SERPINA1B	ENSMUSG00000071178	1.3239775	0.813999	0.8620231	1.626509983
SRSF5	ENSMUSG00000021134	1.4468385	0.78481595	0.768345275	1.883057718
WFDC15A	ENSMUSG00000051769	1.63178325	0.7244695	0.64374755	2.534818579
LUC7L3	ENSMUSG00000020863	1.4268575	0.823917775	0.749224625	1.904445546
SF3A2	ENSMUSG00000020211	1.302734	0.9211251	0.776140925	1.678476109
NLRP5	ENSMUSG00000015721	1.22945425	0.774281825	0.996264075	1.587864018
RBMXL2	ENSMUSG00000073894	1.35065325	0.931208025	0.718138925	1.880768752
DDX46	ENSMUSG00000021500	1.51586725	0.73668565	0.747447075	2.057685323
SLU7	ENSMUSG00000020409	1.516543	0.726324775	0.757132175	2.087968154
POLE3	ENSMUSG00000028394	1.22961175	0.74782625	1.022561975	1.644247912
PSPC1	ENSMUSG00000021938	1.29248875	0.92788355	0.7796275	1.657828578
DHX8	ENSMUSG00000034931	1.3211955	0.8231365	0.8556679	1.605074614
NKAP	ENSMUSG00000016409	1.6173195	0.717384025	0.66529655	2.43097533
STRBP	ENSMUSG00000026915	1.24273225	0.940464875	0.816802775	1.521459388
ILF3	ENSMUSG00000032178	1.308701	0.893609975	0.797688925	1.640615733
CCAR2	ENSMUSG00000033712	1.30346425	0.87714685	0.819388925	1.590776016

MARK3	ENSMUSG00000007411	1.28051125	0.879897475	0.83959125	1.525160309
PDHA2	ENSMUSG00000047674	1.6010435	0.6771319	0.7218245	2.364448492
CPSF6	ENSMUSG00000055531	1.578481	0.7502055	0.67131345	2.351332302
ROPN1L	ENSMUSG00000022236	1.2737645	0.92115165	0.8050838	1.582151448
NAP1L1	ENSMUSG00000058799	1.252732	0.80980155	0.937466675	1.546961722
SAP18B	ENSMUSG00000061104	1.62325225	0.67774005	0.699007525	2.395095657
CIRBP	ENSMUSG00000045193	1.3107885	0.7881209	0.9010906	1.663182007
SNX5	ENSMUSG00000027423	1.27026525	0.91348785	0.81624725	1.55622607
DDX21	ENSMUSG00000020075	1.62519575	0.688041575	0.6867627	2.366458968
PPIL3	ENSMUSG00000026035	1.46036075	0.798557725	0.7410813	1.970581028
PTBP1	ENSMUSG00000006498	1.28997175	0.835759325	0.874268925	1.543472758
MUM1	ENSMUSG00000020156	1.3653255	0.8125701	0.8221044	1.680255648
G3BP1	ENSMUSG00000018583	1.325682	0.911682	0.762636	1.738289302
PPIL4	ENSMUSG00000015757	1.598787375	0.58895775	0.8122547	2.714604528
PTGES3	ENSMUSG00000071072	1.200833	1.001468225	0.797698975	1.505371121
NSRP1	ENSMUSG00000037958	1.499999	0.751135375	0.74886575	2.003027913
SDF4	ENSMUSG00000029076	1.33089875	0.853250225	0.815851075	1.631301093
TOP1	ENSMUSG00000070544	1.4522535	0.7147493	0.832997075	2.031836198
PRPF38A	ENSMUSG00000063800	1.59931925	0.7450758	0.6556046	2.439457029
SF1	ENSMUSG00000024949	1.24503125	0.9589649	0.796003975	1.5641018
SREK1	ENSMUSG00000032621	1.448361	0.7658666	0.7857722	1.89114005
C330007P06RI	ENSMUSG00000006423	1.379403	0.83131645	0.78928075	1.747670902
MRTO4	ENSMUSG00000028741	1.45159	0.70603565	0.84237405	2.055972669
TBC1D14	ENSMUSG00000029192	1.3846455	0.85212885	0.7632256	1.814202118
PAXBP1	ENSMUSG00000022974	1.2596095	0.9015201	0.83887055	1.501554084
RPL23	ENSMUSG00000071415	1.29390575	0.8746121	0.831482375	1.556143328
HNRNPR	ENSMUSG00000066037	1.26746825	0.921760875	0.810770925	1.563287744
LARP7	ENSMUSG00000027968	1.38775425	0.835100075	0.77714565	1.785706772
SON	ENSMUSG00000022961	1.6554495	0.72084915	0.6237015	2.654233636
HMGN5	ENSMUSG00000031245	1.3137305	0.8717235	0.8145458	1.612838099
SMU1	ENSMUSG00000028409	1.30531625	0.848028475	0.846654975	1.541733396
PRPF4B	ENSMUSG00000021413	1.44988975	0.8081028	0.74200755	1.95400943
RPL38	ENSMUSG00000057322	1.2716425	0.92330385	0.805053575	1.579574999
CALB1	ENSMUSG00000028222	1.205248	1.012268375	0.78248385	1.540284825
KHDRBS1	ENSMUSG00000028790	1.363969	0.807003475	0.8290275	1.690164965
RBM17	ENSMUSG00000037197	1.2574585	0.9701997	0.772341875	1.628111256
4930550L24RI	ENSMUSG00000046180	1.3309965	0.81194095	0.857062725	1.639277462
SRI	ENSMUSG00000003161	1.21600075	1.012734275	0.771264875	1.576631828
LUC7L	ENSMUSG00000024188	1.301308	0.89859885	0.80009325	1.626445417
TWISTNB	ENSMUSG00000020561	1.26716	0.89050845	0.84233175	1.504347901
YBX3	ENSMUSG00000030189	1.49229975	0.843288925	0.664411375	2.246047865
PRPF40A	ENSMUSG00000061136	1.541506	0.75853615	0.6999578	2.202284195
RBM14	ENSMUSG00000006456	1.3349895	0.92945665	0.735553925	1.814944431
SF3B6	ENSMUSG00000037361	1.3014285	0.8563149	0.84225675	1.545168382
SREK1IP1	ENSMUSG00000021716	1.64328	0.7514392	0.6052808	2.714905214
RPS27L	ENSMUSG00000036781	1.305201	0.86223825	0.8325609	1.567694327
CLGN	ENSMUSG00000002190	1.3293565	0.8912131	0.7794305	1.705548474
HNRNPM	ENSMUSG00000059208	1.64934825	0.70558995	0.6450619	2.556883688
HNRNPU	ENSMUSG00000039630	1.37962825	0.83465465	0.785717225	1.755883931
HSPA1B	ENSMUSG00000090877	1.31858975	0.93515925	0.746250825	1.766952485
RBM3	ENSMUSG00000031167	1.329284	0.822528675	0.848187325	1.616094418
PNN	ENSMUSG00000020994	1.27501875	0.8339736	0.89100785	1.528847856
RBM25	ENSMUSG00000010608	1.55663925	0.7268809	0.71647975	2.172621417
CACYBP	ENSMUSG00000014226	1.198857	1.011938025	0.7892052	1.519068805
HIST1H1C	ENSMUSG00000036181	1.5213495	0.7960819	0.6825685	2.228859814
CFAP20	ENSMUSG00000031796	1.469441	0.8211582	0.70940065	2.07138378
FKBP6	ENSMUSG00000040013	1.3949305	0.920720875	0.68434845	2.038333688
LDHA	ENSMUSG00000063229	1.7442445	0.667401025	0.58835435	2.964615627

GSTM1	ENSMUSG00000058135	2.81748025	0.108351073	0.074168653	37.98748063
MUP18	ENSMUSG00000078674	2.39896175	0.292422143	0.30861605	8.203762306
HBB-BS	ENSMUSG00000052305	2.37371625	0.361602925	0.264680575	8.968229913
HIST1H1T	ENSMUSG00000036211	2.5759935	0.247426225	0.1765799	14.58826005
DNAJB3	ENSMUSG00000081984	2.369986	0.2978846	0.33212965	7.956054123
HBA-A1	ENSMUSG00000069919	2.40122325	0.309112425	0.2896642	8.289679049
GSTM2	ENSMUSG00000040562	2.0087215	0.44477215	0.546506	4.516293343
FRG1	ENSMUSG00000031590	1.8529945	0.628864325	0.51814145	3.576232899
RP9	ENSMUSG00000032239	1.798862	0.63479645	0.5663417	3.176283858
DDX17	ENSMUSG00000055065	1.7107935	0.713314575	0.57589215	2.970683834
ARGLU1	ENSMUSG00000040459	2.1298345	0.5189807	0.351184825	6.064711082
CEP112	ENSMUSG00000020728	1.8879635	0.6779588	0.4340777	4.349367636
DDX41	ENSMUSG00000021494	1.812764	0.666479525	0.520756625	3.481019565
HNRNPA0	ENSMUSG00000007836	1.695265	0.624450075	0.68028475	2.714812709
PGK2	ENSMUSG00000031233	2.3971985	0.34020995	0.26259155	9.129000914
RBMX	ENSMUSG00000031134	2.12889	0.4612035	0.40990645	5.193599662
LYAR	ENSMUSG00000067367	2.0947465	0.50702685	0.398226575	5.260187621
RSRC2	ENSMUSG00000029422	1.67887625	0.7118992	0.6092246	2.755759124
S100A4	ENSMUSG00000001020	1.82331375	0.63120615	0.54548005	3.342585581
SRSF11	ENSMUSG00000055436	2.4373215	0.307098	0.25558035	9.53641976
LUC7L2	ENSMUSG00000029823	1.8018915	0.624838825	0.573269875	3.143181909
VIM	ENSMUSG00000026728	2.22959375	0.41019575	0.36021015	6.189702733
RBM39	ENSMUSG00000027620	1.82841725	0.6012977	0.57028535	3.20614452
GM5096	ENSMUSG00000069324	1.9792615	0.49146155	0.529277	4.027296744
MED15	ENSMUSG00000012114	2.036728	0.50886735	0.454405	4.482186596
CYPT12	ENSMUSG00000027564	2.04516625	0.3861376	0.568696425	5.296470093
TEX101	ENSMUSG00000062773	2.1088155	0.4984939	0.3926908	5.370167827
PRPF38B	ENSMUSG00000027881	1.70557875	0.804170125	0.490251125	3.478989977
ARL6IP4	ENSMUSG00000029404	2.024988	0.51704705	0.4579648	4.421711014
U2AF2	ENSMUSG00000030435	1.8217265	0.588688225	0.589585325	3.094552299
TEX33	ENSMUSG00000062154	2.3281485	0.2970869	0.37476435	7.83659091
WDR34	ENSMUSG00000039715	1.78368125	0.640913625	0.57540535	3.099869075
INSL6	ENSMUSG00000050957	2.586814	0.1892514	0.2239345	13.66866507
LDHAL6B	ENSMUSG00000101959	2.4484745	0.30192885	0.249597	9.809711255
PPIG	ENSMUSG00000042133	2.2151085	0.47531835	0.3095731	7.15536492
WDR60	ENSMUSG00000042050	2.006912	0.5450076	0.4480808	4.478906483
HIST1H2BM	ENSMUSG00000114279	1.76889675	0.649246375	0.58185695	3.040088719
U2AF1	ENSMUSG00000061613	1.7620095	0.65694165	0.581048825	3.032463752
FAM133B	ENSMUSG00000058503	1.71767	0.725695425	0.556634225	3.085814567
IGKC	ENSMUSG00000076609	0.551648825	1.19506825	1.253283	2.271885561
EPB41	ENSMUSG00000028906	0.938396575	0.798484125	1.26311925	1.581896509
AU022751	ENSMUSG00000073294	0.7961612	0.9861866	1.217652	1.529403844
FAM241B	ENSMUSG00000020083	0.6292973	1.06764925	1.30305375	2.070648881
DCTD	ENSMUSG00000031562	0.428548025	0.48682065	2.0846315	4.864405804
GMNN	ENSMUSG00000006715	0.778479925	0.934244575	1.2872755	1.653575717
APCS	ENSMUSG00000026542	0.499957075	1.44472625	1.055316525	2.889700581
CHGA	ENSMUSG00000021194	0.7994569	1.2317435	0.9687997	1.540725335
CRNKL1	ENSMUSG00000001767	0.8006898	0.81599125	1.38331875	1.727658764
HSF5	ENSMUSG00000070345	0.838080425	0.896806125	1.2651135	1.509537107
BCAS1	ENSMUSG00000013523	0.62015575	1.2429515	1.136893	2.004256995
RNF24	ENSMUSG00000048911	0.739816625	1.5223151	0.7378686	2.063124925
GMPPA	ENSMUSG00000033021	0.7866665	1.02685255	1.186481	1.508238879
CRIP2	ENSMUSG00000006356	1.021546075	0.768071175	1.210382825	1.575873258
LDAH	ENSMUSG00000037669	0.8394438	1.2639065	0.8966497	1.505647549
SIPA1L1	ENSMUSG00000042700	0.96778255	0.79941565	1.232802	1.542128929
MRPS36	ENSMUSG00000061474	0.764567525	0.859009075	1.37642325	1.800263816
SQSTM1	ENSMUSG00000015837	0.77127	1.21019975	1.018530775	1.569099991
MFGE8	ENSMUSG00000030605	0.74366785	1.038798	1.21753425	1.637201675

KNG1	ENSMUSG00000022875	0.96192815	1.2413265	0.7967454	1.557996444
TRPS1	ENSMUSG00000038679	0.7261004	1.00947605	1.264424	1.741390034
KPNA3	ENSMUSG00000021929	0.7757848	1.02718665	1.1970285	1.542990401
SMN1	ENSMUSG00000021645	0.64178285	1.1126175	1.2455995	1.940842607
TICRR	ENSMUSG00000046591	0.71406065	0.95644615	1.3294935	1.861877559
HELQ	ENSMUSG00000035266	0.816083325	0.9334085	1.25050825	1.532329128
MYO1D	ENSMUSG00000035441	0.44977025	1.190147	1.36008275	3.023950006
KRT76	ENSMUSG00000075402	0.755585075	1.01811705	1.22629775	1.62297773
DECR1	ENSMUSG00000028223	0.669047475	1.28124175	1.0497105	1.915023669
CEACAM2	ENSMUSG00000054385	0.79215485	1.188384	1.019461	1.500191535
TSC22D2	ENSMUSG00000027806	0.68832895	1.4657175	0.8459538	2.129385231
GSTO1	ENSMUSG00000025068	0.661848525	1.012412325	1.32573925	2.0030856
NUDT16L1	ENSMUSG00000022516	0.80607535	0.77134215	1.4225825	1.844295038
TCL1B5	ENSMUSG00000000701	0.6799569	1.05126115	1.26878175	1.865973785
PDLIM5	ENSMUSG00000028273	1.222038	0.669801675	1.1081605	1.824477372
RIOK1	ENSMUSG00000021428	0.803657575	0.984698375	1.21164425	1.507662327
