# Supplementary material for: Integrative proteome analysis implicates aberrant RNA splicing in impaired developmental potential of aged mouse oocytes
Source: Aging Cell. 2021 Sep 28;20(10):e13482. doi: 10.1111/acel.13482 (PMC8520726; doi:10.1111/acel.13482)
Supplement: Supplementary file 6 — Table S2 [file ACEL-20-e13482-s005.pdf]

Supplementary Table S2. GO analysis of 187 DE proteins according to biological process

Category	Term	Count	%	P Value	Fold Enrichment	Proteins	List Total	Pop Hits	Pop Total	Bonferroni	Benjamini	FDR
GOTERM_BP_ALL	GO:0008380~RNA splicing	42	22.703	1.48E-34	13.54022251	PRPF4B, CRNKL1, U2AF2, SF3B6, NSRP1, PNN, HNRNPM, DDX46, FRG1, U2AF1, SRRM1, PPIL3, LUC7L2, SREK1IP1, ARL6IP4, DDX41, LUC7L3, CCAR2, RBM25, PRPF40A, DHX8, KHDRBS1, RBFOX2, SREK1, PTBP1, SF1, CDC5L, DDX5, SF3A2, RBMX, SMN1, HNRNPU, SRSF5, SON, PSPC1, SLU7, RBM39, LUC7L, PRPF38B, PUF60, PRPF38A, RBM17	164	342	18082	3.56E-31	3.56E-31	2.60E-31
GOTERM_BP_ALL	GO:0006397~mRNA processing	44	23.784	2.96E-33	11.49589643	PRPF4B, CRNKL1, U2AF2, SF3B6, NSRP1, PNN, HNRNPM, DDX46, FRG1, NUDT21, U2AF1, SRRM1, PPIL3, LUC7L2, SREK1IP1, ARL6IP4, DDX41, LUC7L3, CCAR2, RBM25, PRPF40A, DHX8, KHDRBS1, RBFOX2, SREK1, PTBP1, SF1, CDC5L, DDX5, SF3A2, RBMX, SMN1, HNRNPU, SRSF5, SON, PSPC1, CPSF6, SLU7, RBM39, LUC7L, PRPF38B, PUF60, PRPF38A, RBM17	164	422	18082	7.12E-30	3.56E-30	5.20E-30
GOTERM_BP_ALL	GO:0016071~mRNA metabolic process	46	24.865	1.93E-31	9.605644863	PRPF4B, CRNKL1, U2AF2, SF3B6, NSRP1, PNN, HNRNPM, DDX46, FRG1, NUDT21, U2AF1, SRRM1, PPIL3, LUC7L2, SREK1IP1, ARL6IP4, DDX41, LUC7L3, CCAR2, RBM25, PRPF40A, DHX8, KHDRBS1, RBFOX2, SREK1, PTBP1, SF1, CDC5L, DDX5, SF3A2, HNRNPR, RBMX, SMN1, HNRNPU, MRTO4, SRSF5, SON, PSPC1, CPSF6, SLU7, RBM39, LUC7L, PRPF38B, PUF60, PRPF38A, RBM17	164	528	18082	4.64E-28	1.55E-28	3.39E-28
GOTERM_BP_ALL	GO:0006396~RNA processing	52	28.108	1.21E-30	7.494532122	PRPF4B, CRNKL1, U2AF2, RBM3, U2SURP, NSRP1, PNN, U2AF1, SRRM1, LUC7L2, DDX21, SREK1IP1, CCAR2, LUC7L3, KHDRBS1, RBFOX2, GTPBP4, PTBP1, SF1, CDC5L, SMN1, HNRNPU, MRTO4, LARP7, SLU7, CPSF6, RBM39, PRPF38B, PRPF38A, SF3B6, HNRNPM, DDX46, FRG1, NUDT21, PPIL3, DDX41, ARL6IP4, RBM25, PRPF40A, DHX8, RPSA, SREK1, SF3A2, DDX5, RBMX, FBL, SON, SRSF5, PSPC1, LUC7L, PUF60, RBM17	164	765	18082	2.90E-27	7.25E-28	2.12E-27

GOTERM_BP_ALL	GO:0000377~RNA splicing, via transesterification reactions with bulged adenosine as nucleophile	27	14.595	1.14E-21	13.34939298	CRNKL1, U2AF2, SF3B6, NSRP1, HNRNPM, DDX46, U2AF1, LUC7L2, SRRM1, DDX41, RBM25, LUC7L3, PRPF40A, DHX8, RBFOX2, SREK1, PTBP1, SF1, CDC5L, DDX5, SF3A2, RBMX, SMN1, PSPC1, SLU7, LUC7L, RBM17	164	223	18082	2.75E-18	5.49E-19	2.01E-18
GOTERM_BP_ALL	GO:0000398~mRNA splicing, via spliceosome	27	14.595	1.14E-21	13.34939298	CRNKL1, U2AF2, SF3B6, NSRP1, HNRNPM, DDX46, U2AF1, LUC7L2, SRRM1, DDX41, RBM25, LUC7L3, PRPF40A, DHX8, RBFOX2, SREK1, PTBP1, SF1, CDC5L, DDX5, SF3A2, RBMX, SMN1, PSPC1, SLU7, LUC7L, RBM17	164	223	18082	2.75E-18	5.49E-19	2.01E-18
GOTERM_BP_ALL	GO:0000375~RNA splicing, via transesterification reactions	27	14.595	1.28E-21	13.28979747	CRNKL1, U2AF2, SF3B6, NSRP1, HNRNPM, DDX46, U2AF1, LUC7L2, SRRM1, DDX41, RBM25, LUC7L3, PRPF40A, DHX8, RBFOX2, SREK1, PTBP1, SF1, CDC5L, DDX5, SF3A2, RBMX, SMN1, PSPC1, SLU7, LUC7L, RBM17	164	224	18082	3.08E-18	5.14E-19	2.25E-18
GOTERM_BP_ALL	GO:0010467~gene expression	94	50.811	4.74E-14	1.983934374	PTGES3, NKAP, PRPF4B, CRNKL1, U2AF2, RBM3, U2SURP, YBX3, RPS27L, NSRP1, HMGN5, CBX5, PNN, HIST2H2AB, TOP1, DDX17, FUBP3, H13, HSF5, U2AF1, MUP18, PIWIL1, SRRM1, LUC7L2, H2AFX, DDX21, TWISTNB, SREK1IP1, LUC7L3, CCAR2, KHDRBS1, RBFOX2, NLRP5, GTPBP4, HIST1H1C, PTBP1, SF1, BASP1, CDC5L, HNRNPR, HNRNPU, SMN1, MRTO4, NCK2, EIF2AK1, MED15, LARP7, SLU7, CPSF6, HBB-BS, RBM39, MATR3, PRPF38B, PRPF38A, FKBP6, TICRR, VIM, RPL38, PAXBP1, SF3B6, SERPINH1, HNRNPM, DDX46, FRG1, SQSTM1, NUDT21, PPIL3, ARL6IP4, DDX41, HIST1H4J, RBM25, PRPF40A, DHX8, HIST1H2BA, RPSA, SREK1, GMNN, ILF3, DDX5, SF3A2, RBMX, HNRNPA0, FBL, SRSF5, SON, RPL23, TRPS1, PSPC1, CIRBP, LUC7L, RBM14, PUF60, RBM17, PHF6	164	5224	18082	1.14E-10	1.63E-11	8.33E-11

GOTERM_BP_ALL	GO:0034641~cellular nitrogen compound metabolic process	100	54.054	4.00E-12	1.778897992	LDHA, PRPF4B, U2AF2, RBM3, RPS27L, NSRP1, HMGN5, PNN, CBX5, GSTM1, TOP1, GSTM2, DDX17, H13, U2AF1, SRRM1, LUC7L2, DDX21, H2AFX, PDHA2, SREK1IP1, CCAR2, LUC7L3, RBFOX2, GTPBP4, PTBP1, POLB, BASP1, HNRNPR, HNRNPU, MRTO4, EIF2AK1, MED15, SLU7, HBB-BS, TICRR, MUM1, HNRNPM, DDX46, FRG1, CACYBP, PPIL3, ARL6IP4, DDX41, HIST1H4J, PRPF40A, DHX8, RPSA, SREK1, DDX5, RBMX, AK6, SRSF5, TRPS1, CIRBP, PUF60, PHF6, NKAP, HELQ, CRNKL1, U2SURP, YBX3, HIST2H2AB, FUBP3, HSF5, MUP18, PIWIL1, TWISTNB, KHDRBS1, HIST1H1C, SF1, CDC5L, SMN1, NCK2, CHGA, LARP7, CPSF6, RBM39, PRPF38B, PRPF38A, FKBP6, PAXBP1, SF3B6, RPL38, SQSTM1, NUDT21, GSTO1, RBM25, DCTD, GMNN, ILF3, SF3A2, FBL, SON, RPL23, PSPC1, RBM14, LUC7L, PGK2, RBM17	164	6198	18082	9.61E-09	1.20E-09	7.02E-09
GOTERM_BP_ALL	GO:0006725~cellular aromatic compound metabolic process	93	50.27	8.05E-12	1.844211704	NKAP, LDHA, HELQ, PRPF4B, CRNKL1, U2AF2, RBM3, U2SURP, YBX3, RPS27L, NSRP1, HMGN5, CBX5, PNN, HIST2H2AB, GSTM2, TOP1, DDX17, FUBP3, HSF5, U2AF1, MUP18, PIWIL1, SRRM1, LUC7L2, H2AFX, DDX21, PDHA2, TWISTNB, SREK1IP1, LUC7L3, CCAR2, KHDRBS1, RBFOX2, GTPBP4, HIST1H1C, PTBP1, SF1, POLB, BASP1, CDC5L, HNRNPR, HNRNPU, SMN1, MRTO4, NCK2, CHGA, EIF2AK1, MED15, LARP7, SLU7, CPSF6, RBM39, PRPF38B, PRPF38A, FKBP6, TICRR, MUM1, PAXBP1, SF3B6, HNRNPM, DDX46, FRG1, SQSTM1, CACYBP, NUDT21, PPIL3, ARL6IP4, DDX41, HIST1H4J, RBM25, PRPF40A, DHX8, DCTD, RPSA, SREK1, GMNN, ILF3, DDX5, SF3A2, AK6, RBMX, FBL, SRSF5, SON, TRPS1, PSPC1, LUC7L, RBM14, PUF60, PGK2, RBM17, PHF6	164	5560	18082	1.93E-08	2.15E-09	1.41E-08
GOTERM_BP_ALL	GO:0090304~nucleic acid metabolic process	85	45.946	9.10E-12	1.95042004	NKAP, HELQ, PRPF4B, CRNKL1, U2AF2, RBM3, U2SURP, YBX3, RPS27L, NSRP1, HMGN5, CBX5, PNN, HIST2H2AB, TOP1, DDX17, FUBP3, HSF5, U2AF1, MUP18, PIWIL1, SRRM1, LUC7L2, H2AFX, DDX21, TWISTNB, SREK1IP1, LUC7L3, CCAR2, KHDRBS1, RBFOX2, GTPBP4, HIST1H1C, PTBP1, SF1, POLB, BASP1, CDC5L, HNRNPR, HNRNPU, SMN1, MRTO4, NCK2, MED15, LARP7, SLU7, CPSF6, RBM39, PRPF38B, PRPF38A, FKBP6, TICRR, MUM1, PAXBP1, SF3B6, HNRNPM, DDX46, FRG1, SQSTM1, CACYBP, NUDT21, PPIL3, ARL6IP4, DDX41, HIST1H4J, RBM25, PRPF40A, DHX8, RPSA, SREK1, GMNN, ILF3, DDX5, SF3A2, RBMX, FBL, SRSF5, SON, TRPS1, PSPC1, LUC7L, RBM14, PUF60, RBM17, PHF6	164	4805	18082	2.19E-08	2.19E-09	1.60E-08

GOTERM_BP_ALL	GO:0006139~nucleobase-containing compound metabolic process	91	49.189	1.06E-11	1.861812002	NKAP, LDHA, HELQ, PRPF4B, CRNKL1, U2AF2, RBM3, U2SURP, YBX3, RPS27L, NSRP1, HMGN5, CBX5, PNN, HIST2H2AB, TOP1, DDX17, FUBP3, HSF5, U2AF1, MUP18, PIWIL1, SRRM1, LUC7L2, H2AFX, DDX21, PDHA2, TWISTNB, SREK1IP1, LUC7L3, CCAR2, KHDRBS1, RBFOX2, GTPBP4, HIST1H1C, PTBP1, SF1, POLB, BASP1, CDC5L, HNRNPR, HNRNPU, SMN1, MRTO4, NCK2, CHGA, MED15, LARP7, SLU7, CPSF6, RBM39, PRPF38B, PRPF38A, FKBP6, TICRR, MUM1, PAXBP1, SF3B6, HNRNPM, DDX46, FRG1, SQSTM1, CACYBP, NUDT21, PPIL3, ARL6IP4, DDX41, HIST1H4J, RBM25, PRPF40A, DHX8, DCTD, RPSA, SREK1, GMNN, ILF3, DDX5, SF3A2, AK6, RBMX, FBL, SRSF5, SON, TRPS1, PSPC1, LUC7L, RBM14, PUF60, PGK2, RBM17, PHF6	164	5389	18082	2.54E-08	2.31E-09	1.85E-08
GOTERM_BP_ALL	GO:0046483~heterocycle metabolic process	92	49.73	1.19E-11	1.845290336	NKAP, LDHA, HELQ, PRPF4B, CRNKL1, U2AF2, RBM3, U2SURP, YBX3, RPS27L, NSRP1, HMGN5, CBX5, PNN, HIST2H2AB, TOP1, DDX17, FUBP3, HSF5, U2AF1, MUP18, PIWIL1, SRRM1, LUC7L2, H2AFX, DDX21, PDHA2, TWISTNB, SREK1IP1, LUC7L3, CCAR2, KHDRBS1, RBFOX2, GTPBP4, HIST1H1C, PTBP1, SF1, POLB, BASP1, CDC5L, HNRNPR, HNRNPU, SMN1, MRTO4, NCK2, CHGA, EIF2AK1, MED15, LARP7, SLU7, CPSF6, RBM39, PRPF38B, PRPF38A, FKBP6, TICRR, MUM1, PAXBP1, SF3B6, HNRNPM, DDX46, FRG1, SQSTM1, CACYBP, NUDT21, PPIL3, ARL6IP4, DDX41, HIST1H4J, RBM25, PRPF40A, DHX8, DCTD, RPSA, SREK1, GMNN, ILF3, DDX5, SF3A2, AK6, RBMX, FBL, SRSF5, SON, TRPS1, PSPC1, LUC7L, RBM14, PUF60, PGK2, RBM17, PHF6	164	5497	18082	2.85E-08	2.38E-09	2.09E-08
GOTERM_BP_ALL	GO:0016070~RNA metabolic process	79	42.703	2.27E-11	2.011136391	NKAP, PRPF4B, CRNKL1, U2AF2, RBM3, U2SURP, YBX3, NSRP1, HMGN5, CBX5, PNN, HIST2H2AB, TOP1, DDX17, FUBP3, HSF5, U2AF1, MUP18, PIWIL1, SRRM1, LUC7L2, H2AFX, DDX21, TWISTNB, SREK1IP1, LUC7L3, CCAR2, KHDRBS1, RBFOX2, GTPBP4, HIST1H1C, PTBP1, SF1, BASP1, CDC5L, HNRNPR, HNRNPU, SMN1, MRTO4, NCK2, MED15, LARP7, SLU7, CPSF6, RBM39, PRPF38B, PRPF38A, FKBP6, PAXBP1, SF3B6, HNRNPM, DDX46, FRG1, SQSTM1, NUDT21, PPIL3, ARL6IP4, DDX41, HIST1H4J, RBM25, PRPF40A, DHX8, RPSA, SREK1, GMNN, ILF3, DDX5, SF3A2, RBMX, FBL, SON, SRSF5, TRPS1, PSPC1, LUC7L, RBM14, PUF60, RBM17, PHF6	164	4331	18082	5.45E-08	4.20E-09	3.99E-08

GOTERM_BP_ALL	GO:0006807~nitrogen compound metabolic process	101	54.595	4.68E-11	1.70351321	LDHA, PRPF4B, U2AF2, RBM3, RPS27L, NSRP1, HMGN5, PNN, CBX5, GOT2, GSTM1, TOP1, GSTM2, DDX17, H13, U2AF1, SRRM1, LUC7L2, DDX21, H2AFX, PDHA2, SREK1IP1, CCAR2, LUC7L3, RBFOX2, GTPBP4, PTBP1, POLB, BASP1, HNRNPR, HNRNPU, MRTO4, EIF2AK1, MED15, SLU7, HBB-BS, TICRR, MUM1, HNRNPM, DDX46, FRG1, CACYBP, PPIL3, ARL6IP4, DDX41, HIST1H4J, PRPF40A, DHX8, RPSA, SREK1, DDX5, RBMX, AK6, SRSF5, TRPS1, CIRBP, PUF60, PHF6, NKAP, HELQ, CRNKL1, U2SURP, YBX3, HIST2H2AB, FUBP3, HSF5, MUP18, PIWIL1, TWISTNB, KHDRBS1, HIST1H1C, SF1, CDC5L, SMN1, NCK2, CHGA, LARP7, CPSF6, RBM39, PRPF38B, PRPF38A, FKBP6, PAXBP1, SF3B6, RPL38, SQSTM1, NUDT21, GSTO1, RBM25, DCTD, GMNN, ILF3, SF3A2, FBL, SON, RPL23, PSPC1, RBM14, LUC7L, PGK2, RBM17	164	6537	18082	1.12E-07	8.03E-09	8.21E-08
GOTERM_BP_ALL	GO:1901360~organic cyclic compound metabolic process	93	50.27	5.13E-11	1.789184623	NKAP, LDHA, HELQ, PRPF4B, CRNKL1, U2AF2, RBM3, U2SURP, YBX3, RPS27L, NSRP1, HMGN5, CBX5, PNN, HIST2H2AB, GSTM2, TOP1, DDX17, FUBP3, HSF5, U2AF1, MUP18, PIWIL1, SRRM1, LUC7L2, H2AFX, DDX21, PDHA2, TWISTNB, SREK1IP1, LUC7L3, CCAR2, KHDRBS1, RBFOX2, GTPBP4, HIST1H1C, PTBP1, SF1, POLB, BASP1, CDC5L, HNRNPR, HNRNPU, SMN1, MRTO4, NCK2, CHGA, EIF2AK1, MED15, LARP7, SLU7, CPSF6, RBM39, PRPF38B, PRPF38A, FKBP6, TICRR, MUM1, PAXBP1, SF3B6, HNRNPM, DDX46, FRG1, SQSTM1, CACYBP, NUDT21, PPIL3, ARL6IP4, DDX41, HIST1H4J, RBM25, PRPF40A, DHX8, DCTD, RPSA, SREK1, GMNN, ILF3, DDX5, SF3A2, AK6, RBMX, FBL, SRSF5, SON, TRPS1, PSPC1, LUC7L, RBM14, PUF60, PGK2, RBM17, PHF6	164	5731	18082	1.23E-07	8.23E-09	9.02E-08

GOTERM_BP_ALL	GO:0008152~metabolic process	134	72.432	3.04E-09	1.357933555	MRPS36, TEX101, LDHA, PRPF4B, U2AF2, RBM3, RPS27L, NSRP1, HMGN5, CBX5, PNN, GSTM1, GOT2, GSTM2, TOP1, DDX17, H13, U2AF1, LUC7L2, SRRM1, H2AFX, DDX21, PDHA2, SREK1IP1, LUC7L3, CCAR2, RBFOX2, GTPBP4, STK24, PTBP1, POLB, BASP1, MARK3, HNRNPR, HNRNPU, MRTO4, EIF2AK1, MED15, HSPB1, RNF24, SLU7, HBB-BS, SRI, TICRR, MUM1, HSPA1B, RIOK1, SERPINH1, HNRNPM, DDX46, SERPINA1B, POLE3, FRG1, GMPPA, CACYBP, PPIL4, PPIL3, IGKC, ARL6IP4, DDX41, HIST1H4J, PRPF40A, DHX8, RPSA, SREK1, MYO1D, CDC20, DDX5, AK6, RBMX, HNRNPA0, SRSF5, PPIG, TRPS1, CIRBP, PUF60, PHF6, PTGES3, NKAP, HELQ, CRNKL1, U2SURP, YBX3, HIST2H2AB, FUBP3, MUP18, PIWIL1, HSF5, TWISTNB, KHDRBS1, KNG1, NLRP5, APCS, HIST1H1C, LDHAL6B, SF1, DECR1, CDC5L, SMN1, NCK2, CHGA, LARP7, CFAP20, CPSF6, RBM39, PRPF38B, MATR3, PRPF38A, FKBP6, VIM, RPL38, PAXBP1, SF3B6, ROPN1L, SQSTM1, NUDT21, GSTO1, ACAA1B, RBM25, HIST1H2BA, DCTD, GMNN, ILF3, SF3A2, FBL, ANXA2, SON, RPL23, CENPV, PSPC1, LUC7L, RBM14, PGK2, RBM17	164	10880	18082	7.31E-06	4.57E-07	5.34E-06
GOTERM_BP_ALL	GO:0043484~regulation of RNA splicing	12	6.4865	4.50E-09	12.02793792	KHDRBS1, RBFOX2, SON, SRSF5, U2AF2, SREK1, PTBP1, NSRP1, DDX5, RBMX, RBM25, SMN1	164	110	18082	1.08E-05	6.36E-07	7.90E-06

GOTERM_BP_ALL	GO:0071704~organic substance metabolic process	130	70.27	6.16E-09	1.372263541	MRPS36, TEX101, LDHA, PRPF4B, U2AF2, RBM3, RPS27L, NSRP1, HMGN5, CBX5, PNN, GSTM1, GOT2, GSTM2, TOP1, DDX17, H13, U2AF1, LUC7L2, SRRM1, H2AFX, DDX21, PDHA2, SREK1IP1, LUC7L3, CCAR2, RBFOX2, GTPBP4, STK24, PTBP1, POLB, BASP1, MARK3, HNRNPR, HNRNPU, MRTO4, EIF2AK1, MED15, HSPB1, RNF24, SLU7, HBB-BS, SRI, TICRR, MUM1, HSPA1B, SERPINH1, HNRNPM, DDX46, SERPINA1B, POLE3, FRG1, CACYBP, PPIL4, PPIL3, IGKC, ARL6IP4, DDX41, HIST1H4J, PRPF40A, DHX8, RPSA, SREK1, CDC20, DDX5, AK6, RBMX, HNRNPA0, SRSF5, PPIG, TRPS1, CIRBP, PUF60, PHF6, PTGES3, NKAP, HELQ, CRNKL1, U2SURP, YBX3, HIST2H2AB, FUBP3, MUP18, PIWIL1, HSF5, TWISTNB, KHDRBS1, KNG1, NLRP5, APCS, HIST1H1C, LDHAL6B, SF1, DECR1, CDC5L, SMN1, NCK2, CHGA, LARP7, CFAP20, CPSF6, RBM39, PRPF38B, MATR3, PRPF38A, FKBP6, VIM, RPL38, PAXBP1, SF3B6, ROPN1L, SQSTM1, NUDT21, GSTO1, ACAA1B, RBM25, HIST1H2BA, DCTD, GMNN, ILF3, SF3A2, FBL, ANXA2, SON, RPL23, PSPC1, LUC7L, RBM14, PGK2, RBM17	164	10445	18082	1.48E-05	8.23E-07	1.08E-05
GOTERM_BP_ALL	GO:0044237~cellular metabolic process	124	67.027	1.40E-08	1.395931805	MRPS36, TEX101, LDHA, PRPF4B, U2AF2, RBM3, RPS27L, NSRP1, HMGN5, CBX5, PNN, GSTM1, GOT2, GSTM2, TOP1, DDX17, H13, U2AF1, LUC7L2, SRRM1, H2AFX, DDX21, PDHA2, SREK1IP1, LUC7L3, CCAR2, RBFOX2, GTPBP4, STK24, PTBP1, POLB, BASP1, MARK3, HNRNPR, HNRNPU, MRTO4, EIF2AK1, MED15, HSPB1, RNF24, SLU7, HBB-BS, TICRR, MUM1, HSPA1B, RIOK1, HNRNPM, DDX46, SERPINA1B, POLE3, FRG1, CACYBP, PPIL4, PPIL3, ARL6IP4, DDX41, HIST1H4J, PRPF40A, DHX8, RPSA, SREK1, MYO1D, CDC20, DDX5, AK6, RBMX, SRSF5, PPIG, TRPS1, CIRBP, PUF60, PHF6, PTGES3, NKAP, HELQ, CRNKL1, U2SURP, YBX3, HIST2H2AB, FUBP3, MUP18, PIWIL1, HSF5, TWISTNB, KHDRBS1, KNG1, APCS, HIST1H1C, LDHAL6B, SF1, DECR1, CDC5L, SMN1, NCK2, CHGA, LARP7, CFAP20, CPSF6, RBM39, PRPF38B, PRPF38A, FKBP6, PAXBP1, SF3B6, RPL38, ROPN1L, SQSTM1, NUDT21, GSTO1, ACAA1B, RBM25, DCTD, GMNN, ILF3, SF3A2, FBL, ANXA2, SON, RPL23, PSPC1, LUC7L, RBM14, PGK2, RBM17	164	9794	18082	3.36E-05	1.77E-06	2.45E-05

GOTERM_BP_ALL	GO:0043170~macromolecule metabolic process	116	62.703	2.10E-08	1.439309849	TEX101, PRPF4B, U2AF2, RBM3, RPS27L, NSRP1, HMGN5, CBX5, PNN, TOP1, DDX17, H13, U2AF1, LUC7L2, SRRM1, H2AFX, DDX21, SREK1IP1, LUC7L3, CCAR2, RBFOX2, GTPBP4, STK24, PTBP1, POLB, BASP1, MARK3, HNRNPR, HNRNPU, MRTO4, EIF2AK1, MED15, HSPB1, RNF24, SLU7, HBB-BS, SRI, TICRR, MUM1, HSPA1B, SERPINH1, HNRNPM, DDX46, SERPINA1B, POLE3, FRG1, CACYBP, PPIL4, PPIL3, IGKC, ARL6IP4, DDX41, HIST1H4J, PRPF40A, DHX8, RPSA, SREK1, CDC20, DDX5, RBMX, HNRNPA0, SRSF5, PPIG, TRPS1, CIRBP, PUF60, PHF6, PTGES3, NKAP, HELQ, CRNKL1, U2SURP, YBX3, HIST2H2AB, FUBP3, MUP18, PIWIL1, HSF5, TWISTNB, KHDRBS1, KNG1, NLRP5, APCS, HIST1H1C, SF1, CDC5L, SMN1, NCK2, LARP7, CFAP20, CPSF6, RBM39, PRPF38B, MATR3, PRPF38A, FKBP6, VIM, PAXBP1, SF3B6, RPL38, ROPN1L, SQSTM1, NUDT21, RBM25, HIST1H2BA, GMNN, ILF3, SF3A2, FBL, ANXA2, SON, RPL23, PSPC1, LUC7L, RBM14, RBM17	164	8886	18082	5.04E-05	2.52E-06	3.69E-05
GOTERM_BP_ALL	GO:0048024~regulation of mRNA splicing, via spliceosome	10	5.4054	2.11E-08	14.89947264	KHDRBS1, RBFOX2, SON, U2AF2, SREK1, PTBP1, NSRP1, DDX5, RBMX, RBM25	164	74	18082	5.07E-05	2.41E-06	3.70E-05
GOTERM_BP_ALL	GO:0044260~cellular macromolecule metabolic process	108	58.378	3.60E-08	1.481420569	TEX101, PRPF4B, U2AF2, RBM3, RPS27L, NSRP1, HMGN5, CBX5, PNN, TOP1, DDX17, H13, U2AF1, SRRM1, LUC7L2, H2AFX, DDX21, SREK1IP1, LUC7L3, CCAR2, RBFOX2, GTPBP4, STK24, PTBP1, POLB, BASP1, MARK3, HNRNPR, HNRNPU, MRTO4, EIF2AK1, MED15, HSPB1, RNF24, SLU7, HBB-BS, TICRR, MUM1, HSPA1B, HNRNPM, DDX46, SERPINA1B, FRG1, POLE3, CACYBP, PPIL4, PPIL3, ARL6IP4, DDX41, HIST1H4J, PRPF40A, DHX8, RPSA, SREK1, CDC20, DDX5, RBMX, SRSF5, PPIG, TRPS1, CIRBP, PUF60, PHF6, PTGES3, NKAP, HELQ, CRNKL1, U2SURP, YBX3, HIST2H2AB, FUBP3, HSF5, MUP18, PIWIL1, TWISTNB, KHDRBS1, KNG1, APCS, HIST1H1C, SF1, CDC5L, SMN1, NCK2, LARP7, CFAP20, CPSF6, RBM39, PRPF38B, PRPF38A, FKBP6, PAXBP1, SF3B6, RPL38, ROPN1L, SQSTM1, NUDT21, RBM25, GMNN, ILF3, SF3A2, FBL, ANXA2, SON, RPL23, PSPC1, LUC7L, RBM14, RBM17	164	8038	18082	8.65E-05	3.93E-06	6.33E-05

GOTERM_BP_ALL	GO:0022618~ribonucleoprotein complex assembly	14	7.5676	5.59E-08	7.385575913	RPSA, CRNKL1, TICRR, RPS27L, RPL38, SF3A2, RBMX, SMN1, MRTO4, LUC7L2, CIRBP, SLU7, LUC7L, LUC7L3	164	209	18082	1.34E-04	5.84E-06	9.82E-05
GOTERM_BP_ALL	GO:0071826~ribonucleoprotein complex subunit organization	14	7.5676	1.02E-07	7.016297118	RPSA, CRNKL1, TICRR, RPS27L, RPL38, SF3A2, RBMX, SMN1, MRTO4, LUC7L2, CIRBP, SLU7, LUC7L, LUC7L3	164	220	18082	2.44E-04	1.02E-05	1.78E-04
GOTERM_BP_ALL	GO:0022613~ribonucleoprotein complex biogenesis	18	9.7297	2.28E-07	4.770696529	RPSA, GTPBP4, CRNKL1, TICRR, RPS27L, RPL38, SF3A2, RBMX, FBL, SMN1, MRTO4, FRG1, LUC7L2, CIRBP, SLU7, DDX21, LUC7L, LUC7L3	164	416	18082	5.47E-04	2.19E-05	4.00E-04
GOTERM_BP_ALL	GO:0044238~primary metabolic process	121	65.405	3.46E-07	1.354002619	TEX101, LDHA, PRPF4B, U2AF2, RBM3, RPS27L, NSRP1, HMGN5, CBX5, PNN, GOT2, TOP1, DDX17, H13, U2AF1, LUC7L2, SRRM1, H2AFX, DDX21, PDHA2, SREK1IP1, LUC7L3, CCAR2, RBFOX2, GTPBP4, STK24, PTBP1, POLB, BASP1, MARK3, HNRNPR, HNRNPU, MRTO4, EIF2AK1, MED15, HSPB1, RNF24, SLU7, HBB-BS, SRI, TICRR, MUM1, HSPA1B, SERPINH1, HNRNPM, DDX46, SERPINA1B, POLE3, FRG1, CACYBP, PPIL4, PPIL3, IGKC, ARL6IP4, DDX41, HIST1H4J, PRPF40A, DHX8, RPSA, SREK1, CDC20, DDX5, AK6, RBMX, SRSF5, PPIG, TRPS1, CIRBP, PUF60, PHF6, PTGES3, NKAP, HELQ, CRNKL1, U2SURP, YBX3, HIST2H2AB, FUBP3, MUP18, PIWIL1, HSF5, TWISTNB, KHDRBS1, KNG1, HIST1H1C, LDHAL6B, SF1, DECR1, CDC5L, SMN1, NCK2, CHGA, LARP7, CFAP20, CPSF6, RBM39, PRPF38B, PRPF38A, FKBP6, PAXBP1, SF3B6, RPL38, ROPN1L, SQSTM1, NUDT21, ACAA1B, RBM25, DCTD, HIST1H2BA, GMNN, ILF3, SF3A2, FBL, ANXA2, SON, RPL23, PSPC1, LUC7L, RBM14, PGK2, RBM17	164	9853	18082	8.30E-04	3.19E-05	6.07E-04
GOTERM_BP_ALL	GO:0050684~regulation of mRNA processing	10	5.4054	3.83E-07	10.70447549	KHDRBS1, RBFOX2, SON, U2AF2, SREK1, PTBP1, NSRP1, DDX5, RBMX, RBM25	164	103	18082	9.21E-04	3.41E-05	6.73E-04
GOTERM_BP_ALL	GO:1903311~regulation of mRNA metabolic process	11	5.9459	4.31E-07	8.917772597	KHDRBS1, RBFOX2, SON, U2AF2, SREK1, PTBP1, NSRP1, DDX5, RBMX, HNRNPR, RBM25	164	136	18082	0.0010348	3.70E-05	7.57E-04

GOTERM_BP_ALL	GO:0000381~regulation of alternative mRNA splicing, via spliceosome	7	3.7838	1.25E-06	19.78955597	RBFOX2, SREK1, PTBP1, NSRP1, DDX5, RBMX, RBM25	164	39	18082	0.00299467	1.03E-04	0.0021918
GOTERM_BP_ALL	GO:0000245~spliceosomal complex assembly	7	3.7838	2.26E-06	17.94866704	CRNKL1, LUC7L2, SLU7, SF3A2, LUC7L, RBMX, LUC7L3	164	43	18082	0.00542499	1.81E-04	0.0039754
GOTERM_BP_ALL	GO:0043933~macromolecular complex subunit organization	42	22.703	5.15E-06	2.077503857	CRNKL1, TICRR, NAP1L1, MUM1, RPS27L, RPL38, PAXBP1, HMGN5, SERPINH1, HIST2H2AB, TOP1, HIST1H2BM, SQSTM1, POLE3, NUDT21, LUC7L2, H2AFX, HIST1H4J, LUC7L3, HIST1H2BA, RPSA, NLRP5, APCS, EPB41, HIST1H1C, GMNN, DECR1, SF3A2, RBMX, FBL, SMN1, ANXA2, MRTO4, NCK2, HIST1H1T, CLGN, CENPV, CPSF6, SLU7, CIRBP, RBM14, LUC7L	164	2229	18082	0.01229192	3.99E-04	0.0090385
GOTERM_BP_ALL	GO:0010608~posttranscriptional regulation of gene expression	16	8.6486	9.08E-06	4.074128316	KHDRBS1, NLRP5, RBM3, PTBP1, ILF3, RPL38, HNRNPR, HNRNPU, HNRNPA0, NCK2, EIF2AK1, PIWIL1, CIRBP, HBB-BS, RBM14, MATR3	164	433	18082	0.02157613	6.81E-04	0.0159396
GOTERM_BP_ALL	GO:0034622~cellular macromolecular complex assembly	23	12.432	1.58E-05	2.858951797	HIST1H2BA, RPSA, NLRP5, CRNKL1, HIST1H1C, TICRR, NAP1L1, RPS27L, RPL38, SF3A2, RBMX, SMN1, MRTO4, NCK2, HIST1H2BM, HIST1H1T, CENPV, LUC7L2, SLU7, CIRBP, LUC7L, HIST1H4J, LUC7L3	164	887	18082	0.03733085	0.0011522	0.0278006
GOTERM_BP_ALL	GO:0065003~macromolecular complex assembly	32	17.297	1.80E-05	2.285100468	CRNKL1, TICRR, NAP1L1, RPS27L, RPL38, HIST1H2BM, SQSTM1, NUDT21, LUC7L2, HIST1H4J, LUC7L3, HIST1H2BA, RPSA, NLRP5, APCS, EPB41, HIST1H1C, GMNN, DECR1, SF3A2, RBMX, SMN1, ANXA2, MRTO4, NCK2, HIST1H1T, CLGN, CENPV, CPSF6, SLU7, CIRBP, LUC7L	164	1544	18082	0.04240602	0.0012736	0.0316625
GOTERM_BP_ALL	GO:0006376~mRNA splice site selection	5	2.7027	5.87E-05	22.97002033	LUC7L2, SLU7, LUC7L, RBMX, LUC7L3	164	24	18082	0.13166304	0.0040255	0.1031208
GOTERM_BP_ALL	GO:0006518~peptide metabolic process	20	10.811	3.33E-04	2.528809577	KHDRBS1, RPSA, TICRR, RBM3, PTBP1, ILF3, RPS27L, RPL38, HMGN5, HNRNPR, GSTM1, NCK2, GSTM2, EIF2AK1, H13, RPL23, CIRBP, HBB-BS, GSTO1, RBM14	164	872	18082	0.55083	0.0219867	0.5832084

GOTERM_BP_ALL	GO:0010468~regulation of gene expression	59	31.892	3.62E-04	1.524158799	PTGES3, NKAP, U2AF2, RBM3, YBX3, HMGN5, NSRP1, CBX5, PNN, HIST2H2AB, TOP1, DDX17, FUBP3, HSF5, PIWIL1, MUP18, H2AFX, CCAR2, KHDRBS1, RBFOX2, NLRP5, HIST1H1C, PTBP1, SF1, CDC5L, BASP1, HNRNPR, SMN1, HNRNPU, NCK2, EIF2AK1, MED15, LARP7, HBB-BS, RBM39, MATR3, FKBP6, VIM, PAXBP1, RPL38, DDX46, SQSTM1, DDX41, HIST1H4J, RBM25, SREK1, GMNN, ILF3, DDX5, RBMX, HNRNPA0, SON, SRSF5, TRPS1, PSPC1, CIRBP, RBM14, PUF60, PHF6	164	4268	18082	0.58089388	0.0232295	0.6335297
GOTERM_BP_ALL	GO:0006417~regulation of translation	12	6.4865	5.32E-04	3.585564148	KHDRBS1, NCK2, EIF2AK1, RBM3, PTBP1, PIWIL1, CIRBP, HBB-BS, ILF3, RPL38, RBM14, HNRNPR	164	369	18082	0.72157144	0.0330874	0.9300715
GOTERM_BP_ALL	GO:0044085~cellular component biogenesis	41	22.162	5.88E-04	1.703278071	CRNKL1, TICRR, PDLIM5, NAP1L1, RPS27L, RPL38, HIST1H2BM, FRG1, SQSTM1, TBC1D14, NUDT21, LUC7L2, DDX21, HIST1H4J, LUC7L3, HIST1H2BA, RPSA, NLRP5, GTPBP4, APCS, EPB41, HIST1H1C, GMNN, CDC20, DECR1, SF3A2, RBMX, FBL, SMN1, ANXA2, MRTO4, NCK2, HIST1H1T, CHGA, CLGN, CENPV, CPSF6, SLU7, CIRBP, LUC7L, RBM14	164	2654	18082	0.7569202	0.0356161	1.0283248
GOTERM_BP_ALL	GO:0010501~RNA secondary structure unwinding	5	2.7027	7.11E-04	12.25067751	DDX17, DDX46, DDX21, DDX5, DDX41	164	45	18082	0.81899671	0.0418309	1.2413797
GOTERM_BP_ALL	GO:0043487~regulation of RNA stability	6	3.2432	7.37E-04	8.373880827	NLRP5, PTBP1, CIRBP, HNRNPR, HNRNPU, HNRNPA0	164	79	18082	0.82996866	0.0422935	1.2865021
GOTERM_BP_ALL	GO:0034248~regulation of cellular amide metabolic process	12	6.4865	9.50E-04	3.341093865	KHDRBS1, NCK2, EIF2AK1, RBM3, PTBP1, PIWIL1, CIRBP, HBB-BS, ILF3, RPL38, RBM14, HNRNPR	164	396	18082	0.89803142	0.0529082	1.6546885
GOTERM_BP_ALL	GO:0043603~cellular amide metabolic process	21	11.351	0.0010362	2.239243761	KHDRBS1, RPSA, TICRR, RBM3, PTBP1, ILF3, RPS27L, RPL38, HMGN5, HNRNPR, GSTM1, NCK2, GSTM2, EIF2AK1, H13, RPL23, PIWIL1, CIRBP, HBB-BS, GSTO1, RBM14	164	1034	18082	0.9171951	0.0562901	1.8041979
GOTERM_BP_ALL	GO:0031497~chromatin assembly	7	3.7838	0.0014665	5.674946198	HIST1H2BA, HIST1H1T, HIST1H2BM, HIST1H1C, NAP1L1, CENPV, HIST1H4J	164	136	18082	0.97059731	0.0770236	2.5444346

GOTERM_BP_ALL	GO:0060255~regulation of macromolecule metabolic process	71	38.378	0.0016496	1.364032571	PTGES3, NKAP, TEX101, U2AF2, RBM3, YBX3, HMGN5, NSRP1, CBX5, PNN, HIST2H2AB, TOP1, DDX17, FUBP3, HSF5, PIWIL1, MUP18, H2AFX, CCAR2, KHDRBS1, KNG1, RBFOX2, GTPBP4, NLRP5, APCS, HIST1H1C, PTBP1, SF1, BASP1, CDC5L, HNRNPR, HNRNPU, SMN1, NCK2, EIF2AK1, MED15, LARP7, HSPB1, HBB-BS, RBM39, MATR3, FKBP6, TICRR, VIM, HSPA1B, PAXBP1, RPL38, ROPN1L, DDX46, SERPINA1B, SQSTM1, CACYBP, DDX41, HIST1H4J, RBM25, SREK1, GMNN, CDC20, ILF3, DDX5, RBMX, HNRNPA0, ANXA2, SON, SRSF5, TRPS1, PSPC1, CIRBP, RBM14, PUF60, PHF6	164	5739	18082	0.98107478	0.0843869	2.8577304
GOTERM_BP_ALL	GO:0051172~negative regulation of nitrogen compound metabolic process	27	14.595	0.0024072	1.854775473	NKAP, RBM3, U2AF2, YBX3, CBX5, HIST2H2AB, SQSTM1, MUP18, H2AFX, HIST1H4J, CCAR2, KHDRBS1, RBFOX2, GTPBP4, HIST1H1C, GMNN, PTBP1, ILF3, BASP1, DDX5, HNRNPR, HNRNPU, NCK2, EIF2AK1, LARP7, TRPS1, PSPC1	164	1605	18082	0.99694622	0.1182965	4.1441433
GOTERM_BP_ALL	GO:0006413~translational initiation	6	3.2432	0.0025122	6.360928705	KHDRBS1, NCK2, EIF2AK1, TICRR, PTBP1, HBB-BS	164	104	18082	0.99762873	0.1206768	4.3211829
GOTERM_BP_ALL	GO:2000113~negative regulation of cellular macromolecule biosynthetic process	25	13.514	0.0025486	1.9101888	NKAP, KHDRBS1, RBFOX2, GTPBP4, HIST1H1C, GMNN, RBM3, YBX3, ILF3, BASP1, DDX5, HNRNPR, HNRNPU, CBX5, HIST2H2AB, NCK2, EIF2AK1, SQSTM1, TRPS1, LARP7, PSPC1, MUP18, H2AFX, HIST1H4J, CCAR2	164	1443	18082	0.99782788	0.1199275	4.3825001
GOTERM_BP_ALL	GO:0051171~regulation of nitrogen compound metabolic process	55	29.73	0.0027436	1.436646616	NKAP, U2AF2, RBM3, YBX3, HMGN5, NSRP1, CBX5, PNN, HIST2H2AB, DDX17, FUBP3, HSF5, PIWIL1, MUP18, H2AFX, CCAR2, KHDRBS1, RBFOX2, GTPBP4, HIST1H1C, PTBP1, SF1, CDC5L, BASP1, HNRNPR, SMN1, HNRNPU, NCK2, CHGA, EIF2AK1, MED15, LARP7, HBB-BS, RBM39, TICRR, PAXBP1, RPL38, SQSTM1, CACYBP, DDX41, HIST1H4J, RBM25, GMNN, SREK1, ILF3, DDX5, RBMX, SON, SRSF5, TRPS1, PSPC1, CIRBP, RBM14, PUF60, PHF6	164	4221	18082	0.99864225	0.126051	4.7102852
GOTERM_BP_ALL	GO:0006325~chromatin organization	16	8.6486	0.0028441	2.38714149	HIST1H2BA, HIST1H1C, NAP1L1, MUM1, PAXBP1, HMGN5, FBL, TOP1, HIST2H2AB, HIST1H2BM, HIST1H1T, POLE3, CENPV, H2AFX, RBM14, HIST1H4J	164	739	18082	0.99893427	0.1279268	4.8787794
GOTERM_BP_ALL	GO:0010629~negative regulation of gene expression	27	14.595	0.0029234	1.828571643	NKAP, FKBP6, RBM3, U2AF2, YBX3, CBX5, HIST2H2AB, SQSTM1, MUP18, PIWIL1, H2AFX, HIST1H4J, CCAR2, KHDRBS1, RBFOX2, HIST1H1C, GMNN, PTBP1, ILF3, BASP1, DDX5, HNRNPR, NCK2, EIF2AK1, LARP7, TRPS1, PSPC1	164	1628	18082	0.99911959	0.1288522	5.0114855

GOTERM_BP_ALL	GO:0018208~peptidyl-proline modification	4	2.1622	0.0029441	13.7820122	PPIG, FKBP6, PPIL4, PPIL3	164	32	18082	0.99916246	0.1273762	5.0461276
GOTERM_BP_ALL	GO:0071103~DNA conformation change	8	4.3243	0.002966	4.200232288	HIST1H2BA, TOP1, HIST1H1T, HIST1H2BM, HIST1H1C, NAP1L1, CENPV, HIST1H4J	164	210	18082	0.99920564	0.1260033	5.0828526
GOTERM_BP_ALL	GO:0006333~chromatin assembly or disassembly	7	3.7838	0.0031182	4.884763816	HIST1H2BA, HIST1H1T, HIST1H2BM, HIST1H1C, NAP1L1, CENPV, HIST1H4J	164	158	18082	0.99944952	0.1297512	5.3369119
GOTERM_BP_ALL	GO:0019222~regulation of metabolic process	73	39.459	0.0034228	1.319890968	PTGES3, NKAP, TEX101, U2AF2, RBM3, YBX3, HMGN5, NSRP1, CBX5, PNN, HIST2H2AB, TOP1, DDX17, FUBP3, HSF5, PIWIL1, MUP18, H2AFX, CCAR2, KHDRBS1, KNG1, RBFOX2, GTPBP4, NLRP5, APCS, HIST1H1C, PTBP1, SF1, BASP1, CDC5L, HNRNPR, HNRNPU, SMN1, NCK2, CHGA, EIF2AK1, MED15, LARP7, HSPB1, HBB-BS, RBM39, MATR3, FKBP6, TICRR, VIM, HSPA1B, PAXBP1, RPL38, ROPN1L, DDX46, SERPINA1B, SQSTM1, CACYBP, DDX41, HIST1H4J, RBM25, SREK1, GMNN, MYO1D, CDC20, ILF3, DDX5, RBMX, HNRNPA0, ANXA2, SON, SRSF5, TRPS1, PSPC1, CIRBP, RBM14, PUF60, PHF6	164	6098	18082	0.99973585	0.1391196	5.8435342
GOTERM_BP_ALL	GO:0010605~negative regulation of macromolecule metabolic process	35	18.919	0.0038121	1.626881709	NKAP, FKBP6, U2AF2, RBM3, YBX3, HSPA1B, CBX5, HIST2H2AB, SERPINA1B, SQSTM1, MUP18, PIWIL1, H2AFX, HIST1H4J, CCAR2, KNG1, KHDRBS1, RBFOX2, GTPBP4, APCS, HIST1H1C, GMNN, PTBP1, ILF3, BASP1, DDX5, HNRNPR, HNRNPU, ANXA2, NCK2, EIF2AK1, TRPS1, LARP7, PSPC1, HSPB1	164	2372	18082	0.9998967	0.1511654	6.4873982
GOTERM_BP_ALL	GO:0043488~regulation of mRNA stability	5	2.7027	0.0038692	7.764513913	PTBP1, CIRBP, HNRNPR, HNRNPU, HNRNPA0	164	71	18082	0.99991	0.1507775	6.581562
GOTERM_BP_ALL	GO:0048255~mRNA stabilization	4	2.1622	0.004128	12.25067751	PTBP1, CIRBP, HNRNPU, HNRNPA0	164	36	18082	0.9999518	0.1575004	7.0068954
GOTERM_BP_ALL	GO:0006334~nucleosome assembly	6	3.2432	0.0041696	5.654158849	HIST1H2BA, HIST1H1T, HIST1H2BM, HIST1H1C, NAP1L1, HIST1H4J	164	117	18082	0.99995641	0.1564867	7.075125

GOTERM_BP_ALL	GO:0071840~cellular component organization or biogenesis	72	38.919	0.0041787	1.314964225	NKAP, CRNKL1, U2AF2, PDLIM5, NAP1L1, RPS27L, HMGN5, HIST2H2AB, TOP1, HIST1H2BM, TBC1D14, MUP18, LUC7L2, H2AFX, DDX21, LUC7L3, CCAR2, RBFOX2, GTPBP4, NLRP5, APCS, HIST1H1C, STK24, PTBP1, SF1, MFGE8, DECR1, MARK3, HNRNPU, SMN1, MRTO4, NCK2, CHGA, HIST1H1T, SIPA1L1, CFAP20, HSPB1, SLU7, CPSF6, FKBP6, SNX5, TICRR, VIM, WDR60, MUM1, PAXBP1, RPL38, SERPINH1, POLE3, SQSTM1, FRG1, NUDT21, IGKC, HIST1H4J, ERCC6L, PRPF40A, HIST1H2BA, RPSA, EPB41, GMNN, CDC20, SF3A2, RBMX, FBL, ANXA2, SON, CLGN, CENPV, CIRBP, LUC7L, RBM14, PGK2	164	6037	18082	0.99995735	0.1543981	7.0899307
GOTERM_BP_ALL	GO:0045934~negative regulation of nucleobase-containing compound metabolic process	24	12.973	0.004244	1.867428611	NKAP, KHDRBS1, RBFOX2, GTPBP4, HIST1H1C, GMNN, U2AF2, PTBP1, YBX3, ILF3, BASP1, DDX5, HNRNPU, CBX5, NCK2, HIST2H2AB, SQSTM1, TRPS1, LARP7, MUP18, PSPC1, H2AFX, HIST1H4J, CCAR2	164	1417	18082	0.99996357	0.1542581	7.1968846
GOTERM_BP_ALL	GO:0010558~negative regulation of macromolecule biosynthetic process	25	13.514	0.0043904	1.830280504	NKAP, KHDRBS1, RBFOX2, GTPBP4, HIST1H1C, GMNN, RBM3, YBX3, ILF3, BASP1, DDX5, HNRNPR, HNRNPU, CBX5, HIST2H2AB, NCK2, EIF2AK1, SQSTM1, TRPS1, LARP7, PSPC1, MUP18, H2AFX, HIST1H4J, CCAR2	164	1506	18082	0.99997441	0.1567878	7.4361712
GOTERM_BP_ALL	GO:0009059~macromolecule biosynthetic process	61	32.973	0.0046273	1.365608518	PTGES3, NKAP, RBM3, YBX3, RPS27L, HMGN5, CBX5, PNN, HIST2H2AB, TOP1, DDX17, FUBP3, HSF5, PIWIL1, MUP18, H2AFX, DDX21, TWISTNB, CCAR2, KHDRBS1, RBFOX2, GTPBP4, HIST1H1C, PTBP1, SF1, POLB, CDC5L, BASP1, HNRNPR, SMN1, HNRNPU, NCK2, EIF2AK1, MED15, LARP7, HSPB1, HBB-BS, RBM39, TICRR, PAXBP1, RPL38, SERPINH1, SERPINA1B, SQSTM1, CACYBP, DDX41, HIST1H4J, RPSA, GMNN, ILF3, DDX5, RBMX, SON, SRSF5, RPL23, TRPS1, PSPC1, CIRBP, RBM14, PUF60, PHF6	164	4925	18082	0.99998555	0.1621424	7.8222226
GOTERM_BP_ALL	GO:0043489~RNA stabilization	4	2.1622	0.0048137	11.60590501	PTBP1, CIRBP, HNRNPU, HNRNPA0	164	38	18082	0.99999079	0.1657103	8.1248875
GOTERM_BP_ALL	GO:0006323~DNA packaging	7	3.7838	0.0049866	4.435590132	HIST1H2BA, HIST1H1T, HIST1H2BM, HIST1H1C, NAP1L1, CENPV, HIST1H4J	164	174	18082	0.99999393	0.1687389	8.4048326

GOTERM_BP_ALL	GO:0022607~cellular component assembly	35	18.919	0.005157	1.595931933	CRNKL1, TICRR, NAP1L1, RPS27L, RPL38, HIST1H2BM, SQSTM1, TBC1D14, NUDT21, LUC7L2, HIST1H4J, LUC7L3, HIST1H2BA, RPSA, NLRP5, APCS, EPB41, HIST1H1C, GMNN, CDC20, DECR1, SF3A2, RBMX, SMN1, ANXA2, MRTO4, NCK2, HIST1H1T, CLGN, CENPV, CPSF6, SLU7, CIRBP, RBM14, LUC7L	164	2418	18082	0.99999598	0.1715911	8.6799771
GOTERM_BP_ALL	GO:0006412~translation	15	8.1081	0.0052854	2.316304571	KHDRBS1, RPSA, TICRR, RBM3, PTBP1, ILF3, RPS27L, RPL38, HNRNPR, NCK2, EIF2AK1, RPL23, CIRBP, HBB-BS, RBM14	164	714	18082	0.99999705	0.1730957	8.8866681
GOTERM_BP_ALL	GO:0065004~protein-DNA complex assembly	7	3.7838	0.0054032	4.360410638	HIST1H2BA, HIST1H1T, HIST1H2BM, HIST1H1C, NAP1L1, CENPV, HIST1H4J	164	177	18082	0.99999778	0.1742463	9.0760767
GOTERM_BP_ALL	GO:0051253~negative regulation of RNA metabolic process	22	11.892	0.0057177	1.890595593	NKAP, KHDRBS1, RBFOX2, HIST1H1C, GMNN, U2AF2, PTBP1, YBX3, ILF3, BASP1, DDX5, CBX5, NCK2, HIST2H2AB, SQSTM1, TRPS1, LARP7, MUP18, PSPC1, H2AFX, HIST1H4J, CCAR2	164	1283	18082	0.99999896	0.1810213	9.5796372
GOTERM_BP_ALL	GO:0043043~peptide biosynthetic process	15	8.1081	0.0067683	2.25012444	KHDRBS1, RPSA, TICRR, RBM3, PTBP1, ILF3, RPS27L, RPL38, HNRNPR, NCK2, EIF2AK1, RPL23, CIRBP, HBB-BS, RBM14	164	735	18082	0.99999992	0.2079537	11.242857
GOTERM_BP_ALL	GO:0009566~fertilization	7	3.7838	0.0068526	4.149423026	HIST1H1T, NLRP5, CLGN, YBX3, MFGE8, HSPA1B, INSL6	164	186	18082	0.99999993	0.2076293	11.37512
GOTERM_BP_ALL	GO:0031327~negative regulation of cellular biosynthetic process	25	13.514	0.0069719	1.762405652	NKAP, KHDRBS1, RBFOX2, GTPBP4, HIST1H1C, GMNN, RBM3, YBX3, ILF3, BASP1, DDX5, HNRNPR, HNRNPU, CBX5, HIST2H2AB, NCK2, EIF2AK1, SQSTM1, TRPS1, LARP7, PSPC1, MUP18, H2AFX, HIST1H4J, CCAR2	164	1564	18082	0.99999995	0.208245	11.561912
GOTERM_BP_ALL	GO:0034645~cellular macromolecule biosynthetic process	59	31.892	0.0070072	1.352413671	PTGES3, NKAP, RBM3, YBX3, RPS27L, HMGN5, CBX5, PNN, HIST2H2AB, TOP1, DDX17, FUBP3, HSF5, PIWIL1, MUP18, H2AFX, DDX21, TWISTNB, CCAR2, KHDRBS1, RBFOX2, GTPBP4, HIST1H1C, PTBP1, SF1, POLB, CDC5L, BASP1, HNRNPR, SMN1, HNRNPU, NCK2, EIF2AK1, MED15, LARP7, HBB-BS, RBM39, TICRR, PAXBP1, RPL38, SERPINA1B, SQSTM1, CACYBP, DDX41, HIST1H4J, RPSA, GMNN, ILF3, DDX5, RBMX, SON, SRSF5, RPL23, TRPS1, PSPC1, CIRBP, RBM14, PUF60, PHF6	164	4810	18082	0.99999995	0.2066373	11.617105

GOTERM_BP_ALL	GO:0006351~transcription, DNA-templated	42	22.703	0.0070362	1.479947618	NKAP, YBX3, PAXBP1, HMGN5, PNN, CBX5, HIST2H2AB, TOP1, FUBP3, DDX17, SQSTM1, HSF5, MUP18, H2AFX, DDX21, DDX41, TWISTNB, HIST1H4J, CCAR2, KHDRBS1, RBFOX2, HIST1H1C, GMNN, PTBP1, SF1, ILF3, CDC5L, BASP1, DDX5, RBMX, SMN1, NCK2, SRSF5, SON, MED15, TRPS1, LARP7, PSPC1, RBM39, RBM14, PUF60, PHF6	164	3129	18082	0.99999996	0.2049049	11.662347
GOTERM_BP_ALL	GO:0031324~negative regulation of cellular metabolic process	34	18.378	0.0072675	1.575087108	NKAP, U2AF2, RBM3, YBX3, HSPA1B, CBX5, HIST2H2AB, SERPINA1B, SQSTM1, MUP18, H2AFX, HIST1H4J, CCAR2, KNG1, KHDRBS1, RBFOX2, GTPBP4, APCS, HIST1H1C, GMNN, MYO1D, PTBP1, ILF3, BASP1, DDX5, HNRNPR, HNRNPU, ANXA2, NCK2, EIF2AK1, TRPS1, LARP7, PSPC1, HSPB1	164	2380	18082	0.99999998	0.2084017	12.023052
GOTERM_BP_ALL	GO:0009892~negative regulation of metabolic process	36	19.459	0.0078498	1.539053708	NKAP, FKBP6, U2AF2, RBM3, YBX3, HSPA1B, CBX5, HIST2H2AB, SERPINA1B, SQSTM1, MUP18, PIWIL1, H2AFX, HIST1H4J, CCAR2, KNG1, KHDRBS1, RBFOX2, GTPBP4, APCS, HIST1H1C, GMNN, MYO1D, PTBP1, ILF3, BASP1, DDX5, HNRNPR, HNRNPU, ANXA2, NCK2, EIF2AK1, TRPS1, LARP7, PSPC1, HSPB1	164	2579	18082	0.99999999	0.2205581	12.924881
GOTERM_BP_ALL	GO:0016043~cellular component organization	69	37.297	0.0080248	1.295584253	NKAP, CRNKL1, U2AF2, PDLIM5, NAP1L1, RPS27L, HMGN5, HIST2H2AB, TOP1, HIST1H2BM, TBC1D14, MUP18, LUC7L2, H2AFX, LUC7L3, CCAR2, RBFOX2, NLRP5, APCS, HIST1H1C, STK24, PTBP1, SF1, MFGE8, DECR1, MARK3, HNRNPU, SMN1, MRTO4, NCK2, CHGA, HIST1H1T, SIPA1L1, CFAP20, SLU7, HSPB1, CPSF6, FKBP6, SNX5, TICRR, VIM, WDR60, MUM1, PAXBP1, RPL38, SERPINH1, SQSTM1, POLE3, NUDT21, IGKC, HIST1H4J, PRPF40A, ERCC6L, HIST1H2BA, RPSA, EPB41, GMNN, CDC20, SF3A2, RBMX, FBL, ANXA2, SON, CLGN, CENPV, CIRBP, LUC7L, RBM14, PGK2	164	5872	18082	1	0.2223259	13.194292
GOTERM_BP_ALL	GO:0009890~negative regulation of biosynthetic process	25	13.514	0.0088025	1.728151999	NKAP, KHDRBS1, RBFOX2, GTPBP4, HIST1H1C, GMNN, RBM3, YBX3, ILF3, BASP1, DDX5, HNRNPR, HNRNPU, CBX5, HIST2H2AB, NCK2, EIF2AK1, SQSTM1, TRPS1, LARP7, PSPC1, MUP18, H2AFX, HIST1H4J, CCAR2	164	1595	18082	1	0.23844	14.38171
GOTERM_BP_ALL	GO:0000380~alternative mRNA splicing, via spliceosome	3	1.6216	0.0089203	20.67301829	HNRNPM, SLU7, RBM17	164	16	18082	1	0.2385667	14.560191

GOTERM_BP_ALL	GO:0010556~regulation of macromolecule biosynthetic process	50	27.027	0.0093238	1.390016359	NKAP, RBM3, YBX3, HMGN5, PNN, CBX5, HIST2H2AB, DDX17, FUBP3, HSF5, PIWIL1, MUP18, H2AFX, CCAR2, KHDRBS1, RBFOX2, GTPBP4, HIST1H1C, PTBP1, SF1, CDC5L, BASP1, HNRNPR, HNRNPU, NCK2, EIF2AK1, MED15, LARP7, HSPB1, HBB-BS, RBM39, TICRR, PAXBP1, RPL38, SQSTM1, CACYBP, DDX41, HIST1H4J, GMNN, ILF3, DDX5, RBMX, SON, SRSF5, TRPS1, PSPC1, CIRBP, RBM14, PUF60, PHF6	164	3966	18082	1	0.2452576	15.169081
GOTERM_BP_ALL	GO:0034728~nucleosome organization	6	3.2432	0.0096008	4.626129968	HIST1H2BA, HIST1H1T, HIST1H2BM, HIST1H1C, NAP1L1, HIST1H4J	164	143	18082	1	0.2488871	15.584577
GOTERM_BP_ALL	GO:2000112~regulation of cellular macromolecule biosynthetic process	49	26.486	0.0097038	1.394206137	NKAP, TICRR, RBM3, YBX3, PAXBP1, RPL38, HMGN5, PNN, CBX5, HIST2H2AB, FUBP3, DDX17, SQSTM1, CACYBP, HSF5, MUP18, PIWIL1, H2AFX, DDX41, HIST1H4J, CCAR2, KHDRBS1, RBFOX2, GTPBP4, HIST1H1C, GMNN, PTBP1, SF1, ILF3, CDC5L, BASP1, DDX5, HNRNPR, RBMX, HNRNPU, NCK2, SON, SRSF5, EIF2AK1, MED15, TRPS1, LARP7, PSPC1, CIRBP, HBB-BS, RBM39, RBM14, PUF60, PHF6	164	3875	18082	1	0.2485544	15.738599
GOTERM_BP_ALL	GO:0071824~protein-DNA complex subunit organization	7	3.7838	0.0103762	3.801934399	HIST1H2BA, HIST1H1T, HIST1H2BM, HIST1H1C, NAP1L1, CENPV, HIST1H4J	164	203	18082	1	0.2606458	16.737714
GOTERM_BP_ALL	GO:0008037~cell recognition	6	3.2432	0.0107328	4.50024888	NCK2, HIST1H1T, CLGN, MFGE8, HSPA1B, IGKC	164	147	18082	1	0.2655956	17.263147
GOTERM_BP_ALL	GO:0048523~negative regulation of cellular process	54	29.189	0.0117401	1.347935085	PTGES3, TEX101, SRI, NKAP, TICRR, U2AF2, RBM3, VIM, YBX3, RPS27L, HSPA1B, INSL6, HMGN5, CBX5, HIST2H2AB, SERPINA1B, SQSTM1, TBC1D14, CACYBP, MUP18, H2AFX, GSTO1, CEACAM2, HIST1H4J, CCAR2, KNG1, KHDRBS1, RBFOX2, GTPBP4, APCS, HIST1H1C, STK24, GMNN, G3BP1, MYO1D, PTBP1, SF1, ILF3, BASP1, CDC5L, DDX5, HNRNPR, HNRNPU, ANXA2, NCK2, SON, CHGA, EIF2AK1, TRPS1, LARP7, PSPC1, CIRBP, HSPB1, RBM14	164	4417	18082	1	0.2838492	18.73023

GOTERM_BP_ALL	GO:0006996~organelle organization	46	24.865	0.0118681	1.401044334	NKAP, CRNKL1, FKBP6, SNX5, U2AF2, VIM, NAP1L1, MUM1, RPS27L, PAXBP1, RPL38, HMGN5, HIST2H2AB, TOP1, HIST1H2BM, SQSTM1, POLE3, TBC1D14, MUP18, H2AFX, HIST1H4J, CCAR2, PRPF40A, ERCC6L, HIST1H2BA, RPSA, EPB41, HIST1H1C, PTBP1, SF1, CDC20, MARK3, FBL, SMN1, HNRNPU, ANXA2, MRTO4, NCK2, HIST1H1T, CHGA, SON, SIPA1L1, CENPV, CIRBP, RBM14, PGK2	164	3620	18082	1	0.2836605	18.914854
GOTERM_BP_ALL	GO:0045727~positive regulation of translation	5	2.7027	0.0119371	5.6253111	KHDRBS1, NCK2, RBM3, PTBP1, CIRBP	164	98	18082	1	0.282294	19.014228
GOTERM_BP_ALL	GO:0019219~regulation of nucleobase-containing compound metabolic process	49	26.486	0.0122345	1.376796325	NKAP, TICRR, U2AF2, YBX3, PAXBP1, HMGN5, NSRP1, PNN, CBX5, HIST2H2AB, FUBP3, DDX17, SQSTM1, CACYBP, HSF5, MUP18, H2AFX, DDX41, HIST1H4J, CCAR2, RBM25, KHDRBS1, RBFOX2, GTPBP4, HIST1H1C, GMNN, SREK1, PTBP1, SF1, ILF3, CDC5L, BASP1, DDX5, HNRNPR, RBMX, SMN1, HNRNPU, NCK2, SRSF5, CHGA, SON, MED15, TRPS1, LARP7, PSPC1, RBM39, RBM14, PUF60, PHF6	164	3924	18082	1	0.2854803	19.441159
GOTERM_BP_ALL	GO:0045892~negative regulation of transcription, DNA-templated	20	10.811	0.0125348	1.82694445	KHDRBS1, NKAP, RBFOX2, HIST1H1C, GMNN, YBX3, ILF3, BASP1, DDX5, CBX5, NCK2, HIST2H2AB, SQSTM1, TRPS1, LARP7, PSPC1, MUP18, H2AFX, HIST1H4J, CCAR2	164	1207	18082	1	0.288642	19.870286
GOTERM_BP_ALL	GO:0097193~intrinsic apoptotic signaling pathway	8	4.3243	0.0127096	3.195828915	NCK2, STK24, YBX3, HSPB1, POLB, RPS27L, DDX5, CCAR2	164	276	18082	1	0.2893124	20.119039
GOTERM_BP_ALL	GO:0042254~ribosome biogenesis	8	4.3243	0.0131584	3.17283734	RPSA, GTPBP4, FRG1, DDX21, RPS27L, RPL38, FBL, MRTO4	164	278	18082	1	0.2951523	20.75422
GOTERM_BP_ALL	GO:0048519~negative regulation of biological process	57	30.811	0.0136678	1.321126248	NKAP, TEX101, PTGES3, RBM3, U2AF2, YBX3, RPS27L, HMGN5, INSL6, CBX5, HIST2H2AB, TBC1D14, PIWIL1, MUP18, H2AFX, CEACAM2, CCAR2, KHDRBS1, KNG1, RBFOX2, GTPBP4, APCS, HIST1H1C, STK24, G3BP1, PTBP1, SF1, CDC5L, BASP1, HNRNPR, HNRNPU, NCK2, CHGA, EIF2AK1, LARP7, HSPB1, SRI, FKBP6, TICRR, VIM, HSPA1B, SERPINA1B, SQSTM1, CACYBP, GSTO1, HIST1H4J, GMNN, MYO1D, ILF3, DDX5, ANXA2, SON, TRPS1, PSPC1, CIRBP, RBM14, LUC7L	164	4757	18082	1	0.301947	21.469658

GOTERM_BP_ALL	GO:0000413~protein peptidyl-prolyl isomerization	3	1.6216	0.013795	16.53841463	PPIG, PPIL4, PPIL3	164	20	18082	1	0.301575	21.647323
GOTERM_BP_ALL	GO:0006749~glutathione metabolic process	4	2.1622	0.0140612	7.87543554	GSTM1, GSTM2, GSTO1, HMGN5	164	56	18082	1	0.3037233	22.017794
GOTERM_BP_ALL	GO:1903507~negative regulation of nucleic acid-templated transcription	20	10.811	0.0141305	1.804518782	KHDRBS1, NKAP, RBFOX2, HIST1H1C, GMNN, YBX3, ILF3, BASP1, DDX5, CBX5, NCK2, HIST2H2AB, SQSTM1, TRPS1, LARP7, PSPC1, MUP18, H2AFX, HIST1H4J, CCAR2	164	1222	18082	1	0.3023078	22.114093
GOTERM_BP_ALL	GO:0043604~amide biosynthetic process	15	8.1081	0.0143903	2.051912486	KHDRBS1, RPSA, TICRR, RBM3, PTBP1, ILF3, RPS27L, RPL38, HNRNPR, NCK2, EIF2AK1, RPL23, CIRBP, HBB-BS, RBM14	164	806	18082	1	0.304292	22.473786
GOTERM_BP_ALL	GO:0009058~biosynthetic process	68	36.757	0.0151782	1.26560004	PTGES3, NKAP, LDHA, RBM3, YBX3, RPS27L, HMGN5, CBX5, PNN, GOT2, HIST2H2AB, TOP1, DDX17, FUBP3, HSF5, PIWIL1, MUP18, H2AFX, DDX21, PDHA2, TWISTNB, CCAR2, KHDRBS1, RBFOX2, GTPBP4, HIST1H1C, PTBP1, SF1, POLB, BASP1, CDC5L, HNRNPR, HNRNPU, SMN1, NCK2, EIF2AK1, MED15, LARP7, HSPB1, HBB-BS, RBM39, TICRR, PAXBP1, RPL38, SERPINH1, SERPINA1B, SQSTM1, CACYBP, GMPPA, GSTO1, DDX41, HIST1H4J, DCTD, RPSA, GMNN, ILF3, DDX5, AK6, RBMX, SON, SRSF5, RPL23, TRPS1, PSPC1, CIRBP, RBM14, PUF60, PHF6	164	5924	18082	1	0.3153832	23.554963
GOTERM_BP_ALL	GO:0009988~cell-cell recognition	4	2.1622	0.0154495	7.603868797	NCK2, HIST1H1T, CLGN, HSPA1B	164	58	18082	1	0.3173577	23.923882
GOTERM_BP_ALL	GO:1901576~organic substance biosynthetic process	67	36.216	0.0158458	1.267094089	PTGES3, NKAP, LDHA, RBM3, YBX3, RPS27L, HMGN5, CBX5, PNN, GOT2, HIST2H2AB, TOP1, DDX17, FUBP3, HSF5, PIWIL1, MUP18, H2AFX, DDX21, PDHA2, TWISTNB, CCAR2, KHDRBS1, RBFOX2, GTPBP4, HIST1H1C, PTBP1, SF1, POLB, BASP1, CDC5L, HNRNPR, HNRNPU, SMN1, NCK2, EIF2AK1, MED15, LARP7, HSPB1, HBB-BS, RBM39, TICRR, PAXBP1, RPL38, SERPINH1, SERPINA1B, SQSTM1, CACYBP, GSTO1, DDX41, HIST1H4J, DCTD, RPSA, GMNN, ILF3, DDX5, AK6, RBMX, SON, SRSF5, RPL23, TRPS1, PSPC1, CIRBP, RBM14, PUF60, PHF6	164	5830	18082	1	0.3213852	24.459953
GOTERM_BP_ALL	GO:1902679~negative regulation of RNA biosynthetic process	20	10.811	0.0158833	1.782636986	KHDRBS1, NKAP, RBFOX2, HIST1H1C, GMNN, YBX3, ILF3, BASP1, DDX5, CBX5, NCK2, HIST2H2AB, SQSTM1, TRPS1, LARP7, PSPC1, MUP18, H2AFX, HIST1H4J, CCAR2	164	1237	18082	1	0.3193726	24.510487

GOTERM_BP_ALL	GO:0044249~cellular biosynthetic process	66	35.676	0.0162759	1.269522407	PTGES3, NKAP, LDHA, RBM3, YBX3, RPS27L, HMGN5, CBX5, PNN, GOT2, HIST2H2AB, TOP1, DDX17, FUBP3, HSF5, PIWIL1, MUP18, H2AFX, DDX21, PDHA2, TWISTNB, CCAR2, KHDRBS1, RBFOX2, GTPBP4, HIST1H1C, PTBP1, SF1, POLB, BASP1, CDC5L, HNRNPR, HNRNPU, SMN1, NCK2, EIF2AK1, MED15, LARP7, HSPB1, HBB-BS, RBM39, TICRR, PAXBP1, RPL38, SERPINA1B, SQSTM1, CACYBP, GSTO1, DDX41, HIST1H4J, DCTD, RPSA, GMNN, ILF3, DDX5, AK6, RBMX, SON, SRSF5, RPL23, TRPS1, PSPC1, CIRBP, RBM14, PUF60, PHF6	164	5732	18082	1	0.3232306	25.037633
GOTERM_BP_ALL	GO:0031570~DNA integrity checkpoint	5	2.7027	0.016534	5.104448961	TICRR, H2AFX, RPS27L, CDC5L, CCAR2	164	108	18082	1	0.3248212	25.382222
GOTERM_BP_ALL	GO:0051276~chromosome organization	18	9.7297	0.0171642	1.84614861	HIST1H2BA, HIST1H1C, NAP1L1, MUM1, CDC20, PAXBP1, HMGN5, FBL, HNRNPU, TOP1, HIST2H2AB, HIST1H2BM, HIST1H1T, POLE3, CENPV, H2AFX, RBM14, HIST1H4J	164	1075	18082	1	0.3323018	26.217483
GOTERM_BP_ALL	GO:0034250~positive regulation of cellular amide metabolic process	5	2.7027	0.017569	5.011640798	KHDRBS1, NCK2, RBM3, PTBP1, CIRBP	164	110	18082	1	0.3360533	26.749334
GOTERM_BP_ALL	GO:0044271~cellular nitrogen compound biosynthetic process	56	30.27	0.0178736	1.308400395	NKAP, RBM3, YBX3, RPS27L, HMGN5, CBX5, PNN, HIST2H2AB, TOP1, DDX17, FUBP3, HSF5, MUP18, H2AFX, DDX21, TWISTNB, CCAR2, KHDRBS1, RBFOX2, HIST1H1C, PTBP1, SF1, POLB, CDC5L, BASP1, HNRNPR, SMN1, HNRNPU, NCK2, EIF2AK1, MED15, LARP7, HBB-BS, RBM39, TICRR, PAXBP1, RPL38, SQSTM1, DDX41, HIST1H4J, DCTD, RPSA, GMNN, ILF3, DDX5, RBMX, AK6, SON, SRSF5, RPL23, TRPS1, PSPC1, CIRBP, RBM14, PUF60, PHF6	164	4719	18082	1	0.3381724	27.14718
GOTERM_BP_ALL	GO:0000028~ribosomal small subunit assembly	3	1.6216	0.0180484	14.38123012	RPSA, RPS27L, RPL38	164	23	18082	1	0.3382651	27.374492
GOTERM_BP_ALL	GO:0006457~protein folding	6	3.2432	0.0185425	3.914417665	PTGES3, PPIG, CLGN, FKBP6, PPIL4, PPIL3	164	169	18082	1	0.3431732	28.013607
GOTERM_BP_ALL	GO:0010564~regulation of cell cycle process	11	5.9459	0.0187193	2.350420684	TICRR, LARP7, CENPV, CIRBP, CDC20, RPS27L, CDC5L, HMGN5, RBM14, CCAR2, PRPF40A	164	516	18082	1	0.34325	28.24102
GOTERM_BP_ALL	GO:0051726~regulation of cell cycle	15	8.1081	0.0187807	1.983023337	GTPBP4, TICRR, GMNN, RPS27L, CDC20, CDC5L, HMGN5, SON, SRSF5, LARP7, CENPV, CIRBP, RBM14, CCAR2, PRPF40A	164	834	18082	1	0.3416213	28.31989

GOTERM_BP_ALL	GO:0031323~regulation of cellular metabolic process	65	35.135	0.0194072	1.263290383	NKAP, TEX101, U2AF2, RBM3, YBX3, HMGN5, NSRP1, CBX5, PNN, HIST2H2AB, DDX17, FUBP3, HSF5, PIWIL1, MUP18, H2AFX, CCAR2, KHDRBS1, KNG1, RBFOX2, GTPBP4, APCS, HIST1H1C, PTBP1, SF1, CDC5L, BASP1, HNRNPR, HNRNPU, SMN1, NCK2, CHGA, EIF2AK1, MED15, LARP7, HSPB1, HBB-BS, RBM39, TICRR, HSPA1B, PAXBP1, RPL38, ROPN1L, SERPINA1B, SQSTM1, CACYBP, DDX41, HIST1H4J, RBM25, SREK1, GMNN, MYO1D, ILF3, CDC20, DDX5, RBMX, ANXA2, SON, SRSF5, TRPS1, PSPC1, CIRBP, RBM14, PUF60, PHF6	164	5673	18082	1	0.348271	29.11933
GOTERM_BP_ALL	GO:1990748~cellular detoxification	3	1.6216	0.019574	13.7820122	HBA-A1, GSTM2, HBB-BS	164	24	18082	1	0.3481577	29.330789
GOTERM_BP_ALL	GO:0031326~regulation of cellular biosynthetic process	50	27.027	0.0204027	1.331595381	NKAP, RBM3, YBX3, HMGN5, PNN, CBX5, HIST2H2AB, DDX17, FUBP3, HSF5, PIWIL1, MUP18, H2AFX, CCAR2, KHDRBS1, RBFOX2, GTPBP4, HIST1H1C, PTBP1, SF1, CDC5L, BASP1, HNRNPR, HNRNPU, NCK2, EIF2AK1, MED15, LARP7, HSPB1, HBB-BS, RBM39, TICRR, PAXBP1, RPL38, SQSTM1, CACYBP, DDX41, HIST1H4J, GMNN, ILF3, DDX5, RBMX, SON, SRSF5, TRPS1, PSPC1, CIRBP, RBM14, PUF60, PHF6	164	4140	18082	1	0.3574271	30.372527
GOTERM_BP_ALL	GO:0042255~ribosome assembly	4	2.1622	0.0208982	6.784990619	RPSA, RPS27L, RPL38, MRTO4	164	65	18082	1	0.3618106	30.988435

GOTERM_BP_ALL	GO:0009987~cellular process	154	83.243	0.0220502	1.056395136	TEX101, MRPS36, S100A4, LDHA, PRPF4B, PDLIM5, U2AF2, RBM3, RPS27L, NSRP1, HMGN5, CALB1, CBX5, PNN, GSTM1, GOT2, GSTM2, TOP1, HIST1H2BM, DDX17, H13, TBC1D14, U2AF1, LUC7L2, SRRM1, PDHA2, H2AFX, DDX21, SREK1IP1, LUC7L3, CCAR2, RBFOX2, GTPBP4, STK24, PTBP1, POLB, MFGE8, BASP1, MARK3, HNRNPR, HNRNPU, MRTO4, EIF2AK1, MED15, SIPA1L1, HSPB1, RNF24, SLU7, HBB-BS, SRI, SNX5, TICRR, WDR60, MUM1, HSPA1B, RIOK1, SERPINH1, HNRNPM, DDX46, SERPINA1B, POLE3, FRG1, CACYBP, PPIL4, PPIL3, STRBP, IGKC, ARL6IP4, DDX41, SDF4, HIST1H4J, ERCC6L, PRPF40A, DHX8, RPSA, EPB41, CRIP2, SREK1, MYO1D, CDC20, DDX5, AK6, RBMX, SRSF5, PPIG, CLGN, TRPS1, CIRBP, PUF60, PHF6, PTGES3, NKAP, HELQ, CRNKL1, U2SURP, YBX3, NAP1L1, INSL6, HIST2H2AB, FUBP3, MUP18, PIWIL1, HSF5, GKAP1, TWISTNB, CEACAM2, LDAH, KHDRBS1, KNG1, NLRP5, APCS, HIST1H1C, LDHAL6B, G3BP1, SF1, DECR1, CDC5L, SMN1, NCK2, HIST1H1T, CHGA, LARP7, CFAP20, CPSF6, RBM39, PRPF38B, PRPF38A, FKBP6, VIM, RPL38, PAXBP1, SF3B6, ROPN1L, SQSTM1, NUDT21, GSTO1, ACAA1B, RBM25, HIST1H2BA, DCTD, GMNN, ILF3, SF3A2, FBL, ANXA2, HBA-A1, SON, RPL23, CENPV, PSPC1, LUC7L, RBM14, PGK2, RBM17	164	16073	18082	1	0.3749947	32.400532
GOTERM_BP_ALL	GO:0006364~rRNA processing	6	3.2432	0.0239911	3.654898262	RPSA, GTPBP4, FRG1, DDX21, FBL, MRTO4	164	181	18082	1	0.3979544	34.718416
GOTERM_BP_ALL	GO:0051252~regulation of RNA metabolic process	44	23.784	0.0239923	1.356998124	NKAP, U2AF2, YBX3, PAXBP1, HMGN5, NSRP1, PNN, CBX5, HIST2H2AB, FUBP3, DDX17, SQSTM1, HSF5, MUP18, H2AFX, DDX41, HIST1H4J, CCAR2, RBM25, KHDRBS1, RBFOX2, HIST1H1C, GMNN, SREK1, PTBP1, SF1, ILF3, CDC5L, BASP1, DDX5, RBMX, HNRNPR, SMN1, NCK2, SRSF5, SON, MED15, TRPS1, LARP7, PSPC1, RBM39, RBM14, PUF60, PHF6	164	3575	18082	1	0.3953297	34.719752
GOTERM_BP_ALL	GO:0009889~regulation of biosynthetic process	50	27.027	0.0258074	1.313510812	NKAP, RBM3, YBX3, HMGN5, PNN, CBX5, HIST2H2AB, DDX17, FUBP3, HSF5, PIWIL1, MUP18, H2AFX, CCAR2, KHDRBS1, RBFOX2, GTPBP4, HIST1H1C, PTBP1, SF1, CDC5L, BASP1, HNRNPR, HNRNPU, NCK2, EIF2AK1, MED15, LARP7, HSPB1, HBB-BS, RBM39, TICRR, PAXBP1, RPL38, SQSTM1, CACYBP, DDX41, HIST1H4J, GMNN, ILF3, DDX5, RBMX, SON, SRSF5, TRPS1, PSPC1, CIRBP, RBM14, PUF60, PHF6	164	4197	18082	1	0.4155036	36.819326

GOTERM_BP_ALL	GO:0016072~rRNA metabolic process	6	3.2432	0.0273181	3.537628799	RPSA, GTPBP4, FRG1, DDX21, FBL, MRTO4	164	187	18082	1	0.4311038	38.517991
GOTERM_BP_ALL	GO:0098754~detoxification	3	1.6216	0.0279634	11.4058032	HBA-A1, GSTM2, HBB-BS	164	29	18082	1	0.4360096	39.230366
GOTERM_BP_ALL	GO:0006446~regulation of translational initiation	4	2.1622	0.0282552	6.041430003	KHDRBS1, NCK2, EIF2AK1, HBB-BS	164	73	18082	1	0.4367084	39.549951
GOTERM_BP_ALL	GO:0031123~RNA 3'-end processing	4	2.1622	0.0302825	5.880325203	RPSA, NUDT21, CPSF6, FBL	164	75	18082	1	0.4570231	41.726811
GOTERM_BP_ALL	GO:0033120~positive regulation of RNA splicing	3	1.6216	0.0335695	10.33650915	SRSF5, U2AF2, SMN1	164	32	18082	1	0.4896004	45.100019
GOTERM_BP_ALL	GO:0007049~cell cycle	21	11.351	0.0349727	1.607901423	KHDRBS1, GTPBP4, FKBP6, TICRR, STK24, GMNN, CDC20, RPS27L, CDC5L, HMGN5, SON, CLGN, LARP7, PIWIL1, CENPV, CIRBP, H2AFX, RBM14, CCAR2, ERCC6L, PRPF40A	164	1440	18082	1	0.5011651	46.483179
GOTERM_BP_ALL	GO:0080090~regulation of primary metabolic process	63	34.054	0.0354024	1.233990788	NKAP, TEX101, U2AF2, RBM3, YBX3, HMGN5, NSRP1, CBX5, PNN, HIST2H2AB, DDX17, FUBP3, HSF5, PIWIL1, MUP18, H2AFX, CCAR2, KHDRBS1, KNG1, RBFOX2, GTPBP4, HIST1H1C, PTBP1, SF1, CDC5L, BASP1, HNRNPR, SMN1, HNRNPU, NCK2, CHGA, EIF2AK1, MED15, LARP7, HSPB1, HBB-BS, RBM39, TICRR, HSPA1B, PAXBP1, RPL38, ROPN1L, SERPINA1B, SQSTM1, CACYBP, DDX41, HIST1H4J, RBM25, SREK1, GMNN, ILF3, CDC20, DDX5, RBMX, ANXA2, SON, SRSF5, TRPS1, PSPC1, CIRBP, RBM14, PUF60, PHF6	164	5629	18082	1	0.5026707	46.90016
GOTERM_BP_ALL	GO:0030317~sperm motility	4	2.1622	0.0356768	5.512804878	HIST1H1T, INSL6, ROPN1L, PGK2	164	80	18082	1	0.502612	47.164836
GOTERM_BP_ALL	GO:0030520~intracellular estrogen receptor signaling pathway	3	1.6216	0.0375291	9.728479197	RBFOX2, DDX17, DDX5	164	34	18082	1	0.5178543	48.918991
GOTERM_BP_ALL	GO:0007623~circadian rhythm	6	3.2432	0.0396527	3.195828915	TOP1, MUP18, DDX5, RBM14, HNRNPR, HNRNPU	164	207	18082	1	0.5349268	50.862598

GOTERM_BP_ALL	GO:1901564~organonitrogen compound metabolic process	27	14.595	0.0431469	1.45998756	LDHA, TICRR, RBM3, RPS27L, RPL38, HMGN5, GOT2, GSTM1, GSTM2, H13, PDHA2, GSTO1, KHDRBS1, DCTD, RPSA, PTBP1, ILF3, AK6, HNRNPR, NCK2, CHGA, EIF2AK1, RPL23, CIRBP, HBB-BS, RBM14, PGK2	164	2039	18082	1	0.5630824	53.909518
GOTERM_BP_ALL	GO:0034660~ncRNA metabolic process	9	4.8649	0.0437303	2.281160639	RPSA, TOP1, GTPBP4, FKBP6, FRG1, PIWIL1, DDX21, FBL, MRTO4	164	435	18082	1	0.5652363	54.400529
GOTERM_BP_ALL	GO:0010999~regulation of eIF2 alpha phosphorylation by heme	2	1.0811	0.0442719	44.10243902	EIF2AK1, HBB-BS	164	5	18082	1	0.5670001	54.851976
GOTERM_BP_ALL	GO:0007339~binding of sperm to zona pellucida	3	1.6216	0.0459481	8.704428755	HIST1H1T, CLGN, HSPA1B	164	38	18082	1	0.5780325	56.222489
GOTERM_BP_ALL	GO:0034654~nucleobase-containing compound biosynthetic process	46	24.865	0.0483216	1.283345265	NKAP, YBX3, PAXBP1, HMGN5, PNN, CBX5, HIST2H2AB, TOP1, FUBP3, DDX17, SQSTM1, HSF5, MUP18, H2AFX, DDX21, DDX41, TWISTNB, HIST1H4J, CCAR2, DCTD, KHDRBS1, RBFOX2, HIST1H1C, GMNN, PTBP1, SF1, POLB, ILF3, CDC5L, BASP1, DDX5, RBMX, AK6, SMN1, HNRNPU, NCK2, SRSF5, SON, MED15, TRPS1, LARP7, PSPC1, RBM39, RBM14, PUF60, PHF6	164	3952	18082	1	0.5940956	58.096284
GOTERM_BP_ALL	GO:0097659~nucleic acid-templated transcription	42	22.703	0.048875	1.305173646	NKAP, YBX3, PAXBP1, HMGN5, PNN, CBX5, HIST2H2AB, TOP1, FUBP3, DDX17, SQSTM1, HSF5, MUP18, H2AFX, DDX21, DDX41, TWISTNB, HIST1H4J, CCAR2, KHDRBS1, RBFOX2, HIST1H1C, GMNN, PTBP1, SF1, ILF3, CDC5L, BASP1, DDX5, RBMX, SMN1, NCK2, SRSF5, SON, MED15, TRPS1, LARP7, PSPC1, RBM39, RBM14, PUF60, PHF6	164	3548	18082	1	0.5956065	58.522117
GOTERM_BP_ALL	GO:0048511~rhythmic process	8	4.3243	0.0518629	2.371098872	TOP1, MUP18, PSPC1, DDX5, RBM14, HNRNPR, HNRNPU, CCAR2	164	372	18082	1	0.615201	60.751836

GOTERM_BP_ALL	GO:0032774~RNA biosynthetic process	42	22.703	0.0521325	1.298949817	NKAP, YBX3, PAXBP1, HMGN5, PNN, CBX5, HIST2H2AB, TOP1, FUBP3, DDX17, SQSTM1, HSF5, MUP18, H2AFX, DDX21, DDX41, TWISTNB, HIST1H4J, CCAR2, KHDRBS1, RBFOX2, HIST1H1C, GMNN, PTBP1, SF1, ILF3, CDC5L, BASP1, DDX5, RBMX, SMN1, NCK2, SRSF5, SON, MED15, TRPS1, LARP7, PSPC1, RBM39, RBM14, PUF60, PHF6	164	3565	18082	1	0.6144256	60.947332
GOTERM_BP_ALL	GO:0035036~sperm-egg recognition	3	1.6216	0.0526716	8.067519334	HIST1H1T, CLGN, HSPA1B	164	41	18082	1	0.6155979	61.335584
GOTERM_BP_ALL	GO:0060315~negative regulation of ryanodine-sensitive calcium-release channel activity	2	1.0811	0.0528897	36.75203252	SRI, GSTO1	164	6	18082	1	0.6144664	61.491643
GOTERM_BP_ALL	GO:0051262~protein tetramerization	5	2.7027	0.052907	3.533849281	NUDT21, CPSF6, DECR1, HIST1H4J, ANXA2	164	156	18082	1	0.6119177	61.50397
GOTERM_BP_ALL	GO:0051281~positive regulation of release of sequestered calcium ion into cytosol	3	1.6216	0.0549861	7.87543554	TEX101, SRI, GSTO1	164	42	18082	1	0.6238326	62.961412
GOTERM_BP_ALL	GO:0018130~heterocycle biosynthetic process	46	24.865	0.0592678	1.26509865	NKAP, YBX3, PAXBP1, HMGN5, PNN, CBX5, HIST2H2AB, TOP1, FUBP3, DDX17, SQSTM1, HSF5, MUP18, H2AFX, DDX21, DDX41, TWISTNB, HIST1H4J, CCAR2, DCTD, KHDRBS1, RBFOX2, HIST1H1C, GMNN, PTBP1, SF1, POLB, ILF3, CDC5L, BASP1, DDX5, RBMX, AK6, SMN1, HNRNPU, NCK2, SRSF5, SON, MED15, TRPS1, LARP7, PSPC1, RBM39, RBM14, PUF60, PHF6	164	4009	18082	1	0.6496006	65.800481
GOTERM_BP_ALL	GO:0006259~DNA metabolic process	14	7.5676	0.0596254	1.732419041	TOP1, GTPBP4, HELQ, FKBP6, TICRR, GMNN, CACYBP, MUM1, RPS27L, POLB, H2AFX, CDC5L, HIST1H4J, HNRNPU	164	891	18082	1	0.6492651	66.0281
GOTERM_BP_ALL	GO:0030099~myeloid cell differentiation	8	4.3243	0.0608029	2.285100468	NKAP, HBA-A1, RBFOX2, APCS, EIF2AK1, HBB-BS, HIST1H4J, ANXA2	164	386	18082	1	0.6540806	66.767448
GOTERM_BP_ALL	GO:0000077~DNA damage checkpoint	4	2.1622	0.0617688	4.410243902	H2AFX, RPS27L, CDC5L, CCAR2	164	100	18082	1	0.6574769	67.362549

GOTERM_BP_ALL	GO:0019438~aromatic compound biosynthetic process	46	24.865	0.0626508	1.260069686	NKAP, YBX3, PAXBP1, HMGN5, PNN, CBX5, HIST2H2AB, TOP1, FUBP3, DDX17, SQSTM1, HSF5, MUP18, H2AFX, DDX21, DDX41, TWISTNB, HIST1H4J, CCAR2, DCTD, KHDRBS1, RBFOX2, HIST1H1C, GMNN, PTBP1, SF1, POLB, ILF3, CDC5L, BASP1, DDX5, RBMX, AK6, SMN1, HNRNPU, NCK2, SRSF5, SON, MED15, TRPS1, LARP7, PSPC1, RBM39, RBM14, PUF60, PHF6	164	4025	18082	1	0.6602925	67.897188
GOTERM_BP_ALL	GO:0044419~interspecies interaction between organisms	11	5.9459	0.0640117	1.89799229	APCS, CRNKL1, FKBP6, SQSTM1, PTBP1, HSPB1, DDX21, ILF3, DDX5, DDX41, CEACAM2	164	639	18082	1	0.6658962	68.705973
GOTERM_BP_ALL	GO:0044403~symbiosis, encompassing mutualism through parasitism	11	5.9459	0.0640117	1.89799229	APCS, CRNKL1, FKBP6, SQSTM1, PTBP1, HSPB1, DDX21, ILF3, DDX5, DDX41, CEACAM2	164	639	18082	1	0.6658962	68.705973
GOTERM_BP_ALL	GO:0048869~cellular developmental process	50	27.027	0.068303	1.234670745	NKAP, PDLIM5, INSL6, DDX17, PIWIL1, MUP18, DDX21, H2AFX, LDAH, RBFOX2, GTPBP4, APCS, STK24, PTBP1, SF1, BASP1, CDC5L, SMN1, HNRNPU, NCK2, HIST1H1T, EIF2AK1, SIPA1L1, CFAP20, HSPB1, HBB-BS, FKBP6, VIM, SERPINH1, ROPN1L, SQSTM1, CACYBP, STRBP, IGKC, DDX41, HIST1H4J, SDF4, PRPF40A, HIST1H2BA, RPSA, EPB41, CDC20, SF3A2, DDX5, RBMX, FBL, ANXA2, HBA-A1, CLGN, TRPS1	164	4465	18082	1	0.6878992	71.132241
GOTERM_BP_ALL	GO:0030518~intracellular steroid hormone receptor signaling pathway	4	2.1622	0.0725157	4.121723273	PTGES3, RBFOX2, DDX17, DDX5	164	107	18082	1	0.7078795	73.340663
GOTERM_BP_ALL	GO:0006260~DNA replication	6	3.2432	0.0782614	2.62514518	TOP1, GTPBP4, TICRR, GMNN, CACYBP, POLB	164	252	18082	1	0.7337102	76.09689
GOTERM_BP_ALL	GO:0006275~regulation of DNA replication	4	2.1622	0.0806686	3.93771777	GTPBP4, TICRR, GMNN, CACYBP	164	112	18082	1	0.7424296	77.169761

GOTERM_BP_ALL	GO:0033554~cellular response to stress	22	11.892	0.0817358	1.435286477	HELQ, CRNKL1, STK24, TICRR, YBX3, MUM1, POLB, RPS27L, CDC5L, DDX5, NCK2, GSTM2, EIF2AK1, H13, SQSTM1, HSPB1, CIRBP, SLU7, HBB-BS, H2AFX, CCAR2, PGK2	164	1690	18082	1	0.7448815	77.630748
GOTERM_BP_ALL	GO:0010524~positive regulation of calcium ion transport into cytosol	3	1.6216	0.0826015	6.240911183	TEX101, SRI, GSTO1	164	53	18082	1	0.7463983	77.998214
GOTERM_BP_ALL	GO:0000075~cell cycle checkpoint	5	2.7027	0.0852229	2.996089608	TICRR, H2AFX, RPS27L, CDC5L, CCAR2	164	184	18082	1	0.7554174	79.076544
GOTERM_BP_ALL	GO:0016032~viral process	10	5.4054	0.0857873	1.871920162	APCS, CRNKL1, FKBP6, PTBP1, HSPB1, DDX21, ILF3, DDX5, DDX41, CEACAM2	164	589	18082	1	0.7555372	79.302112
GOTERM_BP_ALL	GO:0007283~spermatogenesis	10	5.4054	0.0857873	1.871920162	HIST1H2BA, HIST1H1T, CLGN, FKBP6, YBX3, PIWIL1, H2AFX, STRBP, INSL6, ROPN1L	164	589	18082	1	0.7555372	79.302112
GOTERM_BP_ALL	GO:0071822~protein complex subunit organization	19	10.27	0.0864333	1.481517577	HIST1H2BA, NLRP5, APCS, EPB41, HIST1H1C, GMNN, NAP1L1, DECR1, SERPINH1, SMN1, ANXA2, NCK2, HIST1H2BM, HIST1H1T, CLGN, SQSTM1, NUDT21, CPSF6, HIST1H4J	164	1414	18082	1	0.7559969	79.557461
GOTERM_BP_ALL	GO:0048232~male gamete generation	10	5.4054	0.0864752	1.868747416	HIST1H2BA, HIST1H1T, CLGN, FKBP6, YBX3, PIWIL1, H2AFX, STRBP, INSL6, ROPN1L	164	590	18082	1	0.7539414	79.573928
GOTERM_BP_ALL	GO:1901362~organic cyclic compound biosynthetic process	46	24.865	0.0882089	1.228331433	NKAP, YBX3, PAXBP1, HMGN5, PNN, CBX5, HIST2H2AB, TOP1, FUBP3, DDX17, SQSTM1, HSF5, MUP18, H2AFX, DDX21, DDX41, TWISTNB, HIST1H4J, CCAR2, DCTD, KHDRBS1, RBFOX2, HIST1H1C, GMNN, PTBP1, SF1, POLB, ILF3, CDC5L, BASP1, DDX5, RBMX, AK6, SMN1, HNRNPU, NCK2, SRSF5, SON, MED15, TRPS1, LARP7, PSPC1, RBM39, RBM14, PUF60, PHF6	164	4129	18082	1	0.7588795	80.244106
GOTERM_BP_ALL	GO:0010628~positive regulation of gene expression	22	11.892	0.0889103	1.420160507	KHDRBS1, PTGES3, RBM3, U2AF2, VIM, PTBP1, ILF3, PAXBP1, DDX5, HMGN5, RBMX, HNRNPU, SMN1, NCK2, FUBP3, SRSF5, DDX17, MUP18, CIRBP, RBM14, DDX41, HIST1H4J	164	1708	18082	1	0.759534	80.509297

GOTERM_BP_ALL	GO:0044764~multi-organism cellular process	10	5.4054	0.08926	1.856163259	APCS, CRNKL1, FKBP6, PTBP1, HSPB1, DDX21, ILF3, DDX5, DDX41, CEACAM2	164	594	18082	1	0.7587679	80.640276
GOTERM_BP_ALL	GO:0010975~regulation of neuron projection development	9	4.8649	0.0932231	1.934317501	STK24, PDLIM5, SIPA1L1, VIM, PTBP1, HSPB1, CDC20, SF3A2, SMN1	164	513	18082	1	0.7721256	82.067603
GOTERM_BP_ALL	GO:0042178~xenobiotic catabolic process	2	1.0811	0.0948338	20.04656319	GSTM2, GSTO1	164	11	18082	1	0.7760693	82.618848
GOTERM_BP_ALL	GO:1901566~organonitrogen compound biosynthetic process	18	9.7297	0.0957757	1.482158145	DCTD, KHDRBS1, RPSA, TICRR, RBM3, PTBP1, ILF3, RPS27L, RPL38, AK6, HNRNPR, GOT2, NCK2, EIF2AK1, RPL23, CIRBP, HBB-BS, RBM14	164	1339	18082	1	0.7774633	82.933751
GOTERM_BP_ALL	GO:0043414~macromolecule methylation	6	3.2432	0.099603	2.432119799	HIST1H1C, FKBP6, ILF3, PAXBP1, HIST1H4J, FBL	164	272	18082	1	0.7890862	84.15883
