# Supplementary material for: Integrative proteome analysis implicates aberrant RNA splicing in impaired developmental potential of aged mouse oocytes
Source: Aging Cell. 2021 Sep 28;20(10):e13482. doi: 10.1111/acel.13482 (PMC8520726; doi:10.1111/acel.13482)
Supplement: Supplementary file 7 — Table S3 [file ACEL-20-e13482-s006.pdf]

Table S3. KEGG pathway analysis of 187 DE proteins.

Category	Term	Count	%	PValue	Genes	List Total	Pop Hits	Pop Total	Fold Enrichment	Bonferroni	Benjamini	FDR
KEGG_PATHWAY	mmu03040:Spliceosome	23	12.43	4.41E-22	DHX8, CRNKL1, U2AF2, U2SURP, CDC5L, SF3B6, HSPA1B, SF3A2, DDX5, RBMX, HNRNPU, HNRNPM, SRSF5, DDX46, U2AF1, RBMXL2, SLU7, RBM25, PUF60, PRPF38B, PRPF38A, PRPF40A, RBM17	75	133	7720	17.800501	3.57E-20	3.57E-20	4.70E-19
KEGG_PATHWAY	mmu03015:mRNA surveillance pathway	5	2.703	0.01331198	NUDT21, SRRM1, CPSF6, SAP18B, PNN	75	96	7720	5.3611111	0.6622712	0.4188556	13.30924
KEGG_PATHWAY	mmu01130:Biosynthesis of antibiotics	7	3.784	0.01638173	GOT2, LDHA, LDHAL6B, PDHA2, ACAA1B, AK6, PGK2	75	214	7720	3.3669782	0.7376062	0.3597968	16.14082
KEGG_PATHWAY	mmu00010:Glycolysis / Gluconeogenesis	4	2.162	0.02514614	LDHA, LDHAL6B, PDHA2, PGK2	75	66	7720	6.2383838	0.8729131	0.40293	23.77019
KEGG_PATHWAY	mmu03008:Ribosome biogenesis in eukaryotes	4	2.162	0.04503193	GTPBP4, RIOK1, AK6, FBL	75	83	7720	4.9606426	0.9760615	0.5259552	38.80236
KEGG_PATHWAY	mmu05322:Systemic lupus erythematosus	5	2.703	0.05213936	HIST1H2BA, HIST2H2AB, HIST1H2BM, H2AFX, HIST1H4J	75	147	7720	3.5011338	0.9869292	0.5146549	43.48569
KEGG_PATHWAY	mmu00620:Pyruvate metabolism	3	1.622	0.05348282	LDHA, LDHAL6B, PDHA2	75	39	7720	7.9179487	0.9883478	0.4706138	44.33354
KEGG_PATHWAY	mmu00270:Cysteine and methionine metabolism	3	1.622	0.05595676	GOT2, LDHA, LDHAL6B	75	40	7720	7.72	0.9905737	0.4417961	45.86472
KEGG_PATHWAY	mmu00480:Glutathione metabolism	3	1.622	0.09730901	GSTM1, GSTM2, GSTO1	75	55	7720	5.6145455	0.9997496	0.6020285	66.41342
