# Supplementary material for: Integrative proteome analysis implicates aberrant RNA splicing in impaired developmental potential of aged mouse oocytes
Source: Aging Cell. 2021 Sep 28;20(10):e13482. doi: 10.1111/acel.13482 (PMC8520726; doi:10.1111/acel.13482)
Supplement: Supplementary file 8 — Table S4 [file ACEL-20-e13482-s002.pdf]

Table S4. List of alternative splicing events and genes at the 2-cell stage (10-12m versus 8-10w).

Group	Event	Gene_name	Gene_id	chrom	strand	riExonStart_0base	riExonEnd	upstreamES	upstreamEE	downstreamES	downstreamEE	ID	IJC_SAMPLE_1	SJC_SAMPLE_1	IJC_SAMPLE_2	SJC_SAMPLE_2	IncFormLen	SkipFormLen	PValue	FDR	IncLevel1	IncLevel2	IncLevelDifference
10-12m_vs_8-10w	A3SS	Gtf2ird1	ENSMUSG00000023079.14	chr5	-	134382529	134382685	134382529	134382586	134382780	134382830	121	9,2	10,19	0,1	15,11	247	149	0.000491403754149	0.0254570348339	0.352,0.06	0.0,0.052	0.18
10-12m_vs_8-10w	A3SS	Reps1	ENSMUSG00000019854.17	chr10	+	18107667	18107748	18107670	18107748	18104153	18104275	134	85,131	4,12	115,117	27,28	151	149	4.28027484695e-05	0.00638902358821	0.954,0.915	0.808,0.805	0.128
10-12m_vs_8-10w	A3SS	Chfr	ENSMUSG00000014668.15	chr5	+	110158798	110158918	110158801	110158918	110154836	110154982	255	1,4	0,1	5,4	7,6	151	149	0.000405237583915	0.0232647935996	1.0,0.798	0.413,0.397	0.494
10-12m_vs_8-10w	A3SS	Ncaph2	ENSMUSG00000008690.15	chr15	+	89370600	89370675	89370603	89370675	89370394	89370488	427	0,0	6,8	4,3	11,5	151	149	4.23382855413e-05	0.00638902358821	0.0,0.0	0.264,0.372	-0.318
10-12m_vs_8-10w	A3SS	Trappc8	ENSMUSG00000033382.14	chr18	-	20865949	20866206	20865949	20866203	20867759	20867853	499	0,1	18,26	3,6	19,23	151	149	0.000368581468933	0.02171720813	0.0,0.037	0.135,0.205	-0.152
10-12m_vs_8-10w	A3SS	Ilf3	ENSMUSG00000032178.14	chr9	+	21397968	21399250	21399124	21399250	21397468	21397668	622	4,10	0,0	7,1	7,2	298	149	1.01044340672e-08	1.27003269716e-05	1.0,1.0	0.333,0.2	0.734
10-12m_vs_8-10w	A3SS	Srrt	ENSMUSG00000037364.12	chr5	-	137296217	137296319	137296217	137296307	137296466	137296623	713	24,41	16,14	31,45	5,4	160	149	0.000694562115491	0.0338070560127	0.583,0.732	0.852,0.913	-0.225
10-12m_vs_8-10w	A3SS	Fam53a	ENSMUSG00000037339.17	chr5	-	33600355	33608227	33600355	33600880	33614602	33614834	732	1,0	1,1	2,2	1,0	298	149	5.1434616661e-05	0.007197631669	0.333,0.0	0.5,1.0	-0.584
10-12m_vs_8-10w	A3SS	Alkbh8	ENSMUSG00000025899.14	chr9	+	3349357	3349490	3349419	3349490	3347803	3347908	966	0,0	7,4	4,3	16,10	210	149	0.00110546066751	0.0452542129158	0.0,0.0	0.151,0.176	-0.163
10-12m_vs_8-10w	A3SS	Ice2	ENSMUSG00000032235.15	chr9	+	69407116	69407366	69407264	69407366	69400476	69400581	973	57,33	3,1	14,21	5,7	296	149	0.000134921993741	0.0120836137594	0.905,0.943	0.585,0.602	0.331
10-12m_vs_8-10w	A3SS	Khdc3	ENSMUSG00000092622.8	chr9	+	73103215	73104438	73104342	73104438	73102829	73103009	1099	16,43	1,5	27,21	0,0	298	149	0.000836773021859	0.0374706959188	0.889,0.811	1.0,1.0	-0.15
10-12m_vs_8-10w	A3SS	Odf2l	ENSMUSG00000028256.16	chr3	+	145145875	145148616	145148530	145148616	145144311	145144416	1236	5,2	7,20	0,0	7,21	298	149	0.000830399015738	0.0374706959188	0.263,0.048	0.0,0.0	0.156
10-12m_vs_8-10w	A3SS	Tmem120a	ENSMUSG00000039886.8	chr5	-	135736596	135736835	135736596	135736662	135736917	135737013	1367	68,127	0,14	184,88	2,0	298	149	0.000158848043039	0.0131726210505	1.0,0.819	0.979,1.0	-0.08
10-12m_vs_8-10w	A3SS	Stk11	ENSMUSG00000003068.16	chr10	+	80126032	80126369	80126232	80126369	80125478	80125568	1495	5,21	0,1	17,7	4,2	298	149	0.000978358897892	0.0429518739682	1.0,0.913	0.68,0.636	0.299
10-12m_vs_8-10w	A3SS	Rpl13	ENSMUSG00000000740.12	chr8	+	123102844	123103356	123103182	123103356	123102652	123102762	1823	196,321	23,32	445,311	21,16	298	149	0.00107778143285	0.0452542129158	0.81,0.834	0.914,0.907	-0.089
10-12m_vs_8-10w	A3SS	Sap130	ENSMUSG00000024260.13	chr18	+	31682006	31682184	31682009	31682184	31680368	31680671	1863	3,2	2,0	0,1	4,3	151	149	3.74801821442e-05	0.00638902358821	0.597,1.0	0.0,0.248	0.675
10-12m_vs_8-10w	A3SS	Mast2	ENSMUSG00000003810.12	chr4	-	116306761	116308337	116306761	116308334	116308646	116308840	1924	0,0	2,3	2,3	2,2	151	149	1.93933064142e-05	0.00482462367349	0.0,0.0	0.497,0.597	-0.547
10-12m_vs_8-10w	A3SS	Pard6a	ENSMUSG00000005699.16	chr8	+	105702607	105703493	105702610	105703493	105702173	105702396	2044	0,0	2,1	1,2	1,0	151	149	2.66487426055e-05	0.0054242304267	0.0,0.0	0.497,1.0	-0.749
10-12m_vs_8-10w	A3SS	Xlr4b	ENSMUSG00000067768.12	chrX	+	73215227	73215355	73215240	73215355	73214773	73214820	2077	11,15	0,1	7,6	3,2	161	149	0.00115207241456	0.0452542129158	1.0,0.933	0.683,0.735	0.257

10-12m_vs_8-10w	A3SS	Mak	ENSMUSG00000021363.14	chr13	-	41030016	41030139	41030016	41030136	41032524	41032599	2121	0,1	5,1	2,2	2,0	151	149	0.000302607054621	0.0199275645675	0.0,0.497	0.497,1.0	-0.5
10-12m_vs_8-10w	A3SS	Setd5	ENSMUSG00000034269.12	chr6	+	113110316	113110525	113110373	113110525	113109884	113109990	2260	1,5	2,0	1,0	3,1	205	149	0.000444647907751	0.0248891666364	0.267,1.0	0.195,0.0	0.536
10-12m_vs_8-10w	A3SS	Vps28	ENSMUSG00000115987.1	chr15	-	76624591	76624718	76624591	76624620	76625133	76625205	2344	9,3	5,5	2,1	16,6	246	149	0.00113321332787	0.0452542129158	0.522,0.267	0.07,0.092	0.314
10-12m_vs_8-10w	A3SS	Canx	ENSMUSG00000020368.15	chr11	-	50311603	50311784	50311603	50311780	50325492	50325673	2384	22,35	0,0	40,12	4,3	152	149	0.000740501285233	0.0352762208008	1.0,1.0	0.907,0.797	0.148
10-12m_vs_8-10w	A3SS	Fam129c	ENSMUSG00000043243.15	chr8	+	71607504	71607924	71607540	71607924	71606660	71606744	2439	1,11	0,1	5,3	4,2	184	149	0.0003437371743	0.0208007441421	1.0,0.899	0.503,0.548	0.424
10-12m_vs_8-10w	A3SS	Csf1	ENSMUSG00000014599.10	chr3	-	107748153	107749169	107748153	107748284	107750310	107750458	2653	0,0	2,2	2,2	2,0	298	149	0.00124095563952	0.0465038669045	0.0,0.0	0.333,1.0	-0.667
10-12m_vs_8-10w	A3SS	Rbm39	ENSMUSG00000027620.16	chr2	-	156167537	156167591	156167537	156167588	156167684	156167750	2673	56,93	6,9	103,74	1,2	151	149	0.000342260732134	0.0208007441421	0.902,0.911	0.99,0.973	-0.075
10-12m_vs_8-10w	A3SS	Tsc1	ENSMUSG00000026812.16	chr2	+	28678951	28679118	28678966	28679118	28678161	28678205	2677	1,3	0,1	0,0	2,3	163	149	3.44252196527e-07	0.000154156133605	1.0,0.733	0.0,0.0	0.867
10-12m_vs_8-10w	A3SS	Jmjd6	ENSMUSG00000056962.11	chr11	-	116839837	116839931	116839837	116839926	116840409	116840545	2760	2,6	0,0	6,6	4,4	153	149	1.41967467452e-05	0.00454093085179	1.0,1.0	0.594,0.594	0.406
10-12m_vs_8-10w	A3SS	Arhgap5	ENSMUSG00000035133.9	chr12	+	52542488	52542636	52542491	52542636	52516106	52519967	2782	13,12	0,6	14,15	0,0	151	149	0.00119797350046	0.0462459080609	1.0,0.664	1.0,1.0	-0.168
10-12m_vs_8-10w	A3SS	Mier2	ENSMUSG00000042570.14	chr10	-	79549536	79549853	79549536	79549679	79550315	79550406	2876	1,2	1,2	2,2	0,0	298	149	7.87473342331e-05	0.00927975164989	0.333,0.333	1.0,1.0	-0.667
10-12m_vs_8-10w	A3SS	Ap4m1	ENSMUSG00000019518.10	chr5	+	138176051	138176263	138176209	138176263	138175911	138175974	2893	1,7	2,5	3,6	0,0	298	149	6.34811372358e-08	3.55335665677e-05	0.2,0.412	1.0,1.0	-0.694
10-12m_vs_8-10w	A3SS	Folr1	ENSMUSG00000001827.12	chr7	-	101868397	101868470	101868397	101868467	101869156	101869317	2987	0,0	5,7	2,4	22,12	151	149	0.00100248211247	0.0431645663427	0.0,0.0	0.082,0.248	-0.165
10-12m_vs_8-10w	A3SS	Glyr1	ENSMUSG00000022536.14	chr16	-	5021337	5021448	5021337	5021430	5026507	5026607	2998	1,8	0,1	2,11	2,7	166	149	0.000500272234342	0.0254570348339	1.0,0.878	0.473,0.585	0.41
10-12m_vs_8-10w	A3SS	Morf4l1	ENSMUSG00000062270.13	chr9	-	90103654	90103771	90103654	90103768	90107234	90107302	3062	2,2	1,4	4,2	0,0	151	149	1.71700493382e-05	0.00480546755853	0.664,0.33	1.0,1.0	-0.503
10-12m_vs_8-10w	A3SS	Zfp740	ENSMUSG00000046897.17	chr15	+	102208235	102208361	102208271	102208361	102207770	102207920	3072	1,1	0,0	0,0	1,1	184	149	2.17999609475e-08	1.62700375205e-05	1.0,1.0	0.0,0.0	1.0
10-12m_vs_8-10w	A3SS	Celf1	ENSMUSG00000005506.16	chr2	+	91006619	91006695	91006631	91006695	91004700	91004862	3080	4,7	10,21	1,1	16,21	160	149	0.00112640533392	0.0452542129158	0.271,0.237	0.055,0.042	0.206
10-12m_vs_8-10w	A3SS	Glcci1	ENSMUSG00000029638.17	chr6	+	8573181	8573298	8573184	8573298	8537816	8537968	3191	13,23	6,5	13,18	1,0	151	149	0.000151732071255	0.0130664656746	0.681,0.819	0.928,1.0	-0.214
10-12m_vs_8-10w	A3SS	Clk2	ENSMUSG00000068917.12	chr3	+	89169614	89170115	89170048	89170115	89168694	89168923	3268	2,3	0,0	4,0	3,3	298	149	1.13446422256e-08	1.27003269716e-05	1.0,1.0	0.4,0.0	0.8
10-12m_vs_8-10w	A3SS	Nr4a2	ENSMUSG00000026826.13	chr2	-	57108560	57108739	57108560	57108618	57109062	57109265	3272	0,1	2,1	11,3	0,2	269	149	6.36161211018e-05	0.00791313861927	0.0,0.356	1.0,0.454	-0.549
10-12m_vs_8-10w	A3SS	Cdc6	ENSMUSG00000017499.15	chr11	+	98911387	98911563	98911390	98911563	98910577	98910777	3283	3,29	0,0	12,5	4,2	151	149	0.000130005382341	0.0120836137594	1.0,1.0	0.747,0.712	0.27

10-12m_vs_8-10w	A3SS	Rnf212b	ENSMUSG00000112858.2	chr14	+	54848296	54848706	54848666	54848706	54842786	54842835	3315	7,4	0,0	2,1	3,1	298	149	4.69894428412e-06	0.00175348937536	1.0,1.0	0.25,0.333	0.709
10-12m_vs_8-10w	A3SS	Cpsf4	ENSMUSG00000029625.16	chr5	+	145178757	145178928	145178835	145178928	145177229	145177302	3386	3,3	7,8	11,17	7,1	226	149	0.000796598355864	0.0371579941412	0.22,0.198	0.509,0.918	-0.504
10-12m_vs_8-10w	A3SS	RP23-233B6.2	ENSMUSG00000034560.6	chr10	+	83550880	83550948	83550886	83550948	83550263	83550309	3412	5,6	2,5	4,11	0,1	154	149	0.000297345753007	0.0199275645675	0.708,0.537	1.0,0.914	-0.335
10-12m_vs_8-10w	A3SS	Pafah1b1	ENSMUSG00000020745.15	chr11	-	74673948	74679614	74673948	74677742	74683458	74683687	3430	9,5	0,1	4,3	5,1	298	149	0.000247495438283	0.0175723130979	1.0,0.714	0.286,0.6	0.414
10-12m_vs_8-10w	A3SS	Kctd10	ENSMUSG00000001098.15	chr5	-	114368940	114369030	114368940	114369027	114370058	114370228	3715	0,1	15,16	8,2	23,10	151	149	0.000183353064474	0.0146616968342	0.0,0.058	0.256,0.165	-0.182
10-12m_vs_8-10w	A3SS	Tex43	ENSMUSG00000032900.14	chr18	+	56594447	56594782	56594608	56594782	56592376	56592485	3814	14,27	1,0	34,33	6,3	298	149	0.00124619562942	0.0465038669045	0.875,1.0	0.739,0.846	0.145
10-12m_vs_8-10w	A3SS	Map4k5	ENSMUSG00000034761.15	chr12	-	69818383	69818501	69818383	69818498	69822802	69822863	3889	2,4	0,1	1,3	6,6	151	149	2.37790914392e-05	0.00532413857324	1.0,0.798	0.141,0.33	0.664
10-12m_vs_8-10w	A3SS	Vps16	ENSMUSG00000027411.17	chr2	+	130441607	130441705	130441616	130441705	130441394	130441503	3956	2,9	0,0	9,4	1,5	157	149	0.000115360150948	0.0118269952685	1.0,1.0	0.895,0.432	0.337
10-12m_vs_8-10w	A3SS	Tmem55b	ENSMUSG00000035953.14	chr14	-	50929005	50929307	50929005	50929058	50929583	50929690	4105	0,4	8,30	6,7	8,6	298	149	0.000119424908836	0.0118269952685	0.0,0.063	0.273,0.368	-0.289
10-12m_vs_8-10w	A3SS	Kctd9	ENSMUSG00000034327.17	chr14	+	67729223	67729409	67729350	67729409	67728649	67728746	4269	8,27	16,20	5,8	34,34	275	149	0.000326651836929	0.0208007441421	0.213,0.422	0.074,0.113	0.224
10-12m_vs_8-10w	A3SS	Hectd2	ENSMUSG00000041180.13	chr19	+	36601428	36601528	36601431	36601528	36599595	36599719	4378	8,10	2,4	4,6	6,17	151	149	0.000460256038432	0.0251344700012	0.798,0.712	0.397,0.258	0.428
10-12m_vs_8-10w	A3SS	Mettl23	ENSMUSG00000090266.10	chr11	+	116845762	116845945	116845818	116845945	116843674	116843774	4525	65,82	3,7	70,44	16,13	204	149	0.000230626295786	0.0175723130979	0.941,0.895	0.762,0.712	0.181
10-12m_vs_8-10w	A3SS	Rhebl1	ENSMUSG00000023755.10	chr15	-	98878452	98879055	98878452	98878534	98879258	98879315	4533	123,206	26,42	208,173	14,19	298	149	0.000243771829246	0.0175723130979	0.703,0.71	0.881,0.82	-0.144
10-12m_vs_8-10w	A3SS	Tardbp	ENSMUSG00000041459.15	chr4	-	148612381	148617705	148612381	148617635	148618664	148618791	4577	33,81	1,1	55,74	7,9	218	149	0.000121492135407	0.0118269952685	0.958,0.982	0.843,0.849	0.124
10-12m_vs_8-10w	A3SS	Tardbp	ENSMUSG00000041459.15	chr4	-	148617565	148617737	148617565	148617635	148618664	148618791	4578	42,76	1,1	51,63	7,9	250	149	4.16150614437e-05	0.00638902358821	0.962,0.978	0.813,0.807	0.16
10-12m_vs_8-10w	A3SS	Tardbp	ENSMUSG00000041459.15	chr4	-	148617565	148617825	148617565	148617635	148618664	148618791	4579	76,121	1,1	92,113	7,9	298	149	5.93069458883e-05	0.00781107363788	0.974,0.984	0.868,0.863	0.114
10-12m_vs_8-10w	A3SS	Snrnp25	ENSMUSG00000040767.10	chr11	+	32207556	32207662	32207573	32207662	32206959	32207043	4592	212,161	5,1	220,215	12,16	165	149	0.000251145162632	0.0175723130979	0.975,0.993	0.943,0.924	0.05
10-12m_vs_8-10w	A3SS	Popdc3	ENSMUSG00000019848.14	chr10	+	45316430	45316539	45316482	45316539	45314560	45315279	4613	8,9	6,0	7,3	0,0	200	149	0.00129572475936	0.0475594710854	0.498,1.0	1.0,1.0	-0.251
10-12m_vs_8-10w	A3SS	Ilf2	ENSMUSG00000001016.12	chr3	+	90486850	90487097	90486982	90487097	90486184	90486272	5274	91,136	9,25	223,120	2,10	280	149	8.40541437742e-05	0.00940986139552	0.843,0.743	0.983,0.865	-0.131
10-12m_vs_8-10w	A3SS	Tle6	ENSMUSG00000034758.12	chr10	-	81595322	81595405	81595322	81595375	81595479	81595555	5439	2,8	2,4	9,6	0,1	178	149	0.000612718523844	0.030486150553	0.456,0.626	1.0,0.834	-0.376
10-12m_vs_8-10w	A3SS	Apmap	ENSMUSG00000033096.7	chr2	-	150588902	150589122	150588902	150589019	150590032	150590125	5475	6,5	3,0	5,1	12,10	251	149	0.000490033038213	0.0254570348339	0.543,1.0	0.198,0.056	0.645

10-12m_vs_8-10w	A5SS	Pnisr	ENSMUSG00000028248.15	chr4	+	21869515	21870405	21869515	21869615	21871437	21871608	161	19,19	0,0	29,28	3,5	298	149	7.56117571346e-05	0.00527842075997	1.0,1.0	0.829,0.737	0.217
10-12m_vs_8-10w	A5SS	Gapvd1	ENSMUSG00000026867.18	chr2	-	34717252	34717382	34717315	34717382	34715204	34715330	258	3,2	0,2	1,0	1,1	211	149	0.000787331307566	0.0256495043754	1.0,0.414	0.414,0.0	0.5
10-12m_vs_8-10w	A5SS	Prkcd	ENSMUSG00000021948.17	chr14	-	30605439	30605901	30605815	30605901	30604106	30604207	288	23,53	3,3	64,31	0,0	298	149	0.00121841865035	0.0364530967635	0.793,0.898	1.0,1.0	-0.154
10-12m_vs_8-10w	A5SS	Slc25a3	ENSMUSG00000061904.12	chr10	-	91122107	91122331	91122206	91122331	91119526	91119706	294	10,6	0,1	12,3	4,1	247	149	0.00207529330742	0.0497741432212	1.0,0.784	0.644,0.644	0.248
10-12m_vs_8-10w	A5SS	Hspd1	ENSMUSG00000025980.14	chr1	-	55087988	55088243	55088144	55088243	55086765	55086941	308	5,25	0,1	8,5	3,2	298	149	0.000326730322759	0.0151680519393	1.0,0.926	0.571,0.556	0.4
10-12m_vs_8-10w	A5SS	Catsperg1	ENSMUSG00000049676.14	chr7	-	29184678	29185090	29184963	29185090	29182236	29182329	365	3,0	5,1	7,1	0,0	298	149	4.17877665804e-09	3.84295279414e-06	0.231,0.0	1.0,1.0	-0.885
10-12m_vs_8-10w	A5SS	Catsperg1	ENSMUSG00000049676.14	chr7	-	29190212	29190789	29190726	29190789	29189946	29190027	369	16,7	1,4	20,10	0,0	298	149	0.000322543479515	0.0151680519393	0.889,0.467	1.0,1.0	-0.322
10-12m_vs_8-10w	A5SS	Camk2g	ENSMUSG00000021820.18	chr14	-	20764860	20765479	20765395	20765479	20764145	20764268	447	11,23	0,2	12,9	3,5	298	149	0.00178986828697	0.0482585916234	1.0,0.852	0.667,0.474	0.355
10-12m_vs_8-10w	A5SS	Gramd2	ENSMUSG00000074259.10	chr9	+	59712083	59712179	59712083	59712146	59713795	59713940	481	2,3	0,5	0,1	4,2	181	149	0.0016377631107	0.046172321544	1.0,0.331	0.0,0.292	0.52
10-12m_vs_8-10w	A5SS	Alg5	ENSMUSG00000036632.9	chr3	+	54738781	54738953	54738781	54738951	54739261	54739308	486	11,22	5,3	26,23	1,0	150	149	0.000272252213739	0.0147822868645	0.686,0.879	0.963,1.0	-0.199
10-12m_vs_8-10w	A5SS	Lman2l	ENSMUSG00000001143.13	chr1	-	36443490	36443609	36443519	36443609	36439608	36439726	587	3,3	0,1	2,1	2,1	177	149	0.00157897034372	0.0453876573312	1.0,0.716	0.457,0.457	0.401
10-12m_vs_8-10w	A5SS	RP23-465J13.1	ENSMUSG00000112913.1	chr10	-	65898711	65898875	65898724	65898875	65883305	65883494	679	5,10	11,10	0,1	4,12	161	149	3.87794267084e-05	0.00379004263697	0.296,0.481	0.0,0.072	0.353
10-12m_vs_8-10w	A5SS	Capn7	ENSMUSG00000021893.14	chr14	+	31360124	31361245	31360124	31360245	31363478	31363623	706	7,6	30,44	0,1	44,33	298	149	0.00019874569425	0.011654447511	0.104,0.064	0.0,0.015	0.076
10-12m_vs_8-10w	A5SS	Ndufa3	ENSMUSG00000035674.13	chr7	+	3617616	3618140	3617616	3617691	3619429	3619507	714	2,5	7,6	1,0	16,8	298	149	0.000491300675076	0.0180992548731	0.125,0.294	0.03,0.0	0.195
10-12m_vs_8-10w	A5SS	Dgke	ENSMUSG00000000276.11	chr11	-	89048848	89049002	89048888	89049002	89044239	89044311	801	4,19	22,55	3,5	61,52	188	149	0.000505945649756	0.0180992548731	0.126,0.215	0.038,0.071	0.116
10-12m_vs_8-10w	A5SS	Mycbp2	ENSMUSG00000033004.15	chr14	-	103138719	103138818	103138728	103138818	103136558	103136639	831	0,2	3,5	6,6	2,2	157	149	0.000250006090805	0.0140964972739	0.0,0.275	0.74,0.74	-0.603
10-12m_vs_8-10w	A5SS	Pan2	ENSMUSG00000005682.9	chr10	+	128304094	128304493	128304094	128304389	128308064	128308234	892	5,34	5,5	18,11	0,1	252	149	0.000344797959275	0.0151680519393	0.372,0.801	1.0,0.867	-0.347
10-12m_vs_8-10w	A5SS	RP23-359F5.1	ENSMUSG00000044551.13	chr14	-	70156160	70156518	70156415	70156518	70155849	70155953	941	0,2	2,1	8,4	0,0	298	149	5.16058314013e-05	0.00445024404908	0.0,0.5	1.0,1.0	-0.75
10-12m_vs_8-10w	A5SS	Psme2	ENSMUSG00000079197.10	chr14	-	55589480	55589705	55589618	55589705	55588443	55588512	1219	1,3	3,2	4,2	0,0	286	149	1.87921736838e-07	5.50986532409e-05	0.148,0.439	1.0,1.0	-0.707
10-12m_vs_8-10w	A5SS	Tcf4	ENSMUSG00000053477.16	chr18	+	69677966	69678129	69677966	69678117	69681568	69681798	1229	0,3	3,3	3,1	1,0	160	149	0.000356990802892	0.0151680519393	0.0,0.482	0.736,1.0	-0.627
10-12m_vs_8-10w	A5SS	Xlr4b	ENSMUSG00000067768.12	chrX	+	73214773	73214856	73214773	73214820	73215240	73215355	1237	15,17	0,1	9,9	3,2	184	149	0.00202695741152	0.0497741432212	1.0,0.932	0.708,0.785	0.22

10-12m_vs_8-10w	A5SS	Xlr4b	ENSMUSG00000067768.12	chrX	+	73214773	73214856	73214773	73214820	73216040	73216109	1238	12,12	0,0	9,8	3,2	184	149	0.000487836668645	0.0180992548731	1.0,1.0	0.708,0.764	0.264
10-12m_vs_8-10w	A5SS	Tmem234	ENSMUSG00000028797.20	chr4	+	129600982	129601243	129600982	129601134	129601869	129601961	1242	2,2	0,0	0,1	1,1	257	149	0.000431307006033	0.0174847985196	1.0,1.0	0.0,0.367	0.817
10-12m_vs_8-10w	A5SS	Cetn4	ENSMUSG00000045031.18	chr3	-	37310292	37312446	37312356	37312446	37309936	37310065	1267	51,50	0,0	27,27	2,4	298	149	0.000344555903256	0.0151680519393	1.0,1.0	0.871,0.771	0.179
10-12m_vs_8-10w	A5SS	Tecr	ENSMUSG00000031708.16	chr8	-	83573027	83573323	83573219	83573323	83572840	83572946	1314	18,60	0,0	37,23	0,6	298	149	0.00121693765724	0.0364530967635	1.0,1.0	1.0,0.657	0.172
10-12m_vs_8-10w	A5SS	Nrde2	ENSMUSG00000021179.8	chr12	-	100151210	100151334	100151225	100151334	100149816	100150050	1395	12,16	0,0	11,17	3,4	163	149	0.000185832053006	0.011654447511	1.0,1.0	0.77,0.795	0.218
10-12m_vs_8-10w	A5SS	Traf2	ENSMUSG00000026942.13	chr2	-	25538844	25539084	25538865	25539084	25537070	25537149	1442	3,4	0,1	2,2	2,2	169	149	0.000928473469877	0.0295900458009	1.0,0.779	0.469,0.469	0.421
10-12m_vs_8-10w	A5SS	Alkbh3	ENSMUSG00000040174.14	chr2	-	94010262	94010752	94010682	94010752	94008423	94008592	1453	1,2	0,0	1,2	3,1	298	149	5.64713539197e-06	0.000919855609403	1.0,1.0	0.143,0.5	0.679
10-12m_vs_8-10w	A5SS	Gpbp1	ENSMUSG00000032745.18	chr13	-	111453023	111453423	111453372	111453423	111448932	111448999	1461	972,1442	111,98	1516,1458	412,161	298	149	0.00191084720391	0.0491456491392	0.814,0.88	0.648,0.819	0.113
10-12m_vs_8-10w	A5SS	Tdrd5	ENSMUSG00000060985.15	chr1	-	156301513	156301906	156301744	156301906	156300596	156300790	1476	2,9	0,0	0,2	2,2	298	149	2.83849187266e-07	6.93538180887e-05	1.0,1.0	0.0,0.333	0.834
10-12m_vs_8-10w	A5SS	Tcf7l2	ENSMUSG00000024985.18	chr19	+	55910578	55910681	55910578	55910669	55912558	55912645	1482	2,4	5,2	4,5	1,0	160	149	0.000359709967269	0.0151680519393	0.271,0.651	0.788,1.0	-0.433
10-12m_vs_8-10w	A5SS	Dleu2	ENSMUSG00000097589.9	chr14	-	61648481	61648822	61648617	61648822	61643963	61644059	1508	13,15	39,38	5,4	37,45	284	149	0.00153920706308	0.0451295510895	0.149,0.172	0.066,0.045	0.105
10-12m_vs_8-10w	A5SS	Eif3k	ENSMUSG00000053565.10	chr7	-	28980318	28980542	28980443	28980542	28977869	28977990	1629	41,27	193,185	29,15	315,180	273	149	0.00174246448529	0.0481972251969	0.104,0.074	0.048,0.044	0.043
10-12m_vs_8-10w	A5SS	Zc3h7a	ENSMUSG00000037965.14	chr16	-	11145179	11145285	11145241	11145285	11141662	11141771	1651	4,5	0,0	9,3	2,2	210	149	0.00210504562054	0.0497741432212	1.0,1.0	0.761,0.516	0.361
10-12m_vs_8-10w	A5SS	Cdk2ap2	ENSMUSG00000024856.10	chr19	+	4097816	4097917	4097816	4097907	4098004	4098137	1686	981,1346	55,67	805,806	82,59	158	149	0.00184412784761	0.0482766325821	0.944,0.95	0.903,0.928	0.031
10-12m_vs_8-10w	A5SS	Habp4	ENSMUSG00000021476.9	chr13	+	64174789	64174858	64174789	64174845	64176004	64176088	1711	1,2	1,3	1,2	0,0	161	149	0.000103809730468	0.00691750294846	0.481,0.382	1.0,1.0	-0.569
10-12m_vs_8-10w	A5SS	Fam214a	ENSMUSG00000034858.16	chr9	+	75023584	75023746	75023584	75023653	75025650	75025847	1717	0,5	39,55	18,15	64,66	241	149	7.26373641897e-05	0.00527842075997	0.0,0.053	0.148,0.123	-0.109
10-12m_vs_8-10w	A5SS	Pprc1	ENSMUSG00000055491.13	chr19	+	46062401	46062503	46062401	46062494	46062611	46065480	1917	1,7	0,1	7,1	2,3	157	149	0.00204121019156	0.0497741432212	1.0,0.869	0.769,0.24	0.43
10-12m_vs_8-10w	A5SS	Cltc	ENSMUSG00000047126.17	chr11	-	86737058	86737278	86737070	86737278	86733575	86733844	2076	3,4	4,6	0,0	4,2	160	149	2.79702315134e-05	0.00292888281419	0.411,0.383	0.0,0.0	0.397
10-12m_vs_8-10w	A5SS	Rpe	ENSMUSG00000026005.15	chr1	+	66700830	66701028	66700830	66701017	66701616	66701648	2216	1,4	0,1	0,0	4,2	159	149	5.98863370005e-09	3.84295279414e-06	1.0,0.789	0.0,0.0	0.895
10-12m_vs_8-10w	A5SS	Rpe	ENSMUSG00000026005.15	chr1	+	66700830	66701417	66700830	66701017	66701616	66701648	2217	5,11	0,1	4,3	4,2	298	149	5.99244419011e-05	0.00488051287928	1.0,0.846	0.333,0.429	0.542
10-12m_vs_8-10w	A5SS	Lrguk	ENSMUSG00000056215.13	chr6	+	34043404	34043535	34043404	34043505	34048858	34048940	2285	7,15	0,2	3,6	4,4	178	149	0.000441294369185	0.0174847985196	1.0,0.863	0.386,0.557	0.46

10-12m_vs_8-10w	A5SS	Enox2	ENSMUSG00000031109.16	chrX	-	49023093	49023201	49023126	49023201	49018782	49018894	2301	3,9	0,0	8,5	6,1	181	149	1.79677320137e-05	0.00209954932251	1.0,1.0	0.523,0.805	0.336
10-12m_vs_8-10w	A5SS	Vps16	ENSMUSG00000027411.17	chr2	+	130437914	130438225	130437914	130438012	130438306	130438451	2353	3,9	1,4	8,4	0,0	298	149	5.1540920596e-05	0.00445024404908	0.6,0.529	1.0,1.0	-0.436
10-12m_vs_8-10w	A5SS	Slc30a5	ENSMUSG00000021629.10	chr13	-	100813297	100813506	100813464	100813506	100812647	100812805	2358	16,17	0,0	23,12	3,2	298	149	0.00104457104643	0.0325817266823	1.0,1.0	0.793,0.75	0.228
10-12m_vs_8-10w	A5SS	Ppie	ENSMUSG00000028651.12	chr4	-	123136800	123136898	123136871	123136898	123135595	123135677	2468	2,5	11,17	0,0	21,14	219	149	0.000506186527828	0.0180992548731	0.11,0.167	0.0,0.0	0.139
10-12m_vs_8-10w	A5SS	Use1	ENSMUSG00000002395.14	chr8	+	71368849	71369303	71368849	71368877	71369526	71369732	2496	6,14	4,3	38,18	0,2	298	149	0.000645342806588	0.0225255370109	0.429,0.7	1.0,0.818	-0.345
10-12m_vs_8-10w	A5SS	RP23-404I24.3	ENSMUSG00000067577.8	chr19	+	10781761	10783081	10781761	10781964	10785232	10785345	2505	0,0	1,3	1,1	0,0	298	149	7.86415987886e-09	3.84295279414e-06	0.0,0.0	1.0,1.0	-1.0
10-12m_vs_8-10w	A5SS	Ankrd27	ENSMUSG00000034867.16	chr7	+	35608320	35608458	35608320	35608453	35612462	35612555	2547	0,1	1,2	1,1	0,0	153	149	1.8618104497e-05	0.00209954932251	0.0,0.327	1.0,1.0	-0.837
10-12m_vs_8-10w	A5SS	Zfand2b	ENSMUSG00000026197.12	chr1	+	75170538	75170753	75170538	75170599	75170975	75171048	2609	2,6	0,0	4,5	2,2	298	149	0.000194034744706	0.011654447511	1.0,1.0	0.5,0.556	0.472
10-12m_vs_8-10w	A5SS	Rrnad1	ENSMUSG00000004896.16	chr3	-	87927562	87928048	87927923	87928048	87927054	87927117	2659	1,0	1,2	1,1	0,0	298	149	1.23509964312e-06	0.000226332009602	0.333,0.0	1.0,1.0	-0.834
10-12m_vs_8-10w	A5SS	Stk19	ENSMUSG00000061207.11	chr17	-	34831997	34832152	34832012	34832152	34824750	34824848	2721	1,6	21,29	0,1	58,29	163	149	0.00181052014958	0.0482585916234	0.042,0.159	0.0,0.031	0.085
10-12m_vs_8-10w	A5SS	Rfxank	ENSMUSG00000036120.10	chr8	-	70135535	70135631	70135565	70135631	70135290	70135391	2756	2,1	0,1	4,0	4,2	178	149	0.00198703703004	0.0497741432212	1.0,0.456	0.456,0.0	0.5
10-12m_vs_8-10w	A5SS	Tardbp	ENSMUSG00000041459.15	chr4	-	148618646	148618791	148618664	148618791	148617565	148617635	2768	5,2	1,1	3,2	7,9	166	149	0.000730428579493	0.0243365522167	0.818,0.642	0.278,0.166	0.508
10-12m_vs_8-10w	A5SS	Flt3l	ENSMUSG00000110206.1	chr7	-	45133881	45134026	45133897	45134026	45132227	45132412	2821	1,7	0,0	8,5	4,1	164	149	0.000349692927986	0.0151680519393	1.0,1.0	0.645,0.82	0.268
10-12m_vs_8-10w	A5SS	Eif4e1b	ENSMUSG00000074895.10	chr13	+	54784808	54785005	54784808	54784904	54785477	54785541	2919	2,11	25,54	0,1	28,20	249	149	0.000702066649567	0.0239355746108	0.046,0.109	0.0,0.029	0.063
10-12m_vs_8-10w	A5SS	Sec61a2	ENSMUSG00000025816.15	chr2	-	5876359	5876561	5876363	5876561	5874226	5874418	2927	0,0	5,2	2,6	5,6	152	149	6.59059187147e-06	0.000966180768358	0.0,0.0	0.282,0.495	-0.388
10-12m_vs_8-10w	A5SS	Brd8	ENSMUSG00000003778.14	chr18	-	34608417	34609949	34609804	34609949	34607774	34608055	2949	1,3	2,4	6,4	0,0	298	149	7.66667476171e-08	2.80983630017e-05	0.2,0.273	1.0,1.0	-0.764
10-12m_vs_8-10w	A5SS	Prss44	ENSMUSG00000032493.8	chr9	+	110814599	110814756	110814599	110814723	110815292	110815552	3051	4,6	0,1	2,1	2,3	181	149	7.4777267725e-05	0.00527842075997	1.0,0.832	0.452,0.215	0.582
10-12m_vs_8-10w	A5SS	Sipa1l3	ENSMUSG00000030583.16	chr7	-	29505289	29505460	29505293	29505460	29494552	29495081	3132	0,0	2,7	4,1	1,1	152	149	6.7112989699e-07	0.00014055377557	0.0,0.0	0.797,0.495	-0.646
10-12m_vs_8-10w	A5SS	Arhgdig	ENSMUSG00000073433.10	chr17	-	26199751	26199828	26199769	26199828	26199612	26199676	3179	20,23	5,3	36,20	1,0	166	149	0.000362129480134	0.0151680519393	0.782,0.873	0.97,1.0	-0.157
10-12m_vs_8-10w	A5SS	Gpr19	ENSMUSG00000032641.18	chr6	-	134896857	134897213	134897161	134897213	134887629	134887785	3292	4,12	1,2	2,1	9,2	298	149	1.2524515526e-05	0.00166917634192	0.667,0.75	0.1,0.2	0.559
10-12m_vs_8-10w	RI	Qars	ENSMUSG00000032604.16	chr9	+	108512482	108512778	108512482	108512582	108512699	108512778	36	1,10	6,23	2,0	28,13	265	149	0.000560310443864	0.0387397682645	0.086,0.196	0.039,0.0	0.122

10-12m_vs_8-10w	RI	Decr2	ENSMUSG00000036775.12	chr17	-	26083844	26084135	26083844	26083938	26084010	26084135	257	7,11	8,14	5,0	32,16	220	149	3.46154680989e-05	0.00768463391796	0.372,0.347	0.096,0.0	0.312
10-12m_vs_8-10w	RI	Catsperg1	ENSMUSG00000049676.14	chr7	-	29189946	29190789	29189946	29190027	29190726	29190789	440	2,2	1,4	4,1	0,0	298	149	2.52051305494e-07	0.000149214372852	0.5,0.2	1.0,1.0	-0.65
10-12m_vs_8-10w	RI	Fbxw9	ENSMUSG00000008167.14	chr8	+	85064383	85064672	85064383	85064496	85064580	85064672	491	0,0	1,4	4,1	0,0	232	149	2.74028355562e-10	4.86674359478e-07	0.0,0.0	1.0,1.0	-1.0
10-12m_vs_8-10w	RI	Syvn1	ENSMUSG00000024807.17	chr19	+	6048454	6048743	6048454	6048607	6048694	6048743	558	0,0	17,18	4,3	12,13	235	149	0.000401185935982	0.0325214382826	0.0,0.0	0.174,0.128	-0.151
10-12m_vs_8-10w	RI	Ctdnep1	ENSMUSG00000018559.16	chr11	+	69984253	69984534	69984253	69984320	69984415	69984534	824	0,0	7,8	3,2	3,6	243	149	0.000848749464838	0.0443311557615	0.0,0.0	0.38,0.17	-0.275
10-12m_vs_8-10w	RI	Phf11c	ENSMUSG00000068245.14	chr14	-	59348731	59349388	59348731	59349051	59349221	59349388	859	3,10	9,15	14,12	4,4	298	149	0.000253589236596	0.0237039202208	0.143,0.25	0.636,0.6	-0.422
10-12m_vs_8-10w	RI	Cnbp	ENSMUSG00000030057.15	chr6	-	87845121	87845557	87845121	87845320	87845464	87845557	1094	10,24	207,551	57,30	524,343	292	149	0.000312404721944	0.0277415393086	0.024,0.022	0.053,0.043	-0.025
10-12m_vs_8-10w	RI	Cnbp	ENSMUSG00000030057.15	chr6	-	87845121	87845557	87845121	87845323	87845464	87845557	1095	7,16	91,128	40,24	172,86	289	149	0.000805876325694	0.043890720365	0.038,0.061	0.107,0.126	-0.067
10-12m_vs_8-10w	RI	Trmt61a	ENSMUSG00000060950.12	chr12	+	111678104	111678962	111678104	111678161	111678602	111678962	1393	2,4	0,0	3,0	2,1	298	149	0.000500107049867	0.0370079216902	1.0,1.0	0.429,0.0	0.786
10-12m_vs_8-10w	RI	RP23-356J2.10	ENSMUSG00000025731.15	chr17	+	25876660	25877169	25876660	25876739	25876949	25877169	1591	4,1	0,0	4,4	5,1	298	149	1.75104731848e-06	0.000777465009405	1.0,1.0	0.286,0.667	0.524
10-12m_vs_8-10w	RI	Adrbk1	ENSMUSG00000024858.13	chr19	-	4287557	4287985	4287557	4287694	4287822	4287985	1606	3,5	16,19	0,0	24,10	276	149	0.000775662590919	0.043890720365	0.092,0.124	0.0,0.0	0.108
10-12m_vs_8-10w	RI	Rab3gap2	ENSMUSG00000039318.12	chr1	+	185266900	185267279	185266900	185267014	185267172	185267279	1641	1,1	0,0	6,0	1,3	298	149	5.36433826411e-05	0.00871150633386	1.0,1.0	0.75,0.0	0.625
10-12m_vs_8-10w	RI	Braf	ENSMUSG00000002413.15	chr6	-	39677377	39677641	39677377	39677472	39677571	39677641	1708	70,72	5,10	67,48	22,16	247	149	0.000418049564674	0.0325214382826	0.894,0.813	0.648,0.644	0.207
10-12m_vs_8-10w	RI	Prph	ENSMUSG00000023484.14	chr15	+	99056768	99057158	99056768	99056936	99057032	99057158	1848	2,3	1,1	1,0	2,1	244	149	0.000725662468321	0.043890720365	0.55,0.647	0.234,0.0	0.482
10-12m_vs_8-10w	RI	Jmjd6	ENSMUSG00000056962.11	chr11	-	116839837	116840545	116839837	116839926	116840409	116840545	1907	5,13	0,0	0,6	4,4	298	149	3.27496363361e-09	2.90816770665e-06	1.0,1.0	0.0,0.429	0.786
10-12m_vs_8-10w	RI	Mfsd10	ENSMUSG00000001082.12	chr5	-	34634590	34634962	34634590	34634699	34634850	34634962	1927	0,0	3,4	3,4	4,4	298	149	0.000130071771721	0.014437966661	0.0,0.0	0.273,0.333	-0.303
10-12m_vs_8-10w	RI	Cenpt	ENSMUSG00000036672.5	chr8	-	105849625	105849923	105849625	105849713	105849832	105849923	2000	6,17	3,5	4,1	11,5	267	149	9.59880665569e-05	0.0113649870803	0.527,0.655	0.169,0.1	0.456
10-12m_vs_8-10w	RI	Ufsp2	ENSMUSG00000031634.13	chr8	+	45980314	45980639	45980314	45980467	45980572	45980639	2376	4,10	2,3	8,9	0,0	253	149	9.00119034583e-05	0.0113649870803	0.541,0.663	1.0,1.0	-0.398
10-12m_vs_8-10w	RI	Cep162	ENSMUSG00000056919.9	chr9	-	87238353	87239933	87238353	87238384	87239814	87239933	2525	0,2	14,27	5,7	13,14	298	149	0.000148899309303	0.0155555984307	0.0,0.036	0.161,0.2	-0.163
10-12m_vs_8-10w	RI	Primpol	ENSMUSG00000038225.15	chr8	-	46592592	46593703	46592592	46592755	46593469	46593703	2610	1,7	9,19	1,0	26,9	298	149	0.00101022045356	0.0498375423756	0.053,0.156	0.019,0.0	0.095
10-12m_vs_8-10w	RI	Cope	ENSMUSG00000055681.14	chr8	+	70304633	70306651	70304633	70304696	70306498	70306651	2796	4,3	0,0	6,4	4,1	298	149	1.15570188801e-05	0.00410505310621	1.0,1.0	0.429,0.667	0.452

10-12m_vs_8-10w	RI	Arhgef39	ENSMUSG00000051517.14	chr4	-	43497086	43497710	43497086	43497316	43497581	43497710	2863	10,11	86,287	31,12	139,94	298	149	5.39564018426e-05	0.00871150633386	0.055,0.019	0.1,0.06	-0.043
10-12m_vs_8-10w	RI	Setdb1	ENSMUSG00000015697.14	chr3	-	95337148	95339032	95337148	95337265	95338345	95339032	2934	4,1	0,0	3,1	2,1	298	149	0.000421167275056	0.0325214382826	1.0,1.0	0.429,0.333	0.619
10-12m_vs_8-10w	RI	Slc11a1	ENSMUSG00000026177.11	chr1	+	74383624	74384173	74383624	74383744	74384023	74384173	2962	1,2	0,0	1,0	0,3	298	149	0.000246201631149	0.0237039202208	1.0,1.0	1.0,0.0	0.5
10-12m_vs_8-10w	RI	Fkbp3	ENSMUSG00000020949.9	chr12	-	65063677	65069109	65063677	65063775	65069001	65069109	3002	36,32	0,1	43,18	4,9	298	149	3.10668088643e-05	0.00768463391796	1.0,0.941	0.843,0.5	0.299
10-12m_vs_8-10w	RI	Pidd1	ENSMUSG00000025507.13	chr7	-	141439077	141439491	141439077	141439277	141439377	141439491	3072	1,3	0,2	0,0	5,1	248	149	0.000815537033809	0.043890720365	1.0,0.474	0.0,0.0	0.737
10-12m_vs_8-10w	RI	Arhgap1	ENSMUSG00000027247.16	chr2	+	91669384	91669729	91669384	91669461	91669651	91669729	3104	2,7	1,9	1,0	6,2	298	149	0.000738865084833	0.043890720365	0.5,0.28	0.077,0.0	0.352
10-12m_vs_8-10w	RI	Tardbp	ENSMUSG00000041459.15	chr4	-	148612381	148618791	148612381	148617635	148618664	148618791	3249	54,99	1,1	74,89	7,9	298	149	7.51280196984e-05	0.0111189469154	0.964,0.98	0.841,0.832	0.135
10-12m_vs_8-10w	RI	Flt3l	ENSMUSG00000110206.1	chr7	-	45132227	45134026	45132227	45132412	45133897	45134026	3281	1,3	0,0	2,7	4,1	298	149	2.94403029788e-05	0.00768463391796	1.0,1.0	0.2,0.778	0.511
10-12m_vs_8-10w	RI	Zfas1	ENSMUSG00000074578.13	chr2	+	167065433	167065862	167065433	167065480	167065686	167065862	3345	7,8	49,31	1,2	62,27	298	149	0.000672284774483	0.043890720365	0.067,0.114	0.008,0.036	0.069
10-12m_vs_8-10w	RI	Snhg17	ENSMUSG00000085385.7	chr2	-	158359181	158360599	158359181	158359432	158360458	158360599	3351	1,0	2,1	3,1	1,0	298	149	3.92416545643e-05	0.00774368650069	0.2,0.0	0.6,1.0	-0.7
10-12m_vs_8-10w	RI	H2-K1	ENSMUSG00000061232.15	chr17	-	33996826	33997157	33996826	33996859	33997037	33997157	3370	4,5	22,19	1,0	20,34	298	149	0.000746612306931	0.043890720365	0.083,0.116	0.024,0.0	0.088
10-12m_vs_8-10w	RI	Rnaseh2a	ENSMUSG00000052926.16	chr8	-	84957666	84958089	84957666	84957853	84957965	84958089	3469	6,14	0,1	17,18	5,5	260	149	0.000567136247115	0.0387397682645	1.0,0.889	0.661,0.674	0.277
10-12m_vs_8-10w	RI	Iws1	ENSMUSG00000024384.4	chr18	+	32086272	32087152	32086272	32086423	32087069	32087152	3758	10,14	114,125	4,6	173,167	298	149	0.000873643272327	0.0443311557615	0.042,0.053	0.011,0.018	0.033
10-12m_vs_8-10w	RI	Ticrr	ENSMUSG00000046591.10	chr7	+	79675271	79675713	79675271	79675408	79675509	79675713	3845	108,115	26,28	101,107	48,71	249	149	8.72447532826e-05	0.0113649870803	0.713,0.711	0.557,0.474	0.196
10-12m_vs_8-10w	SE	Nsfl1c	ENSMUSG00000027455.16	chr2	+	151503013	151503179	151500714	151500789	151504110	151504147	56	32,60	0,2	42,40	11,5	298	149	4.99299236998e-05	0.00649967603321	1.0,0.938	0.656,0.8	0.241
10-12m_vs_8-10w	SE	Tmem190	ENSMUSG00000013091.5	chr7	+	4783933	4784018	4783156	4783192	4784103	4784341	117	1,1	1,2	7,7	0,0	233	149	0.000339912366833	0.0248082469884	0.39,0.242	1.0,1.0	-0.684
10-12m_vs_8-10w	SE	Ccdc69	ENSMUSG00000049588.13	chr11	-	55051151	55051249	55049730	55050558	55052347	55052467	123	499,1332	5,6	565,562	16,8	246	149	0.000560320934552	0.0343585217816	0.984,0.993	0.955,0.977	0.022
10-12m_vs_8-10w	SE	Slc7a5	ENSMUSG00000040010.10	chr8	-	121883590	121883768	121881149	121883095	121884982	121885132	176	1,0	0,4	15,11	0,0	298	149	0.000116822803829	0.0119531182601	1.0,0.0	1.0,1.0	-0.5
10-12m_vs_8-10w	SE	Nelfb	ENSMUSG00000013465.19	chr2	-	25203886	25203982	25203140	25203247	25206159	25206345	347	1,1	0,0	3,0	3,1	244	149	1.49755935809e-05	0.00287484005634	1.0,1.0	0.379,0.0	0.811
10-12m_vs_8-10w	SE	Gpcpd1	ENSMUSG00000027346.15	chr2	-	132568619	132568716	132556934	132556976	132587620	132587729	395	0,2	1,2	4,1	0,0	245	149	0.000153251171073	0.0145258164682	0.0,0.378	1.0,1.0	-0.811
10-12m_vs_8-10w	SE	Nek4	ENSMUSG00000021918.10	chr14	+	30957261	30957369	30956880	30957078	30959140	30959295	414	3,4	0,0	6,1	1,3	256	149	0.00034025356062	0.0248082469884	1.0,1.0	0.777,0.162	0.531

10-12m_vs_8-10w	SE	RP24-534N1.1	ENSMUSG00000113017.1	chr13	+	5978082	5978217	5974340	5974480	6004940	6005008	430	13,23	6,3	12,8	0,0	283	149	1.53732672276e-05	0.00290128892322	0.533,0.801	1.0,1.0	-0.333
10-12m_vs_8-10w	SE	Rnf182	ENSMUSG00000044164.2	chr13	+	43665757	43665822	43615982	43616067	43667759	43668066	501	2,3	0,0	0,2	3,1	213	149	5.51438872365e-06	0.00141743156252	1.0,1.0	0.0,0.583	0.709
10-12m_vs_8-10w	SE	Mysm1	ENSMUSG00000062627.9	chr4	-	94961702	94961833	94960327	94960431	94965128	94965871	541	116,147	0,1	163,121	5,7	279	149	0.000478633434289	0.0312875661539	1.0,0.987	0.946,0.902	0.07
10-12m_vs_8-10w	SE	Gm815	ENSMUSG00000074913.2	chr19	+	26886378	26886484	26885924	26885996	26887754	26887855	562	51,54	0,1	13,32	5,1	254	149	0.000963391393867	0.0487010406123	1.0,0.969	0.604,0.949	0.208
10-12m_vs_8-10w	SE	Becn1	ENSMUSG00000035086.13	chr11	-	101289782	101290499	101288887	101288981	101291382	101291532	577	239,313	19,14	322,276	7,1	298	149	1.58189691554e-05	0.00294358989848	0.863,0.918	0.958,0.993	-0.085
10-12m_vs_8-10w	SE	Becn1	ENSMUSG00000035086.13	chr11	-	101290438	101290499	101288417	101288981	101291382	101291532	578	49,68	19,14	76,60	7,1	209	149	5.20096027754e-05	0.00665613190625	0.648,0.776	0.886,0.977	-0.22
10-12m_vs_8-10w	SE	Myl3	ENSMUSG00000059741.13	chr9	+	110766620	110766770	110766478	110766506	110767907	110768081	620	6,21	0,6	17,3	0,0	298	149	0.000345502816465	0.0250106108024	1.0,0.636	1.0,1.0	-0.182
10-12m_vs_8-10w	SE	Slc18b1	ENSMUSG00000037455.16	chr10	+	23810788	23810945	23805892	23806040	23816292	23816429	676	6,6	0,0	3,0	3,1	298	149	4.29568523674e-06	0.0011896756151	1.0,1.0	0.333,0.0	0.834
10-12m_vs_8-10w	SE	Chchd5	ENSMUSG00000037938.4	chr2	+	129130177	129130318	129129699	129129857	129130400	129130566	703	16,9	2,3	5,7	0,0	289	149	0.000291434552219	0.0228582230511	0.805,0.607	1.0,1.0	-0.294
10-12m_vs_8-10w	SE	Prpf40a	ENSMUSG00000061136.14	chr2	-	53159262	53159316	53157892	53157917	53159557	53159678	879	123,219	3,5	231,197	21,14	202	149	0.000295515583968	0.0229378525288	0.968,0.97	0.89,0.912	0.068
10-12m_vs_8-10w	SE	Map4k1	ENSMUSG00000037337.11	chr7	+	28996088	28996173	28994204	28994287	28999772	28999881	1278	0,0	3,1	1,1	0,0	233	149	3.12123393797e-09	6.7337642837e-06	0.0,0.0	1.0,1.0	-1.0
10-12m_vs_8-10w	SE	Map4k1	ENSMUSG00000037337.11	chr7	+	29000934	29001109	29000190	29000294	29001748	29001838	1279	0,1	2,1	5,9	2,0	298	149	3.52977391845e-05	0.00532644640401	0.0,0.333	0.556,1.0	-0.612
10-12m_vs_8-10w	SE	Mcoln1	ENSMUSG00000004567.16	chr8	+	3510813	3510915	3510539	3510689	3511687	3511810	1335	3,19	1,7	9,3	0,0	250	149	1.9152978695e-05	0.00339724559531	0.641,0.618	1.0,1.0	-0.371
10-12m_vs_8-10w	SE	Strada	ENSMUSG00000069631.14	chr11	-	106170953	106171077	106168308	106168480	106171155	106171264	1397	8,25	0,0	13,1	2,4	272	149	7.48143694396e-06	0.0017322028806	1.0,1.0	0.781,0.12	0.55
10-12m_vs_8-10w	SE	Ccdc62	ENSMUSG00000061882.12	chr5	+	123936388	123936555	123934071	123934264	123938149	123938251	1420	7,13	2,3	7,5	0,0	298	149	0.000316884448544	0.0239278956062	0.636,0.684	1.0,1.0	-0.34
10-12m_vs_8-10w	SE	Ttc14	ENSMUSG00000027677.17	chr3	+	33802858	33802943	33801190	33801390	33803074	33803204	1441	47,67	5,4	95,69	0,0	233	149	0.000114671637827	0.0118343928059	0.857,0.915	1.0,1.0	-0.114
10-12m_vs_8-10w	SE	RP23-145N17.1	ENSMUSG00000086629.8	chrX	+	108871471	108871598	108836228	108836355	108901463	108901555	1549	34,49	1,0	21,36	6,1	275	149	0.00085824554267	0.0456692027276	0.949,1.0	0.655,0.951	0.172
10-12m_vs_8-10w	SE	Scai	ENSMUSG00000035236.17	chr2	-	39082709	39082852	39080373	39080547	39083537	39083610	1559	1,9	10,19	12,15	6,10	291	149	0.000562509467749	0.0343585217816	0.049,0.195	0.506,0.434	-0.348
10-12m_vs_8-10w	SE	Mrpl55	ENSMUSG00000036860.14	chr11	+	59204174	59204255	59202485	59202659	59205648	59205927	1587	12,30	1,0	10,11	5,2	229	149	0.000158964165462	0.0149273749307	0.886,1.0	0.565,0.782	0.27
10-12m_vs_8-10w	SE	Actr6	ENSMUSG00000019948.4	chr10	-	89714869	89715008	89711970	89712560	89717072	89717244	1597	435,321	1,0	742,543	15,8	287	149	0.000560815610189	0.0343585217816	0.996,1.0	0.963,0.972	0.03
10-12m_vs_8-10w	SE	Ewsr1	ENSMUSG00000009079.16	chr11	-	5082252	5082433	5079484	5079519	5083359	5083571	1610	10,19	1,0	7,5	1,4	298	149	0.000701785798074	0.0398611704895	0.833,1.0	0.778,0.385	0.335

10-12m_vs_8-10w	SE	Gtpbp3	ENSMUSG00000007610.15	chr8	+	71489070	71489318	71488102	71488840	71489427	71489514	1633	3,13	4,8	16,7	0,1	298	149	8.62897807763e-06	0.00191040535819	0.273,0.448	1.0,0.778	-0.529
10-12m_vs_8-10w	SE	Csmd3	ENSMUSG00000022311.15	chr15	-	48292777	48292855	48285610	48287572	48359297	48359410	1708	4,2	0,0	1,0	1,1	226	149	0.00064608371676	0.0375409295269	1.0,1.0	0.397,0.0	0.802
10-12m_vs_8-10w	SE	Slc11a2	ENSMUSG00000023030.16	chr15	-	100403807	100403963	100403180	100403339	100405575	100405643	1717	8,29	0,0	20,9	2,3	298	149	0.000398077057994	0.0277454257257	1.0,1.0	0.833,0.6	0.284
10-12m_vs_8-10w	SE	Ppp1r35	ENSMUSG00000029725.10	chr5	+	137779625	137779762	137779301	137779545	137779853	137780110	1749	11,27	1,0	6,5	2,2	285	149	0.00100915640388	0.049932663762	0.852,1.0	0.611,0.567	0.337
10-12m_vs_8-10w	SE	Ppfia1	ENSMUSG00000037519.17	chr7	-	144514871	144514946	144514440	144514524	144515985	144516120	1759	4,5	4,3	0,1	7,2	223	149	0.000212055634926	0.0185891891993	0.401,0.527	0.0,0.25	0.339
10-12m_vs_8-10w	SE	Fbxo6	ENSMUSG00000055401.14	chr4	-	148146320	148146456	148145715	148146185	148146839	148146935	1770	59,108	6,2	113,85	0,0	284	149	0.000827301211993	0.0445699343889	0.838,0.966	1.0,1.0	-0.098
10-12m_vs_8-10w	SE	Tmem230	ENSMUSG00000027341.10	chr2	-	132244018	132244141	132239491	132240626	132245955	132246074	1905	153,300	0,0	86,114	6,4	271	149	5.56235320663e-05	0.00700048693404	1.0,1.0	0.887,0.94	0.087
10-12m_vs_8-10w	SE	N4bp2	ENSMUSG00000037795.14	chr5	+	65786105	65786361	65775584	65775663	65788052	65788141	1941	3,4	1,4	1,2	0,0	298	149	2.83357769082e-06	0.000886033452992	0.6,0.333	1.0,1.0	-0.534
10-12m_vs_8-10w	SE	Txndc11	ENSMUSG00000022498.16	chr16	-	11128486	11128703	11122669	11122767	11134267	11134573	2011	3,25	4,1	11,9	0,0	298	149	0.000734203598039	0.0412391283928	0.273,0.926	1.0,1.0	-0.4
10-12m_vs_8-10w	SE	Txndc11	ENSMUSG00000022498.16	chr16	-	11128486	11128705	11122669	11122767	11134267	11134650	2012	3,9	4,1	8,7	0,0	298	149	6.76332799604e-05	0.00807440017334	0.273,0.818	1.0,1.0	-0.455
10-12m_vs_8-10w	SE	Pnisr	ENSMUSG00000028248.15	chr4	+	21870351	21870405	21869515	21869615	21871437	21871608	2017	20,20	0,0	32,28	3,5	202	149	0.000207401645307	0.0182869165808	1.0,1.0	0.887,0.805	0.154
10-12m_vs_8-10w	SE	Kctd20	ENSMUSG00000005936.18	chr17	+	28962809	28962912	28961413	28961687	28963250	28963371	2185	11,22	6,2	31,8	0,0	251	149	2.64909931684e-05	0.00441482589995	0.521,0.867	1.0,1.0	-0.306
10-12m_vs_8-10w	SE	Bphl	ENSMUSG00000038286.11	chr13	+	34046785	34046952	34044423	34044527	34050280	34050323	2271	11,11	0,0	2,5	1,3	298	149	4.62975663873e-05	0.00621236548802	1.0,1.0	0.5,0.455	0.523
10-12m_vs_8-10w	SE	Nup62cl	ENSMUSG00000072944.11	chrX	-	140027567	140027702	140025228	140025340	140027838	140027888	2285	93,93	33,46	70,77	88,65	283	149	4.53194235249e-05	0.00611779162035	0.597,0.516	0.295,0.384	0.217
10-12m_vs_8-10w	SE	Gas8	ENSMUSG00000040220.10	chr8	+	123528377	123528464	123527911	123528079	123530826	123531036	2297	4,25	0,0	25,15	6,0	235	149	0.000742906655326	0.0416452135448	1.0,1.0	0.725,1.0	0.137
10-12m_vs_8-10w	SE	Alg9	ENSMUSG00000032059.13	chr9	+	50783257	50783328	50778969	50779104	50788152	50788241	2350	1,1	0,0	0,0	2,2	219	149	3.42625983141e-09	6.7337642837e-06	1.0,1.0	0.0,0.0	1.0
10-12m_vs_8-10w	SE	RP23-286I5.3	ENSMUSG00000006423.15	chrX	-	36854282	36854421	36853329	36853419	36856851	36856893	2358	44,68	1,2	44,39	7,10	287	149	4.41474584113e-05	0.00600464825593	0.958,0.946	0.765,0.669	0.235
10-12m_vs_8-10w	SE	Mkrn2	ENSMUSG00000000439.9	chr6	+	115611634	115611939	115610518	115610700	115613326	115613541	2382	71,220	2,4	151,114	10,12	298	149	0.000600409221197	0.0357078668395	0.947,0.965	0.883,0.826	0.101
10-12m_vs_8-10w	SE	Grcc10	ENSMUSG00000072772.3	chr6	-	124740518	124740695	124739182	124739413	124740919	124741374	2403	69,70	9,1	106,67	1,0	298	149	0.00027727644472	0.0220736793827	0.793,0.972	0.981,1.0	-0.108
10-12m_vs_8-10w	SE	Exoc3l2	ENSMUSG00000011263.16	chr7	+	19489607	19489763	19489051	19489174	19491655	19491777	2489	134,302	49,90	426,234	56,37	298	149	3.89229261943e-06	0.00111374648528	0.578,0.627	0.792,0.76	-0.174
10-12m_vs_8-10w	SE	Smim8	ENSMUSG00000028295.14	chr4	-	34771873	34772053	34771257	34771414	34778223	34778337	2525	7,7	1,6	11,7	0,0	298	149	6.4865981364e-06	0.00156146831806	0.778,0.368	1.0,1.0	-0.427

10-12m_vs_8-10w	SE	Smim8	ENSMUSG00000028295.14	chr4	-	34771877	34772053	34770887	34771414	34778223	34778270	2527	2,9	1,6	2,1	0,0	298	149	4.89045808072e-06	0.00132439717898	0.5,0.429	1.0,1.0	-0.536
10-12m_vs_8-10w	SE	Gadd45a	ENSMUSG00000036390.8	chr6	-	67036677	67036915	67035095	67035772	67037200	67037364	2732	19,40	4,7	44,33	0,0	298	149	1.0456252274e-06	0.000480528163216	0.704,0.741	1.0,1.0	-0.278
10-12m_vs_8-10w	SE	Gadd45a	ENSMUSG00000036390.8	chr6	-	67037009	67037111	67035465	67035772	67037200	67037251	2733	10,8	4,7	6,5	0,0	250	149	1.04148255742e-06	0.000480528163216	0.598,0.405	1.0,1.0	-0.498
10-12m_vs_8-10w	SE	Rnf216	ENSMUSG00000045078.12	chr5	-	143090119	143090758	143089742	143089819	143092994	143093125	2844	76,141	3,6	201,102	0,1	298	149	0.000942962510512	0.0482310217645	0.927,0.922	1.0,0.981	-0.066
10-12m_vs_8-10w	SE	Ncoa1	ENSMUSG00000020647.10	chr12	-	4249835	4249890	4247361	4249750	4253656	4253746	2995	38,41	1,0	50,35	5,4	203	149	0.000918315821424	0.0476142409778	0.965,1.0	0.88,0.865	0.11
10-12m_vs_8-10w	SE	Arpc1a	ENSMUSG00000029621.14	chr5	+	145096051	145096274	145092457	145092562	145097215	145097228	3003	494,686	14,35	666,493	10,7	298	149	0.000189956427192	0.0171987116214	0.946,0.907	0.971,0.972	-0.045
10-12m_vs_8-10w	SE	Arpc1a	ENSMUSG00000029621.14	chr5	+	145104441	145104517	145101056	145101269	145104697	145104891	3009	77,133	6,6	189,112	2,1	224	149	0.000873188860124	0.0459006781914	0.895,0.936	0.984,0.987	-0.07
10-12m_vs_8-10w	SE	Rad18	ENSMUSG00000030254.16	chr6	-	112644587	112644747	112619849	112620904	112665277	112665338	3042	12,20	0,0	11,5	4,1	298	149	6.36868746322e-05	0.00782059349988	1.0,1.0	0.579,0.714	0.354
10-12m_vs_8-10w	SE	Wdr45	ENSMUSG00000039382.12	chrX	+	7727346	7727492	7727169	7727271	7727749	7727842	3072	65,83	7,22	162,117	7,6	294	149	0.000282288800432	0.0223553566734	0.825,0.657	0.921,0.908	-0.174
10-12m_vs_8-10w	SE	Tdp2	ENSMUSG00000035958.3	chr13	+	24836015	24836186	24831680	24831929	24836854	24836944	3128	148,174	24,23	136,142	42,45	298	149	0.000428520071835	0.0289678928642	0.755,0.791	0.618,0.612	0.158
10-12m_vs_8-10w	SE	AC123856.3	ENSMUSG00000079455.9	chr1	+	85272811	85272929	85272358	85272509	85275105	85275253	3217	59,162	52,80	48,19	89,71	266	149	3.60977554625e-07	0.000195514468024	0.389,0.531	0.232,0.13	0.279
10-12m_vs_8-10w	SE	AC123856.3	ENSMUSG00000079455.9	chr1	+	85272811	85272961	85272358	85272509	85275105	85275384	3218	40,125	52,80	15,5	89,71	298	149	2.4436008772e-13	7.41168582064e-09	0.278,0.439	0.078,0.034	0.303
10-12m_vs_8-10w	SE	Gapvd1	ENSMUSG00000026867.18	chr2	-	34754265	34754559	34730462	34730680	34754977	34755232	3407	3,1	0,0	2,1	1,3	298	149	5.64263086023e-06	0.00143820703043	1.0,1.0	0.5,0.143	0.679
10-12m_vs_8-10w	SE	Pim2	ENSMUSG00000031155.16	chrX	+	7881247	7881620	7878301	7878556	7882072	7882249	3460	5,4	0,0	8,1	4,1	298	149	7.52964420891e-06	0.0017322028806	1.0,1.0	0.5,0.333	0.584
10-12m_vs_8-10w	SE	Ophn1	ENSMUSG00000031214.13	chrX	-	98780955	98781017	98763704	98763776	98803084	98803180	3467	101,94	0,0	71,62	5,3	210	149	0.000925838168322	0.0477196764268	1.0,1.0	0.91,0.936	0.077
10-12m_vs_8-10w	SE	Zfp280d	ENSMUSG00000038535.17	chr9	+	72329509	72329610	72329027	72329162	72330865	72331173	3664	2,6	17,20	0,0	26,21	249	149	0.000413855338617	0.0283355446402	0.066,0.152	0.0,0.0	0.109
10-12m_vs_8-10w	SE	Dip2b	ENSMUSG00000023026.16	chr15	+	100151150	100151363	100142021	100142147	100154156	100154306	3747	13,11	0,0	7,2	2,2	298	149	0.000271844163533	0.0216981719056	1.0,1.0	0.636,0.333	0.516
10-12m_vs_8-10w	SE	RP23-220D12.12	ENSMUSG00000098950.7	chr2	-	156103348	156103397	156079338	156079464	156111785	156111901	3776	4,2	0,0	2,2	5,6	197	149	5.85441541912e-08	4.6729019494e-05	1.0,1.0	0.232,0.201	0.784
10-12m_vs_8-10w	SE	RP23-220D12.12	ENSMUSG00000098950.7	chr2	-	156103348	156103429	156079338	156079464	156111785	156111926	3777	7,4	0,0	5,6	5,6	229	149	3.19942245142e-07	0.000183097513913	1.0,1.0	0.394,0.394	0.606
10-12m_vs_8-10w	SE	Csad	ENSMUSG00000023044.2	chr15	-	102178780	102178832	102177318	102177786	102178960	102179160	3783	2,0	1,2	1,3	0,0	200	149	0.000353789365608	0.0254888010647	0.598,0.0	1.0,1.0	-0.701
10-12m_vs_8-10w	SE	Csad	ENSMUSG00000023044.2	chr15	-	102202443	102202574	102200855	102201082	102203136	102203215	3794	1,0	2,1	3,1	0,0	279	149	1.47541724926e-06	0.00061302576147	0.211,0.0	1.0,1.0	-0.895

10-12m_vs_8-10w	SE	Prdm9	ENSMUSG00000051977.14	chr17	-	15555086	15555358	15554687	15554755	15555565	15555667	3909	24,22	0,0	26,18	3,4	298	149	4.86451062081e-05	0.00644984676891	1.0,1.0	0.813,0.692	0.248
10-12m_vs_8-10w	SE	Drc1	ENSMUSG00000073102.7	chr5	+	30348662	30348749	30347055	30347193	30350400	30350523	3960	17,17	3,5	49,21	1,1	235	149	0.000998385476585	0.0498059702143	0.782,0.683	0.969,0.93	-0.217
10-12m_vs_8-10w	SE	Slco4c1	ENSMUSG00000040693.7	chr1	-	96836879	96837075	96831985	96832139	96837453	96837598	4191	15,21	10,11	61,42	10,3	298	149	0.000157977681708	0.0148808107574	0.429,0.488	0.753,0.875	-0.356
10-12m_vs_8-10w	SE	Pitpnb	ENSMUSG00000050017.11	chr5	+	111338282	111338428	111330762	111330833	111349490	111349574	4198	4,1	2,2	7,4	1,0	294	149	0.000465303159843	0.0306920307144	0.503,0.202	0.78,1.0	-0.538
10-12m_vs_8-10w	SE	Rsph10b	ENSMUSG00000075569.10	chr5	+	143979857	143979948	143977046	143977166	143983903	143984102	4308	2,3	0,0	2,3	2,2	239	149	8.61302275658e-05	0.0095692891293	1.0,1.0	0.384,0.483	0.567
10-12m_vs_8-10w	SE	Tank	ENSMUSG00000064289.15	chr2	+	61593996	61594201	61593096	61593208	61613650	61613798	4366	59,92	99,137	69,85	62,54	298	149	0.000219498078889	0.0189136256556	0.23,0.251	0.358,0.44	-0.159
10-12m_vs_8-10w	SE	Git2	ENSMUSG00000041890.17	chr5	-	114737347	114737437	114733860	114733943	114739032	114739281	4376	0,1	5,16	2,6	11,4	238	149	0.000562994471843	0.0343585217816	0.0,0.038	0.102,0.484	-0.274
10-12m_vs_8-10w	SE	Dhx40	ENSMUSG00000018425.18	chr11	-	86789166	86789349	86785756	86785837	86789485	86789572	4584	73,118	0,1	101,97	4,7	298	149	0.000330759137612	0.0245287418164	1.0,0.983	0.927,0.874	0.091
10-12m_vs_8-10w	SE	Lrch3	ENSMUSG00000022801.13	chr16	+	33009188	33009436	33005663	33005741	33012340	33012699	4651	3,3	0,0	3,3	2,5	298	149	1.63718464341e-08	1.93217406535e-05	1.0,1.0	0.429,0.231	0.67
10-12m_vs_8-10w	SE	Catsperg1	ENSMUSG00000049676.14	chr7	-	29190212	29190331	29189946	29190027	29190726	29190789	4665	13,7	1,4	20,13	0,0	267	149	0.000295895219587	0.0229378525288	0.879,0.494	1.0,1.0	-0.314
10-12m_vs_8-10w	SE	Catsperg1	ENSMUSG00000049676.14	chr7	-	29190212	29190635	29189946	29190027	29190726	29190789	4666	15,5	1,4	20,10	0,0	298	149	0.000258419059894	0.0210136957256	0.882,0.385	1.0,1.0	-0.367
10-12m_vs_8-10w	SE	Ilf3	ENSMUSG00000032178.14	chr9	+	21397968	21398136	21397468	21397668	21399124	21399250	4932	4,20	0,0	8,1	7,2	298	149	5.61674895483e-09	9.4645340305e-06	1.0,1.0	0.364,0.2	0.718
10-12m_vs_8-10w	SE	Tex30	ENSMUSG00000026049.11	chr1	-	44091360	44091591	44088475	44088527	44099847	44099921	4968	1027,1095	59,48	1091,953	102,77	298	149	0.000543666152119	0.0338602424228	0.897,0.919	0.842,0.861	0.057
10-12m_vs_8-10w	SE	Riok1	ENSMUSG00000021428.9	chr13	+	38045272	38045315	38043908	38043978	38047244	38047337	5046	27,57	4,4	33,28	0,0	191	149	0.000773250552367	0.0424881567099	0.84,0.917	1.0,1.0	-0.121
10-12m_vs_8-10w	SE	Riok1	ENSMUSG00000021428.9	chr13	+	38046850	38046907	38043908	38043978	38047244	38047337	5047	27,38	4,4	25,23	0,0	205	149	0.000359526248746	0.0258407361391	0.831,0.873	1.0,1.0	-0.148
10-12m_vs_8-10w	SE	Lrrc2	ENSMUSG00000032495.8	chr9	+	110966440	110966577	110962514	110962671	110970013	110970138	5083	32,15	8,0	27,12	0,0	285	149	0.000288625059567	0.0227384069655	0.677,1.0	1.0,1.0	-0.161
10-12m_vs_8-10w	SE	Wnk2	ENSMUSG00000037989.15	chr13	-	49044373	49044418	49042627	49042866	49051356	49051527	5197	0,1	7,2	5,5	4,5	193	149	0.000213401415659	0.0185996503976	0.0,0.279	0.491,0.436	-0.324
10-12m_vs_8-10w	SE	Fbxw9	ENSMUSG00000008167.14	chr8	+	85064383	85064496	85062171	85062300	85064580	85064672	5309	2,5	0,4	0,1	3,4	261	149	0.000399747817155	0.0277454257257	1.0,0.416	0.0,0.125	0.646
10-12m_vs_8-10w	SE	Dydc1	ENSMUSG00000021790.12	chr14	+	41086353	41086413	41082271	41082361	41087384	41087489	5375	1,1	3,2	10,5	0,1	208	149	5.11044458684e-06	0.0013531253808	0.193,0.264	1.0,0.782	-0.663
10-12m_vs_8-10w	SE	Eif4a2	ENSMUSG00000022884.14	chr16	+	23109984	23110153	23108587	23108851	23110233	23110318	5386	261,507	19,35	393,301	52,47	298	149	0.000365228402119	0.0261885169378	0.873,0.879	0.791,0.762	0.1
10-12m_vs_8-10w	SE	Ccdc60	ENSMUSG00000043913.14	chr5	-	116136329	116136452	116134027	116134159	116137031	116137094	5696	28,41	3,0	29,31	9,14	271	149	1.54003335412e-05	0.00290128892322	0.837,1.0	0.639,0.549	0.324

10-12m_vs_8-10w	SE	Ube2h	ENSMUSG00000039159.16	chr6	-	30242537	30242577	30221667	30221796	30260928	30261003	5758	11,10	1,0	15,19	7,6	188	149	0.000131394031332	0.0131095801458	0.897,1.0	0.629,0.715	0.277
10-12m_vs_8-10w	SE	Dnase1l1	ENSMUSG00000019088.13	chrX	-	74273777	74274026	74273216	74273691	74276478	74276556	5765	4,0	6,1	4,2	0,1	298	149	0.000378773670004	0.0269053493791	0.25,0.0	1.0,0.5	-0.625
10-12m_vs_8-10w	SE	Ipo4	ENSMUSG00000002319.16	chr14	-	55628591	55628684	55628105	55628231	55628793	55628987	5991	0,1	1,1	6,3	0,0	241	149	0.000527635797871	0.0330655400521	0.0,0.382	1.0,1.0	-0.809
10-12m_vs_8-10w	SE	Calm1	ENSMUSG00000001175.14	chr12	+	100203574	100203718	100199434	100199716	100206097	100206233	6052	2,2	0,0	4,4	1,3	292	149	0.000245931703919	0.020565823823	1.0,1.0	0.671,0.405	0.462
10-12m_vs_8-10w	SE	Bola1	ENSMUSG00000015943.11	chr3	-	96198164	96198226	96196587	96197279	96198436	96198489	6090	1,0	2,2	2,1	1,0	210	149	9.62273401727e-05	0.0104238266242	0.262,0.0	0.587,1.0	-0.663
10-12m_vs_8-10w	SE	Zfp810	ENSMUSG00000066829.7	chr9	-	22298416	22298509	22290538	22290642	22305511	22305602	6208	32,28	3,10	45,23	0,0	241	149	2.55967370377e-06	0.000866201167484	0.868,0.634	1.0,1.0	-0.249
10-12m_vs_8-10w	SE	Amhr2	ENSMUSG00000023047.11	chr15	+	102452148	102452263	102446999	102447112	102453031	102453168	6250	1,8	0,8	1,4	0,0	263	149	0.000483112955249	0.0315124710659	1.0,0.362	1.0,1.0	-0.319
10-12m_vs_8-10w	SE	Cdk10	ENSMUSG00000033862.7	chr8	+	123227659	123227741	123226945	123227017	123228328	123228396	6428	10,9	6,3	7,5	0,1	230	149	0.000798050863079	0.0434572364956	0.519,0.66	1.0,0.764	-0.293
10-12m_vs_8-10w	SE	Cdk10	ENSMUSG00000033862.7	chr8	+	123230267	123230327	123229111	123229181	123230593	123230717	6430	3,5	2,3	7,1	0,0	208	149	6.72799958005e-05	0.00806588755978	0.518,0.544	1.0,1.0	-0.469
10-12m_vs_8-10w	SE	Cd200r1	ENSMUSG00000022667.17	chr16	+	44792488	44792630	44789972	44790224	44792726	44792826	6447	2,1	1,0	1,0	2,6	290	149	0.000398464901775	0.0277454257257	0.507,1.0	0.204,0.0	0.652
10-12m_vs_8-10w	SE	Ptpn9	ENSMUSG00000032290.7	chr9	+	57056713	57056792	57048190	57048257	57059853	57060061	6484	1,2	0,0	3,3	4,4	227	149	1.62010995119e-06	0.000629994293969	1.0,1.0	0.33,0.33	0.67
10-12m_vs_8-10w	SE	RP23-331A21.2	ENSMUSG00000035401.9	chr7	-	98600671	98600827	98597032	98597184	98610666	98610865	6546	4,15	0,2	7,2	4,5	298	149	0.000617593613633	0.0362325568571	1.0,0.789	0.467,0.167	0.578
10-12m_vs_8-10w	SE	Zfp708	ENSMUSG00000058883.16	chr13	-	67074832	67074916	67069398	67071528	67097792	67097976	6564	6,10	5,7	5,5	0,1	232	149	0.000222429286395	0.0191119056251	0.435,0.478	1.0,0.763	-0.425
10-12m_vs_8-10w	SE	Zfp708	ENSMUSG00000058883.16	chr13	-	67074832	67074959	67069398	67071528	67097792	67097976	6565	11,41	5,7	10,20	0,1	275	149	0.000248914244137	0.0206844327094	0.544,0.76	1.0,0.916	-0.306
10-12m_vs_8-10w	SE	RP23-477C12.1	ENSMUSG00000113930.1	chr12	-	16014647	16014748	16007136	16007242	16015402	16015545	6571	1,5	3,2	7,2	1,0	249	149	0.000568348617816	0.0344806282389	0.166,0.599	0.807,1.0	-0.521
10-12m_vs_8-10w	SE	Zfp346	ENSMUSG00000021481.11	chr13	+	55115482	55115624	55113677	55113770	55122329	55122515	6617	0,2	2,1	1,3	0,1	290	149	0.000555174547395	0.0342953140469	0.0,0.507	1.0,0.607	-0.55
10-12m_vs_8-10w	SE	Troap	ENSMUSG00000032783.9	chr15	+	99082908	99083102	99082125	99082705	99083227	99083403	6645	18,27	4,2	25,15	0,0	298	149	0.000147667020512	0.0141736974657	0.692,0.871	1.0,1.0	-0.219
10-12m_vs_8-10w	SE	Nipa2	ENSMUSG00000030452.16	chr7	-	55937571	55937662	55931286	55933547	55962199	55962392	6677	5,6	2,2	11,1	0,0	239	149	0.000586822944384	0.0353153308058	0.609,0.652	1.0,1.0	-0.369
10-12m_vs_8-10w	SE	Nav2	ENSMUSG00000052512.17	chr7	+	49552839	49552905	49551596	49552049	49556909	49557070	6707	0,0	10,9	3,3	6,1	214	149	2.40158526287e-05	0.00411539449763	0.0,0.0	0.258,0.676	-0.467
10-12m_vs_8-10w	SE	Spdye4b	ENSMUSG00000029586.9	chr5	+	143202017	143202186	143195671	143195730	143202347	143202450	6759	17,52	0,0	49,30	0,9	298	149	0.000399516319715	0.0277454257257	1.0,1.0	1.0,0.625	0.188
10-12m_vs_8-10w	SE	Tram2	ENSMUSG00000041779.5	chr1	-	21006158	21006234	21005566	21005637	21006852	21006911	7119	10,25	5,2	15,19	0,0	224	149	0.000109742929208	0.0115176913004	0.571,0.893	1.0,1.0	-0.268

10-12m_vs_8-10w	SE	AC159266.3	ENSMUSG00000008129.14	chr8	+	84166580	84167690	84161212	84161291	84170354	84170452	7138	3,10	0,1	3,6	2,3	298	149	0.000504878541587	0.0323221713132	1.0,0.833	0.429,0.5	0.452
10-12m_vs_8-10w	SE	Acsbg2	ENSMUSG00000024207.8	chr17	-	56845403	56845513	56843102	56843377	56853736	56853913	7274	3,1	0,0	2,0	3,2	258	149	6.55378595837e-09	1.04622569423e-05	1.0,1.0	0.278,0.0	0.861
10-12m_vs_8-10w	SE	Lman2l	ENSMUSG00000001143.13	chr1	-	36438749	36438832	36428299	36428461	36439608	36439668	7426	4,15	3,4	13,1	0,0	231	149	1.11924605346e-05	0.00237397566766	0.462,0.708	1.0,1.0	-0.415
10-12m_vs_8-10w	SE	Lman2l	ENSMUSG00000001143.13	chr1	-	36444036	36444142	36443519	36443609	36445049	36445271	7427	2,0	4,1	1,1	0,0	254	149	1.08024230672e-08	1.56022997167e-05	0.227,0.0	1.0,1.0	-0.887
10-12m_vs_8-10w	SE	Cd72	ENSMUSG00000028459.11	chr4	-	43448259	43448394	43447723	43448036	43449451	43449528	7643	6,3	0,0	4,5	3,1	283	149	0.000428821466356	0.0289678928642	1.0,1.0	0.412,0.725	0.432
10-12m_vs_8-10w	SE	Atp13a3	ENSMUSG00000022533.14	chr16	-	30332198	30332386	30328955	30329044	30336189	30336262	7774	186,211	0,0	339,288	11,8	298	149	4.64939159863e-05	0.00621236548802	1.0,1.0	0.939,0.947	0.057
10-12m_vs_8-10w	SE	Fam178b	ENSMUSG00000046337.17	chr1	-	36564830	36564908	36562691	36563020	36578518	36578644	7903	10,7	1,2	3,2	9,3	226	149	8.31814270248e-05	0.00934435504848	0.868,0.698	0.18,0.305	0.541
10-12m_vs_8-10w	SE	Tango2	ENSMUSG00000013539.13	chr16	-	18302685	18302790	18300824	18301552	18310868	18310983	7925	15,34	2,6	35,31	0,0	253	149	3.36956445539e-05	0.00524114151264	0.815,0.769	1.0,1.0	-0.208
10-12m_vs_8-10w	SE	Tango2	ENSMUSG00000013539.13	chr16	-	18307969	18308040	18300824	18301552	18310868	18310983	7930	5,12	2,6	17,9	0,0	219	149	1.91085107537e-06	0.00069111201667	0.63,0.576	1.0,1.0	-0.397
10-12m_vs_8-10w	SE	Trip4	ENSMUSG00000032386.15	chr9	-	65874830	65875043	65873098	65873177	65879074	65879208	7955	7,16	1,0	14,3	8,9	298	149	2.10163647374e-06	0.000741217859128	0.778,1.0	0.467,0.143	0.584
10-12m_vs_8-10w	SE	Myef2	ENSMUSG00000027201.16	chr2	-	125097976	125098075	125095707	125095759	125098449	125098518	7992	10,10	0,0	4,11	1,3	247	149	0.000968498128058	0.0488777316508	1.0,1.0	0.707,0.689	0.302
10-12m_vs_8-10w	SE	Myef2	ENSMUSG00000027201.16	chr2	-	125098796	125098847	125098449	125098518	125100318	125100420	7997	1,4	20,20	19,13	6,13	199	149	1.56566327603e-06	0.00061672899773	0.036,0.13	0.703,0.428	-0.483
10-12m_vs_8-10w	SE	Cacul1	ENSMUSG00000033417.15	chr19	-	60537413	60537503	60534135	60534274	60543009	60543112	8181	12,11	0,0	18,8	6,1	238	149	3.69931700409e-05	0.00545517696261	1.0,1.0	0.653,0.834	0.256
10-12m_vs_8-10w	SE	RP23-359G8.3	ENSMUSG00000116016.1	chr13	-	111453023	111453247	111448932	111448999	111453372	111453496	8202	1805,2811	111,98	2752,2688	412,161	298	149	2.75060535253e-05	0.00450965464582	0.89,0.935	0.77,0.893	0.081
10-12m_vs_8-10w	SE	Qtrt1	ENSMUSG00000002825.7	chr9	+	21417271	21417387	21417064	21417143	21418283	21418314	8238	18,26	5,1	63,35	0,0	264	149	0.000251244132088	0.0207642664043	0.67,0.936	1.0,1.0	-0.197
10-12m_vs_8-10w	SE	Qtrt1	ENSMUSG00000002825.7	chr9	+	21417271	21417397	21417064	21417143	21418283	21418422	8239	15,15	5,1	41,21	0,0	274	149	5.82680282318e-05	0.00725086066935	0.62,0.891	1.0,1.0	-0.244
10-12m_vs_8-10w	SE	Qtrt1	ENSMUSG00000002825.7	chr9	+	21419438	21419548	21419290	21419366	21419624	21419712	8241	37,20	0,0	66,36	7,4	258	149	6.80204271634e-05	0.00807440017334	1.0,1.0	0.845,0.839	0.158
10-12m_vs_8-10w	SE	Mbtd1	ENSMUSG00000059474.13	chr11	+	93887021	93887073	93885851	93886062	93921366	93921484	8511	5,3	1,4	4,2	0,0	200	149	7.12272548259e-05	0.00837361963614	0.788,0.358	1.0,1.0	-0.427
10-12m_vs_8-10w	SE	Med24	ENSMUSG00000017210.14	chr11	-	98707117	98707299	98706825	98706916	98707536	98707801	8581	10,21	3,2	21,10	0,0	298	149	0.000504787728934	0.0323221713132	0.625,0.84	1.0,1.0	-0.268
10-12m_vs_8-10w	SE	Pex2	ENSMUSG00000040374.13	chr3	-	5570486	5570629	5563619	5563729	5576037	5576128	8621	0,0	1,1	1,1	0,0	291	149	1.79966217484e-08	1.9494840509e-05	0.0,0.0	1.0,1.0	-1.0
10-12m_vs_8-10w	SE	Pdk1	ENSMUSG00000006494.11	chr2	+	71895688	71895799	71888895	71888994	71899983	71901986	8653	1,8	0,1	5,2	3,6	259	149	0.000121273729433	0.0123021855767	1.0,0.822	0.489,0.161	0.586

10-12m_vs_8-10w	SE	Pdk1	ENSMUSG00000006494.11	chr2	+	71897646	71897760	71888895	71888994	71899983	71901986	8654	2,8	0,1	3,0	3,6	262	149	3.83499339729e-06	0.00110780175936	1.0,0.82	0.363,0.0	0.728
10-12m_vs_8-10w	SE	Bcs1l	ENSMUSG00000026172.12	chr1	+	74590264	74590459	74589959	74590099	74590670	74590734	8671	2,13	0,1	11,10	6,3	298	149	0.000403825346614	0.0279007439366	1.0,0.867	0.478,0.625	0.382
10-12m_vs_8-10w	SE	Cep85	ENSMUSG00000037443.13	chr4	-	134164627	134164757	134156096	134156371	134172812	134172880	8686	1,0	1,1	2,1	0,0	278	149	0.000337016429901	0.0247507150976	0.349,0.0	1.0,1.0	-0.826
10-12m_vs_8-10w	SE	Tmem14c	ENSMUSG00000021361.7	chr13	+	41017114	41017173	41016291	41016398	41017705	41017782	8733	353,693	9,13	386,303	29,16	207	149	3.484323447e-05	0.00531070424477	0.966,0.975	0.905,0.932	0.052
10-12m_vs_8-10w	SE	Max	ENSMUSG00000059436.13	chr12	-	76939429	76939514	76938665	76938755	76939966	76939994	8768	23,42	19,49	12,9	32,32	233	149	0.000428029018061	0.0289678928642	0.436,0.354	0.193,0.152	0.223
10-12m_vs_8-10w	SE	RP23-313A10.2	ENSMUSG00000097483.2	chr13	-	66505097	66505203	66503159	66503970	66519346	66519523	8867	0,8	2,2	9,6	1,0	254	149	0.000583178936644	0.0352358572258	0.0,0.701	0.841,1.0	-0.57
10-12m_vs_8-10w	SE	Rev1	ENSMUSG00000026082.11	chr1	-	38107567	38107694	38098852	38099021	38108403	38108467	8967	48,64	0,0	63,35	6,1	275	149	0.000744179411866	0.0416452135448	1.0,1.0	0.851,0.95	0.1
10-12m_vs_8-10w	SE	Rev1	ENSMUSG00000026082.11	chr1	-	38108403	38108467	38098852	38099021	38128251	38128315	8968	1,1	2,1	6,2	0,0	212	149	0.000560739921574	0.0343585217816	0.26,0.413	1.0,1.0	-0.664
10-12m_vs_8-10w	SE	RP23-414K1.4	ENSMUSG00000033029.12	chr15	-	79139237	79139300	79135636	79135765	79140880	79140978	9109	3,6	0,0	19,5	3,2	211	149	0.000869983334355	0.0458175340413	1.0,1.0	0.817,0.638	0.272
10-12m_vs_8-10w	SE	Zbtb18	ENSMUSG00000063659.11	chr1	+	177444738	177444822	177443984	177444092	177447088	177447096	9217	3,2	0,2	1,0	1,2	232	149	0.000670824919062	0.038682111445	1.0,0.391	0.391,0.0	0.5
10-12m_vs_8-10w	SE	Myrfl	ENSMUSG00000034057.8	chr10	-	116776948	116777023	116776534	116776814	116777343	116777455	9286	1,2	3,1	4,1	0,0	223	149	3.1664931525e-05	0.00497631625951	0.182,0.572	1.0,1.0	-0.623
10-12m_vs_8-10w	SE	Cd63	ENSMUSG00000025351.14	chr10	+	128912437	128912521	128912160	128912301	128912674	128912815	9427	482,665	14,8	858,505	2,3	232	149	0.000918622258182	0.0476142409778	0.957,0.982	0.996,0.991	-0.024
10-12m_vs_8-10w	SE	Mcrs1	ENSMUSG00000037570.16	chr15	-	99249367	99249506	99248701	99248860	99251861	99251917	9559	8,58	4,6	13,18	0,0	287	149	4.89744624306e-05	0.00644984676891	0.509,0.834	1.0,1.0	-0.329
10-12m_vs_8-10w	SE	Ube2f	ENSMUSG00000034343.14	chr1	+	91253759	91253827	91250310	91250437	91254262	91254393	9565	2,2	1,1	1,0	2,3	216	149	0.00075948930117	0.042241544786	0.58,0.58	0.256,0.0	0.452
10-12m_vs_8-10w	SE	Pacrgl	ENSMUSG00000029089.8	chr5	+	48380123	48380231	48379329	48379464	48381786	48381867	9616	262,445	5,4	277,255	21,10	256	149	1.31743686698e-05	0.00269994443327	0.968,0.985	0.885,0.937	0.065
10-12m_vs_8-10w	SE	Isyna1	ENSMUSG00000019139.10	chr8	+	70595138	70595271	70594822	70594984	70595704	70595854	9636	1,4	6,1	8,1	1,0	281	149	0.000333223400745	0.0246463100869	0.081,0.68	0.809,1.0	-0.524
10-12m_vs_8-10w	SE	Isyna1	ENSMUSG00000019139.10	chr8	+	70595138	70595554	70594822	70594984	70595704	70595854	9637	2,9	6,1	21,5	1,0	298	149	0.000299954189965	0.023091143492	0.143,0.818	0.913,1.0	-0.476
10-12m_vs_8-10w	SE	Phf20l1	ENSMUSG00000072501.5	chr15	+	66597676	66597754	66597358	66597447	66604026	66604240	9912	7,12	7,13	19,9	1,3	226	149	0.000568405727455	0.0344806282389	0.397,0.378	0.926,0.664	-0.408
10-12m_vs_8-10w	SE	Fam172a	ENSMUSG00000064138.14	chr13	+	77761837	77761938	77759427	77759529	77825322	77825388	10007	12,20	1,5	12,23	0,0	249	149	0.000100303940822	0.0107502432123	0.878,0.705	1.0,1.0	-0.209
10-12m_vs_8-10w	SE	Fam118b	ENSMUSG00000050471.17	chr9	-	35227163	35227391	35223613	35223742	35235230	35235483	10112	147,217	14,21	155,159	47,30	298	149	0.000171371459703	0.0158471577569	0.84,0.838	0.622,0.726	0.165
10-12m_vs_8-10w	SE	Tulp2	ENSMUSG00000023467.18	chr7	+	45518585	45518885	45517779	45517967	45519730	45519843	10154	0,0	1,1	4,3	3,0	298	149	1.47879599545e-05	0.00287484005634	0.0,0.0	0.4,1.0	-0.7

10-12m_vs_8-10w	SE	Tulp2	ENSMUSG00000023467.18	chr7	+	45518585	45518906	45517779	45517967	45519730	45519843	10155	0,0	1,1	7,2	3,0	298	149	2.80541236641e-06	0.000886033452992	0.0,0.0	0.538,1.0	-0.769
10-12m_vs_8-10w	SE	RP23-294B15.2	ENSMUSG00000092152.1	chr14	-	42256546	42256730	42254658	42255402	42257383	42257515	10242	12,22	1,1	17,17	12,5	298	149	0.000296751176132	0.0229378525288	0.857,0.917	0.415,0.63	0.365
10-12m_vs_8-10w	SE	Psmd8	ENSMUSG00000030591.17	chr7	-	29178918	29179021	29176840	29177231	29180276	29180481	10283	23,57	0,1	48,25	5,4	251	149	0.000307705607548	0.0235088634321	1.0,0.971	0.851,0.788	0.166
10-12m_vs_8-10w	SE	Sycp2l	ENSMUSG00000038651.15	chr13	+	41157433	41157517	41155518	41155645	41163071	41163145	10344	1,12	4,13	1,0	4,13	232	149	0.00015154193228	0.0144541457484	0.138,0.372	0.138,0.0	0.186
10-12m_vs_8-10w	SE	Fam173b	ENSMUSG00000039065.11	chr15	+	31603448	31603574	31602117	31602149	31616870	31618609	10355	0,0	1,5	4,2	0,5	274	149	0.000269407623513	0.0216290635501	0.0,0.0	1.0,0.179	-0.59
10-12m_vs_8-10w	SE	Tmem120a	ENSMUSG00000039886.8	chr5	-	135736745	135736835	135736596	135736662	135736917	135737013	10402	84,183	0,14	287,139	2,0	238	149	7.95792037295e-05	0.00907412341473	1.0,0.891	0.989,1.0	-0.049
10-12m_vs_8-10w	SE	Tex9	ENSMUSG00000090626.9	chr9	-	72479923	72480001	72478339	72478422	72480596	72480775	10408	0,0	7,4	7,0	17,4	226	149	0.000763813903202	0.042241544786	0.0,0.0	0.214,0.0	-0.107
10-12m_vs_8-10w	SE	Tex9	ENSMUSG00000090626.9	chr9	-	72482446	72482523	72480596	72480775	72484068	72484114	10409	2,7	0,1	5,1	4,2	225	149	0.000270802396845	0.0216720514478	1.0,0.823	0.453,0.249	0.561
10-12m_vs_8-10w	SE	Ubac2	ENSMUSG00000041765.6	chr14	+	121973608	121973732	121908210	121908320	121994224	121994308	10415	8,30	1,8	26,15	1,0	272	149	0.000161396316289	0.0150624974442	0.814,0.673	0.934,1.0	-0.224
10-12m_vs_8-10w	SE	Ubac2	ENSMUSG00000041765.6	chr14	+	121983267	121983315	121908210	121908320	121994224	121994308	10416	2,6	1,8	7,3	1,0	196	149	0.000138552157121	0.0136886823376	0.603,0.363	0.842,1.0	-0.438
10-12m_vs_8-10w	SE	Slc30a2	ENSMUSG00000028836.14	chr4	+	134347339	134347493	134345902	134346049	134348476	134348645	10502	8,7	0,0	11,13	3,3	298	149	9.79923904543e-05	0.0105772498038	1.0,1.0	0.647,0.684	0.335
10-12m_vs_8-10w	SE	Mnat1	ENSMUSG00000021103.12	chr12	+	73219020	73219142	73170593	73170667	73272413	73272851	10544	16,39	0,0	60,20	2,6	270	149	0.000258358823049	0.0210136957256	1.0,1.0	0.943,0.648	0.205
10-12m_vs_8-10w	SE	Adam5	ENSMUSG00000031554.18	chr8	-	24742097	24742151	24732984	24733157	24746656	24746771	10736	2,2	0,0	10,5	4,4	202	149	5.72237161822e-06	0.00144637711294	1.0,1.0	0.648,0.48	0.436
10-12m_vs_8-10w	SE	Nudt22	ENSMUSG00000037349.9	chr19	-	6993661	6993759	6993432	6993526	6995241	6995735	10762	35,40	2,8	51,32	1,0	246	149	0.000177377429384	0.0163018008011	0.914,0.752	0.969,1.0	-0.151
10-12m_vs_8-10w	SE	Nudt22	ENSMUSG00000037349.9	chr19	-	6994670	6994769	6993432	6993526	6995241	6995735	10763	64,78	2,8	75,39	1,0	247	149	0.000849846076123	0.0454615191091	0.951,0.855	0.978,1.0	-0.086
10-12m_vs_8-10w	SE	Ube2d3	ENSMUSG00000078578.9	chr3	+	135458797	135458875	135439136	135439798	135465194	135465237	10840	15,20	5,1	11,21	0,0	226	149	0.00022380966137	0.0191221713775	0.664,0.93	1.0,1.0	-0.203
10-12m_vs_8-10w	SE	Ube2d3	ENSMUSG00000078578.9	chr3	+	135459974	135460080	135439136	135439798	135465194	135465237	10846	0,1	5,1	2,1	0,0	254	149	5.28270760469e-11	4.00574510895e-07	0.0,0.37	1.0,1.0	-0.815
10-12m_vs_8-10w	SE	Idh2	ENSMUSG00000030541.16	chr7	-	80098135	80098272	80097803	80097955	80098770	80098914	10869	4,4	0,0	5,0	5,1	285	149	1.44983845374e-07	8.97450002865e-05	1.0,1.0	0.343,0.0	0.829
10-12m_vs_8-10w	SE	Idh2	ENSMUSG00000030541.16	chr7	-	80098135	80098381	80097803	80097955	80098770	80098914	10870	4,4	0,0	4,0	5,1	298	149	2.3278930561e-08	2.27765562208e-05	1.0,1.0	0.286,0.0	0.857
10-12m_vs_8-10w	SE	Fbxo47	ENSMUSG00000070336.3	chr11	-	97877956	97878127	97872857	97872934	97879383	97879588	11158	2,11	0,1	0,3	3,2	298	149	1.64276599093e-05	0.00301980213763	1.0,0.846	0.0,0.429	0.709
10-12m_vs_8-10w	SE	Chac2	ENSMUSG00000020309.6	chr11	-	30979564	30979600	30976706	30977746	30986111	30986350	11391	3,5	0,1	1,1	4,1	184	149	2.06327016368e-05	0.00359661191578	1.0,0.802	0.168,0.447	0.594

10-12m_vs_8-10w	SE	Gemin8	ENSMUSG00000040621.14	chrX	+	166178533	166178642	166170494	166170550	166178791	166178830	11421	1,1	0,0	0,0	1,1	257	149	1.93188353093e-08	2.02055032333e-05	1.0,1.0	0.0,0.0	1.0
10-12m_vs_8-10w	SE	Epb41l4a	ENSMUSG00000024376.6	chr18	-	33810218	33810338	33802512	33802560	33824458	33824536	11594	44,95	3,8	57,45	0,0	268	149	6.57539291914e-05	0.00797752970522	0.891,0.868	1.0,1.0	-0.121
10-12m_vs_8-10w	SE	Tchp	ENSMUSG00000002486.15	chr5	+	114709306	114709517	114708653	114708838	114711252	114711309	11659	50,27	4,5	56,46	0,0	298	149	1.78768805239e-05	0.00324684828246	0.862,0.73	1.0,1.0	-0.204
10-12m_vs_8-10w	SE	Sfta2	ENSMUSG00000090509.2	chr17	+	35616594	35616719	35601577	35601781	35627993	35628163	11662	42,30	20,17	9,11	20,17	273	149	0.000139497778572	0.0137373607853	0.534,0.491	0.197,0.261	0.284
10-12m_vs_8-10w	SE	RP23-359F5.1	ENSMUSG00000044551.13	chr14	-	70156160	70156276	70155849	70155953	70156415	70156518	11736	0,4	2,1	9,7	0,0	264	149	0.00100338656708	0.049932663762	0.0,0.693	1.0,1.0	-0.654
10-12m_vs_8-10w	SE	Aak1	ENSMUSG00000057230.13	chr6	+	86959031	86959274	86956258	86956572	86963992	86964225	11815	0,4	2,7	2,2	0,0	298	149	5.76644287875e-10	1.74901978955e-06	0.0,0.222	1.0,1.0	-0.889
10-12m_vs_8-10w	SE	Ecd	ENSMUSG00000021810.3	chr14	-	20324372	20324581	20319851	20320951	20327223	20327291	11869	209,181	1,1	303,196	9,6	298	149	0.0010078289796	0.049932663762	0.991,0.989	0.944,0.942	0.047
10-12m_vs_8-10w	SE	Fnip1	ENSMUSG00000035992.15	chr11	+	54487711	54487795	54482494	54482586	54489268	54489340	11899	18,19	0,0	18,22	3,3	232	149	0.000670033610391	0.038682111445	1.0,1.0	0.794,0.825	0.191
10-12m_vs_8-10w	SE	Prkcz	ENSMUSG00000029053.16	chr4	-	155356426	155356516	155354653	155354704	155357480	155357602	12046	71,88	0,0	66,57	8,1	238	149	0.000177485130895	0.0163018008011	1.0,1.0	0.838,0.973	0.095
10-12m_vs_8-10w	SE	Actl6a	ENSMUSG00000027671.14	chr3	+	32712100	32712275	32711867	32711944	32714516	32714617	12048	107,151	8,8	170,146	1,3	298	149	0.00060365059834	0.0358185431004	0.87,0.904	0.988,0.961	-0.087
10-12m_vs_8-10w	SE	Zfp932	ENSMUSG00000066613.14	chr5	+	110006943	110007070	109996520	109996633	110008631	110009103	12106	11,8	1,0	5,7	1,4	275	149	0.000970368578039	0.0488907796354	0.856,1.0	0.73,0.487	0.319
10-12m_vs_8-10w	SE	Sorbs2	ENSMUSG00000031626.17	chr8	+	45782801	45782960	45775588	45775703	45789975	45790025	12294	1,12	5,14	0,0	8,5	298	149	3.76298283102e-05	0.00545517696261	0.091,0.3	0.0,0.0	0.196
10-12m_vs_8-10w	SE	RP24-458J4.8	ENSMUSG00000112188.1	chr10	+	95734814	95734928	95731226	95731502	95739073	95739353	12382	17,5	4,2	8,3	0,0	262	149	3.93151247235e-05	0.00557227592518	0.707,0.587	1.0,1.0	-0.353
10-12m_vs_8-10w	SE	Dnah2	ENSMUSG00000005237.14	chr11	-	69421269	69421420	69420808	69421147	69421561	69421636	12440	4,3	2,2	14,3	0,0	298	149	6.72637087901e-05	0.00806588755978	0.5,0.429	1.0,1.0	-0.536
10-12m_vs_8-10w	SE	RP24-353D23.1	ENSMUSG00000026319.13	chr1	+	105741012	105741095	105740434	105740517	105741296	105741360	12453	0,0	9,4	6,1	5,5	231	149	0.000105699863762	0.0112886710133	0.0,0.0	0.436,0.114	-0.275
10-12m_vs_8-10w	SE	Ggps1	ENSMUSG00000021302.10	chr13	-	14058923	14059211	14057795	14057888	14059318	14059462	12496	71,90	16,18	64,53	43,25	298	149	0.000131333682648	0.0131095801458	0.689,0.714	0.427,0.515	0.231
10-12m_vs_8-10w	SE	Gpn3	ENSMUSG00000029464.10	chr5	+	122381397	122381494	122381189	122381305	122382047	122382176	12529	185,313	5,11	455,339	3,2	245	149	0.000484644910941	0.0315445596432	0.957,0.945	0.989,0.99	-0.039
10-12m_vs_8-10w	SE	Sft2d1	ENSMUSG00000073468.11	chr17	+	8323276	8323335	8320547	8320681	8326954	8327008	12556	1,1	1,3	2,2	0,0	207	149	3.63022403815e-06	0.00105873389713	0.419,0.194	1.0,1.0	-0.694
10-12m_vs_8-10w	SE	Fam169b	ENSMUSG00000074071.11	chr7	+	68329221	68329399	68304551	68304651	68353608	68353743	12651	0,0	1,2	1,2	0,1	298	149	0.000430575476417	0.0290217439449	0.0,0.0	1.0,0.5	-0.75
10-12m_vs_8-10w	SE	Nlrp2	ENSMUSG00000035177.8	chr7	-	5327407	5329010	5324893	5325064	5332959	5333019	12735	17,21	0,0	26,21	6,0	298	149	0.000732728562879	0.0412326345838	1.0,1.0	0.684,1.0	0.158
10-12m_vs_8-10w	SE	Arhgap10	ENSMUSG00000037148.8	chr8	-	77365085	77365160	77358546	77358634	77382724	77382790	12872	10,29	0,0	15,5	3,4	223	149	8.83525173589e-06	0.00194189869856	1.0,1.0	0.77,0.455	0.387

10-12m_vs_8-10w	SE	Unkl	ENSMUSG00000015127.14	chr17	+	25218577	25218755	25213046	25213106	25228582	25228666	12901	1,10	2,2	1,0	3,1	298	149	0.00100909794407	0.049932663762	0.2,0.714	0.143,0.0	0.385
10-12m_vs_8-10w	SE	Ist1	ENSMUSG00000031729.6	chr8	-	109675642	109675802	109675375	109675424	109676757	109676850	12969	26,68	10,17	44,17	43,17	298	149	6.63896313075e-05	0.00802256536728	0.565,0.667	0.338,0.333	0.281
10-12m_vs_8-10w	SE	Ist1	ENSMUSG00000031729.6	chr8	-	109677485	109677686	109675375	109676850	109678854	109678965	12978	69,146	0,1	179,79	11,4	298	149	4.91166956241e-05	0.00644984676891	1.0,0.986	0.891,0.908	0.094
10-12m_vs_8-10w	SE	Ist1	ENSMUSG00000031729.6	chr8	-	109682597	109682778	109682106	109682194	109683675	109683778	12979	69,111	0,0	154,73	6,5	298	149	0.000213202410528	0.0185996503976	1.0,1.0	0.928,0.88	0.096
10-12m_vs_8-10w	SE	Gon4l	ENSMUSG00000054199.17	chr3	+	88858386	88858506	88857127	88857226	88858888	88859059	12987	0,1	3,1	6,7	0,3	268	149	6.83459883674e-05	0.00807440017334	0.0,0.357	1.0,0.565	-0.604
10-12m_vs_8-10w	SE	ZNF654	ENSMUSG00000047141.5	chr16	-	64788304	64788446	64784506	64786802	64791086	64791226	13612	15,41	0,0	41,31	4,3	290	149	0.0005180612103	0.0328044145503	1.0,1.0	0.84,0.842	0.159
10-12m_vs_8-10w	SE	Sar1b	ENSMUSG00000020386.5	chr11	+	51788149	51788253	51782771	51782837	51789196	51789328	13647	212,236	8,1	243,201	15,14	252	149	0.000714747606254	0.0404459135173	0.94,0.993	0.905,0.895	0.066
10-12m_vs_8-10w	SE	Ptges2	ENSMUSG00000026820.5	chr2	+	32401486	32401604	32400049	32400108	32402175	32405772	13653	6,10	4,1	6,8	0,0	266	149	0.000159841269612	0.014963412187	0.457,0.849	1.0,1.0	-0.347
10-12m_vs_8-10w	SE	Eif5	ENSMUSG00000021282.17	chr12	+	111542679	111542841	111542151	111542310	111543099	111543264	13807	1178,1665	31,41	1560,1700	98,113	298	149	1.39181012271e-08	1.83543447095e-05	0.95,0.953	0.888,0.883	0.066
10-12m_vs_8-10w	SE	Eif5	ENSMUSG00000021282.17	chr12	+	111542779	111542841	111542224	111542310	111543099	111543264	13809	296,364	31,41	384,393	98,113	210	149	1.01741947645e-07	7.06675203677e-05	0.871,0.863	0.735,0.712	0.143
10-12m_vs_8-10w	SE	Bcl7c	ENSMUSG00000030814.17	chr7	-	127707175	127707304	127705726	127705812	127707879	127707958	13884	151,299	8,14	212,145	3,1	277	149	0.000503461894319	0.0323221713132	0.91,0.92	0.974,0.987	-0.065
10-12m_vs_8-10w	SE	Bcl7c	ENSMUSG00000030814.17	chr7	-	127707175	127707337	127705726	127705812	127707879	127707958	13885	163,338	8,14	233,155	3,1	298	149	0.00059294667509	0.035414553526	0.911,0.923	0.975,0.987	-0.064
10-12m_vs_8-10w	SE	Mri1	ENSMUSG00000004996.9	chr8	-	84253862	84254087	84249905	84251711	84254175	84254352	13889	8,6	3,5	32,17	1,0	298	149	1.73533687631e-06	0.000657931284942	0.571,0.375	0.941,1.0	-0.497
10-12m_vs_8-10w	SE	RP24-483G17.1	ENSMUSG00000033632.15	chr18	+	25339713	25340384	25289938	25290066	25344863	25344962	14044	24,34	3,9	21,23	0,1	298	149	0.000107240417936	0.0113439078197	0.8,0.654	1.0,0.92	-0.233
10-12m_vs_8-10w	SE	RP24-483G17.1	ENSMUSG00000033632.15	chr18	+	25344863	25344962	25339713	25340384	25399925	25400107	14047	11,31	0,1	13,14	2,5	247	149	0.000210809969616	0.0185335570679	1.0,0.949	0.797,0.628	0.262
10-12m_vs_8-10w	SE	Fam63a	ENSMUSG00000038712.16	chr3	+	95291479	95291544	95291021	95291079	95292124	95292283	14130	41,124	0,1	66,92	4,8	213	149	0.000141677225504	0.0139068347144	1.0,0.989	0.92,0.889	0.09
10-12m_vs_8-10w	SE	Lrrc46	ENSMUSG00000020878.6	chr11	-	97035458	97035604	97034601	97035024	97035871	97035941	14175	1,5	0,0	7,1	5,1	294	149	1.393940394e-06	0.000595487409724	1.0,1.0	0.415,0.336	0.625
10-12m_vs_8-10w	SE	Huwe1	ENSMUSG00000025261.17	chrX	+	151804445	151804540	151803260	151803569	151806275	151806407	14278	4,2	3,6	4,1	0,0	243	149	1.65627660476e-08	1.93217406535e-05	0.45,0.17	1.0,1.0	-0.69
10-12m_vs_8-10w	SE	Pigf	ENSMUSG00000024145.6	chr17	-	87008802	87008911	86997255	86997567	87020400	87020517	14335	1525,2600	31,30	1004,1221	40,36	257	149	4.18518146877e-05	0.00579638078216	0.966,0.98	0.936,0.952	0.029
10-12m_vs_8-10w	SE	Gpd1l	ENSMUSG00000050627.13	chr9	-	114904774	114905008	114903345	114903767	114910875	114910988	14338	105,235	12,25	351,233	9,14	298	149	0.000411691611536	0.0282511725554	0.814,0.825	0.951,0.893	-0.103
10-12m_vs_8-10w	SE	Tnfrsf12a	ENSMUSG00000023905.14	chr17	-	23676138	23676273	23675446	23676046	23677326	23677449	14348	58,124	5,9	126,81	2,1	283	149	0.000852634593961	0.0455303870941	0.859,0.879	0.971,0.977	-0.105

10-12m_vs_8-10w	SE	Tnfrsf12a	ENSMUSG00000023905.14	chr17	-	23676518	23676623	23675446	23676046	23677326	23677449	14349	11,23	5,9	45,18	2,1	253	149	5.95287753231e-05	0.007369662385	0.564,0.601	0.93,0.914	-0.34
10-12m_vs_8-10w	SE	Tnfrsf12a	ENSMUSG00000023905.14	chr17	-	23676518	23676623	23676138	23676273	23677326	23677449	14350	45,64	3,19	152,64	2,1	253	149	1.913995892e-06	0.00069111201667	0.898,0.665	0.978,0.974	-0.194
10-12m_vs_8-10w	SE	Ncapd3	ENSMUSG00000035024.16	chr9	+	27030622	27030830	27030174	27030499	27036984	27037139	14353	8,9	6,1	10,6	0,0	298	149	1.56511299882e-05	0.00293033594859	0.4,0.818	1.0,1.0	-0.391
10-12m_vs_8-10w	SE	Dmgdh	ENSMUSG00000042102.7	chr13	+	93711292	93711458	93709181	93709335	93712045	93712176	14479	5,7	0,0	1,2	2,1	298	149	0.000676992722137	0.0388898982105	1.0,1.0	0.2,0.5	0.65
10-12m_vs_8-10w	SE	RP24-414A22.6	ENSMUSG00000097919.1	chr8	-	120628471	120628517	120608785	120609030	120667756	120668079	14697	2,2	4,1	12,4	0,0	194	149	4.00187145599e-06	0.00113439965544	0.277,0.606	1.0,1.0	-0.559
10-12m_vs_8-10w	SE	Col4a3bp	ENSMUSG00000021669.15	chr13	+	96542854	96543259	96542617	96542644	96549196	96549331	14777	39,62	6,4	53,38	0,0	298	149	3.51853593286e-05	0.00532644640401	0.765,0.886	1.0,1.0	-0.175
10-12m_vs_8-10w	SE	Atp5j	ENSMUSG00000022890.13	chr16	-	84834808	84834983	84831328	84831507	84835420	84835503	14801	41,45	3,6	84,63	0,2	298	149	0.000684268628901	0.0392335572461	0.872,0.789	1.0,0.94	-0.139
10-12m_vs_8-10w	SE	Uchl5	ENSMUSG00000018189.12	chr1	+	143783965	143784029	143777271	143777476	143794276	143794402	14833	13,13	2,3	22,21	0,0	212	149	0.000791779140464	0.0432332843647	0.82,0.753	1.0,1.0	-0.214
10-12m_vs_8-10w	SE	Timm23	ENSMUSG00000013701.13	chr14	-	32191341	32191400	32189143	32189254	32198968	32199027	14888	549,817	7,8	714,540	32,20	207	149	2.35883704547e-06	0.000813021436661	0.983,0.987	0.941,0.951	0.039
10-12m_vs_8-10w	SE	Timm23	ENSMUSG00000013701.13	chr14	-	32193853	32193947	32189143	32189254	32198968	32199027	14904	699,1063	7,8	904,688	32,20	242	149	1.70261603205e-06	0.000653696795799	0.984,0.988	0.946,0.955	0.036
10-12m_vs_8-10w	SE	Fastkd2	ENSMUSG00000025962.15	chr1	+	63737778	63737918	63735462	63735571	63745841	63746005	15049	5,3	0,0	0,1	1,1	288	149	0.000301751298761	0.0231706801082	1.0,1.0	0.0,0.341	0.83
10-12m_vs_8-10w	SE	Oosp3	ENSMUSG00000055933.13	chr19	+	11699351	11699530	11697054	11697171	11700913	11701011	15159	2032,2894	1833,2931	1779,1640	925,1068	298	149	2.78050632452e-06	0.000886033452992	0.357,0.331	0.49,0.434	-0.118
10-12m_vs_8-10w	SE	Atg13	ENSMUSG00000027244.14	chr2	-	91680342	91680457	91678726	91678855	91681558	91681695	15328	4,15	0,0	24,6	1,5	263	149	0.000216581105791	0.0187993669021	1.0,1.0	0.931,0.405	0.332
10-12m_vs_8-10w	SE	Atg13	ENSMUSG00000027244.14	chr2	-	91685781	91685878	91685150	91685249	91686500	91686541	15332	8,23	0,0	25,18	5,1	245	149	0.000334782227945	0.0246463100869	1.0,1.0	0.753,0.916	0.165
10-12m_vs_8-10w	SE	Srsf3	ENSMUSG00000071172.12	chr17	+	29039453	29039909	29038489	29038624	29040774	29040813	15419	116,177	119,191	153,120	100,59	298	149	0.000291653170709	0.0228582230511	0.328,0.317	0.433,0.504	-0.146
10-12m_vs_8-10w	SE	Xlr4b	ENSMUSG00000067768.12	chrX	+	73218685	73218785	73217349	73217840	73219973	73220059	15781	126,151	2,7	75,126	8,16	248	149	0.000463593074903	0.0306920307144	0.974,0.928	0.849,0.826	0.114
10-12m_vs_8-10w	SE	Tmem234	ENSMUSG00000028797.20	chr4	+	129601406	129601625	129600982	129601134	129601869	129601930	15826	1,1	0,0	0,0	1,1	298	149	1.77269918744e-08	1.9494840509e-05	1.0,1.0	0.0,0.0	1.0
10-12m_vs_8-10w	SE	Prdx6	ENSMUSG00000026701.15	chr1	-	161249358	161249469	161247256	161247413	161250993	161251169	15902	22,36	0,0	30,25	8,2	259	149	1.457023784e-05	0.00286967457094	1.0,1.0	0.683,0.878	0.22
10-12m_vs_8-10w	SE	Cetn4	ENSMUSG00000045031.18	chr3	-	37310292	37310439	37309936	37310065	37312356	37312420	16077	68,62	0,0	30,34	2,4	295	149	0.000441402287891	0.029554465329	1.0,1.0	0.883,0.811	0.153
10-12m_vs_8-10w	SE	Cbfa2t2	ENSMUSG00000038533.15	chr2	+	154517708	154517962	154510507	154510597	154523903	154524099	16125	3,4	1,0	2,7	7,3	298	149	0.000688883512892	0.0393696295024	0.6,1.0	0.125,0.538	0.469
10-12m_vs_8-10w	SE	Chn2	ENSMUSG00000004633.17	chr6	+	54194025	54194081	54173463	54173502	54215661	54215693	16276	2,5	3,1	2,2	0,0	204	149	0.000861716543039	0.0456874300533	0.327,0.785	1.0,1.0	-0.444

10-12m_vs_8-10w	SE	Sgf29	ENSMUSG00000030714.14	chr7	+	126662565	126662651	126661051	126661148	126663928	126664004	16308	3,7	33,36	1,0	39,37	234	149	0.000849291505517	0.0454615191091	0.055,0.11	0.016,0.0	0.075
10-12m_vs_8-10w	SE	Ubxn11	ENSMUSG00000012126.16	chr4	+	134124833	134124954	134123841	134123919	134125065	134125165	16358	1,7	12,9	23,28	3,11	269	149	3.74452317574e-05	0.00545517696261	0.044,0.301	0.809,0.585	-0.525
10-12m_vs_8-10w	SE	RP23-204P24.2	ENSMUSG00000051297.8	chr16	+	92492013	92492175	92478741	92478968	92497049	92497366	16532	3,4	1,3	2,5	0,0	298	149	0.000129041230493	0.0130464985403	0.6,0.4	1.0,1.0	-0.5
10-12m_vs_8-10w	SE	Plekha1	ENSMUSG00000040268.17	chr7	+	130900516	130900660	130896990	130897450	130902176	130902245	16971	81,109	0,0	138,127	6,5	292	149	0.000465171857563	0.0306920307144	1.0,1.0	0.921,0.928	0.075
10-12m_vs_8-10w	SE	Vrk1	ENSMUSG00000021115.15	chr12	+	106055801	106055910	106042836	106042892	106057842	106057975	17004	2,6	0,0	2,2	3,2	257	149	1.41440324486e-06	0.000595837011387	1.0,1.0	0.279,0.367	0.677
10-12m_vs_8-10w	SE	Pate2	ENSMUSG00000074452.13	chr9	+	35669878	35669902	35669422	35669734	35670478	35670610	17026	0,0	3,1	4,3	2,2	172	149	3.06815364737e-06	0.000949593553861	0.0,0.0	0.634,0.565	-0.599
10-12m_vs_8-10w	SE	RP23-425K3.2	ENSMUSG00000085909.1	chr4	+	12312876	12312997	12271398	12271516	12314307	12314583	17051	4,5	3,5	3,4	1,0	269	149	0.000759745001002	0.042241544786	0.425,0.356	0.624,1.0	-0.422
10-12m_vs_8-10w	SE	Luc7l3	ENSMUSG00000020863.15	chr11	-	94292393	94292472	94291328	94291578	94292926	94293032	17058	3,3	1,3	0,0	8,5	227	149	5.13037548355e-06	0.0013531253808	0.663,0.396	0.0,0.0	0.53
10-12m_vs_8-10w	SE	Zc3h11a	ENSMUSG00000102976.6	chr1	-	133660575	133660621	133657388	133657416	133661236	133661380	17474	0,0	10,2	2,2	1,1	194	149	3.80507186153e-05	0.00545517696261	0.0,0.0	0.606,0.606	-0.606
10-12m_vs_8-10w	SE	Zc3h11a	ENSMUSG00000102976.6	chr1	-	133660575	133660621	133657388	133657416	133661255	133661359	17475	0,1	4,4	3,2	1,3	194	149	0.000928535809138	0.0477346095372	0.0,0.161	0.697,0.339	-0.438
10-12m_vs_8-10w	SE	Lilra6	ENSMUSG00000030427.17	chr7	-	3911955	3912252	3911409	3911712	3912479	3912788	17572	3,2	0,0	1,0	1,4	298	149	1.00103147993e-10	5.06038096963e-07	1.0,1.0	0.333,0.0	0.834
10-12m_vs_8-10w	SE	Copg1	ENSMUSG00000030058.17	chr6	+	87903484	87903560	87902197	87902441	87904087	87904191	17706	0,4	1,3	13,3	0,1	224	149	0.000505117180523	0.0323221713132	0.0,0.47	1.0,0.666	-0.598
10-12m_vs_8-10w	SE	Xiap	ENSMUSG00000025860.15	chrX	+	42066680	42066802	42060393	42060487	42094366	42094717	17713	0,1	2,2	3,4	1,0	270	149	1.33646331002e-06	0.000579089552232	0.0,0.216	0.623,1.0	-0.704
10-12m_vs_8-10w	SE	Echdc2	ENSMUSG00000028601.18	chr4	+	108172738	108172972	108172088	108172175	108173794	108173851	17922	1,5	0,0	8,5	4,2	298	149	1.4934460113e-05	0.00287484005634	1.0,1.0	0.5,0.556	0.472
10-12m_vs_8-10w	SE	Echdc2	ENSMUSG00000028601.18	chr4	+	108172750	108172972	108172088	108172175	108173794	108173851	17924	1,5	0,0	8,5	4,2	298	149	1.4934460113e-05	0.00287484005634	1.0,1.0	0.5,0.556	0.472
10-12m_vs_8-10w	SE	Echdc2	ENSMUSG00000028601.18	chr4	+	108172879	108172972	108172088	108172175	108173794	108173851	17927	1,7	0,0	18,7	4,2	241	149	0.000315133295139	0.0238957699372	1.0,1.0	0.736,0.684	0.29
10-12m_vs_8-10w	SE	Banp	ENSMUSG00000025316.16	chr8	+	122020518	122020644	122007752	122007791	122024002	122024146	17969	14,28	0,0	17,15	1,4	274	149	0.000861261547674	0.0456874300533	1.0,1.0	0.902,0.671	0.214
10-12m_vs_8-10w	SE	Kiz	ENSMUSG00000074749.10	chr2	+	146935802	146935884	146863706	146863869	146950522	146950588	18053	3,0	1,2	2,1	0,0	230	149	0.000892136470147	0.0467346999586	0.66,0.0	1.0,1.0	-0.67
10-12m_vs_8-10w	SE	Kiz	ENSMUSG00000074749.10	chr2	+	146942009	146942175	146863706	146863869	146950522	146950588	18056	2,0	1,2	3,1	0,0	298	149	5.1546491852e-05	0.00662481628967	0.5,0.0	1.0,1.0	-0.75
10-12m_vs_8-10w	SE	RP24-93B5.3	ENSMUSG00000086147.2	chr4	+	146986633	146986760	146982693	146982797	146988926	146990755	18285	2,8	0,0	9,4	3,1	275	149	0.000701421944522	0.0398611704895	1.0,1.0	0.619,0.684	0.349
10-12m_vs_8-10w	SE	Zfp846	ENSMUSG00000058192.16	chr9	+	20589456	20589558	20588508	20588635	20590812	20590884	18513	0,1	2,2	4,2	1,0	250	149	1.9882425174e-06	0.000709475103474	0.0,0.23	0.704,1.0	-0.737

10-12m_vs_8-10w	SE	Pdcd10	ENSMUSG00000027835.11	chr3	-	75533540	75533590	75528809	75528927	75541159	75541378	18576	158,172	9,14	261,245	8,2	198	149	0.0006003481371	0.0357078668395	0.93,0.902	0.961,0.989	-0.059
10-12m_vs_8-10w	SE	Rpl36	ENSMUSG00000057863.5	chr17	+	56613906	56614041	56613585	56613680	56614123	56614243	18644	170,174	8,14	380,134	5,0	283	149	6.90085730559e-06	0.00163523361669	0.918,0.867	0.976,1.0	-0.095
10-12m_vs_8-10w	SE	RP24-498L21.2	ENSMUSG00000116366.1	chr15	-	100190223	100190321	100189899	100190105	100195157	100195311	18900	1,1	0,0	1,0	1,2	246	149	2.80493064886e-06	0.000886033452992	1.0,1.0	0.377,0.0	0.812
10-12m_vs_8-10w	SE	Alkbh3	ENSMUSG00000040174.14	chr2	-	94010262	94010408	94008423	94008592	94010682	94010754	19012	1,2	0,0	1,2	3,1	294	149	6.13130209315e-06	0.0015165516556	1.0,1.0	0.145,0.503	0.676
10-12m_vs_8-10w	SE	Wdr3	ENSMUSG00000033285.15	chr3	-	100142212	100142260	100141554	100141598	100142452	100142545	19042	21,37	0,1	74,41	7,11	196	149	8.00179412468e-05	0.0090899781871	1.0,0.966	0.889,0.739	0.169
10-12m_vs_8-10w	SE	Gpbp1	ENSMUSG00000032745.18	chr13	-	111453372	111453496	111440631	111440816	111487422	111487712	19100	15,24	0,6	46,36	0,0	272	149	0.000517472888881	0.0328044145503	1.0,0.687	1.0,1.0	-0.156
10-12m_vs_8-10w	SE	Tfdp2	ENSMUSG00000032411.15	chr9	+	96223093	96223198	96196274	96196560	96294964	96295127	19238	1,0	0,3	3,5	0,0	253	149	0.000396688017239	0.0277454257257	1.0,0.0	1.0,1.0	-0.5
10-12m_vs_8-10w	SE	Tfdp2	ENSMUSG00000032411.15	chr9	+	96232681	96232748	96196316	96196560	96294964	96295127	19242	1,0	0,3	1,2	0,0	215	149	0.000642193315826	0.0374583951198	1.0,0.0	1.0,1.0	-0.5
10-12m_vs_8-10w	SE	Tfdp2	ENSMUSG00000032411.15	chr9	+	96247049	96247088	96223093	96223198	96273838	96273942	19248	0,0	16,20	2,4	10,13	187	149	0.000564580431249	0.0343861226109	0.0,0.0	0.137,0.197	-0.167
10-12m_vs_8-10w	SE	Pmm1	ENSMUSG00000022474.15	chr15	-	81951923	81952039	81951107	81951612	81952724	81952800	19331	265,499	11,10	547,375	4,2	264	149	0.000394770319715	0.0277170800168	0.931,0.966	0.987,0.991	-0.04
10-12m_vs_8-10w	SE	Cwc22	ENSMUSG00000027014.14	chr2	-	77932698	77932763	77931434	77931545	77936592	77936669	19498	10,27	2,4	26,4	0,0	213	149	0.000783670989365	0.0429828657838	0.778,0.825	1.0,1.0	-0.199
10-12m_vs_8-10w	SE	Cwc22	ENSMUSG00000027014.14	chr2	-	77932698	77932766	77931434	77931545	77936592	77936722	19499	5,20	2,4	19,3	0,0	216	149	8.23765768884e-05	0.00928834183495	0.633,0.775	1.0,1.0	-0.296
10-12m_vs_8-10w	SE	Smarcad1	ENSMUSG00000029920.9	chr6	+	65111775	65111885	65110369	65110552	65114300	65115598	19518	38,53	0,0	55,41	5,6	258	149	1.44013900913e-05	0.00285495792718	1.0,1.0	0.864,0.798	0.169
10-12m_vs_8-10w	SE	Mpv17	ENSMUSG00000107283.3	chr5	-	31145239	31145278	31144699	31144752	31145494	31145590	19591	8,2	0,1	3,2	4,1	187	149	0.000959084863332	0.0486454899494	1.0,0.614	0.374,0.614	0.313
10-12m_vs_8-10w	SE	Aaas	ENSMUSG00000036678.8	chr15	-	102338640	102338725	102338251	102338505	102338816	102338898	19599	53,68	2,1	55,23	5,7	233	149	0.000824822587841	0.044515469594	0.944,0.978	0.876,0.678	0.184
10-12m_vs_8-10w	SE	Tatdn3	ENSMUSG00000026632.17	chr1	-	191052859	191052972	191049281	191049359	191054855	191054911	19618	14,17	0,0	19,8	5,1	261	149	4.9313504256e-05	0.00644984676891	1.0,1.0	0.684,0.82	0.248
10-12m_vs_8-10w	SE	Tatdn3	ENSMUSG00000026632.17	chr1	-	191052859	191052972	191049281	191049362	191054855	191054911	19619	9,9	0,0	15,6	5,1	261	149	2.87252555291e-05	0.00465917500242	1.0,1.0	0.631,0.774	0.298
10-12m_vs_8-10w	SE	Usp48	ENSMUSG00000043411.15	chr4	+	137634897	137635008	137633342	137633465	137644342	137644498	19891	0,5	1,3	9,2	0,0	259	149	0.000120054535339	0.0122193762126	0.0,0.489	1.0,1.0	-0.756
10-12m_vs_8-10w	SE	Eif4g3	ENSMUSG00000028760.16	chr4	+	138082892	138082988	138057948	138058077	138096751	138096931	19930	2,3	0,0	3,2	2,1	244	149	0.000895157818518	0.0468121237818	1.0,1.0	0.478,0.55	0.486
10-12m_vs_8-10w	SE	Dleu2	ENSMUSG00000097589.9	chr14	-	61643963	61644059	61643415	61643455	61648481	61648822	19956	85,54	1,2	42,52	6,7	244	149	0.000388484956315	0.027466520303	0.981,0.943	0.81,0.819	0.147
10-12m_vs_8-10w	SE	Rrp1b	ENSMUSG00000058392.12	chr17	+	32051682	32051849	32051145	32051210	32052738	32052827	20127	55,53	1,0	67,44	7,2	298	149	0.000500059561709	0.0323221713132	0.965,1.0	0.827,0.917	0.11

10-12m_vs_8-10w	SE	Rrp1b	ENSMUSG00000058392.12	chr17	+	32052738	32052827	32051145	32051210	32055104	32055124	20128	0,0	1,2	6,2	10,5	237	149	0.000235948331663	0.0200463553156	0.0,0.0	0.274,0.201	-0.238
10-12m_vs_8-10w	SE	Ccdc155	ENSMUSG00000038292.14	chr7	-	45189636	45189693	45189340	45189400	45189949	45190027	20149	1,2	0,1	0,1	3,2	205	149	8.89699696131e-05	0.00977734836353	1.0,0.592	0.0,0.267	0.663
10-12m_vs_8-10w	SE	H2-Q7	ENSMUSG00000060550.15	chr17	+	35442677	35442794	35442266	35442551	35442966	35442999	20160	8,8	0,1	15,15	8,6	265	149	0.000167486989092	0.0155829689146	1.0,0.818	0.513,0.584	0.361
10-12m_vs_8-10w	SE	H2-Q7	ENSMUSG00000060550.15	chr17	+	35442966	35442999	35442266	35442551	35443348	35443643	20162	6,5	0,0	20,12	5,1	181	149	0.000444379517486	0.0296882712442	1.0,1.0	0.767,0.908	0.162
10-12m_vs_8-10w	SE	Fbxw27	ENSMUSG00000104614.5	chr9	-	109773123	109773282	109766051	109766210	109788020	109788139	20261	7,6	19,15	10,14	7,7	298	149	0.00094555554781	0.0482822311795	0.156,0.167	0.417,0.5	-0.297
10-12m_vs_8-10w	SE	Fbxw27	ENSMUSG00000104614.5	chr9	-	109789678	109789716	109789324	109789488	109790529	109790650	20278	160,203	68,72	117,108	77,96	186	149	0.000117044480012	0.0119531182601	0.653,0.693	0.549,0.474	0.161
10-12m_vs_8-10w	SE	Ak7	ENSMUSG00000041323.6	chr12	+	105738573	105738664	105733432	105733513	105741008	105741086	20497	2,3	2,2	1,2	0,0	239	149	8.956006758e-05	0.00980666573924	0.384,0.483	1.0,1.0	-0.567
10-12m_vs_8-10w	SE	Snap91	ENSMUSG00000033419.15	chr9	-	86769458	86769561	86765922	86767336	86773518	86773623	20538	3,4	5,7	11,13	2,3	251	149	0.000343776535229	0.025005002614	0.263,0.253	0.766,0.72	-0.485
10-12m_vs_8-10w	SE	Pwwp2a	ENSMUSG00000044950.7	chr11	+	43717029	43717149	43704533	43705477	43720220	43720411	20552	0,1	9,13	5,2	8,3	268	149	0.000319408070686	0.0239648385706	0.0,0.041	0.258,0.27	-0.244
10-12m_vs_8-10w	SE	Dppa4	ENSMUSG00000058550.14	chr16	+	48292910	48293106	48291013	48291314	48293698	48294292	20560	1164,1464	96,107	2430,1736	80,89	298	149	1.42205925516e-05	0.00283766310975	0.858,0.872	0.938,0.907	-0.057
10-12m_vs_8-10w	SE	Rbm39	ENSMUSG00000027620.16	chr2	-	156177751	156177906	156177335	156177385	156179176	156179240	20628	34,46	66,91	9,15	61,53	298	149	0.000697350314378	0.0397581435816	0.205,0.202	0.069,0.124	0.107
10-12m_vs_8-10w	SE	Rbm39	ENSMUSG00000027620.16	chr2	-	156178879	156178952	156177632	156177906	156179176	156179240	20631	351,482	34,46	532,379	7,13	221	149	2.24537466398e-09	5.38457304111e-06	0.874,0.876	0.981,0.952	-0.091
10-12m_vs_8-10w	SE	RP23-147K15.4	ENSMUSG00000087403.9	chrX	-	152317354	152317434	152315215	152315419	152327160	152327239	20856	1,3	6,8	1,3	1,0	228	149	0.000722034478276	0.0407063713022	0.098,0.197	0.395,1.0	-0.55
10-12m_vs_8-10w	SE	Styx	ENSMUSG00000053205.9	chr14	+	45358216	45358307	45356712	45356766	45359258	45359356	20888	0,0	9,25	6,5	19,30	239	149	0.000110122907659	0.0115177169386	0.0,0.0	0.164,0.094	-0.129
10-12m_vs_8-10w	SE	Timm44	ENSMUSG00000002949.15	chr8	-	4269854	4270025	4261907	4261997	4274136	4274232	20928	13,11	0,0	13,7	5,8	298	149	1.064049584e-07	7.06675203677e-05	1.0,1.0	0.565,0.304	0.566
10-12m_vs_8-10w	SE	Fanca	ENSMUSG00000032815.16	chr8	-	123270231	123270294	123269859	123269965	123274147	123274223	20931	1,1	0,0	5,3	4,1	211	149	2.51732616195e-05	0.00424183443434	1.0,1.0	0.469,0.679	0.426
10-12m_vs_8-10w	SE	Inip	ENSMUSG00000038544.14	chr4	-	59773915	59774006	59769636	59772459	59775446	59775549	20946	86,111	0,1	122,99	5,6	239	149	0.000940278436008	0.0481749750719	1.0,0.986	0.938,0.911	0.069
10-12m_vs_8-10w	SE	Slc5a11	ENSMUSG00000030769.15	chr7	+	123232987	123233104	123228883	123228991	123235597	123235754	20959	0,3	2,3	3,1	1,0	265	149	0.000855774967048	0.0456177689377	0.0,0.36	0.628,1.0	-0.634
10-12m_vs_8-10w	SE	Agrp	ENSMUSG00000005705.10	chr8	-	105567605	105567726	105567359	105567492	105579649	105579845	21113	19,32	0,0	30,11	4,4	269	149	1.68379414514e-05	0.00307657591664	1.0,1.0	0.806,0.604	0.295
10-12m_vs_8-10w	SE	Pkd1l2	ENSMUSG00000034416.18	chr8	-	117011497	117011625	117009322	117009730	117013863	117013997	21147	0,1	12,5	4,5	5,8	276	149	0.000183659503243	0.0166783724337	0.0,0.097	0.302,0.252	-0.229
10-12m_vs_8-10w	SE	RP24-213G21.1	ENSMUSG00000113856.1	chr13	+	41910074	41910252	41885541	41885637	41911218	41911293	21213	33,45	6,1	38,26	0,0	298	149	0.000400960615	0.0277660648712	0.733,0.957	1.0,1.0	-0.155

10-12m_vs_8-10w	SE	Hivep3	ENSMUSG00000028634.17	chr4	+	119814677	119814790	119762219	119762348	119872036	119872272	21233	2,8	1,4	10,7	0,0	261	149	3.99323495144e-05	0.00563052218681	0.533,0.533	1.0,1.0	-0.467
10-12m_vs_8-10w	SE	Baz2a	ENSMUSG00000040054.16	chr10	+	128125498	128125645	128125268	128125401	128126017	128126241	21294	21,35	0,0	44,36	0,7	295	149	0.000815763700006	0.0441050423973	1.0,1.0	1.0,0.722	0.139
10-12m_vs_8-10w	SE	RP24-166N8.6	ENSMUSG00000057802.8	chr9	+	111006776	111006939	111006342	111006430	111007452	111008029	21398	1,1	0,0	1,1	2,2	298	149	2.14886158689e-06	0.000749162307954	1.0,1.0	0.2,0.2	0.8
10-12m_vs_8-10w	SE	Fuom	ENSMUSG00000025466.19	chr7	-	140101331	140101402	140100556	140101188	140101600	140101669	21584	10,16	1,0	8,10	7,2	219	149	0.000143514872677	0.0139966225182	0.872,1.0	0.437,0.773	0.331
10-12m_vs_8-10w	SE	Rab25	ENSMUSG00000008601.12	chr3	-	88543319	88543513	88542700	88542781	88548032	88548249	21627	8,3	0,0	9,3	2,2	298	149	0.000668278336833	0.038682111445	1.0,1.0	0.692,0.429	0.44
10-12m_vs_8-10w	SE	Adrm1	ENSMUSG00000039041.15	chr2	+	180175159	180175392	180174864	180174946	180176073	180176286	21644	73,88	1,10	143,104	0,1	298	149	7.9291223155e-05	0.00907412341473	0.973,0.815	1.0,0.981	-0.097
10-12m_vs_8-10w	SE	Adrm1	ENSMUSG00000039041.15	chr2	+	180175853	180175956	180175354	180175755	180176073	180176282	21649	107,148	10,5	225,146	0,3	251	149	8.10786953775e-05	0.00917611160259	0.864,0.946	1.0,0.967	-0.079
10-12m_vs_8-10w	SE	Mfsd10	ENSMUSG00000001082.12	chr5	-	34634590	34634699	34634422	34634513	34634850	34634962	21690	2,15	0,0	12,4	2,4	257	149	2.13240448315e-05	0.0036958834502	1.0,1.0	0.777,0.367	0.428
10-12m_vs_8-10w	SE	Spg7	ENSMUSG00000000738.18	chr8	+	123093590	123093747	123091634	123091750	123095157	123095235	21729	54,36	5,2	85,56	1,0	298	149	0.000975467263596	0.0489849297552	0.844,0.9	0.977,1.0	-0.116
10-12m_vs_8-10w	SE	Rab11fip1	ENSMUSG00000031488.14	chr8	-	27151596	27153159	27150694	27150803	27154145	27154935	21793	20,18	0,0	30,26	5,6	298	149	8.48767039974e-06	0.00189872333803	1.0,1.0	0.75,0.684	0.283
10-12m_vs_8-10w	SE	Pkmyt1	ENSMUSG00000023908.7	chr17	+	23735199	23735372	23732336	23732824	23736323	23736401	22108	7,7	1,5	2,3	0,0	298	149	1.62055747541e-05	0.00299714199919	0.778,0.412	1.0,1.0	-0.405
10-12m_vs_8-10w	SE	Eml3	ENSMUSG00000071647.5	chr19	+	8940578	8940677	8939187	8939285	8941129	8941582	22196	4,2	0,0	1,1	1,3	247	149	1.20182021324e-06	0.000528295780982	1.0,1.0	0.376,0.167	0.728
10-12m_vs_8-10w	SE	Atg3	ENSMUSG00000022663.3	chr16	+	45182655	45182690	45178229	45178311	45182820	45182976	22203	98,112	8,5	200,140	1,1	183	149	0.000115395705899	0.0118646344258	0.909,0.948	0.994,0.991	-0.064
10-12m_vs_8-10w	SE	Cyc1	ENSMUSG00000022551.8	chr15	+	76344581	76344708	76343532	76344498	76345476	76345577	22288	1,13	3,2	15,6	0,0	275	149	4.53827145356e-05	0.00611779162035	0.153,0.779	1.0,1.0	-0.534
10-12m_vs_8-10w	SE	Cyc1	ENSMUSG00000022551.8	chr15	+	76344811	76344969	76343532	76344498	76345476	76345577	22292	1,1	3,2	3,4	0,0	298	149	1.08949980371e-07	7.06675203677e-05	0.143,0.2	1.0,1.0	-0.829
10-12m_vs_8-10w	SE	Psmd14	ENSMUSG00000026914.15	chr2	+	61764848	61764999	61720443	61720571	61797394	61797457	22331	0,3	2,1	10,2	0,0	298	149	0.000296889621406	0.0229378525288	0.0,0.6	1.0,1.0	-0.7
10-12m_vs_8-10w	SE	Psmd14	ENSMUSG00000026914.15	chr2	+	61776672	61776780	61760974	61761094	61797394	61797457	22345	1,1	0,0	4,0	6,1	256	149	1.47623554492e-08	1.86565417971e-05	1.0,1.0	0.28,0.0	0.86
10-12m_vs_8-10w	SE	Ap4m1	ENSMUSG00000019518.10	chr5	+	138176051	138176118	138175911	138175974	138176209	138176263	22790	0,6	2,5	6,9	0,0	215	149	2.53233929604e-07	0.000147708429208	0.0,0.454	1.0,1.0	-0.773
10-12m_vs_8-10w	SE	Uap1l1	ENSMUSG00000026956.15	chr2	-	25362070	25362137	25359888	25361739	25363805	25363857	22956	7,11	0,10	17,7	0,0	215	149	0.000428014990028	0.0289678928642	1.0,0.433	1.0,1.0	-0.284
10-12m_vs_8-10w	SE	Uap1l1	ENSMUSG00000026956.15	chr2	-	25362662	25362848	25359888	25361739	25363805	25363857	22958	1,3	0,10	2,3	0,0	298	149	0.00049275132912	0.0319351294093	1.0,0.13	1.0,1.0	-0.435
10-12m_vs_8-10w	SE	Uap1l1	ENSMUSG00000026956.15	chr2	-	25363243	25363384	25359888	25361739	25363805	25363999	22961	10,21	0,10	13,10	0,0	289	149	0.000256621603458	0.0210136957256	1.0,0.52	1.0,1.0	-0.24

10-12m_vs_8-10w	SE	Ddx60	ENSMUSG00000037921.15	chr8	+	62012270	62012471	62009941	62010046	62017140	62017245	23026	2,8	0,0	4,0	4,2	298	149	1.27646255788e-08	1.75983572014e-05	1.0,1.0	0.333,0.0	0.834
10-12m_vs_8-10w	SE	Dph3	ENSMUSG00000021905.14	chr14	-	32084906	32084981	32080568	32083246	32085409	32085609	23102	341,414	12,10	466,305	47,25	223	149	2.70024446369e-06	0.000886033452992	0.95,0.965	0.869,0.891	0.078
10-12m_vs_8-10w	SE	Dph3	ENSMUSG00000021905.14	chr14	-	32084906	32085107	32080568	32083246	32085409	32085609	23103	150,192	12,10	221,142	47,25	298	149	9.9184152198e-06	0.00211855952135	0.862,0.906	0.702,0.74	0.163
10-12m_vs_8-10w	SE	Edrf1	ENSMUSG00000039990.16	chr7	+	133646989	133647091	133643992	133644149	133647344	133647466	23147	34,38	0,1	40,22	7,1	250	149	0.000591624224712	0.035414553526	1.0,0.958	0.773,0.929	0.128
10-12m_vs_8-10w	SE	RP24-103K4.2	ENSMUSG00000087700.2	chr12	-	73928822	73929100	73926480	73926754	73949623	73949785	23211	5,6	0,0	2,1	2,3	298	149	3.78233856457e-08	3.27777460006e-05	1.0,1.0	0.333,0.143	0.762
10-12m_vs_8-10w	SE	Szrd1	ENSMUSG00000040842.17	chr4	-	141120323	141120373	141118526	141118781	141139691	141139727	23235	3,2	2,6	9,2	1,0	198	149	3.80226491997e-05	0.00545517696261	0.53,0.201	0.871,1.0	-0.57
10-12m_vs_8-10w	SE	Tra2b	ENSMUSG00000022858.15	chr16	-	22258985	22259261	22254983	22255117	22265740	22265829	23253	558,611	29,26	773,455	93,31	298	149	0.000181272167663	0.0165607413174	0.906,0.922	0.806,0.88	0.071
10-12m_vs_8-10w	SE	AC152939.2	ENSMUSG00000045411.16	chr7	+	44247909	44248059	44246786	44246823	44248251	44248295	23260	2,1	2,1	1,2	0,0	298	149	8.40739154321e-05	0.00937516885651	0.333,0.333	1.0,1.0	-0.667
10-12m_vs_8-10w	SE	RP23-129J8.5	ENSMUSG00000116220.1	chr15	-	80696703	80697043	80696469	80696615	80697566	80697602	23334	2,0	5,1	2,3	0,0	298	149	4.88702633916e-10	1.64698217659e-06	0.167,0.0	1.0,1.0	-0.917
10-12m_vs_8-10w	SE	Lig3	ENSMUSG00000020697.16	chr11	+	82789546	82789713	82788755	82788915	82789816	82789894	23454	2,6	7,9	8,14	2,5	298	149	0.000834440795358	0.0447954402903	0.125,0.25	0.667,0.583	-0.438
10-12m_vs_8-10w	SE	Pum1	ENSMUSG00000028580.15	chr4	+	130718153	130718262	130701041	130701110	130730277	130730548	23480	1,1	3,2	2,2	0,0	257	149	3.48835317299e-07	0.0001923731638	0.162,0.225	1.0,1.0	-0.807
10-12m_vs_8-10w	SE	Pum1	ENSMUSG00000028580.15	chr4	+	130727997	130728164	130701041	130701110	130730277	130730548	23481	1,1	3,2	4,4	0,0	298	149	9.8230742096e-08	7.06675203677e-05	0.143,0.2	1.0,1.0	-0.829
10-12m_vs_8-10w	SE	Cfap157	ENSMUSG00000038987.8	chr2	-	32777827	32778017	32777380	32777569	32778116	32778283	23564	1,0	5,1	5,4	0,0	298	149	7.888956155e-11	4.78559858275e-07	0.091,0.0	1.0,1.0	-0.955
10-12m_vs_8-10w	SE	Loxhd1	ENSMUSG00000032818.15	chr18	+	77293233	77293314	77286364	77286479	77321572	77321671	23604	0,5	1,3	6,1	0,0	229	149	0.000244219625386	0.020519184093	0.0,0.52	1.0,1.0	-0.74
10-12m_vs_8-10w	SE	Cab39l	ENSMUSG00000021981.10	chr14	+	59444196	59444330	59440971	59441022	59459093	59459241	23705	0,0	3,1	2,2	0,1	282	149	6.97858418153e-06	0.00164083284349	0.0,0.0	1.0,0.514	-0.757
10-12m_vs_8-10w	SE	Ube2q2	ENSMUSG00000032307.16	chr9	+	55194986	55195035	55191841	55191900	55195423	55195519	23736	94,94	2,1	76,89	10,9	197	149	2.84290075957e-05	0.00463591521175	0.973,0.986	0.852,0.882	0.113
10-12m_vs_8-10w	SE	Cars2	ENSMUSG00000056228.10	chr8	-	11526001	11526069	11518923	11518990	11529579	11529713	23752	10,10	0,0	28,21	1,6	216	149	0.000593142105147	0.035414553526	1.0,1.0	0.951,0.707	0.171
10-12m_vs_8-10w	SE	Smu1	ENSMUSG00000028409.11	chr4	-	40739518	40739686	40736541	40737395	40744030	40744158	23824	1,1	0,0	1,2	5,1	298	149	2.00869507694e-08	2.03085767929e-05	1.0,1.0	0.091,0.5	0.705
10-12m_vs_8-10w	SE	Gtf2i	ENSMUSG00000060261.15	chr5	-	134239496	134239571	134237833	134238892	134240242	134240284	23888	10,2	0,0	10,14	4,1	223	149	0.000790972475038	0.0432332843647	1.0,1.0	0.626,0.903	0.236
10-12m_vs_8-10w	SE	Cldn34c1	ENSMUSG00000079450.11	chrX	+	123122897	123122938	123117453	123117569	123142609	123143270	24001	0,0	23,30	6,1	12,27	189	149	0.000547897184169	0.0339841911923	0.0,0.0	0.283,0.028	-0.156
10-12m_vs_8-10w	SE	Ddx1	ENSMUSG00000037149.10	chr12	-	13223675	13223718	13220737	13220833	13223792	13223865	24066	21,19	3,3	31,23	0,0	191	149	0.000689237851234	0.0393696295024	0.845,0.832	1.0,1.0	-0.161

10-12m_vs_8-10w	SE	Kxd1	ENSMUSG00000055553.16	chr8	-	70518364	70518517	70515394	70515441	70519936	70520061	24167	43,82	1,0	59,47	3,5	298	149	0.000926582867723	0.0477196764268	0.956,1.0	0.908,0.825	0.111
10-12m_vs_8-10w	SE	RP23-440J15.6	ENSMUSG00000092463.1	chrX	-	101265629	101265726	101265375	101265442	101266180	101266343	24254	4,11	5,8	3,10	0,1	245	149	4.00973522914e-05	0.00563052218681	0.327,0.455	1.0,0.859	-0.539
10-12m_vs_8-10w	SE	Gtf3c1	ENSMUSG00000032777.9	chr7	-	125662576	125662655	125661957	125662447	125662958	125663094	24341	6,11	3,2	4,9	0,0	227	149	0.000197647316524	0.0176838960398	0.568,0.783	1.0,1.0	-0.325
10-12m_vs_8-10w	SE	Hnrnpc	ENSMUSG00000060373.15	chr14	-	52097321	52098544	52084114	52084391	52103888	52104004	24397	222,293	145,213	206,124	271,192	298	149	4.0224482023e-07	0.000210353235214	0.434,0.408	0.275,0.244	0.161
10-12m_vs_8-10w	SE	Hnrnpc	ENSMUSG00000060373.15	chr14	-	52098014	52098040	52081788	52081903	52103888	52103999	24399	7,12	1,8	3,1	0,0	174	149	4.93344911275e-05	0.00644984676891	0.857,0.562	1.0,1.0	-0.291
10-12m_vs_8-10w	SE	Hnrnpc	ENSMUSG00000060373.15	chr14	-	52098014	52098544	52084271	52084391	52103888	52103993	24402	275,361	145,213	265,201	271,192	298	149	2.9828828406e-05	0.00476177997043	0.487,0.459	0.328,0.344	0.137
10-12m_vs_8-10w	SE	Hnrnpc	ENSMUSG00000060373.15	chr14	-	52098315	52098544	52084114	52084391	52103888	52103990	24403	182,222	145,213	147,87	271,192	298	149	2.55355574463e-08	2.42037185282e-05	0.386,0.343	0.213,0.185	0.166
10-12m_vs_8-10w	SE	Klf17	ENSMUSG00000048626.5	chr4	-	117760314	117761113	117757835	117759288	117765500	117765648	24468	111,341	6,16	194,164	3,1	298	149	0.000519885295575	0.0328513352085	0.902,0.914	0.97,0.988	-0.071
10-12m_vs_8-10w	SE	Ergic2	ENSMUSG00000030304.11	chr6	-	148190918	148191023	148189499	148189584	148195198	148195241	24522	3,2	0,0	10,6	2,3	253	149	0.000107174096218	0.0113439078197	1.0,1.0	0.746,0.541	0.357
10-12m_vs_8-10w	SE	Qrsl1	ENSMUSG00000019863.7	chr10	-	43876471	43876677	43874187	43874706	43881472	43881590	24628	2,8	0,0	12,7	7,2	298	149	1.1660938295e-06	0.000520129293273	1.0,1.0	0.462,0.636	0.451
10-12m_vs_8-10w	SE	Kif16b	ENSMUSG00000038844.10	chr2	-	142648427	142648511	142617473	142619916	142672288	142672378	24812	24,23	1,6	12,23	0,0	232	149	0.000283913796094	0.0224254930972	0.939,0.711	1.0,1.0	-0.175
10-12m_vs_8-10w	SE	Dhps	ENSMUSG00000060038.14	chr8	+	85073502	85073599	85072451	85072616	85073680	85073767	24862	36,43	17,25	56,38	7,5	245	149	2.5707410688e-05	0.0043079086938	0.563,0.511	0.83,0.822	-0.289
10-12m_vs_8-10w	SE	Trpc1	ENSMUSG00000032839.13	chr9	-	95710115	95710291	95708645	95708847	95716082	95716226	24940	0,3	2,1	2,3	0,0	298	149	0.000297206687673	0.0229378525288	0.0,0.6	1.0,1.0	-0.7
10-12m_vs_8-10w	SE	Abo	ENSMUSG00000015787.15	chr2	-	26846573	26846609	26845955	26846090	26848250	26848307	25061	3,1	0,0	1,0	0,4	184	149	0.000465475392458	0.0306920307144	1.0,1.0	1.0,0.0	0.5
10-12m_vs_8-10w	SE	Fbxw16	ENSMUSG00000074062.5	chr9	-	109446548	109446667	109439540	109439699	109449018	109449124	25225	5,8	4,1	5,6	0,0	267	149	8.68416273314e-05	0.00960156084581	0.411,0.817	1.0,1.0	-0.386
10-12m_vs_8-10w	SE	Rsrp1	ENSMUSG00000037266.18	chr4	+	134923874	134924482	134923591	134923767	134925292	134925432	25260	320,621	8,19	593,454	2,1	298	149	1.53119312218e-06	0.000616728997731	0.952,0.942	0.993,0.996	-0.047
10-12m_vs_8-10w	SE	Zyg11b	ENSMUSG00000034636.9	chr4	-	108272217	108272383	108265817	108266572	108300890	108301096	25299	115,144	0,2	148,143	4,11	298	149	0.000251065173181	0.0207642664043	1.0,0.973	0.949,0.867	0.079
10-12m_vs_8-10w	SE	Cdk9	ENSMUSG00000009555.16	chr2	-	32709466	32709615	32705783	32708289	32710042	32710209	25446	13,45	3,9	40,29	1,0	297	149	8.90241290241e-06	0.00194258335067	0.685,0.715	0.953,1.0	-0.276
10-12m_vs_8-10w	SE	Cdk9	ENSMUSG00000009555.16	chr2	-	32709466	32709944	32707848	32708289	32710042	32710209	25447	19,55	3,9	55,39	1,0	298	149	8.48534271403e-06	0.00189872333803	0.76,0.753	0.965,1.0	-0.226
10-12m_vs_8-10w	SE	Tmem62	ENSMUSG00000054484.14	chr2	+	120993477	120993637	120990330	120990486	120996405	120996519	25455	11,47	0,0	27,17	4,4	298	149	1.13710550518e-05	0.00239510743595	1.0,1.0	0.771,0.68	0.274
10-12m_vs_8-10w	SE	Ptpn4	ENSMUSG00000026384.13	chr1	-	119765877	119765922	119765428	119765481	119773048	119773127	25473	5,4	0,0	19,11	6,5	193	149	3.55379642571e-05	0.00533614848456	1.0,1.0	0.71,0.629	0.331

10-12m_vs_8-10w	SE	Clk2	ENSMUSG00000068917.12	chr3	+	89169614	89169702	89168694	89168923	89170048	89170115	25541	5,10	0,0	10,6	3,3	236	149	1.41935936494e-05	0.00283766310975	1.0,1.0	0.678,0.558	0.382
10-12m_vs_8-10w	SE	Clk2	ENSMUSG00000068917.12	chr3	+	89169617	89169702	89168694	89168923	89170048	89170115	25543	4,7	0,0	6,11	3,3	233	149	2.91413671535e-05	0.00470152556986	1.0,1.0	0.561,0.701	0.369
10-12m_vs_8-10w	SE	Ctla2b	ENSMUSG00000074874.10	chr13	-	60896627	60896745	60896237	60896360	60897334	60897405	25548	2,2	5,1	7,2	0,0	266	149	8.87699425078e-08	6.73120281551e-05	0.183,0.528	1.0,1.0	-0.645
10-12m_vs_8-10w	SE	Fam188a	ENSMUSG00000026767.12	chr2	-	12364446	12364519	12347262	12348203	12396178	12396258	25558	0,2	3,1	1,1	0,0	221	149	4.31454016225e-06	0.0011896756151	0.0,0.574	1.0,1.0	-0.713
10-12m_vs_8-10w	SE	Klf7	ENSMUSG00000025959.13	chr1	-	64120899	64121139	64078706	64079334	64122201	64122282	25624	3,19	0,1	10,9	5,2	298	149	0.000320847244587	0.0239695019103	1.0,0.905	0.5,0.692	0.357
10-12m_vs_8-10w	SE	Slbp	ENSMUSG00000004642.13	chr5	-	33649742	33649847	33645499	33646069	33651962	33652079	25670	2900,4944	52,78	2709,2609	92,59	253	149	0.000863106967147	0.0456874300533	0.97,0.974	0.945,0.963	0.018
10-12m_vs_8-10w	SE	Gamt	ENSMUSG00000020150.13	chr10	-	80259767	80259913	80259176	80259244	80260720	80260918	25712	0,0	1,5	5,6	0,0	294	149	5.81534820299e-12	8.81926631724e-08	0.0,0.0	1.0,1.0	-1.0
10-12m_vs_8-10w	SE	Apoa1bp	ENSMUSG00000028070.7	chr3	-	88056985	88057133	88056519	88056776	88057723	88057837	25834	115,141	62,60	207,164	26,32	296	149	6.92126607138e-08	5.38279285156e-05	0.483,0.542	0.8,0.721	-0.248
10-12m_vs_8-10w	SE	Yipf1	ENSMUSG00000057375.13	chr4	+	107335648	107335707	107335008	107335119	107336113	107336194	25868	1,0	7,12	9,7	9,8	207	149	3.2877577133e-06	0.000987336427744	0.093,0.0	0.419,0.386	-0.356
10-12m_vs_8-10w	SE	Fam184a	ENSMUSG00000019856.14	chr10	-	53640944	53641062	53635231	53635434	53647571	53647756	26089	14,3	0,0	5,5	6,4	266	149	3.08489365164e-08	2.83539119236e-05	1.0,1.0	0.318,0.412	0.635
10-12m_vs_8-10w	SE	Fam184a	ENSMUSG00000019856.14	chr10	-	53646982	53647129	53634935	53635434	53647571	53647756	26092	54,60	0,0	61,51	6,4	295	149	3.48095529026e-05	0.00531070424477	1.0,1.0	0.837,0.866	0.149
10-12m_vs_8-10w	SE	Setdb2	ENSMUSG00000071350.12	chr14	-	59423387	59423484	59419000	59419561	59431142	59431268	26122	58,59	5,6	82,72	0,1	245	149	6.50198973683e-05	0.00795209075435	0.876,0.857	1.0,0.978	-0.122
10-12m_vs_8-10w	SE	Fbxw18	ENSMUSG00000074059.3	chr9	-	109693169	109693379	109691500	109691659	109701240	109701404	26196	427,480	8,12	410,397	0,2	298	149	0.000200785643945	0.0179118510779	0.964,0.952	1.0,0.99	-0.037
10-12m_vs_8-10w	SE	Fbxw18	ENSMUSG00000074059.3	chr9	-	109700063	109700182	109691500	109691659	109701240	109701404	26197	265,343	8,12	255,242	0,2	267	149	0.000100173262279	0.0107502432123	0.949,0.941	1.0,0.985	-0.047
10-12m_vs_8-10w	SE	Clec10a	ENSMUSG00000000318.16	chr11	+	70169128	70169224	70168582	70168696	70169307	70169379	26275	1,7	1,6	14,5	0,0	244	149	4.44558251989e-07	0.00022854061595	0.379,0.416	1.0,1.0	-0.603
10-12m_vs_8-10w	SE	Ppp2r2d	ENSMUSG00000041769.13	chr7	+	138860836	138860909	138846619	138846704	138868409	138868507	26302	0,0	22,39	5,5	22,33	221	149	0.000132393065143	0.0131659477339	0.0,0.0	0.133,0.093	-0.113
10-12m_vs_8-10w	SE	Epn2	ENSMUSG00000001036.17	chr11	-	61548007	61548042	61546505	61546911	61565821	61565894	26387	2,8	2,4	6,2	0,0	183	149	1.50867888348e-05	0.0028779710198	0.449,0.62	1.0,1.0	-0.466
10-12m_vs_8-10w	SE	Matr3	ENSMUSG00000037236.14	chr18	+	35562894	35563100	35562191	35562259	35571842	35572123	26470	1,1	1,4	1,1	0,0	298	149	3.55214890835e-09	6.7337642837e-06	0.333,0.111	1.0,1.0	-0.778
10-12m_vs_8-10w	SE	Rbms2	ENSMUSG00000040043.16	chr10	-	128145672	128145764	128143394	128143552	128146601	128146660	26498	7,19	0,7	14,7	0,0	240	149	0.000313657754012	0.0238434920725	1.0,0.628	1.0,1.0	-0.186
10-12m_vs_8-10w	SE	Map2k2	ENSMUSG00000035027.18	chr10	+	81118976	81119160	81118151	81118229	81119256	81119381	26632	1,1	1,1	7,7	1,0	298	149	0.00044780077722	0.0297856258199	0.333,0.333	0.778,1.0	-0.556
10-12m_vs_8-10w	SE	Faxc	ENSMUSG00000028246.13	chr4	+	21948691	21948888	21936632	21936768	21982385	21982502	26641	1,2	2,4	5,7	0,1	298	149	6.41244549549e-06	0.00155596707459	0.2,0.2	1.0,0.778	-0.689

10-12m_vs_8-10w	SE	Tpra1	ENSMUSG00000002871.14	chr6	+	88910796	88910877	88910340	88910401	88911682	88911747	26665	2,6	1,5	4,7	1,0	229	149	0.000522440831078	0.032944184714	0.565,0.438	0.722,1.0	-0.36
10-12m_vs_8-10w	SE	Tiam1	ENSMUSG00000002489.15	chr16	-	89908546	89908715	89897604	89898578	89955935	89956107	26899	19,12	7,1	14,14	0,1	298	149	0.000898820744924	0.0469227745513	0.576,0.857	1.0,0.875	-0.221
10-12m_vs_8-10w	SE	Capn11	ENSMUSG00000058626.16	chr17	-	45630725	45630784	45630203	45630611	45631787	45631901	26991	25,34	4,4	49,44	0,0	207	149	7.90686445153e-05	0.00907412341473	0.818,0.86	1.0,1.0	-0.161
10-12m_vs_8-10w	SE	Gsdmd	ENSMUSG00000022575.5	chr15	+	75866789	75866863	75866295	75866471	75866996	75867408	27028	0,0	1,2	1,2	1,0	222	149	0.000148626387032	0.0142207790065	0.0,0.0	0.402,1.0	-0.701
10-12m_vs_8-10w	SE	Pbrm1	ENSMUSG00000042323.17	chr14	+	31064745	31064851	31061468	31061745	31067321	31067964	27173	74,79	1,0	112,92	3,8	254	149	0.000884608437272	0.0464205164548	0.977,1.0	0.956,0.871	0.075
10-12m_vs_8-10w	SE	Rsl1d1	ENSMUSG00000005846.12	chr16	-	11199570	11199719	11199384	11199486	11201294	11201430	27804	2737,3976	32,53	3381,2753	86,61	297	149	0.000144643494737	0.0140165553957	0.977,0.974	0.952,0.958	0.021
10-12m_vs_8-10w	SE	Napepld	ENSMUSG00000044968.16	chr5	-	21683155	21683465	21675454	21676101	21700974	21701394	27908	4,21	0,0	15,16	2,4	298	149	5.39219556637e-05	0.00684312484199	1.0,1.0	0.789,0.667	0.272
10-12m_vs_8-10w	SE	Adgrb3	ENSMUSG00000033569.17	chr1	-	25074684	25074789	25067475	25068356	25084243	25084279	27909	16,12	0,2	6,10	3,7	253	149	0.000319994712377	0.0239648385706	1.0,0.779	0.541,0.457	0.39
10-12m_vs_8-10w	SE	Hmbs	ENSMUSG00000032126.16	chr9	-	44337117	44337171	44336338	44336845	44337914	44337975	27934	7,14	3,3	10,10	0,0	202	149	5.22648732237e-05	0.00666069693171	0.633,0.775	1.0,1.0	-0.296
10-12m_vs_8-10w	SE	Hmbs	ENSMUSG00000032126.16	chr9	-	44337355	44337475	44336338	44336845	44337914	44337975	27938	11,26	3,3	24,20	0,0	268	149	0.00011471140038	0.0118343928059	0.671,0.828	1.0,1.0	-0.25
10-12m_vs_8-10w	SE	Kntc1	ENSMUSG00000029414.11	chr5	+	123812009	123812105	123811305	123811441	123812630	123812687	28001	58,70	6,12	96,88	2,4	244	149	0.000762931502458	0.042241544786	0.855,0.781	0.967,0.931	-0.131
10-12m_vs_8-10w	SE	RP24-502H14.1	ENSMUSG00000112161.1	chr10	-	119138750	119138800	119135850	119135892	119140681	119140830	28022	2,2	0,2	0,0	1,5	198	149	0.000107339076992	0.0113439078197	1.0,0.429	0.0,0.0	0.715
10-12m_vs_8-10w	SE	Qpctl	ENSMUSG00000030407.2	chr7	-	19144883	19145036	19144659	19144759	19146869	19147151	28040	3,2	0,0	5,3	2,2	298	149	0.00016897178585	0.0156730374208	1.0,1.0	0.556,0.429	0.507
10-12m_vs_8-10w	SE	Rnf170	ENSMUSG00000013878.18	chr8	+	26138868	26138979	26135628	26135702	26140810	26141078	28176	4,3	2,2	5,11	0,0	259	149	0.000114612591995	0.0118343928059	0.535,0.463	1.0,1.0	-0.501
10-12m_vs_8-10w	SE	Slco4a1	ENSMUSG00000038963.15	chr2	+	180472404	180472570	180472053	180472249	180473102	180473167	28182	3,1	1,3	6,6	0,1	298	149	0.00031713474774	0.0239278956062	0.6,0.143	1.0,0.75	-0.504
10-12m_vs_8-10w	SE	Dock9	ENSMUSG00000025558.17	chr14	-	121577346	121577424	121575912	121576019	121578154	121578212	28310	1,7	3,4	4,5	0,0	226	149	3.71786804676e-07	0.000197836238116	0.18,0.536	1.0,1.0	-0.642
10-12m_vs_8-10w	SE	Catsperd	ENSMUSG00000040828.9	chr17	+	56650742	56650893	56647727	56647811	56651794	56651890	28321	2,7	0,0	4,1	4,3	298	149	4.80956019455e-09	8.58110413299e-06	1.0,1.0	0.333,0.143	0.762
10-12m_vs_8-10w	SE	Catsperd	ENSMUSG00000040828.9	chr17	+	56659528	56659581	56655988	56656047	56660170	56660245	28325	0,0	1,9	6,0	13,4	201	149	0.000764584355527	0.042241544786	0.0,0.0	0.255,0.0	-0.128
10-12m_vs_8-10w	SE	Efemp1	ENSMUSG00000020467.15	chr11	+	28916849	28916969	28915660	28915780	28921402	28921526	28439	2,6	1,0	0,2	2,12	268	149	1.01302055999e-06	0.000480092603204	0.527,1.0	0.0,0.085	0.721
10-12m_vs_8-10w	SE	Arntl2	ENSMUSG00000040187.15	chr6	+	146825166	146825228	146823093	146823244	146828098	146828159	28584	1,16	3,4	11,4	0,0	210	149	1.81108586826e-05	0.00326976461132	0.191,0.739	1.0,1.0	-0.535
10-12m_vs_8-10w	SE	Arntl2	ENSMUSG00000040187.15	chr6	+	146827215	146827324	146823093	146823244	146828098	146828159	28585	9,23	3,4	16,19	0,0	257	149	3.43591323603e-05	0.00529008550061	0.635,0.769	1.0,1.0	-0.298

10-12m_vs_8-10w	SE	Arl16	ENSMUSG00000057594.12	chr11	-	120465983	120466099	120465437	120465821	120466653	120466732	28593	14,18	1,5	31,35	0,0	264	149	7.47903622818e-05	0.00872487107065	0.888,0.67	1.0,1.0	-0.221
10-12m_vs_8-10w	SE	Cldn6	ENSMUSG00000023906.2	chr17	+	23679757	23679821	23679364	23679407	23681047	23682446	28716	2,13	3,6	4,5	0,0	212	149	1.90017882951e-06	0.00069111201667	0.319,0.604	1.0,1.0	-0.539
10-12m_vs_8-10w	SE	Armc9	ENSMUSG00000062590.13	chr1	+	86218847	86218898	86213049	86213105	86244565	86244670	28731	0,0	2,3	1,3	1,0	199	149	3.39655909309e-06	0.00101001013581	0.0,0.0	0.428,1.0	-0.714
10-12m_vs_8-10w	SE	Armc9	ENSMUSG00000062590.13	chr1	+	86252276	86252392	86244565	86244670	86257225	86257362	28732	0,0	1,2	9,2	6,1	264	149	1.34442979607e-05	0.00273677182179	0.0,0.0	0.458,0.53	-0.494
10-12m_vs_8-10w	SE	Tnfrsf13b	ENSMUSG00000010142.12	chr11	+	61146840	61147017	61141260	61141491	61147415	61147642	28892	10,19	0,0	12,8	5,1	298	149	1.98690251657e-05	0.00348351099596	1.0,1.0	0.545,0.8	0.327
10-12m_vs_8-10w	SE	Ccl25	ENSMUSG00000023235.14	chr8	+	4347713	4348340	4327756	4327893	4348502	4348788	28906	0,0	1,2	3,1	0,1	298	149	4.1029523674e-05	0.00573486858321	0.0,0.0	1.0,0.333	-0.667
10-12m_vs_8-10w	SE	RP23-95L9.5	ENSMUSG00000113204.1	chr13	-	74611263	74611321	74610199	74610326	74612723	74612948	29014	1,4	1,0	2,0	4,2	206	149	0.000113042328766	0.0117824291196	0.42,1.0	0.266,0.0	0.577
10-12m_vs_8-10w	SE	RP23-95L9.5	ENSMUSG00000113204.1	chr13	-	74611263	74611387	74610199	74610326	74612723	74612948	29016	1,6	1,0	1,2	4,2	272	149	0.000584638338963	0.0352538080698	0.354,1.0	0.12,0.354	0.44
10-12m_vs_8-10w	SE	Timm10	ENSMUSG00000027076.10	chr2	+	84827598	84827713	84826996	84827147	84829789	84830113	29116	389,438	19,10	673,319	5,6	263	149	0.000717959034003	0.0405519840975	0.921,0.961	0.987,0.968	-0.036
10-12m_vs_8-10w	SE	Oas1h	ENSMUSG00000001168.16	chr5	+	120870918	120871154	120866983	120867180	120871789	120871943	29264	22,41	4,2	23,26	0,0	298	149	0.000524093151269	0.0329798119733	0.733,0.911	1.0,1.0	-0.178
10-12m_vs_8-10w	SE	Atf7ip2	ENSMUSG00000039200.16	chr16	+	10209133	10209268	10204603	10204722	10211004	10211103	29339	2046,2520	17,11	2701,2445	42,36	283	149	7.43823828917e-05	0.00871078013702	0.984,0.992	0.971,0.973	0.016
10-12m_vs_8-10w	SE	Atf7ip2	ENSMUSG00000039200.16	chr16	+	10240560	10240646	10237165	10237232	10247444	10247597	29346	105,119	14,25	188,144	5,4	234	149	4.78839209395e-08	4.03435335004e-05	0.827,0.752	0.96,0.958	-0.169
10-12m_vs_8-10w	SE	Nipal1	ENSMUSG00000067219.9	chr5	+	72658681	72658948	72647794	72647933	72663533	72663624	29367	8,8	0,0	21,13	3,3	298	149	0.00014302461382	0.013993805038	1.0,1.0	0.778,0.684	0.269
10-12m_vs_8-10w	SE	Dus2	ENSMUSG00000031901.12	chr8	+	106048652	106048774	106045900	106046069	106050316	106050466	29565	20,31	4,5	42,40	0,1	270	149	5.05595935558e-05	0.00655351723137	0.734,0.774	1.0,0.957	-0.224
10-12m_vs_8-10w	SE	Dus2	ENSMUSG00000031901.12	chr8	+	106053005	106053079	106051801	106051889	106053291	106053819	29572	19,33	3,3	54,36	0,0	222	149	0.000659916749656	0.0382713861067	0.81,0.881	1.0,1.0	-0.154
10-12m_vs_8-10w	SE	Primpol	ENSMUSG00000038225.15	chr8	-	46599718	46599866	46592592	46593703	46605084	46605214	29659	13,47	1,0	35,13	7,1	296	149	0.000408337847969	0.0281483983335	0.867,1.0	0.716,0.867	0.142
10-12m_vs_8-10w	SE	RP23-266O23.1	ENSMUSG00000038925.13	chr14	+	24296008	24296138	24293283	24293307	24297981	24298052	29698	2,3	0,0	6,3	2,2	278	149	0.000340180534925	0.0248082469884	1.0,1.0	0.617,0.446	0.469
10-12m_vs_8-10w	SE	Slc25a19	ENSMUSG00000020744.13	chr11	-	115624198	115624368	115623411	115624027	115628064	115628093	29955	4,12	0,1	1,3	2,4	298	149	5.05966555986e-06	0.0013531253808	1.0,0.857	0.2,0.273	0.692
10-12m_vs_8-10w	SE	Dnmt3l	ENSMUSG00000000730.13	chr10	+	78056411	78056750	78055334	78055716	78057267	78057407	30159	0,0	1,3	7,1	5,4	298	149	0.000154008856757	0.0145521577392	0.0,0.0	0.412,0.111	-0.262
10-12m_vs_8-10w	SE	Wdr18	ENSMUSG00000035754.8	chr10	+	79966559	79966726	79961001	79961112	79967319	79967388	30506	28,42	0,0	50,41	2,6	298	149	0.000217552266085	0.0187993669021	1.0,1.0	0.926,0.774	0.15
10-12m_vs_8-10w	SE	Map4k5	ENSMUSG00000034761.15	chr12	-	69820939	69820942	69818383	69818498	69822802	69822863	30530	74,114	0,1	81,98	6,6	151	149	0.000571181675396	0.0345798630667	1.0,0.991	0.93,0.942	0.06

10-12m_vs_8-10w	SE	Traf7	ENSMUSG00000052752.15	chr17	-	24516504	24516599	24514124	24514217	24518741	24518794	30582	1,2	0,0	1,0	1,1	243	149	0.000514089794671	0.0327509398812	1.0,1.0	0.38,0.0	0.81
10-12m_vs_8-10w	SE	Pcmt1	ENSMUSG00000019795.17	chr10	-	7638008	7638225	7629380	7630976	7640694	7640815	30669	3,5	0,1	6,3	9,1	298	149	0.000507253468197	0.032390536724	1.0,0.714	0.25,0.6	0.432
10-12m_vs_8-10w	SE	Slc25a40	ENSMUSG00000054099.11	chr5	+	8424796	8424887	8422872	8423039	8427195	8427267	30717	5,5	1,0	1,1	1,2	239	149	0.000234833342865	0.0200076688833	0.757,1.0	0.384,0.238	0.568
10-12m_vs_8-10w	SE	Tma7	ENSMUSG00000091537.2	chr9	-	109082170	109082226	109082001	109082089	109082326	109082587	30992	826,542	116,83	899,645	65,67	204	149	0.000614456568489	0.0361183763156	0.839,0.827	0.91,0.875	-0.06
10-12m_vs_8-10w	SE	Ddx43	ENSMUSG00000070291.4	chr9	+	78413742	78413884	78412965	78413076	78414223	78414324	31023	32,38	4,4	35,35	12,19	290	149	0.000752242840958	0.0420189274569	0.804,0.83	0.6,0.486	0.274
10-12m_vs_8-10w	SE	Ddx43	ENSMUSG00000070291.4	chr9	+	78418036	78418164	78414223	78414324	78420588	78420676	31025	20,10	2,3	18,23	0,0	276	149	0.00059067418391	0.035414553526	0.844,0.643	1.0,1.0	-0.256
10-12m_vs_8-10w	SE	Vps16	ENSMUSG00000027411.17	chr2	+	130438096	130438225	130437914	130438012	130438306	130438451	31070	5,12	1,4	13,4	0,0	277	149	0.000130875206154	0.0131095801458	0.729,0.617	1.0,1.0	-0.327
10-12m_vs_8-10w	SE	Zc3h14	ENSMUSG00000021012.16	chr12	+	98779107	98779267	98763822	98763978	98783581	98783702	31308	3,6	5,7	1,0	3,9	298	149	0.00047155117991	0.0308911853949	0.231,0.3	0.143,0.0	0.194
10-12m_vs_8-10w	SE	Slc26a8	ENSMUSG00000036196.15	chr17	-	28661864	28662014	28659592	28659675	28669902	28670084	31397	5,13	1,2	2,1	5,1	298	149	0.000468227013453	0.0307725492525	0.714,0.765	0.167,0.333	0.49
10-12m_vs_8-10w	SE	Ptma	ENSMUSG00000026238.14	chr1	+	86529455	86529552	86529172	86529244	86529964	86530712	31492	72,165	1,1	147,79	12,2	245	149	0.000223707425823	0.0191221713775	0.978,0.99	0.882,0.96	0.063
10-12m_vs_8-10w	SE	Hsf1	ENSMUSG00000022556.11	chr15	+	76496835	76496960	76496452	76496589	76497618	76497694	31587	21,70	0,0	25,21	1,6	273	149	6.84158400497e-05	0.00807440017334	1.0,1.0	0.932,0.656	0.206
10-12m_vs_8-10w	SE	Gtf2f2	ENSMUSG00000067995.4	chr14	-	75949437	75949519	75906782	75906926	76010679	76010865	31737	1,1	0,0	5,4	3,1	230	149	0.000771807439762	0.0424858284127	1.0,1.0	0.519,0.722	0.379
10-12m_vs_8-10w	SE	Gtf2f2	ENSMUSG00000067995.4	chr14	-	75995420	75995565	75906782	75906926	76010679	76010865	31742	5,8	0,0	7,0	3,1	293	149	0.000269390244478	0.0216290635501	1.0,1.0	0.543,0.0	0.728
10-12m_vs_8-10w	SE	Dph7	ENSMUSG00000026975.10	chr2	+	24969505	24969678	24969049	24969115	24971522	24972161	31785	71,88	10,16	137,76	4,3	298	149	3.39544742712e-05	0.00525445489347	0.78,0.733	0.945,0.927	-0.18
10-12m_vs_8-10w	SE	Phkg1	ENSMUSG00000025537.12	chr5	-	129869460	129869526	129866861	129867025	129873668	129873847	31851	23,25	10,12	46,34	5,3	214	149	0.000259393867327	0.0210365652136	0.616,0.592	0.865,0.888	-0.273
10-12m_vs_8-10w	SE	Heatr5b	ENSMUSG00000039414.9	chr17	-	78761999	78762266	78760415	78760640	78762932	78763077	31872	8,27	4,1	20,18	0,0	298	149	0.000954346407445	0.0484862326369	0.5,0.931	1.0,1.0	-0.285
10-12m_vs_8-10w	SE	Pard3	ENSMUSG00000025812.17	chr8	+	127410731	127410773	127409555	127409707	127415569	127415797	32067	34,48	0,0	60,50	1,8	190	149	0.000613570400231	0.0361183763156	1.0,1.0	0.979,0.831	0.095
10-12m_vs_8-10w	SE	Ceacam1	ENSMUSG00000074272.10	chr7	-	25465467	25465520	25464848	25464880	25471797	25472073	32158	2,5	2,0	0,0	3,7	201	149	3.46367404536e-07	0.0001923731638	0.426,1.0	0.0,0.0	0.713
10-12m_vs_8-10w	SE	Ceacam1	ENSMUSG00000074272.10	chr7	-	25466316	25466437	25465467	25465520	25471797	25472073	32160	1,6	2,3	3,3	0,0	269	149	3.43202655906e-06	0.00101064852003	0.217,0.526	1.0,1.0	-0.629
10-12m_vs_8-10w	SE	Utp20	ENSMUSG00000004356.8	chr10	-	88751344	88751532	88750651	88750723	88752906	88753039	32271	25,42	3,4	32,43	0,0	298	149	0.00025830634595	0.0210136957256	0.806,0.84	1.0,1.0	-0.177
10-12m_vs_8-10w	SE	Mtmr2	ENSMUSG00000031918.16	chr9	+	13783605	13783710	13782276	13782382	13785851	13785927	32351	22,21	23,29	38,34	16,6	253	149	0.000144313426505	0.0140165553957	0.36,0.299	0.583,0.769	-0.346

10-12m_vs_8-10w	SE	Xlr3a	ENSMUSG00000057836.12	chrX	-	73094960	73095082	73093269	73093304	73096980	73097095	32453	22,33	1,5	26,35	0,0	270	149	0.000554369417391	0.0342953140469	0.924,0.785	1.0,1.0	-0.145
10-12m_vs_8-10w	SE	Map3k3	ENSMUSG00000020700.11	chr11	+	106151008	106151140	106150269	106150418	106151617	106151747	32504	3,5	0,0	2,0	2,1	280	149	8.36831493978e-05	0.00936602806046	1.0,1.0	0.347,0.0	0.827
10-12m_vs_8-10w	SE	Rnmt	ENSMUSG00000009535.13	chr18	+	68313987	68314152	68313664	68313846	68317993	68318111	32618	299,439	0,0	427,274	14,4	298	149	3.13452681321e-05	0.00495173608185	1.0,1.0	0.938,0.972	0.045
10-12m_vs_8-10w	SE	Sec11a	ENSMUSG00000025724.12	chr7	-	80927736	80927886	80920894	80923220	80947401	80947511	32650	231,262	2,0	272,279	15,5	298	149	3.31132201689e-05	0.00517709835538	0.983,1.0	0.901,0.965	0.059
10-12m_vs_8-10w	SE	Sec11a	ENSMUSG00000025724.12	chr7	-	80935026	80935136	80920894	80923220	80947401	80947511	32652	72,93	2,0	92,103	15,5	258	149	2.93922503869e-05	0.00471691188616	0.954,1.0	0.78,0.922	0.126
10-12m_vs_8-10w	SE	Fam135a	ENSMUSG00000026153.15	chr1	-	24086519	24086611	24067898	24067978	24100060	24100194	32667	11,12	0,0	12,11	2,4	240	149	3.7984326558e-05	0.00545517696261	1.0,1.0	0.788,0.631	0.291
10-12m_vs_8-10w	SE	Immt	ENSMUSG00000052337.15	chr6	+	71853172	71853268	71852729	71852867	71856911	71857048	32716	31,29	6,14	14,17	16,23	244	149	0.000804331347737	0.0436442813064	0.759,0.558	0.348,0.311	0.329
10-12m_vs_8-10w	SE	Scrn3	ENSMUSG00000008226.14	chr2	+	73321984	73322194	73319302	73319499	73329762	73329802	32756	14,26	1,0	34,18	4,7	298	149	0.000217051568927	0.0187993669021	0.875,1.0	0.81,0.563	0.251
10-12m_vs_8-10w	SE	Hdlbp	ENSMUSG00000034088.15	chr1	-	93461343	93461599	93442185	93442250	93478712	93478815	32863	4,8	23,38	0,1	26,23	298	149	0.00061276236881	0.0361183763156	0.08,0.095	0.0,0.021	0.077
10-12m_vs_8-10w	SE	Rpf2	ENSMUSG00000038510.12	chr10	-	40239747	40239787	40233107	40233207	40244584	40244717	32930	5,1	3,2	9,10	1,0	188	149	0.000204754052678	0.0181590502099	0.569,0.284	0.877,1.0	-0.512
10-12m_vs_8-10w	SE	Eef1akmt1	ENSMUSG00000021951.6	chr14	-	57558033	57558116	57550361	57550642	57565921	57566084	33115	97,55	20,15	129,102	9,3	231	149	6.15000671389e-06	0.0015165516556	0.758,0.703	0.902,0.956	-0.199
10-12m_vs_8-10w	SE	Cdk1	ENSMUSG00000019942.13	chr10	-	69345060	69345184	69340714	69340878	69350095	69350158	33133	1,0	1,3	4,6	2,0	272	149	4.1463778215e-05	0.00576898099559	0.354,0.0	0.523,1.0	-0.585
10-12m_vs_8-10w	SE	Drg1	ENSMUSG00000020457.13	chr11	-	3256542	3256712	3254410	3254642	3259305	3259375	33241	272,314	2,2	453,407	13,14	298	149	0.000182664735411	0.0166378501194	0.986,0.987	0.946,0.936	0.045
10-12m_vs_8-10w	SE	Tpd52l2	ENSMUSG00000000827.18	chr2	+	181511573	181511615	181508165	181508267	181515079	181515387	33292	35,46	15,17	61,43	7,1	190	149	1.82388891637e-05	0.00327339495399	0.647,0.68	0.872,0.971	-0.258
10-12m_vs_8-10w	SE	Tpd52l2	ENSMUSG00000000827.18	chr2	+	181513038	181513087	181508165	181508267	181515079	181515115	33294	579,868	15,17	867,682	7,1	197	149	0.000193820262241	0.017444398736	0.967,0.975	0.989,0.998	-0.023
10-12m_vs_8-10w	SE	AC122423.2	ENSMUSG00000030924.16	chr7	+	119843241	119843334	119834263	119834401	119843847	119844064	33379	5,3	4,5	9,7	0,1	241	149	1.23192934768e-05	0.00257694131341	0.436,0.271	1.0,0.812	-0.553
10-12m_vs_8-10w	SE	Nasp	ENSMUSG00000028693.15	chr4	-	116610402	116611377	116605683	116605763	116611971	116612081	33572	152,290	184,223	171,175	523,314	298	149	1.14123304029e-07	7.21140403022e-05	0.292,0.394	0.141,0.218	0.163
10-12m_vs_8-10w	SE	Thoc2	ENSMUSG00000037475.15	chrX	-	41873881	41873952	41872289	41872423	41879885	41879937	33632	45,62	2,6	54,35	0,0	219	149	0.000935475671288	0.0480100043754	0.939,0.875	1.0,1.0	-0.093
10-12m_vs_8-10w	SE	Thoc2	ENSMUSG00000037475.15	chrX	-	41881904	41882000	41879885	41879937	41897791	41897846	33638	69,73	0,0	49,36	2,5	244	149	0.000487870215641	0.0316864914574	1.0,1.0	0.937,0.815	0.124
10-12m_vs_8-10w	SE	Ntmt1	ENSMUSG00000026857.9	chr2	+	30815358	30815498	30807941	30808083	30819628	30819832	33744	12,19	21,36	13,6	66,49	288	149	0.000446012892966	0.0297319056188	0.228,0.214	0.092,0.06	0.145
10-12m_vs_8-10w	SE	Mov10l1	ENSMUSG00000015365.15	chr15	+	88996055	88996196	88993805	88993918	88996668	88996895	33810	4,9	0,0	10,13	4,1	289	149	0.000294310773402	0.0229378525288	1.0,1.0	0.563,0.87	0.284

10-12m_vs_8-10w	SE	Isoc2b	ENSMUSG00000052605.12	chr7	-	4849449	4849567	4844958	4845365	4851392	4851533	33840	15,8	4,1	22,4	0,0	266	149	0.00035109060224	0.0253545929918	0.677,0.818	1.0,1.0	-0.252
10-12m_vs_8-10w	SE	Isoc2b	ENSMUSG00000052605.12	chr7	-	4851023	4851233	4849449	4849567	4851392	4851533	33844	4,7	3,2	14,8	1,0	298	149	0.000394720176745	0.0277170800168	0.4,0.636	0.875,1.0	-0.42
10-12m_vs_8-10w	SE	Tarbp2	ENSMUSG00000023051.11	chr15	+	102519121	102519291	102518191	102518602	102520474	102520577	33854	2,12	0,0	4,0	4,4	298	149	2.80202305802e-10	1.21411659104e-06	1.0,1.0	0.333,0.0	0.834
10-12m_vs_8-10w	SE	Tarbp2	ENSMUSG00000023051.11	chr15	+	102520474	102520577	102518454	102518602	102521137	102521213	33859	0,0	1,4	4,1	1,0	251	149	1.09504251666e-07	7.06675203677e-05	0.0,0.0	0.704,1.0	-0.852
10-12m_vs_8-10w	SE	Pdss1	ENSMUSG00000026784.14	chr2	+	22899918	22899983	22895618	22895762	22901280	22901345	33898	0,0	8,11	2,6	19,11	213	149	0.000368278332373	0.0262829414099	0.0,0.0	0.069,0.276	-0.173
10-12m_vs_8-10w	SE	Hnrnpd	ENSMUSG00000000568.15	chr5	-	99976533	99976590	99967218	99967387	99978391	99978449	34028	6,6	0,1	24,4	9,7	205	149	0.000241462488127	0.0204005535582	1.0,0.813	0.66,0.293	0.43
10-12m_vs_8-10w	SE	RP23-100C5.2	ENSMUSG00000060029.13	chr2	-	130552352	130552435	130552138	130552242	130552841	130552916	34123	0,4	2,2	4,1	1,0	231	149	0.000870096587906	0.0458175340413	0.0,0.563	0.721,1.0	-0.579
10-12m_vs_8-10w	SE	Trappc6b	ENSMUSG00000020993.9	chr12	-	59050267	59050385	59048122	59048206	59061155	59061421	34162	67,110	0,1	78,57	9,2	266	149	7.89425305304e-05	0.00907412341473	1.0,0.984	0.829,0.941	0.107
10-12m_vs_8-10w	SE	Zfand2b	ENSMUSG00000026197.12	chr1	+	75170685	75170753	75170538	75170599	75170975	75171048	34167	6,9	0,0	6,4	2,2	216	149	0.000868132457219	0.0458175340413	1.0,1.0	0.674,0.58	0.373
10-12m_vs_8-10w	SE	Sat2	ENSMUSG00000069835.10	chr11	+	69622859	69622961	69622706	69622790	69623111	69623152	34188	35,64	6,3	75,43	0,0	250	149	7.69730215989e-05	0.00894509087401	0.777,0.927	1.0,1.0	-0.148
10-12m_vs_8-10w	SE	Tmem258	ENSMUSG00000036372.14	chr19	+	10205749	10206124	10204013	10204264	10206423	10206533	34291	7,8	41,51	1,0	39,23	298	149	0.000322750246019	0.0240524268108	0.079,0.073	0.013,0.0	0.069
10-12m_vs_8-10w	SE	RP23-423J10.4	ENSMUSG00000105153.4	chr3	-	34700818	34700942	34699639	34699677	34701376	34701499	34384	46,66	3,9	40,28	0,1	272	149	0.000367170446205	0.0262656764242	0.894,0.801	1.0,0.939	-0.122
10-12m_vs_8-10w	SE	Vps26a	ENSMUSG00000020078.16	chr10	-	62480557	62480707	62468289	62468454	62486657	62486732	34672	71,169	2,8	103,108	0,0	298	149	0.000515057146924	0.0327509398812	0.947,0.914	1.0,1.0	-0.07
10-12m_vs_8-10w	SE	Zwilch	ENSMUSG00000032400.17	chr9	-	64144079	64144192	64139890	64140016	64146824	64146920	34699	95,110	1,1	152,146	9,7	261	149	0.000547736015491	0.0339841911923	0.982,0.984	0.906,0.923	0.068
10-12m_vs_8-10w	SE	Zwilch	ENSMUSG00000032400.17	chr9	-	64149265	64149334	64147269	64147406	64150155	64150268	34708	3,0	2,4	7,9	0,0	217	149	5.24704908411e-08	4.30130393973e-05	0.507,0.0	1.0,1.0	-0.746
10-12m_vs_8-10w	SE	Rrnad1	ENSMUSG00000004896.16	chr3	-	87927562	87927715	87927054	87927117	87927923	87928048	34754	1,1	1,2	1,3	0,0	298	149	9.51090442243e-05	0.0103396144099	0.333,0.2	1.0,1.0	-0.734
10-12m_vs_8-10w	SE	Rrnad1	ENSMUSG00000004896.16	chr3	-	87927562	87927755	87927054	87927117	87927923	87928048	34756	2,0	1,2	1,2	0,0	298	149	5.15161022406e-05	0.00662481628967	0.5,0.0	1.0,1.0	-0.75
10-12m_vs_8-10w	SE	Dimt1	ENSMUSG00000021692.9	chr13	+	106954675	106954739	106953433	106953498	106957093	106957200	34889	359,527	10,6	602,422	40,22	212	149	8.19412001274e-06	0.00186869063238	0.962,0.984	0.914,0.931	0.05
10-12m_vs_8-10w	SE	Hnrnpa3	ENSMUSG00000059005.13	chr2	+	75663570	75663714	75662660	75662756	75665027	75665150	34912	38,50	1,8	39,33	0,1	292	149	0.000962612344225	0.0487010406123	0.951,0.761	1.0,0.944	-0.116
10-12m_vs_8-10w	SE	Rad1	ENSMUSG00000022248.14	chr15	+	10487989	10488098	10486048	10486716	10490255	10490347	34961	88,122	0,3	134,95	7,10	257	149	0.000468725652126	0.0307725492525	1.0,0.959	0.917,0.846	0.098
10-12m_vs_8-10w	SE	Phf7	ENSMUSG00000021902.7	chr14	-	31238336	31238482	31237688	31238153	31239630	31239737	35113	294,353	34,14	245,237	46,43	294	149	7.77373821276e-05	0.00899943716531	0.814,0.927	0.73,0.736	0.138

10-12m_vs_8-10w	SE	Nol11	ENSMUSG00000018433.14	chr11	-	107183572	107183630	107181738	107181883	107186634	107186691	35230	20,27	1,0	39,19	3,7	206	149	0.000426305149037	0.0289678928642	0.935,1.0	0.904,0.663	0.184
10-12m_vs_8-10w	SE	Fbxo34	ENSMUSG00000037536.14	chr14	+	47509056	47509229	47507264	47507446	47526204	47526314	35326	16,11	16,10	3,1	18,10	298	149	3.69778571945e-05	0.00545517696261	0.333,0.355	0.077,0.048	0.282
10-12m_vs_8-10w	SE	Ranbp17	ENSMUSG00000040594.19	chr11	-	33404037	33404199	33329470	33329544	33441802	33441938	35420	2,1	10,8	13,4	10,5	298	149	0.000708816759377	0.0401852731377	0.091,0.059	0.394,0.286	-0.265
10-12m_vs_8-10w	SE	Ranbp17	ENSMUSG00000040594.19	chr11	-	33404037	33404199	33337854	33337914	33441802	33441938	35421	1,0	1,2	6,2	2,1	298	149	0.000604632028862	0.0358185431004	0.333,0.0	0.6,0.5	-0.384
10-12m_vs_8-10w	SE	Akap11	ENSMUSG00000022016.16	chr14	-	78502938	78503022	78502189	78502236	78507724	78507880	35580	0,0	11,6	2,10	9,9	232	149	1.90235734518e-05	0.00339414121392	0.0,0.0	0.125,0.416	-0.27
10-12m_vs_8-10w	SE	Mpzl1	ENSMUSG00000026566.15	chr1	-	165601749	165601855	165592239	165593677	165605608	165605822	35644	4,9	4,8	9,8	1,1	254	149	0.000953477739331	0.0484862326369	0.37,0.398	0.841,0.824	-0.449
10-12m_vs_8-10w	SE	Tph2	ENSMUSG00000006764.8	chr10	-	115174766	115174933	115174079	115174119	115175472	115175558	35663	19,7	0,1	14,8	5,6	298	149	3.90139948885e-05	0.00555555623926	1.0,0.778	0.583,0.4	0.398
10-12m_vs_8-10w	SE	Hltf	ENSMUSG00000002428.12	chr3	+	20065321	20065419	20064565	20064699	20067345	20067420	35719	8,19	8,3	4,18	0,0	246	149	6.24164203156e-06	0.00152673584241	0.377,0.793	1.0,1.0	-0.415
10-12m_vs_8-10w	SE	Trmt11	ENSMUSG00000019792.8	chr10	-	30567760	30567871	30566425	30566506	30587438	30587595	35856	15,15	233,316	4,2	214,194	259	149	0.000926672032422	0.0477196764268	0.036,0.027	0.011,0.006	0.023
10-12m_vs_8-10w	SE	Srm	ENSMUSG00000006442.10	chr4	+	148592481	148592574	148591917	148592164	148593287	148593441	36022	14,40	3,4	25,20	15,12	241	149	0.000421720026819	0.028808986787	0.743,0.861	0.507,0.507	0.295
10-12m_vs_8-10w	SE	Rhebl1	ENSMUSG00000023755.10	chr15	-	98879007	98879055	98878452	98878534	98879258	98879315	36063	111,158	26,42	209,161	14,19	196	149	1.40855045766e-05	0.00283766310975	0.764,0.741	0.919,0.866	-0.14
10-12m_vs_8-10w	SE	Rhebl1	ENSMUSG00000023755.10	chr15	-	98879404	98879487	98879258	98879315	98879764	98879844	36065	75,124	4,8	122,96	0,1	231	149	0.000193541649325	0.017444398736	0.924,0.909	1.0,0.984	-0.075
10-12m_vs_8-10w	SE	Atf2	ENSMUSG00000027104.18	chr2	-	73823200	73823306	73816508	73819148	73842359	73842446	36146	151,252	1,0	267,248	15,9	254	149	2.57024513117e-06	0.000866201167484	0.989,1.0	0.913,0.942	0.067
10-12m_vs_8-10w	SE	Atf2	ENSMUSG00000027104.18	chr2	-	73828471	73828678	73816508	73819148	73842359	73842446	36150	24,21	1,0	18,15	15,9	298	149	1.37344347007e-09	3.78708308097e-06	0.923,1.0	0.375,0.455	0.547
10-12m_vs_8-10w	SE	Atf2	ENSMUSG00000027104.18	chr2	-	73842359	73842446	73816508	73819148	73863168	73863238	36154	1,0	10,10	7,6	3,6	235	149	1.06393026311e-06	0.000481642818066	0.06,0.0	0.597,0.388	-0.463
10-12m_vs_8-10w	SE	Atf2	ENSMUSG00000027104.18	chr2	-	73880284	73880379	73850863	73850982	73892474	73892614	36190	3,3	0,1	2,1	2,1	243	149	0.000805802562777	0.0436442813064	1.0,0.648	0.38,0.38	0.444
10-12m_vs_8-10w	SE	RP23-48M16.9	ENSMUSG00000072574.4	chr14	-	44478891	44479054	44472515	44473299	44535034	44535359	36336	10,14	4,1	11,19	0,0	298	149	0.000303322165404	0.0232324863608	0.556,0.875	1.0,1.0	-0.285
10-12m_vs_8-10w	SE	Eed	ENSMUSG00000030619.9	chr7	-	89964728	89964862	89956254	89956413	89969570	89969652	36425	3,2	0,0	5,2	2,3	282	149	2.61847192928e-06	0.000872756836121	1.0,1.0	0.569,0.26	0.586
10-12m_vs_8-10w	SE	Cers6	ENSMUSG00000027035.10	chr2	+	69105742	69105766	69105002	69105159	69108441	69108644	36456	3,3	15,13	0,0	15,18	172	149	0.000992131731933	0.0496569650624	0.148,0.167	0.0,0.0	0.158
10-12m_vs_8-10w	SE	Rsbn1l	ENSMUSG00000039968.9	chr5	-	20905779	20905922	20902287	20902455	20908106	20908244	36470	22,27	9,4	34,40	1,2	291	149	0.000919915110381	0.0476142409778	0.556,0.776	0.946,0.911	-0.262
10-12m_vs_8-10w	SE	RP23-167L1.2	ENSMUSG00000033255.8	chr10	+	75992394	75992530	75985919	75986140	75993824	75993932	36584	11,19	4,3	33,14	1,0	284	149	0.000177900368111	0.0163018008011	0.591,0.769	0.945,1.0	-0.293

10-12m_vs_8-10w	SE	Usp33	ENSMUSG00000025437.15	chr3	+	152389740	152389831	152380513	152380640	152391649	152391718	36807	1,1	0,0	0,1	1,1	239	149	0.000541660784368	0.033804759775	1.0,1.0	0.0,0.384	0.808
10-12m_vs_8-10w	SE	Usp33	ENSMUSG00000025437.15	chr3	+	152389740	152389831	152382910	152382992	152391649	152391718	36808	4,1	1,1	0,1	4,4	239	149	0.000257465876158	0.0210136957256	0.714,0.384	0.0,0.135	0.481
10-12m_vs_8-10w	SE	Macf1	ENSMUSG00000028649.18	chr4	-	123465858	123466074	123465355	123465505	123469722	123469872	36820	9,7	2,3	8,19	0,0	298	149	5.7072318476e-05	0.00715314252767	0.692,0.538	1.0,1.0	-0.385
10-12m_vs_8-10w	SE	Cenpi	ENSMUSG00000031262.12	chrX	+	134347224	134347360	134343075	134343170	134349159	134349284	36921	7,25	0,1	18,26	2,7	284	149	0.000536710859985	0.0335649012252	1.0,0.929	0.825,0.661	0.222
10-12m_vs_8-10w	SE	Sp100	ENSMUSG00000026222.16	chr1	+	85681085	85681139	85679058	85679109	85694258	85694309	36965	1,1	1,3	3,1	0,0	202	149	4.2363621261e-06	0.0011896756151	0.425,0.197	1.0,1.0	-0.689
10-12m_vs_8-10w	SE	RP23-262M12.1	ENSMUSG00000101344.1	chr1	-	73825886	73825924	73823898	73823995	73841807	73841917	37058	1,1	3,3	7,1	0,0	186	149	1.61257956099e-07	9.78223013288e-05	0.211,0.211	1.0,1.0	-0.789
10-12m_vs_8-10w	SE	RP23-262M12.1	ENSMUSG00000101344.1	chr1	-	73829785	73829966	73823898	73823995	73841807	73841917	37059	14,13	3,3	2,13	0,0	298	149	0.000147346999324	0.0141736974657	0.7,0.684	1.0,1.0	-0.308
10-12m_vs_8-10w	SE	Atp6v0d1	ENSMUSG00000013160.7	chr8	-	105530814	105530993	105525623	105525701	105539320	105539492	37075	13,44	0,1	26,25	3,4	298	149	0.000906114533662	0.0472222679046	1.0,0.957	0.813,0.758	0.193
10-12m_vs_8-10w	SE	AC145199.4	ENSMUSG00000085601.7	chr7	-	19104763	19105000	19103689	19103783	19105720	19105846	37099	17,22	2,4	25,12	0,0	298	149	0.000246808871174	0.020565823823	0.81,0.733	1.0,1.0	-0.228
10-12m_vs_8-10w	SE	Mpv17l	ENSMUSG00000022679.13	chr16	+	13942119	13942173	13940694	13941023	13944665	13944736	37151	1,4	3,1	2,2	0,0	202	149	0.000381177509494	0.0270128388796	0.197,0.747	1.0,1.0	-0.528
10-12m_vs_8-10w	SE	Hnrnpa1	ENSMUSG00000046434.16	chr15	+	103244255	103245966	103243958	103244028	103246345	103246421	37167	212,344	140,200	221,187	240,189	298	149	0.000133810562597	0.0132634254057	0.431,0.462	0.315,0.331	0.124
10-12m_vs_8-10w	SE	Noxo1	ENSMUSG00000019320.13	chr17	+	24699897	24700015	24699543	24699657	24700120	24700313	37485	8,3	12,16	0,2	19,19	266	149	0.00076312126841	0.042241544786	0.272,0.095	0.0,0.056	0.156
10-12m_vs_8-10w	SE	Mad2l2	ENSMUSG00000029003.11	chr4	+	148140914	148141033	148140181	148140834	148142985	148143057	37605	87,189	15,21	174,129	8,3	267	149	8.70538140054e-05	0.00960156084581	0.764,0.834	0.924,0.96	-0.143
10-12m_vs_8-10w	SE	Dst	ENSMUSG00000026131.18	chr1	+	34177795	34180498	34174696	34174795	34181134	34183904	37735	5,6	4,1	10,13	0,1	298	149	0.000644589825281	0.037526015337	0.385,0.75	1.0,0.867	-0.366
10-12m_vs_8-10w	SE	Fyttd1	ENSMUSG00000022800.13	chr16	+	32898400	32898470	32895084	32895197	32905531	32908963	37748	0,0	1,2	2,4	0,5	218	149	5.52530005935e-05	0.00698282817084	0.0,0.0	1.0,0.353	-0.677
10-12m_vs_8-10w	SE	H2-K1	ENSMUSG00000061232.15	chr17	-	33996826	33996859	33996615	33996654	33997037	33997157	37763	36,24	5,7	27,43	2,0	181	149	0.000345142754909	0.0250106108024	0.856,0.738	0.917,1.0	-0.162
10-12m_vs_8-10w	SE	Ube2w	ENSMUSG00000025939.17	chr1	-	16581926	16582002	16543948	16544032	16602261	16602353	37877	9,13	0,0	13,4	6,0	224	149	0.000436571789858	0.0292957056597	1.0,1.0	0.59,1.0	0.205
10-12m_vs_8-10w	SE	Brwd1	ENSMUSG00000022914.15	chr16	-	96066024	96066185	96064749	96064971	96066495	96066594	37925	3,10	3,2	9,18	0,0	298	149	2.45267274475e-05	0.00417932679893	0.333,0.714	1.0,1.0	-0.477
10-12m_vs_8-10w	SE	Fmr1nb	ENSMUSG00000062170.12	chrX	+	68768955	68769093	68761933	68762231	68803604	68803693	38070	2,1	4,3	10,4	2,0	286	149	9.04028109406e-05	0.00986333690158	0.207,0.148	0.723,1.0	-0.684
10-12m_vs_8-10w	SE	Ccdc163	ENSMUSG00000028689.14	chr4	+	116709239	116709273	116708508	116709094	116709437	116709619	38293	1,1	0,0	1,1	4,2	182	149	3.24568446564e-08	2.89543692727e-05	1.0,1.0	0.17,0.29	0.77
10-12m_vs_8-10w	SE	Ccdc163	ENSMUSG00000028689.14	chr4	+	116709437	116709619	116708597	116709094	116710100	116710142	38297	0,0	1,1	5,2	0,0	298	149	2.30785168753e-09	5.38457304111e-06	0.0,0.0	1.0,1.0	-1.0

10-12m_vs_8-10w	SE	Tmem116	ENSMUSG00000029452.18	chr5	+	121482332	121482437	121467853	121467985	121487749	121487799	38418	4,2	3,2	3,4	1,0	253	149	0.000830643104501	0.0446706312103	0.44,0.371	0.639,1.0	-0.414
10-12m_vs_8-10w	SE	Mrpl2	ENSMUSG00000002767.13	chr17	+	46648658	46648769	46648471	46648587	46649034	46649108	38469	118,177	6,10	209,136	2,0	259	149	3.8129224756e-05	0.00545517696261	0.919,0.911	0.984,1.0	-0.077
10-12m_vs_8-10w	SE	Mrpl2	ENSMUSG00000002767.13	chr17	+	46648669	46648769	46648471	46648587	46649034	46649108	38470	46,61	6,10	99,46	2,0	248	149	5.31575010077e-06	0.00137805142142	0.822,0.786	0.967,1.0	-0.18
10-12m_vs_8-10w	SE	Zdhhc16	ENSMUSG00000025157.8	chr19	+	41941515	41941639	41940003	41940735	41941935	41942006	38495	23,33	6,1	30,17	0,0	272	149	0.000269552142097	0.0216290635501	0.677,0.948	1.0,1.0	-0.188
10-12m_vs_8-10w	SE	Brd8	ENSMUSG00000003778.14	chr18	-	34608417	34608627	34607774	34608055	34609804	34609949	38647	0,8	2,4	3,5	0,0	298	149	6.77859021914e-07	0.000337051508093	0.0,0.5	1.0,1.0	-0.75
10-12m_vs_8-10w	SE	Xpnpep3	ENSMUSG00000022401.12	chr15	+	81415846	81415994	81414431	81414548	81427275	81427538	38730	4,4	0,1	8,1	3,3	296	149	0.00100903021035	0.049932663762	1.0,0.668	0.573,0.144	0.476
10-12m_vs_8-10w	SE	Med27	ENSMUSG00000026799.15	chr2	+	29379933	29380004	29349845	29349990	29389812	29389873	38776	3,3	4,4	7,4	1,0	219	149	2.75015929709e-05	0.00450965464582	0.338,0.338	0.826,1.0	-0.575
10-12m_vs_8-10w	SE	Med27	ENSMUSG00000026799.15	chr2	+	29379933	29380004	29349845	29349990	29413387	29413518	38777	53,88	130,181	41,26	357,184	219	149	9.36706268106e-12	9.47041260597e-08	0.217,0.249	0.072,0.088	0.153
10-12m_vs_8-10w	SE	Med27	ENSMUSG00000026799.15	chr2	+	29389812	29389873	29349845	29349990	29413387	29413518	38784	12,27	130,181	10,12	357,184	209	149	0.00010971135488	0.0115176913004	0.062,0.096	0.02,0.044	0.047
10-12m_vs_8-10w	SE	Med27	ENSMUSG00000026799.15	chr2	+	29389812	29389873	29349845	29349990	29471292	29471386	38785	1,3	0,2	0,0	2,3	209	149	0.00026536539842	0.0214634610653	1.0,0.517	0.0,0.0	0.759
10-12m_vs_8-10w	SE	Slc4a11	ENSMUSG00000074796.10	chr2	-	130692028	130692078	130691548	130691780	130696040	130696103	38818	0,0	2,1	1,1	0,0	198	149	9.18933851235e-09	1.39360913209e-05	0.0,0.0	1.0,1.0	-1.0
10-12m_vs_8-10w	SE	Clybl	ENSMUSG00000025545.10	chr14	+	122371240	122371429	122311243	122311430	122401946	122402227	38902	168,227	0,3	318,213	15,5	298	149	0.00039166262479	0.0276267885407	1.0,0.974	0.914,0.955	0.052
10-12m_vs_8-10w	SE	Clybl	ENSMUSG00000025545.10	chr14	+	122384197	122384322	122311243	122311430	122401946	122402227	38915	56,77	0,3	125,53	15,5	273	149	0.000434909220513	0.029248850482	1.0,0.933	0.82,0.853	0.13
10-12m_vs_8-10w	SE	Gabpb1	ENSMUSG00000027361.15	chr2	-	126646562	126646748	126627441	126630382	126676033	126676337	38929	5,5	7,3	0,0	1,5	298	149	2.23871484761e-05	0.00385809432062	0.263,0.455	0.0,0.0	0.359
10-12m_vs_8-10w	SE	Gabpb1	ENSMUSG00000027361.15	chr2	-	126653554	126653722	126646562	126646748	126676033	126676337	38956	18,11	4,4	31,26	0,0	298	149	1.80387827897e-06	0.000675474470117	0.692,0.579	1.0,1.0	-0.365
10-12m_vs_8-10w	SE	RP24-372K3.1	ENSMUSG00000096044.4	chr5	-	93485074	93485388	93481828	93482833	93493569	93493701	39038	9,28	0,0	34,18	1,5	298	149	0.000309546373043	0.0235900779919	1.0,1.0	0.944,0.643	0.207
10-12m_vs_8-10w	SE	Taf1b	ENSMUSG00000059669.8	chr12	+	24504946	24505034	24500435	24500534	24509453	24509549	39234	26,43	2,0	26,30	4,11	236	149	5.83300914352e-05	0.00725086066935	0.891,1.0	0.804,0.633	0.227
10-12m_vs_8-10w	SE	Taf1b	ENSMUSG00000059669.8	chr12	+	24544270	24544418	24516858	24517009	24547569	24547616	39241	6,8	0,0	8,10	3,3	296	149	3.71058777736e-05	0.00545517696261	1.0,1.0	0.573,0.627	0.4
10-12m_vs_8-10w	SE	RP23-250A14.2	ENSMUSG00000087174.7	chrX	-	105043129	105043304	105042603	105042817	105044001	105044111	39566	3,3	0,0	0,0	1,3	298	149	3.84015041988e-10	1.45594502982e-06	1.0,1.0	0.0,0.0	1.0
10-12m_vs_8-10w	SE	Hars2	ENSMUSG00000019143.15	chr18	+	36789156	36789284	36788165	36788264	36790133	36790250	39601	0,0	1,5	2,3	3,2	276	149	0.000319086539555	0.0239648385706	0.0,0.0	0.265,0.447	-0.356
10-12m_vs_8-10w	SE	Asl	ENSMUSG00000025533.15	chr5	-	130014678	130014731	130013962	130014025	130016096	130016174	39645	10,10	2,4	23,10	0,0	201	149	4.24169214976e-05	0.00582148256083	0.788,0.65	1.0,1.0	-0.281

10-12m_vs_8-10w	SE	1700086O06Rik	ENSMUSG00000097080.7	chr18	-	38240838	38240953	38239572	38239751	38249713	38250198	39697	2,2	5,4	7,4	1,0	263	149	3.15726768418e-06	0.000967303900292	0.185,0.221	0.799,1.0	-0.696
10-12m_vs_8-10w	SE	1700086O06Rik	ENSMUSG00000097080.7	chr18	-	38240838	38240953	38239572	38239751	38250177	38250565	39698	1,2	2,2	5,2	0,0	263	149	7.53851769608e-06	0.0017322028806	0.221,0.362	1.0,1.0	-0.709
10-12m_vs_8-10w	SE	Ulk2	ENSMUSG00000004798.14	chr11	-	61782005	61782124	61781591	61781749	61783536	61783715	39770	10,34	7,6	33,24	0,1	267	149	8.51361227694e-06	0.00189872333803	0.444,0.76	1.0,0.931	-0.364
10-12m_vs_8-10w	SE	RP23-18J12.6	ENSMUSG00000115121.1	chr14	-	122081872	122082087	122077322	122077513	122082689	122082833	39799	5,20	0,11	10,6	0,0	298	149	0.000203818786618	0.0181291132461	1.0,0.476	1.0,1.0	-0.262
10-12m_vs_8-10w	SE	Dtl	ENSMUSG00000037474.13	chr1	-	191558060	191558134	191556783	191556887	191561424	191561537	40030	18,45	4,17	44,40	2,4	222	149	0.000628000627467	0.0367213854947	0.751,0.64	0.937,0.87	-0.208
10-12m_vs_8-10w	SE	Ndufs1	ENSMUSG00000025968.16	chr1	-	63172153	63172257	63170848	63170914	63176585	63176669	40094	2,6	0,0	2,2	4,1	252	149	7.72878773536e-07	0.000372098191748	1.0,1.0	0.228,0.542	0.615
10-12m_vs_8-10w	SE	RP24-502J3.1	ENSMUSG00000110018.2	chr15	+	5580628	5581268	5539624	5539725	5587685	5587771	40107	3,1	2,2	5,2	0,0	298	149	1.3150306863e-05	0.00269994443327	0.429,0.2	1.0,1.0	-0.686
10-12m_vs_8-10w	SE	AC161108.3	ENSMUSG00000033488.11	chr1	+	157472187	157472416	157470615	157470797	157488442	157489060	40345	7,11	3,0	4,1	10,4	298	149	0.000146127313224	0.0141152469344	0.538,1.0	0.167,0.111	0.63
10-12m_vs_8-10w	SE	Strn3	ENSMUSG00000020954.16	chr12	-	51643103	51643214	51633578	51633719	51647993	51648135	40358	3,3	1,1	0,0	1,5	259	149	6.68457227337e-06	0.00159645481593	0.633,0.633	0.0,0.0	0.633
10-12m_vs_8-10w	SE	Gpx4	ENSMUSG00000075706.10	chr10	+	80054699	80054844	80054464	80054608	80054967	80055107	40448	377,593	45,89	708,377	47,35	293	149	0.000978623125952	0.0490621785674	0.81,0.772	0.885,0.846	-0.074
10-12m_vs_8-10w	SE	Cdk8	ENSMUSG00000029635.15	chr5	+	146286107	146286239	146271594	146271735	146296358	146296431	40506	1,7	0,0	3,5	4,3	280	149	9.14484089565e-08	6.76517485868e-05	1.0,1.0	0.285,0.47	0.623
10-12m_vs_8-10w	SE	Zfml	ENSMUSG00000030016.14	chr6	+	83958183	83958252	83954710	83954761	83959930	83959968	40520	15,17	0,0	14,16	0,7	217	149	0.000411030374497	0.0282511725554	1.0,1.0	1.0,0.611	0.195
10-12m_vs_8-10w	SE	Rock2	ENSMUSG00000020580.10	chr12	+	16958627	16958874	16958339	16958431	16959401	16959508	40541	69,61	0,0	52,71	10,0	298	149	0.000195748972712	0.0175658641755	1.0,1.0	0.722,1.0	0.139
10-12m_vs_8-10w	SE	Nid2	ENSMUSG00000021806.4	chr14	+	19802337	19802559	19798396	19798645	19803048	19803218	40664	3,5	3,3	6,5	0,0	298	149	6.61023151216e-07	0.000334158219992	0.333,0.455	1.0,1.0	-0.606
10-12m_vs_8-10w	SE	Colq	ENSMUSG00000057606.14	chr14	-	31525985	31526088	31523081	31524479	31528275	31528396	40680	5,6	1,3	4,4	0,0	251	149	0.000993761425369	0.0496569650624	0.748,0.543	1.0,1.0	-0.355
10-12m_vs_8-10w	SE	Med15	ENSMUSG00000012114.16	chr16	-	17663376	17663496	17655677	17655805	17671544	17671673	40698	8,24	3,5	8,8	0,0	268	149	2.47341444741e-05	0.00419112478237	0.597,0.727	1.0,1.0	-0.338
10-12m_vs_8-10w	SE	Cenpa	ENSMUSG00000029177.9	chr5	+	30673266	30673443	30672981	30673059	30674037	30674386	40727	824,1374	86,143	1504,1088	96,67	298	149	0.000527045967751	0.0330655400521	0.827,0.828	0.887,0.89	-0.061
10-12m_vs_8-10w	SE	Cenpa	ENSMUSG00000029177.9	chr5	+	30673292	30673443	30672981	30673059	30674037	30674202	40728	1558,2482	86,143	2881,2017	96,67	298	149	0.000252743887893	0.0208314534339	0.901,0.897	0.938,0.938	-0.039
10-12m_vs_8-10w	SE	Aprt	ENSMUSG00000006589.8	chr8	-	122576118	122576231	122575401	122575535	122576514	122576570	40805	3,2	129,129	14,13	223,143	261	149	0.000768972798338	0.0424067526298	0.013,0.009	0.035,0.049	-0.031
10-12m_vs_8-10w	SE	Prrg1	ENSMUSG00000047996.16	chrX	-	78561575	78561644	78520770	78520821	78583703	78583782	40855	2,10	1,2	1,1	5,3	217	149	0.000207111021517	0.0182869165808	0.579,0.774	0.121,0.186	0.523
10-12m_vs_8-10w	SE	Prrg1	ENSMUSG00000047996.16	chrX	-	78561575	78561644	78520770	78520821	78583768	78583891	40856	2,10	6,13	0,1	8,6	217	149	0.000242439368942	0.0204261902761	0.186,0.346	0.0,0.103	0.215

10-12m_vs_8-10w	SE	RP23-366P9.8	ENSMUSG00000107071.1	chr6	+	39587368	39587628	39586840	39586894	39587776	39587848	40889	6,2	0,0	6,4	4,2	298	149	3.26842265419e-06	0.000987336427744	1.0,1.0	0.429,0.5	0.536
10-12m_vs_8-10w	SE	Pcgf1	ENSMUSG00000069678.10	chr6	+	83079639	83079745	83079433	83079505	83079921	83079955	41009	2075,2242	32,51	2491,1949	17,19	254	149	6.5649341601e-05	0.00797752970522	0.974,0.963	0.988,0.984	-0.018
10-12m_vs_8-10w	SE	Guca1a	ENSMUSG00000023982.7	chr17	-	47395547	47395697	47395107	47395201	47400218	47400584	41073	496,915	47,67	1335,662	40,47	298	149	0.000952424958337	0.0484862326369	0.841,0.872	0.943,0.876	-0.053
10-12m_vs_8-10w	SE	Stk31	ENSMUSG00000023403.14	chr6	+	49457655	49457778	49447243	49447393	49465468	49465537	41112	185,207	3,1	198,191	7,16	271	149	4.33742497306e-05	0.00592605571432	0.971,0.991	0.94,0.868	0.077
10-12m_vs_8-10w	SE	Cadm1	ENSMUSG00000032076.19	chr9	+	47829376	47829409	47818733	47818817	47836716	47836770	41320	0,2	2,2	2,2	0,0	181	149	5.8687962976e-06	0.00147112777275	0.0,0.452	1.0,1.0	-0.774
10-12m_vs_8-10w	SE	Cadm1	ENSMUSG00000032076.19	chr9	+	47836716	47836770	47818733	47818817	47848166	47848298	41322	4,5	12,11	0,0	30,13	202	149	9.85026475198e-06	0.00211855952135	0.197,0.251	0.0,0.0	0.224
10-12m_vs_8-10w	SE	Cadm1	ENSMUSG00000032076.19	chr9	+	47836716	47836770	47829376	47829409	47848166	47848298	41323	2,3	3,4	0,0	1,2	202	149	0.000559239027542	0.0343585217816	0.33,0.356	0.0,0.0	0.343
10-12m_vs_8-10w	SE	Ilf2	ENSMUSG00000001016.12	chr3	+	90486850	90486912	90486184	90486272	90486982	90487097	41438	108,162	9,25	279,166	2,10	210	149	4.74479156387e-06	0.00129652498129	0.895,0.821	0.99,0.922	-0.098
10-12m_vs_8-10w	SE	Zfp957	ENSMUSG00000071262.10	chr14	-	79223378	79223863	79212354	79214469	79247216	79247367	41440	88,117	15,17	100,78	7,0	298	149	0.000279973494555	0.0222300420506	0.746,0.775	0.877,1.0	-0.178
10-12m_vs_8-10w	SE	Rpusd3	ENSMUSG00000051169.14	chr6	-	113417846	113417946	113417140	113417411	113418030	113418075	41550	13,17	0,0	43,11	6,4	248	149	3.73475353652e-05	0.00545517696261	1.0,1.0	0.812,0.623	0.283
10-12m_vs_8-10w	SE	Samd7	ENSMUSG00000051860.13	chr3	+	30754262	30754341	30753099	30753224	30756137	30756748	41559	8,20	4,2	12,6	0,0	227	149	0.000130626786545	0.0131095801458	0.568,0.868	1.0,1.0	-0.282
10-12m_vs_8-10w	SE	Ankrd16	ENSMUSG00000047909.11	chr2	+	11785169	11785260	11784292	11784452	11786223	11786302	41733	0,0	1,13	7,2	4,2	239	149	6.89909642082e-07	0.000337510473451	0.0,0.0	0.522,0.384	-0.453
10-12m_vs_8-10w	SE	Ifi27	ENSMUSG00000064215.13	chr12	+	103437675	103437705	103436562	103436682	103439400	103439562	41825	3,4	0,0	9,7	4,4	178	149	3.13358935718e-05	0.00495173608185	1.0,1.0	0.653,0.594	0.377
10-12m_vs_8-10w	SE	Xpnpep1	ENSMUSG00000025027.17	chr19	-	53002473	53002551	53000388	53000460	53002678	53002737	41865	29,34	4,3	41,52	0,1	226	149	0.000972922955181	0.0489381859927	0.827,0.882	1.0,0.972	-0.131
10-12m_vs_8-10w	SE	Crem	ENSMUSG00000063889.16	chr18	-	3276693	3276729	3273421	3273578	3295039	3295182	42153	1,1	18,9	9,5	18,12	184	149	0.000804510405067	0.0436442813064	0.043,0.083	0.288,0.252	-0.207
10-12m_vs_8-10w	SE	Tars2	ENSMUSG00000028107.14	chr3	-	95750866	95750984	95750700	95750765	95753006	95753130	42254	0,0	1,3	2,1	0,1	266	149	0.000334055505099	0.0246463100869	0.0,0.0	1.0,0.359	-0.68
10-12m_vs_8-10w	SE	Elk4	ENSMUSG00000026436.15	chr1	+	132013025	132013234	132008296	132008923	132014382	132014456	42312	6,6	4,2	4,4	0,0	298	149	2.79767704148e-06	0.000886033452992	0.429,0.6	1.0,1.0	-0.486
10-12m_vs_8-10w	SE	Ercc6	ENSMUSG00000054051.7	chr14	+	32574805	32575010	32569738	32570431	32575753	32575826	42418	11,8	0,0	16,8	2,6	298	149	9.54691732069e-06	0.00206833963753	1.0,1.0	0.8,0.4	0.4
10-12m_vs_8-10w	SE	Ercc6	ENSMUSG00000054051.7	chr14	+	32575753	32575826	32569738	32570431	32576685	32577104	42419	7,7	6,3	23,12	0,1	221	149	5.22890091059e-06	0.00136722235792	0.44,0.611	1.0,0.89	-0.42
10-12m_vs_8-10w	SE	Tmco3	ENSMUSG00000038497.7	chr8	+	13303727	13303914	13298212	13298305	13313805	13313956	42580	2,8	5,9	9,9	2,1	298	149	0.0006109267192 7	0.0361183763156	0.167,0.308	0.692,0.818	-0.517
10-12m_vs_8-10w	SE	Cnot6	ENSMUSG00000020362.13	chr11	-	49679920	49680151	49677299	49677502	49681140	49681295	42660	155,260	4,6	225,249	22,17	298	149	0.000152226678977	0.014473941693	0.951,0.956	0.836,0.88	0.096

10-12m_vs_8-10w	SE	AC133502.2	ENSMUSG00000031085.16	chr7	+	143891659	143891821	143881165	143881276	143894019	143894208	42801	0,1	5,6	8,2	6,1	298	149	4.20462266782e-05	0.00579683682444	0.0,0.077	0.4,0.5	-0.412
10-12m_vs_8-10w	SE	Acox1	ENSMUSG00000020777.16	chr11	-	116178119	116178310	116175204	116175384	116178681	116178844	42880	4,34	0,0	6,18	2,3	298	149	0.000237636868394	0.0201334185901	1.0,1.0	0.6,0.75	0.325
10-12m_vs_8-10w	SE	Trmt13	ENSMUSG00000033439.12	chr3	-	116588471	116588524	116585754	116585827	116589490	116589597	42930	8,19	0,0	6,13	4,3	201	149	1.29180227817e-05	0.00268367499309	1.0,1.0	0.527,0.763	0.355
10-12m_vs_8-10w	SE	Trmt13	ENSMUSG00000033439.12	chr3	-	116588471	116588639	116585754	116585827	116589490	116589597	42931	52,70	0,0	46,43	4,3	298	149	0.000376564038625	0.0268111827595	1.0,1.0	0.852,0.878	0.135
10-12m_vs_8-10w	SE	RP23-186O3.11	ENSMUSG00000111497.1	chr9	-	21068029	21068117	21067513	21067925	21076566	21076641	43158	11,34	4,2	22,28	0,0	236	149	0.000327069680933	0.0243145845401	0.635,0.915	1.0,1.0	-0.225
10-12m_vs_8-10w	SE	RP23-186O3.11	ENSMUSG00000111497.1	chr9	-	21073074	21073181	21068029	21068117	21076566	21076641	43162	10,10	0,6	15,19	0,0	255	149	0.000911441285722	0.0474183973194	1.0,0.493	1.0,1.0	-0.254
10-12m_vs_8-10w	SE	RP23-186O3.11	ENSMUSG00000111497.1	chr9	-	21075963	21076107	21067513	21067925	21076566	21076641	43166	0,5	4,2	3,3	0,0	292	149	1.03087419601e-07	7.06675203677e-05	0.0,0.561	1.0,1.0	-0.72
10-12m_vs_8-10w	SE	Tnfrsf19	ENSMUSG00000060548.13	chr14	-	61005074	61005253	60996604	60996690	61024168	61024279	43173	3,17	1,8	9,8	0,0	298	149	1.53739932318e-06	0.000616728997731	0.6,0.515	1.0,1.0	-0.443
10-12m_vs_8-10w	SE	Pdia4	ENSMUSG00000025823.9	chr6	-	47799185	47799342	47798364	47798598	47800937	47801089	43272	81,148	0,4	117,121	9,10	298	149	0.000500943457328	0.0323221713132	1.0,0.949	0.867,0.858	0.112
10-12m_vs_8-10w	SE	Rbm26	ENSMUSG00000022119.15	chr14	-	105139090	105139162	105131881	105132007	105140315	105140438	43325	2,7	49,46	20,14	53,53	220	149	0.000628347204897	0.0367213854947	0.027,0.093	0.204,0.152	-0.118
10-12m_vs_8-10w	SE	Zfyve19	ENSMUSG00000068580.11	chr2	+	119216431	119216541	119216208	119216307	119216628	119217049	43348	5,12	4,2	11,23	0,1	258	149	0.000246365111331	0.020565823823	0.419,0.776	1.0,0.93	-0.368
10-12m_vs_8-10w	SE	Orc4	ENSMUSG00000026761.12	chr2	-	48910185	48910242	48909418	48909514	48912561	48912648	43359	173,142	3,0	208,205	9,17	205	149	1.97177660037e-05	0.00347709046894	0.977,1.0	0.944,0.898	0.067
10-12m_vs_8-10w	SE	Orc4	ENSMUSG00000026761.12	chr2	-	48910185	48910245	48909418	48909514	48912561	48912575	43361	187,167	3,0	235,239	9,17	208	149	2.73707234272e-05	0.00450965464582	0.978,1.0	0.949,0.91	0.06
10-12m_vs_8-10w	SE	Orc4	ENSMUSG00000026761.12	chr2	-	48910185	48910294	48909418	48909514	48912561	48912648	43363	454,426	3,0	547,512	9,17	257	149	6.33347016148e-05	0.0078089627426	0.989,1.0	0.972,0.946	0.035
10-12m_vs_8-10w	SE	Mrpl4	ENSMUSG00000003299.10	chr9	+	21007327	21007540	21006826	21006856	21007822	21007877	43443	1,5	0,0	6,2	4,2	298	149	1.56446792121e-06	0.000616728997731	1.0,1.0	0.429,0.333	0.619
10-12m_vs_8-10w	SE	Mrpl4	ENSMUSG00000003299.10	chr9	+	21007433	21007540	21006826	21006856	21007822	21007899	43450	1,2	0,0	4,1	4,2	255	149	2.11895821001e-07	0.000126019846015	1.0,1.0	0.369,0.226	0.703
10-12m_vs_8-10w	SE	Tsr3	ENSMUSG00000015126.9	chr17	+	25241063	25241257	25240169	25240364	25242158	25242222	43499	19,15	4,1	17,10	0,0	298	149	0.000792512812197	0.0432332843647	0.704,0.882	1.0,1.0	-0.207
10-12m_vs_8-10w	SE	Tsr3	ENSMUSG00000015126.9	chr17	+	25241687	25241861	25241063	25241257	25242453	25242798	43507	57,59	10,12	103,44	4,3	298	149	0.000676380290965	0.0388898982105	0.74,0.711	0.928,0.88	-0.179
