# Supplementary material for: Integrative proteome analysis implicates aberrant RNA splicing in impaired developmental potential of aged mouse oocytes
Source: Aging Cell. 2021 Sep 28;20(10):e13482. doi: 10.1111/acel.13482 (PMC8520726; doi:10.1111/acel.13482)
Supplement: Supplementary file 9 — Table S5 [file ACEL-20-e13482-s004.pdf]

Table S5. List of alternative splicing events and genes at the 2-cell stage (6-8m versus 8-10w).

Group	Event	Gene_name	Gene_id	chrom	strand	riExonStart_0base	riExonEnd	upstreamES	upstreamEE	downstreamES	downstreamEE	ID	IJC_SAMPLE_1	SJC_SAMPLE_1	IJC_SAMPLE_2	SJC_SAMPLE_2	IncFormLen	SkipFormLen	PValue	FDR	IncLevel1	IncLevel2	IncLevelDifference
6-8m_vs_8-10w	A3SS	Reps1	ENSMUSG00000019854.17	chr10	+	18107667	18107748	18107670	18107748	18104153	18104275	130	164,119	16,7	115,117	27,28	151	149	5.26761950939e-05	0.005607620405	0.91,0.944	0.808,0.805	0.121
6-8m_vs_8-10w	A3SS	Ilkap	ENSMUSG00000026309.14	chr1	-	91388344	91388464	91388344	91388446	91390606	91390663	263	5,5	4,1	5,1	0,0	166	149	0.000178771352923	0.0113497526148	0.529,0.818	1.0,1.0	-0.327
6-8m_vs_8-10w	A3SS	Dusp22	ENSMUSG00000069255.13	chr13	+	30708676	30711231	30710705	30711231	30707929	30708101	289	40,19	5,3	33,29	1,0	298	149	0.000505720973928	0.0241713983865	0.8,0.76	0.943,1.0	-0.192
6-8m_vs_8-10w	A3SS	Catsperg1	ENSMUSG00000049676.14	chr7	-	29190212	29190635	29190212	29190331	29190726	29190789	577	8,6	1,0	5,1	2,4	298	149	9.39274403691e-05	0.00786736193488	0.8,1.0	0.556,0.111	0.567
6-8m_vs_8-10w	A3SS	Ercc1	ENSMUSG00000003549.9	chr7	+	19354325	19354425	19354330	19354425	19350692	19350792	607	18,18	8,7	20,19	2,0	153	149	9.40589812881e-05	0.00786736193488	0.687,0.715	0.907,1.0	-0.252
6-8m_vs_8-10w	A3SS	Mat2b	ENSMUSG00000042032.13	chr11	-	40685226	40685341	40685226	40685313	40687600	40687795	723	2792,1881	62,30	1587,1363	13,14	176	149	0.000734654669945	0.0307243078038	0.974,0.982	0.99,0.988	-0.011
6-8m_vs_8-10w	A3SS	Cnot1	ENSMUSG00000036550.16	chr8	-	95754964	95755111	95754964	95755108	95756091	95756293	725	12,13	0,1	7,8	3,5	151	149	7.06446753206e-05	0.00689374290004	1.0,0.928	0.697,0.612	0.31
6-8m_vs_8-10w	A3SS	Fam53a	ENSMUSG00000037339.17	chr5	-	33600355	33608227	33600355	33600880	33610431	33610486	728	7,3	0,0	2,2	2,2	298	149	1.21676720199e-05	0.00203547770504	1.0,1.0	0.333,0.333	0.667
6-8m_vs_8-10w	A3SS	Cox18	ENSMUSG00000035505.14	chr5	-	90218911	90219036	90218911	90219033	90222230	90222331	734	8,7	0,2	1,3	8,4	151	149	1.93176881506e-05	0.00266129562639	1.0,0.775	0.11,0.425	0.62
6-8m_vs_8-10w	A3SS	RP23-136M3.6	ENSMUSG00000092515.8	chr12	+	56593879	56594007	56593907	56594007	56591810	56591895	881	93,98	9,6	61,60	1,1	176	149	0.00134177555136	0.0455425846563	0.897,0.933	0.981,0.981	-0.066
6-8m_vs_8-10w	A3SS	Cd72	ENSMUSG00000028459.11	chr4	-	43449451	43449549	43449451	43449528	43450069	43450221	978	1,4	2,4	0,0	10,1	169	149	7.7575517368e-05	0.00726727446703	0.306,0.469	0.0,0.0	0.387
6-8m_vs_8-10w	A3SS	Odf2l	ENSMUSG00000028256.16	chr3	+	145145875	145148616	145148530	145148616	145144311	145144416	1240	3,8	12,17	0,0	7,21	298	149	3.06315776688e-05	0.00377574499475	0.111,0.19	0.0,0.0	0.151
6-8m_vs_8-10w	A3SS	Ica1l	ENSMUSG00000026018.12	chr1	-	60028067	60028246	60028067	60028236	60042796	60043087	1322	0,0	1,4	1,2	1,0	158	149	3.25854341242e-05	0.00381575433594	0.0,0.0	0.485,1.0	-0.742
6-8m_vs_8-10w	A3SS	AC121113.1	ENSMUSG00000006010.14	chr1	-	150388522	150388657	150388522	150388619	150390235	150390356	1351	26,22	8,4	17,13	1,0	186	149	0.000227507918143	0.0129956962022	0.722,0.815	0.932,1.0	-0.198
6-8m_vs_8-10w	A3SS	Ift88	ENSMUSG00000040040.16	chr14	+	57437176	57437283	57437226	57437283	57434794	57434857	1408	1,0	7,5	2,1	0,1	198	149	6.26242769886e-05	0.00637678507423	0.097,0.0	1.0,0.429	-0.666
6-8m_vs_8-10w	A3SS	Zfp90	ENSMUSG00000031907.9	chr8	+	106419420	106419610	106419514	106419610	106419070	106419197	1440	1,1	0,0	0,0	1,2	242	149	7.59807139428e-09	5.93156106847e-06	1.0,1.0	0.0,0.0	1.0
6-8m_vs_8-10w	A3SS	Olfr653	ENSMUSG00000073926.4	chr7	+	104579373	104579523	104579394	104579523	104577545	104577598	1454	5,5	0,0	3,1	6,2	169	149	1.20228503819e-07	4.69291926573e-05	1.0,1.0	0.306,0.306	0.694

6-8m_vs_8-10w	A3SS	Stk11	ENSMUSG00000003068.16	chr10	+	80126032	80126369	80126232	80126369	80125478	80125568	1500	25,19	0,0	17,7	4,2	298	149	4.09938451351e-05	0.0045717897765	1.0,1.0	0.68,0.636	0.342
6-8m_vs_8-10w	A3SS	Msi2	ENSMUSG00000069769.13	chr11	-	88348783	88348992	88348783	88348938	88366784	88366847	1550	5,0	8,1	3,3	0,1	202	149	0.000181478136928	0.0113497526148	0.316,0.0	1.0,0.689	-0.687
6-8m_vs_8-10w	A3SS	Mvk	ENSMUSG00000041939.14	chr5	+	114450240	114450421	114450276	114450421	114446158	114446306	1599	4,1	4,2	0,0	1,2	184	149	0.000120305519169	0.0093123272307 6	0.447,0.288	0.0,0.0	0.368
6-8m_vs_8-10w	A3SS	Ankrd17	ENSMUSG00000055204.15	chr5	-	90278242	90278391	90278242	90278388	90282850	90283603	1610	14,5	4,8	6,2	0,0	151	149	1.3403259797e-05	0.0020926956296 4	0.775,0.381	1.0,1.0	-0.422
6-8m_vs_8-10w	A3SS	Rpl13	ENSMUSG00000000740.12	chr8	+	123102844	123103356	123103182	123103356	123102652	123102762	1827	549,313	48,38	445,311	21,16	298	149	0.000831971617282	0.0330923492302	0.851,0.805	0.914,0.907	-0.083
6-8m_vs_8-10w	A3SS	Uhrf1	ENSMUSG00000001228.14	chr17	+	56313040	56313251	56313064	56313251	56312863	56312967	1837	7,2	0,0	2,4	3,1	172	149	0.0010068982265	0.0380347684913	1.0,1.0	0.366,0.776	0.429
6-8m_vs_8-10w	A3SS	Atxn7l3	ENSMUSG00000059995.12	chr11	-	102293066	102293152	102293066	102293095	102293275	102293321	1853	1,0	14,19	3,5	26,6	205	149	0.00100005633956	0.0380347684913	0.049,0.0	0.077,0.377	-0.203
6-8m_vs_8-10w	A3SS	Fxyd4	ENSMUSG00000004988.9	chr6	-	117935853	117936006	117935853	117935922	117936737	117936780	1961	3,3	1,7	2,7	0,1	232	149	0.000302602617359	0.0150786240395	0.658,0.216	1.0,0.818	-0.472
6-8m_vs_8-10w	A3SS	RP23-414P13.3	ENSMUSG00000110393.2	chr13	+	65247441	65247767	65247640	65247767	65241752	65242015	2029	8,2	11,8	1,0	5,6	298	149	0.00139236564401	0.046584576261	0.267,0.111	0.091,0.0	0.144
6-8m_vs_8-10w	A3SS	Xlr4b	ENSMUSG00000067768.12	chrX	+	73215227	73215355	73215240	73215355	73214773	73214820	2085	15,13	0,0	7,6	3,2	161	149	0.000242140355483	0.0134066337861	1.0,1.0	0.683,0.735	0.291
6-8m_vs_8-10w	A3SS	Get4	ENSMUSG00000025858.12	chr5	+	139262869	139262951	139262872	139262951	139262480	139262559	2237	38,10	4,5	12,11	0,0	151	149	0.000246150833819	0.0134066337861	0.904,0.664	1.0,1.0	-0.216
6-8m_vs_8-10w	A3SS	Setd5	ENSMUSG00000034269.12	chr6	+	113110316	113110525	113110373	113110525	113109884	113109990	2261	6,3	0,0	1,0	3,1	205	149	3.6493456812e-08	1.70935351707e-05	1.0,1.0	0.195,0.0	0.903
6-8m_vs_8-10w	A3SS	Pum2	ENSMUSG00000020594.14	chr12	+	8718987	8719113	8719005	8719113	8713843	8713937	2284	46,8	8,2	18,7	0,0	166	149	0.000130967021968	0.0093123272307 6	0.838,0.782	1.0,1.0	-0.19
6-8m_vs_8-10w	A3SS	Pum2	ENSMUSG00000020594.14	chr12	+	8719002	8719113	8719005	8719113	8713843	8713937	2285	9,2	8,2	5,2	0,0	151	149	5.22653433055e-06	0.0011127766729 2	0.526,0.497	1.0,1.0	-0.488
6-8m_vs_8-10w	A3SS	Abhd3	ENSMUSG00000002475.15	chr18	-	10644412	10645235	10644412	10645181	10647725	10647891	2321	21,17	5,4	21,4	0,1	202	149	0.00134060677857	0.0455425846563	0.756,0.758	1.0,0.747	-0.116
6-8m_vs_8-10w	A3SS	Aaas	ENSMUSG00000036678.8	chr15	-	102345576	102345960	102345576	102345675	102346486	102346533	2524	4,4	19,5	0,0	7,4	298	149	0.000637403143923	0.0290471284671	0.095,0.286	0.0,0.0	0.191
6-8m_vs_8-10w	A3SS	Tatdn3	ENSMUSG00000026632.17	chr1	-	191049281	191049362	191049281	191049359	191054855	191054911	2526	5,2	0,0	5,2	5,1	151	149	1.16380297056e-05	0.0020354777050 4	1.0,1.0	0.497,0.664	0.42
6-8m_vs_8-10w	A3SS	Nfat5	ENSMUSG00000003847.16	chr8	+	107361743	107361824	107361746	107361824	107358633	107358766	2538	0,1	1,7	3,2	2,3	151	149	0.000756946836917	0.0311012191589	0.0,0.124	0.597,0.397	-0.435
6-8m_vs_8-10w	A3SS	Ralgapb	ENSMUSG00000027652.15	chr2	+	158450176	158450310	158450188	158450310	158448319	158448449	2548	5,4	4,1	1,4	0,0	160	149	0.000135191770216	0.0093123272307 6	0.538,0.788	1.0,1.0	-0.337

6-8m_vs_8-10w	A3SS	Sntg2	ENSMUSG00000020672.14	chr12	-	30307326	30307383	30307326	30307378	30312535	30312673	2578	5,2	0,0	2,4	3,4	153	149	2.2293262536e-06	0.000544463817361	1.0,1.0	0.394,0.493	0.557
6-8m_vs_8-10w	A3SS	Helz	ENSMUSG00000020721.16	chr11	+	107627349	107627454	107627352	107627454	107626534	107626714	2586	0,0	3,6	4,1	5,3	151	149	0.00011894634068 8	0.0093123272307 6	0.0,0.0	0.441,0.248	-0.345
6-8m_vs_8-10w	A3SS	Cic	ENSMUSG00000005442.13	chr7	+	25291051	25291342	25291054	25291342	25290798	25290965	2587	2,2	2,2	4,1	1,0	151	149	0.00131843232329	0.0455425846563	0.497,0.497	0.798,1.0	-0.402
6-8m_vs_8-10w	A3SS	Vmn1r69	ENSMUSG00000091662.9	chr7	-	10578929	10581879	10578929	10580579	10583182	10583237	2613	0,5	2,1	7,3	0,0	298	149	0.00098394800167 8	0.0380347684913	0.0,0.714	1.0,1.0	-0.643
6-8m_vs_8-10w	A3SS	Nsun3	ENSMUSG00000050312.11	chr16	-	62776287	62776631	62776287	62776383	62785787	62785897	2659	89,18	10,8	41,28	1,0	298	149	1.7251851957e-05	0.00252523983021	0.817,0.529	0.953,1.0	-0.303
6-8m_vs_8-10w	A3SS	Ank3	ENSMUSG00000069601.14	chr10	+	70001922	70002601	70002510	70002601	69999354	69999498	2791	10,14	1,0	6,13	3,4	298	149	0.00028415403441 1	0.014477109772	0.833,1.0	0.5,0.619	0.357
6-8m_vs_8-10w	A3SS	Ap4m1	ENSMUSG00000019518.10	chr5	+	138176051	138176263	138176209	138176263	138175911	138175974	2899	9,4	6,2	3,6	0,0	298	149	1.86384579226e-06	0.000544463817361	0.429,0.5	1.0,1.0	-0.536
6-8m_vs_8-10w	A3SS	Ankrd9	ENSMUSG00000037904.14	chr12	-	110976426	110977770	110976426	110977548	110977947	110978127	2973	4,2	7,0	5,4	0,0	298	149	0.00124994107924	0.0443539698118	0.222,1.0	1.0,1.0	-0.389
6-8m_vs_8-10w	A3SS	Ly6e	ENSMUSG00000022587.14	chr15	+	74957776	74957877	74957779	74957877	74957414	74957463	3125	4,2	0,0	3,2	3,1	151	149	0.00064095083625 4	0.0290471284671	1.0,1.0	0.497,0.664	0.42
6-8m_vs_8-10w	A3SS	Clk2	ENSMUSG00000068917.12	chr3	+	89169614	89170115	89170048	89170115	89168694	89168923	3276	3,5	0,0	4,0	3,3	298	149	1.0662698724e-08	6.2430101029e-06	1.0,1.0	0.4,0.0	0.8
6-8m_vs_8-10w	A3SS	Cdc6	ENSMUSG00000017499.15	chr11	+	98911387	98911563	98911390	98911563	98910577	98910777	3291	17,5	0,1	12,5	4,2	151	149	0.00145010168168	0.0478329315281	1.0,0.831	0.747,0.712	0.186
6-8m_vs_8-10w	A3SS	Shroom2	ENSMUSG00000045180.13	chrX	-	152623125	152623335	152623125	152623317	152657423	152657524	3314	2,0	2,1	4,1	0,0	166	149	0.0010544895458	0.0392002304169	0.473,0.0	1.0,1.0	-0.764
6-8m_vs_8-10w	A3SS	Pafah1b1	ENSMUSG00000020745.15	chr11	-	74673948	74679614	74673948	74677742	74683458	74683687	3437	2,5	0,0	4,3	5,1	298	149	5.15059778161e-07	0.00017232428577 9	1.0,1.0	0.286,0.6	0.557
6-8m_vs_8-10w	A3SS	Cpsf4l	ENSMUSG00000018727.19	chr11	-	113702412	113702509	113702412	113702506	113703270	113703366	3478	7,1	46,12	0,0	8,9	151	149	0.00120630002771	0.0434639179215	0.131,0.076	0.0,0.0	0.104
6-8m_vs_8-10w	A3SS	Zfp653	ENSMUSG00000038895.16	chr9	-	22057500	22057702	22057500	22057678	22057803	22058415	3532	3,4	1,0	0,0	3,3	172	149	1.26953114687e-10	2.97324194597e-07	0.722,1.0	0.0,0.0	0.861
6-8m_vs_8-10w	A3SS	Brd9	ENSMUSG00000057649.6	chr13	+	73942630	73942806	73942693	73942806	73941943	73942054	3664	1,0	0,3	3,1	0,0	211	149	0.00067867126297 9	0.0290471284671	1.0,0.0	1.0,1.0	-0.5
6-8m_vs_8-10w	A3SS	Alg10b	ENSMUSG00000075470.2	chr15	+	90227323	90233471	90227472	90233471	90225656	90225854	4148	8,2	2,3	9,8	0,0	297	149	9.17615374973e-06	0.00179087934016	0.667,0.251	1.0,1.0	-0.541
6-8m_vs_8-10w	A3SS	RP23-221O14.1	ENSMUSG00000044320.14	chr2	-	30797867	30798028	30797867	30797980	30800723	30800816	4223	1,2	1,6	4,1	1,0	196	149	0.00016469216226 7	0.0110202584008	0.432,0.202	0.753,1.0	-0.56
6-8m_vs_8-10w	A3SS	Thoc2	ENSMUSG00000037475.15	chrX	-	41802946	41802992	41802946	41802986	41803455	41803532	4241	3,6	0,0	9,4	3,2	154	149	0.00068214861899 6	0.0290471284671	1.0,1.0	0.744,0.659	0.299

6-8m_vs_8-10w	A3SS	Miip	ENSMUSG00000029022.18	chr4	-	147860790	147861085	147860790	147861013	147861353	147861491	4357	10,4	0,0	15,6	1,4	220	149	0.00068070245969 5	0.0290471284671	1.0,1.0	0.91,0.504	0.293
6-8m_vs_8-10w	A3SS	Rhebl1	ENSMUSG00000023755.10	chr15	-	98878452	98878593	98878452	98878534	98879258	98879315	4547	30,19	52,29	29,27	14,19	207	149	0.00065007962306 8	0.0290471284671	0.293,0.32	0.599,0.506	-0.246
6-8m_vs_8-10w	A3SS	Rhebl1	ENSMUSG00000023755.10	chr15	-	98878452	98879055	98878452	98878534	98879258	98879315	4548	300,138	52,29	208,173	14,19	298	149	0.00083366720947 1	0.0330923492302	0.743,0.704	0.881,0.82	-0.127
6-8m_vs_8-10w	A3SS	C2cd3	ENSMUSG00000047248.20	chr7	+	100379967	100380125	100380025	100380125	100374263	100374533	4557	43,30	6,1	18,13	0,0	206	149	0.0011002309037	0.0402615746323	0.838,0.956	1.0,1.0	-0.103
6-8m_vs_8-10w	A3SS	Foxred1	ENSMUSG00000039048.15	chr9	-	35204207	35205141	35204207	35205123	35205304	35205409	4700	2,2	2,1	0,0	9,5	166	149	0.00021562056088 2	0.0126245838396	0.473,0.642	0.0,0.0	0.558
6-8m_vs_8-10w	A3SS	Phrf1	ENSMUSG00000038611.17	chr7	+	141261212	141261542	141261287	141261542	141260959	141261114	4744	22,13	1,0	20,19	6,3	223	149	0.00028434972225 2	0.014477109772	0.936,1.0	0.69,0.809	0.218
6-8m_vs_8-10w	A3SS	Zfas1	ENSMUSG00000074578.13	chr2	+	167065158	167065480	167065433	167065480	167063635	167063727	4772	57,51	2,6	69,24	0,0	298	149	0.00026110396360 7	0.0138978518811	0.934,0.81	1.0,1.0	-0.128
6-8m_vs_8-10w	A3SS	Alkbh6	ENSMUSG00000042831.13	chr7	+	30312505	30312707	30312590	30312707	30312236	30312388	4797	5,3	4,4	7,4	0,1	233	149	0.00013199676664 9	0.00931232723076	0.444,0.324	1.0,0.719	-0.475
6-8m_vs_8-10w	A3SS	Rnaseh2a	ENSMUSG00000052926.16	chr8	-	84957666	84957864	84957666	84957853	84957965	84958089	4954	66,46	0,0	82,43	5,5	159	149	0.00049919918606 3	0.0241713983865	1.0,1.0	0.939,0.89	0.086
6-8m_vs_8-10w	A3SS	Uevld	ENSMUSG00000043262.16	chr7	-	46940132	46940303	46940132	46940277	46943787	46943890	5184	26,2	1,0	6,8	5,2	174	149	0.00013235726182 2	0.00931232723076	0.957,1.0	0.507,0.774	0.338
6-8m_vs_8-10w	A3SS	Atp5h	ENSMUSG00000034566.10	chr11	-	115418385	115418550	115418385	115418516	115418698	115418741	5267	10,19	0,0	6,9	1,5	182	149	2.73073398342e-05	0.00355298832732	1.0,1.0	0.831,0.596	0.287
6-8m_vs_8-10w	A3SS	Ipmk	ENSMUSG00000060733.13	chr10	+	71372704	71372880	71372707	71372880	71347835	71348090	5401	1,0	17,6	3,6	7,12	151	149	9.00337726204e-05	0.00786736193488	0.055,0.0	0.297,0.33	-0.286
6-8m_vs_8-10w	A3SS	Msantd2	ENSMUSG00000042138.8	chr9	+	37517216	37517472	37517372	37517472	37513613	37513703	5406	6,5	4,2	10,3	0,0	298	149	2.32478145756e-06	0.00054446381736 1	0.429,0.556	1.0,1.0	-0.507
6-8m_vs_8-10w	A3SS	Cpsf1	ENSMUSG00000034022.8	chr15	-	76596219	76596571	76596219	76596349	76596650	76596698	5461	14,5	10,5	20,14	0,0	298	149	1.25848187427e-09	1.47368227477e-06	0.412,0.333	1.0,1.0	-0.628
6-8m_vs_8-10w	A3SS	Mrpl4	ENSMUSG00000003299.10	chr9	+	21007327	21007540	21007433	21007540	21006826	21006856	5542	41,22	8,3	30,15	1,0	254	149	0.00018415482466 3	0.0113497526148	0.75,0.811	0.946,1.0	-0.193
6-8m_vs_8-10w	A3SS	Mrpl4	ENSMUSG00000003299.10	chr9	+	21007363	21007540	21007433	21007540	21006826	21006856	5543	28,19	8,3	23,11	1,0	218	149	0.00020131377215 3	0.0120891501124	0.705,0.812	0.94,1.0	-0.212
6-8m_vs_8-10w	A5SS	Pkib	ENSMUSG00000019876.15	chr10	+	57708073	57709635	57708073	57708181	57728089	57728266	1	11,5	0,0	0,0	1,1	298	149	9.23670695485e-10	7.10764600176e-07	1.0,1.0	0.0,0.0	1.0
6-8m_vs_8-10w	A5SS	Rtbdn	ENSMUSG00000048617.16	chr8	+	84949881	84950676	84949881	84949956	84952643	84952788	26	3,2	0,0	0,0	4,1	298	149	1.97759253417e-10	3.04351491009e-07	1.0,1.0	0.0,0.0	1.0
6-8m_vs_8-10w	A5SS	Strada	ENSMUSG00000069631.14	chr11	-	106187100	106187180	106187124	106187180	106173678	106173781	107	2,2	0,0	2,0	2,1	172	149	0.00088943422209 4	0.0431151708234	1.0,1.0	0.464,0.0	0.768

6-8m_vs_8-10w	A5SS	Mcph1	ENSMUSG00000039842.15	chr8	+	18627117	18627318	18627117	18627240	18629545	18629635	217	1,0	14,4	4,4	6,12	226	149	0.000600792100522	0.0355622708732	0.045,0.0	0.305,0.18	-0.22
6-8m_vs_8-10w	A5SS	Eif4a2	ENSMUSG00000022884.14	chr16	+	23110579	23111263	23110579	23110723	23111502	23111592	431	622,375	15,32	494,383	6,1	298	149	1.85636232719e-07	5.71388324309e-05	0.954,0.854	0.976,0.995	-0.082
6-8m_vs_8-10w	A5SS	Alg5	ENSMUSG00000036632.9	chr3	+	54738781	54738953	54738781	54738951	54739261	54739308	493	20,14	5,2	26,23	1,0	150	149	0.000980526951799	0.0431151708234	0.799,0.874	0.963,1.0	-0.145
6-8m_vs_8-10w	A5SS	Gckr	ENSMUSG00000059434.8	chr5	+	31324571	31324721	31324571	31324613	31326356	31326491	631	19,6	1,4	7,7	0,0	256	149	0.000690922964608	0.0393826089827	0.917,0.466	1.0,1.0	-0.309
6-8m_vs_8-10w	A5SS	RP23-414K1.4	ENSMUSG00000033029.12	chr15	-	79140768	79140992	79140880	79140992	79139237	79139300	738	8,6	16,9	1,0	18,15	260	149	4.44489789941e-06	0.000977242552456	0.223,0.276	0.031,0.0	0.234
6-8m_vs_8-10w	A5SS	Uhrf1bp1l	ENSMUSG00000019951.10	chr10	+	89809627	89809778	89809627	89809763	89811486	89811740	763	7,13	3,2	11,4	0,0	163	149	0.00113730520114	0.0437578176139	0.681,0.856	1.0,1.0	-0.232
6-8m_vs_8-10w	A5SS	Sel1l	ENSMUSG00000020964.14	chr12	-	91833054	91833222	91833204	91833222	91831568	91831674	787	12,19	3,1	8,4	11,5	298	149	7.77374805438e-05	0.0119637982557	0.667,0.905	0.267,0.286	0.51
6-8m_vs_8-10w	A5SS	Slc17a2	ENSMUSG00000036110.14	chr13	+	23819248	23820229	23819248	23819410	23820984	23821109	793	5,6	0,0	1,1	1,2	298	149	9.51090442138e-05	0.0133066199132	1.0,1.0	0.333,0.2	0.734
6-8m_vs_8-10w	A5SS	Spata6	ENSMUSG00000034401.16	chr4	+	111805930	111806030	111805930	111805982	111822740	111822832	1042	12,30	1,5	20,19	0,0	196	149	0.00104674539635	0.0437578176139	0.901,0.82	1.0,1.0	-0.139
6-8m_vs_8-10w	A5SS	Ncapg	ENSMUSG00000015880.13	chr5	+	45691601	45693338	45691601	45691826	45693751	45693926	1164	103,104	15,5	114,111	3,2	298	149	0.0010640342076	0.0437578176139	0.774,0.912	0.95,0.965	-0.115
6-8m_vs_8-10w	A5SS	Nob1	ENSMUSG00000003848.14	chr8	-	107424737	107424870	107424751	107424870	107421481	107421612	1233	63,27	3,10	62,32	2,0	162	149	0.000538377054298	0.0342134909649	0.951,0.713	0.966,1.0	-0.151
6-8m_vs_8-10w	A5SS	Xlr4b	ENSMUSG00000067768.12	chrX	+	73214773	73214856	73214773	73214820	73215240	73215355	1251	30,15	0,0	9,9	3,2	184	149	0.000391164480442	0.0286667683524	1.0,1.0	0.708,0.785	0.254
6-8m_vs_8-10w	A5SS	Tmem234	ENSMUSG00000028797.20	chr4	+	129600982	129601243	129600982	129601134	129601869	129601961	1256	6,1	0,0	0,1	1,1	257	149	0.000430848454489	0.0296484020593	1.0,1.0	0.0,0.367	0.817
6-8m_vs_8-10w	A5SS	Tmem234	ENSMUSG00000028797.20	chr4	+	129600982	129601243	129600982	129601134	129602194	129602261	1257	8,4	2,0	3,3	6,3	257	149	0.00108611918412	0.0437578176139	0.699,1.0	0.225,0.367	0.553
6-8m_vs_8-10w	A5SS	Xlr4c	ENSMUSG00000031362.17	chrX	-	73242577	73242660	73242613	73242660	73241315	73241384	1289	9,5	0,0	7,9	4,2	184	149	4.65236040731e-05	0.0079555362965	1.0,1.0	0.586,0.785	0.315
6-8m_vs_8-10w	A5SS	Rabep1	ENSMUSG00000020817.16	chr11	+	70908385	70908556	70908385	70908517	70917314	70917782	1405	6,4	19,11	0,0	12,8	187	149	4.42151146719e-05	0.0079555362965	0.201,0.225	0.0,0.0	0.213
6-8m_vs_8-10w	A5SS	Fbxl3	ENSMUSG00000022124.15	chr14	-	103095192	103095541	103095336	103095541	103092303	103092426	1459	10,7	2,2	8,4	0,0	292	149	0.00141449398332	0.0499676767232	0.718,0.641	1.0,1.0	-0.321
6-8m_vs_8-10w	A5SS	Traf2	ENSMUSG00000026942.13	chr2	-	25538844	25539084	25538865	25539084	25537070	25537149	1462	8,9	1,0	2,2	2,2	169	149	0.000443088529801	0.0296484020593	0.876,1.0	0.469,0.469	0.469
6-8m_vs_8-10w	A5SS	Prkcsh	ENSMUSG00000003402.14	chr9	+	22011437	22011596	22011437	22011575	22011673	22011770	1465	2,3	0,0	0,0	1,1	169	149	3.31208860338e-09	1.27432609015e-06	1.0,1.0	0.0,0.0	1.0

6-8m_vs_8-10w	A5SS	Gpbp1	ENSMUSG00000032745.18	chr13	-	111453023	111453423	111453372	111453423	111448932	111448999	1482	1969,1465	192,110	1516,1458	412,161	298	149	0.00127284971604	0.0466408503092	0.837,0.869	0.648,0.819	0.119
6-8m_vs_8-10w	A5SS	Sntg2	ENSMUSG00000020672.14	chr12	-	30201274	30201367	30201285	30201367	30195537	30195648	1542	12,18	2,5	7,8	0,0	159	149	0.00024563686801	0.0210019522149	0.849,0.771	1.0,1.0	-0.19
6-8m_vs_8-10w	A5SS	Adrm1	ENSMUSG00000039041.15	chr2	+	180172855	180174033	180172855	180172972	180174308	180174395	1658	161,57	9,2	95,62	1,0	298	149	0.000902119397464	0.0431151708234	0.899,0.934	0.979,1.0	-0.073
6-8m_vs_8-10w	A5SS	Yap1	ENSMUSG00000053110.13	chr9	-	7962282	7962402	7962288	7962402	7952952	7953134	1715	3,3	20,11	0,0	10,11	154	149	0.00142857555284	0.0499676767232	0.127,0.209	0.0,0.0	0.168
6-8m_vs_8-10w	A5SS	Pum1	ENSMUSG00000028580.15	chr4	+	130765920	130766055	130765920	130766049	130768815	130768953	1820	2,4	8,4	7,7	1,1	154	149	0.000215385623306	0.0204567466114	0.195,0.492	0.871,0.871	-0.528
6-8m_vs_8-10w	A5SS	Dip2c	ENSMUSG00000048264.16	chr13	+	9614346	9614468	9614346	9614456	9615744	9615825	1887	2,2	0,0	0,0	2,1	160	149	2.82926826412e-09	1.27432609015e-06	1.0,1.0	0.0,0.0	1.0
6-8m_vs_8-10w	A5SS	Celf2	ENSMUSG00000002107.18	chr2	-	6560658	6560790	6560670	6560790	6553784	6553982	1985	6,3	9,4	1,0	8,5	160	149	0.000204569069124	0.0204567466114	0.383,0.411	0.104,0.0	0.345
6-8m_vs_8-10w	A5SS	Phkg2	ENSMUSG00000030815.11	chr7	+	127577534	127577710	127577534	127577688	127577974	127578029	2028	22,5	7,0	13,12	0,0	170	149	0.000329087864925	0.025323311206	0.734,1.0	1.0,1.0	-0.133
6-8m_vs_8-10w	A5SS	Phkg2	ENSMUSG00000030815.11	chr7	+	127577534	127578029	127577534	127577688	127579666	127579732	2033	30,14	1,0	30,19	8,1	298	149	0.000555774707032	0.0342134909649	0.938,1.0	0.652,0.905	0.191
6-8m_vs_8-10w	A5SS	Pbrm1	ENSMUSG00000042323.17	chr14	+	31023066	31023179	31023066	31023170	31025497	31025650	2099	2,0	1,2	2,2	0,0	157	149	0.00116752546546	0.043824919301	0.655,0.0	1.0,1.0	-0.673
6-8m_vs_8-10w	A5SS	Cltc	ENSMUSG00000047126.17	chr11	-	86737058	86737278	86737070	86737278	86733575	86733844	2104	7,1	11,8	0,0	4,2	160	149	0.000125010125934	0.016032548651	0.372,0.104	0.0,0.0	0.238
6-8m_vs_8-10w	A5SS	Slc25a26	ENSMUSG00000045100.11	chr6	+	94507513	94507707	94507513	94507670	94510751	94510861	2236	4,1	4,4	0,0	6,3	185	149	0.000321252460673	0.025323311206	0.446,0.168	0.0,0.0	0.307
6-8m_vs_8-10w	A5SS	Birc6	ENSMUSG00000024073.14	chr17	+	74605988	74606135	74605988	74606123	74608096	74608284	2260	2,2	1,4	0,0	2,2	160	149	0.00097329811197	0.0431151708234	0.651,0.318	0.0,0.0	0.485
6-8m_vs_8-10w	A5SS	Cecr2	ENSMUSG00000071226.11	chr6	+	120756548	120756937	120756548	120756853	120757565	120757691	2434	1,0	5,4	5,2	7,1	232	149	0.000877834392213	0.0431151708234	0.114,0.0	0.314,0.562	-0.381
6-8m_vs_8-10w	A5SS	Zfand2b	ENSMUSG00000026197.12	chr1	+	75170538	75170753	75170538	75170599	75170975	75171048	2642	10,4	0,0	4,5	2,2	298	149	0.000150147761695	0.0177751850191	1.0,1.0	0.5,0.556	0.472
6-8m_vs_8-10w	A5SS	Ccdc130	ENSMUSG00000004994.12	chr8	-	84262966	84263048	84262992	84263048	84261739	84261800	2969	1,1	27,11	3,7	11,15	174	149	0.000839434925478	0.0431151708234	0.031,0.072	0.189,0.286	-0.186
6-8m_vs_8-10w	A5SS	Tle1	ENSMUSG00000008305.18	chr4	-	72169091	72169196	72169121	72169196	72158212	72158417	3011	2,5	7,1	0,1	5,16	178	149	0.000188797109811	0.0204567466114	0.193,0.807	0.0,0.05	0.475
6-8m_vs_8-10w	A5SS	Smarcb1	ENSMUSG00000000902.13	chr10	-	75904383	75906196	75906029	75906196	75897444	75897576	3021	56,23	1,0	53,33	6,2	298	149	0.000934084115456	0.0431151708234	0.966,1.0	0.815,0.892	0.13
6-8m_vs_8-10w	A5SS	Prss44	ENSMUSG00000032493.8	chr9	+	110814599	110814756	110814599	110814723	110815292	110815552	3079	2,2	0,0	2,1	2,3	181	149	9.15281769709e-07	0.00023476977393	1.0,1.0	0.452,0.215	0.667

6-8m_vs_8-10w	A5SS	Smc2	ENSMUSG00000028312.19	chr4	+	52439242	52439410	52439242	52439334	52440200	52440423	3090	14,16	2,1	15,3	6,8	224	149	0.00112714964425	0.0437578176139	0.823,0.914	0.624,0.2	0.457
6-8m_vs_8-10w	A5SS	Gpr19	ENSMUSG00000032641.18	chr6	-	134896857	134897213	134897161	134897213	134887629	134887785	3320	13,7	5,3	2,1	9,2	298	149	0.000225967961269	0.0204567466114	0.565,0.538	0.1,0.2	0.401
6-8m_vs_8-10w	A5SS	Mrpl4	ENSMUSG00000003299.10	chr9	+	21006826	21006944	21006826	21006856	21007433	21007540	3326	179,47	8,3	146,68	1,0	236	149	0.000927836044396	0.0431151708234	0.934,0.908	0.989,1.0	-0.073
6-8m_vs_8-10w	RI	Trmt1l	ENSMUSG00000053286.11	chr1	+	151433793	151436707	151433793	151433904	151435727	151436707	47	2,0	4,3	2,2	1,0	298	149	0.000176716905788	0.0214887757438	0.2,0.0	0.5,1.0	-0.65
6-8m_vs_8-10w	RI	Dpp7	ENSMUSG00000026958.13	chr2	-	25353152	25354293	25353152	25353217	25354232	25354293	197	10,4	2,2	1,1	0,0	298	149	0.0010359978478	0.0497278966944	0.714,0.5	1.0,1.0	-0.393
6-8m_vs_8-10w	RI	Catsperg1	ENSMUSG00000049676.14	chr7	-	29190212	29190789	29190212	29190331	29190726	29190789	446	5,7	1,0	4,1	2,4	298	149	9.55125151783e-05	0.0145179023071	0.714,1.0	0.5,0.111	0.552
6-8m_vs_8-10w	RI	Cdk10	ENSMUSG00000033862.7	chr8	+	123227659	123228396	123227659	123227765	123228328	123228396	581	2,2	0,0	1,1	4,0	298	149	0.000995315251251	0.0490663518455	1.0,1.0	0.111,1.0	0.445
6-8m_vs_8-10w	RI	Mia	ENSMUSG00000089661.9	chr7	-	27180168	27180833	27180168	27180279	27180699	27180833	741	2,1	0,0	1,0	2,1	298	149	1.12296609867e-06	0.000409658032795	1.0,1.0	0.2,0.0	0.9
6-8m_vs_8-10w	RI	Phf11c	ENSMUSG00000068245.14	chr14	-	59348731	59349388	59348731	59349051	59349221	59349388	867	13,10	16,16	14,12	4,4	298	149	0.000907272460991	0.048790968483	0.289,0.238	0.636,0.6	-0.355
6-8m_vs_8-10w	RI	Spsb3	ENSMUSG00000024160.16	chr17	+	24890567	24890935	24890567	24890755	24890832	24890935	940	7,0	8,6	0,0	8,1	225	149	0.000698220197109	0.0439156427423	0.367,0.0	0.0,0.0	0.184
6-8m_vs_8-10w	RI	Rit1	ENSMUSG00000028057.14	chr3	+	88725994	88726471	88725994	88726179	88726279	88726471	1024	21,16	10,8	7,6	18,11	248	149	0.000256288904031	0.0274982918207	0.558,0.546	0.189,0.247	0.334
6-8m_vs_8-10w	RI	Mroh1	ENSMUSG00000022558.16	chr15	+	76446515	76447365	76446515	76446681	76447168	76447365	1088	0,0	2,1	1,1	0,0	298	149	5.94589077973e-09	5.42265239111e-06	0.0,0.0	1.0,1.0	-1.0
6-8m_vs_8-10w	RI	Prmt5	ENSMUSG00000023110.12	chr14	-	54516105	54516615	54516105	54516191	54516496	54516615	1214	1,0	17,9	3,3	9,2	298	149	0.000903217324586	0.048790968483	0.029,0.0	0.143,0.429	-0.271
6-8m_vs_8-10w	RI	Rps18	ENSMUSG00000008668.14	chr17	-	33952220	33952591	33952220	33952293	33952402	33952591	1215	82,48	1,12	61,54	0,0	257	149	5.49601090641e-05	0.0091133853575 4	0.979,0.699	1.0,1.0	-0.161
6-8m_vs_8-10w	RI	Fxyd4	ENSMUSG00000004988.9	chr6	-	117935853	117936780	117935853	117935922	117936737	117936780	1368	1,2	1,7	1,4	0,1	298	149	0.000225245754598	0.0256780160242	0.333,0.125	1.0,0.667	-0.605
6-8m_vs_8-10w	RI	Trmt61a	ENSMUSG00000060950.12	chr12	+	111678104	111678962	111678104	111678161	111678602	111678962	1403	3,1	0,0	3,0	2,1	298	149	0.000500107049867	0.0396545597492	1.0,1.0	0.429,0.0	0.786
6-8m_vs_8-10w	RI	Xlr4b	ENSMUSG00000067768.12	chrX	+	73214773	73215355	73214773	73214856	73215227	73215355	1457	40,26	23,18	10,11	26,25	298	149	2.16488376837e-05	0.00564106856215	0.465,0.419	0.161,0.18	0.272
6-8m_vs_8-10w	RI	Xlr4b	ENSMUSG00000067768.12	chrX	+	73214773	73215355	73214773	73214820	73215240	73215355	1458	75,49	0,0	18,18	3,2	298	149	0.000461406534264	0.0382547962953	1.0,1.0	0.75,0.818	0.216
6-8m_vs_8-10w	RI	Lilra6	ENSMUSG00000030427.17	chr7	-	3911409	3912788	3911409	3911712	3912479	3912788	1621	3,3	6,2	0,0	1,4	298	149	0.000153828257608	0.0200416244198	0.2,0.429	0.0,0.0	0.315

6-8m_vs_8-10w	RI	Adrm1	ENSMUSG00000039041.15	chr2	+	180173909	180174946	180173909	180174033	180174864	180174946	1928	11,8	2,3	16,6	0,0	298	149	0.000105198793646	0.01476019997	0.733,0.571	1.0,1.0	-0.348
6-8m_vs_8-10w	RI	Rpl13a	ENSMUSG00000074129.13	chr7	-	45126990	45127274	45126990	45127087	45127208	45127274	1936	61,33	1,0	46,40	4,4	269	149	0.000918090207863	0.048790968483	0.971,1.0	0.864,0.847	0.13
6-8m_vs_8-10w	RI	Pusl1	ENSMUSG00000051557.15	chr4	-	155891051	155891415	155891051	155891239	155891357	155891415	2002	56,18	1,0	35,10	9,1	266	149	2.62353407645e-05	0.00598165769431	0.969,1.0	0.685,0.849	0.217
6-8m_vs_8-10w	RI	Slc25a39	ENSMUSG00000018677.9	chr11	-	102403632	102403872	102403632	102403713	102403790	102403872	2010	13,5	46,30	4,0	48,28	225	149	0.00096610950867	0.0489495484393	0.158,0.099	0.052,0.0	0.103
6-8m_vs_8-10w	RI	Xlr3b	ENSMUSG00000073125.10	chrX	+	73197352	73198385	73197352	73197470	73198285	73198385	2200	2,2	76,42	6,6	29,24	298	149	0.000412712822716	0.0376394094317	0.013,0.023	0.094,0.111	-0.085
6-8m_vs_8-10w	RI	Clk2	ENSMUSG00000068917.12	chr3	+	89168694	89170115	89168694	89168923	89170048	89170115	2284	1,1	0,0	0,0	3,3	298	149	3.78187814398e-09	5.42265239111e-06	1.0,1.0	0.0,0.0	1.0
6-8m_vs_8-10w	RI	Phkg2	ENSMUSG00000030815.11	chr7	+	127577534	127578029	127577534	127577688	127577974	127578029	2330	13,4	7,0	12,11	0,0	298	149	0.000367837829392	0.0353124316216	0.481,1.0	1.0,1.0	-0.26
6-8m_vs_8-10w	RI	Fam134a	ENSMUSG00000049339.16	chr1	+	75146429	75147909	75146429	75146719	75146830	75147909	2512	7,4	0,0	0,3	2,1	259	149	0.000541813602076	0.0396545597492	1.0,1.0	0.0,0.633	0.684
6-8m_vs_8-10w	RI	Tagln	ENSMUSG00000032085.5	chr9	-	45930832	45931477	45930832	45930935	45931299	45931477	2534	0,0	6,1	2,1	0,1	298	149	0.000555870683982	0.0396545597492	0.0,0.0	1.0,0.333	-0.667
6-8m_vs_8-10w	RI	Chchd2	ENSMUSG00000070493.3	chr5	-	129882428	129884183	129882428	129882579	129883933	129884183	2704	38,14	1605,1011	54,46	1538,952	298	149	0.000297485700988	0.0301452177001	0.012,0.007	0.017,0.024	-0.011
6-8m_vs_8-10w	RI	Tial1	ENSMUSG00000030846.15	chr7	-	128446730	128447107	128446730	128446798	128447038	128447107	2864	7,3	6,0	12,12	0,0	298	149	0.000818650168015	0.0481683195632	0.368,1.0	1.0,1.0	-0.316
6-8m_vs_8-10w	RI	Use1	ENSMUSG00000002395.14	chr8	+	71367643	71367848	71367643	71367693	71367769	71367848	2875	0,2	21,18	7,5	25,11	224	149	0.00067476812098	0.0439156427423	0.0,0.069	0.157,0.232	-0.16
6-8m_vs_8-10w	RI	Golga1	ENSMUSG00000026754.16	chr2	-	39047022	39047766	39047022	39047134	39047596	39047766	2904	3,1	10,9	5,9	3,6	298	149	0.000660319453672	0.0439156427423	0.13,0.053	0.455,0.429	-0.351
6-8m_vs_8-10w	RI	Raf1	ENSMUSG00000000441.17	chr6	-	115620208	115621862	115620208	115620327	115621815	115621862	2976	25,9	447,206	24,30	191,190	298	149	4.44279561992e-05	0.0090040657897	0.027,0.021	0.059,0.073	-0.042
6-8m_vs_8-10w	RI	Mrps10	ENSMUSG00000034729.16	chr17	+	47372426	47372645	47372426	47372491	47372575	47372645	2990	6,8	9,11	2,1	12,15	232	149	0.000936230206636	0.048790968483	0.3,0.318	0.097,0.041	0.24
6-8m_vs_8-10w	RI	Tra2a	ENSMUSG00000029817.11	chr6	-	49245475	49246964	49245475	49245610	49246848	49246964	3156	2,3	2,0	0,1	4,4	298	149	0.000451472256218	0.0382547962953	0.333,1.0	0.0,0.111	0.611
6-8m_vs_8-10w	RI	Alkbh6	ENSMUSG00000042831.13	chr7	+	30312236	30312707	30312236	30312388	30312505	30312707	3386	6,7	1,0	9,4	6,1	265	149	0.00072910703045	0.0443297074514	0.771,1.0	0.458,0.692	0.311
6-8m_vs_8-10w	RI	Alkbh6	ENSMUSG00000042831.13	chr7	+	30312236	30312707	30312236	30312388	30312590	30312707	3387	9,9	4,4	6,7	0,1	298	149	0.000565251399934	0.0396545597492	0.529,0.529	1.0,0.778	-0.36
6-8m_vs_8-10w	RI	Rnaseh2a	ENSMUSG00000052926.16	chr8	-	84957666	84958089	84957666	84957853	84957965	84958089	3479	42,13	0,0	17,18	5,5	260	149	6.41773047705e-07	0.000292648509753	1.0,1.0	0.661,0.674	0.333

6-8m_vs_8-10w	RI	Mrps24	ENSMUSG00000020477.10	chr11	-	5707297	5707555	5707297	5707409	5707486	5707555	3686	60,37	90,64	32,13	112,66	225	149	5.32957372401e-05	0.00911338535754	0.306,0.277	0.159,0.115	0.154
6-8m_vs_8-10w	RI	Tcf12	ENSMUSG00000032228.16	chr9	-	71868071	71870147	71868071	71868278	71870073	71870147	3746	1,2	0,0	2,4	3,4	298	149	2.89032186407e-07	0.000175731569335	1.0,1.0	0.25,0.333	0.709
6-8m_vs_8-10w	RI	Iws1	ENSMUSG00000024384.4	chr18	+	32086272	32087152	32086272	32086423	32087069	32087152	3769	22,16	138,172	4,6	173,167	298	149	1.44653750841e-05	0.00439747402557	0.074,0.044	0.011,0.018	0.045
6-8m_vs_8-10w	SE	Bcas1	ENSMUSG00000013523.13	chr2	-	170406273	170406848	170397531	170397714	170418606	170418655	113	13,8	2,4	11,2	0,0	298	149	2.53776710682e-05	0.00373866644049	0.765,0.5	1.0,1.0	-0.367
6-8m_vs_8-10w	SE	Tmem190	ENSMUSG00000013091.5	chr7	+	4783717	4783843	4783156	4783192	4784103	4784341	117	19,24	5,2	30,27	0,0	274	149	5.45537581953e-05	0.00663654734167	0.674,0.867	1.0,1.0	-0.23
6-8m_vs_8-10w	SE	Tmem190	ENSMUSG00000013091.5	chr7	+	4783933	4784018	4783156	4783192	4784103	4784341	118	4,2	5,2	7,7	0,0	233	149	2.28234611099e-07	0.000107793864266	0.338,0.39	1.0,1.0	-0.636
6-8m_vs_8-10w	SE	Pms2	ENSMUSG00000079109.11	chr5	+	143923476	143923632	143919508	143919608	143928098	143928266	235	10,3	1,0	0,2	1,2	298	149	4.90422715662e-05	0.00615250622508	0.833,1.0	0.0,0.333	0.75
6-8m_vs_8-10w	SE	Rhbdf1	ENSMUSG00000020282.18	chr11	-	32210826	32210902	32210397	32210551	32211110	32211139	285	3,1	3,2	8,2	0,0	224	149	1.17276792022e-06	0.000384332801284	0.399,0.25	1.0,1.0	-0.676
6-8m_vs_8-10w	SE	Nelfb	ENSMUSG00000013465.19	chr2	-	25204197	25204300	25203886	25203982	25206159	25206345	356	6,0	7,1	1,2	0,0	251	149	8.57358032347e-08	4.7473983736e-05	0.337,0.0	1.0,1.0	-0.832
6-8m_vs_8-10w	SE	Gpcpd1	ENSMUSG00000027346.15	chr2	-	132568619	132568716	132564626	132564711	132572184	132572254	403	448,407	5,12	377,280	20,15	245	149	0.00099323238399	0.0432817520274	0.982,0.954	0.92,0.919	0.048
6-8m_vs_8-10w	SE	Nek4	ENSMUSG00000021918.10	chr14	+	30957261	30957369	30956880	30957078	30959140	30959295	424	17,7	0,0	6,1	1,3	256	149	0.000339381758308	0.0220193627269	1.0,1.0	0.777,0.162	0.531
6-8m_vs_8-10w	SE	Rtbdn	ENSMUSG00000048617.16	chr8	+	84950501	84950676	84949859	84949956	84952643	84952788	433	6,2	0,0	0,0	4,1	298	149	5.7735816128e-11	3.09040578461e-07	1.0,1.0	0.0,0.0	1.0
6-8m_vs_8-10w	SE	RP24-534N1.1	ENSMUSG00000113017.1	chr13	+	5978082	5978217	5974340	5974480	6004940	6005008	442	25,24	0,7	12,8	0,0	283	149	0.0007242833787	0.034718037299	1.0,0.644	1.0,1.0	-0.178
6-8m_vs_8-10w	SE	Nin	ENSMUSG00000021068.16	chr12	-	70111435	70111482	70102650	70102854	70113473	70113717	482	4,2	7,8	0,0	5,1	195	149	0.000197971160509	0.0160023263425	0.304,0.16	0.0,0.0	0.232
6-8m_vs_8-10w	SE	Cdyl	ENSMUSG00000059288.14	chr13	+	35790100	35790175	35762811	35762922	35815908	35816090	496	4,9	0,0	3,2	2,2	223	149	9.8198496985e-05	0.00988634147075	1.0,1.0	0.501,0.401	0.549
6-8m_vs_8-10w	SE	Rnf182	ENSMUSG00000044164.2	chr13	+	43665757	43665822	43615982	43616067	43667759	43668066	505	2,4	0,0	0,2	3,1	213	149	5.51212810951e-06	0.00122935768309	1.0,1.0	0.0,0.583	0.709
6-8m_vs_8-10w	SE	Gm815	ENSMUSG00000074913.2	chr19	+	26886378	26886484	26885924	26885996	26887754	26887855	578	44,37	0,0	13,32	5,1	254	149	0.000357504721321	0.0229766750542	1.0,1.0	0.604,0.949	0.224
6-8m_vs_8-10w	SE	Myl3	ENSMUSG00000059741.13	chr9	+	110766620	110766770	110766478	110766506	110767907	110768081	643	2,9	3,1	17,3	0,0	298	149	0.000671486699039	0.0330133035861	0.25,0.818	1.0,1.0	-0.466
6-8m_vs_8-10w	SE	Aamp	ENSMUSG00000006299.13	chr1	-	74281735	74281819	74281059	74281642	74282026	74282171	760	45,26	8,5	55,34	2,0	232	149	0.000102341155694	0.0102392166862	0.783,0.77	0.946,1.0	-0.197

6-8m_vs_8-10w	SE	Fam179b	ENSMUSG00000035614.11	chr12	+	64970225	64970255	64966055	64968028	64976694	64976851	848	5,5	65,42	14,12	36,36	178	149	0.000378904140017	0.0238410125652	0.06,0.091	0.246,0.218	-0.156
6-8m_vs_8-10w	SE	RP23-47P3.3	ENSMUSG00000114188.1	chr12	+	19049552	19049690	19044705	19044820	19054983	19055179	862	12,8	0,1	14,4	6,3	286	149	0.000114727251504	0.0109660131229	1.0,0.806	0.549,0.41	0.424
6-8m_vs_8-10w	SE	Ythdf3	ENSMUSG00000047213.14	chr3	+	16189477	16189563	16183921	16183946	16213954	16215006	978	19,16	2,4	19,11	0,0	234	149	0.000191471598549	0.0158896688863	0.858,0.718	1.0,1.0	-0.212
6-8m_vs_8-10w	SE	Carkd	ENSMUSG00000031505.11	chr8	+	11511876	11511997	11510226	11510347	11512894	11513287	1176	11,6	4,7	1,0	8,2	269	149	2.3663608807e-05	0.00363627014567	0.604,0.322	0.065,0.0	0.431
6-8m_vs_8-10w	SE	Map4k1	ENSMUSG00000037337.11	chr7	+	28998451	28998528	28996088	28996173	28998612	28998673	1302	1,0	0,3	1,1	0,0	225	149	0.000561051492535	0.0296360686419	1.0,0.0	1.0,1.0	-0.5
6-8m_vs_8-10w	SE	RP23-326E2.4	ENSMUSG00000065999.13	chr4	+	147562441	147562521	147553338	147553426	147562914	147562958	1332	19,12	2,5	16,6	0,0	228	149	7.83542703452e-05	0.00856865451497	0.861,0.611	1.0,1.0	-0.264
6-8m_vs_8-10w	SE	Raver1	ENSMUSG00000010205.11	chr9	-	21079208	21079514	21076566	21076641	21086244	21086714	1380	12,2	9,2	5,2	0,0	298	149	1.29814351557e-06	0.00041691177146	0.4,0.333	1.0,1.0	-0.634
6-8m_vs_8-10w	SE	Gtpbp3	ENSMUSG00000007610.15	chr8	+	71489070	71489318	71488102	71488840	71489427	71489514	1652	3,8	6,11	16,7	0,1	298	149	5.60319619591e-08	3.39532545336e-05	0.2,0.267	1.0,0.778	-0.656
6-8m_vs_8-10w	SE	Csmd3	ENSMUSG00000022311.15	chr15	-	48292777	48292855	48285610	48287572	48359297	48359410	1733	3,2	0,0	1,0	1,1	226	149	0.00064608371676	0.0323066031958	1.0,1.0	0.397,0.0	0.802
6-8m_vs_8-10w	SE	Slc11a2	ENSMUSG00000023030.16	chr15	-	100403807	100403963	100403180	100403339	100405575	100405643	1742	37,8	0,0	20,9	2,3	298	149	0.000393628058627	0.0242644121514	1.0,1.0	0.833,0.6	0.284
6-8m_vs_8-10w	SE	Mrpl30	ENSMUSG00000026087.11	chr1	+	37892942	37893023	37890476	37890584	37893938	37893984	1747	63,43	0,1	43,28	7,1	229	149	0.00043847999942	0.025886440554	1.0,0.965	0.8,0.948	0.108
6-8m_vs_8-10w	SE	Ppfia1	ENSMUSG00000037519.17	chr7	-	144481084	144481250	144479026	144479097	144481710	144481779	1790	399,293	3,2	354,323	16,15	298	149	1.86315730655e-06	0.00053602275849	0.985,0.987	0.917,0.915	0.07
6-8m_vs_8-10w	SE	Slc25a36	ENSMUSG00000032449.13	chr9	-	97090092	97090174	97085056	97085123	97093076	97093177	1855	14,7	222,144	2,1	190,155	230	149	0.000490592467577	0.0274971512892	0.039,0.031	0.007,0.004	0.03
6-8m_vs_8-10w	SE	Tm9sf1	ENSMUSG00000002320.15	chr14	-	55637909	55638132	55635967	55636612	55640384	55640570	1868	0,1	1,1	2,3	0,0	298	149	0.000271237360505	0.0194260017558	0.0,0.333	1.0,1.0	-0.834
6-8m_vs_8-10w	SE	Bclaf1	ENSMUSG00000037608.16	chr10	+	20334498	20334645	20333253	20333572	20339708	20339921	1888	179,216	84,106	209,228	189,173	295	149	0.000294362623554	0.0204141055594	0.518,0.507	0.358,0.4	0.133
6-8m_vs_8-10w	SE	Ell3	ENSMUSG00000027246.12	chr2	-	121440115	121440158	121439847	121440019	121440233	121440426	1956	14,8	5,3	15,8	0,0	191	149	1.23665315056e-05	0.00227436359863	0.686,0.675	1.0,1.0	-0.319
6-8m_vs_8-10w	SE	Pnisr	ENSMUSG00000028248.15	chr4	+	21861986	21862177	21847582	21848139	21865876	21866014	2079	39,21	6,0	28,28	0,0	298	149	0.000842408786792	0.0383957135466	0.765,1.0	1.0,1.0	-0.117
6-8m_vs_8-10w	SE	Bphl	ENSMUSG00000038286.11	chr13	+	34046785	34046952	34044423	34044527	34050280	34050323	2350	9,7	0,0	2,5	1,3	298	149	6.14041868443e-05	0.0071193388617	1.0,1.0	0.5,0.455	0.523
6-8m_vs_8-10w	SE	Alg9	ENSMUSG00000032059.13	chr9	+	50783257	50783328	50778969	50779104	50788152	50788241	2437	6,2	1,3	0,0	2,2	219	149	5.289067457e-06	0.00119622317218	0.803,0.312	0.0,0.0	0.558

6-8m_vs_8-10w	SE	Mkrn2	ENSMUSG00000000439.9	chr6	+	115611634	115611939	115610518	115610700	115613326	115613541	2468	299,115	8,1	151,114	10,12	298	149	0.000387563184663	0.024108677136	0.949,0.983	0.883,0.826	0.111
6-8m_vs_8-10w	SE	Exoc3l2	ENSMUSG00000011263.16	chr7	+	19489607	19489763	19489051	19489174	19491655	19491777	2583	443,149	130,52	426,234	56,37	298	149	4.24851969028e-06	0.00101824968935	0.63,0.589	0.792,0.76	-0.167
6-8m_vs_8-10w	SE	Med13	ENSMUSG00000034297.14	chr11	-	86306983	86307062	86303533	86303655	86308698	86308912	2603	30,11	0,0	17,8	0,7	227	149	0.00117239795696	0.0483347019072	1.0,1.0	1.0,0.429	0.286
6-8m_vs_8-10w	SE	Fam49a	ENSMUSG00000020589.17	chr12	+	12358044	12358166	12340678	12340758	12359212	12359318	2611	40,34	9,10	34,34	2,1	270	149	0.000261224192855	0.0190190497563	0.71,0.652	0.904,0.949	-0.245
6-8m_vs_8-10w	SE	Smim8	ENSMUSG00000028295.14	chr4	-	34771873	34772053	34771257	34771414	34778223	34778337	2627	8,2	5,2	11,7	0,0	298	149	2.76446335712e-07	0.000126833578825	0.444,0.333	1.0,1.0	-0.611
6-8m_vs_8-10w	SE	Smim8	ENSMUSG00000028295.14	chr4	-	34771877	34772053	34770887	34771414	34778223	34778270	2629	5,3	5,2	2,1	0,0	298	149	4.8053737467e-07	0.000192911729061	0.333,0.429	1.0,1.0	-0.619
6-8m_vs_8-10w	SE	RP23-128E7.2	ENSMUSG00000108981.1	chrX	+	161300839	161301022	161287455	161287576	161307692	161307699	2633	25,10	0,0	13,6	4,1	298	149	0.000117315102896	0.0111470172917	1.0,1.0	0.619,0.75	0.316
6-8m_vs_8-10w	SE	Esrp1	ENSMUSG00000040728.15	chr4	-	11353294	11353463	11331932	11333473	11359737	11359956	2708	99,73	1,0	81,100	6,8	298	149	2.61522448799e-05	0.00383518491581	0.98,1.0	0.871,0.862	0.123
6-8m_vs_8-10w	SE	Esrp1	ENSMUSG00000040728.15	chr4	-	11357459	11357658	11353294	11353463	11359737	11359956	2710	180,135	0,2	149,146	8,5	298	149	0.00045246597245	0.0261826978832	1.0,0.971	0.903,0.936	0.066
6-8m_vs_8-10w	SE	Esrp1	ENSMUSG00000040728.15	chr4	-	11357471	11357658	11353294	11353463	11359737	11359956	2711	82,59	0,2	71,83	8,5	298	149	0.000599870530731	0.0306774553582	1.0,0.937	0.816,0.892	0.115
6-8m_vs_8-10w	SE	Asxl2	ENSMUSG00000037486.18	chr12	+	3445451	3445454	3442473	3442556	3451661	3451770	2827	1,1	40,12	10,5	26,22	151	149	0.000108655258816	0.0107135826724	0.024,0.076	0.275,0.183	-0.179
6-8m_vs_8-10w	SE	Gadd45a	ENSMUSG00000036390.8	chr6	-	67036677	67036915	67035095	67035772	67037200	67037364	2838	53,35	8,0	44,33	0,0	298	149	0.00040631911905	0.0247615651374	0.768,1.0	1.0,1.0	-0.116
6-8m_vs_8-10w	SE	Gadd45a	ENSMUSG00000036390.8	chr6	-	67037009	67037111	67035465	67035772	67037200	67037251	2839	13,10	8,0	6,5	0,0	250	149	0.000512169114075	0.0281188657677	0.492,1.0	1.0,1.0	-0.254
6-8m_vs_8-10w	SE	Tbp	ENSMUSG00000014767.16	chr17	+	15514229	15514397	15513529	15513621	15515615	15515710	2947	840,609	24,3	720,610	51,29	298	149	3.04519769956e-07	0.000135832735165	0.946,0.99	0.876,0.913	0.073
6-8m_vs_8-10w	SE	Sct	ENSMUSG00000038580.13	chr7	-	141278617	141278934	141278329	141278521	141279015	141279123	3165	0,3	2,2	7,2	0,0	298	149	2.80968264499e-06	0.000719311342878	0.0,0.429	1.0,1.0	-0.786
6-8m_vs_8-10w	SE	Rad18	ENSMUSG00000030254.16	chr6	-	112644587	112644747	112619849	112620904	112665277	112665338	3168	12,14	0,0	11,5	4,1	298	149	6.57677011864e-05	0.0074900549337	1.0,1.0	0.579,0.714	0.354
6-8m_vs_8-10w	SE	Tdp2	ENSMUSG00000035958.3	chr13	+	24832009	24832095	24831678	24831929	24840387	24840558	3262	47,45	9,14	47,28	27,27	234	149	0.00108938803648	0.0458564851173	0.769,0.672	0.526,0.398	0.259
6-8m_vs_8-10w	SE	Tdp2	ENSMUSG00000035958.3	chr13	+	24836015	24836186	24831680	24831929	24836854	24836944	3266	248,163	27,25	136,142	42,45	298	149	2.12173395542e-05	0.00335672944395	0.821,0.765	0.618,0.612	0.178
6-8m_vs_8-10w	SE	Tdp2	ENSMUSG00000035958.3	chr13	+	24838146	24838265	24831678	24831929	24840387	24840558	3274	315,197	9,14	214,174	27,27	267	149	1.7175479072e-05	0.00288799835537	0.951,0.887	0.816,0.782	0.12

6-8m_vs_8-10w	SE	Tdp2	ENSMUSG00000035958.3	chr13	+	24838146	24838265	24836854	24836946	24840387	24840558	3276	599,388	34,38	435,352	69,63	267	149	4.82527773571e-06	0.00109906822525	0.908,0.851	0.779,0.757	0.111
6-8m_vs_8-10w	SE	Timd2	ENSMUSG00000040413.15	chr11	-	46679648	46679717	46668959	46671018	46686917	46687253	3289	2,2	1,0	0,1	1,3	217	149	9.77393761092e-06	0.00190242291098	0.579,1.0	0.0,0.186	0.697
6-8m_vs_8-10w	SE	AC123856.3	ENSMUSG00000079455.9	chr1	+	85281484	85281562	85279831	85279909	85282728	85282809	3367	191,121	8,15	129,95	2,2	226	149	0.000839487020251	0.0383513017673	0.94,0.842	0.977,0.969	-0.082
6-8m_vs_8-10w	SE	P4ha2	ENSMUSG00000018906.14	chr11	+	54126345	54126411	54125762	54125816	54129047	54129116	3388	8,3	0,0	9,7	1,4	214	149	0.000295593759718	0.0204141055594	1.0,1.0	0.862,0.549	0.295
6-8m_vs_8-10w	SE	Commd10	ENSMUSG00000042705.8	chr18	+	46967739	46967895	46963659	46963770	46990478	46990589	3589	145,65	8,4	60,59	11,18	298	149	6.43631766556e-05	0.00736284893711	0.901,0.89	0.732,0.621	0.219
6-8m_vs_8-10w	SE	Cr1l	ENSMUSG00000016481.15	chr1	-	195106817	195106922	195097381	195098841	195111079	195111155	3661	11,7	3,4	4,3	0,0	253	149	7.91786354537e-06	0.00163006477964	0.683,0.508	1.0,1.0	-0.404
6-8m_vs_8-10w	SE	Aptx	ENSMUSG00000028411.15	chr4	-	40695376	40695418	40694851	40695136	40697216	40697353	3702	30,12	1,0	22,7	8,1	190	149	9.41046111032e-05	0.0095723350821	0.959,1.0	0.683,0.846	0.215
6-8m_vs_8-10w	SE	Aptx	ENSMUSG00000028411.15	chr4	-	40695376	40695423	40694851	40695136	40697216	40697353	3703	38,21	1,0	41,21	8,1	195	149	0.000656172971426	0.03252106659	0.967,1.0	0.797,0.941	0.115
6-8m_vs_8-10w	SE	Zscan29	ENSMUSG00000050619.14	chr2	-	121169198	121169403	121166043	121166718	121169809	121170268	3736	20,4	1,0	8,5	1,6	298	149	0.000372887493964	0.023714167834	0.909,1.0	0.8,0.294	0.408
6-8m_vs_8-10w	SE	Dip2b	ENSMUSG00000023026.16	chr15	+	100151150	100151363	100142021	100142147	100154156	100154306	3897	14,11	0,0	7,2	2,2	298	149	0.000271586585763	0.0194260017558	1.0,1.0	0.636,0.333	0.516
6-8m_vs_8-10w	SE	RP24-238M4.4	ENSMUSG00000110685.1	chr8	-	71830411	71830549	71825777	71826180	71831934	71831977	3977	308,171	2,2	285,188	16,9	286	149	1.23930013003e-05	0.00227436359863	0.988,0.978	0.903,0.916	0.074
6-8m_vs_8-10w	SE	Hmg20b	ENSMUSG00000020232.17	chr10	-	81346840	81347056	81346047	81346477	81347435	81347508	4016	6,0	1,3	5,5	0,0	298	149	5.35868850198e-05	0.006593855936	0.75,0.0	1.0,1.0	-0.625
6-8m_vs_8-10w	SE	Acbd5	ENSMUSG00000026781.16	chr2	+	23089590	23089788	23069326	23069619	23094288	23094407	4048	0,0	1,2	4,1	0,0	298	149	1.7239969452e-09	2.516722086e-06	0.0,0.0	1.0,1.0	-1.0
6-8m_vs_8-10w	SE	Ankhd1	ENSMUSG00000024483.18	chr18	+	36641398	36641505	36640264	36640360	36641576	36641693	4213	2,8	15,6	0,0	14,8	255	149	8.65553381793e-05	0.00908435046067	0.072,0.438	0.0,0.0	0.255
6-8m_vs_8-10w	SE	Ttc19	ENSMUSG00000042298.18	chr11	+	62284130	62284169	62283596	62283707	62285002	62285059	4250	7,6	3,4	13,5	1,0	187	149	0.000198030551928	0.0160023263425	0.65,0.544	0.912,1.0	-0.359
6-8m_vs_8-10w	SE	Parp3	ENSMUSG00000023249.15	chr9	-	106471759	106471915	106470321	106471402	106473432	106473519	4334	3,4	0,0	0,1	2,0	298	149	0.00119563857489	0.048483765083	1.0,1.0	0.0,1.0	0.5
6-8m_vs_8-10w	SE	Nat9	ENSMUSG00000015542.17	chr11	-	115183624	115183684	115183354	115183449	115184482	115184626	4514	11,0	1,3	5,3	0,0	208	149	0.00106478366397	0.0451591027273	0.887,0.0	1.0,1.0	-0.557
6-8m_vs_8-10w	SE	Ydjc	ENSMUSG00000041774.15	chr16	+	17147796	17147974	17147597	17147697	17148155	17148857	4663	0,1	8,3	6,3	3,1	298	149	1.80018362429e-05	0.00299308453822	0.0,0.143	0.5,0.6	-0.479
6-8m_vs_8-10w	SE	Fam192a	ENSMUSG00000031774.8	chr8	-	94597050	94597108	94589310	94589407	94601681	94601862	4685	3,4	1,0	1,5	2,4	206	149	0.000738710112525	0.0351994272609	0.685,1.0	0.266,0.475	0.472

6-8m_vs_8-10w	SE	Cbx7	ENSMUSG00000053411.16	chr15	-	79918276	79918476	79915806	79918050	79921371	79921438	4727	2,5	1,0	1,1	2,1	298	149	0.000205361528816	0.016406444924	0.5,1.0	0.2,0.333	0.484
6-8m_vs_8-10w	SE	Cbx7	ENSMUSG00000053411.16	chr15	-	79918752	79918927	79918416	79918476	79921371	79921438	4729	15,4	1,4	11,8	0,0	298	149	0.000252491218315	0.0185986421271	0.882,0.333	1.0,1.0	-0.392
6-8m_vs_8-10w	SE	Cbx7	ENSMUSG00000053411.16	chr15	-	79918752	79919104	79915809	79918476	79921371	79921438	4730	11,6	1,4	16,9	0,0	298	149	0.000132596948664	0.0121324319182	0.846,0.429	1.0,1.0	-0.363
6-8m_vs_8-10w	SE	Dhx40	ENSMUSG00000018425.18	chr11	-	86789166	86789349	86785756	86785837	86789485	86789572	4757	152,117	0,0	101,97	4,7	298	149	3.5688379972e-05	0.00485766165367	1.0,1.0	0.927,0.874	0.099
6-8m_vs_8-10w	SE	Atpif1	ENSMUSG00000054428.12	chr4	-	132533295	132533387	132530554	132530823	132533481	132533659	4773	834,952	116,124	892,531	51,51	240	149	8.17009459158e-05	0.00881911409954	0.817,0.827	0.916,0.866	-0.069
6-8m_vs_8-10w	SE	Lrr1	ENSMUSG00000034883.9	chr12	+	69171861	69171960	69168813	69169062	69174391	69175113	4780	822,694	4,4	805,588	21,9	247	149	0.000140529943604	0.0127493210983	0.992,0.991	0.959,0.975	0.025
6-8m_vs_8-10w	SE	Pik3r3	ENSMUSG00000028698.13	chr4	+	116292096	116292267	116291674	116291749	116299633	116299841	4867	47,37	0,1	32,14	5,2	298	149	0.000237845688566	0.0178891150679	1.0,0.949	0.762,0.778	0.204
6-8m_vs_8-10w	SE	Pik3c2a	ENSMUSG00000030660.9	chr7	-	116442590	116442678	116417452	116418581	116443303	116443449	4937	251,147	42,19	132,127	45,36	236	149	0.000641084437339	0.0321558013852	0.79,0.83	0.649,0.69	0.141
6-8m_vs_8-10w	SE	Chn1	ENSMUSG00000056486.18	chr2	-	73679614	73679903	73659679	73659757	73706918	73707032	5069	223,173	13,1	130,164	0,1	298	149	0.000509998704962	0.0280945427248	0.896,0.989	1.0,0.988	-0.051
6-8m_vs_8-10w	SE	Rprd1a	ENSMUSG00000040446.3	chr18	-	24508169	24508276	24484961	24488331	24529915	24530204	5089	18,7	1,6	21,17	0,0	255	149	8.54379071681e-05	0.00905585421324	0.913,0.405	1.0,1.0	-0.341
6-8m_vs_8-10w	SE	Sepw1	ENSMUSG00000041571.9	chr7	-	15920304	15920358	15920042	15920117	15920448	15920473	5129	1412,857	23,24	663,646	7,3	202	149	0.00113739946595	0.0475015880994	0.978,0.963	0.986,0.994	-0.02
6-8m_vs_8-10w	SE	Tex30	ENSMUSG00000026049.11	chr1	-	44091360	44091591	44088475	44088527	44099847	44099921	5139	1366,1131	52,52	1091,953	102,77	298	149	3.62511188945e-06	0.000875369123621	0.929,0.916	0.842,0.861	0.071
6-8m_vs_8-10w	SE	Lrrfip1	ENSMUSG00000026305.15	chr1	+	91110354	91110447	91107297	91107390	91112155	91112297	5167	4,2	3,11	0,0	6,10	241	149	0.000332068872784	0.0218092513667	0.452,0.101	0.0,0.0	0.277
6-8m_vs_8-10w	SE	Pold3	ENSMUSG00000030726.16	chr7	-	100096372	100096535	100089651	100089755	100100419	100100681	5349	0,0	12,3	4,4	5,10	298	149	9.14368750161e-05	0.00941200160701	0.0,0.0	0.286,0.167	-0.226
6-8m_vs_8-10w	SE	Ovgp1	ENSMUSG00000074340.9	chr3	+	105979897	105980006	105978273	105978398	105984572	105984814	5375	4,0	1,2	5,5	1,0	257	149	0.00115648912851	0.0479868279732	0.699,0.0	0.744,1.0	-0.523
6-8m_vs_8-10w	SE	Ovgp1	ENSMUSG00000074340.9	chr3	+	105981232	105981349	105980091	105980277	105984572	105984814	5377	2,5	0,0	6,6	3,1	265	149	0.00111716481639	0.0467781815426	1.0,1.0	0.529,0.771	0.35
6-8m_vs_8-10w	SE	Wnk2	ENSMUSG00000037989.15	chr13	-	49050483	49050582	49044373	49044418	49051356	49051527	5382	7,2	0,0	0,0	2,2	247	149	1.01798680596e-10	4.38162262584e-07	1.0,1.0	0.0,0.0	1.0
6-8m_vs_8-10w	SE	Eef1kmt4	ENSMUSG00000115219.1	chr16	+	20617625	20617909	20611592	20611820	20618389	20619007	5423	341,272	7,5	383,225	33,12	298	149	1.4444957643e-05	0.00256306220808	0.961,0.965	0.853,0.904	0.084
6-8m_vs_8-10w	SE	Dydc1	ENSMUSG00000021790.12	chr14	+	41086353	41086413	41082271	41082361	41087384	41087489	5585	7,9	2,8	10,5	0,1	208	149	0.000293099218548	0.0204141055594	0.715,0.446	1.0,0.782	-0.311

6-8m_vs_8-10w	SE	Eif4a2	ENSMUSG00000022884.14	chr16	+	23111125	23111263	23110579	23110723	23111502	23111592	5602	1234,740	15,32	941,717	6,1	286	149	3.7837229061e-07	0.000155792365195	0.977,0.923	0.988,0.997	-0.042
6-8m_vs_8-10w	SE	Rce1	ENSMUSG00000024889.4	chr19	-	4625012	4625096	4624176	4624763	4625192	4625276	5629	10,2	5,0	13,3	0,0	232	149	0.0010075536189 4	0.0436470183008	0.562,1.0	1.0,1.0	-0.219
6-8m_vs_8-10w	SE	RP23-67E6.3	ENSMUSG00000078867.9	chr2	-	177393293	177393395	177389426	177389553	177394696	177394751	5682	6,13	2,4	21,15	1,0	250	149	0.000280624799146	0.0198514230162	0.641,0.66	0.926,1.0	-0.313
6-8m_vs_8-10w	SE	Tdh	ENSMUSG00000021953.14	chr14	-	63494356	63494422	63493741	63494011	63495719	63495892	5699	5,3	4,1	7,2	0,0	214	149	2.30699119497e-05	0.0035620831354 6	0.465,0.676	1.0,1.0	-0.43
6-8m_vs_8-10w	SE	Rnf14	ENSMUSG00000060450.13	chr18	+	38297499	38297575	38296634	38296798	38299753	38299926	5890	13,6	75,22	13,16	24,18	224	149	0.000620867642309	0.0314012365361	0.103,0.154	0.265,0.372	-0.19
6-8m_vs_8-10w	SE	P4ha1	ENSMUSG00000019916.14	chr10	+	59354339	59354410	59352082	59352259	59355349	59355420	5944	13,2	15,17	1,0	15,5	219	149	0.000381996151296	0.0239145972613	0.371,0.074	0.043,0.0	0.201
6-8m_vs_8-10w	SE	Mllt10	ENSMUSG00000026743.16	chr2	+	18126133	18126229	18123708	18123812	18146776	18146872	6033	99,107	17,5	110,99	1,2	244	149	3.36768660919e-05	0.0047437115412 6	0.781,0.929	0.985,0.968	-0.121
6-8m_vs_8-10w	SE	Cox18	ENSMUSG00000035505.14	chr5	-	90219359	90219523	90218911	90219033	90222230	90222331	6177	213,103	0,2	94,93	8,4	298	149	6.03043566587e-05	0.0070942663679 5	1.0,0.963	0.855,0.921	0.094
6-8m_vs_8-10w	SE	Map3k7	ENSMUSG00000028284.13	chr4	+	31994873	31994954	31992386	31992516	32002088	32002153	6305	7,6	4,0	1,3	6,11	229	149	0.000282479657755	0.0199387179966	0.532,1.0	0.098,0.151	0.642
6-8m_vs_8-10w	SE	Ankrd7	ENSMUSG00000029517.13	chr6	+	18869893	18870031	18869232	18869339	18879340	18879582	6308	7,1	1,3	1,2	0,0	286	149	0.000295188794894	0.0204141055594	0.785,0.148	1.0,1.0	-0.534
6-8m_vs_8-10w	SE	Cpsf6	ENSMUSG00000055531.12	chr10	-	117361773	117361884	117360855	117361360	117362974	117363120	6319	6,3	15,6	0,1	13,3	259	149	0.0011948482698 6	0.048483765083	0.187,0.223	0.0,0.161	0.125
6-8m_vs_8-10w	SE	Bola1	ENSMUSG00000015943.11	chr3	-	96198164	96198226	96196587	96197279	96198436	96198489	6331	0,1	2,1	2,1	1,0	210	149	0.000113900191466	0.0109273757275	0.0,0.415	0.587,1.0	-0.586
6-8m_vs_8-10w	SE	Gnpnat1	ENSMUSG00000037722.8	chr14	-	45380936	45381016	45378163	45378486	45381555	45381683	6409	252,168	2,1	185,124	11,7	228	149	1.64451304469e-05	0.0028093181352 8	0.988,0.991	0.917,0.92	0.071
6-8m_vs_8-10w	SE	Zfp141	ENSMUSG00000092416.2	chr7	-	42502873	42502941	42501357	42501425	42505609	42505725	6503	0,0	9,2	2,5	1,2	216	149	1.02435262683e-06	0.000357588140905	0.0,0.0	0.58,0.633	-0.607
6-8m_vs_8-10w	SE	Comtd1	ENSMUSG00000021773.11	chr14	-	21847613	21847747	21845854	21847427	21847872	21847927	6520	4,2	2,1	7,8	0,0	282	149	0.000749428542883	0.0356045075196	0.514,0.514	1.0,1.0	-0.486
6-8m_vs_8-10w	SE	Rad9b	ENSMUSG00000038569.13	chr5	-	122333295	122333415	122331468	122331697	122334409	122334516	6579	38,14	0,0	20,21	8,1	268	149	3.94512583509e-05	0.0053013247414 1	1.0,1.0	0.582,0.921	0.248
6-8m_vs_8-10w	SE	Rad9b	ENSMUSG00000038569.13	chr5	-	122334261	122334326	122331468	122331697	122334409	122334516	6580	5,12	0,0	3,4	8,1	213	149	2.24892829537e-06	0.000617321206274	1.0,1.0	0.208,0.737	0.528
6-8m_vs_8-10w	SE	Nipsnap1	ENSMUSG00000034285.15	chr11	+	4889096	4889237	4884021	4884116	4889532	4889564	6656	29,21	4,4	19,16	0,0	289	149	5.3372532286e-05	0.0065927394111 4	0.789,0.73	1.0,1.0	-0.24
6-8m_vs_8-10w	SE	Nipsnap1	ENSMUSG00000034285.15	chr11	+	4891402	4891486	4889891	4889986	4893119	4893617	6658	32,30	10,8	37,35	0,0	232	149	1.17827100299e-08	9.22959793464e-06	0.673,0.707	1.0,1.0	-0.31

6-8m_vs_8-10w	SE	Cdk10	ENSMUSG00000033862.7	chr8	+	123227659	123227741	123226945	123227017	123228328	123228396	6696	16,15	12,8	7,5	0,1	230	149	0.000161645280403	0.014030810339	0.463,0.548	1.0,0.764	-0.376
6-8m_vs_8-10w	SE	Anapc4	ENSMUSG00000029176.13	chr5	+	52842820	52842905	52842023	52842068	52843486	52843591	6762	39,40	8,1	15,25	0,0	233	149	0.000375597959802	0.0237721098649	0.757,0.962	1.0,1.0	-0.141
6-8m_vs_8-10w	SE	Lrrc28	ENSMUSG00000030556.13	chr7	-	67579689	67579750	67545365	67545468	67617992	67618130	6785	1,0	17,9	4,3	5,12	209	149	0.000509824860007	0.0280945427248	0.04,0.0	0.363,0.151	-0.237
6-8m_vs_8-10w	SE	Zfp708	ENSMUSG00000058883.16	chr13	-	67074832	67074916	67069398	67071528	67097792	67097976	6846	10,5	5,3	5,5	0,1	232	149	0.000768314934393	0.0361672891129	0.562,0.517	1.0,0.763	-0.342
6-8m_vs_8-10w	SE	Zfp708	ENSMUSG00000058883.16	chr13	-	67074832	67074959	67069398	67071528	67097792	67097976	6847	22,11	5,3	10,20	0,1	275	149	0.000455461387741	0.0263086293681	0.704,0.665	1.0,0.916	-0.273
6-8m_vs_8-10w	SE	Nolc1	ENSMUSG00000015176.10	chr19	+	46077675	46077783	46076053	46076149	46078702	46079021	6858	1,3	146,118	14,9	144,97	256	149	4.8816832631e-05	0.00614824077168	0.004,0.015	0.054,0.051	-0.043
6-8m_vs_8-10w	SE	Irx2	ENSMUSG00000001504.10	chr13	+	72631256	72631970	72630570	72630976	72632585	72632661	6870	2,1	1,2	4,1	0,0	298	149	0.000676992722137	0.0331437473539	0.5,0.2	1.0,1.0	-0.65
6-8m_vs_8-10w	SE	RP23-302F9.3	ENSMUSG00000098257.1	chr8	+	87284871	87285123	87215940	87216004	87287035	87287139	6929	4,0	2,7	2,2	0,0	298	149	5.15566067616e-09	5.18981436546e-06	0.5,0.0	1.0,1.0	-0.75
6-8m_vs_8-10w	SE	Troap	ENSMUSG00000032783.9	chr15	+	99082908	99083102	99082125	99082705	99083227	99083403	6946	27,22	2,8	25,15	0,0	298	149	2.03745235088e-05	0.00328818189452	0.871,0.579	1.0,1.0	-0.275
6-8m_vs_8-10w	SE	Nipa2	ENSMUSG00000030452.16	chr7	-	55942979	55943036	55937571	55937662	55944480	55944709	6977	107,62	7,14	95,74	0,3	205	149	4.82633677359e-05	0.00612658623797	0.917,0.763	1.0,0.947	-0.133
6-8m_vs_8-10w	SE	Nipa2	ENSMUSG00000030452.16	chr7	-	55942979	55943036	55937571	55937662	55962199	55962439	6978	37,21	0,1	23,31	7,2	205	149	0.000392699099362	0.0242537005291	1.0,0.939	0.705,0.918	0.158
6-8m_vs_8-10w	SE	Nipa2	ENSMUSG00000030452.16	chr7	-	55944480	55944709	55937571	55937662	55962199	55962439	6981	22,25	0,1	13,12	7,2	298	149	2.86247491785e-05	0.00412247733012	1.0,0.926	0.481,0.75	0.348
6-8m_vs_8-10w	SE	Nav2	ENSMUSG00000052512.17	chr7	+	49552839	49552905	49551596	49552049	49556909	49557070	6999	0,0	13,6	3,3	6,1	214	149	2.40166253181e-05	0.00365677963838	0.0,0.0	0.258,0.676	-0.467
6-8m_vs_8-10w	SE	Lrrc6	ENSMUSG00000022375.6	chr15	-	66469816	66469989	66449442	66449634	66481240	66481318	7186	21,9	0,0	16,7	3,2	298	149	0.00029247276414	0.0204141055594	1.0,1.0	0.727,0.636	0.319
6-8m_vs_8-10w	SE	Ctps	ENSMUSG00000028633.7	chr4	-	120562777	120562878	120561397	120561514	120565268	120565439	7309	347,171	8,3	143,101	13,8	249	149	0.000217493450191	0.0169484375579	0.963,0.972	0.868,0.883	0.092
6-8m_vs_8-10w	SE	Dcun1d2	ENSMUSG00000038506.13	chr8	-	13261384	13261467	13255962	13257976	13271589	13271720	7366	4,4	4,7	1,0	8,7	231	149	0.000216461046724	0.0169484375579	0.392,0.269	0.075,0.0	0.293
6-8m_vs_8-10w	SE	Ppat	ENSMUSG00000029246.14	chr5	-	76949440	76949596	76925673	76925880	76950956	76951572	7403	1,1	0,0	0,1	3,0	298	149	0.00024621179249	0.0183464917114	1.0,1.0	0.0,1.0	0.5
6-8m_vs_8-10w	SE	Tmf1	ENSMUSG00000030059.15	chr6	-	97175769	97176968	97173299	97173403	97178881	97179037	7405	41,40	0,0	25,50	0,8	298	149	0.000256559163883	0.0187264866074	1.0,1.0	1.0,0.758	0.121
6-8m_vs_8-10w	SE	AC159266.3	ENSMUSG00000008129.14	chr8	+	84166580	84167690	84161212	84161291	84170354	84170452	7439	8,9	0,0	3,6	2,3	298	149	1.53565794917e-05	0.00269503774293	1.0,1.0	0.429,0.5	0.536

6-8m_vs_8-10w	SE	RP24-336D11.5	ENSMUSG00000110369.1	chr8	-	34421936	34421984	34419236	34419494	34428372	34428505	7501	5,1	0,0	0,3	1,4	196	149	1.1107167317e-06	0.0003753365124 5	1.0,1.0	0.0,0.363	0.819
6-8m_vs_8-10w	SE	Acsbg2	ENSMUSG00000024207.8	chr17	-	56845403	56845513	56843102	56843377	56853736	56853913	7575	10,3	2,1	2,0	3,2	258	149	0.000672272404574	0.0330133035861	0.743,0.634	0.278,0.0	0.55
6-8m_vs_8-10w	SE	Acsbg2	ENSMUSG00000024207.8	chr17	-	56846092	56846339	56845403	56845513	56846521	56846661	7576	2,1	0,0	0,1	3,1	298	149	8.49017656179e-09	7.36947325563e-06	1.0,1.0	0.0,0.333	0.834
6-8m_vs_8-10w	SE	Kif20b	ENSMUSG00000024795.11	chr19	+	34936776	34936848	34935625	34935737	34936937	34936942	7604	523,440	2,1	353,393	4,15	220	149	0.000294705478329	0.0204141055594	0.994,0.997	0.984,0.947	0.03
6-8m_vs_8-10w	SE	Scamp3	ENSMUSG00000028049.15	chr3	+	89181925	89182043	89181174	89181276	89182298	89182763	7670	130,45	41,13	75,54	11,3	266	149	0.0010475718309 1	0.044798690974	0.64,0.66	0.792,0.91	-0.201
6-8m_vs_8-10w	SE	Lman2l	ENSMUSG00000001143.13	chr1	-	36438749	36438832	36428299	36428461	36439608	36439668	7756	11,1	4,1	13,1	0,0	231	149	8.15427643019e-05	0.0088191140995 4	0.639,0.392	1.0,1.0	-0.484
6-8m_vs_8-10w	SE	Lman2l	ENSMUSG00000001143.13	chr1	-	36444036	36444142	36443519	36443609	36445049	36445271	7757	10,2	2,2	1,1	0,0	254	149	0.0012229053671 7	0.0492165774086	0.746,0.37	1.0,1.0	-0.442
6-8m_vs_8-10w	SE	Kcnu1	ENSMUSG00000031576.16	chr8	+	25911959	25912018	25910849	25910946	25913583	25913808	7773	4,2	0,0	0,2	3,3	207	149	2.50294718285e-09	3.04631551948e-06	1.0,1.0	0.0,0.324	0.838
6-8m_vs_8-10w	SE	Kcnu1	ENSMUSG00000031576.16	chr8	+	25932239	25932434	25921419	25921559	25934404	25934517	7776	4,5	2,3	1,0	2,3	298	149	0.000323792625886	0.0214715690827	0.5,0.455	0.2,0.0	0.378
6-8m_vs_8-10w	SE	Alkbh8	ENSMUSG00000025899.14	chr9	+	3349419	3349490	3345780	3345876	3359483	3359590	7933	10,14	0,0	7,8	5,1	219	149	3.45820585425e-05	0.0048115829942 2	1.0,1.0	0.488,0.845	0.334
6-8m_vs_8-10w	SE	Chd9	ENSMUSG00000056608.14	chr8	+	90871370	90871602	90849666	90849759	90890976	90891521	7948	11,2	7,0	1,0	2,3	298	149	0.000447373017716	0.0260759198493	0.44,1.0	0.2,0.0	0.62
6-8m_vs_8-10w	SE	Ice2	ENSMUSG00000032235.15	chr9	+	69398945	69399087	69397905	69398053	69400476	69400581	7970	20,34	1,7	13,21	0,0	290	149	0.000110789514069	0.0108149423521	0.911,0.714	1.0,1.0	-0.188
6-8m_vs_8-10w	SE	Dcaf8	ENSMUSG00000026554.15	chr1	+	172188938	172189069	172187352	172187460	172192517	172192571	8028	40,15	5,3	29,18	1,0	279	149	0.000573509076267	0.0298832226738	0.81,0.728	0.939,1.0	-0.201
6-8m_vs_8-10w	SE	Tmem38b	ENSMUSG00000028420.13	chr4	+	53827292	53827383	53826054	53826220	53840626	53840783	8059	2,0	28,11	7,10	8,2	239	149	6.1947015928e-07	0.000236844090898	0.043,0.0	0.353,0.757	-0.534
6-8m_vs_8-10w	SE	Stac2	ENSMUSG00000017400.10	chr11	-	98039618	98039756	98036622	98038070	98040097	98040149	8090	4,0	5,2	6,2	1,0	286	149	5.75190366954e-05	0.0068672170353 5	0.294,0.0	0.758,1.0	-0.732
6-8m_vs_8-10w	SE	Ednrb	ENSMUSG00000022122.15	chr14	-	103823054	103823259	103821637	103821787	103823391	103823504	8093	1,1	1,2	3,2	1,0	298	149	0.000379363774843	0.0238410125652	0.333,0.2	0.6,1.0	-0.534
6-8m_vs_8-10w	SE	Atp13a3	ENSMUSG00000022533.14	chr16	-	30332198	30332386	30328955	30329044	30336189	30336262	8121	267,255	1,1	339,288	11,8	298	149	0.000125139497015	0.0115820751762	0.993,0.992	0.939,0.947	0.049
6-8m_vs_8-10w	SE	Trip4	ENSMUSG00000032386.15	chr9	-	65874830	65875043	65873098	65873177	65879074	65879208	8325	31,14	0,2	14,3	8,9	298	149	1.40639201662e-06	0.000422127906596	1.0,0.778	0.467,0.143	0.584
6-8m_vs_8-10w	SE	Myef2	ENSMUSG00000027201.16	chr2	-	125098796	125098847	125098449	125098518	125100318	125100420	8371	3,2	16,16	19,13	6,13	199	149	1.18229902111e-05	0.0022075997303 5	0.123,0.086	0.703,0.428	-0.461

6-8m_vs_8-10w	SE	RP23-127B8.5	ENSMUSG00000014837.15	chr8	-	105282986	105283084	105280408	105282144	105283174	105283480	8466	2,8	4,1	3,1	0,0	246	149	0.000109083993457	0.0107135826724	0.232,0.829	1.0,1.0	-0.47
6-8m_vs_8-10w	SE	RP23-359G8.3	ENSMUSG00000116016.1	chr13	-	111453023	111453247	111448932	111448999	111453372	111453496	8599	3653,2831	192,110	2752,2688	412,161	298	149	1.58526093111e-05	0.00276696956867	0.905,0.928	0.77,0.893	0.085
6-8m_vs_8-10w	SE	Mkln1	ENSMUSG00000025609.15	chr6	+	31499886	31499941	31496668	31496771	31507624	31507847	8675	235,225	6,14	144,184	21,21	203	149	0.000130408900756	0.0120006081853	0.966,0.922	0.834,0.865	0.095
6-8m_vs_8-10w	SE	Rab11a	ENSMUSG00000004771.12	chr9	-	64728233	64728429	64726623	64726817	64737583	64737623	8683	646,396	33,19	342,317	44,34	298	149	3.41134546216e-05	0.00478422580187	0.907,0.912	0.795,0.823	0.101
6-8m_vs_8-10w	SE	Dusp12	ENSMUSG00000026659.13	chr1	-	170880129	170880226	170874187	170874570	170885066	170885199	8723	0,0	2,1	8,1	0,0	245	149	2.72590283679e-10	9.72723283404e-07	0.0,0.0	1.0,1.0	-1.0
6-8m_vs_8-10w	SE	Dusp12	ENSMUSG00000026659.13	chr1	-	170880572	170880691	170880129	170880226	170885066	170885199	8725	144,70	1,0	73,61	8,2	267	149	9.54626754949e-05	0.00967154348957	0.988,1.0	0.836,0.945	0.104
6-8m_vs_8-10w	SE	Dusp12	ENSMUSG00000026659.13	chr1	-	170880914	170881028	170880129	170880226	170885066	170885199	8726	30,8	1,0	22,12	8,2	262	149	6.90618582806e-05	0.00775521202986	0.945,1.0	0.61,0.773	0.281
6-8m_vs_8-10w	SE	Dusp12	ENSMUSG00000026659.13	chr1	-	170880914	170881041	170880129	170880226	170885066	170885396	8729	24,14	1,0	6,8	8,2	275	149	8.24299490443e-06	0.00166498128522	0.929,1.0	0.289,0.684	0.478
6-8m_vs_8-10w	SE	Ankrd31	ENSMUSG00000109561.1	chr13	+	96795738	96796299	96791700	96791744	96798727	96798891	8810	0,0	2,1	2,1	1,0	298	149	0.000430575476417	0.025513583027	0.0,0.0	0.5,1.0	-0.75
6-8m_vs_8-10w	SE	Nrg1	ENSMUSG00000062991.7	chr8	-	31838260	31838328	31837669	31837728	31849309	31849375	8951	0,1	22,13	4,5	11,1	216	149	4.58854472142e-05	0.00594216541424	0.0,0.05	0.201,0.775	-0.463
6-8m_vs_8-10w	SE	Pex2	ENSMUSG00000040374.13	chr3	-	5570486	5570629	5563619	5563729	5576037	5576128	9022	0,1	2,1	1,1	0,0	291	149	1.36647450877e-06	0.000422127906596	0.0,0.339	1.0,1.0	-0.831
6-8m_vs_8-10w	SE	Cep85	ENSMUSG00000037443.13	chr4	-	134164627	134164757	134156096	134156371	134172812	134172880	9090	1,0	1,2	2,1	0,0	278	149	1.66039196836e-06	0.000486192695501	0.349,0.0	1.0,1.0	-0.826
6-8m_vs_8-10w	SE	Hnrnpm	ENSMUSG00000059208.14	chr17	-	33652272	33652326	33648812	33648864	33658387	33658465	9166	2,7	3,2	7,2	0,0	202	149	4.12698744031e-05	0.00547695572864	0.33,0.721	1.0,1.0	-0.475
6-8m_vs_8-10w	SE	Yipf3	ENSMUSG00000071074.9	chr17	+	46251363	46251487	46251113	46251227	46251565	46252537	9259	29,20	5,3	37,32	1,0	272	149	0.000209134483893	0.0166032095462	0.761,0.785	0.953,1.0	-0.203
6-8m_vs_8-10w	SE	RP23-414K1.4	ENSMUSG00000033029.12	chr15	-	79136306	79136489	79135636	79135765	79140880	79140992	9534	5,5	0,2	0,0	3,2	298	149	7.25184294836e-08	4.31296644684e-05	1.0,0.556	0.0,0.0	0.778
6-8m_vs_8-10w	SE	Pik3cd	ENSMUSG00000039936.18	chr4	-	149697048	149697223	149674075	149674194	149701502	149701590	9631	7,4	10,20	0,1	13,11	298	149	0.000451300158578	0.0261690495914	0.259,0.091	0.0,0.043	0.154
6-8m_vs_8-10w	SE	Rtel1	ENSMUSG00000038685.18	chr2	+	181352040	181352180	181351575	181351953	181352752	181352848	9947	11,3	5,0	2,5	0,0	288	149	0.00100425807462	0.0436470183008	0.532,1.0	1.0,1.0	-0.234
6-8m_vs_8-10w	SE	Epb41l3	ENSMUSG00000024044.17	chr17	+	69283884	69284007	69281032	69281107	69284538	69284890	10205	6,4	0,0	5,2	1,3	271	149	0.000279556811436	0.0198195288214	1.0,1.0	0.733,0.268	0.5
6-8m_vs_8-10w	SE	Tesk2	ENSMUSG00000033985.17	chr4	+	116801159	116801244	116800532	116800615	116801448	116801532	10372	55,21	6,3	51,11	0,0	233	149	0.000110585963967	0.0108149423521	0.854,0.817	1.0,1.0	-0.165

6-8m_vs_8-10w	SE	Tmem114	ENSMUSG00000022715.3	chr16	-	8412100	8412238	8409274	8409761	8424062	8424143	10391	7,16	3,2	3,13	0,0	286	149	0.00021838677984	0.0169484375579	0.549,0.806	1.0,1.0	-0.323
6-8m_vs_8-10w	SE	Spsb3	ENSMUSG00000024160.16	chr17	+	24890567	24890755	24890282	24890460	24890832	24890935	10464	22,11	7,2	13,4	1,0	298	149	0.00104317091287	0.0447296088621	0.611,0.733	0.867,1.0	-0.262
6-8m_vs_8-10w	SE	RP23-294B15.2	ENSMUSG00000092152.1	chr14	-	42256546	42256730	42254658	42255402	42257383	42257515	10689	23,21	2,1	17,17	12,5	298	149	0.000428399791164	0.025478680913	0.852,0.913	0.415,0.63	0.36
6-8m_vs_8-10w	SE	Sycp2l	ENSMUSG00000038651.15	chr13	+	41157433	41157517	41155518	41155645	41163071	41163145	10796	9,3	8,10	1,0	4,13	232	149	0.000194376068681	0.0159716897997	0.419,0.162	0.138,0.0	0.221
6-8m_vs_8-10w	SE	Tex9	ENSMUSG00000090626.9	chr9	-	72482446	72482523	72480596	72480775	72484068	72484114	10868	8,6	0,0	5,1	4,2	225	149	4.63422766273e-07	0.00018839601967	1.0,1.0	0.453,0.249	0.649
6-8m_vs_8-10w	SE	Tex9	ENSMUSG00000090626.9	chr9	-	72482446	72482529	72479923	72480775	72484068	72484114	10869	28,14	0,0	19,5	4,2	231	149	4.85105101283e-05	0.00613371473732	1.0,1.0	0.754,0.617	0.315
6-8m_vs_8-10w	SE	Ubac2	ENSMUSG00000041765.6	chr14	+	121973608	121973732	121908210	121908320	121994224	121994308	10874	27,8	7,1	26,15	1,0	272	149	0.000230238178376	0.0174860313633	0.679,0.814	0.934,1.0	-0.221
6-8m_vs_8-10w	SE	Slc30a2	ENSMUSG00000028836.14	chr4	+	134347339	134347493	134345902	134346049	134348476	134348645	10966	7,4	0,0	11,13	3,3	298	149	0.000111161686162	0.0108183900387	1.0,1.0	0.647,0.684	0.335
6-8m_vs_8-10w	SE	Mnat1	ENSMUSG00000021103.12	chr12	+	73219020	73219142	73170593	73170667	73272413	73272851	11007	58,32	1,0	60,20	2,6	270	149	0.000705694000941	0.0342357530728	0.97,1.0	0.943,0.648	0.19
6-8m_vs_8-10w	SE	Ipp	ENSMUSG00000028696.12	chr4	+	116530412	116530535	116529670	116529808	116532557	116532778	11081	13,11	0,0	14,14	1,4	271	149	0.00060652713729	0.0308704049781	1.0,1.0	0.885,0.658	0.228
6-8m_vs_8-10w	SE	AC167036.4	ENSMUSG00000072109.11	chr1	+	85112191	85112311	85111808	85111959	85112450	85112585	11168	4,5	297,132	19,7	221,128	268	149	0.00058935252933	0.0304302987652	0.007,0.021	0.046,0.03	-0.024
6-8m_vs_8-10w	SE	AC167036.4	ENSMUSG00000072109.11	chr1	+	85112450	85112585	85111808	85111959	85113046	85113463	11170	638,298	17,6	388,281	1,0	283	149	5.50796177343e-05	0.0066501391096	0.952,0.963	0.995,1.0	-0.04
6-8m_vs_8-10w	SE	Hirip3	ENSMUSG00000042606.9	chr7	+	126862817	126862926	126862480	126862720	126863168	126863431	11199	6,8	4,5	6,3	1,0	257	149	0.000544034588571	0.0291912914895	0.465,0.481	0.777,1.0	-0.416
6-8m_vs_8-10w	SE	Adam5	ENSMUSG00000031554.18	chr8	-	24742097	24742151	24732984	24733157	24747481	24747568	11210	2,0	2,2	6,1	0,0	202	149	2.99969523765e-06	0.00074680784618	0.425,0.0	1.0,1.0	-0.788
6-8m_vs_8-10w	SE	Nudt22	ENSMUSG00000037349.9	chr19	-	6993661	6993759	6993432	6993526	6995241	6995735	11231	82,43	8,12	51,32	1,0	246	149	8.0429503132e-06	0.00164527001439	0.861,0.685	0.969,1.0	-0.211
6-8m_vs_8-10w	SE	Nudt22	ENSMUSG00000037349.9	chr19	-	6994670	6994769	6993432	6993526	6995241	6995735	11232	99,80	8,12	75,39	1,0	247	149	9.59012487134e-06	0.00188954877526	0.882,0.801	0.978,1.0	-0.147
6-8m_vs_8-10w	SE	Ube2d3	ENSMUSG00000078578.9	chr3	+	135458797	135458875	135439136	135439798	135465194	135465237	11310	38,19	4,3	11,21	0,0	226	149	0.000762163500019	0.0360495478153	0.862,0.807	1.0,1.0	-0.165
6-8m_vs_8-10w	SE	Ube2d3	ENSMUSG00000078578.9	chr3	+	135459974	135460080	135439136	135439798	135465194	135465237	11315	0,3	4,3	2,1	0,0	254	149	1.46478851271e-09	2.33283169685e-06	0.0,0.37	1.0,1.0	-0.815
6-8m_vs_8-10w	SE	Dnajc11	ENSMUSG00000039768.16	chr4	+	151969845	151969968	151968498	151968627	151970856	151970930	11347	11,7	0,0	12,5	4,4	271	149	8.59484323157e-07	0.00031014829806	1.0,1.0	0.623,0.407	0.485

6-8m_vs_8-10w	SE	Lsm6	ENSMUSG00000031683.16	chr8	-	78819986	78820085	78812975	78813080	78821047	78821140	11559	149,167	14,12	169,121	5,1	247	149	0.000324253051597	0.0214715690827	0.865,0.894	0.953,0.986	-0.09
6-8m_vs_8-10w	SE	Arhgef7	ENSMUSG00000031511.15	chr8	+	11791455	11791544	11785712	11785914	11800459	11800551	11744	23,13	6,0	17,7	0,0	237	149	0.000647386426996	0.0323066031958	0.707,1.0	1.0,1.0	-0.147
6-8m_vs_8-10w	SE	RP23-445H7.1	ENSMUSG00000030994.15	chr7	-	134375916	134376042	134357994	134358086	134385532	134385661	11796	19,12	23,21	7,5	43,20	274	149	0.000527907812276	0.028638998816	0.31,0.237	0.081,0.12	0.173
6-8m_vs_8-10w	SE	Gpatch2l	ENSMUSG00000021254.9	chr12	+	86281448	86281543	86268975	86269061	86288799	86291784	11806	1,2	0,1	0,0	1,1	243	149	0.00017513099626	0.0149191169122	1.0,0.551	0.0,0.0	0.776
6-8m_vs_8-10w	SE	Mtf2	ENSMUSG00000029267.17	chr5	+	108069161	108069363	108065715	108065965	108080824	108081023	11824	224,225	42,17	112,90	36,33	298	149	0.000142993613572	0.012899951948	0.727,0.869	0.609,0.577	0.205
6-8m_vs_8-10w	SE	Mtf2	ENSMUSG00000029267.17	chr5	+	108069161	108069523	108065715	108065965	108080824	108081023	11825	205,205	42,17	95,78	36,33	298	149	7.43514068887e-05	0.00823403373668	0.709,0.858	0.569,0.542	0.228
6-8m_vs_8-10w	SE	Gemin8	ENSMUSG00000040621.14	chrX	+	166176844	166177079	166170453	166170550	166178791	166178830	11920	2,3	0,0	0,0	1,1	298	149	5.17106923947e-09	5.18981436546e-06	1.0,1.0	0.0,0.0	1.0
6-8m_vs_8-10w	SE	Stk11	ENSMUSG00000003068.16	chr10	+	80126032	80126165	80125374	80125568	80126232	80126369	11994	57,37	0,0	39,20	4,2	281	149	0.000495553678409	0.0277268326407	1.0,1.0	0.838,0.841	0.161
6-8m_vs_8-10w	SE	Stk11	ENSMUSG00000003068.16	chr10	+	80128390	80129700	80127903	80128100	80129904	80130111	12000	5,2	121,65	12,11	83,73	298	149	0.000570939346029	0.0298761554985	0.02,0.015	0.067,0.07	-0.051
6-8m_vs_8-10w	SE	Epb41l4a	ENSMUSG00000024376.6	chr18	-	33810218	33810338	33802512	33802560	33824458	33824536	12101	134,56	8,9	57,45	0,0	268	149	1.08564271405e-05	0.00207538698836	0.903,0.776	1.0,1.0	-0.16
6-8m_vs_8-10w	SE	Zfp410	ENSMUSG00000042472.11	chr12	+	84332934	84333024	84327278	84327470	84342994	84343720	12122	1,2	0,0	0,1	4,1	238	149	5.46735545726e-10	1.46324656554e-06	1.0,1.0	0.0,0.385	0.808
6-8m_vs_8-10w	SE	Minos1	ENSMUSG00000050608.11	chr4	-	139105855	139105903	139101813	139104012	139130787	139130994	12183	19,19	0,0	26,20	2,8	196	149	4.52804859045e-05	0.00588756309842	1.0,1.0	0.908,0.655	0.218
6-8m_vs_8-10w	SE	Gm21411	ENSMUSG00000045699.3	chr4	-	146893748	146893875	146892405	146892658	146897711	146897815	12186	600,610	15,13	359,345	26,14	275	149	0.000823258752267	0.037771111554	0.956,0.962	0.882,0.93	0.053
6-8m_vs_8-10w	SE	G6pc3	ENSMUSG00000034793.15	chr11	+	102189870	102190143	102189619	102189652	102192034	102192141	12220	1,1	0,0	1,0	2,2	298	149	5.45200673496e-09	5.30595903939e-06	1.0,1.0	0.2,0.0	0.9
6-8m_vs_8-10w	SE	G6pc3	ENSMUSG00000034793.15	chr11	+	102190259	102190436	102189754	102190143	102192034	102192141	12222	0,0	2,1	2,1	1,0	298	149	0.000430575476417	0.025513583027	0.0,0.0	0.5,1.0	-0.75
6-8m_vs_8-10w	SE	Ecd	ENSMUSG00000021810.3	chr14	-	20324372	20324581	20319851	20320951	20327223	20327291	12401	292,273	1,0	303,196	9,6	298	149	0.00010904706689	0.0107135826724	0.993,1.0	0.944,0.942	0.053
6-8m_vs_8-10w	SE	RP24-149E13.1	ENSMUSG00000099922.1	chr1	+	59386785	59386867	59385858	59385997	59391555	59392299	12420	96,87	0,2	62,49	6,3	230	149	0.00086298497858	0.0389811892716	1.0,0.966	0.87,0.914	0.091
6-8m_vs_8-10w	SE	Bzw1	ENSMUSG00000051223.14	chr1	+	58404231	58404354	58403614	58403753	58405034	58405131	12510	3709,2336	63,43	3076,2650	107,72	271	149	0.000311492763287	0.0209286644053	0.97,0.968	0.94,0.953	0.023
6-8m_vs_8-10w	SE	Rbm6	ENSMUSG00000032582.14	chr9	-	107833433	107833507	107812096	107812367	107860348	107860459	12545	10,6	5,1	9,4	0,0	222	149	4.1154625419e-05	0.00547695572864	0.573,0.801	1.0,1.0	-0.313

6-8m_vs_8-10w	SE	Tktl1	ENSMUSG00000031397.11	chrX	+	74193518	74193710	74191221	74191319	74193793	74193921	12567	198,85	9,6	136,83	0,0	298	149	1.67500088351e-05	0.0028314319853 7	0.917,0.876	1.0,1.0	-0.103
6-8m_vs_8-10w	SE	Tktl1	ENSMUSG00000031397.11	chrX	+	74204873	74204957	74202530	74202661	74206344	74206464	12569	92,51	0,0	59,43	4,3	232	149	0.0010894413421 5	0.0458564851173	1.0,1.0	0.905,0.902	0.097
6-8m_vs_8-10w	SE	Nfyb	ENSMUSG00000020248.18	chr10	-	82754964	82755095	82749286	82752604	82756701	82756795	12669	916,682	4,12	756,654	15,30	279	149	4.7943970609e-05	0.0061101926987 2	0.992,0.968	0.964,0.921	0.037
6-8m_vs_8-10w	SE	H2-T22	ENSMUSG00000056116.18	chr17	-	36042341	36042460	36041867	36042128	36042569	36042690	12709	15,3	6,0	11,6	0,0	267	149	0.000357713835069	0.0229766750542	0.582,1.0	1.0,1.0	-0.209
6-8m_vs_8-10w	SE	RP23-360A2.8	ENSMUSG00000078870.9	chr2	-	177202826	177202881	177195701	177195828	177206339	177206421	12749	25,23	4,4	9,11	0,0	203	149	0.000241336735577	0.0180670643352	0.821,0.808	1.0,1.0	-0.186
6-8m_vs_8-10w	SE	Sorbs2	ENSMUSG00000031626.17	chr8	+	45785268	45785325	45775588	45775703	45789975	45790025	12838	6,1	14,10	0,0	8,5	205	149	0.000478362494361	0.0270953965942	0.238,0.068	0.0,0.0	0.153
6-8m_vs_8-10w	SE	Gnl3	ENSMUSG00000042354.7	chr14	-	31017807	31017866	31017056	31017167	31018928	31018989	12872	17,24	7,0	25,8	0,0	207	149	0.000670746721965	0.0330133035861	0.636,1.0	1.0,1.0	-0.182
6-8m_vs_8-10w	SE	Cyba	ENSMUSG00000006519.11	chr8	-	122428399	122428694	122427654	122427724	122432809	122432905	12972	2,2	3,1	1,3	0,0	298	149	1.64101183712e-05	0.0028093181352 8	0.25,0.5	1.0,1.0	-0.625
6-8m_vs_8-10w	SE	Thrap3	ENSMUSG00000043962.16	chr4	-	126173136	126173329	126171693	126171881	126173443	126173527	12974	6,6	26,24	1,0	15,23	298	149	0.000521785549329	0.0285966974441	0.103,0.111	0.032,0.0	0.091
6-8m_vs_8-10w	SE	Bola2	ENSMUSG00000047721.8	chr7	+	126696220	126696280	126695965	126696099	126696554	126696693	13019	142,163	51,50	212,113	32,21	208	149	0.000681357959529	0.0333066852789	0.666,0.7	0.826,0.794	-0.127
6-8m_vs_8-10w	SE	Nckap5	ENSMUSG00000049690.15	chr1	-	125940162	125940237	125914959	125915021	125965147	125965200	13030	8,5	3,1	1,1	6,5	223	149	3.72438830134e-05	0.0050469390162 8	0.641,0.77	0.1,0.118	0.597
6-8m_vs_8-10w	SE	Ggps1	ENSMUSG00000021302.10	chr13	-	14058923	14059211	14057795	14057888	14059318	14059462	13051	144,105	29,8	64,53	43,25	298	149	2.50700753768e-06	0.000673976755968	0.713,0.868	0.427,0.515	0.32
6-8m_vs_8-10w	SE	Lrrc51	ENSMUSG00000064307.13	chr7	-	101921503	101921566	101920636	101920774	101933749	101933830	13140	8,3	3,1	5,3	0,0	211	149	0.000988036623424	0.0431138372254	0.653,0.679	1.0,1.0	-0.334
6-8m_vs_8-10w	SE	Chuk	ENSMUSG00000025199.16	chr19	-	44086907	44087059	44084416	44084478	44087915	44088039	13189	98,50	4,2	67,34	15,11	298	149	5.73333244813e-06	0.001252596632	0.925,0.926	0.691,0.607	0.276
6-8m_vs_8-10w	SE	Fam169b	ENSMUSG00000074071.11	chr7	+	68321819	68321905	68304551	68304651	68350233	68350416	13207	10,5	0,0	6,8	6,3	234	149	5.23398312913e-07	0.000207524200216	1.0,1.0	0.389,0.629	0.491
6-8m_vs_8-10w	SE	Fam169b	ENSMUSG00000074071.11	chr7	+	68329221	68329399	68304551	68304651	68350233	68350416	13210	1,1	0,0	4,1	6,3	298	149	1.57394327571e-08	1.12330582762e-05	1.0,1.0	0.25,0.143	0.804
6-8m_vs_8-10w	SE	Nlrp2	ENSMUSG00000035177.8	chr7	-	5327407	5329010	5324893	5325064	5332959	5333019	13293	33,25	0,0	26,21	6,0	298	149	0.0006873026764 8	0.0335462199967	1.0,1.0	0.684,1.0	0.158
6-8m_vs_8-10w	SE	Wbp2	ENSMUSG00000034341.17	chr11	-	116080545	116080680	116080221	116080344	116082275	116082411	13324	13,2	6,0	2,1	0,0	283	149	0.000417543854571	0.0250651185671	0.533,1.0	1.0,1.0	-0.234
6-8m_vs_8-10w	SE	Wbp2	ENSMUSG00000034341.17	chr11	-	116081231	116081324	116080221	116080344	116082275	116082411	13325	38,7	6,0	7,5	0,0	241	149	0.000660845788394	0.0326617463853	0.797,1.0	1.0,1.0	-0.101

6-8m_vs_8-10w	SE	RP23-314E23.2	ENSMUSG00000111852.1	chr9	-	59510875	59511143	59499360	59500479	59512772	59512865	13363	1,1	3,2	3,3	0,0	298	149	1.20860175867e-07	6.16118318753e-05	0.143,0.2	1.0,1.0	-0.829
6-8m_vs_8-10w	SE	Arhgap10	ENSMUSG00000037148.8	chr8	-	77365085	77365160	77358546	77358634	77382724	77382790	13431	37,20	0,0	15,5	3,4	223	149	5.64536309977e-06	0.00125038952629	1.0,1.0	0.77,0.455	0.387
6-8m_vs_8-10w	SE	Unkl	ENSMUSG00000015127.14	chr17	+	25218577	25218755	25213046	25213106	25228582	25228666	13467	9,4	10,1	1,0	3,1	298	149	0.00110467294591	0.04643674912417	0.31,0.667	0.143,0.0	0.417
6-8m_vs_8-10w	SE	Eif3i	ENSMUSG00000028798.16	chr4	-	129596873	129596939	129593525	129593636	129600408	129600501	13487	3,3	3,9	0,0	1,3	214	149	0.000192432142316	0.0159282234088	0.41,0.188	0.0,0.0	0.299
6-8m_vs_8-10w	SE	Psat1	ENSMUSG00000024640.9	chr19	-	15919454	15919524	15918205	15918411	15920935	15920996	13500	238,94	0,0	193,145	5,7	218	149	0.000741697360567	0.0352894110103	1.0,1.0	0.963,0.934	0.051
6-8m_vs_8-10w	SE	Zfp664	ENSMUSG00000079215.8	chr5	+	124863353	124863508	124862690	124862882	124884707	124884772	13579	358,217	29,22	243,199	10,2	298	149	6.25837450402e-05	0.00720408442907	0.861,0.831	0.924,0.98	-0.106
6-8m_vs_8-10w	SE	Pdha1	ENSMUSG00000031299.10	chrX	-	160132068	160132242	160130108	160130235	160133172	160133232	13661	75,48	8,3	75,69	2,0	298	149	0.000718744034412	0.034504011075	0.824,0.889	0.949,1.0	-0.118
6-8m_vs_8-10w	SE	Rph3al	ENSMUSG00000020847.15	chr11	-	75911606	75911731	75910949	75911059	75937662	75937697	13927	11,1	1,0	8,1	4,1	273	149	0.000769762816769	0.0361672891129	0.857,1.0	0.522,0.353	0.491
6-8m_vs_8-10w	SE	Tex2	ENSMUSG00000040548.16	chr11	-	106519881	106520014	106513586	106513712	106529261	106529361	13970	9,18	0,0	5,10	7,1	281	149	2.06664366115e-05	0.00331861639107	1.0,1.0	0.275,0.841	0.442
6-8m_vs_8-10w	SE	Plekhb1	ENSMUSG00000030701.17	chr7	-	100649927	100650032	100645569	100645669	100653653	100653693	14078	15,8	19,12	4,1	16,15	253	149	0.00106584256655	0.0451591027273	0.317,0.282	0.128,0.038	0.216
6-8m_vs_8-10w	SE	Prdx5	ENSMUSG00000024953.16	chr19	-	6907541	6907673	6907241	6907280	6907981	6908116	14137	594,407	16,30	444,325	7,5	280	149	0.000297477936287	0.0204141055594	0.952,0.878	0.971,0.972	-0.056
6-8m_vs_8-10w	SE	Atpaf2	ENSMUSG00000042709.14	chr11	-	60411666	60411729	60409585	60409630	60416985	60417042	14162	11,9	0,1	7,3	4,4	211	149	3.03534068538e-05	0.0043325778423	1.0,0.864	0.553,0.346	0.482
6-8m_vs_8-10w	SE	Nhsl1	ENSMUSG00000039835.16	chr10	+	18516028	18516160	18511549	18511742	18523790	18525959	14166	7,4	1,0	0,3	1,3	280	149	8.61065927404e-05	0.00908435046067	0.788,1.0	0.0,0.347	0.721
6-8m_vs_8-10w	SE	Ptges2	ENSMUSG00000026820.5	chr2	+	32401486	32401604	32400049	32400108	32402175	32405772	14189	14,5	3,2	6,8	0,0	266	149	0.000191310750647	0.0158896688863	0.723,0.583	1.0,1.0	-0.347
6-8m_vs_8-10w	SE	Lat2	ENSMUSG00000040751.12	chr5	-	134606045	134606081	134605931	134605962	134606559	134606620	14250	1,0	6,4	2,4	3,3	184	149	0.000460591094983	0.0264070591364	0.119,0.0	0.351,0.519	-0.376
6-8m_vs_8-10w	SE	Lat2	ENSMUSG00000040751.12	chr5	-	134606045	134606093	134605937	134605962	134606360	134606392	14251	0,0	13,3	2,3	3,9	196	149	0.000971504058005	0.0425659267761	0.0,0.0	0.336,0.202	-0.269
6-8m_vs_8-10w	SE	Trnt1	ENSMUSG00000013736.16	chr6	+	106770801	106770915	106769829	106770003	106773284	106773478	14309	11,4	182,144	25,14	137,126	262	149	0.000122519717224	0.0114053427199	0.033,0.016	0.094,0.059	-0.052
6-8m_vs_8-10w	SE	Eif5	ENSMUSG00000021282.17	chr12	+	111542679	111542841	111542151	111542310	111543099	111543264	14327	1779,1634	37,46	1560,1700	98,113	298	149	1.14160159104e-09	2.27260550428e-06	0.96,0.947	0.888,0.883	0.068
6-8m_vs_8-10w	SE	Eif5	ENSMUSG00000021282.17	chr12	+	111542779	111542841	111542224	111542310	111543099	111543264	14329	408,358	37,46	384,393	98,113	210	149	3.79590159305e-08	2.34440722235e-05	0.887,0.847	0.735,0.712	0.143

6-8m_vs_8-10w	SE	Rpl13	ENSMUSG00000000740.12	chr8	+	123102844	123102986	123102650	123102762	123103182	123103356	14357	1179,643	48,38	1008,625	21,16	290	149	0.000487256163816	0.0274058125344	0.927,0.897	0.961,0.953	-0.045
6-8m_vs_8-10w	SE	Fam134c	ENSMUSG00000017802.14	chr11	-	101117185	101117300	101106306	101106413	101119540	101119765	14378	5,1	2,2	0,0	3,3	263	149	4.07870784178e-05	0.00545799087694	0.586,0.221	0.0,0.0	0.403
6-8m_vs_8-10w	SE	Brca2	ENSMUSG00000041147.10	chr5	+	150560427	150560591	150560003	150560196	150560684	150560823	14498	227,138	2,0	165,179	8,5	298	149	0.000597570069013	0.0306085491809	0.983,1.0	0.912,0.947	0.062
6-8m_vs_8-10w	SE	RP24-91A16.3	ENSMUSG00000099564.2	chr9	-	96519400	96519553	96517642	96517791	96521185	96521395	14641	9,3	6,0	2,11	0,0	298	149	0.000697618967639	0.0338952053929	0.429,1.0	1.0,1.0	-0.286
6-8m_vs_8-10w	SE	Fam63a	ENSMUSG00000038712.16	chr3	+	95291479	95291544	95291021	95291079	95292124	95292283	14659	213,81	1,1	66,92	4,8	213	149	5.45217527458e-05	0.00663654734167	0.993,0.983	0.92,0.889	0.083
6-8m_vs_8-10w	SE	Dnajb13	ENSMUSG00000030708.14	chr7	-	100507375	100507533	100506081	100506195	100510694	100510856	14710	20,5	0,0	4,0	4,2	298	149	9.50127843069e-09	8.03008047579e-06	1.0,1.0	0.333,0.0	0.834
6-8m_vs_8-10w	SE	Huwe1	ENSMUSG00000025261.17	chrX	+	151804445	151804540	151803260	151803569	151806275	151806407	14815	0,2	5,14	4,1	0,0	243	149	1.52539125775e-09	2.33283169685e-06	0.0,0.081	1.0,1.0	-0.96
6-8m_vs_8-10w	SE	Tnfrsf12a	ENSMUSG00000023905.14	chr17	-	23676518	23676623	23675446	23676046	23677326	23677449	14893	21,16	5,6	45,18	2,1	253	149	0.00103811345909	0.0446917585149	0.712,0.611	0.93,0.914	-0.261
6-8m_vs_8-10w	SE	Tnfrsf12a	ENSMUSG00000023905.14	chr17	-	23676518	23676623	23676138	23676273	23677326	23677449	14894	98,68	9,7	152,64	2,1	253	149	8.31560979112e-05	0.00892879742877	0.865,0.851	0.978,0.974	-0.118
6-8m_vs_8-10w	SE	RP24-510G5.3	ENSMUSG00000092570.7	chr5	-	147239188	147239413	147231201	147231289	147271219	147271521	15010	2,1	0,0	1,0	1,1	298	149	0.000271237360505	0.0194260017558	1.0,1.0	0.333,0.0	0.834
6-8m_vs_8-10w	SE	Dmgdh	ENSMUSG00000042102.7	chr13	+	93711292	93711458	93709181	93709335	93712045	93712176	15013	8,6	0,0	1,2	2,1	298	149	0.000676992722137	0.0331437473539	1.0,1.0	0.2,0.5	0.65
6-8m_vs_8-10w	SE	Ncapg	ENSMUSG00000015880.13	chr5	+	45691601	45691826	45688560	45688680	45695653	45695815	15116	95,81	1,2	96,132	7,13	298	149	0.000348425816394	0.0225151781073	0.979,0.953	0.873,0.835	0.112
6-8m_vs_8-10w	SE	Ncapg	ENSMUSG00000015880.13	chr5	+	45691601	45693338	45688560	45688680	45695653	45695815	15118	93,83	1,2	90,121	7,13	298	149	0.000198310059918	0.0160023263425	0.979,0.954	0.865,0.823	0.122
6-8m_vs_8-10w	SE	Ncapg	ENSMUSG00000015880.13	chr5	+	45693156	45693338	45691601	45691826	45693751	45693926	15121	197,171	15,5	202,191	3,2	298	149	0.000653209998711	0.03242425397	0.868,0.945	0.971,0.979	-0.069
6-8m_vs_8-10w	SE	Ncapg	ENSMUSG00000015880.13	chr5	+	45693156	45693338	45691601	45691826	45695653	45695815	15122	100,74	3,0	93,89	9,14	298	149	1.3248924899e-05	0.00240396876868	0.943,1.0	0.838,0.761	0.172
6-8m_vs_8-10w	SE	Ncapg	ENSMUSG00000015880.13	chr5	+	45693751	45693926	45688560	45688680	45695653	45695815	15123	151,138	1,2	132,128	7,13	298	149	1.75947170503e-05	0.00294308298327	0.987,0.972	0.904,0.831	0.112
6-8m_vs_8-10w	SE	Ncapg	ENSMUSG00000015880.13	chr5	+	45693751	45693926	45691601	45691826	45695653	45695815	15124	166,142	3,0	134,130	9,14	298	149	1.39973503122e-06	0.00042212790659 6	0.965,1.0	0.882,0.823	0.13
6-8m_vs_8-10w	SE	Flad1	ENSMUSG00000042642.13	chr3	-	89407408	89407556	89405580	89405770	89408421	89409166	15145	13,4	3,3	13,8	1,0	296	149	0.000612246947041	0.0310630694331	0.686,0.402	0.867,1.0	-0.389
6-8m_vs_8-10w	SE	Uchl5	ENSMUSG00000018189.12	chr1	+	143794482	143794544	143786129	143786235	143796761	143796825	15368	2,4	0,0	1,2	4,2	210	149	3.44753208203e-08	2.19704427932e-05	1.0,1.0	0.151,0.415	0.717

6-8m_vs_8-10w	SE	Rmnd1	ENSMUSG00000019763.11	chr10	-	4428408	4428465	4427444	4427691	4429076	4429171	15403	1,0	4,2	2,7	3,1	205	149	0.000270424424605	0.0194260017558	0.154,0.0	0.326,0.836	-0.504
6-8m_vs_8-10w	SE	Ampd3	ENSMUSG00000005686.17	chr7	+	110788609	110788814	110777689	110778024	110791187	110791314	15451	6,3	0,0	1,0	0,2	298	149	0.0011956388548 8	0.048483765083	1.0,1.0	1.0,0.0	0.5
6-8m_vs_8-10w	SE	Usp26	ENSMUSG00000055780.10	chrX	-	51801001	51801144	51800812	51800928	51801220	51801233	15527	25,11	4,2	11,8	0,0	291	149	0.000392212940562	0.0242537005291	0.762,0.738	1.0,1.0	-0.25
6-8m_vs_8-10w	SE	Fastkd2	ENSMUSG00000025962.15	chr1	+	63737778	63737918	63735462	63735571	63745841	63746005	15563	3,5	0,1	0,1	1,1	288	149	0.000301751298761	0.0205754664777	1.0,0.721	0.0,0.341	0.69
6-8m_vs_8-10w	SE	RP23-49C8.7	ENSMUSG00000046774.17	chrX	+	101798101	101798156	101797715	101797761	101798341	101798383	15568	34,21	37,18	54,33	19,13	203	149	0.000591674349336	0.0305011451096	0.403,0.461	0.676,0.651	-0.231
6-8m_vs_8-10w	SE	Skiv2l	ENSMUSG00000040356.16	chr17	-	34839690	34839831	34839243	34839605	34839996	34840215	15613	18,4	12,1	23,17	1,2	289	149	0.0011781921311 3	0.048483765083	0.436,0.673	0.922,0.814	-0.314
6-8m_vs_8-10w	SE	Dnajc25	ENSMUSG00000070972.13	chr4	+	59013631	59013855	59003192	59003557	59017669	59017822	15626	5,6	3,1	1,2	10,4	298	149	0.000512191321277	0.0281188657677	0.455,0.75	0.048,0.2	0.479
6-8m_vs_8-10w	SE	Oosp3	ENSMUSG00000055933.13	chr19	+	11699351	11699530	11697054	11697171	11700913	11701011	15684	2174,1977	2349,2250	1779,1640	925,1068	298	149	1.26174881654e-09	2.27260550428e-06	0.316,0.305	0.49,0.434	-0.151
6-8m_vs_8-10w	SE	Scp2	ENSMUSG00000028603.15	chr4	-	108087011	108087162	108085154	108085302	108091236	108091323	15731	9,9	4,1	11,16	0,0	298	149	0.000111896351406	0.0108370971265	0.529,0.818	1.0,1.0	-0.327
6-8m_vs_8-10w	SE	Ltbp3	ENSMUSG00000024940.10	chr19	+	5757310	5757472	5756793	5756931	5757546	5757627	15744	1,0	6,2	14,1	7,2	298	149	0.000153680633953	0.0135300037886	0.077,0.0	0.5,0.2	-0.312
6-8m_vs_8-10w	SE	Dpm1	ENSMUSG00000078919.10	chr2	-	168217680	168217776	168212959	168213028	168220036	168220113	15759	400,256	53,32	380,273	27,13	244	149	0.0004612766277 1	0.0264070591364	0.822,0.83	0.896,0.928	-0.086
6-8m_vs_8-10w	SE	Sppl2a	ENSMUSG00000027366.12	chr2	-	126913299	126913353	126912642	126912745	126913531	126913588	15766	0,1	1,2	3,2	0,0	202	149	5.70420574819e-06	0.001252596632	0.0,0.269	1.0,1.0	-0.865
6-8m_vs_8-10w	SE	Ankrd26	ENSMUSG00000007827.10	chr6	-	118540450	118540549	118539846	118539875	118542889	118542983	15824	18,22	2,4	8,18	0,0	247	149	0.000596663466679	0.0306085491809	0.844,0.768	1.0,1.0	-0.194
6-8m_vs_8-10w	SE	AC123686.3	ENSMUSG00000089889.2	chr5	+	143330703	143330885	143329326	143329353	143332310	143332701	15929	13,1	0,0	1,1	1,1	298	149	0.000578811000203	0.0299824098105	1.0,1.0	0.333,0.333	0.667
6-8m_vs_8-10w	SE	Srsf3	ENSMUSG00000071172.12	chr17	+	29039453	29039909	29038489	29038624	29040774	29040813	15951	264,119	262,118	153,120	100,59	298	149	0.0009204728378 8	0.0408878363228	0.335,0.335	0.433,0.504	-0.134
6-8m_vs_8-10w	SE	Atp9a	ENSMUSG00000027546.15	chr2	-	168687340	168687435	168683712	168683793	168689298	168689350	16015	25,22	5,3	28,10	0,1	243	149	0.0010353563545 3	0.0446469990778	0.754,0.818	1.0,0.86	-0.144
6-8m_vs_8-10w	SE	Clcn3	ENSMUSG00000004319.15	chr8	-	60914577	60914828	60910860	60913175	60919339	60919556	16041	13,12	15,8	6,4	23,20	298	149	0.000472154207147	0.0267909973794	0.302,0.429	0.115,0.091	0.262
6-8m_vs_8-10w	SE	March8	ENSMUSG00000025702.15	chr6	+	116338385	116338490	116338023	116338215	116376692	116376863	16087	0,0	1,1	1,6	0,0	253	149	1.92574933777e-09	2.68901590138e-06	0.0,0.0	1.0,1.0	-1.0
6-8m_vs_8-10w	SE	Nrg4	ENSMUSG00000032311.17	chr9	-	55280889	55280983	55240699	55242207	55282540	55282616	16150	1,1	5,1	1,4	1,0	242	149	0.000190400081744	0.0158843568606	0.11,0.381	0.381,1.0	-0.445

6-8m_vs_8-10w	SE	Tmem234	ENSMUSG00000028797.20	chr4	+	129601406	129601625	129600982	129601134	129601869	129601930	16370	1,2	0,0	0,0	1,1	298	149	1.17288146972e-08	9.22959793464e-06	1.0,1.0	0.0,0.0	1.0
6-8m_vs_8-10w	SE	Rsrc2	ENSMUSG00000029422.15	chr5	-	123733555	123733630	123729083	123729356	123735821	123735896	16405	1,0	0,3	1,4	0,0	223	149	0.000575965712887	0.0298832226738	1.0,0.0	1.0,1.0	-0.5
6-8m_vs_8-10w	SE	Zbed5	ENSMUSG00000034173.13	chr5	+	129899182	129899432	129895722	129895852	129900344	129900495	16570	14,3	3,5	9,6	1,0	298	149	0.000470087668578	0.0267868759236	0.7,0.231	0.818,1.0	-0.444
6-8m_vs_8-10w	SE	Anapc15	ENSMUSG00000030649.18	chr7	+	101872596	101872736	101871616	101871671	101897732	101897817	16701	7,3	0,0	2,2	4,2	288	149	2.67457073155e-08	1.75299007376e-05	1.0,1.0	0.206,0.341	0.726
6-8m_vs_8-10w	SE	Anapc15	ENSMUSG00000030649.18	chr7	+	101879216	101879301	101871641	101871671	101897732	101897817	16702	1,4	0,0	0,1	4,2	233	149	2.12960760138e-11	2.75983081166e-07	1.0,1.0	0.0,0.242	0.879
6-8m_vs_8-10w	SE	Xlr4c	ENSMUSG00000031362.17	chrX	-	73242067	73242182	73241315	73241384	73242613	73242660	16731	61,44	0,0	37,32	4,2	263	149	0.000941749957528	0.0416601124462	1.0,1.0	0.84,0.901	0.129
6-8m_vs_8-10w	SE	Ylpm1	ENSMUSG00000021244.15	chr12	+	85041822	85041915	85040572	85040993	85042121	85042311	16813	45,34	0,0	28,38	5,2	241	149	0.000217400705906	0.0169484375579	1.0,1.0	0.776,0.922	0.151
6-8m_vs_8-10w	SE	Dhdh	ENSMUSG00000011382.8	chr7	-	45481788	45482041	45479008	45479133	45486740	45486904	17046	10,5	0,1	4,1	4,3	298	149	1.39737476631e-05	0.0024932271108 2	1.0,0.714	0.333,0.143	0.619
6-8m_vs_8-10w	SE	RP23-290N23.1	ENSMUSG00000096276.9	chr12	+	87955402	87955585	87954474	87954582	87956562	87956613	17087	98,2	5,7	61,111	0,2	298	149	0.000186773460855	0.0157066732194	0.907,0.125	1.0,0.965	-0.466
6-8m_vs_8-10w	SE	RP23-204P24.2	ENSMUSG00000051297.8	chr16	+	92492013	92492175	92478741	92478968	92497049	92497366	17105	1,0	4,2	2,5	0,0	298	149	3.31821237154e-11	2.75983081166e-07	0.111,0.0	1.0,1.0	-0.945
6-8m_vs_8-10w	SE	Slc39a9	ENSMUSG00000048833.9	chr12	+	80677131	80677266	80675848	80675934	80679474	80679875	17123	43,16	0,0	28,23	4,2	283	149	0.000557837145471	0.0296123929983	1.0,1.0	0.787,0.858	0.178
6-8m_vs_8-10w	SE	Zfp414	ENSMUSG00000073423.9	chr17	+	33629621	33629698	33629089	33629216	33629796	33630079	17163	13,8	4,1	2,0	6,1	225	149	8.72810015113e-05	0.0091306731092 4	0.683,0.841	0.181,0.0	0.672
6-8m_vs_8-10w	SE	Zfp414	ENSMUSG00000073423.9	chr17	+	33631128	33631463	33630575	33630681	33631541	33631716	17165	51,12	4,3	38,8	0,0	298	149	0.000210107369483	0.0166202174343	0.864,0.667	1.0,1.0	-0.234
6-8m_vs_8-10w	SE	Dscc1	ENSMUSG00000022422.13	chr15	-	55086752	55086843	55084048	55084187	55087601	55087736	17253	79,34	1,0	49,24	3,5	239	149	0.000310968162791	0.0209286644053	0.98,1.0	0.911,0.75	0.159
6-8m_vs_8-10w	SE	Tollip	ENSMUSG00000025139.14	chr7	-	141898694	141898783	141892739	141892889	141902275	141902308	17331	2,3	7,3	0,0	6,4	237	149	0.0010620095394 8	0.0451591027273	0.152,0.386	0.0,0.0	0.269
6-8m_vs_8-10w	SE	Gal	ENSMUSG00000024907.6	chr19	-	3413294	3413349	3411557	3411647	3414031	3414112	17457	25,26	4,4	21,19	0,0	203	149	0.000173861062628	0.014889925033	0.821,0.827	1.0,1.0	-0.176
6-8m_vs_8-10w	SE	H2-M10.4	ENSMUSG00000048231.14	chr17	-	36461235	36461511	36460389	36460665	36461745	36462015	17492	2,5	0,0	5,0	2,2	298	149	1.94152165568e-05	0.0031491873481 7	1.0,1.0	0.556,0.0	0.722
6-8m_vs_8-10w	SE	Plekha1	ENSMUSG00000040268.17	chr7	+	130900516	130900660	130896990	130897450	130902176	130902245	17566	173,97	0,0	138,127	6,5	292	149	0.0002195776835 5	0.0169518194348	1.0,1.0	0.921,0.928	0.075
6-8m_vs_8-10w	SE	Pate2	ENSMUSG00000074452.13	chr9	+	35611771	35611871	35607594	35607719	35646146	35646457	17629	70,86	2,0	31,46	2,6	248	149	0.0010466274576 4	0.044798690974	0.955,1.0	0.903,0.822	0.115

6-8m_vs_8-10w	SE	Pkig	ENSMUSG00000035268.14	chr2	+	163703485	163703555	163658476	163658659	163721127	163721198	17739	98,50	10,0	63,38	0,0	218	149	0.000602322285844	0.0307392915696	0.87,1.0	1.0,1.0	-0.065
6-8m_vs_8-10w	SE	Mrpl48	ENSMUSG00000030706.17	chr7	-	100565193	100565427	100552578	100552681	100571251	100571289	17760	15,5	2,2	3,2	10,3	298	149	0.000140182659398	0.0127493210983	0.789,0.556	0.13,0.25	0.483
6-8m_vs_8-10w	SE	Psma3	ENSMUSG00000060073.9	chr12	+	70988423	70988489	70986862	70986935	70990484	70990531	17883	2037,1762	10,23	2321,1541	51,30	214	149	6.74888888768e-05	0.00763194772946	0.993,0.982	0.969,0.973	0.017
6-8m_vs_8-10w	SE	AC131791.1	ENSMUSG00000040139.14	chr7	-	137387539	137387701	137376954	137377117	137410639	137410732	17998	294,149	1,3	227,136	17,2	298	149	0.000159670837531	0.0139347516797	0.993,0.961	0.87,0.971	0.056
6-8m_vs_8-10w	SE	Pou2f1	ENSMUSG00000026565.18	chr1	-	165945901	165946002	165931661	165931727	166002561	166002609	18025	3,0	2,1	4,1	0,0	249	149	0.00112933857138	0.0472263509876	0.473,0.0	1.0,1.0	-0.764
6-8m_vs_8-10w	SE	Zc3h11a	ENSMUSG00000102976.6	chr1	-	133655344	133655398	133653972	133654032	133661236	133661318	18098	209,203	26,38	131,119	45,40	202	149	0.000153115475048	0.0135300037886	0.856,0.798	0.682,0.687	0.142
6-8m_vs_8-10w	SE	Ppp4c	ENSMUSG00000030697.7	chr7	-	126787436	126787610	126787219	126787346	126788383	126788434	18145	348,117	57,17	184,109	10,2	298	149	2.51828405518e-06	0.000673976755968	0.753,0.775	0.902,0.965	-0.169
6-8m_vs_8-10w	SE	Ppp4c	ENSMUSG00000030697.7	chr7	-	126788116	126788218	126787219	126787346	126788383	126788434	18146	210,39	57,17	98,52	10,2	250	149	2.41230891364e-05	0.00365677963838	0.687,0.578	0.854,0.939	-0.264
6-8m_vs_8-10w	SE	Smim20	ENSMUSG00000061461.11	chr5	+	53268022	53268331	53267137	53267358	53277142	53277199	18228	4,4	10,9	1,0	13,16	298	149	0.000446107240112	0.0260705962205	0.167,0.182	0.037,0.0	0.156
6-8m_vs_8-10w	SE	Aamdc	ENSMUSG00000035642.16	chr7	-	97569686	97569774	97565150	97565300	97579365	97579442	18253	27,22	2,3	20,12	8,15	236	149	0.000297435273886	0.0204141055594	0.895,0.822	0.612,0.336	0.385
6-8m_vs_8-10w	SE	Aamdc	ENSMUSG00000035642.16	chr7	-	97575587	97575691	97550541	97550741	97579255	97579474	18255	28,25	0,6	48,12	0,0	252	149	0.00118710026368	0.048483765083	1.0,0.711	1.0,1.0	-0.145
6-8m_vs_8-10w	SE	Aamdc	ENSMUSG00000035642.16	chr7	-	97575587	97575691	97565150	97565300	97579365	97579398	18260	145,163	2,3	191,117	8,15	252	149	0.000255359945127	0.0186840536116	0.977,0.97	0.934,0.822	0.096
6-8m_vs_8-10w	SE	Eif3h	ENSMUSG00000022312.11	chr15	-	51796387	51796537	51789992	51790113	51797612	51797712	18460	1298,748	18,6	1165,821	27,27	298	149	0.000717933183506	0.034504011075	0.973,0.984	0.956,0.938	0.031
6-8m_vs_8-10w	SE	Echdc2	ENSMUSG00000028601.18	chr4	+	108172738	108172972	108172088	108172175	108173794	108173851	18590	7,4	0,1	8,5	4,2	298	149	0.00118676388201	0.048483765083	1.0,0.667	0.5,0.556	0.306
6-8m_vs_8-10w	SE	Tet1	ENSMUSG00000047146.17	chr10	-	62878151	62880133	62864807	62864861	62898943	62899118	18626	10,18	4,1	4,13	0,0	298	149	0.000500796089889	0.0278272816688	0.556,0.9	1.0,1.0	-0.272
6-8m_vs_8-10w	SE	Armc6	ENSMUSG00000002343.11	chr8	-	70222105	70222243	70220171	70220877	70222517	70222649	18703	0,0	1,3	1,2	0,0	286	149	1.41759115557e-09	2.33283169685e-06	0.0,0.0	1.0,1.0	-1.0
6-8m_vs_8-10w	SE	Ubp1	ENSMUSG00000009741.15	chr9	+	113970141	113970260	113969224	113969416	113972750	113972893	18910	58,24	5,3	51,69	1,0	267	149	0.000374957549712	0.0237721098649	0.866,0.817	0.966,1.0	-0.142
6-8m_vs_8-10w	SE	RP24-309H3.4	ENSMUSG00000097145.5	chr5	+	52192917	52192978	52190671	52190937	52196892	52197023	18955	14,4	9,0	7,6	0,0	209	149	0.000248092267653	0.0184438223795	0.526,1.0	1.0,1.0	-0.237
6-8m_vs_8-10w	SE	RP24-309H3.4	ENSMUSG00000097145.5	chr5	+	52194800	52194890	52190678	52190937	52196892	52197023	18957	13,15	9,0	17,11	0,0	238	149	0.000531766325492	0.0287996750582	0.475,1.0	1.0,1.0	-0.262

6-8m_vs_8-10w	SE	RP24-370N20.1	ENSMUSG00000109619.1	chr7	-	133321701	133321774	133319984	133320183	133333070	133333201	19181	23,15	0,10	7,4	0,0	221	149	0.000697211935804	0.0338952053929	1.0,0.503	1.0,1.0	-0.248
6-8m_vs_8-10w	SE	Zfp846	ENSMUSG00000058192.16	chr9	+	20589456	20589558	20588508	20588635	20590812	20590884	19216	4,0	3,3	4,2	1,0	250	149	0.000255396049803	0.0186840536116	0.443,0.0	0.704,1.0	-0.631
6-8m_vs_8-10w	SE	Rpl36	ENSMUSG00000057863.5	chr17	+	56613585	56613680	56613394	56613448	56614123	56614243	19354	23,13	6,3	16,5	0,0	243	149	2.68162992809e-05	0.0039146921259 3	0.702,0.727	1.0,1.0	-0.286
6-8m_vs_8-10w	SE	Rpl36	ENSMUSG00000057863.5	chr17	+	56613906	56614041	56613585	56613680	56614123	56614243	19356	273,243	16,11	380,134	5,0	283	149	2.20249825111e-05	0.0034548208957	0.9,0.921	0.976,1.0	-0.077
6-8m_vs_8-10w	SE	Spata24	ENSMUSG00000024352.11	chr18	-	35660443	35660574	35660016	35660353	35660791	35660857	19373	3,6	2,2	6,4	0,0	279	149	0.000329166056675	0.0217520515971	0.445,0.616	1.0,1.0	-0.47
6-8m_vs_8-10w	SE	Rgs19	ENSMUSG00000002458.13	chr2	-	181689376	181689611	181688421	181689295	181689701	181689776	19519	27,12	6,3	5,2	7,4	298	149	0.000416831526342	0.0250651185671	0.692,0.667	0.263,0.2	0.448
6-8m_vs_8-10w	SE	Mrps26	ENSMUSG00000037740.8	chr2	+	130564080	130564227	130563741	130563990	130564307	130564431	19523	174,65	38,13	188,91	14,6	295	149	9.17286244549e-05	0.00941200160701	0.698,0.716	0.872,0.885	-0.172
6-8m_vs_8-10w	SE	Mrps26	ENSMUSG00000037740.8	chr2	+	130564080	130564431	130563741	130563990	130564937	130565394	19524	258,130	73,61	244,118	34,25	298	149	0.000964146873999	0.042375015323	0.639,0.516	0.782,0.702	-0.164
6-8m_vs_8-10w	SE	Coq9	ENSMUSG00000031782.15	chr8	+	94853604	94853658	94853114	94853270	94854113	94854124	19615	13,2	9,1	17,8	1,0	202	149	8.10888823488e-06	0.00164825983893	0.516,0.596	0.926,1.0	-0.407
6-8m_vs_8-10w	SE	RP24-63B17.4	ENSMUSG00000115483.1	chr14	+	43300657	43300839	43299845	43299977	43304880	43306004	19619	4,6	0,0	4,9	1,5	298	149	4.59384139251e-06	0.00107891352453	1.0,1.0	0.667,0.474	0.43
6-8m_vs_8-10w	SE	Fam129c	ENSMUSG00000043243.15	chr8	+	71606660	71606744	71604927	71605035	71607504	71607924	19664	7,0	1,5	4,4	1,0	232	149	0.000965827351365	0.042375015323	0.818,0.0	0.72,1.0	-0.451
6-8m_vs_8-10w	SE	RP23-62O7.9	ENSMUSG00000110423.1	chr19	+	8735986	8736093	8735840	8735905	8736175	8736297	19758	58,28	12,7	75,47	5,0	255	149	0.000172619196374	0.014862836758	0.739,0.7	0.898,1.0	-0.23
6-8m_vs_8-10w	SE	Dnajb14	ENSMUSG00000074212.7	chr3	+	137905884	137906057	137904744	137904854	137908344	137916557	19890	821,419	86,61	559,336	131,59	298	149	0.000436186069234	0.0257984379365	0.827,0.774	0.681,0.74	0.09
6-8m_vs_8-10w	SE	Tfdp2	ENSMUSG00000032411.15	chr9	+	96247049	96247088	96223093	96223198	96273838	96273942	20012	0,0	35,22	2,4	10,13	187	149	0.00036108191626	0.0231467202048	0.0,0.0	0.137,0.197	-0.167
6-8m_vs_8-10w	SE	Rfc2	ENSMUSG00000023104.9	chr5	+	134596881	134596995	134595218	134595363	134597848	134597955	20039	48,24	3,5	38,31	0,0	262	149	0.000117916881863	0.0111711462475	0.901,0.732	1.0,1.0	-0.184
6-8m_vs_8-10w	SE	Pmm1	ENSMUSG00000022474.15	chr15	-	81951923	81952039	81951107	81951612	81952724	81952800	20091	718,271	19,20	547,375	4,2	264	149	3.47874499096e-07	0.00014700443964 4	0.955,0.884	0.987,0.991	-0.07
6-8m_vs_8-10w	SE	Ift20	ENSMUSG00000001105.15	chr11	+	78538509	78538738	78536360	78536576	78539959	78540045	20210	439,403	47,68	423,266	26,14	298	149	4.44843267587e-05	0.00580755543977	0.824,0.748	0.891,0.905	-0.112
6-8m_vs_8-10w	SE	Ift20	ENSMUSG00000001105.15	chr11	+	78538833	78538962	78536442	78536576	78539959	78540045	20212	1763,1631	47,68	1708,1144	26,14	277	149	9.10980730262e-06	0.00180599118106	0.953,0.928	0.972,0.978	-0.034
6-8m_vs_8-10w	SE	Map7d2	ENSMUSG00000041020.14	chrX	+	159496034	159496309	159492897	159493084	159497583	159498583	20274	13,7	1,0	4,8	9,7	298	149	3.25487652053e-07	0.00013937815244 4	0.867,1.0	0.182,0.364	0.661

6-8m_vs_8-10w	SE	Tatdn3	ENSMUSG00000026632.17	chr1	-	191052859	191052972	191049281	191049359	191054855	191054911	20383	11,7	0,0	19,8	5,1	261	149	5.87095775501e-05	0.00698339552814	1.0,1.0	0.684,0.82	0.248
6-8m_vs_8-10w	SE	Nmd3	ENSMUSG00000027787.14	chr3	+	69735992	69736089	69731564	69731655	69746728	69746835	20489	1,1	0,0	0,0	1,1	245	149	1.97806967472e-08	1.35165288667e-05	1.0,1.0	0.0,0.0	1.0
6-8m_vs_8-10w	SE	Nmd3	ENSMUSG00000027787.14	chr3	+	69741508	69741654	69731564	69731655	69746728	69746835	20494	4,3	0,0	0,0	1,1	294	149	2.27196039759e-09	3.04026167204e-06	1.0,1.0	0.0,0.0	1.0
6-8m_vs_8-10w	SE	Dclk2	ENSMUSG00000028078.14	chr3	-	86788621	86788634	86786152	86787412	86791994	86792315	20637	5,2	34,10	8,8	4,4	161	149	1.94025044997e-06	0.000546605995186	0.12,0.156	0.649,0.649	-0.511
6-8m_vs_8-10w	SE	Usp48	ENSMUSG00000043411.15	chr4	+	137634897	137635008	137633342	137633465	137644342	137644498	20679	3,0	1,2	9,2	0,0	259	149	0.000541813602076	0.0291472121345	0.633,0.0	1.0,1.0	-0.684
6-8m_vs_8-10w	SE	Usp48	ENSMUSG00000043411.15	chr4	+	137636933	137636987	137634897	137635008	137637764	137637890	20681	6,7	6,3	17,5	1,1	202	149	0.00116222585362	0.0481005741171	0.425,0.633	0.926,0.787	-0.328
6-8m_vs_8-10w	SE	Uchl3	ENSMUSG00000022111.9	chr14	+	101666991	101667077	101665760	101665889	101695158	101695217	20757	3,1	2,4	1,1	0,0	234	149	1.00452857921e-07	5.46804065253e-05	0.489,0.137	1.0,1.0	-0.687
6-8m_vs_8-10w	SE	Uchl3	ENSMUSG00000022111.9	chr14	+	101668528	101668576	101665760	101665889	101690571	101690647	20760	284,353	8,9	333,333	0,2	196	149	0.00118311130985	0.048483765083	0.964,0.968	1.0,0.992	-0.03
6-8m_vs_8-10w	SE	Uchl3	ENSMUSG00000022111.9	chr14	+	101685570	101685762	101665795	101665889	101690571	101690647	20764	28,39	8,9	36,23	0,2	298	149	8.89050052534e-05	0.00923593460185	0.636,0.684	1.0,0.852	-0.266
6-8m_vs_8-10w	SE	RP24-147G7.1	ENSMUSG00000047361.16	chr1	+	59632213	59632416	59629994	59630326	59633146	59633319	20793	16,4	3,4	9,6	1,0	298	149	0.00103993164611	0.0447101000622	0.727,0.333	0.818,1.0	-0.379
6-8m_vs_8-10w	SE	Tcf25	ENSMUSG00000001472.17	chr8	+	123397006	123397094	123395550	123395710	123398515	123398606	20850	6,17	8,6	6,6	1,0	236	149	0.000277733424383	0.01978046505	0.321,0.641	0.791,1.0	-0.415
6-8m_vs_8-10w	SE	Tcf25	ENSMUSG00000001472.17	chr8	+	123397982	123398141	123395550	123395710	123398515	123398606	20851	30,26	8,6	27,26	1,0	298	149	7.1368647595e-06	0.00149808855305	0.652,0.684	0.931,1.0	-0.298
6-8m_vs_8-10w	SE	Pah	ENSMUSG00000020051.17	chr10	+	87578852	87578986	87578259	87578355	87581847	87581963	20955	33,17	3,4	25,27	1,0	282	149	0.000903594598275	0.040530508545	0.853,0.692	0.93,1.0	-0.193
6-8m_vs_8-10w	SE	Mroh8	ENSMUSG00000074627.11	chr2	-	157216909	157217032	157214899	157214999	157222763	157222904	21114	56,41	2,6	55,40	0,0	271	149	0.000224175440977	0.0172239676134	0.939,0.79	1.0,1.0	-0.135
6-8m_vs_8-10w	SE	Mroh8	ENSMUSG00000074627.11	chr2	-	157220059	157220215	157214899	157214999	157222763	157222904	21115	15,22	2,6	24,14	0,0	298	149	1.21789868444e-05	0.00226092682945	0.789,0.647	1.0,1.0	-0.282
6-8m_vs_8-10w	SE	Tmem161b	ENSMUSG00000035762.11	chr13	+	84292378	84292553	84260142	84260240	84293380	84293477	21169	127,75	0,0	60,68	7,1	298	149	0.000218385434646	0.0169484375579	1.0,1.0	0.811,0.971	0.109
6-8m_vs_8-10w	SE	AC123684.1	ENSMUSG00000036873.13	chr3	+	145938542	145938669	145938041	145938328	145938897	145939096	21174	106,43	175,113	108,107	130,100	275	149	0.000444124063864	0.0260705962205	0.247,0.171	0.31,0.367	-0.13
6-8m_vs_8-10w	SE	Ppp2r2a	ENSMUSG00000022052.9	chr14	-	67037937	67038008	67028864	67029030	67038874	67038972	21181	30,18	59,26	10,4	49,38	219	149	8.45547160258e-05	0.00899191807909	0.257,0.32	0.122,0.067	0.194
6-8m_vs_8-10w	SE	RP23-433F5.1	ENSMUSG00000097248.9	chr8	+	87494856	87495157	87472843	87472960	87495709	87495861	21184	11,13	0,2	1,5	3,4	298	149	0.000144363481545	0.0129663561278	1.0,0.765	0.143,0.385	0.619

6-8m_vs_8-10w	SE	Senp7	ENSMUSG00000052917.14	chr16	+	56187469	56187570	56186128	56186209	56188234	56188719	21233	44,35	62,34	29,5	82,48	249	149	2.11732473506e-05	0.0033567294439 5	0.298,0.381	0.175,0.059	0.223
6-8m_vs_8-10w	SE	Dppa4	ENSMUSG00000058550.14	chr16	+	48292910	48293106	48291013	48291314	48293698	48294292	21359	1853,1463	139,112	2430,1736	80,89	298	149	1.62469505781e-05	0.0028093181352 8	0.87,0.867	0.938,0.907	-0.054
6-8m_vs_8-10w	SE	Rbm39	ENSMUSG00000027620.16	chr2	-	156177751	156177906	156177335	156177385	156179176	156179240	21416	55,51	108,110	9,15	61,53	298	149	0.000774680666685	0.0362677030485	0.203,0.188	0.069,0.124	0.099
6-8m_vs_8-10w	SE	Rbm39	ENSMUSG00000027620.16	chr2	-	156178879	156178952	156177632	156177906	156179176	156179240	21419	703,405	54,50	532,379	7,13	221	149	6.0096227994e-10	1.48465419866e-06	0.898,0.845	0.981,0.952	-0.095
6-8m_vs_8-10w	SE	Rbm25	ENSMUSG00000010608.15	chr12	+	83639127	83639375	83632305	83632417	83642401	83642519	21523	11,7	6,0	6,3	0,0	298	149	0.000811216683271	0.0374325215516	0.478,1.0	1.0,1.0	-0.261
6-8m_vs_8-10w	SE	RP23-147K15.4	ENSMUSG00000087403.9	chrX	-	152317354	152317434	152315215	152315419	152327160	152327239	21636	2,1	8,5	1,3	1,0	228	149	0.000565264080635	0.0297119823464	0.14,0.116	0.395,1.0	-0.57
6-8m_vs_8-10w	SE	Rab34	ENSMUSG00000002059.18	chr11	+	78189570	78189662	78188439	78188511	78190129	78190135	21683	27,8	6,0	20,13	0,0	240	149	0.000449634839549	0.0261602762807	0.736,1.0	1.0,1.0	-0.132
6-8m_vs_8-10w	SE	Rab34	ENSMUSG00000002059.18	chr11	+	78191574	78191682	78191364	78191455	78191773	78191838	21703	147,94	37,22	115,86	13,5	256	149	0.000306905817132	0.0207944878123	0.698,0.713	0.837,0.909	-0.167
6-8m_vs_8-10w	SE	Fanca	ENSMUSG00000032815.16	chr8	-	123270231	123270294	123269859	123269965	123274147	123274223	21717	4,2	0,0	5,3	4,1	211	149	2.48601451572e-05	0.0037190747932 4	1.0,1.0	0.469,0.679	0.426
6-8m_vs_8-10w	SE	Snx14	ENSMUSG00000032422.17	chr9	-	88398238	88398371	88394366	88394568	88401919	88401978	21828	13,4	7,3	7,17	0,0	281	149	1.834467247e-07	8.79418883437e-05	0.496,0.414	1.0,1.0	-0.545
6-8m_vs_8-10w	SE	Spata21	ENSMUSG00000045004.3	chr4	+	141107143	141107215	141104829	141104967	141107326	141107444	21864	19,10	6,2	9,11	0,0	220	149	3.50295129211e-05	0.0048115829942 2	0.682,0.772	1.0,1.0	-0.273
6-8m_vs_8-10w	SE	Tfb2m	ENSMUSG00000026492.11	chr1	-	179544897	179544986	179542306	179542457	179545821	179546267	21899	597,361	32,22	394,336	41,46	237	149	0.000222491138681	0.0171355525417	0.921,0.912	0.858,0.821	0.077
6-8m_vs_8-10w	SE	Tfb2m	ENSMUSG00000026492.11	chr1	-	179545366	179545458	179533654	179533722	179545821	179545892	21900	21,12	0,0	14,10	1,4	240	149	0.000610684664052	0.0310328301751	1.0,1.0	0.897,0.608	0.248
6-8m_vs_8-10w	SE	Agrp	ENSMUSG00000005705.10	chr8	-	105567119	105567202	105566697	105566938	105567359	105567492	21906	39,10	3,0	16,15	3,16	231	149	0.000837429132746	0.0383117863636	0.893,1.0	0.775,0.377	0.37
6-8m_vs_8-10w	SE	Agrp	ENSMUSG00000005705.10	chr8	-	105567605	105567726	105567359	105567492	105579649	105579845	21907	43,16	1,0	30,11	4,4	269	149	0.000144537161968	0.0129663561278	0.96,1.0	0.806,0.604	0.275
6-8m_vs_8-10w	SE	Med12l	ENSMUSG00000056476.13	chr3	+	59039165	59039198	59037559	59037751	59042253	59042413	21991	5,9	0,8	10,7	0,0	181	149	0.000481485482954	0.0271876506443	1.0,0.481	1.0,1.0	-0.26
6-8m_vs_8-10w	SE	Baz2a	ENSMUSG00000040054.16	chr10	+	128108893	128109031	128096379	128096523	128110754	128111348	22105	20,7	6,0	8,3	0,0	286	149	0.000405708280817	0.0247615651374	0.635,1.0	1.0,1.0	-0.183
6-8m_vs_8-10w	SE	Orc6	ENSMUSG00000031697.12	chr8	+	85305208	85305321	85303361	85303451	85306216	85306318	22155	6974,5295	141,155	4938,4186	190,163	261	149	0.000305227219049	0.0207561708573	0.966,0.951	0.937,0.936	0.022
6-8m_vs_8-10w	SE	Ptdss2	ENSMUSG00000025495.15	chr7	+	141147080	141147148	141135308	141135410	141154556	141154702	22174	12,8	4,1	5,13	0,0	216	149	0.00046867451333	0.0267828303739	0.674,0.847	1.0,1.0	-0.24

6-8m_vs_8-10w	SE	Ptdss2	ENSMUSG00000025495.15	chr7	+	141151649	141151784	141135308	141135410	141154556	141154702	22182	15,7	4,1	9,3	0,0	283	149	0.000278389905424	0.01978046505	0.664,0.787	1.0,1.0	-0.274
6-8m_vs_8-10w	SE	Mus81	ENSMUSG00000024906.9	chr19	-	5483634	5483665	5483454	5483558	5483929	5484025	22210	6,5	1,4	3,2	0,0	179	149	0.000196575668257	0.0160023263425	0.833,0.51	1.0,1.0	-0.329
6-8m_vs_8-10w	SE	Far1	ENSMUSG00000030759.16	chr7	+	113553641	113553813	113551234	113551353	113566384	113566512	22307	3,5	2,2	3,4	0,0	298	149	0.000166404862407	0.0143662864545	0.429,0.556	1.0,1.0	-0.507
6-8m_vs_8-10w	SE	Fuom	ENSMUSG00000025466.19	chr7	-	140101331	140101402	140100556	140101188	140101600	140101669	22404	21,18	0,0	8,10	7,2	219	149	1.66525085643e-06	0.000486192695501	1.0,1.0	0.437,0.773	0.395
6-8m_vs_8-10w	SE	Tmem209	ENSMUSG00000029782.19	chr6	-	30501900	30502102	30497817	30497993	30505732	30505831	22420	224,94	1,1	89,75	4,6	298	149	0.000194946518915	0.0159716897997	0.991,0.979	0.918,0.862	0.095
6-8m_vs_8-10w	SE	Rnf185	ENSMUSG00000020448.16	chr11	-	3428883	3428902	3426565	3426678	3452208	3452334	22446	5,6	2,5	1,3	0,0	167	149	9.66189653184e-06	0.0018920821281 5	0.69,0.517	1.0,1.0	-0.397
6-8m_vs_8-10w	SE	Rnf185	ENSMUSG00000020448.16	chr11	-	3432399	3432620	3426565	3426678	3452208	3452363	22448	27,23	2,5	10,17	0,0	298	149	0.000155159866427	0.0135779680386	0.871,0.697	1.0,1.0	-0.216
6-8m_vs_8-10w	SE	Rab25	ENSMUSG00000008601.12	chr3	-	88543319	88543513	88542700	88542781	88548032	88548249	22451	5,4	0,0	9,3	2,2	298	149	0.00066992780131	0.0330133035861	1.0,1.0	0.692,0.429	0.44
6-8m_vs_8-10w	SE	Adrm1	ENSMUSG00000039041.15	chr2	+	180175853	180175956	180174864	180174946	180176073	180176282	22480	108,49	4,6	92,62	0,1	251	149	0.000553832387186	0.0294484783889	0.941,0.829	1.0,0.974	-0.102
6-8m_vs_8-10w	SE	Ank3	ENSMUSG00000069601.14	chr10	+	70001922	70002231	69999354	69999498	70002510	70002601	22514	3,10	1,0	2,1	3,4	298	149	6.58761595662e-06	0.00141045249375	0.6,1.0	0.25,0.111	0.62
6-8m_vs_8-10w	SE	Mfsd10	ENSMUSG00000001082.12	chr5	-	34634590	34634644	34634422	34634513	34634850	34634962	22524	11,8	0,2	2,1	2,4	202	149	0.000120687107807	0.0113445293809	1.0,0.747	0.425,0.156	0.583
6-8m_vs_8-10w	SE	Parp2	ENSMUSG00000036023.6	chr14	+	50817331	50817470	50816972	50817123	50819209	50819273	22546	147,84	14,0	154,114	1,1	287	149	0.000305694009699	0.0207561708573	0.845,1.0	0.988,0.983	-0.063
6-8m_vs_8-10w	SE	Spg7	ENSMUSG00000000738.18	chr8	+	123079968	123080131	123079413	123079539	123080636	123080810	22559	6,2	13,7	10,7	1,3	298	149	0.000284310440974	0.0199801184296	0.188,0.125	0.833,0.538	-0.529
6-8m_vs_8-10w	SE	Trnau1ap	ENSMUSG00000028898.15	chr4	-	132321678	132321810	132314204	132314367	132325207	132325260	22862	204,125	22,12	78,88	23,20	280	149	0.00052548417706	0.028638998816	0.831,0.847	0.643,0.701	0.167
6-8m_vs_8-10w	SE	Azin1	ENSMUSG00000037458.15	chr15	-	38495272	38495354	38494291	38494366	38497263	38497388	22949	357,188	0,0	260,184	7,7	230	149	0.000228569251909	0.017477928796	1.0,1.0	0.96,0.945	0.048
6-8m_vs_8-10w	SE	Pkmyt1	ENSMUSG00000023908.7	chr17	+	23735199	23735372	23732336	23732824	23736323	23736401	22976	1,2	4,3	2,3	0,0	298	149	8.25711943264e-10	1.89418319785e-06	0.111,0.25	1.0,1.0	-0.82
6-8m_vs_8-10w	SE	Oma1	ENSMUSG00000035069.3	chr4	+	103328837	103328966	103325292	103325400	103331304	103331379	23046	11,7	1,0	3,1	1,6	277	149	2.42525240682e-05	0.00365677963838	0.855,1.0	0.617,0.082	0.578
6-8m_vs_8-10w	SE	Psmd14	ENSMUSG00000026914.15	chr2	+	61720443	61720571	61711693	61711873	61776672	61776780	23168	184,183	7,22	185,166	2,2	276	149	6.42796903205e-06	0.00139486927995	0.934,0.818	0.98,0.978	-0.103
6-8m_vs_8-10w	SE	Psmd14	ENSMUSG00000026914.15	chr2	+	61722709	61722761	61720443	61720571	61797394	61797457	23178	89,57	3,7	92,73	0,0	200	149	0.000186821184762	0.0157066732194	0.957,0.858	1.0,1.0	-0.093

6-8m_vs_8-10w	SE	Psmd14	ENSMUSG00000026914.15	chr2	+	61764848	61764999	61720443	61720571	61797394	61797457	23194	5,14	3,7	10,2	0,0	298	149	2.21159304636e-06	0.000612306226525	0.455,0.5	1.0,1.0	-0.523
6-8m_vs_8-10w	SE	Psmd14	ENSMUSG00000026914.15	chr2	+	61764848	61764999	61760974	61761094	61797394	61797457	23201	6,13	0,0	12,2	6,1	298	149	1.87667720553e-06	0.000536022758949	1.0,1.0	0.5,0.5	0.5
6-8m_vs_8-10w	SE	Psmd14	ENSMUSG00000026914.15	chr2	+	61776672	61776780	61720443	61720571	61797394	61797457	23206	9,38	3,7	10,11	0,0	256	149	3.50576167844e-05	0.0048115829942	0.636,0.76	1.0,1.0	-0.302
6-8m_vs_8-10w	SE	Psmd14	ENSMUSG00000026914.15	chr2	+	61776672	61776780	61760974	61761094	61797394	61797457	23211	3,3	0,0	4,0	6,1	256	149	1.21553764831e-08	9.29481121741e-06	1.0,1.0	0.28,0.0	0.86
6-8m_vs_8-10w	SE	Wdr46	ENSMUSG00000024312.9	chr17	+	33943800	33943936	33943557	33943708	33944481	33944581	23243	2,1	2,1	4,2	0,0	284	149	8.1831361367e-05	0.0088191140995 4	0.344,0.344	1.0,1.0	-0.656
6-8m_vs_8-10w	SE	Pmvk	ENSMUSG00000027952.16	chr3	+	89461881	89461945	89459117	89459464	89467546	89467676	23258	9,1	0,0	0,1	4,0	212	149	0.000308634755967	0.0208676080477	1.0,1.0	0.0,1.0	0.5
6-8m_vs_8-10w	SE	RP23-424N5.2	ENSMUSG00000085427.7	chr2	-	72794928	72795014	72792176	72792275	72800069	72800154	23360	20,11	152,96	1,3	71,72	234	149	0.000227748158291	0.0174567060899	0.077,0.068	0.009,0.026	0.055
6-8m_vs_8-10w	SE	Il17f	ENSMUSG00000041872.9	chr1	-	20788605	20788755	20784494	20784659	20790487	20790585	23440	30,28	10,13	6,14	13,19	298	149	0.000177497779799	0.0150807372911	0.6,0.519	0.188,0.269	0.331
6-8m_vs_8-10w	SE	AC134411.2	ENSMUSG00000089798.9	chr5	+	107546200	107546279	107545687	107545856	107548116	107548220	23542	9,15	3,5	8,6	0,0	227	149	2.41697759427e-05	0.0036567796383 8	0.663,0.663	1.0,1.0	-0.337
6-8m_vs_8-10w	SE	Mpc1	ENSMUSG00000023861.17	chr17	+	8288599	8288738	8283865	8283961	8296534	8296631	23548	133,90	55,38	73,58	57,70	287	149	9.41853869082e-05	0.0095723350821	0.557,0.551	0.399,0.301	0.204
6-8m_vs_8-10w	SE	Riox2	ENSMUSG00000022724.15	chr16	+	59486532	59486635	59483004	59483108	59487427	59487599	23585	173,68	0,1	174,102	4,9	251	149	0.000291428938531	0.0204141055594	1.0,0.976	0.963,0.871	0.071
6-8m_vs_8-10w	SE	Slc25a39	ENSMUSG00000018677.9	chr11	-	102403632	102403713	102402984	102403535	102403790	102403872	23631	67,52	29,12	73,45	5,4	229	149	7.6663494073e-05	0.0084609098819 5	0.601,0.738	0.905,0.88	-0.223
6-8m_vs_8-10w	SE	Ap4m1	ENSMUSG00000019518.10	chr5	+	138176051	138176118	138175911	138175974	138176209	138176263	23641	12,4	6,2	6,9	0,0	215	149	5.45796306328e-06	0.0012257898023 8	0.581,0.581	1.0,1.0	-0.419
6-8m_vs_8-10w	SE	Ddx60	ENSMUSG00000037921.15	chr8	+	62012270	62012471	62009941	62010046	62017140	62017245	23863	6,5	1,0	4,0	4,2	298	149	3.11082325701e-05	0.0044206725540 8	0.75,1.0	0.333,0.0	0.709
6-8m_vs_8-10w	SE	CH36-17G18.1	ENSMUSG00000100091.1	chrY	+	41273948	41274047	41271823	41271923	41274406	41274433	23934	50,64	1,2	51,20	10,2	247	149	0.000638539018575	0.0320928311746	0.968,0.951	0.755,0.858	0.153
6-8m_vs_8-10w	SE	RP24-103K4.2	ENSMUSG00000087700.2	chr12	-	73928822	73929100	73926480	73926754	73949623	73949785	24046	5,3	0,1	2,1	2,3	298	149	0.000118794974377	0.0112212335209	1.0,0.6	0.333,0.143	0.562
6-8m_vs_8-10w	SE	Tra2b	ENSMUSG00000022858.15	chr16	-	22258985	22259261	22254983	22255117	22265740	22265829	24087	746,707	54,19	773,455	93,31	298	149	0.000388098956262	0.024108677136	0.874,0.949	0.806,0.88	0.069
6-8m_vs_8-10w	SE	R3hdm1	ENSMUSG00000056211.13	chr1	+	128162201	128162243	128153188	128153399	128162768	128162858	24292	22,18	74,37	4,4	60,41	190	149	2.79590693092e-05	0.0040447453600 6	0.189,0.276	0.05,0.071	0.172
6-8m_vs_8-10w	SE	Zfy1	ENSMUSG00000053211.10	chrY	-	732927	733068	729613	729736	735018	735168	24387	7,13	3,4	29,28	1,1	289	149	0.000713231487595	0.034504011075	0.546,0.626	0.937,0.935	-0.35

6-8m_vs_8-10w	SE	Cfap157	ENSMUSG00000038987.8	chr2	-	32777827	32778017	32777380	32777569	32778116	32778283	24391	3,2	4,5	5,4	0,0	298	149	3.1160942715e-09	3.57416012941e-06	0.273,0.167	1.0,1.0	-0.78
6-8m_vs_8-10w	SE	Nek3	ENSMUSG00000031478.16	chr8	-	22142889	22142939	22132992	22133095	22146930	22147003	24394	4,2	1,0	0,0	2,1	198	149	2.37776809353e-09	3.04631551948e-06	0.751,1.0	0.0,0.0	0.876
6-8m_vs_8-10w	SE	Loxhd1	ENSMUSG00000032818.15	chr18	+	77295732	77295917	77293233	77293314	77321572	77321671	24429	35,13	0,0	7,10	6,1	298	149	1.36904877264e-05	0.0024687705128 7	1.0,1.0	0.368,0.833	0.399
6-8m_vs_8-10w	SE	Gpr107	ENSMUSG00000000194.13	chr2	+	31177784	31177895	31176897	31176954	31178253	31178387	24464	23,10	1,5	9,9	0,0	259	149	0.000209077893	0.0166032095462	0.93,0.535	1.0,1.0	-0.267
6-8m_vs_8-10w	SE	Cab39l	ENSMUSG00000021981.10	chr14	+	59479043	59479184	59461634	59461702	59496802	59496944	24541	14,15	108,87	8,5	154,109	289	149	0.0011161223057	0.0467781815426	0.063,0.082	0.026,0.023	0.048
6-8m_vs_8-10w	SE	Pja2	ENSMUSG00000024083.15	chr17	-	64297723	64297909	64292838	64293024	64308621	64309666	24610	8,3	7,5	5,3	0,1	298	149	0.000369489848572	0.0235915228166	0.364,0.231	1.0,0.6	-0.503
6-8m_vs_8-10w	SE	RP23-17O9.4	ENSMUSG00000074758.10	chr2	-	144174300	144174403	144168482	144168626	144174526	144174744	24632	6,9	5,1	4,4	0,0	251	149	5.70895193061e-05	0.0068413694105 8	0.416,0.842	1.0,1.0	-0.371
6-8m_vs_8-10w	SE	Ndufaf7	ENSMUSG00000024082.4	chr17	+	78942068	78942282	78939594	78939705	78943252	78943311	24639	173,159	64,50	113,127	95,67	298	149	0.0011430323744 4	0.0476748412176	0.575,0.614	0.373,0.487	0.165
6-8m_vs_8-10w	SE	Smu1	ENSMUSG00000028409.11	chr4	-	40739518	40739686	40736541	40737395	40744030	40744158	24649	3,5	0,1	1,2	5,1	298	149	8.91499478943e-05	0.0092359346018 5	1.0,0.714	0.091,0.5	0.562
6-8m_vs_8-10w	SE	Rbm28	ENSMUSG00000029701.15	chr6	-	29127797	29127900	29123575	29125524	29128428	29128685	24664	106,64	0,2	114,64	6,6	251	149	0.0010356835942 5	0.0446469990778	1.0,0.95	0.919,0.864	0.084
6-8m_vs_8-10w	SE	Gtf2i	ENSMUSG00000060261.15	chr5	-	134239496	134239571	134237833	134238892	134240242	134240284	24711	28,7	0,0	10,14	4,1	223	149	0.000770283527002	0.0361672891129	1.0,1.0	0.626,0.903	0.236
6-8m_vs_8-10w	SE	Nlrp4b	ENSMUSG00000034087.14	chr7	+	10714148	10715703	10688061	10688160	10724911	10725082	24743	11,2	3,4	6,1	0,0	298	149	1.08365587226e-06	0.000370241404186	0.647,0.2	1.0,1.0	-0.577
6-8m_vs_8-10w	SE	Cldn34c1	ENSMUSG00000079450.11	chrX	+	123122897	123122938	123117453	123117569	123142609	123143270	24829	1,0	49,40	6,1	12,27	189	149	0.000867425052922	0.0391267176961	0.016,0.0	0.283,0.028	-0.148
6-8m_vs_8-10w	SE	Ggta1	ENSMUSG00000035778.17	chr2	-	35414218	35414284	35413325	35413427	35422179	35422268	25041	17,18	3,5	7,4	0,0	214	149	0.000148414734992	0.01327015976	0.798,0.715	1.0,1.0	-0.244
6-8m_vs_8-10w	SE	RP23-440J15.6	ENSMUSG00000092463.1	chrX	-	101265629	101265726	101265375	101265442	101266180	101266343	25098	19,7	8,3	3,10	0,1	245	149	0.000551567108858	0.0294254639005	0.591,0.587	1.0,0.859	-0.341
6-8m_vs_8-10w	SE	Ly6e	ENSMUSG00000022587.14	chr15	+	74957414	74957463	74956554	74956713	74957779	74957877	25141	0,0	1,3	3,1	0,0	197	149	1.27372334902e-09	2.27260550428e-06	0.0,0.0	1.0,1.0	-1.0
6-8m_vs_8-10w	SE	Mfsd4	ENSMUSG00000059149.17	chr1	-	132053548	132053664	132052379	132052553	132054058	132054224	25173	1,0	2,3	1,4	0,0	264	149	1.09144915328e-10	4.38162262584e-07	0.22,0.0	1.0,1.0	-0.89
6-8m_vs_8-10w	SE	Hnrnpc	ENSMUSG00000060373.15	chr14	-	52097321	52098544	52084114	52084391	52103888	52104004	25241	295,280	279,159	206,124	271,192	298	149	0.000338342511966	0.0220193627269	0.346,0.468	0.275,0.244	0.148
6-8m_vs_8-10w	SE	Hnrnpc	ENSMUSG00000060373.15	chr14	-	52098014	52098040	52083868	52084391	52103888	52103999	25246	611,334	279,159	322,272	271,192	174	149	0.000105617454991	0.0105341931195	0.652,0.643	0.504,0.548	0.121

6-8m_vs_8-10w	SE	Hnrnpc	ENSMUSG00000060373.15	chr14	-	52098014	52098544	52084271	52084391	52103888	52103993	25247	456,385	279,159	265,201	271,192	298	149	2.11308101544e-05	0.0033567294439 5	0.45,0.548	0.328,0.344	0.163
6-8m_vs_8-10w	SE	Hnrnpc	ENSMUSG00000060373.15	chr14	-	52098315	52098544	52084114	52084391	52103888	52103990	25248	200,232	279,159	147,87	271,192	298	149	0.0010831717338 4	0.0457725571105	0.264,0.422	0.213,0.185	0.144
6-8m_vs_8-10w	SE	Klf17	ENSMUSG00000048626.5	chr4	-	117760314	117761113	117757835	117759288	117765500	117765648	25315	368,138	10,14	194,164	3,1	298	149	0.000471401005742	0.0267909973794	0.948,0.831	0.97,0.988	-0.09
6-8m_vs_8-10w	SE	Ndufs4	ENSMUSG00000021764.7	chr13	-	114307816	114307890	114287794	114288866	114316856	114317029	25389	83,56	2,2	61,49	9,6	222	149	0.0012369989126 9	0.0497214731914	0.965,0.949	0.82,0.846	0.124
6-8m_vs_8-10w	SE	Serpinb1c	ENSMUSG00000079049.9	chr13	-	32884220	32884363	32883876	32884044	32888871	32889009	25425	6,2	0,0	1,2	2,1	291	149	0.000756439107415	0.0358844879967	1.0,1.0	0.204,0.506	0.645
6-8m_vs_8-10w	SE	Qrsl1	ENSMUSG00000019863.7	chr10	-	43876471	43876677	43874187	43874706	43881472	43881590	25478	4,4	0,0	12,7	7,2	298	149	1.38383187631e-06	0.000422127906596	1.0,1.0	0.462,0.636	0.451
6-8m_vs_8-10w	SE	Zfp229	ENSMUSG00000061544.13	chr17	+	21734620	21734739	21733679	21733958	21736057	21736081	25661	3,2	3,2	0,0	8,1	267	149	0.000301042254932	0.0205707937434	0.358,0.358	0.0,0.0	0.358
6-8m_vs_8-10w	SE	Dhps	ENSMUSG00000060038.14	chr8	+	85073502	85073599	85073057	85073416	85073680	85073767	25732	95,57	10,4	82,41	2,0	245	149	0.000632245649601	0.0318263342987	0.852,0.897	0.961,1.0	-0.106
6-8m_vs_8-10w	SE	Gpat2	ENSMUSG00000046338.4	chr2	+	127434791	127434923	127434387	127434465	127435006	127435127	25840	8,3	0,0	5,4	1,3	280	149	0.000445241108601	0.0260705962205	1.0,1.0	0.727,0.415	0.429
6-8m_vs_8-10w	SE	Rarg	ENSMUSG00000001288.15	chr15	-	102239857	102240034	102239389	102239594	102240127	102240288	25932	8,4	2,5	9,8	1,0	298	149	0.000278039297471	0.01978046505	0.667,0.286	0.818,1.0	-0.433
6-8m_vs_8-10w	SE	Tsks	ENSMUSG00000059891.14	chr7	+	44957596	44957753	44956451	44956587	44957855	44958032	25971	0,0	1,2	3,1	1,0	298	149	1.10528068905e-05	0.0020880702711 5	0.0,0.0	0.6,1.0	-0.8
6-8m_vs_8-10w	SE	Dhx30	ENSMUSG00000032480.17	chr9	-	110085684	110085878	110085144	110085302	110085949	110086031	25976	15,5	2,5	13,4	0,0	298	149	1.09874640168e-05	0.0020880082506 7	0.789,0.333	1.0,1.0	-0.439
6-8m_vs_8-10w	SE	Dhx30	ENSMUSG00000032480.17	chr9	-	110116677	110116755	110115490	110115582	110117357	110117385	25982	5,1	12,2	0,0	5,3	226	149	0.000946380064804	0.0417742525024	0.216,0.248	0.0,0.0	0.232
6-8m_vs_8-10w	SE	Dhx30	ENSMUSG00000032480.17	chr9	-	110116677	110116792	110115490	110115582	110117357	110117431	25983	8,2	12,2	1,0	5,3	263	149	0.000915209192098	0.040766793916	0.274,0.362	0.102,0.0	0.267
6-8m_vs_8-10w	SE	Vta1	ENSMUSG00000019868.16	chr10	-	14675933	14676042	14667931	14668108	14684063	14684139	26132	567,454	7,0	292,252	10,6	257	149	0.000836265317125	0.0383117863636	0.979,1.0	0.944,0.961	0.037
6-8m_vs_8-10w	SE	Sumf1	ENSMUSG00000030101.15	chr6	-	108144590	108144704	108118619	108118679	108173355	108173430	26287	25,10	6,0	7,7	0,0	262	149	0.000481684307404	0.0271876506443	0.703,1.0	1.0,1.0	-0.149
6-8m_vs_8-10w	SE	Cdk9	ENSMUSG00000009555.16	chr2	-	32709466	32709944	32707848	32708289	32710042	32710209	26331	69,23	7,4	55,39	1,0	298	149	6.99785711049e-05	0.0078035826028	0.831,0.742	0.965,1.0	-0.196
6-8m_vs_8-10w	SE	Snhg5	ENSMUSG00000097195.9	chr9	-	88522483	88522586	88521917	88521975	88522850	88522929	26370	1,5	0,1	3,0	2,1	251	149	0.0010634034022 4	0.0451591027273	1.0,0.748	0.471,0.0	0.639
6-8m_vs_8-10w	SE	Clk2	ENSMUSG00000068917.12	chr3	+	89169614	89169702	89168694	89168923	89170048	89170115	26422	6,6	0,0	10,6	3,3	236	149	1.80800348865e-05	0.0029930845382 2	1.0,1.0	0.678,0.558	0.382

6-8m_vs_8-10w	SE	Clk2	ENSMUSG00000068917.12	chr3	+	89169617	89169702	89168694	89168923	89170048	89170115	26424	2,1	0,0	6,11	3,3	233	149	4.99541528804e-05	0.0062425197428 3	1.0,1.0	0.561,0.701	0.369
6-8m_vs_8-10w	SE	Fam188a	ENSMUSG00000026767.12	chr2	-	12403997	12404166	12401024	12401076	12405859	12405920	26462	76,58	2,0	33,35	6,3	298	149	0.000323961505754	0.0214715690827	0.95,1.0	0.733,0.854	0.182
6-8m_vs_8-10w	SE	Fam188a	ENSMUSG00000026767.12	chr2	-	12403997	12404168	12401024	12401076	12405859	12405920	26466	161,110	2,0	67,57	6,3	298	149	0.000246065421058	0.0183464917114	0.976,1.0	0.848,0.905	0.111
6-8m_vs_8-10w	SE	Klf7	ENSMUSG00000025959.13	chr1	-	64120899	64121139	64041925	64042437	64122201	64122282	26500	4,3	2,2	3,1	0,0	298	149	0.000106900520804	0.0105963491548	0.5,0.429	1.0,1.0	-0.536
6-8m_vs_8-10w	SE	Fbxw20	ENSMUSG00000061701.11	chr9	-	109225858	109226068	109224355	109224514	109233464	109233628	26527	160,133	26,17	126,106	4,5	298	149	6.10638904527e-05	0.0071193388617	0.755,0.796	0.94,0.914	-0.151
6-8m_vs_8-10w	SE	Fbxw20	ENSMUSG00000061701.11	chr9	-	109232268	109232387	109224355	109224514	109233464	109233628	26528	181,189	26,17	164,161	4,5	267	149	1.29958133147e-05	0.0023714405705 4	0.795,0.861	0.958,0.947	-0.124
6-8m_vs_8-10w	SE	Arl6ip6	ENSMUSG00000026960.6	chr2	+	53202918	53203051	53194283	53194337	53217294	53217534	26708	445,279	2,1	339,284	7,8	281	149	0.0011875922038 2	0.048483765083	0.992,0.993	0.963,0.95	0.036
6-8m_vs_8-10w	SE	Sept2	ENSMUSG00000026276.20	chr1	+	93501506	93501652	93500457	93500559	93503434	93503518	26722	215,125	7,7	140,121	17,14	294	149	0.0011564026328 5	0.0479868279732	0.94,0.9	0.807,0.814	0.109
6-8m_vs_8-10w	SE	Apoa1bp	ENSMUSG00000028070.7	chr3	-	88056985	88057133	88056519	88056776	88057723	88057837	26724	214,123	78,45	207,164	26,32	296	149	3.77830267331e-05	0.0050984860779 8	0.58,0.579	0.8,0.721	-0.181
6-8m_vs_8-10w	SE	Ipcef1	ENSMUSG00000064065.15	chr10	-	6919713	6920086	6911542	6911958	6935186	6935275	26745	18,10	3,2	7,6	0,0	298	149	0.000858054694541	0.0389666571823	0.75,0.714	1.0,1.0	-0.268
6-8m_vs_8-10w	SE	Yipf1	ENSMUSG00000057375.13	chr4	+	107336113	107336194	107324654	107324721	107336436	107336502	26758	159,98	4,8	117,84	1,0	229	149	0.000819395855159	0.0377251181137	0.963,0.889	0.987,1.0	-0.068
6-8m_vs_8-10w	SE	Yipf1	ENSMUSG00000057375.13	chr4	+	107345008	107345191	107340985	107341102	107359606	107359822	26779	26,17	33,22	34,40	17,18	298	149	0.000777786466676	0.0363601021307	0.283,0.279	0.5,0.526	-0.232
6-8m_vs_8-10w	SE	Myo1g	ENSMUSG00000020437.12	chr11	-	6509324	6509469	6508189	6508294	6510147	6510371	26900	7,2	1,0	2,2	5,3	293	149	2.20525060287e-05	0.0034548208957	0.781,1.0	0.169,0.253	0.68
6-8m_vs_8-10w	SE	RP23-175J8.3	ENSMUSG00000027309.18	chr2	-	130814178	130814303	130812844	130812941	130820468	130820588	26923	62,20	9,0	54,34	0,0	273	149	0.000152417924923	0.0135241758734	0.79,1.0	1.0,1.0	-0.105
6-8m_vs_8-10w	SE	Slc37a3	ENSMUSG00000029924.12	chr6	-	39372078	39372137	39367643	39367766	39372846	39372927	26966	1,0	0,4	4,1	0,0	207	149	0.000330720923903	0.021765231951	1.0,0.0	1.0,1.0	-0.5
6-8m_vs_8-10w	SE	Fam184a	ENSMUSG00000019856.14	chr10	-	53640944	53641062	53633365	53633715	53647571	53647756	27006	4,8	1,1	2,1	3,3	266	149	0.000501947171229	0.0278272816688	0.691,0.818	0.272,0.157	0.54
6-8m_vs_8-10w	SE	Setdb2	ENSMUSG00000071350.12	chr14	-	59426488	59426554	59422520	59423484	59440586	59440875	27038	35,25	0,0	24,19	6,0	214	149	0.0010066366938 4	0.0436470183008	1.0,1.0	0.736,1.0	0.132
6-8m_vs_8-10w	SE	Fkbp1a	ENSMUSG00000032966.14	chr2	+	151544012	151544140	151542817	151542865	151559457	151559616	27092	15,1	0,0	2,2	3,3	276	149	1.31228862443e-07	6.58522835347e-05	1.0,1.0	0.265,0.265	0.735
6-8m_vs_8-10w	SE	Fbxw18	ENSMUSG00000074059.3	chr9	-	109693169	109693379	109691500	109691659	109701240	109701404	27116	505,426	9,10	410,397	0,2	298	149	0.000458343372903	0.0263802074626	0.966,0.955	1.0,0.99	-0.035

6-8m_vs_8-10w	SE	Clec10a	ENSMUSG00000000318.16	chr11	+	70169128	70169224	70168582	70168696	70169307	70169379	27195	8,1	5,3	14,5	0,0	244	149	7.95390423525e-08	4.48153663893e-05	0.494,0.169	1.0,1.0	-0.669
6-8m_vs_8-10w	SE	Tmem191c	ENSMUSG00000055692.20	chr16	+	17276961	17277039	17276836	17276887	17277341	17277401	27242	2,0	4,1	9,1	0,0	226	149	2.35791195458e-08	1.57763959028e-05	0.248,0.0	1.0,1.0	-0.876
6-8m_vs_8-10w	SE	Tmem191c	ENSMUSG00000055692.20	chr16	+	17277193	17277250	17276836	17276887	17277341	17277401	27245	6,2	4,1	6,3	0,0	205	149	6.06158689013e-05	0.0071048877577 9	0.522,0.592	1.0,1.0	-0.443
6-8m_vs_8-10w	SE	Epn2	ENSMUSG00000001036.17	chr11	-	61548007	61548042	61546505	61546911	61565821	61565894	27316	8,9	3,3	6,2	0,0	183	149	0.000218780554333	0.0169484375579	0.685,0.71	1.0,1.0	-0.303
6-8m_vs_8-10w	SE	Phkg2	ENSMUSG00000030815.11	chr7	+	127577974	127578029	127577534	127577688	127579666	127579732	27369	11,7	1,0	12,1	8,1	203	149	4.61772998978e-05	0.0059559444318	0.89,1.0	0.524,0.423	0.472
6-8m_vs_8-10w	SE	Tpra1	ENSMUSG00000002871.14	chr6	+	88910796	88910877	88910340	88910401	88911682	88911747	27613	19,7	7,4	4,7	1,0	229	149	0.00115443023832	0.0479868279732	0.638,0.532	0.722,1.0	-0.276
6-8m_vs_8-10w	SE	St3gal6	ENSMUSG00000022747.16	chr16	-	58523501	58523672	58507447	58507614	58523926	58524134	27630	14,8	2,3	6,7	0,0	298	149	0.000164975774194	0.0142812990944	0.778,0.571	1.0,1.0	-0.326
6-8m_vs_8-10w	SE	Tomm70a	ENSMUSG00000022752.8	chr16	+	57138029	57138178	57136697	57136807	57142720	57142855	27887	12,28	0,0	23,13	7,0	297	149	0.000295995066896	0.0204141055594	1.0,1.0	0.622,1.0	0.189
6-8m_vs_8-10w	SE	Tomm70a	ENSMUSG00000022752.8	chr16	+	57140578	57140786	57136697	57136807	57142720	57142855	27888	23,38	0,0	33,28	7,0	298	149	0.000446469919083	0.0260705962205	1.0,1.0	0.702,1.0	0.149
6-8m_vs_8-10w	SE	Setd2	ENSMUSG00000044791.16	chr9	+	110599118	110599253	110594090	110594760	110602122	110602262	27906	217,144	3,2	159,161	10,9	283	149	0.00071418450536	0.034504011075	0.974,0.974	0.893,0.904	0.075
6-8m_vs_8-10w	SE	Capn11	ENSMUSG00000058626.16	chr17	-	45632008	45632087	45631787	45631901	45632380	45632449	27946	93,49	6,8	74,60	1,1	227	149	0.000284040663676	0.0199801184296	0.911,0.801	0.98,0.975	-0.121
6-8m_vs_8-10w	SE	Gsdmd	ENSMUSG00000022575.5	chr15	+	75866789	75866863	75866295	75866471	75866996	75867408	27980	0,0	3,1	1,2	1,0	222	149	0.000148750078266	0.01327015976	0.0,0.0	0.402,1.0	-0.701
6-8m_vs_8-10w	SE	E4f1	ENSMUSG00000024137.9	chr17	-	24445121	24445313	24444775	24444974	24445445	24445554	28016	4,1	3,1	2,2	0,0	298	149	3.56958572134e-05	0.00485766165367	0.4,0.333	1.0,1.0	-0.634
6-8m_vs_8-10w	SE	E4f1	ENSMUSG00000024137.9	chr17	-	24445121	24445339	24444775	24444974	24445445	24445551	28017	9,2	3,1	1,2	0,0	298	149	0.000497899144577	0.0278096155256	0.6,0.5	1.0,1.0	-0.45
6-8m_vs_8-10w	SE	Dnajc12	ENSMUSG00000036764.12	chr10	+	63395784	63395863	63386562	63386775	63407001	63407206	28047	76,80	0,0	86,50	9,1	227	149	0.000121606171147	0.0113751711555	1.0,1.0	0.862,0.97	0.084
6-8m_vs_8-10w	SE	Wwp2	ENSMUSG00000031930.11	chr8	+	107550046	107550135	107549780	107549852	107552276	107552436	28092	11,10	6,2	13,5	1,0	237	149	0.000575913048004	0.0298832226738	0.535,0.759	0.891,1.0	-0.299
6-8m_vs_8-10w	SE	Man1b1	ENSMUSG00000036646.13	chr2	+	25338112	25338267	25334376	25334492	25341606	25341716	28100	44,14	4,3	17,4	0,0	298	149	0.000315566511693	0.0211140293532	0.846,0.7	1.0,1.0	-0.227
6-8m_vs_8-10w	SE	Pbrm1	ENSMUSG00000042323.17	chr14	+	31110414	31110579	31107108	31107310	31113848	31114004	28124	1,1	3,1	3,2	0,0	298	149	3.7507379036e-07	0.000155792365195	0.143,0.333	1.0,1.0	-0.762
6-8m_vs_8-10w	SE	Cpsf4l	ENSMUSG00000018727.19	chr11	-	113702055	113702095	113699835	113699982	113702412	113702506	28133	34,11	10,0	11,17	0,0	188	149	0.000230853073174	0.0174860313633	0.729,1.0	1.0,1.0	-0.135

6-8m_vs_8-10w	SE	Zdhhc20	ENSMUSG00000021969.8	chr14	-	57846920	57847062	57843187	57843314	57856609	57856730	28146	711,571	1,2	479,545	4,16	290	149	0.000197989601791	0.0160023263425	0.997,0.993	0.984,0.946	0.03
6-8m_vs_8-10w	SE	Zfp655	ENSMUSG00000007812.16	chr5	+	145235736	145235863	145235305	145235391	145242919	145243024	28174	24,13	2,0	3,13	5,4	275	149	0.000300069184714	0.0205480211861	0.867,1.0	0.245,0.638	0.492
6-8m_vs_8-10w	SE	Raet1d	ENSMUSG00000078452.10	chr10	+	22370865	22371114	22362687	22362805	22371366	22371630	28405	36,38	3,0	15,11	4,6	298	149	0.000194579120578	0.0159716897997	0.857,1.0	0.652,0.478	0.364
6-8m_vs_8-10w	SE	Noc2l	ENSMUSG00000095567.7	chr4	+	156244039	156244141	156243509	156243623	156245429	156245543	28431	17,11	0,7	28,14	0,0	250	149	0.000821718527053	0.037754380851	1.0,0.484	1.0,1.0	-0.258
6-8m_vs_8-10w	SE	Noc2l	ENSMUSG00000095567.7	chr4	+	156245166	156245310	156243509	156243623	156245429	156245543	28432	69,27	0,7	45,47	0,0	292	149	0.00107015878887	0.0452822393457	1.0,0.663	1.0,1.0	-0.168
6-8m_vs_8-10w	SE	Jam3	ENSMUSG00000031990.15	chr9	-	27100082	27100182	27098711	27098766	27101237	27101440	28543	34,7	1,0	16,19	4,2	248	149	0.00108689172134	0.0458564851173	0.953,1.0	0.706,0.851	0.198
6-8m_vs_8-10w	SE	Bnc2	ENSMUSG00000028487.18	chr4	-	84506527	84506611	84414245	84414348	84546204	84546405	28609	2,1	1,0	0,0	1,2	232	149	0.000142886660893	0.012899951948	0.562,1.0	0.0,0.0	0.781
6-8m_vs_8-10w	SE	Calu	ENSMUSG00000029767.16	chr6	+	29374398	29374536	29372552	29372670	29376718	29377110	28738	94,71	4,7	59,73	1,0	286	149	0.000396554715352	0.0243513407997	0.924,0.841	0.968,1.0	-0.101
6-8m_vs_8-10w	SE	Tex264	ENSMUSG00000040813.16	chr9	-	106662359	106662528	106658745	106659307	106673516	106673738	28791	76,32	0,0	46,27	6,1	298	149	0.000132495358129	0.0121324319182	1.0,1.0	0.793,0.931	0.138
6-8m_vs_8-10w	SE	Nudt6	ENSMUSG00000050174.15	chr3	-	37409507	37409562	37404985	37405491	37416700	37416904	28797	1,0	3,4	6,6	4,4	203	149	0.000100107180248	0.0100470068776	0.197,0.0	0.524,0.524	-0.426
6-8m_vs_8-10w	SE	Nmu	ENSMUSG00000029236.4	chr5	-	76344063	76344093	76343921	76343972	76345438	76345498	28881	37,27	4,5	22,11	0,0	178	149	0.000230182887532	0.0174860313633	0.886,0.819	1.0,1.0	-0.147
6-8m_vs_8-10w	SE	Napepld	ENSMUSG00000044968.16	chr5	-	21683155	21683465	21675454	21676101	21700974	21701394	28884	21,10	0,0	15,16	2,4	298	149	5.20156481213e-05	0.00647494013591	1.0,1.0	0.789,0.667	0.272
6-8m_vs_8-10w	SE	RP24-168G14.1	ENSMUSG00000071691.11	chr19	-	4637217	4637316	4625840	4626190	4642112	4642223	28892	38,20	0,0	21,22	6,0	247	149	0.000914289082378	0.040766793916	1.0,1.0	0.679,1.0	0.16
6-8m_vs_8-10w	SE	Rpl3l	ENSMUSG00000002500.15	chr17	+	24735416	24735512	24733916	24734018	24735999	24736143	28904	21,19	3,3	21,12	0,0	244	149	0.000641794391827	0.0321558013852	0.81,0.795	1.0,1.0	-0.198
6-8m_vs_8-10w	SE	Rpl3l	ENSMUSG00000002500.15	chr17	+	24735721	24735841	24733916	24734018	24735999	24736143	28905	25,21	3,3	15,14	0,0	268	149	0.000859491333119	0.0389666571823	0.822,0.796	1.0,1.0	-0.191
6-8m_vs_8-10w	SE	Qpctl	ENSMUSG00000030407.2	chr7	-	19144883	19145036	19144659	19144759	19146869	19147151	29026	10,7	0,0	5,3	2,2	298	149	0.00015419047785	0.0135300037886	1.0,1.0	0.556,0.429	0.507
6-8m_vs_8-10w	SE	Ndufv1	ENSMUSG00000037916.13	chr19	-	4009343	4009533	4008593	4008806	4009825	4010009	29090	29,13	0,0	22,7	5,1	298	149	5.97183839678e-05	0.00705116036585	1.0,1.0	0.688,0.778	0.267
6-8m_vs_8-10w	SE	Gufl	ENSMUSG00000029208.16	chr5	+	69563082	69563222	69561653	69561857	69564523	69564647	29193	5,7	4,2	3,1	0,0	288	149	3.34991492912e-06	0.000821266166898	0.393,0.644	1.0,1.0	-0.482
6-8m_vs_8-10w	SE	Catsperd	ENSMUSG00000040828.9	chr17	+	56641476	56641590	56636270	56636335	56647727	56647811	29307	20,4	2,3	8,3	0,0	262	149	0.000354809454732	0.0228816474863	0.85,0.431	1.0,1.0	-0.36

6-8m_vs_8-10w	SE	Catsperd	ENSMUSG00000040828.9	chr17	+	56659528	56659581	56655988	56656047	56660170	56660245	29312	0,0	15,2	6,0	13,4	201	149	0.000736777592649	0.0351595084183	0.0,0.0	0.255,0.0	-0.128
6-8m_vs_8-10w	SE	RP23-395N7.8	ENSMUSG00000078500.3	chr4	+	145896643	145896770	145892412	145892516	145898928	145899975	29432	3,1	0,1	0,0	1,1	275	149	2.52700220522e-05	0.00373866644049	1.0,0.351	0.0,0.0	0.676
6-8m_vs_8-10w	SE	Efemp1	ENSMUSG00000020467.15	chr11	+	28916849	28916969	28915660	28915780	28921402	28921526	29434	2,1	0,0	0,2	2,12	268	149	1.2898969115e-08	9.63403004878e-06	1.0,1.0	0.0,0.085	0.958
6-8m_vs_8-10w	SE	Taz	ENSMUSG00000009995.17	chrX	+	74289104	74289182	74288447	74288513	74289298	74289348	29450	32,11	10,4	21,14	0,0	226	149	8.41882217495e-07	0.000307248742012	0.678,0.645	1.0,1.0	-0.339
6-8m_vs_8-10w	SE	Arl16	ENSMUSG00000057594.12	chr11	-	120465983	120466099	120465437	120465821	120466653	120466732	29596	54,32	3,7	31,35	0,0	264	149	5.94378765197e-05	0.00704393668748	0.91,0.721	1.0,1.0	-0.185
6-8m_vs_8-10w	SE	Armc12	ENSMUSG00000024223.2	chr17	+	28537552	28537726	28532342	28532477	28537880	28537952	29718	9,1	4,3	12,6	0,0	298	149	1.63836009537e-07	8.09501120352e-05	0.529,0.143	1.0,1.0	-0.664
6-8m_vs_8-10w	SE	Elovl3	ENSMUSG00000038754.4	chr19	+	46134000	46134152	46133123	46133255	46134374	46135307	29721	2,2	3,1	4,1	0,0	298	149	1.63820002911e-05	0.00280931813528	0.25,0.5	1.0,1.0	-0.625
6-8m_vs_8-10w	SE	Cldn6	ENSMUSG00000023906.2	chr17	+	23679757	23679821	23679364	23679407	23681047	23682446	29722	22,1	11,2	4,5	0,0	212	149	4.69333028796e-06	0.00108439565128	0.584,0.26	1.0,1.0	-0.578
6-8m_vs_8-10w	SE	Armc9	ENSMUSG00000062590.13	chr1	+	86218847	86218898	86213049	86213105	86244565	86244670	29737	1,0	1,7	1,3	1,0	199	149	1.67509053811e-05	0.00283143198537	0.428,0.0	0.428,1.0	-0.5
6-8m_vs_8-10w	SE	Mlkl	ENSMUSG00000012519.14	chr8	-	111316362	111316514	111314940	111315105	111319377	111319453	29849	7,6	3,6	0,1	1,3	298	149	0.000733915215765	0.0350750313534	0.538,0.333	0.0,0.143	0.364
6-8m_vs_8-10w	SE	Clcc1	ENSMUSG00000027884.16	chr3	+	108667962	108668184	108661931	108662033	108670901	108670995	29864	3,1	2,1	4,2	0,0	298	149	0.000413294804142	0.0249969414874	0.429,0.333	1.0,1.0	-0.619
6-8m_vs_8-10w	SE	RP23-435G20.2	ENSMUSG00000086447.2	chr2	+	52386413	52386550	52384581	52384751	52390111	52390298	29888	2,1	4,3	4,1	0,0	285	149	1.02995313722e-07	5.51299582583e-05	0.207,0.148	1.0,1.0	-0.823
6-8m_vs_8-10w	SE	Tnfrsf13b	ENSMUSG00000010142.12	chr11	+	61146840	61147017	61141260	61141491	61147415	61147642	29899	22,10	1,0	12,8	5,1	298	149	0.000489097152426	0.0274612659918	0.917,1.0	0.545,0.8	0.286
6-8m_vs_8-10w	SE	Cnot2	ENSMUSG00000020166.15	chr10	-	116537220	116537278	116507040	116507188	116549032	116549175	30007	0,0	15,2	2,4	2,1	206	149	1.39215315065e-06	0.000422127906596	0.0,0.0	0.42,0.743	-0.582
6-8m_vs_8-10w	SE	Cnot2	ENSMUSG00000020166.15	chr10	-	116537220	116537278	116517316	116517383	116549032	116549175	30008	0,0	12,1	2,4	10,6	206	149	0.000233663898248	0.0176572935438	0.0,0.0	0.126,0.325	-0.226
6-8m_vs_8-10w	SE	Timm10	ENSMUSG00000027076.10	chr2	+	84827598	84827713	84826996	84827147	84829789	84830113	30140	666,444	22,21	673,319	5,6	263	149	5.50357694514e-05	0.0066501391096	0.945,0.923	0.987,0.968	-0.044
6-8m_vs_8-10w	SE	Atf7ip2	ENSMUSG00000039200.16	chr16	+	10209133	10209268	10204603	10204722	10211004	10211103	30375	2879,2703	10,24	2701,2445	42,36	283	149	3.300110324e-05	0.0046690019015 7	0.993,0.983	0.971,0.973	0.016
6-8m_vs_8-10w	SE	Ifi35	ENSMUSG00000010358.13	chr11	+	101457415	101457522	101456565	101456664	101458200	101458698	30595	7,6	0,0	4,4	3,1	255	149	0.000408597286865	0.0248062579678	1.0,1.0	0.438,0.7	0.431
6-8m_vs_8-10w	SE	Ifi35	ENSMUSG00000010358.13	chr11	+	101457619	101457806	101457415	101457522	101458200	101458698	30596	268,143	4,7	257,121	0,0	298	149	0.0011891025269	0.048483765083	0.971,0.911	1.0,1.0	-0.059

6-8m_vs_8-10w	SE	Dus2	ENSMUSG00000031901.12	chr8	+	106053005	106053079	106051801	106051889	106053291	106053819	30637	102,41	5,8	54,36	0,0	222	149	5.28706686138e-05	0.0065559629081	0.932,0.775	1.0,1.0	-0.146
6-8m_vs_8-10w	SE	Primpol	ENSMUSG00000038225.15	chr8	-	46581567	46581650	46581437	46581484	46586342	46586451	30720	92,39	0,0	72,49	2,7	231	149	0.000211380775066	0.0166798647961	1.0,1.0	0.959,0.819	0.111
6-8m_vs_8-10w	SE	Primpol	ENSMUSG00000038225.15	chr8	-	46599718	46599866	46592592	46593703	46605084	46605214	30728	64,30	0,1	35,13	7,1	296	149	7.78814246004e-05	0.0085658898372	1.0,0.938	0.716,0.867	0.178
6-8m_vs_8-10w	SE	Tmem175	ENSMUSG00000013495.15	chr5	+	108641821	108641873	108639471	108639569	108642116	108642152	30751	56,24	0,0	16,21	6,0	200	149	0.0010645273832	0.0451591027273	1.0,1.0	0.665,1.0	0.167
6-8m_vs_8-10w	SE	RP23-266O23.1	ENSMUSG00000038925.13	chr14	+	24296008	24296138	24293283	24293307	24297981	24298052	30770	7,2	0,0	6,3	2,2	278	149	0.0003350485081	0.0219153113823	1.0,1.0	0.617,0.446	0.469
6-8m_vs_8-10w	SE	AC121576.1	ENSMUSG00000063087.14	chr18	-	5583712	5583796	5583011	5583133	5592159	5592190	30815	4,2	12,3	0,0	2,1	232	149	0.00054445085326	0.0291912914895	0.176,0.3	0.0,0.0	0.238
6-8m_vs_8-10w	SE	RP23-255F14.8	ENSMUSG00000111149.1	chr10	-	34231318	34231403	34230906	34231074	34233406	34233531	30837	30,32	7,3	12,21	1,0	233	149	0.0012026979439	0.0485990738756	0.733,0.872	0.885,1.0	-0.14
6-8m_vs_8-10w	SE	Eef1d	ENSMUSG00000055762.16	chr15	-	75896639	75896740	75896242	75896464	75900690	75900762	30964	152,118	13,7	220,99	2,2	249	149	6.44214893302e-05	0.0073628489371	0.875,0.91	0.985,0.967	-0.083
6-8m_vs_8-10w	SE	Eef1d	ENSMUSG00000055762.16	chr15	-	75900690	75900762	75896675	75896919	75901131	75901252	30969	136,104	41,28	158,67	84,44	220	149	0.00050533458548	0.0279334347028	0.692,0.716	0.56,0.508	0.17
6-8m_vs_8-10w	SE	Clhc1	ENSMUSG00000020461.10	chr11	+	29556068	29556256	29553503	29553762	29560449	29560651	31102	26,14	0,0	24,15	1,4	298	149	0.0012030222858	0.0485990738756	1.0,1.0	0.923,0.652	0.212
6-8m_vs_8-10w	SE	Clhc1	ENSMUSG00000020461.10	chr11	+	29557652	29557786	29553503	29553762	29560449	29560651	31103	39,11	0,0	20,7	1,4	282	149	0.00059502501941	0.0305757176374	1.0,1.0	0.914,0.48	0.303
6-8m_vs_8-10w	SE	Clhc1	ENSMUSG00000020461.10	chr11	+	29563849	29563956	29561371	29561456	29571649	29571852	31108	22,22	11,14	21,6	44,28	255	149	7.06078128343e-06	0.0014918687611	0.539,0.479	0.218,0.111	0.345
6-8m_vs_8-10w	SE	Clhc1	ENSMUSG00000020461.10	chr11	+	29569200	29569375	29561371	29561456	29571649	29571852	31109	114,79	11,14	118,80	44,28	298	149	0.00015243964585	0.0135241758734	0.838,0.738	0.573,0.588	0.208
6-8m_vs_8-10w	SE	Clhc1	ENSMUSG00000020461.10	chr11	+	29575344	29575524	29571649	29571852	29578037	29578204	31119	114,101	1,6	144,106	17,11	298	149	0.0010417815196	0.0447296088621	0.983,0.894	0.809,0.828	0.12
6-8m_vs_8-10w	SE	Yif1a	ENSMUSG00000024875.4	chr19	+	5090003	5090082	5089771	5089876	5092301	5092879	31126	94,51	6,3	107,54	0,0	227	149	0.00044398061243	0.0260705962205	0.911,0.918	1.0,1.0	-0.085
6-8m_vs_8-10w	SE	Yif1a	ENSMUSG00000024875.4	chr19	+	5091367	5091425	5089771	5089876	5092301	5092879	31128	12,3	6,3	6,1	0,0	206	149	2.57269318094e-06	0.00068284805123	0.591,0.42	1.0,1.0	-0.495
6-8m_vs_8-10w	SE	Yif1a	ENSMUSG00000024875.4	chr19	+	5091540	5091696	5089771	5089876	5092301	5092879	31131	6,10	6,3	3,3	0,0	298	149	7.62866648185e-07	0.00028488634038	0.333,0.625	1.0,1.0	-0.521
6-8m_vs_8-10w	SE	Alkbh7	ENSMUSG00000002661.14	chr17	+	56998670	56998795	56997338	56997567	56998929	56999311	31146	64,64	6,5	46,29	0,0	273	149	8.79935512714e-05	0.0091753275734	0.853,0.875	1.0,1.0	-0.136
6-8m_vs_8-10w	SE	Grina	ENSMUSG00000022564.7	chr15	+	76247639	76247737	76246769	76246900	76247850	76248177	31184	2,1	1,0	0,2	2,2	246	149	0.00092288896872	0.0409385388391	0.548,1.0	0.0,0.377	0.586

6-8m_vs_8-10w	SE	Ssc4d	ENSMUSG00000029699.13	chr5	-	135962922	135963234	135961888	135961966	135963355	135963430	31204	1,0	6,5	6,1	3,2	298	149	0.000392110117272	0.0242537005291	0.077,0.0	0.5,0.2	-0.312
6-8m_vs_8-10w	SE	Cyb5a	ENSMUSG00000024646.13	chr18	+	84873139	84873169	84851337	84851607	84877826	84877861	31269	51,32	3,6	28,18	0,0	178	149	0.000446092365217	0.0260705962205	0.934,0.817	1.0,1.0	-0.125
6-8m_vs_8-10w	SE	Tpd52	ENSMUSG00000027506.15	chr3	-	8947480	8947629	8934991	8935040	8963846	8964046	31393	4,1	4,2	0,0	5,1	297	149	0.000796820200133	0.0369273846284	0.334,0.201	0.0,0.0	0.268
6-8m_vs_8-10w	SE	Wdr18	ENSMUSG00000035754.8	chr10	+	79966559	79966726	79961001	79961112	79967319	79967388	31613	50,27	0,0	50,41	2,6	298	149	0.000190418401773	0.0158843568606	1.0,1.0	0.926,0.774	0.15
6-8m_vs_8-10w	SE	Map4k5	ENSMUSG00000034761.15	chr12	-	69820939	69820942	69818383	69818498	69822802	69822863	31637	140,85	0,2	81,98	6,6	151	149	0.000757947309245	0.0359030026308	1.0,0.977	0.93,0.942	0.052
6-8m_vs_8-10w	SE	Btbd19	ENSMUSG00000073771.11	chr4	-	117120970	117121096	117120174	117120861	117121197	117121329	31642	12,1	10,7	0,0	5,4	274	149	9.39653824641e-05	0.0095723350821	0.395,0.072	0.0,0.0	0.234
6-8m_vs_8-10w	SE	Btbd19	ENSMUSG00000073771.11	chr4	-	117121413	117121482	117120650	117120861	117122218	117122278	31644	21,6	5,4	10,12	0,1	217	149	0.000384421220376	0.0240195951626	0.743,0.507	1.0,0.892	-0.321
6-8m_vs_8-10w	SE	Ppp6r2	ENSMUSG00000036561.8	chr15	+	89256662	89256849	89246257	89246340	89259065	89259203	31753	24,27	0,0	26,11	6,0	298	149	0.000403612848552	0.0246903433221	1.0,1.0	0.684,1.0	0.158
6-8m_vs_8-10w	SE	Atrn	ENSMUSG00000027312.14	chr2	+	130947558	130947727	130935523	130935637	130957020	130957111	31916	25,14	0,0	8,14	0,6	298	149	0.000548900125584	0.0293807940554	1.0,1.0	1.0,0.538	0.231
6-8m_vs_8-10w	SE	Farsa	ENSMUSG00000003808.18	chr8	+	84868008	84868086	84864436	84864552	84868223	84868338	31948	12,10	1,4	9,8	0,0	226	149	0.000573171310752	0.0298832226738	0.888,0.622	1.0,1.0	-0.245
6-8m_vs_8-10w	SE	Tma7	ENSMUSG00000091537.2	chr9	-	109082170	109082226	109082001	109082089	109082326	109082587	32103	731,944	137,151	899,645	65,67	204	149	2.2780711626e-06	0.000620021469984	0.796,0.82	0.91,0.875	-0.085
6-8m_vs_8-10w	SE	Ddx43	ENSMUSG00000070291.4	chr9	+	78412192	78412311	78411591	78411748	78412965	78413076	32136	49,22	7,4	16,19	11,13	267	149	0.000205215444808	0.016406444924	0.796,0.754	0.448,0.449	0.327
6-8m_vs_8-10w	SE	Zfp951	ENSMUSG00000072774.10	chr5	-	104816996	104817123	104813168	104815435	104859666	104860070	32230	15,10	3,2	5,7	0,0	275	149	0.000781352131602	0.036473699213	0.73,0.73	1.0,1.0	-0.27
6-8m_vs_8-10w	SE	Tti2	ENSMUSG00000031577.10	chr8	+	31155777	31155921	31155513	31155701	31158592	31158755	32267	16,6	0,0	17,17	6,0	292	149	0.000819906976067	0.0377251181137	1.0,1.0	0.591,1.0	0.205
6-8m_vs_8-10w	SE	Tlk2	ENSMUSG00000020694.16	chr11	+	105269625	105269795	105260260	105260352	105275836	105275948	32494	6,12	0,0	16,19	0,6	298	149	0.000716973888235	0.034504011075	1.0,1.0	1.0,0.613	0.194
6-8m_vs_8-10w	SE	Slc26a8	ENSMUSG00000036196.15	chr17	-	28648120	28648213	28647896	28648028	28649281	28649351	32501	2,0	2,1	5,1	0,1	241	149	0.000395508551165	0.0243336257265	0.382,0.0	1.0,0.382	-0.5
6-8m_vs_8-10w	SE	Cog7	ENSMUSG00000034951.10	chr7	-	121949744	121949899	121930529	121930613	121951162	121951290	32647	4,2	0,0	1,0	0,2	298	149	0.0011956384344	0.048483765083	1.0,1.0	1.0,0.0	0.5
6-8m_vs_8-10w	SE	RP23-409B11.1	ENSMUSG00000097393.8	chr8	+	109229148	109229285	109223133	109223179	109249856	109249923	32714	12,5	7,0	2,0	4,6	285	149	0.000212603498431	0.0167352302834	0.473,1.0	0.207,0.0	0.633
6-8m_vs_8-10w	SE	Hsf1	ENSMUSG00000022556.11	chr15	+	76496835	76496960	76496452	76496589	76497618	76497694	32717	110,18	0,0	25,21	1,6	273	149	4.35783974329e-05	0.00573591726211	1.0,1.0	0.932,0.656	0.206

6-8m_vs_8-10w	SE	Senp5	ENSMUSG00000022772.11	chr16	-	31988939	31990465	31983767	31983873	32002991	32003178	32840	41,54	1,0	47,36	4,4	298	149	0.00065004210694	0.0323169540348	0.953,1.0	0.855,0.818	0.14
6-8m_vs_8-10w	SE	Gtf2f2	ENSMUSG00000067995.4	chr14	-	75995420	75995565	75906782	75906926	76010679	76010865	32870	11,2	1,0	7,0	3,1	293	149	0.00100807954063	0.0436470183008	0.848,1.0	0.543,0.0	0.652
6-8m_vs_8-10w	SE	Sapcd1	ENSMUSG00000036185.9	chr17	-	35026337	35026457	35025958	35026147	35026693	35026786	32953	0,0	3,3	2,6	9,5	268	149	0.000202420702201	0.0162523581797	0.0,0.0	0.11,0.4	-0.255
6-8m_vs_8-10w	SE	Phkg1	ENSMUSG00000025537.12	chr5	-	129869460	129869526	129866861	129867025	129873668	129873847	32973	29,39	16,11	46,34	5,3	214	149	0.000964658536826	0.042375015323	0.558,0.712	0.865,0.888	-0.242
6-8m_vs_8-10w	SE	Arfrp1	ENSMUSG00000038671.15	chr2	-	181361000	181361472	181359427	181359561	181364013	181364096	33024	125,57	1,2	80,68	6,8	298	149	0.000553068591795	0.0294484783889	0.984,0.934	0.87,0.81	0.119
6-8m_vs_8-10w	SE	Arfrp1	ENSMUSG00000038671.15	chr2	-	181361390	181361472	181357689	181359561	181364013	181364096	33030	143,72	1,2	92,91	6,8	230	149	0.000714907723453	0.034504011075	0.989,0.959	0.909,0.881	0.079
6-8m_vs_8-10w	SE	Arfrp1	ENSMUSG00000038671.15	chr2	-	181364604	181364698	181364310	181364398	181365218	181365338	33059	101,66	33,22	110,89	16,12	242	149	0.00120625716919	0.0486685367408	0.653,0.649	0.809,0.82	-0.163
6-8m_vs_8-10w	SE	Arfrp1	ENSMUSG00000038671.15	chr2	-	181364604	181364711	181364013	181364398	181365218	181365267	33063	148,93	33,22	169,135	16,12	255	149	0.000400495997538	0.0245464302613	0.724,0.712	0.861,0.868	-0.147
6-8m_vs_8-10w	SE	Tbc1d23	ENSMUSG00000022749.7	chr16	-	57177931	57178020	57173023	57173159	57182850	57182862	33066	126,72	10,3	74,78	16,16	237	149	0.000418654184051	0.0250756154424	0.888,0.938	0.744,0.754	0.164
6-8m_vs_8-10w	SE	Lgals9	ENSMUSG00000001123.15	chr11	-	78969713	78969803	78967997	78968033	78971316	78971427	33131	0,0	2,1	1,1	0,0	238	149	7.73263719722e-09	6.89837156183e-06	0.0,0.0	1.0,1.0	-1.0
6-8m_vs_8-10w	SE	Ccdc178	ENSMUSG00000024306.12	chr18	-	21844888	21845023	21810896	21811580	21914946	21915096	33133	0,1	1,1	1,10	0,0	283	149	0.000318701807777	0.02127947455	0.0,0.345	1.0,1.0	-0.828
6-8m_vs_8-10w	SE	Pard3	ENSMUSG00000025812.17	chr8	+	127410731	127410773	127409555	127409707	127415569	127415797	33194	48,30	0,0	60,50	1,8	190	149	0.000661045433755	0.0326617463853	1.0,1.0	0.979,0.831	0.095
6-8m_vs_8-10w	SE	Plekha4	ENSMUSG00000040428.19	chr7	+	45549803	45549965	45549229	45549306	45553244	45553294	33281	1,5	0,0	0,1	1,1	298	149	0.000271237360505	0.0194260017558	1.0,1.0	0.0,0.333	0.834
6-8m_vs_8-10w	SE	Gclc	ENSMUSG00000032350.9	chr9	+	77792052	77792173	77791879	77791983	77793039	77794485	33297	32,34	5,2	35,38	0,1	269	149	0.00116427274694	0.0481232735402	0.78,0.904	1.0,0.955	-0.135
6-8m_vs_8-10w	SE	Ap2b1	ENSMUSG00000035152.14	chr11	+	83364794	83364836	83350978	83351171	83365646	83365793	33466	0,0	7,5	2,2	3,2	190	149	0.0010084097515	0.0436470183008	0.0,0.0	0.343,0.44	-0.392
6-8m_vs_8-10w	SE	Zfp120	ENSMUSG00000068134.13	chr2	-	150119480	150119628	150114405	150118200	150119824	150119951	33467	65,31	1,0	41,28	3,4	296	149	0.000773537372988	0.0362670456509	0.97,1.0	0.873,0.779	0.159
6-8m_vs_8-10w	SE	Srpk2	ENSMUSG00000062604.11	chr5	-	23513951	23514044	23503263	23507720	23514633	23514703	33477	172,155	3,0	139,101	10,4	241	149	0.000266532188705	0.0193226812019	0.973,1.0	0.896,0.94	0.069
6-8m_vs_8-10w	SE	Mtg2	ENSMUSG00000039069.19	chr2	+	180073139	180073233	180071793	180071886	180078618	180078823	33503	1,0	2,1	1,2	0,0	242	149	2.66908405988e-06	0.000702625439894	0.235,0.0	1.0,1.0	-0.883
6-8m_vs_8-10w	SE	Mtg2	ENSMUSG00000039069.19	chr2	+	180084450	180084589	180083344	180083460	180085379	180085895	33513	4,2	1,0	1,0	1,3	287	149	3.41482466892e-06	0.000830837189902	0.675,1.0	0.342,0.0	0.667

6-8m_vs_8-10w	SE	Xlr3a	ENSMUSG00000057836.12	chrX	-	73094182	73094252	73090858	73093304	73096980	73097019	33617	149,116	6,6	97,95	0,0	218	149	0.000562507133256	0.0296641692802	0.944,0.93	1.0,1.0	-0.063
6-8m_vs_8-10w	SE	Xlr3a	ENSMUSG00000057836.12	chrX	-	73094960	73095082	73093269	73093304	73096980	73097095	33619	42,36	6,6	26,35	0,0	270	149	6.75690892926e-06	0.00143711845809	0.794,0.768	1.0,1.0	-0.219
6-8m_vs_8-10w	SE	Zfp715	ENSMUSG00000012640.16	chr7	-	43298006	43298139	43296196	43296448	43301169	43301265	33662	2,6	7,2	1,1	0,0	281	149	2.70646616118e-07	0.000125972271351	0.132,0.614	1.0,1.0	-0.627
6-8m_vs_8-10w	SE	Map3k3	ENSMUSG00000020700.11	chr11	+	106151008	106151140	106150269	106150418	106151617	106151747	33665	2,2	0,0	2,0	2,1	280	149	8.36831493978e-05	0.00892879742877	1.0,1.0	0.347,0.0	0.827
6-8m_vs_8-10w	SE	Arhgef39	ENSMUSG00000051517.14	chr4	-	43497086	43497710	43496523	43496720	43498585	43498704	33700	0,1	4,0	2,2	0,1	298	149	0.000564185015868	0.0297038786387	0.0,1.0	1.0,0.5	-0.25
6-8m_vs_8-10w	SE	Use1	ENSMUSG00000002395.14	chr8	+	71368849	71368877	71368549	71368562	71369526	71369729	33723	6,0	2,2	5,4	0,0	176	149	0.000410686792948	0.0248860698912	0.717,0.0	1.0,1.0	-0.642
6-8m_vs_8-10w	SE	Rnmt	ENSMUSG00000009535.13	chr18	+	68313987	68314152	68313664	68313846	68317993	68318111	33780	521,349	0,2	427,274	14,4	298	149	6.62383895258e-05	0.00751700395057	1.0,0.989	0.938,0.972	0.039
6-8m_vs_8-10w	SE	Sec11a	ENSMUSG00000025724.12	chr7	-	80927736	80927886	80920894	80923220	80947401	80947511	33814	290,245	1,4	272,279	15,5	298	149	0.00118019631918	0.048483765083	0.993,0.968	0.901,0.965	0.047
6-8m_vs_8-10w	SE	Sec11a	ENSMUSG00000025724.12	chr7	-	80935026	80935136	80920894	80923220	80947401	80947511	33816	112,105	1,4	92,103	15,5	258	149	0.000860569012955	0.0389666571823	0.985,0.938	0.78,0.922	0.111
6-8m_vs_8-10w	SE	Fam135a	ENSMUSG00000026153.15	chr1	-	24098630	24098719	24086519	24086611	24100060	24100182	33833	5,7	30,25	0,1	22,14	237	149	0.000583567471885	0.0301801174349	0.095,0.15	0.0,0.043	0.101
6-8m_vs_8-10w	SE	Mia3	ENSMUSG00000056050.12	chr1	-	183329922	183329991	183328203	183328345	183331091	183331192	33864	7,0	2,2	3,2	0,0	217	149	0.000236258118004	0.0178114218728	0.706,0.0	1.0,1.0	-0.647
6-8m_vs_8-10w	SE	Scrn3	ENSMUSG00000008226.14	chr2	+	73319302	73319499	73315436	73315592	73321984	73322022	33911	16,16	7,2	19,5	0,0	298	149	8.66768539776e-06	0.00173309714853	0.533,0.8	1.0,1.0	-0.333
6-8m_vs_8-10w	SE	Hcn3	ENSMUSG00000028051.10	chr3	-	89149815	89150056	89148716	89148881	89150354	89150501	34025	1,2	0,0	0,0	1,2	298	149	3.92319521314e-09	4.34473577466e-06	1.0,1.0	0.0,0.0	1.0
6-8m_vs_8-10w	SE	Park7	ENSMUSG00000028964.14	chr4	-	150903814	150903884	150901007	150901094	150905249	150905309	34045	420,305	15,19	333,232	3,2	218	149	1.08521294785e-05	0.00207538698836	0.95,0.916	0.987,0.988	-0.054
6-8m_vs_8-10w	SE	Dock10	ENSMUSG00000038608.15	chr1	-	80503918	80503994	80501187	80501826	80505324	80505463	34132	5,1	1,0	1,1	2,3	224	149	7.97552700552e-05	0.00868278051896	0.769,1.0	0.25,0.181	0.669
6-8m_vs_8-10w	SE	Luc7l2	ENSMUSG00000029823.16	chr6	+	38568846	38568917	38551513	38551908	38570509	38570604	34145	3,1	0,0	1,0	1,2	219	149	4.63600904177e-06	0.00107891352453	1.0,1.0	0.405,0.0	0.798
6-8m_vs_8-10w	SE	Luc7l2	ENSMUSG00000029823.16	chr6	+	38571041	38571088	38570509	38570604	38585040	38585139	34148	1,6	9,12	1,0	20,15	195	149	0.000648827969276	0.0323066031958	0.078,0.276	0.037,0.0	0.159
6-8m_vs_8-10w	SE	Abca8b	ENSMUSG00000020620.14	chr11	-	109934722	109934778	109932189	109934611	109935715	109935795	34222	6,4	0,0	3,3	2,3	204	149	1.45294347769e-05	0.00256388641371	1.0,1.0	0.523,0.422	0.528
6-8m_vs_8-10w	SE	Golga1	ENSMUSG00000026754.16	chr2	-	39017738	39017914	39016778	39016942	39018338	39018406	34247	1,0	0,2	6,4	0,0	298	149	0.00119563899445	0.048483765083	1.0,0.0	1.0,1.0	-0.5

6-8m_vs_8-10w	SE	Golga1	ENSMUSG00000026754.16	chr2	-	39048309	39048438	39047022	39047766	39050158	39050191	34251	28,16	2,4	20,19	0,0	277	149	0.000173318082271	0.0148831110434	0.883,0.683	1.0,1.0	-0.217
6-8m_vs_8-10w	SE	Drg1	ENSMUSG00000020457.13	chr11	-	3252643	3252811	3252159	3252282	3259305	3259375	34398	414,293	2,3	368,313	22,11	298	149	5.42046795005e-07	0.000212297254492	0.99,0.98	0.893,0.934	0.072
6-8m_vs_8-10w	SE	Drg1	ENSMUSG00000020457.13	chr11	-	3256542	3256712	3252159	3252282	3259305	3259375	34403	243,149	2,3	207,184	22,11	298	149	1.1336293968e-06	0.00037533651245	0.984,0.961	0.825,0.893	0.113
6-8m_vs_8-10w	SE	Drg1	ENSMUSG00000020457.13	chr11	-	3256542	3256712	3254410	3254642	3259305	3259375	34405	548,348	2,5	453,407	13,14	298	149	0.00019000153442	0.0158843568606	0.993,0.972	0.946,0.936	0.041
6-8m_vs_8-10w	SE	Kmt2e	ENSMUSG00000029004.15	chr5	+	23446525	23446598	23434448	23434783	23450151	23450336	34408	1,3	1,4	5,1	1,0	221	149	0.000524652827789	0.028638998816	0.403,0.336	0.771,1.0	-0.516
6-8m_vs_8-10w	SE	Nagpa	ENSMUSG00000023143.10	chr16	-	5198730	5198936	5198575	5198623	5199615	5199744	34491	3,0	1,2	1,4	0,0	298	149	0.000297206687673	0.0204141055594	0.6,0.0	1.0,1.0	-0.7
6-8m_vs_8-10w	SE	AC122423.2	ENSMUSG00000030924.16	chr7	+	119819431	119819547	119805142	119805286	119827302	119827376	34541	7,2	0,0	1,0	2,2	264	149	9.81302350489e-09	8.08089904828e-06	1.0,1.0	0.22,0.0	0.89
6-8m_vs_8-10w	SE	AC122423.2	ENSMUSG00000030924.16	chr7	+	119825535	119825638	119824044	119824139	119827302	119827376	34548	70,31	2,1	34,30	4,8	251	149	0.000765830206639	0.0361165975278	0.954,0.948	0.835,0.69	0.189
6-8m_vs_8-10w	SE	AC122423.2	ENSMUSG00000030924.16	chr7	+	119844258	119844446	119843847	119844064	119848731	119848943	34556	2,3	6,8	5,5	0,0	298	149	2.56104493168e-09	3.04631551948e-06	0.143,0.158	1.0,1.0	-0.85
6-8m_vs_8-10w	SE	AC122423.2	ENSMUSG00000030924.16	chr7	+	119845382	119845501	119843864	119844064	119848731	119848939	34557	18,12	6,8	14,6	0,0	267	149	1.05933911243e-06	0.000365825106826	0.626,0.456	1.0,1.0	-0.459
6-8m_vs_8-10w	SE	Sgms1	ENSMUSG00000040451.17	chr19	-	32125225	32125392	32122726	32124524	32129540	32129694	34745	167,115	14,5	136,137	2,2	298	149	0.000575704928756	0.0298832226738	0.856,0.92	0.971,0.972	-0.084
6-8m_vs_8-10w	SE	Nasp	ENSMUSG00000028693.15	chr4	-	116610402	116611377	116605683	116605763	116611971	116612081	34767	407,232	331,247	171,175	523,314	298	149	2.13359330203e-11	2.75983081166e-07	0.381,0.32	0.141,0.218	0.171
6-8m_vs_8-10w	SE	Ddx5	ENSMUSG00000020719.14	chr11	-	106788272	106788459	106786908	106787074	106789015	106789185	34990	153,61	9,6	101,71	2,0	298	149	0.000338724976112	0.0220193627269	0.895,0.836	0.962,1.0	-0.116
6-8m_vs_8-10w	SE	Pramef12	ENSMUSG00000028591.11	chr4	-	144403337	144403450	144395679	144396002	144408356	144408417	35012	8,5	3,2	0,1	4,1	261	149	0.00012080673335	0.0113445293809	0.604,0.588	0.0,0.363	0.415
6-8m_vs_8-10w	SE	Slc11a1	ENSMUSG00000026177.11	chr1	+	74384696	74384770	74383599	74383744	74385392	74385755	35032	3,4	3,1	1,4	0,0	222	149	0.000526600585607	0.028638998816	0.402,0.729	1.0,1.0	-0.435
6-8m_vs_8-10w	SE	Tarbp2	ENSMUSG00000023051.11	chr15	+	102520474	102520577	102518454	102518602	102521137	102521213	35075	1,0	1,3	4,1	1,0	251	149	6.55150011308e-06	0.00141045249375	0.373,0.0	0.704,1.0	-0.666
6-8m_vs_8-10w	SE	Farsb	ENSMUSG00000026245.16	chr1	-	78479956	78480013	78472483	78472634	78482307	78482363	35150	4,2	2,2	6,3	0,0	205	149	0.000309684364222	0.0208945862213	0.592,0.421	1.0,1.0	-0.494
6-8m_vs_8-10w	SE	Rrh	ENSMUSG00000028012.15	chr3	-	129812607	129812761	129811610	129811779	129815559	129815750	35256	31,27	6,1	21,17	0,0	298	149	0.000267681516531	0.0193622963624	0.721,0.931	1.0,1.0	-0.174
6-8m_vs_8-10w	SE	Fam91a1	ENSMUSG00000037119.4	chr15	+	58442831	58442966	58441577	58441726	58443160	58443274	35334	16,2	7,0	15,1	0,0	283	149	0.000196574946361	0.0160023263425	0.546,1.0	1.0,1.0	-0.227

6-8m_vs_8-10w	SE	Nedd1	ENSMUSG00000019988.7	chr10	-	92700719	92700949	92698725	92698927	92711170	92711311	35368	253,186	1,2	116,91	8,5	298	149	2.90240334612e-05	0.0041613207975	0.992,0.979	0.879,0.901	0.096
6-8m_vs_8-10w	SE	Zfand2b	ENSMUSG00000026197.12	chr1	+	75170685	75170753	75170538	75170599	75170975	75171048	35390	15,5	0,0	6,4	2,2	216	149	0.000794518425069	0.0368739215889	1.0,1.0	0.674,0.58	0.373
6-8m_vs_8-10w	SE	Lta4h	ENSMUSG00000015889.8	chr10	+	93482249	93482332	93480690	93480786	93482961	93483001	35446	1,1	0,0	1,0	1,1	231	149	0.000602993949708	0.0307392915696	1.0,1.0	0.392,0.0	0.804
6-8m_vs_8-10w	SE	Gstm1	ENSMUSG00000058135.12	chr3	-	108016327	108016428	108014944	108015040	108016518	108016600	35503	55,47	9,5	27,12	1,0	249	149	0.000567999607106	0.029806985918	0.785,0.849	0.942,1.0	-0.154
6-8m_vs_8-10w	SE	Tmem258	ENSMUSG00000036372.14	chr19	+	10205749	10206124	10204013	10204264	10206423	10206533	35515	11,3	50,42	1,0	39,23	298	149	0.000571178212606	0.0298761554985	0.099,0.034	0.013,0.0	0.06
6-8m_vs_8-10w	SE	Atrx	ENSMUSG00000031229.16	chrX	-	105911469	105911654	105890916	105890972	105928487	105928577	35783	6,2	2,5	7,3	0,1	298	149	0.000627257082212	0.0316247856394	0.6,0.167	1.0,0.6	-0.417
6-8m_vs_8-10w	SE	Hells	ENSMUSG00000025001.11	chr19	+	38950187	38950384	38946724	38946868	38951834	38951931	35791	411,276	9,3	314,279	20,21	298	149	7.86226576444e-06	0.00162906146639	0.958,0.979	0.887,0.869	0.09
6-8m_vs_8-10w	SE	Rasa4	ENSMUSG00000004952.13	chr5	+	136101944	136102057	136101243	136101689	136102234	136102295	35837	2,2	0,0	0,0	1,1	261	149	7.67041463678e-09	6.89837156183e-06	1.0,1.0	0.0,0.0	1.0
6-8m_vs_8-10w	SE	D2Wsu81e	ENSMUSG00000039660.16	chr2	-	30174921	30175069	30173452	30174185	30175186	30175289	35893	50,19	3,4	37,33	0,0	296	149	0.000174679700909	0.0149191169122	0.893,0.705	1.0,1.0	-0.201
6-8m_vs_8-10w	SE	Erc1	ENSMUSG00000030172.15	chr6	-	119713679	119713811	119694570	119694731	119722172	119722308	35925	1,4	0,0	8,3	3,2	280	149	2.23149582964e-05	0.0034621603896	1.0,1.0	0.587,0.444	0.485
6-8m_vs_8-10w	SE	Zwilch	ENSMUSG00000032400.17	chr9	-	64149265	64149334	64147269	64147406	64149398	64149427	35945	1,2	144,69	7,7	84,69	217	149	0.000484644631956	0.0273067491226	0.005,0.02	0.054,0.065	-0.047
6-8m_vs_8-10w	SE	Zwilch	ENSMUSG00000032400.17	chr9	-	64149265	64149334	64147269	64147406	64150155	64150268	35946	1,2	2,3	7,9	0,0	217	149	9.64649670143e-07	0.00034255304760 2	0.256,0.314	1.0,1.0	-0.715
6-8m_vs_8-10w	SE	Rrnad1	ENSMUSG00000004896.16	chr3	-	87927562	87927755	87924343	87924528	87927923	87928048	35996	7,3	0,0	0,1	1,2	298	149	1.12296609867e-06	0.0003753365124 5	1.0,1.0	0.0,0.2	0.9
6-8m_vs_8-10w	SE	Ube2d-ps	ENSMUSG00000083844.8	chr11	+	5768885	5769072	5765508	5765571	5769166	5769243	36209	1,0	2,1	1,2	1,0	298	149	0.000560882837732	0.0296360686419	0.2,0.0	0.333,1.0	-0.567
6-8m_vs_8-10w	SE	Rnf141	ENSMUSG00000030788.16	chr7	-	110815938	110815984	110815582	110815790	110821253	110821361	36248	36,24	7,2	20,30	0,0	194	149	8.64390106724e-05	0.00908435046067	0.798,0.902	1.0,1.0	-0.15
6-8m_vs_8-10w	SE	Sel1l2	ENSMUSG00000074764.11	chr2	-	140295993	140296144	140293847	140294074	140304608	140304777	36280	36,17	0,0	19,20	7,3	298	149	1.57116834176e-06	0.000467218911703	1.0,1.0	0.576,0.769	0.328
6-8m_vs_8-10w	SE	RP23-211P15.1	ENSMUSG00000050812.18	chr4	-	58905003	58905107	58885367	58885498	58912475	58912704	36297	14,2	4,6	7,3	1,0	252	149	0.000500918148382	0.0278272816688	0.674,0.165	0.805,1.0	-0.483
6-8m_vs_8-10w	SE	Pou2f2	ENSMUSG00000008496.19	chr7	-	25093287	25093361	25092519	25093002	25093484	25093686	36419	11,0	2,4	6,2	1,0	222	149	0.000879774598886	0.0395726064675	0.787,0.0	0.801,1.0	-0.507
6-8m_vs_8-10w	SE	Ap3m2	ENSMUSG00000031539.6	chr8	-	22793065	22793151	22792286	22792851	22797081	22797219	36424	117,68	12,0	89,73	1,0	234	149	0.000532787213911	0.0288063874781	0.861,1.0	0.983,1.0	-0.061

6-8m_vs_8-10w	SE	Fbxo34	ENSMUSG00000037536.14	chr14	+	47509056	47509229	47465418	47465554	47526204	47526314	36586	2,5	9,5	0,0	11,2	298	149	0.000261751774576	0.0190190497563	0.1,0.333	0.0,0.0	0.217
6-8m_vs_8-10w	SE	Fbxo34	ENSMUSG00000037536.14	chr14	+	47509056	47509229	47507264	47507446	47526204	47526314	36588	6,15	13,13	3,1	18,10	298	149	0.0010213448164 3	0.0441473891312	0.188,0.366	0.077,0.048	0.215
6-8m_vs_8-10w	SE	Aldh7a1	ENSMUSG00000053644.13	chr18	-	56542252	56542322	56538700	56538742	56544964	56545042	36611	2,1	2,1	4,3	0,0	218	149	0.000185353807391	0.0156653233636	0.406,0.406	1.0,1.0	-0.594
6-8m_vs_8-10w	SE	Ranbp17	ENSMUSG00000040594.19	chr11	-	33337854	33337914	33329470	33329544	33404037	33404199	36692	2,1	0,0	1,0	8,2	208	149	4.74943195883e-10	1.39317900388e-06	1.0,1.0	0.082,0.0	0.959
6-8m_vs_8-10w	SE	Ranbp17	ENSMUSG00000040594.19	chr11	-	33404037	33404199	33329470	33329544	33441802	33441938	36694	1,2	24,8	13,4	10,5	298	149	1.15681710987e-05	0.0021726513626 1	0.02,0.111	0.394,0.286	-0.274
6-8m_vs_8-10w	SE	Ythdc2	ENSMUSG00000034653.11	chr18	+	44833021	44833218	44830151	44830242	44837340	44837507	36772	31,25	4,4	34,20	1,0	298	149	0.000550682036726	0.0294254639005	0.795,0.758	0.944,1.0	-0.196
6-8m_vs_8-10w	SE	Atad3a	ENSMUSG00000029036.18	chr4	-	155746028	155746137	155740640	155741352	155753881	155754047	36867	28,13	3,5	16,12	0,0	257	149	4.42823735051e-05	0.0058047865611 8	0.844,0.601	1.0,1.0	-0.278
6-8m_vs_8-10w	SE	Nubp2	ENSMUSG00000039183.5	chr17	-	24885418	24885617	24883805	24883875	24885718	24885837	36926	66,22	5,2	58,25	0,0	298	149	0.000647884109192	0.0323066031958	0.868,0.846	1.0,1.0	-0.143
6-8m_vs_8-10w	SE	Camkmt	ENSMUSG00000071037.5	chr17	+	85431470	85431545	85402425	85402492	85439526	85439590	37099	69,44	11,2	33,31	1,0	223	149	0.000460538120727	0.0264070591364	0.807,0.936	0.957,1.0	-0.107
6-8m_vs_8-10w	SE	Prkx	ENSMUSG00000035725.13	chrX	-	77773115	77773235	77771246	77771342	77782037	77782301	37249	24,18	0,0	11,10	2,3	268	149	0.000249861397085	0.0185324448702	1.0,1.0	0.754,0.65	0.298
6-8m_vs_8-10w	SE	Prkx	ENSMUSG00000035725.13	chrX	-	77782037	77782301	77771246	77771342	77786127	77786296	37251	18,6	5,0	2,6	0,0	298	149	0.0011896066683 6	0.048483765083	0.643,1.0	1.0,1.0	-0.179
6-8m_vs_8-10w	SE	Rhebl1	ENSMUSG00000023755.10	chr15	-	98879404	98879487	98879258	98879315	98879764	98879844	37361	177,86	7,5	122,96	0,1	231	149	0.000901016378497	0.0404713874291	0.942,0.917	1.0,0.984	-0.063
6-8m_vs_8-10w	SE	Atf2	ENSMUSG00000027104.18	chr2	-	73842359	73842446	73816508	73819148	73892474	73892530	37466	9,3	1,7	7,6	0,0	235	149	3.48655157852e-05	0.0048115829942 2	0.851,0.214	1.0,1.0	-0.468
6-8m_vs_8-10w	SE	Atf2	ENSMUSG00000027104.18	chr2	-	73863168	73863238	73816508	73819148	73880284	73880379	37487	6,3	4,8	2,7	1,0	218	149	0.000250692722026	0.0185512614299	0.506,0.204	0.578,1.0	-0.434
6-8m_vs_8-10w	SE	Atf2	ENSMUSG00000027104.18	chr2	-	73863168	73863238	73816508	73819148	73892474	73892530	37488	10,2	1,7	4,5	0,0	218	149	4.14412916748e-05	0.0054770721128 7	0.872,0.163	1.0,1.0	-0.483
6-8m_vs_8-10w	SE	Atf2	ENSMUSG00000027104.18	chr2	-	73873669	73873745	73816508	73819148	73892474	73892553	37505	7,13	1,7	9,2	0,0	224	149	1.83778741936e-05	0.0030113459571 5	0.823,0.553	1.0,1.0	-0.312
6-8m_vs_8-10w	SE	Atf2	ENSMUSG00000027104.18	chr2	-	73880284	73880379	73816508	73819148	73892474	73892614	37516	4,12	1,7	2,1	0,0	243	149	4.77405570631e-06	0.0010951683790 3	0.71,0.512	1.0,1.0	-0.389
6-8m_vs_8-10w	SE	Exosc5	ENSMUSG00000061286.7	chr7	+	25665420	25665561	25659163	25659383	25667699	25667803	37552	1,4	0,0	0,0	1,1	289	149	5.12538411712e-09	5.18981436546e-06	1.0,1.0	0.0,0.0	1.0
6-8m_vs_8-10w	SE	Eed	ENSMUSG00000030619.9	chr7	-	89971539	89971605	89970305	89970431	89972230	89972298	37783	384,307	7,1	253,213	7,14	214	149	0.000500476789718	0.0278272816688	0.974,0.995	0.962,0.914	0.046

6-8m_vs_8-10w	SE	Snrnp25	ENSMUSG00000040767.10	chr11	+	32208333	32208408	32207556	32207662	32208684	32208984	37851	1549,1473	126,94	1511,1223	53,62	223	149	0.0003864797055 7	0.024101324707	0.891,0.913	0.95,0.929	-0.037
6-8m_vs_8-10w	SE	Nup205	ENSMUSG00000038759.15	chr6	+	35189854	35190083	35189095	35189255	35191873	35191959	37996	27,4	0,0	8,7	2,2	298	149	0.000416757586059	0.0250651185671	1.0,1.0	0.667,0.636	0.349
6-8m_vs_8-10w	SE	Milr1	ENSMUSG00000040528.15	chr11	+	106761185	106761296	106754781	106755051	106763855	106763948	38083	4,2	2,1	1,0	3,1	259	149	0.000457945889144	0.0263802074626	0.535,0.535	0.161,0.0	0.455
6-8m_vs_8-10w	SE	Abca5	ENSMUSG00000018800.14	chr11	-	110313328	110313517	110311310	110311458	110314523	110314665	38134	8,3	1,3	7,3	0,0	298	149	0.000717040216461	0.034504011075	0.8,0.333	1.0,1.0	-0.434
6-8m_vs_8-10w	SE	Mad1l1	ENSMUSG00000029554.15	chr5	-	140105514	140105605	140088629	140088837	140117236	140117325	38148	11,7	0,0	6,10	1,4	239	149	0.00015397886975	0.0135300037886	1.0,1.0	0.789,0.609	0.301
6-8m_vs_8-10w	SE	Usp33	ENSMUSG00000025437.15	chr3	+	152389740	152389831	152384115	152384224	152391649	152391718	38181	140,87	4,2	92,74	9,12	239	149	0.000160128316137	0.0139368048809	0.956,0.964	0.864,0.794	0.131
6-8m_vs_8-10w	SE	Usp33	ENSMUSG00000025437.15	chr3	+	152389740	152389843	152384115	152384224	152391649	152391718	38182	324,229	4,2	255,208	9,12	251	149	0.000428172641368	0.025478680913	0.98,0.986	0.944,0.911	0.055
6-8m_vs_8-10w	SE	Ppih	ENSMUSG00000060288.13	chr4	-	119311531	119311572	119310758	119310847	119317027	119317120	38218	422,449	25,45	372,297	65,41	189	149	0.000315524244103	0.0211140293532	0.93,0.887	0.819,0.851	0.074
6-8m_vs_8-10w	SE	Sp100	ENSMUSG00000026222.16	chr1	+	85691974	85692049	85681085	85681139	85694258	85694309	38340	4,2	1,0	2,0	2,1	223	149	0.000230408219346	0.0174860313633	0.728,1.0	0.401,0.0	0.664
6-8m_vs_8-10w	SE	Mpv17l	ENSMUSG00000022679.13	chr16	+	13942119	13942173	13903160	13903280	13944665	13944736	38518	6,2	2,0	1,1	1,4	202	149	0.00090918882201	0.0406678387293	0.689,1.0	0.425,0.156	0.554
6-8m_vs_8-10w	SE	Ap1m1	ENSMUSG00000003033.15	chr8	+	72251742	72251869	72250680	72250828	72253208	72253280	38550	40,11	0,0	16,10	1,4	275	149	0.000502547744673	0.0278272816688	1.0,1.0	0.897,0.575	0.264
6-8m_vs_8-10w	SE	Ap1m1	ENSMUSG00000003033.15	chr8	+	72253208	72253280	72250680	72250828	72255696	72255822	38554	0,0	2,3	2,2	0,3	220	149	0.000920165451108	0.0408878363228	0.0,0.0	1.0,0.311	-0.656
6-8m_vs_8-10w	SE	RP24-358K11.2	ENSMUSG00000113093.1	chr12	+	17562850	17563102	17557698	17557908	17563525	17563684	38646	8,7	1,4	9,8	0,0	298	149	6.86133869984e-05	0.00773188609418	0.8,0.467	1.0,1.0	-0.366
6-8m_vs_8-10w	SE	RP23-276L18.1	ENSMUSG00000096401.2	chr8	+	20147548	20147716	20145519	20145639	20151480	20151665	38657	7,6	0,0	4,1	3,1	298	149	2.22489821498e-05	0.0034621603896	1.0,1.0	0.4,0.333	0.634
6-8m_vs_8-10w	SE	Foxn3	ENSMUSG00000033713.12	chr12	-	99219597	99219663	99209460	99209566	99291236	99291278	38791	2,5	33,20	11,12	16,17	214	149	0.000193192530944	0.015950054817	0.04,0.148	0.324,0.33	-0.233
6-8m_vs_8-10w	SE	Lyrm1	ENSMUSG00000030922.12	chr7	+	119909670	119909962	119895869	119895890	119914172	119914265	38901	104,123	5,5	115,115	0,0	298	149	0.000321764051539	0.0214393657245	0.912,0.925	1.0,1.0	-0.081
6-8m_vs_8-10w	SE	Lyrm1	ENSMUSG00000030922.12	chr7	+	119909803	119909962	119895860	119895890	119914172	119914265	38903	104,124	5,5	113,114	0,0	298	149	0.000339097849436	0.0220193627269	0.912,0.925	1.0,1.0	-0.081
6-8m_vs_8-10w	SE	Fam155a	ENSMUSG00000079157.4	chr8	-	9209500	9209546	9207626	9208032	9232933	9233105	38938	8,4	6,4	4,4	0,0	194	149	2.13784868353e-06	0.00059703607235	0.506,0.434	1.0,1.0	-0.53
6-8m_vs_8-10w	SE	Snhg17	ENSMUSG00000085385.7	chr2	-	158360463	158360599	158359238	158359432	158361404	158361475	38973	21,11	2,4	24,14	1,0	284	149	0.00105101312649	0.0448860871946	0.846,0.591	0.926,1.0	-0.245

6-8m_vs_8-10w	SE	Snhg17	ENSMUSG00000085385.7	chr2	-	158360526	158360599	158359181	158359432	158361404	158361519	38975	9,1	2,4	9,2	1,0	221	149	0.000798374717904	0.0369461130262	0.752,0.144	0.859,1.0	-0.482
6-8m_vs_8-10w	SE	Tdrd9	ENSMUSG00000054003.13	chr12	+	112063500	112063619	112051867	112052006	112067602	112067675	39012	32,40	7,2	40,13	1,0	267	149	0.00090863141497	0.0406678387293	0.718,0.918	0.957,1.0	-0.16
6-8m_vs_8-10w	SE	Unc45b	ENSMUSG00000018845.14	chr11	+	82928654	82928749	82925886	82926187	82929420	82929561	39086	1,1	3,1	3,3	0,0	243	149	1.32824944898e-06	0.000422127906596	0.17,0.38	1.0,1.0	-0.725
6-8m_vs_8-10w	SE	H2-K1	ENSMUSG00000061232.15	chr17	-	33996826	33996859	33996052	33996503	33997037	33997157	39158	7,2	10,5	5,7	1,1	181	149	0.000451415290622	0.0261690495914	0.366,0.248	0.805,0.852	-0.522
6-8m_vs_8-10w	SE	H2-K1	ENSMUSG00000061232.15	chr17	-	33996826	33996859	33996615	33996654	33997037	33997157	39159	58,39	5,10	27,43	2,0	181	149	0.000783354027209	0.0365140753815	0.905,0.762	0.917,1.0	-0.125
6-8m_vs_8-10w	SE	Usp20	ENSMUSG00000026854.16	chr2	+	30998772	30998868	30995755	30996147	31000056	31000078	39286	4,2	2,1	7,4	0,0	244	149	0.000976407964365	0.0427225043373	0.55,0.55	1.0,1.0	-0.45
6-8m_vs_8-10w	SE	Fmr1nb	ENSMUSG00000062170.12	chrX	+	68768955	68769093	68761933	68762231	68803604	68803693	39457	1,1	10,5	10,4	2,0	286	149	1.8346327435e-07	8.79418883437e-05	0.05,0.094	0.723,1.0	-0.789
6-8m_vs_8-10w	SE	Fmr1nb	ENSMUSG00000062170.12	chrX	+	68803604	68803693	68772044	68772141	68804425	68804567	39465	869,813	10,7	1092,771	37,26	237	149	4.34642587532e-06	0.00103399861787	0.982,0.986	0.949,0.949	0.035
6-8m_vs_8-10w	SE	Gltscr2	ENSMUSG00000041560.12	chr7	-	15943984	15944087	15942847	15942897	15945289	15945354	39508	0,1	57,5	4,6	17,14	251	149	4.76369806991e-05	0.00609525606427	0.0,0.106	0.123,0.203	-0.11
6-8m_vs_8-10w	SE	RP23-320I11.1	ENSMUSG00000114115.1	chr13	+	12561014	12561122	12541253	12541574	12562346	12562716	39525	20,11	4,3	10,20	1,0	256	149	0.000861449950163	0.0389666571823	0.744,0.681	0.853,1.0	-0.214
6-8m_vs_8-10w	SE	Gcdh	ENSMUSG00000003809.14	chr8	-	84889261	84889387	84888526	84888687	84892401	84892572	39590	22,15	4,2	13,1	0,0	274	149	0.000541641757247	0.0291472121345	0.749,0.803	1.0,1.0	-0.224
6-8m_vs_8-10w	SE	Gcdh	ENSMUSG00000003809.14	chr8	-	84892401	84892572	84891012	84891142	84892830	84892893	39600	16,7	4,2	3,3	0,0	298	149	0.000116684700092	0.0111200172942	0.667,0.636	1.0,1.0	-0.349
6-8m_vs_8-10w	SE	Pdcd2l	ENSMUSG00000002635.11	chr7	-	34192735	34193106	34189348	34189456	34196065	34196126	39625	448,303	8,10	369,249	29,15	298	149	0.000128074794085	0.0118196841576	0.966,0.938	0.864,0.892	0.074
6-8m_vs_8-10w	SE	Ccdc163	ENSMUSG00000028689.14	chr4	+	116711310	116711378	116710100	116710280	116712371	116712439	39715	10,4	8,0	2,5	0,0	216	149	0.000470413439435	0.0267868759236	0.463,1.0	1.0,1.0	-0.268
6-8m_vs_8-10w	SE	Ccdc163	ENSMUSG00000028689.14	chr4	+	116712722	116712826	116712371	116712439	116714839	116715099	39724	6,1	3,1	6,5	0,0	252	149	0.000238650649061	0.0179077201992	0.542,0.372	1.0,1.0	-0.543
6-8m_vs_8-10w	SE	Sec61a2	ENSMUSG00000025816.15	chr2	-	5876359	5876561	5873797	5873819	5876840	5877024	39759	49,12	0,1	11,16	1,5	298	149	0.000533926441149	0.028819464847	1.0,0.857	0.846,0.615	0.198
6-8m_vs_8-10w	SE	Tmem116	ENSMUSG00000029452.18	chr5	+	121482332	121482437	121467853	121467985	121487749	121487799	39838	4,0	7,2	3,4	1,0	253	149	5.64686478015e-05	0.00679231120896	0.252,0.0	0.639,1.0	-0.694
6-8m_vs_8-10w	SE	Mrpl2	ENSMUSG00000002767.13	chr17	+	46648669	46648769	46648471	46648587	46649034	46649108	39892	112,64	10,3	99,46	2,0	248	149	0.000987981716868	0.0431138372254	0.871,0.928	0.967,1.0	-0.084
6-8m_vs_8-10w	SE	CAAA01093181.2	ENSMUSG00000054753.9	chr7	-	3158554	3158657	3158217	3158429	3158754	3158894	39944	5,2	6,0	6,1	0,0	251	149	0.00115302572089	0.0479868279732	0.331,1.0	1.0,1.0	-0.335

6-8m_vs_8-10w	SE	Nmbr	ENSMUSG00000019865.9	chr10	+	14766676	14767025	14734802	14734912	14770154	14770850	39986	0,2	1,2	1,1	0,0	298	149	6.1303059256e-05	0.0071193388617	0.0,0.333	1.0,1.0	-0.834
6-8m_vs_8-10w	SE	Tsta3	ENSMUSG00000022570.6	chr15	-	75926966	75927095	75926742	75926872	75928428	75928512	40116	4,1	1,3	8,1	0,0	277	149	8.36271553373e-05	0.0089287974287 7	0.683,0.152	1.0,1.0	-0.583
6-8m_vs_8-10w	SE	Surf2	ENSMUSG00000014873.15	chr2	+	26917427	26917531	26916668	26916859	26918864	26919055	40192	53,32	0,0	34,20	3,3	252	149	0.0006197021852 5	0.0313917277311	1.0,1.0	0.87,0.798	0.166
6-8m_vs_8-10w	SE	Med27	ENSMUSG00000026799.15	chr2	+	29389812	29389873	29349845	29349990	29471292	29471386	40226	2,1	0,0	0,0	2,3	209	149	8.92650953155e-10	1.91122520077e-06	1.0,1.0	0.0,0.0	1.0
6-8m_vs_8-10w	SE	Hira	ENSMUSG00000022702.15	chr16	+	18947143	18947242	18946345	18946547	18947413	18947518	40324	5,1	1,0	0,0	1,2	247	149	1.32514161866e-08	9.67232914202e-06	0.751,1.0	0.0,0.0	0.876
6-8m_vs_8-10w	SE	Clybl	ENSMUSG00000025545.10	chr14	+	122371240	122371429	122311243	122311430	122401946	122402227	40333	299,195	1,1	318,213	15,5	298	149	2.4897281123e-05	0.0037190747932 4	0.993,0.99	0.914,0.955	0.057
6-8m_vs_8-10w	SE	Clybl	ENSMUSG00000025545.10	chr14	+	122375881	122375983	122311243	122311430	122379202	122379370	40334	5,3	0,0	5,0	2,2	250	149	4.70212730722e-05	0.0060405408239 5	1.0,1.0	0.598,0.0	0.701
6-8m_vs_8-10w	SE	Clybl	ENSMUSG00000025545.10	chr14	+	122375881	122375983	122311243	122311430	122401946	122402227	40335	5,3	1,1	5,1	15,5	250	149	0.000560408161795	0.0296360686419	0.749,0.641	0.166,0.107	0.559
6-8m_vs_8-10w	SE	Clybl	ENSMUSG00000025545.10	chr14	+	122384197	122384322	122311243	122311430	122401946	122402227	40346	95,107	1,1	125,53	15,5	273	149	1.2785014063e-06	0.000414751021866	0.981,0.983	0.82,0.853	0.145
6-8m_vs_8-10w	SE	Magi3	ENSMUSG00000052539.15	chr3	-	104016601	104016692	104013258	104016068	104017500	104017639	40470	1,1	4,4	8,2	1,1	239	149	7.84401677482e-05	0.0085686545149 7	0.135,0.135	0.833,0.555	-0.559
6-8m_vs_8-10w	SE	Rcc1	ENSMUSG00000028896.13	chr4	-	132339202	132339241	132338050	132338238	132339924	132340006	40605	0,1	2,1	2,4	1,0	187	149	1.03477716109e-05	0.0020019821268 4	0.0,0.443	0.614,1.0	-0.585
6-8m_vs_8-10w	SE	Taf1b	ENSMUSG00000059669.8	chr12	+	24504946	24505034	24500435	24500534	24509453	24509549	40674	35,27	0,2	26,30	4,11	236	149	0.000112028778366	0.0108370971265	1.0,0.895	0.804,0.633	0.229
6-8m_vs_8-10w	SE	Taf1b	ENSMUSG00000059669.8	chr12	+	24509083	24509181	24500435	24500534	24509453	24509549	40675	35,18	0,2	21,41	4,11	246	149	0.000330055686386	0.021765231951	1.0,0.845	0.761,0.693	0.196
6-8m_vs_8-10w	SE	Lnpk	ENSMUSG00000009207.15	chr2	-	74537303	74537501	74520290	74522297	74568940	74569128	40720	1,2	0,0	2,1	1,5	298	149	1.96978767741e-08	1.35165288667e-05	1.0,1.0	0.5,0.091	0.705
6-8m_vs_8-10w	SE	Lnpk	ENSMUSG00000009207.15	chr2	-	74551060	74551101	74520290	74522297	74568940	74569128	40724	3,2	0,0	4,4	1,5	189	149	1.82203650236e-05	0.0030008474005	1.0,1.0	0.759,0.387	0.427
6-8m_vs_8-10w	SE	Lnpk	ENSMUSG00000009207.15	chr2	-	74554987	74555046	74520290	74522297	74568940	74569128	40727	42,16	0,0	18,31	1,5	207	149	0.0010558449962 9	0.0450325602933	1.0,1.0	0.928,0.817	0.127
6-8m_vs_8-10w	SE	Cog4	ENSMUSG00000031753.16	chr8	+	110865976	110866095	110863250	110863384	110866560	110866727	40784	20,12	4,2	8,3	0,0	267	149	0.000407864744208	0.0248062579678	0.736,0.77	1.0,1.0	-0.247
6-8m_vs_8-10w	SE	Mum1l1	ENSMUSG00000042515.13	chrX	+	139210738	139210806	139210041	139210217	139213090	139213238	40929	4,3	2,2	1,3	0,0	216	149	0.000341969117796	0.0221425003773	0.58,0.509	1.0,1.0	-0.456
6-8m_vs_8-10w	SE	RP23-250A14.2	ENSMUSG00000087174.7	chrX	-	105043129	105043304	105042603	105042817	105044001	105044111	41031	4,8	0,0	0,0	1,3	298	149	3.43732819985e-11	2.75983081166e-07	1.0,1.0	0.0,0.0	1.0

6-8m_vs_8-10w	SE	RP23-250A14.2	ENSMUSG00000087174.7	chrX	-	105044001	105044111	105038807	105039812	105066758	105066877	41033	12,30	1,0	5,9	13,4	258	149	1.0777477899e-07	5.67425377384e-05	0.874,1.0	0.182,0.565	0.564
6-8m_vs_8-10w	SE	RP23-250A14.2	ENSMUSG00000087174.7	chrX	-	105047908	105048010	105037196	105039812	105066758	105066877	41034	33,19	1,0	7,13	13,4	250	149	2.95586856769e-07	0.000133705175944	0.952,1.0	0.243,0.66	0.525
6-8m_vs_8-10w	SE	Slc1a3	ENSMUSG00000005360.14	chr15	-	8687710	8687812	8680520	8681176	8688287	8688425	41159	2,0	1,4	1,4	1,0	250	149	0.000380078416782	0.0238410125652	0.544,0.0	0.373,1.0	-0.415
6-8m_vs_8-10w	SE	1700086O06Rik	ENSMUSG00000097080.7	chr18	-	38240838	38240953	38238404	38239189	38249713	38250198	41168	1,2	0,0	0,1	1,2	263	149	1.88599364059e-06	0.000536022758949	1.0,1.0	0.0,0.221	0.89
6-8m_vs_8-10w	SE	1700086O06Rik	ENSMUSG00000097080.7	chr18	-	38240838	38240953	38239572	38239751	38250177	38250565	41171	4,1	3,3	5,2	0,0	263	149	7.66859479251e-08	4.47790164284e-05	0.43,0.159	1.0,1.0	-0.706
6-8m_vs_8-10w	SE	Ncor1	ENSMUSG00000018501.17	chr11	-	62349352	62349493	62345234	62345318	62351377	62351546	41175	2,5	1,2	10,3	0,0	289	149	0.000785484103674	0.0365603006864	0.508,0.563	1.0,1.0	-0.465
6-8m_vs_8-10w	SE	Ncor1	ENSMUSG00000018501.17	chr11	-	62349394	62349493	62345234	62345318	62351377	62351390	41176	1,0	1,2	5,1	0,0	247	149	2.75637115887e-06	0.000713900130147	0.376,0.0	1.0,1.0	-0.812
6-8m_vs_8-10w	SE	Cers2	ENSMUSG00000015714.11	chr3	+	95320941	95321060	95320641	95320759	95321189	95321247	41305	36,4	1,0	12,13	6,1	267	149	0.000423571557466	0.0252851749806	0.953,1.0	0.527,0.879	0.273
6-8m_vs_8-10w	SE	Iars	ENSMUSG00000037851.13	chr13	+	49731417	49731561	49730014	49730140	49732339	49732492	41454	430,320	5,0	387,376	7,15	292	149	0.000368576052659	0.0235800567872	0.978,1.0	0.966,0.927	0.042
6-8m_vs_8-10w	SE	Ndufs1	ENSMUSG00000025968.16	chr1	-	63172153	63172257	63170848	63170914	63176585	63176669	41558	4,2	0,0	2,2	4,1	252	149	7.74995376651e-07	0.000286089097891	1.0,1.0	0.228,0.542	0.615
6-8m_vs_8-10w	SE	Cfdp1	ENSMUSG00000031954.6	chr8	-	111840357	111840485	111779250	111779409	111845093	111845211	41696	1,6	3,2	3,3	0,0	276	149	3.17251119109e-06	0.000783756687793	0.153,0.618	1.0,1.0	-0.615
6-8m_vs_8-10w	SE	Meg3	ENSMUSG00000021268.17	chr12	+	109549290	109549371	109546429	109546542	109549478	109549552	41711	4,3	7,2	2,7	0,0	229	149	3.48889208635e-08	2.19704427932e-05	0.271,0.494	1.0,1.0	-0.617
6-8m_vs_8-10w	SE	RP23-455F6.7	ENSMUSG00000115536.1	chr14	-	44232703	44232806	44226219	44227010	44311922	44312363	41739	7,9	5,2	8,7	0,0	251	149	4.60974764294e-06	0.00107891352453	0.454,0.728	1.0,1.0	-0.409
6-8m_vs_8-10w	SE	AC161108.3	ENSMUSG00000033488.11	chr1	+	157472187	157472416	157470615	157470797	157488442	157489060	41799	4,5	0,2	4,1	10,4	298	149	0.000725485960328	0.0347238555915	1.0,0.556	0.167,0.111	0.639
6-8m_vs_8-10w	SE	Kif18a	ENSMUSG00000027115.14	chr2	+	109292064	109292169	109289678	109289836	109292970	109293081	41833	1231,797	37,13	840,798	46,32	253	149	0.00120116918144	0.0485990738756	0.951,0.973	0.915,0.936	0.036
6-8m_vs_8-10w	SE	Kif18a	ENSMUSG00000027115.14	chr2	+	109292970	109293081	109289678	109289836	109302802	109302965	41840	28,9	14,10	42,24	5,4	259	149	0.000416267701331	0.0250651185671	0.535,0.341	0.829,0.775	-0.364
6-8m_vs_8-10w	SE	Kif18a	ENSMUSG00000027115.14	chr2	+	109292970	109293081	109289678	109289836	109310546	109310668	41842	28,8	6,10	42,26	3,1	259	149	0.000801873138587	0.0370546154228	0.729,0.315	0.89,0.937	-0.391
6-8m_vs_8-10w	SE	Stk36	ENSMUSG00000033276.18	chr1	+	74633506	74633654	74633141	74633289	74634009	74634100	41896	1,0	0,2	6,6	0,0	296	149	0.00122005826665	0.0491636026245	1.0,0.0	1.0,1.0	-0.5
6-8m_vs_8-10w	SE	Spns1	ENSMUSG00000030741.15	chr7	-	126373921	126374073	126373769	126373836	126375066	126375203	41914	2,5	3,0	1,0	3,3	298	149	0.000523915651191	0.028638998816	0.25,1.0	0.143,0.0	0.554

6-8m_vs_8-10w	SE	Gpx4	ENSMUSG00000075706.10	chr10	+	80054699	80054844	80054464	80054608	80054967	80055107	41918	845,428	132,49	708,377	47,35	293	149	0.000419280280625	0.0250756154424	0.765,0.816	0.885,0.846	-0.075
6-8m_vs_8-10w	SE	Chtf8	ENSMUSG00000046691.13	chr8	-	106886919	106887051	106886121	106886526	106887300	106887356	41925	0,1	1,4	3,3	1,3	280	149	0.00115950959016	0.0480500774162	0.0,0.117	0.615,0.347	-0.423
6-8m_vs_8-10w	SE	Cdk8	ENSMUSG00000029635.15	chr5	+	146286107	146286239	146271594	146271735	146296358	146296431	41975	6,5	0,0	3,5	4,3	280	149	7.90924485905e-08	4.48153663893e-05	1.0,1.0	0.285,0.47	0.623
6-8m_vs_8-10w	SE	Slc30a6	ENSMUSG00000024069.12	chr17	+	74412273	74412322	74410333	74410428	74412606	74412726	42085	135,73	4,7	130,146	0,0	197	149	0.000219006152277	0.0169484375579	0.962,0.887	1.0,1.0	-0.076
6-8m_vs_8-10w	SE	Slc30a6	ENSMUSG00000024069.12	chr17	+	74418619	74418667	74415619	74415722	74423016	74424229	42087	45,20	0,0	26,35	0,7	196	149	0.0006950256484 5	0.0338716900237	1.0,1.0	1.0,0.792	0.104
6-8m_vs_8-10w	SE	Slc30a6	ENSMUSG00000024069.12	chr17	+	74419531	74419600	74415619	74415722	74423016	74424229	42088	57,36	0,0	45,36	0,7	217	149	0.000946931617316	0.0417742525024	1.0,1.0	1.0,0.779	0.111
6-8m_vs_8-10w	SE	Slc22a21	ENSMUSG00000063652.10	chr11	-	53951706	53951842	53949964	53951355	53953000	53953183	42155	4,5	0,0	2,3	3,1	284	149	6.25711342861e-05	0.0072040844290 7	1.0,1.0	0.259,0.611	0.565
6-8m_vs_8-10w	SE	Colq	ENSMUSG00000057606.14	chr14	-	31525985	31526088	31523081	31524479	31528275	31528396	42164	7,6	2,2	4,4	0,0	251	149	0.000956509686857	0.0421389096064	0.675,0.64	1.0,1.0	-0.343
6-8m_vs_8-10w	SE	Cenpa	ENSMUSG00000029177.9	chr5	+	30673266	30673443	30672981	30673059	30674037	30674386	42214	1847,1092	194,118	1504,1088	96,67	298	149	0.000181009969925	0.015338565156	0.826,0.822	0.887,0.89	-0.065
6-8m_vs_8-10w	SE	Cenpa	ENSMUSG00000029177.9	chr5	+	30673292	30673443	30672981	30673059	30674037	30674202	42215	3434,2035	194,118	2881,2017	96,67	298	149	0.000106890098775	0.0105963491548	0.898,0.896	0.938,0.938	-0.041
6-8m_vs_8-10w	SE	RP23-366P9.8	ENSMUSG00000107071.1	chr6	+	39587368	39587628	39586840	39586894	39587776	39587848	42372	5,13	1,0	6,4	4,2	298	149	0.0002007352821	0.0161574293732	0.714,1.0	0.429,0.5	0.392
6-8m_vs_8-10w	SE	RP23-382G13.1	ENSMUSG00000108060.2	chr6	+	54009984	54010086	54009711	54009786	54016210	54018030	42410	33,9	0,0	19,9	1,4	250	149	0.000870666314699	0.0392178392186	1.0,1.0	0.919,0.573	0.254
6-8m_vs_8-10w	SE	Rad50	ENSMUSG00000020380.16	chr11	-	53654876	53655019	53652233	53652367	53655532	53655618	42424	134,74	3,3	68,57	14,6	291	149	9.7189923052e-05	0.0098155709708 7	0.958,0.927	0.713,0.829	0.172
6-8m_vs_8-10w	SE	Nr1i3	ENSMUSG00000005677.14	chr1	+	171217625	171217731	171217075	171217430	171218588	171218844	42476	3,2	1,0	0,1	2,1	254	149	8.68815048305e-06	0.0017330971485 3	0.638,1.0	0.0,0.37	0.634
6-8m_vs_8-10w	SE	Pcgf1	ENSMUSG00000069678.10	chr6	+	83079639	83079745	83079433	83079505	83079921	83079955	42504	2390,2407	48,46	2491,1949	17,19	254	149	2.75608394298e-05	0.0040051761046 5	0.967,0.968	0.988,0.984	-0.018
6-8m_vs_8-10w	SE	Prg4	ENSMUSG00000006014.16	chr1	-	150451853	150452010	150451248	150451449	150452222	150452359	42526	68,59	7,6	29,64	0,1	298	149	9.16318985535e-05	0.0094120016070 1	0.829,0.831	1.0,0.97	-0.155
6-8m_vs_8-10w	SE	Guca1a	ENSMUSG00000023982.7	chr17	-	47395547	47395697	47395107	47395201	47400218	47400584	42562	1408,730	131,58	1335,662	40,47	298	149	7.33192604219e-05	0.0081478248017 6	0.843,0.863	0.943,0.876	-0.056
6-8m_vs_8-10w	SE	Metap2	ENSMUSG00000036112.15	chr10	-	93880072	93880138	93871487	93871649	93886993	93887098	42807	167,127	1,0	158,107	5,6	214	149	0.0011680927571 7	0.0482191092407	0.991,1.0	0.957,0.925	0.054
6-8m_vs_8-10w	SE	Cadm1	ENSMUSG00000032076.19	chr9	+	47836716	47836770	47829376	47829409	47848166	47848298	42817	1,4	1,0	0,0	1,2	202	149	3.14077464036e-07	0.000138176874452	0.425,1.0	0.0,0.0	0.713

6-8m_vs_8-10w	SE	Bcl2l14	ENSMUSG00000030200.13	chr6	+	134423610	134424062	134414427	134414635	134427295	134427460	42819	56,24	5,8	59,19	0,0	298	149	2.8220584507e-06	0.000719311342878	0.848,0.6	1.0,1.0	-0.276
6-8m_vs_8-10w	SE	Prpf39	ENSMUSG00000035597.19	chr12	+	65042460	65042807	65036332	65036468	65043231	65043357	43230	240,171	9,14	114,137	17,23	298	149	0.000251864321469	0.0185951139041	0.93,0.859	0.77,0.749	0.135
6-8m_vs_8-10w	SE	Ifi27	ENSMUSG00000064215.13	chr12	+	103437389	103437524	103436562	103436682	103437675	103437705	43325	9,3	1,0	18,8	7,3	283	149	0.000913735164294	0.040766793916	0.826,1.0	0.575,0.584	0.334
6-8m_vs_8-10w	SE	Tcf12	ENSMUSG00000032228.16	chr9	-	71868998	71869070	71868071	71868278	71870073	71870147	43584	9,8	0,0	14,15	3,4	220	149	0.000113025575913	0.0109006888769	1.0,1.0	0.76,0.717	0.262
6-8m_vs_8-10w	SE	Klhl15	ENSMUSG00000043929.16	chrX	+	94238048	94238157	94235220	94235420	94252414	94255968	43598	6,3	8,0	5,5	0,0	257	149	0.000794069308314	0.0368739215889	0.303,1.0	1.0,1.0	-0.349
6-8m_vs_8-10w	SE	Klhl15	ENSMUSG00000043929.16	chrX	+	94241944	94241995	94235220	94235420	94252414	94252740	43599	56,8	8,0	18,19	0,0	199	149	0.000592672427612	0.0305036341109	0.84,1.0	1.0,1.0	-0.08
6-8m_vs_8-10w	SE	Sycp1	ENSMUSG00000027855.13	chr3	-	102840861	102841041	102820538	102820682	102845031	102845088	43616	180,139	14,11	114,116	20,24	298	149	0.00111172122388	0.0466719461779	0.865,0.863	0.74,0.707	0.14
6-8m_vs_8-10w	SE	Sycp1	ENSMUSG00000027855.13	chr3	-	102932642	102932807	102931627	102931695	102934204	102934261	43647	687,527	189,166	486,356	292,177	298	149	1.34334304369e-06	0.000422127906596	0.645,0.614	0.454,0.501	0.152
6-8m_vs_8-10w	SE	Tars2	ENSMUSG00000028107.14	chr3	-	95745784	95745862	95742577	95742678	95746032	95746170	43758	10,5	3,3	7,7	0,0	226	149	1.88413550357e-05	0.0030716190778	0.687,0.524	1.0,1.0	-0.394
6-8m_vs_8-10w	SE	Ipmk	ENSMUSG00000060733.13	chr10	+	71363449	71363535	71347762	71348090	71372704	71372880	43869	70,36	0,0	44,35	3,5	234	149	0.000138761038239	0.0126603679093	1.0,1.0	0.903,0.817	0.14
6-8m_vs_8-10w	SE	Phtf2	ENSMUSG00000039987.15	chr5	-	20801901	20802066	20793301	20794481	20805654	20805707	43885	261,187	0,1	211,156	8,4	298	149	0.000370552882394	0.023612453117	1.0,0.989	0.93,0.951	0.054
6-8m_vs_8-10w	SE	Phtf2	ENSMUSG00000039987.15	chr5	-	20803184	20803350	20793301	20794481	20805654	20805813	43886	363,216	0,1	247,195	8,4	298	149	0.000623562632351	0.0314879520449	1.0,0.991	0.939,0.961	0.046
6-8m_vs_8-10w	SE	Phtf2	ENSMUSG00000039987.15	chr5	-	20855121	20855201	20813239	20813296	20881955	20882045	43894	204,180	1,0	157,131	9,2	228	149	0.000846486041942	0.0385067219873	0.993,1.0	0.919,0.977	0.048
6-8m_vs_8-10w	SE	Msantd2	ENSMUSG00000042138.8	chr9	+	37491455	37491569	37489599	37489833	37518726	37518787	43903	0,0	5,5	4,2	1,2	262	149	7.26212963187e-06	0.00151448412505	0.0,0.0	0.695,0.363	-0.529
6-8m_vs_8-10w	SE	Msantd2	ENSMUSG00000042138.8	chr9	+	37517372	37517472	37491455	37491569	37518726	37518752	43908	19,15	0,0	19,20	5,3	248	149	3.49799400269e-05	0.00481158299422	1.0,1.0	0.695,0.8	0.252
6-8m_vs_8-10w	SE	Masp2	ENSMUSG00000028979.17	chr4	+	148607940	148608059	148606028	148606176	148612034	148612166	43943	22,12	0,0	10,3	3,1	267	149	0.000376636378758	0.0237721098649	1.0,1.0	0.65,0.626	0.362
6-8m_vs_8-10w	SE	Igf2bp2	ENSMUSG00000033581.16	chr16	-	22151974	22152004	22151463	22151652	22161245	22161306	44033	1,0	11,1	2,5	4,3	178	149	0.000209375385048	0.0166032095462	0.071,0.0	0.295,0.582	-0.403
6-8m_vs_8-10w	SE	Pqlc2	ENSMUSG00000028744.15	chr4	-	139295773	139295933	139294028	139294592	139301011	139301104	44114	12,8	0,0	9,7	3,2	298	149	0.00011398277708	0.0109273757275	1.0,1.0	0.6,0.636	0.382
6-8m_vs_8-10w	SE	Pqlc2	ENSMUSG00000028744.15	chr4	-	139298645	139298969	139294028	139294592	139301011	139301104	44117	3,3	0,0	0,1	3,2	298	149	5.66202640329e-11	3.09040578461e-07	1.0,1.0	0.0,0.2	0.9

6-8m_vs_8-10w	SE	Pqlc2	ENSMUSG00000028744.15	chr4	-	139298645	139298969	139295773	139295933	139302424	139302554	44122	2,1	0,0	2,0	1,3	298	149	6.05715160251e-07	0.000234375278152	1.0,1.0	0.5,0.0	0.75
6-8m_vs_8-10w	SE	RP23-78N8.3	ENSMUSG00000040177.10	chr7	-	131348691	131351102	131343185	131343804	131361732	131361936	44255	5,4	2,4	8,3	0,0	298	149	6.43697802172e-07	0.000243211748407	0.556,0.333	1.0,1.0	-0.556
6-8m_vs_8-10w	SE	RP23-78N8.3	ENSMUSG00000040177.10	chr7	-	131357459	131357666	131338219	131343804	131361732	131361936	44260	6,6	2,4	4,2	0,0	298	149	2.94552045854e-06	0.000744868779893	0.6,0.429	1.0,1.0	-0.486
6-8m_vs_8-10w	SE	Fbxo17	ENSMUSG00000030598.15	chr7	+	28737338	28737546	28736067	28736203	28737664	28738138	44268	3,1	89,85	20,9	69,36	298	149	6.28684004855e-09	5.93847514704e-06	0.017,0.006	0.127,0.111	-0.108
6-8m_vs_8-10w	SE	Prr14l	ENSMUSG00000054280.14	chr5	-	32844043	32844564	32835615	32835688	32854092	32854256	44350	91,66	1,0	54,64	5,7	298	149	1.37598057605e-05	0.0024687705128 7	0.978,1.0	0.844,0.821	0.156
6-8m_vs_8-10w	SE	Prr14l	ENSMUSG00000054280.14	chr5	-	32851881	32851998	32835615	32835688	32854092	32854256	44351	6,8	1,0	9,5	5,7	265	149	5.39796349928e-05	0.0066168318985 8	0.771,1.0	0.503,0.287	0.49
6-8m_vs_8-10w	SE	Tyms	ENSMUSG00000025747.12	chr5	-	30069833	30069955	30068465	30068640	30071633	30071707	44392	13,3	58,50	1,1	86,29	270	149	0.000124321145459	0.0115395893282	0.11,0.032	0.006,0.019	0.059
6-8m_vs_8-10w	SE	Cpsf1	ENSMUSG00000034022.8	chr15	-	76596415	76596571	76596219	76596349	76596650	76596698	44400	16,8	10,5	27,22	0,0	298	149	4.77175521318e-10	1.39317900388e-06	0.444,0.444	1.0,1.0	-0.556
6-8m_vs_8-10w	SE	Trmt13	ENSMUSG00000033439.12	chr3	-	116588471	116588524	116585754	116585827	116589490	116589597	44463	23,7	0,1	6,13	4,3	201	149	0.000333780498961	0.0218769275605	1.0,0.838	0.527,0.763	0.274
6-8m_vs_8-10w	SE	Trmt13	ENSMUSG00000033439.12	chr3	-	116588471	116588639	116585754	116585827	116589490	116589597	44464	85,52	0,1	46,43	4,3	298	149	0.000932904045816	0.0413257190833	1.0,0.963	0.852,0.878	0.117
6-8m_vs_8-10w	SE	RP23-186O3.11	ENSMUSG00000111497.1	chr9	-	21068029	21068117	21067513	21067925	21076566	21076641	44702	39,23	5,5	22,28	0,0	236	149	2.50625051259e-05	0.0037264232158 5	0.831,0.744	1.0,1.0	-0.213
6-8m_vs_8-10w	SE	RP23-186O3.11	ENSMUSG00000111497.1	chr9	-	21075963	21076107	21067513	21067925	21076566	21076641	44710	7,8	5,5	3,3	0,0	292	149	2.99450232044e-06	0.000746807846918	0.417,0.449	1.0,1.0	-0.567
6-8m_vs_8-10w	SE	Fxr1	ENSMUSG00000027680.15	chr3	+	34064973	34065054	34064118	34064319	34068160	34068252	44828	12,17	91,79	2,5	95,79	229	149	0.000121841414793	0.0113751711555	0.079,0.123	0.014,0.04	0.074
6-8m_vs_8-10w	SE	Pdia4	ENSMUSG00000025823.9	chr6	-	47808174	47808343	47807005	47807211	47813216	47813430	44835	114,48	0,3	60,50	5,7	298	149	0.000842850231983	0.0383957135466	1.0,0.889	0.857,0.781	0.126
6-8m_vs_8-10w	SE	Mpv17l2	ENSMUSG00000035559.9	chr8	-	70759390	70759467	70759108	70759237	70760367	70760538	44851	63,15	15,7	45,26	4,0	225	149	0.000527692946852	0.028638998816	0.736,0.587	0.882,1.0	-0.28
6-8m_vs_8-10w	SE	Zfyve19	ENSMUSG00000068580.11	chr2	+	119216431	119216541	119216208	119216307	119216628	119217049	44912	19,10	8,1	11,23	0,1	258	149	0.000253521370439	0.0186317902357	0.578,0.852	1.0,0.93	-0.25
6-8m_vs_8-10w	SE	Orc4	ENSMUSG00000026761.12	chr2	-	48929680	48929729	48917164	48917338	48949116	48949231	44935	3,2	4,7	0,0	1,2	197	149	0.000763358558747	0.0360529756952	0.362,0.178	0.0,0.0	0.27
6-8m_vs_8-10w	SE	Mrpl4	ENSMUSG00000003299.10	chr9	+	21007327	21007540	21006826	21006856	21007822	21007877	45013	7,3	0,0	6,2	4,2	298	149	9.70616743423e-07	0.000342553047602	1.0,1.0	0.429,0.333	0.619
6-8m_vs_8-10w	SE	Mrpl4	ENSMUSG00000003299.10	chr9	+	21007433	21007540	21006826	21006856	21007822	21007899	45020	13,4	0,0	4,1	4,2	255	149	1.18631110158e-07	6.14509150618e-05	1.0,1.0	0.369,0.226	0.703

6-8m_vs_8-10w	SE	Mrpl4	ENSMUSG00000003299.10	chr9	+	21007630	21007740	21006826	21006944	21007822	21007877	45024	134,53	24,8	126,60	6,3	258	149	0.000376759369823	0.0237721098649	0.763,0.793	0.924,0.92	-0.144
6-8m_vs_8-10w	SE	RP23-268A17.3	ENSMUSG00000111901.1	chr10	+	9688795	9688890	9684721	9685082	9689412	9691906	45053	4,5	3,4	3,5	0,0	243	149	2.75465124355e-06	0.000713900130147	0.45,0.434	1.0,1.0	-0.558
6-8m_vs_8-10w	SE	Zfp672	ENSMUSG00000049755.14	chr11	-	58319866	58319954	58319323	58319590	58330211	58330331	45097	1,4	4,2	2,5	0,0	236	149	3.2231332936e-07	0.000139378152444	0.136,0.558	1.0,1.0	-0.653
6-8m_vs_8-10w	SE	Zfp672	ENSMUSG00000049755.14	chr11	-	58322599	58322729	58319323	58319590	58330211	58330331	45100	14,8	4,2	14,9	0,0	278	149	6.9418702612e-05	0.0077681221361 9	0.652,0.682	1.0,1.0	-0.333
