# Supplementary material for: Integrative proteome analysis implicates aberrant RNA splicing in impaired developmental potential of aged mouse oocytes
Source: Aging Cell. 2021 Sep 28;20(10):e13482. doi: 10.1111/acel.13482 (PMC8520726; doi:10.1111/acel.13482)
Supplement: Supplementary file 10 — Table S6 [file ACEL-20-e13482-s007.pdf]

Table S6. List of PUF60-binding DSGs in the two reproductively aging groups versus the younger group.

sequence name	score	p-value
8-Mar	10.1954	9.66E-05
8-Mar	10.1954	9.66E-05
8-Mar	10.7126	7.93E-05
2-Sep	10.7126	7.93E-05
1700086O06Rik	10.7126	7.93E-05
1700086O06Rik	10.7126	7.93E-05
Aaas	10.1954	9.66E-05
Aaas	10.1954	9.66E-05
Aaas	10.1954	9.66E-05
Aak1	10.1954	9.66E-05
Aak1	10.1954	9.66E-05
Aak1	10.1954	9.66E-05
Aak1	10.7126	7.93E-05
Aamdc	10.1954	9.66E-05
Aamdc	10.1954	9.66E-05
Aamdc	10.1954	9.66E-05
Aamdc	10.1954	9.66E-05
Aamdc	10.1954	9.66E-05
Aamdc	10.1954	9.66E-05
Aamdc	10.1954	9.66E-05
Aamdc	10.1954	9.66E-05
Aamdc	10.1954	9.66E-05
Aamdc	10.1954	9.66E-05
Aamdc	10.7126	7.93E-05
Aamdc	10.7126	7.93E-05
Aamdc	10.7126	7.93E-05
Aamdc	10.7126	7.93E-05
Aamdc	10.7126	7.93E-05
Abca5	10.1954	9.66E-05
Abca5	10.1954	9.66E-05
Abca5	10.1954	9.66E-05
Abca5	10.1954	9.66E-05
Abca5	10.1954	9.66E-05
Abca5	10.1954	9.66E-05
Abca5	10.7126	7.93E-05
Abca5	10.7126	7.93E-05
Abca5	10.7126	7.93E-05
Abca8b	10.1954	9.66E-05
Abca8b	10.7126	7.93E-05
Abca8b	10.7126	7.93E-05
Abhd3	10.1954	9.66E-05
Abhd3	10.1954	9.66E-05
Abo	10.1954	9.66E-05
Abo	10.1954	9.66E-05
AC121113.1	10.1954	9.66E-05
AC121576.1	10.7126	7.93E-05
AC122423.2	10.1954	9.66E-05
AC122423.2	10.1954	9.66E-05
AC122423.2	10.1954	9.66E-05
AC122423.2	10.1954	9.66E-05
AC122423.2	10.7126	7.93E-05
AC122423.2	10.7126	7.93E-05
AC122423.2	10.7126	7.93E-05
AC122423.2	10.1954	9.66E-05
AC122423.2	10.7126	7.93E-05

AC123684.1	10.1954	9.66E-05
AC123684.1	10.1954	9.66E-05
AC123684.1	10.1954	9.66E-05
AC123684.1	10.1954	9.66E-05
AC123686.3	10.1954	9.66E-05
AC123686.3	10.1954	9.66E-05
AC123686.3	10.1954	9.66E-05
AC123686.3	10.1954	9.66E-05
AC123686.3	10.1954	9.66E-05
AC123686.3	10.1954	9.66E-05
AC123686.3	10.1954	9.66E-05
AC123686.3	10.1954	9.66E-05
AC123686.3	10.1954	9.66E-05
AC123686.3	10.7126	7.93E-05
AC123686.3	10.7126	7.93E-05
AC123686.3	10.7126	7.93E-05
AC123686.3	10.7126	7.93E-05
AC123856.3	10.1954	9.66E-05
AC123856.3	10.1954	9.66E-05
AC123856.3	10.7126	7.93E-05
AC123856.3	10.1954	9.66E-05
AC123856.3	10.1954	9.66E-05
AC134411.2	10.1954	9.66E-05
AC145199.4	10.7126	7.93E-05
AC152939.2	10.7126	7.93E-05
AC161108.3	10.1954	9.66E-05
AC161108.3	10.7126	7.93E-05
AC161108.3	10.1954	9.66E-05
AC161108.3	10.7126	7.93E-05
AC167036.4	10.7126	7.93E-05
AC167036.4	10.7126	7.93E-05
AC167036.4	10.7126	7.93E-05
AC167036.4	10.7126	7.93E-05
Acbd5	10.1954	9.66E-05
Acbd5	10.1954	9.66E-05
Acbd5	10.1954	9.66E-05
Acbd5	10.1954	9.66E-05
Acbd5	10.1954	9.66E-05
Acbd5	10.1954	9.66E-05
Acsbg2	10.1954	9.66E-05
Acsbg2	10.1954	9.66E-05
Acsbg2	10.1954	9.66E-05
Acsbg2	10.1954	9.66E-05
Acsbg2	10.1954	9.66E-05
Acsbg2	10.1954	9.66E-05
Acsbg2	10.1954	9.66E-05
Actl6a	10.1954	9.66E-05
Actr6	10.1954	9.66E-05
Actr6	10.1954	9.66E-05
Adam5	10.1954	9.66E-05
Adam5	10.1954	9.66E-05
Adgrb3	10.1954	9.66E-05
Adgrb3	10.1954	9.66E-05
Adgrb3	10.1954	9.66E-05
Adgrb3	10.1954	9.66E-05
Adrm1	10.1954	9.66E-05
Adrm1	10.1954	9.66E-05
Adrm1	10.1954	9.66E-05

Adrm1	10.1954	9.66E-05
Akap11	10.1954	9.66E-05
Akap11	10.7126	7.93E-05
Alg10b	10.1954	9.66E-05
Alg10b	10.1954	9.66E-05
Alg5	10.7126	7.93E-05
Alg5	10.7126	7.93E-05
Alg5	10.7126	7.93E-05
Alg5	10.7126	7.93E-05
Alg9	10.1954	9.66E-05
Alg9	10.1954	9.66E-05
Alg9	10.1954	9.66E-05
Alg9	10.1954	9.66E-05
Alg9	10.7126	7.93E-05
Alg9	10.1954	9.66E-05
Alg9	10.1954	9.66E-05
Alg9	10.1954	9.66E-05
Alg9	10.1954	9.66E-05
Alg9	10.7126	7.93E-05
Alkbh3	10.7126	7.93E-05
Alkbh3	10.7126	7.93E-05
Alkbh7	10.7126	7.93E-05
Alkbh8	10.1954	9.66E-05
Alkbh8	10.1954	9.66E-05
Alkbh8	10.7126	7.93E-05
Alkbh8	10.1954	9.66E-05
Alkbh8	10.1954	9.66E-05
Alkbh8	10.1954	9.66E-05
Alkbh8	10.1954	9.66E-05
Alkbh8	10.7126	7.93E-05
Alkbh8	10.7126	7.93E-05
Ampd3	10.1954	9.66E-05
Anapc15	10.1954	9.66E-05
Anapc15	10.1954	9.66E-05
Anapc15	10.1954	9.66E-05
Anapc15	10.1954	9.66E-05
Anapc15	10.1954	9.66E-05
Anapc15	10.1954	9.66E-05
Anapc15	10.7126	7.93E-05
Ank3	10.1954	9.66E-05
Ank3	10.1954	9.66E-05
Ank3	10.1954	9.66E-05
Ank3	10.7126	7.93E-05
Ank3	10.7126	7.93E-05
Ankhd1	10.1954	9.66E-05
Ankhd1	10.1954	9.66E-05
Ankrd16	10.1954	9.66E-05
Ankrd17	10.1954	9.66E-05
Ankrd17	10.1954	9.66E-05
Ankrd17	10.1954	9.66E-05
Ankrd17	10.1954	9.66E-05
Ankrd17	10.1954	9.66E-05
Ankrd26	10.1954	9.66E-05
Ankrd31	10.1954	9.66E-05
Ankrd31	10.1954	9.66E-05
Ankrd31	10.1954	9.66E-05
Ankrd31	10.7126	7.93E-05
Ap1m1	10.7126	7.93E-05

Ap1m1	10.7126	7.93E-05
Ap2b1	10.7126	7.93E-05
Ap2b1	10.7126	7.93E-05
Ap4m1	10.1954	9.66E-05
Ap4m1	10.1954	9.66E-05
Ap4m1	10.1954	9.66E-05
Ap4m1	10.1954	9.66E-05
Ap4m1	10.1954	9.66E-05
Ap4m1	10.7126	7.93E-05
Ap4m1	10.1954	9.66E-05
Ap4m1	10.1954	9.66E-05
Ap4m1	10.1954	9.66E-05
Ap4m1	10.1954	9.66E-05
Ap4m1	10.1954	9.66E-05
Ap4m1	10.7126	7.93E-05
Apmap	10.1954	9.66E-05
Apoa1bp	10.7126	7.93E-05
Apoa1bp	10.7126	7.93E-05
Apoa1bp	10.7126	7.93E-05
Apoa1bp	10.7126	7.93E-05
Aprt	10.7126	7.93E-05
Aptx	10.1954	9.66E-05
Aptx	10.1954	9.66E-05
Aptx	10.7126	7.93E-05
Aptx	10.7126	7.93E-05
Arfrp1	10.7126	7.93E-05
Arfrp1	10.7126	7.93E-05
Arfrp1	10.7126	7.93E-05
Arfrp1	10.7126	7.93E-05
Arfrp1	10.7126	7.93E-05
Arhgap10	10.7126	7.93E-05
Arhgap10	10.7126	7.93E-05
Arhgap5	10.1954	9.66E-05
Arhgap5	10.1954	9.66E-05
Arhgap5	10.1954	9.66E-05
Arhgef7	10.7126	7.93E-05
Arl6ip6	10.7126	7.93E-05
Armc12	10.7126	7.93E-05
Armc6	10.7126	7.93E-05
Armc9	10.1954	9.66E-05
Armc9	10.1954	9.66E-05
Armc9	10.7126	7.93E-05
Armc9	10.1954	9.66E-05
Armc9	10.1954	9.66E-05
Armc9	10.1954	9.66E-05
Armc9	10.7126	7.93E-05
Armc9	10.7126	7.93E-05
Arntl2	10.1954	9.66E-05
Arntl2	10.1954	9.66E-05
Arntl2	10.7126	7.93E-05
Arntl2	10.7126	7.93E-05
Arntl2	10.7126	7.93E-05
Arpc1a	10.1954	9.66E-05
Arpc1a	10.1954	9.66E-05
Arpc1a	10.1954	9.66E-05
Arpc1a	10.1954	9.66E-05
Arpc1a	10.1954	9.66E-05
Arpc1a	10.1954	9.66E-05

Arpc1a	10.7126	7.93E-05
Arpc1a	10.7126	7.93E-05
Arpc1a	10.7126	7.93E-05
Asxl2	10.1954	9.66E-05
Asxl2	10.1954	9.66E-05
Asxl2	10.1954	9.66E-05
Atad3a	10.1954	9.66E-05
Atad3a	10.1954	9.66E-05
Atf2	10.1954	9.66E-05
Atf2	10.1954	9.66E-05
Atf2	10.1954	9.66E-05
Atf2	10.1954	9.66E-05
Atf2	10.1954	9.66E-05
Atf2	10.1954	9.66E-05
Atf2	10.1954	9.66E-05
Atf2	10.1954	9.66E-05
Atf2	10.1954	9.66E-05
Atf2	10.1954	9.66E-05
Atf2	10.1954	9.66E-05
Atf2	10.1954	9.66E-05
Atf2	10.1954	9.66E-05
Atf2	10.1954	9.66E-05
Atf2	10.1954	9.66E-05
Atf2	10.1954	9.66E-05
Atf2	10.1954	9.66E-05
Atf2	10.1954	9.66E-05
Atf2	10.7126	7.93E-05
Atf2	10.7126	7.93E-05
Atf2	10.7126	7.93E-05
Atf2	10.7126	7.93E-05
Atf2	10.7126	7.93E-05
Atf2	10.7126	7.93E-05
Atf2	10.1954	9.66E-05
Atf2	10.1954	9.66E-05
Atf2	10.1954	9.66E-05
Atf2	10.1954	9.66E-05
Atf2	10.1954	9.66E-05
Atf2	10.1954	9.66E-05
Atf2	10.1954	9.66E-05
Atf2	10.1954	9.66E-05
Atf2	10.1954	9.66E-05
Atf2	10.1954	9.66E-05
Atf2	10.1954	9.66E-05
Atf2	10.1954	9.66E-05
Atf2	10.1954	9.66E-05
Atf2	10.1954	9.66E-05
Atf2	10.1954	9.66E-05
Atf2	10.1954	9.66E-05
Atf2	10.1954	9.66E-05
Atf2	10.1954	9.66E-05
Atf2	10.1954	9.66E-05
Atf2	10.7126	7.93E-05
Atf2	10.7126	7.93E-05
Atf2	10.7126	7.93E-05
Atf2	10.7126	7.93E-05
Atf2	10.7126	7.93E-05
Atf2	10.7126	7.93E-05
Atf7ip2	10.1954	9.66E-05

Atf7ip2	10.1954	9.66E-05
Atf7ip2	10.7126	7.93E-05
Atf7ip2	10.1954	9.66E-05
Atf7ip2	10.1954	9.66E-05
Atf7ip2	10.1954	9.66E-05
Atf7ip2	10.7126	7.93E-05
Atg13	10.1954	9.66E-05
Atg13	10.1954	9.66E-05
Atg13	10.7126	7.93E-05
Atg13	10.7126	7.93E-05
Atg3	10.1954	9.66E-05
Atg3	10.1954	9.66E-05
Atg3	10.1954	9.66E-05
Atg3	10.1954	9.66E-05
Atp13a3	10.1954	9.66E-05
Atp13a3	10.1954	9.66E-05
Atp13a3	10.7126	7.93E-05
Atp13a3	10.7126	7.93E-05
Atp13a3	10.7126	7.93E-05
Atp13a3	10.1954	9.66E-05
Atp13a3	10.1954	9.66E-05
Atp13a3	10.7126	7.93E-05
Atp13a3	10.7126	7.93E-05
Atp13a3	10.7126	7.93E-05
Atp5h	10.7126	7.93E-05
Atp5h	10.7126	7.93E-05
Atp5h	10.7126	7.93E-05
Atp5j	10.7126	7.93E-05
Atp6v0d1	10.1954	9.66E-05
Atp9a	10.7126	7.93E-05
Atp9a	10.7126	7.93E-05
Atpaf2	10.1954	9.66E-05
Atpif1	10.1954	9.66E-05
Atpif1	10.1954	9.66E-05
Atpif1	10.1954	9.66E-05
Azin1	10.1954	9.66E-05
Azin1	10.1954	9.66E-05
Azin1	10.1954	9.66E-05
Azin1	10.1954	9.66E-05
Azin1	10.1954	9.66E-05
Baz2a	10.1954	9.66E-05
Baz2a	10.7126	7.93E-05
Bcas1	10.1954	9.66E-05
Bcl2l14	10.1954	9.66E-05
Bcl2l14	10.7126	7.93E-05
Bcl7c	10.1954	9.66E-05
Bcl7c	10.1954	9.66E-05
Bclaf1	10.1954	9.66E-05
Bclaf1	10.1954	9.66E-05
Bcs1l	10.1954	9.66E-05
Bcs1l	10.1954	9.66E-05
Becn1	10.7126	7.93E-05
Becn1	10.7126	7.93E-05
Becn1	10.7126	7.93E-05
Birc6	10.1954	9.66E-05
Birc6	10.1954	9.66E-05
Birc6	10.1954	9.66E-05
Birc6	10.1954	9.66E-05

Birc6	10.7126	7.93E-05
Bnc2	10.1954	9.66E-05
Bnc2	10.1954	9.66E-05
Bnc2	10.1954	9.66E-05
Bnc2	10.7126	7.93E-05
Bnc2	10.7126	7.93E-05
Bola1	10.1954	9.66E-05
Bola1	10.1954	9.66E-05
Bola1	10.7126	7.93E-05
Bola1	10.7126	7.93E-05
Bola1	10.1954	9.66E-05
Bola1	10.1954	9.66E-05
Bola1	10.7126	7.93E-05
Bola1	10.7126	7.93E-05
Bphl	10.1954	9.66E-05
Bphl	10.1954	9.66E-05
Braf	10.1954	9.66E-05
Braf	10.1954	9.66E-05
Brca2	10.7126	7.93E-05
Brca2	10.7126	7.93E-05
Brd8	10.1954	9.66E-05
Brd8	10.1954	9.66E-05
Brd8	10.1954	9.66E-05
Brd8	10.7126	7.93E-05
Brd9	10.1954	9.66E-05
Brwd1	10.7126	7.93E-05
Btbd19	10.1954	9.66E-05
Btbd19	10.7126	7.93E-05
C2cd3	10.1954	9.66E-05
C2cd3	10.1954	9.66E-05
C2cd3	10.1954	9.66E-05
C2cd3	10.1954	9.66E-05
C2cd3	10.1954	9.66E-05
Cab39l	10.7126	7.93E-05
Cab39l	10.7126	7.93E-05
Cab39l	10.1954	9.66E-05
Cab39l	10.1954	9.66E-05
Cab39l	10.7126	7.93E-05
Cacul1	10.1954	9.66E-05
Cacul1	10.1954	9.66E-05
Cacul1	10.1954	9.66E-05
Cacul1	10.1954	9.66E-05
Cacul1	10.1954	9.66E-05
Cacul1	10.1954	9.66E-05
Cacul1	10.1954	9.66E-05
Cacul1	10.1954	9.66E-05
Cacul1	10.1954	9.66E-05
Cacul1	10.1954	9.66E-05
Cadm1	10.1954	9.66E-05
Cadm1	10.1954	9.66E-05
Cadm1	10.1954	9.66E-05
Cadm1	10.7126	7.93E-05
Cadm1	10.1954	9.66E-05
Cadm1	10.1954	9.66E-05
Cadm1	10.1954	9.66E-05
Cadm1	10.1954	9.66E-05
Cadm1	10.1954	9.66E-05
Cadm1	10.1954	9.66E-05

Cadm1	10.1954	9.66E-05
Cadm1	10.1954	9.66E-05
Cadm1	10.7126	7.93E-05
Cadm1	10.7126	7.93E-05
Calm1	10.7126	7.93E-05
Calm1	10.7126	7.93E-05
Camk2g	10.7126	7.93E-05
Camk2g	10.7126	7.93E-05
Camkmt	10.7126	7.93E-05
Canx	10.1954	9.66E-05
Canx	10.1954	9.66E-05
Canx	10.1954	9.66E-05
Canx	10.1954	9.66E-05
Canx	10.1954	9.66E-05
Canx	10.7126	7.93E-05
Canx	10.7126	7.93E-05
Capn11	10.7126	7.93E-05
Capn11	10.7126	7.93E-05
Capn7	10.1954	9.66E-05
Capn7	10.1954	9.66E-05
Capn7	10.7126	7.93E-05
Carkd	10.7126	7.93E-05
Cars2	10.1954	9.66E-05
Cars2	10.1954	9.66E-05
Cars2	10.7126	7.93E-05
Cars2	10.7126	7.93E-05
Catsperd	10.1954	9.66E-05
Catsperd	10.1954	9.66E-05
Catsperd	10.1954	9.66E-05
Catsperd	10.1954	9.66E-05
Catsperg1	10.7126	7.93E-05
Catsperg1	10.7126	7.93E-05
Catsperg1	10.7126	7.93E-05
Catsperg1	10.7126	7.93E-05
Catsperg1	10.7126	7.93E-05
Catsperg1	10.7126	7.93E-05
Catsperg1	10.7126	7.93E-05
Catsperg1	10.7126	7.93E-05
Cbfa2t2	10.1954	9.66E-05
Cbfa2t2	10.7126	7.93E-05
Ccdc130	10.1954	9.66E-05
Ccdc163	10.1954	9.66E-05
Ccdc163	10.1954	9.66E-05
Ccdc163	10.1954	9.66E-05
Ccdc163	10.7126	7.93E-05
Ccdc163	10.7126	7.93E-05
Ccdc163	10.7126	7.93E-05
Ccdc163	10.1954	9.66E-05
Ccdc163	10.1954	9.66E-05
Ccdc163	10.1954	9.66E-05
Ccdc163	10.1954	9.66E-05
Ccdc163	10.1954	9.66E-05
Ccdc163	10.7126	7.93E-05
Ccdc178	10.7126	7.93E-05
Ccdc178	10.7126	7.93E-05
Ccdc60	10.7126	7.93E-05
Ccdc62	10.1954	9.66E-05
Ccdc69	10.1954	9.66E-05

Ccdc69	10.1954	9.66E-05
Ccl25	10.1954	9.66E-05
Ccl25	10.7126	7.93E-05
Cd200r1	10.1954	9.66E-05
Cd200r1	10.1954	9.66E-05
Cd72	10.7126	7.93E-05
Cd72	10.7126	7.93E-05
Cdc6	10.1954	9.66E-05
Cdc6	10.1954	9.66E-05
Cdc6	10.1954	9.66E-05
Cdc6	10.7126	7.93E-05
Cdc6	10.7126	7.93E-05
Cdc6	10.1954	9.66E-05
Cdc6	10.1954	9.66E-05
Cdc6	10.1954	9.66E-05
Cdc6	10.7126	7.93E-05
Cdc6	10.7126	7.93E-05
Cdk1	10.7126	7.93E-05
Cdk10	10.7126	7.93E-05
Cdk10	10.7126	7.93E-05
Cdk10	10.7126	7.93E-05
Cdk2ap2	10.1954	9.66E-05
Cdk2ap2	10.1954	9.66E-05
Cdk2ap2	10.1954	9.66E-05
Cdk8	10.7126	7.93E-05
Cdk8	10.7126	7.93E-05
Cdk8	10.7126	7.93E-05
Cdk8	10.7126	7.93E-05
Cdk9	10.7126	7.93E-05
Cdk9	10.7126	7.93E-05
Cdk9	10.7126	7.93E-05
Cdk9	10.7126	7.93E-05
Cdk9	10.7126	7.93E-05
Cdk9	10.7126	7.93E-05
Cdyl	10.1954	9.66E-05
Cdyl	10.7126	7.93E-05
Cdyl	10.7126	7.93E-05
Ceacam1	10.1954	9.66E-05
Ceacam1	10.1954	9.66E-05
Ceacam1	10.7126	7.93E-05
Ceacam1	10.7126	7.93E-05
Cecr2	10.7126	7.93E-05
Celf1	10.1954	9.66E-05
Celf1	10.1954	9.66E-05
Celf2	10.1954	9.66E-05
Celf2	10.1954	9.66E-05
Celf2	10.7126	7.93E-05
Celf2	10.7126	7.93E-05
Celf2	10.7126	7.93E-05
Celf2	10.7126	7.93E-05
Celf2	10.7126	7.93E-05
Cenpi	10.1954	9.66E-05
Cenpi	10.7126	7.93E-05
Cep162	10.1954	9.66E-05
Cep162	10.7126	7.93E-05
Cep162	10.7126	7.93E-05
Cers6	10.1954	9.66E-05
Cers6	10.7126	7.93E-05

Cers6	10.7126	7.93E-05
Cetn4	10.1954	9.66E-05
Cetn4	10.1954	9.66E-05
Cetn4	10.1954	9.66E-05
Cetn4	10.7126	7.93E-05
Cfap157	10.1954	9.66E-05
Cfap157	10.1954	9.66E-05
Cfap157	10.7126	7.93E-05
Cfap157	10.7126	7.93E-05
Cfap157	10.1954	9.66E-05
Cfap157	10.1954	9.66E-05
Cfap157	10.7126	7.93E-05
Cfap157	10.7126	7.93E-05
Cfdp1	10.7126	7.93E-05
CH36-17G18.1	10.1954	9.66E-05
CH36-17G18.1	10.1954	9.66E-05
Chchd2	10.7126	7.93E-05
Chd9	10.1954	9.66E-05
Chn1	10.1954	9.66E-05
Chn1	10.7126	7.93E-05
Chn2	10.1954	9.66E-05
Chtf8	10.1954	9.66E-05
Chuk	10.1954	9.66E-05
Chuk	10.1954	9.66E-05
Clcc1	10.7126	7.93E-05
Cldn34c1	10.1954	9.66E-05
Cldn34c1	10.1954	9.66E-05
Cldn34c1	10.1954	9.66E-05
Cldn34c1	10.7126	7.93E-05
Cldn34c1	10.1954	9.66E-05
Cldn34c1	10.1954	9.66E-05
Cldn34c1	10.1954	9.66E-05
Cldn34c1	10.7126	7.93E-05
Clhc1	10.1954	9.66E-05
Clhc1	10.1954	9.66E-05
Clhc1	10.1954	9.66E-05
Clhc1	10.1954	9.66E-05
Clhc1	10.1954	9.66E-05
Clhc1	10.1954	9.66E-05
Clhc1	10.7126	7.93E-05
Clhc1	10.7126	7.93E-05
Clhc1	10.7126	7.93E-05
Clk2	10.1954	9.66E-05
Clk2	10.1954	9.66E-05
Clk2	10.1954	9.66E-05
Clk2	10.1954	9.66E-05
Clk2	10.1954	9.66E-05
Cltc	10.7126	7.93E-05
Cltc	10.7126	7.93E-05
Clybl	10.1954	9.66E-05
Clybl	10.1954	9.66E-05
Clybl	10.1954	9.66E-05
Clybl	10.1954	9.66E-05
Clybl	10.1954	9.66E-05
Clybl	10.1954	9.66E-05
Clybl	10.1954	9.66E-05
Clybl	10.1954	9.66E-05
Clybl	10.1954	9.66E-05

Clybl	10.7126	7.93E-05
Clybl	10.7126	7.93E-05
Clybl	10.7126	7.93E-05
Clybl	10.7126	7.93E-05
Clybl	10.7126	7.93E-05
Clybl	10.1954	9.66E-05
Clybl	10.1954	9.66E-05
Clybl	10.7126	7.93E-05
Clybl	10.7126	7.93E-05
Cnbp	10.1954	9.66E-05
Cnbp	10.1954	9.66E-05
Cnot1	10.1954	9.66E-05
Cnot2	10.1954	9.66E-05
Cnot2	10.1954	9.66E-05
Cnot2	10.1954	9.66E-05
Cnot6	10.7126	7.93E-05
Cog7	10.7126	7.93E-05
Col4a3bp	10.1954	9.66E-05
Cope	10.1954	9.66E-05
Copg1	10.7126	7.93E-05
Cox18	10.1954	9.66E-05
Cox18	10.1954	9.66E-05
Cox18	10.1954	9.66E-05
Cpsf4	10.1954	9.66E-05
Cpsf4	10.1954	9.66E-05
Cpsf4l	10.1954	9.66E-05
Cpsf4l	10.1954	9.66E-05
Cpsf4l	10.1954	9.66E-05
Cpsf6	10.7126	7.93E-05
Cpsf6	10.7126	7.93E-05
Csad	10.7126	7.93E-05
Csf1	10.7126	7.93E-05
Csmd3	10.1954	9.66E-05
Csmd3	10.7126	7.93E-05
Csmd3	10.7126	7.93E-05
Csmd3	10.1954	9.66E-05
Csmd3	10.7126	7.93E-05
Csmd3	10.7126	7.93E-05
Ctla2b	10.7126	7.93E-05
Ctla2b	10.7126	7.93E-05
Ctla2b	10.7126	7.93E-05
Ctla2b	10.7126	7.93E-05
Ctps	10.1954	9.66E-05
Ctps	10.7126	7.93E-05
Cwc22	10.1954	9.66E-05
Cwc22	10.1954	9.66E-05
Cwc22	10.1954	9.66E-05
Cwc22	10.1954	9.66E-05
Cyb5a	10.1954	9.66E-05
Cyba	10.7126	7.93E-05
Dcaf8	10.1954	9.66E-05
Dcun1d2	10.1954	9.66E-05
Dcun1d2	10.1954	9.66E-05
Dcun1d2	10.7126	7.93E-05
Dcun1d2	10.7126	7.93E-05
Dcun1d2	10.7126	7.93E-05
Ddx1	10.1954	9.66E-05
Ddx1	10.1954	9.66E-05

Ddx1	10.1954	9.66E-05
Ddx1	10.1954	9.66E-05
Ddx1	10.1954	9.66E-05
Ddx1	10.1954	9.66E-05
Ddx43	10.1954	9.66E-05
Ddx43	10.7126	7.93E-05
Ddx5	10.1954	9.66E-05
Ddx60	10.1954	9.66E-05
Ddx60	10.1954	9.66E-05
Decr2	10.7126	7.93E-05
Dgke	10.1954	9.66E-05
Dhdh	10.1954	9.66E-05
Dhps	10.7126	7.93E-05
Dhx40	10.1954	9.66E-05
Dhx40	10.1954	9.66E-05
Dimt1	10.1954	9.66E-05
Dimt1	10.7126	7.93E-05
Dip2c	10.1954	9.66E-05
Dip2c	10.7126	7.93E-05
Dleu2	10.7126	7.93E-05
Dnah2	10.1954	9.66E-05
Dnah2	10.1954	9.66E-05
Dnah2	10.7126	7.93E-05
Dnah2	10.7126	7.93E-05
Dnajb13	10.1954	9.66E-05
Dnajb14	10.1954	9.66E-05
Dnajb14	10.1954	9.66E-05
Dnajb14	10.1954	9.66E-05
Dnajb14	10.1954	9.66E-05
Dnajb14	10.7126	7.93E-05
Dnajc11	10.1954	9.66E-05
Dnajc11	10.1954	9.66E-05
Dnajc11	10.7126	7.93E-05
Dnajc11	10.7126	7.93E-05
Dnajc11	10.7126	7.93E-05
Dnajc25	10.1954	9.66E-05
Dnajc25	10.1954	9.66E-05
Dnmt3l	10.1954	9.66E-05
Dnmt3l	10.1954	9.66E-05
Dnmt3l	10.1954	9.66E-05
Dnmt3l	10.1954	9.66E-05
Dnmt3l	10.1954	9.66E-05
Dnmt3l	10.1954	9.66E-05
Dnmt3l	10.1954	9.66E-05
Dnmt3l	10.1954	9.66E-05
Dnmt3l	10.1954	9.66E-05
Dnmt3l	10.1954	9.66E-05
Dnmt3l	10.1954	9.66E-05
Dnmt3l	10.1954	9.66E-05
Dnmt3l	10.1954	9.66E-05
Dnmt3l	10.1954	9.66E-05
Dnmt3l	10.1954	9.66E-05
Dnmt3l	10.7126	7.93E-05
Dnmt3l	10.7126	7.93E-05
Dock10	10.1954	9.66E-05
Dock10	10.7126	7.93E-05
Dock10	10.7126	7.93E-05
Dock10	10.7126	7.93E-05

Dph3	10.1954	9.66E-05
Dph3	10.1954	9.66E-05
Dph3	10.1954	9.66E-05
Dph3	10.1954	9.66E-05
Dph3	10.1954	9.66E-05
Dph3	10.1954	9.66E-05
Dph3	10.1954	9.66E-05
Dph3	10.1954	9.66E-05
Dph3	10.1954	9.66E-05
Dph3	10.1954	9.66E-05
Dph3	10.1954	9.66E-05
Dph3	10.1954	9.66E-05
Dpm1	10.1954	9.66E-05
Dpm1	10.1954	9.66E-05
Dpm1	10.7126	7.93E-05
Dpm1	10.7126	7.93E-05
Dpp7	10.1954	9.66E-05
Dpp7	10.7126	7.93E-05
Drg1	10.1954	9.66E-05
Drg1	10.1954	9.66E-05
Drg1	10.1954	9.66E-05
Drg1	10.1954	9.66E-05
Drg1	10.7126	7.93E-05
Drg1	10.7126	7.93E-05
Drg1	10.7126	7.93E-05
Drg1	10.1954	9.66E-05
Drg1	10.1954	9.66E-05
Drg1	10.1954	9.66E-05
Dscc1	10.1954	9.66E-05
Dscc1	10.1954	9.66E-05
Dscc1	10.1954	9.66E-05
Dst	10.1954	9.66E-05
Dst	10.1954	9.66E-05
Dtl	10.1954	9.66E-05
Dus2	10.1954	9.66E-05
Dus2	10.7126	7.93E-05
Dusp12	10.7126	7.93E-05
Dusp12	10.7126	7.93E-05
Dusp22	10.1954	9.66E-05
Dusp22	10.1954	9.66E-05
Dusp22	10.7126	7.93E-05
Dusp22	10.7126	7.93E-05
Dusp22	10.7126	7.93E-05
Dydc1	10.1954	9.66E-05
Dydc1	10.1954	9.66E-05
Ednrb	10.1954	9.66E-05
Edrf1	10.1954	9.66E-05
Edrf1	10.1954	9.66E-05
Edrf1	10.7126	7.93E-05
Eed	10.1954	9.66E-05
Eed	10.1954	9.66E-05
Eed	10.1954	9.66E-05
Eed	10.7126	7.93E-05
Eed	10.1954	9.66E-05
Eed	10.1954	9.66E-05
Eed	10.1954	9.66E-05
Eed	10.1954	9.66E-05
Eed	10.1954	9.66E-05

Eed	10.7126	7.93E-05
Eef1akmt1	10.1954	9.66E-05
Eef1akmt1	10.1954	9.66E-05
Eef1akmt1	10.7126	7.93E-05
Eef1d	10.7126	7.93E-05
Eef1kmt4	10.1954	9.66E-05
Eef1kmt4	10.7126	7.93E-05
Eef1kmt4	10.7126	7.93E-05
Eif4e1b	10.1954	9.66E-05
Eif4g3	10.1954	9.66E-05
Eif4g3	10.7126	7.93E-05
Eif5	10.1954	9.66E-05
Eif5	10.1954	9.66E-05
Eif5	10.1954	9.66E-05
Eif5	10.1954	9.66E-05
Eif5	10.7126	7.93E-05
Eif5	10.7126	7.93E-05
Eif5	10.1954	9.66E-05
Eif5	10.1954	9.66E-05
Eif5	10.1954	9.66E-05
Eif5	10.1954	9.66E-05
Eif5	10.7126	7.93E-05
Eif5	10.7126	7.93E-05
Elk4	10.7126	7.93E-05
Elovl3	10.7126	7.93E-05
Elovl3	10.7126	7.93E-05
Eml3	10.7126	7.93E-05
Enox2	10.1954	9.66E-05
Enox2	10.1954	9.66E-05
Enox2	10.7126	7.93E-05
Enox2	10.7126	7.93E-05
Enox2	10.7126	7.93E-05
Enox2	10.7126	7.93E-05
Epb41l3	10.1954	9.66E-05
Epb41l4a	10.7126	7.93E-05
Epb41l4a	10.7126	7.93E-05
Epn2	10.1954	9.66E-05
Epn2	10.1954	9.66E-05
Epn2	10.7126	7.93E-05
Epn2	10.7126	7.93E-05
Epn2	10.1954	9.66E-05
Epn2	10.1954	9.66E-05
Epn2	10.7126	7.93E-05
Epn2	10.7126	7.93E-05
Erc1	10.7126	7.93E-05
Erc1	10.7126	7.93E-05
Ercc6	10.1954	9.66E-05
Ercc6	10.7126	7.93E-05
Ergic2	10.1954	9.66E-05
Ergic2	10.1954	9.66E-05
Ewsr1	10.1954	9.66E-05
Ewsr1	10.7126	7.93E-05
Ewsr1	10.7126	7.93E-05
Fam118b	10.1954	9.66E-05
Fam129c	10.1954	9.66E-05
Fam129c	10.1954	9.66E-05
Fam129c	10.7126	7.93E-05
Fam129c	10.7126	7.93E-05

Fam134a	10.7126	7.93E-05
Fam134c	10.1954	9.66E-05
Fam134c	10.7126	7.93E-05
Fam134c	10.7126	7.93E-05
Fam135a	10.1954	9.66E-05
Fam135a	10.7126	7.93E-05
Fam135a	10.1954	9.66E-05
Fam155a	10.1954	9.66E-05
Fam155a	10.1954	9.66E-05
Fam169b	10.1954	9.66E-05
Fam169b	10.7126	7.93E-05
Fam169b	10.1954	9.66E-05
Fam172a	10.1954	9.66E-05
Fam172a	10.7126	7.93E-05
Fam173b	10.1954	9.66E-05
Fam178b	10.7126	7.93E-05
Fam179b	10.1954	9.66E-05
Fam179b	10.1954	9.66E-05
Fam179b	10.7126	7.93E-05
Fam184a	10.1954	9.66E-05
Fam184a	10.7126	7.93E-05
Fam184a	10.1954	9.66E-05
Fam184a	10.7126	7.93E-05
Fam188a	10.1954	9.66E-05
Fam188a	10.1954	9.66E-05
Fam188a	10.7126	7.93E-05
Fam188a	10.7126	7.93E-05
Fam188a	10.1954	9.66E-05
Fam188a	10.7126	7.93E-05
Fam188a	10.7126	7.93E-05
Fam192a	10.1954	9.66E-05
Fam192a	10.1954	9.66E-05
Fam192a	10.1954	9.66E-05
Fam192a	10.1954	9.66E-05
Fam214a	10.1954	9.66E-05
Fam49a	10.1954	9.66E-05
Fam49a	10.7126	7.93E-05
Fam49a	10.7126	7.93E-05
Fam49a	10.7126	7.93E-05
Far1	10.1954	9.66E-05
Far1	10.1954	9.66E-05
Far1	10.7126	7.93E-05
Farsa	10.7126	7.93E-05
Farsb	10.1954	9.66E-05
Farsb	10.1954	9.66E-05
Farsb	10.7126	7.93E-05
Fastkd2	10.1954	9.66E-05
Fastkd2	10.1954	9.66E-05
Fastkd2	10.1954	9.66E-05
Fastkd2	10.1954	9.66E-05
Faxc	10.1954	9.66E-05
Fbxl3	10.1954	9.66E-05
Fbxo34	10.1954	9.66E-05
Fbxo47	10.1954	9.66E-05
Fbxo47	10.1954	9.66E-05
Fbxo47	10.1954	9.66E-05
Fbxo47	10.1954	9.66E-05
Fbxw18	10.1954	9.66E-05

Fbxw18	10.7126	7.93E-05
Fbxw18	10.1954	9.66E-05
Fbxw18	10.1954	9.66E-05
Fbxw18	10.7126	7.93E-05
Fbxw18	10.7126	7.93E-05
Fbxw27	10.7126	7.93E-05
Fbxw27	10.7126	7.93E-05
Fbxw9	10.1954	9.66E-05
Fkbp1a	10.7126	7.93E-05
Fkbp3	10.1954	9.66E-05
Fkbp3	10.7126	7.93E-05
Fkbp3	10.7126	7.93E-05
Flad1	10.7126	7.93E-05
Flt3l	10.1954	9.66E-05
Flt3l	10.1954	9.66E-05
Flt3l	10.7126	7.93E-05
Flt3l	10.7126	7.93E-05
Flt3l	10.7126	7.93E-05
Flt3l	10.7126	7.93E-05
Fmr1nb	10.1954	9.66E-05
Fmr1nb	10.1954	9.66E-05
Fmr1nb	10.1954	9.66E-05
Fmr1nb	10.1954	9.66E-05
Fmr1nb	10.1954	9.66E-05
Fmr1nb	10.1954	9.66E-05
Fmr1nb	10.7126	7.93E-05
Fmr1nb	10.7126	7.93E-05
Fmr1nb	10.1954	9.66E-05
Fmr1nb	10.1954	9.66E-05
Fmr1nb	10.1954	9.66E-05
Fnip1	10.1954	9.66E-05
Fnip1	10.7126	7.93E-05
Foxn3	10.1954	9.66E-05
Foxn3	10.1954	9.66E-05
Foxn3	10.1954	9.66E-05
Foxn3	10.7126	7.93E-05
Foxn3	10.7126	7.93E-05
Foxn3	10.7126	7.93E-05
Foxred1	10.1954	9.66E-05
Fxr1	10.1954	9.66E-05
Fxr1	10.1954	9.66E-05
Fxr1	10.1954	9.66E-05
Fxr1	10.7126	7.93E-05
Fxyd4	10.1954	9.66E-05
Fxyd4	10.1954	9.66E-05
Fxyd4	10.1954	9.66E-05
Fxyd4	10.7126	7.93E-05
Fxyd4	10.7126	7.93E-05
Fyttd1	10.1954	9.66E-05
Fyttd1	10.1954	9.66E-05
G6pc3	10.7126	7.93E-05
G6pc3	10.7126	7.93E-05
Gabpb1	10.1954	9.66E-05
Gabpb1	10.1954	9.66E-05
Gabpb1	10.1954	9.66E-05
Gabpb1	10.1954	9.66E-05
Gabpb1	10.7126	7.93E-05
Gal	10.7126	7.93E-05

Gas8	10.7126	7.93E-05
Gcdh	10.1954	9.66E-05
Gcdh	10.1954	9.66E-05
Gcdh	10.1954	9.66E-05
Gcdh	10.1954	9.66E-05
Gcdh	10.7126	7.93E-05
Gckr	10.1954	9.66E-05
Gclc	10.1954	9.66E-05
Gemin8	10.1954	9.66E-05
Gemin8	10.1954	9.66E-05
Gemin8	10.1954	9.66E-05
Gemin8	10.1954	9.66E-05
Gemin8	10.1954	9.66E-05
Gemin8	10.1954	9.66E-05
Ggps1	10.1954	9.66E-05
Ggps1	10.7126	7.93E-05
Ggps1	10.1954	9.66E-05
Ggps1	10.7126	7.93E-05
Ggta1	10.7126	7.93E-05
Ggta1	10.7126	7.93E-05
Git2	10.7126	7.93E-05
Glcci1	10.1954	9.66E-05
Gltscr2	10.7126	7.93E-05
Gltscr2	10.7126	7.93E-05
Gltscr2	10.7126	7.93E-05
Glyr1	10.1954	9.66E-05
Glyr1	10.7126	7.93E-05
Gm21411	10.1954	9.66E-05
Gm21411	10.1954	9.66E-05
Gm21411	10.7126	7.93E-05
Gm21411	10.7126	7.93E-05
Gm21411	10.7126	7.93E-05
Gm21411	10.7126	7.93E-05
Gm21411	10.7126	7.93E-05
Gm815	10.1954	9.66E-05
Gm815	10.1954	9.66E-05
Gnl3	10.1954	9.66E-05
Gnl3	10.1954	9.66E-05
Gnl3	10.7126	7.93E-05
Gnpnat1	10.7126	7.93E-05
Golga1	10.1954	9.66E-05
Golga1	10.1954	9.66E-05
Golga1	10.1954	9.66E-05
Golga1	10.1954	9.66E-05
Golga1	10.7126	7.93E-05
Gpat2	10.7126	7.93E-05
Gpatch2l	10.1954	9.66E-05
Gpatch2l	10.7126	7.93E-05
Gpbp1	10.7126	7.93E-05
Gpcpd1	10.1954	9.66E-05
Gpcpd1	10.1954	9.66E-05
Gpcpd1	10.1954	9.66E-05
Gpcpd1	10.7126	7.93E-05
Gpcpd1	10.7126	7.93E-05
Gpcpd1	10.1954	9.66E-05
Gpcpd1	10.1954	9.66E-05
Gpcpd1	10.7126	7.93E-05
Gpcpd1	10.7126	7.93E-05

Gpd1l	10.1954	9.66E-05
Gpd1l	10.1954	9.66E-05
Gpd1l	10.1954	9.66E-05
Gpd1l	10.1954	9.66E-05
Gpd1l	10.1954	9.66E-05
Gpn3	10.7126	7.93E-05
Gpr19	10.7126	7.93E-05
Gpr19	10.7126	7.93E-05
Gpr19	10.7126	7.93E-05
Gpr19	10.7126	7.93E-05
Gpx4	10.7126	7.93E-05
Gpx4	10.7126	7.93E-05
Gpx4	10.7126	7.93E-05
Gpx4	10.7126	7.93E-05
Gramd2	10.1954	9.66E-05
Gstm1	10.7126	7.93E-05
Gtf2f2	10.1954	9.66E-05
Gtf2f2	10.1954	9.66E-05
Gtf2f2	10.1954	9.66E-05
Gtf2f2	10.1954	9.66E-05
Gtf2f2	10.1954	9.66E-05
Gtf2f2	10.1954	9.66E-05
Guf1	10.1954	9.66E-05
Guf1	10.7126	7.93E-05
H2-K1	10.7126	7.93E-05
H2-K1	10.7126	7.93E-05
H2-K1	10.7126	7.93E-05
H2-K1	10.7126	7.93E-05
H2-K1	10.7126	7.93E-05
H2-K1	10.7126	7.93E-05
H2-K1	10.7126	7.93E-05
H2-K1	10.7126	7.93E-05
H2-K1	10.7126	7.93E-05
H2-K1	10.7126	7.93E-05
H2-K1	10.7126	7.93E-05
H2-K1	10.7126	7.93E-05
H2-K1	10.7126	7.93E-05
H2-K1	10.7126	7.93E-05
H2-K1	10.7126	7.93E-05
H2-Q7	10.7126	7.93E-05
Hars2	10.1954	9.66E-05
Hars2	10.7126	7.93E-05
Hdlbp	10.1954	9.66E-05
Hdlbp	10.1954	9.66E-05
Heatr5b	10.1954	9.66E-05
Heatr5b	10.7126	7.93E-05
Hectd2	10.7126	7.93E-05
Hells	10.1954	9.66E-05
Hells	10.1954	9.66E-05
Hells	10.1954	9.66E-05
Helz	10.1954	9.66E-05
Hira	10.1954	9.66E-05
Hmbs	10.1954	9.66E-05
Hmbs	10.1954	9.66E-05
Hmbs	10.1954	9.66E-05
Hnrnpa3	10.1954	9.66E-05
Hnrnpa3	10.1954	9.66E-05
Hnrnpa3	10.7126	7.93E-05

Hnrnpc	10.1954	9.66E-05
Hnrnpc	10.1954	9.66E-05
Hnrnpc	10.1954	9.66E-05
Hnrnpc	10.1954	9.66E-05
Hnrnpc	10.1954	9.66E-05
Hnrnpc	10.1954	9.66E-05
Hnrnpc	10.7126	7.93E-05
Hnrnpc	10.7126	7.93E-05
Hnrnpc	10.1954	9.66E-05
Hnrnpc	10.1954	9.66E-05
Hnrnpc	10.1954	9.66E-05
Hnrnpc	10.1954	9.66E-05
Hnrnpc	10.1954	9.66E-05
Hnrnpc	10.1954	9.66E-05
Hnrnpc	10.7126	7.93E-05
Hnrnpc	10.7126	7.93E-05
Hnrnpd	10.1954	9.66E-05
Huwe1	10.7126	7.93E-05
Huwe1	10.7126	7.93E-05
Iars	10.7126	7.93E-05
Iars	10.7126	7.93E-05
Ice2	10.7126	7.93E-05
Ice2	10.1954	9.66E-05
Ice2	10.1954	9.66E-05
Ice2	10.7126	7.93E-05
Ice2	10.7126	7.93E-05
Ice2	10.7126	7.93E-05
Ice2	10.7126	7.93E-05
Ice2	10.7126	7.93E-05
Ifi27	10.1954	9.66E-05
Ifi27	10.1954	9.66E-05
Ifi27	10.7126	7.93E-05
Ifi27	10.1954	9.66E-05
Ifi27	10.1954	9.66E-05
Ifi27	10.7126	7.93E-05
Ift20	10.1954	9.66E-05
Ift20	10.1954	9.66E-05
Ift20	10.7126	7.93E-05
Ift20	10.7126	7.93E-05
Igf2bp2	10.1954	9.66E-05
Igf2bp2	10.1954	9.66E-05
Igf2bp2	10.1954	9.66E-05
Igf2bp2	10.1954	9.66E-05
Il17f	10.1954	9.66E-05
Il17f	10.1954	9.66E-05
Il17f	10.1954	9.66E-05
Il17f	10.1954	9.66E-05
Il17f	10.1954	9.66E-05
Il17f	10.7126	7.93E-05
Il17f	10.7126	7.93E-05
Il17f	10.7126	7.93E-05
Il17f	10.7126	7.93E-05
Il17f	10.7126	7.93E-05
Ilf2	10.1954	9.66E-05
Ilf3	10.1954	9.66E-05
Ilf3	10.7126	7.93E-05
Ilf3	10.7126	7.93E-05
Ilf3	10.7126	7.93E-05

Ilkap	10.1954	9.66E-05
Immt	10.1954	9.66E-05
Immt	10.1954	9.66E-05
Immt	10.1954	9.66E-05
Immt	10.1954	9.66E-05
Immt	10.1954	9.66E-05
Inip	10.1954	9.66E-05
Inip	10.1954	9.66E-05
Ipcef1	10.1954	9.66E-05
Ipcef1	10.1954	9.66E-05
Ipcef1	10.1954	9.66E-05
Ipcef1	10.1954	9.66E-05
Ipcef1	10.1954	9.66E-05
Ipcef1	10.7126	7.93E-05
Ipcef1	10.7126	7.93E-05
Ipcef1	10.7126	7.93E-05
Ipmk	10.7126	7.93E-05
Ipmk	10.7126	7.93E-05
Ipp	10.1954	9.66E-05
Ist1	10.1954	9.66E-05
Ist1	10.1954	9.66E-05
Ist1	10.1954	9.66E-05
Ist1	10.1954	9.66E-05
Ist1	10.1954	9.66E-05
Ist1	10.1954	9.66E-05
Ist1	10.1954	9.66E-05
Ist1	10.7126	7.93E-05
Ist1	10.7126	7.93E-05
Iws1	10.1954	9.66E-05
Iws1	10.1954	9.66E-05
Iws1	10.1954	9.66E-05
Iws1	10.1954	9.66E-05
Iws1	10.7126	7.93E-05
Iws1	10.1954	9.66E-05
Iws1	10.1954	9.66E-05
Iws1	10.1954	9.66E-05
Iws1	10.1954	9.66E-05
Iws1	10.7126	7.93E-05
Jmjd6	10.7126	7.93E-05
Jmjd6	10.7126	7.93E-05
Kcnu1	10.7126	7.93E-05
Kctd20	10.1954	9.66E-05
Kctd9	10.1954	9.66E-05
Kctd9	10.1954	9.66E-05
Kif16b	10.1954	9.66E-05
Kif16b	10.7126	7.93E-05
Kif16b	10.7126	7.93E-05
Kif18a	10.1954	9.66E-05
Kif18a	10.1954	9.66E-05
Kif18a	10.1954	9.66E-05
Kif18a	10.1954	9.66E-05
Kif18a	10.1954	9.66E-05
Kif18a	10.1954	9.66E-05
Kif18a	10.7126	7.93E-05
Kif18a	10.7126	7.93E-05
Kif18a	10.7126	7.93E-05
Kif18a	10.7126	7.93E-05
Kif18a	10.7126	7.93E-05

Kif18a	10.7126	7.93E-05
Kiz	10.1954	9.66E-05
Kiz	10.1954	9.66E-05
Kiz	10.1954	9.66E-05
Klf17	10.1954	9.66E-05
Klf17	10.1954	9.66E-05
Klf7	10.1954	9.66E-05
Klf7	10.7126	7.93E-05
Klf7	10.1954	9.66E-05
Klf7	10.7126	7.93E-05
Klhl15	10.1954	9.66E-05
Klhl15	10.1954	9.66E-05
Klhl15	10.1954	9.66E-05
Klhl15	10.1954	9.66E-05
Klhl15	10.1954	9.66E-05
Klhl15	10.1954	9.66E-05
Klhl15	10.1954	9.66E-05
Klhl15	10.1954	9.66E-05
Klhl15	10.1954	9.66E-05
Klhl15	10.1954	9.66E-05
Klhl15	10.1954	9.66E-05
Klhl15	10.1954	9.66E-05
Klhl15	10.7126	7.93E-05
Klhl15	10.7126	7.93E-05
Klhl15	10.7126	7.93E-05
Kmt2e	10.1954	9.66E-05
Kmt2e	10.1954	9.66E-05
Kmt2e	10.1954	9.66E-05
Kmt2e	10.1954	9.66E-05
Kntc1	10.1954	9.66E-05
Kntc1	10.7126	7.93E-05
Kxd1	10.1954	9.66E-05
Lgals9	10.7126	7.93E-05
Lilra6	10.7126	7.93E-05
Lilra6	10.7126	7.93E-05
Lilra6	10.7126	7.93E-05
Lman2l	10.1954	9.66E-05
Lman2l	10.1954	9.66E-05
Lman2l	10.1954	9.66E-05
Lman2l	10.1954	9.66E-05
Lman2l	10.7126	7.93E-05
Lman2l	10.7126	7.93E-05
Lman2l	10.7126	7.93E-05
Lman2l	10.1954	9.66E-05
Lman2l	10.1954	9.66E-05
Lman2l	10.1954	9.66E-05
Lman2l	10.1954	9.66E-05
Lman2l	10.1954	9.66E-05
Lman2l	10.1954	9.66E-05
Lman2l	10.1954	9.66E-05
Lman2l	10.1954	9.66E-05
Lman2l	10.7126	7.93E-05
Lman2l	10.7126	7.93E-05
Lman2l	10.7126	7.93E-05
Lnpk	10.1954	9.66E-05
Lnpk	10.1954	9.66E-05
Lnpk	10.1954	9.66E-05
Lnpk	10.1954	9.66E-05

Lnpk	10.1954	9.66E-05
Lnpk	10.7126	7.93E-05
Lnpk	10.7126	7.93E-05
Lnpk	10.7126	7.93E-05
Lnpk	10.7126	7.93E-05
Lnpk	10.7126	7.93E-05
Lnpk	10.7126	7.93E-05
Lnpk	10.7126	7.93E-05
Lnpk	10.7126	7.93E-05
Lnpk	10.7126	7.93E-05
Loxhd1	10.7126	7.93E-05
Loxhd1	10.7126	7.93E-05
Lrch3	10.1954	9.66E-05
Lrch3	10.7126	7.93E-05
Lrguk	10.7126	7.93E-05
Lrrc2	10.7126	7.93E-05
Lrrc28	10.1954	9.66E-05
Lrrc6	10.7126	7.93E-05
Lrrc6	10.7126	7.93E-05
Lrrc6	10.7126	7.93E-05
Lrrfip1	10.1954	9.66E-05
Lsm6	10.1954	9.66E-05
Lta4h	10.1954	9.66E-05
Lta4h	10.7126	7.93E-05
Lta4h	10.7126	7.93E-05
Lta4h	10.7126	7.93E-05
Luc7l2	10.1954	9.66E-05
Luc7l2	10.1954	9.66E-05
Luc7l2	10.1954	9.66E-05
Luc7l2	10.7126	7.93E-05
Luc7l2	10.7126	7.93E-05
Luc7l2	10.7126	7.93E-05
Luc7l3	10.1954	9.66E-05
Luc7l3	10.1954	9.66E-05
Luc7l3	10.1954	9.66E-05
Luc7l3	10.1954	9.66E-05
Luc7l3	10.7126	7.93E-05
Lyrm1	10.1954	9.66E-05
Lyrm1	10.1954	9.66E-05
Lyrm1	10.7126	7.93E-05
Lyrm1	10.7126	7.93E-05
Macf1	10.1954	9.66E-05
Macf1	10.1954	9.66E-05
Mad1l1	10.7126	7.93E-05
Mad2l2	10.7126	7.93E-05
Magi3	10.1954	9.66E-05
Magi3	10.1954	9.66E-05
Magi3	10.7126	7.93E-05
Man1b1	10.7126	7.93E-05
Map3k7	10.1954	9.66E-05
Map3k7	10.7126	7.93E-05
Map3k7	10.7126	7.93E-05
Map4k5	10.1954	9.66E-05
Map4k5	10.1954	9.66E-05
Map4k5	10.1954	9.66E-05
Map4k5	10.1954	9.66E-05
Map4k5	10.1954	9.66E-05
Map4k5	10.1954	9.66E-05

Map4k5	10.1954	9.66E-05
Map4k5	10.1954	9.66E-05
Map4k5	10.1954	9.66E-05
Map4k5	10.1954	9.66E-05
Map4k5	10.1954	9.66E-05
Map7d2	10.1954	9.66E-05
Map7d2	10.1954	9.66E-05
Map7d2	10.1954	9.66E-05
Map7d2	10.1954	9.66E-05
Map7d2	10.7126	7.93E-05
Masp2	10.1954	9.66E-05
Masp2	10.1954	9.66E-05
Matr3	10.1954	9.66E-05
Matr3	10.1954	9.66E-05
Matr3	10.1954	9.66E-05
Matr3	10.1954	9.66E-05
Matr3	10.7126	7.93E-05
Matr3	10.7126	7.93E-05
Mbtd1	10.1954	9.66E-05
Mbtd1	10.1954	9.66E-05
Mcoln1	10.1954	9.66E-05
Mcoln1	10.1954	9.66E-05
Mcph1	10.1954	9.66E-05
Med12l	10.1954	9.66E-05
Med12l	10.1954	9.66E-05
Med12l	10.1954	9.66E-05
Med12l	10.1954	9.66E-05
Med13	10.7126	7.93E-05
Med15	10.1954	9.66E-05
Med15	10.1954	9.66E-05
Med27	10.1954	9.66E-05
Med27	10.1954	9.66E-05
Med27	10.1954	9.66E-05
Med27	10.1954	9.66E-05
Med27	10.1954	9.66E-05
Med27	10.1954	9.66E-05
Med27	10.1954	9.66E-05
Med27	10.7126	7.93E-05
Med27	10.7126	7.93E-05
Meg3	10.1954	9.66E-05
Metap2	10.1954	9.66E-05
Metap2	10.1954	9.66E-05
Metap2	10.1954	9.66E-05
Metap2	10.1954	9.66E-05
Metap2	10.7126	7.93E-05
Metap2	10.7126	7.93E-05
Mettl23	10.7126	7.93E-05
Mettl23	10.7126	7.93E-05
Mfsd4	10.7126	7.93E-05
Mia	10.1954	9.66E-05
Mia3	10.1954	9.66E-05
Milr1	10.1954	9.66E-05
Milr1	10.1954	9.66E-05
Milr1	10.1954	9.66E-05
Milr1	10.1954	9.66E-05
Milr1	10.1954	9.66E-05
Milr1	10.1954	9.66E-05
Milr1	10.1954	9.66E-05

Milr1	10.1954	9.66E-05
Milr1	10.1954	9.66E-05
Milr1	10.7126	7.93E-05
Milr1	10.7126	7.93E-05
Milr1	10.7126	7.93E-05
Minos1	10.7126	7.93E-05
Mkln1	10.1954	9.66E-05
Mkln1	10.1954	9.66E-05
Mkln1	10.1954	9.66E-05
Mkln1	10.7126	7.93E-05
Mkln1	10.7126	7.93E-05
Mkrn2	10.1954	9.66E-05
Mkrn2	10.1954	9.66E-05
Mlkl	10.1954	9.66E-05
Mlkl	10.1954	9.66E-05
Mlkl	10.1954	9.66E-05
Mlkl	10.1954	9.66E-05
Mllt10	10.1954	9.66E-05
Mllt10	10.1954	9.66E-05
Mllt10	10.1954	9.66E-05
Mllt10	10.1954	9.66E-05
Mllt10	10.1954	9.66E-05
Mllt10	10.1954	9.66E-05
Mllt10	10.7126	7.93E-05
Mnat1	10.1954	9.66E-05
Mnat1	10.1954	9.66E-05
Mnat1	10.7126	7.93E-05
Mnat1	10.1954	9.66E-05
Mnat1	10.1954	9.66E-05
Mnat1	10.7126	7.93E-05
Morf4l1	10.1954	9.66E-05
Mov10l1	10.7126	7.93E-05
Mpc1	10.1954	9.66E-05
Mpv17l2	10.1954	9.66E-05
Mpzl1	10.7126	7.93E-05
Mpzl1	10.7126	7.93E-05
Mroh8	10.7126	7.93E-05
Mrpl30	10.1954	9.66E-05
Mrpl30	10.1954	9.66E-05
Mrpl30	10.7126	7.93E-05
Mrpl30	10.7126	7.93E-05
Mrpl48	10.1954	9.66E-05
Mrpl48	10.1954	9.66E-05
Mrpl48	10.7126	7.93E-05
Mrpl48	10.7126	7.93E-05
Mrpl55	10.7126	7.93E-05
Mrps26	10.7126	7.93E-05
Msantd2	10.1954	9.66E-05
Msantd2	10.1954	9.66E-05
Msantd2	10.7126	7.93E-05
Msantd2	10.7126	7.93E-05
Msi2	10.1954	9.66E-05
Mtf2	10.1954	9.66E-05
Mtf2	10.1954	9.66E-05
Mtf2	10.1954	9.66E-05
Mtf2	10.1954	9.66E-05
Mtf2	10.1954	9.66E-05
Mtf2	10.1954	9.66E-05

Mtg2	10.1954	9.66E-05
Mtg2	10.7126	7.93E-05
Mtg2	10.7126	7.93E-05
Mtmr2	10.1954	9.66E-05
Mum1l1	10.7126	7.93E-05
Mum1l1	10.7126	7.93E-05
Mvk	10.7126	7.93E-05
Mvk	10.7126	7.93E-05
Mycbp2	10.1954	9.66E-05
Myef2	10.1954	9.66E-05
Myef2	10.1954	9.66E-05
Myef2	10.1954	9.66E-05
Myef2	10.1954	9.66E-05
Myef2	10.1954	9.66E-05
Myef2	10.1954	9.66E-05
Myef2	10.1954	9.66E-05
Myef2	10.1954	9.66E-05
Myef2	10.7126	7.93E-05
Myo1g	10.1954	9.66E-05
Myo1g	10.7126	7.93E-05
Myrfl	10.1954	9.66E-05
Myrfl	10.1954	9.66E-05
Myrfl	10.1954	9.66E-05
Myrfl	10.1954	9.66E-05
Myrfl	10.7126	7.93E-05
Myrfl	10.7126	7.93E-05
Myrfl	10.7126	7.93E-05
Myrfl	10.7126	7.93E-05
N4bp2	10.1954	9.66E-05
N4bp2	10.7126	7.93E-05
Napepld	10.1954	9.66E-05
Napepld	10.1954	9.66E-05
Napepld	10.1954	9.66E-05
Napepld	10.1954	9.66E-05
Napepld	10.1954	9.66E-05
Napepld	10.1954	9.66E-05
Napepld	10.1954	9.66E-05
Napepld	10.1954	9.66E-05
Napepld	10.1954	9.66E-05
Napepld	10.1954	9.66E-05
Napepld	10.1954	9.66E-05
Napepld	10.1954	9.66E-05
Napepld	10.1954	9.66E-05
Napepld	10.1954	9.66E-05
Nasp	10.1954	9.66E-05
Nasp	10.7126	7.93E-05
Nasp	10.1954	9.66E-05
Nasp	10.7126	7.93E-05
Nav2	10.1954	9.66E-05
Nav2	10.1954	9.66E-05
Ncapd3	10.1954	9.66E-05
Ncapd3	10.1954	9.66E-05
Ncapg	10.1954	9.66E-05
Ncapg	10.1954	9.66E-05
Ncapg	10.1954	9.66E-05
Ncapg	10.1954	9.66E-05
Ncapg	10.1954	9.66E-05
Ncapg	10.1954	9.66E-05

Ncapg	10.1954	9.66E-05
Ncapg	10.1954	9.66E-05
Ncapg	10.1954	9.66E-05
Ncapg	10.7126	7.93E-05
Ncapg	10.7126	7.93E-05
Ncapg	10.7126	7.93E-05
Ncapg	10.7126	7.93E-05
Ncapg	10.7126	7.93E-05
Ncapg	10.7126	7.93E-05
Ncapg	10.7126	7.93E-05
Ncapg	10.7126	7.93E-05
Ncapg	10.7126	7.93E-05
Ncapg	10.7126	7.93E-05
Ncapg	10.7126	7.93E-05
Ncapg	10.7126	7.93E-05
Nckap5	10.1954	9.66E-05
Nckap5	10.1954	9.66E-05
Ncoa1	10.1954	9.66E-05
Ncoa1	10.1954	9.66E-05
Ncor1	10.1954	9.66E-05
Ncor1	10.1954	9.66E-05
Ndufaf7	10.1954	9.66E-05
Ndufaf7	10.1954	9.66E-05
Ndufs1	10.1954	9.66E-05
Ndufs1	10.1954	9.66E-05
Ndufs4	10.1954	9.66E-05
Ndufs4	10.1954	9.66E-05
Ndufs4	10.1954	9.66E-05
Ndufs4	10.1954	9.66E-05
Ndufs4	10.1954	9.66E-05
Ndufv1	10.7126	7.93E-05
Nek3	10.1954	9.66E-05
Nek3	10.1954	9.66E-05
Nek3	10.1954	9.66E-05
Nek3	10.7126	7.93E-05
Nek4	10.1954	9.66E-05
Nek4	10.1954	9.66E-05
Nek4	10.7126	7.93E-05
Nek4	10.1954	9.66E-05
Nek4	10.1954	9.66E-05
Nek4	10.7126	7.93E-05
Nelfb	10.1954	9.66E-05
Nelfb	10.1954	9.66E-05
Nfat5	10.7126	7.93E-05
Nfat5	10.7126	7.93E-05
Nfat5	10.7126	7.93E-05
Nfyb	10.1954	9.66E-05
Nfyb	10.1954	9.66E-05
Nhsl1	10.1954	9.66E-05
Nhsl1	10.1954	9.66E-05
Nhsl1	10.7126	7.93E-05
Nid2	10.1954	9.66E-05
Nipa2	10.1954	9.66E-05
Nipa2	10.1954	9.66E-05
Nipa2	10.7126	7.93E-05
Nipa2	10.7126	7.93E-05
Nipa2	10.7126	7.93E-05
Nipa2	10.1954	9.66E-05

Nipa2	10.7126	7.93E-05
Nipal1	10.1954	9.66E-05
Nipal1	10.1954	9.66E-05
Nipsnap1	10.1954	9.66E-05
Nipsnap1	10.7126	7.93E-05
Nlrp2	10.7126	7.93E-05
Nlrp2	10.7126	7.93E-05
Nlrp4b	10.1954	9.66E-05
Nlrp4b	10.7126	7.93E-05
Nmd3	10.1954	9.66E-05
Nmd3	10.1954	9.66E-05
Nmd3	10.7126	7.93E-05
Nmu	10.1954	9.66E-05
Nmu	10.1954	9.66E-05
Nmu	10.7126	7.93E-05
Nmu	10.7126	7.93E-05
Nob1	10.1954	9.66E-05
Noc2l	10.1954	9.66E-05
Noc2l	10.1954	9.66E-05
Noc2l	10.1954	9.66E-05
Noc2l	10.7126	7.93E-05
Nol11	10.1954	9.66E-05
Nol11	10.1954	9.66E-05
Nol11	10.1954	9.66E-05
Nol11	10.1954	9.66E-05
Nol11	10.7126	7.93E-05
Nolc1	10.1954	9.66E-05
Nolc1	10.7126	7.93E-05
Nr4a2	10.7126	7.93E-05
Nrde2	10.7126	7.93E-05
Nrde2	10.7126	7.93E-05
Nrg1	10.7126	7.93E-05
Nrg4	10.7126	7.93E-05
Nsfl1c	10.7126	7.93E-05
Nsun3	10.7126	7.93E-05
Ntmt1	10.7126	7.93E-05
Nudt22	10.7126	7.93E-05
Nudt22	10.7126	7.93E-05
Nudt6	10.1954	9.66E-05
Nup205	10.1954	9.66E-05
Nup205	10.1954	9.66E-05
Nup205	10.1954	9.66E-05
Nup62cl	10.7126	7.93E-05
Oas1h	10.7126	7.93E-05
Olfr653	10.1954	9.66E-05
Oma1	10.1954	9.66E-05
Oma1	10.1954	9.66E-05
Oosp3	10.1954	9.66E-05
Oosp3	10.1954	9.66E-05
Oosp3	10.7126	7.93E-05
Oosp3	10.1954	9.66E-05
Oosp3	10.1954	9.66E-05
Oosp3	10.7126	7.93E-05
Ophn1	10.1954	9.66E-05
Ophn1	10.1954	9.66E-05
Ophn1	10.1954	9.66E-05
Ophn1	10.1954	9.66E-05
Ophn1	10.7126	7.93E-05

Orc4	10.1954	9.66E-05
Orc4	10.1954	9.66E-05
Orc4	10.1954	9.66E-05
Orc4	10.1954	9.66E-05
Orc4	10.1954	9.66E-05
Orc4	10.1954	9.66E-05
Orc4	10.1954	9.66E-05
Orc4	10.1954	9.66E-05
Orc4	10.7126	7.93E-05
Orc4	10.7126	7.93E-05
Orc4	10.7126	7.93E-05
Orc4	10.7126	7.93E-05
Orc4	10.7126	7.93E-05
Orc4	10.7126	7.93E-05
Orc6	10.1954	9.66E-05
Orc6	10.1954	9.66E-05
Orc6	10.1954	9.66E-05
Orc6	10.1954	9.66E-05
Orc6	10.7126	7.93E-05
Ovgp1	10.1954	9.66E-05
P4ha1	10.1954	9.66E-05
P4ha1	10.1954	9.66E-05
P4ha1	10.1954	9.66E-05
P4ha1	10.7126	7.93E-05
Pafah1b1	10.1954	9.66E-05
Pafah1b1	10.1954	9.66E-05
Pafah1b1	10.7126	7.93E-05
Pafah1b1	10.1954	9.66E-05
Pafah1b1	10.1954	9.66E-05
Pafah1b1	10.7126	7.93E-05
Pah	10.7126	7.93E-05
Pard3	10.7126	7.93E-05
Pard3	10.7126	7.93E-05
Parp2	10.1954	9.66E-05
Parp2	10.1954	9.66E-05
Parp2	10.7126	7.93E-05
Parp3	10.7126	7.93E-05
Pate2	10.1954	9.66E-05
Pate2	10.1954	9.66E-05
Pate2	10.7126	7.93E-05
Pate2	10.1954	9.66E-05
Pate2	10.7126	7.93E-05
Pbrm1	10.1954	9.66E-05
Pbrm1	10.7126	7.93E-05
Pbrm1	10.1954	9.66E-05
Pbrm1	10.7126	7.93E-05
Pcgf1	10.7126	7.93E-05
Pcgf1	10.7126	7.93E-05
Pcmt1	10.7126	7.93E-05
Pdcd10	10.7126	7.93E-05
Pdcd2l	10.1954	9.66E-05
Pdcd2l	10.7126	7.93E-05
Pdha1	10.1954	9.66E-05
Pdia4	10.1954	9.66E-05
Pdk1	10.1954	9.66E-05
Pdk1	10.7126	7.93E-05
Pdk1	10.7126	7.93E-05
Pdk1	10.7126	7.93E-05

Pdk1	10.7126	7.93E-05
Pex2	10.7126	7.93E-05
Pex2	10.7126	7.93E-05
Pex2	10.7126	7.93E-05
Pex2	10.7126	7.93E-05
Phf11c	10.1954	9.66E-05
Phf11c	10.1954	9.66E-05
Phf11c	10.1954	9.66E-05
Phf11c	10.1954	9.66E-05
Phkg1	10.7126	7.93E-05
Phkg1	10.7126	7.93E-05
Phkg2	10.1954	9.66E-05
Phkg2	10.1954	9.66E-05
Phkg2	10.1954	9.66E-05
Phtf2	10.1954	9.66E-05
Phtf2	10.1954	9.66E-05
Phtf2	10.1954	9.66E-05
Phtf2	10.1954	9.66E-05
Phtf2	10.1954	9.66E-05
Phtf2	10.7126	7.93E-05
Pik3cd	10.1954	9.66E-05
Pik3cd	10.1954	9.66E-05
Pik3cd	10.7126	7.93E-05
Pim2	10.1954	9.66E-05
Pja2	10.1954	9.66E-05
Pkd1l2	10.7126	7.93E-05
Pkd1l2	10.7126	7.93E-05
Pkib	10.7126	7.93E-05
Pkib	10.7126	7.93E-05
Pkig	10.1954	9.66E-05
Pkig	10.1954	9.66E-05
Pkig	10.1954	9.66E-05
Pkig	10.1954	9.66E-05
Pkig	10.1954	9.66E-05
Pkig	10.1954	9.66E-05
Pkig	10.1954	9.66E-05
Pkig	10.7126	7.93E-05
Pkmyt1	10.1954	9.66E-05
Pkmyt1	10.1954	9.66E-05
Plekha1	10.1954	9.66E-05
Plekha1	10.1954	9.66E-05
Plekha1	10.1954	9.66E-05
Plekha1	10.1954	9.66E-05
Plekha4	10.1954	9.66E-05
Plekha4	10.1954	9.66E-05
Plekha4	10.1954	9.66E-05
Plekha4	10.1954	9.66E-05
Plekha4	10.1954	9.66E-05
Plekha4	10.7126	7.93E-05
Pms2	10.7126	7.93E-05
Pmvk	10.7126	7.93E-05
Pnisr	10.1954	9.66E-05
Pnisr	10.7126	7.93E-05
Pnisr	10.7126	7.93E-05
Pnisr	10.7126	7.93E-05
Pnisr	10.7126	7.93E-05
Pnisr	10.7126	7.93E-05
Pnisr	10.7126	7.93E-05

Popdc3	10.1954	9.66E-05
Pou2f1	10.1954	9.66E-05
Pou2f1	10.7126	7.93E-05
Pou2f1	10.7126	7.93E-05
Ppat	10.1954	9.66E-05
Ppat	10.1954	9.66E-05
Ppat	10.7126	7.93E-05
Ppat	10.7126	7.93E-05
Ppat	10.7126	7.93E-05
Ppfia1	10.7126	7.93E-05
Ppfia1	10.1954	9.66E-05
Ppfia1	10.7126	7.93E-05
Ppp2r2a	10.1954	9.66E-05
Ppp2r2a	10.7126	7.93E-05
Ppp2r2d	10.1954	9.66E-05
Ppp2r2d	10.1954	9.66E-05
Ppp2r2d	10.7126	7.93E-05
Ppp2r2d	10.7126	7.93E-05
Ppp4c	10.7126	7.93E-05
Ppp4c	10.7126	7.93E-05
Ppp4c	10.7126	7.93E-05
Ppp6r2	10.1954	9.66E-05
Ppp6r2	10.1954	9.66E-05
Ppp6r2	10.1954	9.66E-05
Ppp6r2	10.7126	7.93E-05
Pqlc2	10.7126	7.93E-05
Pqlc2	10.7126	7.93E-05
Prdm9	10.1954	9.66E-05
Prdm9	10.1954	9.66E-05
Prdm9	10.1954	9.66E-05
Prg4	10.1954	9.66E-05
Primpol	10.1954	9.66E-05
Primpol	10.7126	7.93E-05
Primpol	10.7126	7.93E-05
Prkcd	10.7126	7.93E-05
Prkcz	10.1954	9.66E-05
Prkcz	10.7126	7.93E-05
Prkcz	10.7126	7.93E-05
Prmt5	10.1954	9.66E-05
Prmt5	10.1954	9.66E-05
Prpf39	10.1954	9.66E-05
Prpf39	10.7126	7.93E-05
Prpf40a	10.1954	9.66E-05
Prr14l	10.1954	9.66E-05
Prr14l	10.1954	9.66E-05
Prr14l	10.1954	9.66E-05
Prr14l	10.1954	9.66E-05
Prr14l	10.1954	9.66E-05
Prr14l	10.7126	7.93E-05
Prr14l	10.7126	7.93E-05
Psat1	10.1954	9.66E-05
Psat1	10.7126	7.93E-05
Psma3	10.1954	9.66E-05
Psma3	10.1954	9.66E-05
Psma3	10.1954	9.66E-05
Psma3	10.1954	9.66E-05
Psmd14	10.1954	9.66E-05
Psmd14	10.1954	9.66E-05

Psmd14	10.1954	9.66E-05
Psmd14	10.1954	9.66E-05
Psmd14	10.1954	9.66E-05
Psmd14	10.1954	9.66E-05
Psmd14	10.1954	9.66E-05
Psmd14	10.1954	9.66E-05
Psmd14	10.1954	9.66E-05
Psmd14	10.1954	9.66E-05
Psmd14	10.7126	7.93E-05
Psmd14	10.7126	7.93E-05
Psmd14	10.1954	9.66E-05
Psmd14	10.1954	9.66E-05
Psmd14	10.1954	9.66E-05
Psmd14	10.1954	9.66E-05
Psmd14	10.1954	9.66E-05
Psmd14	10.7126	7.93E-05
Psme2	10.7126	7.93E-05
Psme2	10.7126	7.93E-05
Psme2	10.7126	7.93E-05
Ptpn4	10.1954	9.66E-05
Ptpn4	10.1954	9.66E-05
Ptpn4	10.1954	9.66E-05
Pum1	10.7126	7.93E-05
Pum1	10.7126	7.93E-05
Pum1	10.7126	7.93E-05
Pum1	10.7126	7.93E-05
Pum1	10.7126	7.93E-05
Pum1	10.7126	7.93E-05
Pum1	10.7126	7.93E-05
Pum1	10.1954	9.66E-05
Pum1	10.7126	7.93E-05
Pum1	10.7126	7.93E-05
Pum2	10.7126	7.93E-05
Pum2	10.7126	7.93E-05
Pusl1	10.7126	7.93E-05
Pusl1	10.7126	7.93E-05
Pwwp2a	10.1954	9.66E-05
Qrsl1	10.1954	9.66E-05
Qrsl1	10.1954	9.66E-05
Qrsl1	10.1954	9.66E-05
Qrsl1	10.7126	7.93E-05
Qrsl1	10.1954	9.66E-05
Qrsl1	10.1954	9.66E-05
Qrsl1	10.1954	9.66E-05
Qrsl1	10.7126	7.93E-05
Qtrt1	10.7126	7.93E-05
Qtrt1	10.7126	7.93E-05
R3hdm1	10.1954	9.66E-05
R3hdm1	10.1954	9.66E-05
R3hdm1	10.1954	9.66E-05
R3hdm1	10.1954	9.66E-05
R3hdm1	10.1954	9.66E-05
R3hdm1	10.1954	9.66E-05
R3hdm1	10.7126	7.93E-05
Rab11a	10.7126	7.93E-05
Rab25	10.7126	7.93E-05
Rab25	10.7126	7.93E-05
Rab34	10.7126	7.93E-05

Rab3gap2	10.1954	9.66E-05
Rabep1	10.7126	7.93E-05
Rad1	10.1954	9.66E-05
Rad18	10.1954	9.66E-05
Rad18	10.1954	9.66E-05
Rad18	10.1954	9.66E-05
Rad18	10.1954	9.66E-05
Rad18	10.1954	9.66E-05
Rad18	10.1954	9.66E-05
Rad50	10.7126	7.93E-05
Rad50	10.7126	7.93E-05
Rad9b	10.1954	9.66E-05
Raet1d	10.7126	7.93E-05
Raet1d	10.7126	7.93E-05
Raet1d	10.7126	7.93E-05
Ralgapb	10.1954	9.66E-05
Ralgapb	10.1954	9.66E-05
Ranbp17	10.1954	9.66E-05
Ranbp17	10.1954	9.66E-05
Ranbp17	10.7126	7.93E-05
Ranbp17	10.1954	9.66E-05
Ranbp17	10.1954	9.66E-05
Ranbp17	10.7126	7.93E-05
Ranbp17	10.7126	7.93E-05
Rasa4	10.7126	7.93E-05
Rasa4	10.7126	7.93E-05
Rbm25	10.1954	9.66E-05
Rbm26	10.1954	9.66E-05
Rbm28	10.1954	9.66E-05
Rbm28	10.1954	9.66E-05
Rbm28	10.1954	9.66E-05
Rbm28	10.7126	7.93E-05
Rbm28	10.7126	7.93E-05
Rbm39	10.1954	9.66E-05
Rbm39	10.1954	9.66E-05
Rbm39	10.7126	7.93E-05
Rbm6	10.1954	9.66E-05
Rbm6	10.1954	9.66E-05
Rcc1	10.7126	7.93E-05
Reps1	10.1954	9.66E-05
Reps1	10.7126	7.93E-05
Reps1	10.7126	7.93E-05
Rev1	10.7126	7.93E-05
Rev1	10.7126	7.93E-05
Rev1	10.7126	7.93E-05
Rhebl1	10.1954	9.66E-05
Rhebl1	10.1954	9.66E-05
Rhebl1	10.7126	7.93E-05
Rhebl1	10.7126	7.93E-05
Rhebl1	10.7126	7.93E-05
Rhebl1	10.1954	9.66E-05
Rhebl1	10.1954	9.66E-05
Rhebl1	10.7126	7.93E-05
Rhebl1	10.7126	7.93E-05
Riok1	10.1954	9.66E-05
Riok1	10.1954	9.66E-05
Riok1	10.7126	7.93E-05
Riok1	10.7126	7.93E-05

Riox2	10.1954	9.66E-05
Rit1	10.1954	9.66E-05
Rmnd1	10.1954	9.66E-05
Rnf141	10.1954	9.66E-05
Rnf141	10.1954	9.66E-05
Rnf141	10.7126	7.93E-05
Rnf141	10.7126	7.93E-05
Rnf182	10.1954	9.66E-05
Rnf182	10.1954	9.66E-05
Rnf182	10.1954	9.66E-05
Rnf182	10.1954	9.66E-05
Rnf182	10.1954	9.66E-05
Rnf182	10.1954	9.66E-05
Rnf185	10.7126	7.93E-05
Rnf185	10.7126	7.93E-05
Rnf185	10.7126	7.93E-05
Rnf185	10.7126	7.93E-05
Rnf216	10.1954	9.66E-05
Rnf216	10.1954	9.66E-05
Rnf216	10.7126	7.93E-05
Rnf216	10.7126	7.93E-05
Rnmt	10.1954	9.66E-05
Rnmt	10.1954	9.66E-05
RP23-127B8.5	10.7126	7.93E-05
RP23-128E7.2	10.1954	9.66E-05
RP23-128E7.2	10.7126	7.93E-05
RP23-128E7.2	10.7126	7.93E-05
RP23-129J8.5	10.1954	9.66E-05
RP23-129J8.5	10.7126	7.93E-05
RP23-129J8.5	10.7126	7.93E-05
RP23-129J8.5	10.7126	7.93E-05
RP23-136M3.6	10.1954	9.66E-05
RP23-136M3.6	10.1954	9.66E-05
RP23-145N17.1	10.1954	9.66E-05
RP23-145N17.1	10.1954	9.66E-05
RP23-145N17.1	10.7126	7.93E-05
RP23-147K15.4	10.1954	9.66E-05
RP23-147K15.4	10.1954	9.66E-05
RP23-147K15.4	10.1954	9.66E-05
RP23-147K15.4	10.1954	9.66E-05
RP23-147K15.4	10.1954	9.66E-05
RP23-147K15.4	10.1954	9.66E-05
RP23-147K15.4	10.1954	9.66E-05
RP23-147K15.4	10.7126	7.93E-05
RP23-147K15.4	10.1954	9.66E-05
RP23-147K15.4	10.1954	9.66E-05
RP23-147K15.4	10.1954	9.66E-05
RP23-147K15.4	10.1954	9.66E-05
RP23-147K15.4	10.1954	9.66E-05
RP23-147K15.4	10.1954	9.66E-05
RP23-147K15.4	10.1954	9.66E-05
RP23-147K15.4	10.7126	7.93E-05
RP23-175J8.3	10.1954	9.66E-05
RP23-175J8.3	10.1954	9.66E-05
RP23-175J8.3	10.7126	7.93E-05
RP23-186O3.11	10.7126	7.93E-05
RP23-186O3.11	10.7126	7.93E-05
RP23-211P15.1	10.1954	9.66E-05

RP23-211P15.1	10.1954	9.66E-05
RP23-233B6.2	10.1954	9.66E-05
RP23-233B6.2	10.1954	9.66E-05
RP23-233B6.2	10.7126	7.93E-05
RP23-250A14.2	10.1954	9.66E-05
RP23-250A14.2	10.1954	9.66E-05
RP23-250A14.2	10.1954	9.66E-05
RP23-250A14.2	10.1954	9.66E-05
RP23-250A14.2	10.1954	9.66E-05
RP23-250A14.2	10.1954	9.66E-05
RP23-250A14.2	10.1954	9.66E-05
RP23-250A14.2	10.1954	9.66E-05
RP23-250A14.2	10.1954	9.66E-05
RP23-250A14.2	10.1954	9.66E-05
RP23-250A14.2	10.1954	9.66E-05
RP23-250A14.2	10.1954	9.66E-05
RP23-250A14.2	10.1954	9.66E-05
RP23-250A14.2	10.1954	9.66E-05
RP23-250A14.2	10.1954	9.66E-05
RP23-250A14.2	10.1954	9.66E-05
RP23-250A14.2	10.1954	9.66E-05
RP23-250A14.2	10.1954	9.66E-05
RP23-250A14.2	10.7126	7.93E-05
RP23-250A14.2	10.7126	7.93E-05
RP23-250A14.2	10.7126	7.93E-05
RP23-255F14.8	10.1954	9.66E-05
RP23-255F14.8	10.1954	9.66E-05
RP23-262M12.1	10.7126	7.93E-05
RP23-262M12.1	10.7126	7.93E-05
RP23-268A17.3	10.1954	9.66E-05
RP23-268A17.3	10.1954	9.66E-05
RP23-268A17.3	10.7126	7.93E-05
RP23-268A17.3	10.7126	7.93E-05
RP23-276L18.1	10.1954	9.66E-05
RP23-276L18.1	10.1954	9.66E-05
RP23-286I5.3	10.7126	7.93E-05
RP23-290N23.1	10.1954	9.66E-05
RP23-290N23.1	10.1954	9.66E-05
RP23-294B15.2	10.1954	9.66E-05
RP23-294B15.2	10.1954	9.66E-05
RP23-302F9.3	10.7126	7.93E-05
RP23-313A10.2	10.1954	9.66E-05
RP23-313A10.2	10.1954	9.66E-05
RP23-313A10.2	10.7126	7.93E-05
RP23-314E23.2	10.1954	9.66E-05
RP23-314E23.2	10.1954	9.66E-05
RP23-314E23.2	10.1954	9.66E-05
RP23-320I11.1	10.7126	7.93E-05
RP23-326E2.4	10.1954	9.66E-05
RP23-326E2.4	10.7126	7.93E-05
RP23-331A21.2	10.7126	7.93E-05
RP23-331A21.2	10.7126	7.93E-05
RP23-356J2.10	10.7126	7.93E-05
RP23-356J2.10	10.7126	7.93E-05
RP23-359F5.1	10.7126	7.93E-05
RP23-359G8.3	10.1954	9.66E-05
RP23-359G8.3	10.1954	9.66E-05
RP23-359G8.3	10.7126	7.93E-05

RP23-359G8.3	10.7126	7.93E-05
RP23-359G8.3	10.1954	9.66E-05
RP23-359G8.3	10.1954	9.66E-05
RP23-359G8.3	10.7126	7.93E-05
RP23-359G8.3	10.7126	7.93E-05
RP23-360A2.8	10.7126	7.93E-05
RP23-366P9.8	10.1954	9.66E-05
RP23-366P9.8	10.7126	7.93E-05
RP23-366P9.8	10.1954	9.66E-05
RP23-366P9.8	10.7126	7.93E-05
RP23-382G13.1	10.1954	9.66E-05
RP23-382G13.1	10.1954	9.66E-05
RP23-395N7.8	10.1954	9.66E-05
RP23-395N7.8	10.1954	9.66E-05
RP23-395N7.8	10.1954	9.66E-05
RP23-395N7.8	10.7126	7.93E-05
RP23-395N7.8	10.7126	7.93E-05
RP23-395N7.8	10.7126	7.93E-05
RP23-404I24.3	10.1954	9.66E-05
RP23-404I24.3	10.1954	9.66E-05
RP23-404I24.3	10.7126	7.93E-05
RP23-409B11.1	10.1954	9.66E-05
RP23-409B11.1	10.1954	9.66E-05
RP23-409B11.1	10.1954	9.66E-05
RP23-414K1.4	10.1954	9.66E-05
RP23-414K1.4	10.7126	7.93E-05
RP23-414K1.4	10.7126	7.93E-05
RP23-414K1.4	10.7126	7.93E-05
RP23-414K1.4	10.7126	7.93E-05
RP23-414K1.4	10.7126	7.93E-05
RP23-414P13.3	10.7126	7.93E-05
RP23-414P13.3	10.7126	7.93E-05
RP23-423J10.4	10.7126	7.93E-05
RP23-423J10.4	10.7126	7.93E-05
RP23-424N5.2	10.1954	9.66E-05
RP23-424N5.2	10.1954	9.66E-05
RP23-424N5.2	10.7126	7.93E-05
RP23-424N5.2	10.7126	7.93E-05
RP23-424N5.2	10.7126	7.93E-05
RP23-424N5.2	10.7126	7.93E-05
RP23-425K3.2	10.7126	7.93E-05
RP23-440J15.6	10.7126	7.93E-05
RP23-440J15.6	10.7126	7.93E-05
RP23-440J15.6	10.7126	7.93E-05
RP23-440J15.6	10.7126	7.93E-05
RP23-445H7.1	10.1954	9.66E-05
RP23-445H7.1	10.7126	7.93E-05
RP23-477C12.1	10.1954	9.66E-05
RP23-477C12.1	10.1954	9.66E-05
RP23-47P3.3	10.1954	9.66E-05
RP23-48M16.9	10.1954	9.66E-05
RP23-48M16.9	10.1954	9.66E-05
RP23-48M16.9	10.1954	9.66E-05
RP23-48M16.9	10.7126	7.93E-05
RP23-62O7.9	10.7126	7.93E-05
RP23-62O7.9	10.7126	7.93E-05
RP23-67E6.3	10.7126	7.93E-05
RP23-78N8.3	10.1954	9.66E-05

RP23-78N8.3	10.1954	9.66E-05
RP23-78N8.3	10.1954	9.66E-05
RP23-78N8.3	10.1954	9.66E-05
RP23-78N8.3	10.1954	9.66E-05
RP23-78N8.3	10.7126	7.93E-05
RP23-78N8.3	10.7126	7.93E-05
RP23-95L9.5	10.7126	7.93E-05
RP23-95L9.5	10.7126	7.93E-05
RP24-103K4.2	10.1954	9.66E-05
RP24-103K4.2	10.1954	9.66E-05
RP24-103K4.2	10.1954	9.66E-05
RP24-103K4.2	10.1954	9.66E-05
RP24-147G7.1	10.1954	9.66E-05
RP24-147G7.1	10.7126	7.93E-05
RP24-149E13.1	10.1954	9.66E-05
RP24-149E13.1	10.1954	9.66E-05
RP24-149E13.1	10.1954	9.66E-05
RP24-149E13.1	10.7126	7.93E-05
RP24-166N8.6	10.1954	9.66E-05
RP24-166N8.6	10.1954	9.66E-05
RP24-213G21.1	10.1954	9.66E-05
RP24-213G21.1	10.7126	7.93E-05
RP24-238M4.4	10.1954	9.66E-05
RP24-238M4.4	10.7126	7.93E-05
RP24-238M4.4	10.7126	7.93E-05
RP24-309H3.4	10.1954	9.66E-05
RP24-309H3.4	10.1954	9.66E-05
RP24-309H3.4	10.1954	9.66E-05
RP24-336D11.5	10.1954	9.66E-05
RP24-336D11.5	10.7126	7.93E-05
RP24-353D23.1	10.7126	7.93E-05
RP24-370N20.1	10.1954	9.66E-05
RP24-370N20.1	10.1954	9.66E-05
RP24-370N20.1	10.7126	7.93E-05
RP24-372K3.1	10.1954	9.66E-05
RP24-372K3.1	10.1954	9.66E-05
RP24-458J4.8	10.1954	9.66E-05
RP24-458J4.8	10.7126	7.93E-05
RP24-483G17.1	10.7126	7.93E-05
RP24-483G17.1	10.7126	7.93E-05
RP24-498L21.2	10.1954	9.66E-05
RP24-498L21.2	10.1954	9.66E-05
RP24-498L21.2	10.7126	7.93E-05
RP24-502H14.1	10.1954	9.66E-05
RP24-502H14.1	10.7126	7.93E-05
RP24-502J3.1	10.1954	9.66E-05
RP24-510G5.3	10.1954	9.66E-05
RP24-510G5.3	10.1954	9.66E-05
RP24-510G5.3	10.7126	7.93E-05
RP24-510G5.3	10.7126	7.93E-05
RP24-510G5.3	10.7126	7.93E-05
RP24-510G5.3	10.7126	7.93E-05
RP24-510G5.3	10.7126	7.93E-05
RP24-534N1.1	10.1954	9.66E-05
RP24-534N1.1	10.1954	9.66E-05
RP24-534N1.1	10.1954	9.66E-05
RP24-534N1.1	10.1954	9.66E-05
RP24-63B17.4	10.7126	7.93E-05

RP24-91A16.3	10.1954	9.66E-05
RP24-91A16.3	10.1954	9.66E-05
RP24-91A16.3	10.1954	9.66E-05
RP24-93B5.3	10.1954	9.66E-05
RP24-93B5.3	10.1954	9.66E-05
RP24-93B5.3	10.1954	9.66E-05
RP24-93B5.3	10.1954	9.66E-05
RP24-93B5.3	10.7126	7.93E-05
RP24-93B5.3	10.7126	7.93E-05
RP24-93B5.3	10.7126	7.93E-05
Rpe	10.7126	7.93E-05
Rpe	10.7126	7.93E-05
Rpe	10.7126	7.93E-05
Rph3al	10.7126	7.93E-05
Rpl36	10.7126	7.93E-05
Rpl36	10.7126	7.93E-05
Rpl36	10.7126	7.93E-05
Rpl36	10.7126	7.93E-05
Rpl36	10.7126	7.93E-05
Rpl3l	10.1954	9.66E-05
Rpl3l	10.1954	9.66E-05
Rpl3l	10.1954	9.66E-05
Rpusd3	10.7126	7.93E-05
Rrh	10.1954	9.66E-05
Rrh	10.7126	7.93E-05
Rrp1b	10.7126	7.93E-05
Rrp1b	10.7126	7.93E-05
Rsl1d1	10.1954	9.66E-05
Rsl1d1	10.7126	7.93E-05
Rsl1d1	10.7126	7.93E-05
Rsph10b	10.1954	9.66E-05
Rsph10b	10.1954	9.66E-05
Rsph10b	10.7126	7.93E-05
Rsph10b	10.7126	7.93E-05
Rsrc2	10.1954	9.66E-05
Rsrc2	10.1954	9.66E-05
Rsrc2	10.1954	9.66E-05
Rsrc2	10.7126	7.93E-05
Rsrc2	10.7126	7.93E-05
Rsrp1	10.1954	9.66E-05
Rsrp1	10.7126	7.93E-05
Rsrp1	10.7126	7.93E-05
Rsrp1	10.7126	7.93E-05
Rsrp1	10.7126	7.93E-05
Rtbdn	10.1954	9.66E-05
Rtbdn	10.1954	9.66E-05
Rtbdn	10.7126	7.93E-05
Rtbdn	10.7126	7.93E-05
Rtbdn	10.7126	7.93E-05
Rtbdn	10.7126	7.93E-05
Rtbdn	10.7126	7.93E-05
Rtbdn	10.7126	7.93E-05
Rtbdn	10.7126	7.93E-05
Rtbdn	10.7126	7.93E-05
Rtel1	10.1954	9.66E-05
Rtel1	10.1954	9.66E-05
Samd7	10.1954	9.66E-05
Sap130	10.1954	9.66E-05

Sap130	10.1954	9.66E-05
Sar1b	10.7126	7.93E-05
Sar1b	10.7126	7.93E-05
Sat2	10.1954	9.66E-05
Scp2	10.7126	7.93E-05
Sec11a	10.1954	9.66E-05
Sec11a	10.1954	9.66E-05
Sec11a	10.1954	9.66E-05
Sec11a	10.1954	9.66E-05
Sec11a	10.1954	9.66E-05
Sec11a	10.1954	9.66E-05
Sec11a	10.1954	9.66E-05
Sec11a	10.1954	9.66E-05
Sec11a	10.1954	9.66E-05
Sec11a	10.1954	9.66E-05
Sec61a2	10.1954	9.66E-05
Sel1l	10.1954	9.66E-05
Sel1l	10.1954	9.66E-05
Sel1l	10.1954	9.66E-05
Sel1l2	10.1954	9.66E-05
Sel1l2	10.7126	7.93E-05
Senp5	10.1954	9.66E-05
Senp5	10.7126	7.93E-05
sequence name	score	p-value
Serpinb1c	10.7126	7.93E-05
Serpinb1c	10.7126	7.93E-05
Setd2	10.1954	9.66E-05
Setd2	10.1954	9.66E-05
Setd2	10.7126	7.93E-05
Setd2	10.7126	7.93E-05
Setd5	10.1954	9.66E-05
Setd5	10.1954	9.66E-05
Setdb1	10.1954	9.66E-05
Setdb1	10.1954	9.66E-05
Setdb1	10.1954	9.66E-05
Setdb1	10.7126	7.93E-05
Setdb1	10.7126	7.93E-05
Sft2d1	10.1954	9.66E-05
Sfta2	10.7126	7.93E-05
Sgf29	10.7126	7.93E-05
Sgf29	10.7126	7.93E-05
Sgms1	10.7126	7.93E-05
Sgms1	10.7126	7.93E-05
Shroom2	10.1954	9.66E-05
Shroom2	10.7126	7.93E-05
Sipa1l3	10.7126	7.93E-05
Slbp	10.1954	9.66E-05
Slbp	10.1954	9.66E-05
Slbp	10.1954	9.66E-05
Slbp	10.1954	9.66E-05
Slbp	10.1954	9.66E-05
Slbp	10.7126	7.93E-05
Slc11a1	10.7126	7.93E-05
Slc11a1	10.7126	7.93E-05
Slc17a2	10.1954	9.66E-05
Slc17a2	10.1954	9.66E-05
Slc17a2	10.7126	7.93E-05
Slc18b1	10.1954	9.66E-05

Slc1a3	10.1954	9.66E-05
Slc1a3	10.1954	9.66E-05
Slc1a3	10.1954	9.66E-05
Slc1a3	10.1954	9.66E-05
Slc1a3	10.1954	9.66E-05
Slc1a3	10.1954	9.66E-05
Slc1a3	10.1954	9.66E-05
Slc1a3	10.1954	9.66E-05
Slc1a3	10.1954	9.66E-05
Slc1a3	10.1954	9.66E-05
Slc1a3	10.1954	9.66E-05
Slc1a3	10.1954	9.66E-05
Slc1a3	10.1954	9.66E-05
Slc1a3	10.1954	9.66E-05
Slc1a3	10.1954	9.66E-05
Slc1a3	10.1954	9.66E-05
Slc1a3	10.1954	9.66E-05
Slc1a3	10.1954	9.66E-05
Slc1a3	10.1954	9.66E-05
Slc1a3	10.1954	9.66E-05
Slc1a3	10.7126	7.93E-05
Slc1a3	10.7126	7.93E-05
Slc22a21	10.1954	9.66E-05
Slc22a21	10.7126	7.93E-05
Slc25a19	10.1954	9.66E-05
Slc25a19	10.1954	9.66E-05
Slc25a26	10.7126	7.93E-05
Slc25a36	10.1954	9.66E-05
Slc25a36	10.1954	9.66E-05
Slc25a40	10.1954	9.66E-05
Slc25a40	10.7126	7.93E-05
Slc30a2	10.7126	7.93E-05
Slc30a2	10.7126	7.93E-05
Slc30a5	10.1954	9.66E-05
Slc30a5	10.1954	9.66E-05
Slc30a5	10.1954	9.66E-05
Slc30a5	10.7126	7.93E-05
Slc30a5	10.7126	7.93E-05
Slc30a5	10.7126	7.93E-05
Slc30a6	10.1954	9.66E-05
Slc30a6	10.1954	9.66E-05
Slc30a6	10.1954	9.66E-05
Slc30a6	10.1954	9.66E-05
Slc30a6	10.1954	9.66E-05
Slc30a6	10.7126	7.93E-05
Slc30a6	10.7126	7.93E-05
Slc30a6	10.7126	7.93E-05
Slc30a6	10.7126	7.93E-05
Slc37a3	10.7126	7.93E-05
Slc37a3	10.7126	7.93E-05
Slc39a9	10.7126	7.93E-05
Slc39a9	10.7126	7.93E-05
Slco4a1	10.1954	9.66E-05
Smarcad1	10.1954	9.66E-05
Smarcb1	10.7126	7.93E-05
Smarcb1	10.7126	7.93E-05
Smarcb1	10.7126	7.93E-05
Smc2	10.1954	9.66E-05

Smc2	10.1954	9.66E-05
Smc2	10.1954	9.66E-05
Smc2	10.1954	9.66E-05
Smim8	10.1954	9.66E-05
Smim8	10.1954	9.66E-05
Smim8	10.7126	7.93E-05
Smim8	10.7126	7.93E-05
Smim8	10.1954	9.66E-05
Smim8	10.1954	9.66E-05
Smim8	10.7126	7.93E-05
Smim8	10.7126	7.93E-05
Smu1	10.7126	7.93E-05
Smu1	10.7126	7.93E-05
Snap91	10.1954	9.66E-05
Snap91	10.1954	9.66E-05
Snap91	10.7126	7.93E-05
Snhg5	10.1954	9.66E-05
Snrnp25	10.7126	7.93E-05
Snrnp25	10.7126	7.93E-05
Snx14	10.1954	9.66E-05
Sorbs2	10.7126	7.93E-05
Sp100	10.7126	7.93E-05
Sp100	10.7126	7.93E-05
Spata21	10.1954	9.66E-05
Spata21	10.1954	9.66E-05
Spata6	10.1954	9.66E-05
Spata6	10.1954	9.66E-05
Spata6	10.7126	7.93E-05
Spata6	10.7126	7.93E-05
Spdye4b	10.7126	7.93E-05
Spg7	10.1954	9.66E-05
Spg7	10.7126	7.93E-05
Spg7	10.7126	7.93E-05
Sppl2a	10.1954	9.66E-05
Sppl2a	10.1954	9.66E-05
Sppl2a	10.7126	7.93E-05
Sppl2a	10.7126	7.93E-05
Srm	10.1954	9.66E-05
Srsf3	10.1954	9.66E-05
Srsf3	10.1954	9.66E-05
Srsf3	10.7126	7.93E-05
Srsf3	10.1954	9.66E-05
Srsf3	10.1954	9.66E-05
Srsf3	10.7126	7.93E-05
Stac2	10.7126	7.93E-05
Stac2	10.7126	7.93E-05
Stk36	10.1954	9.66E-05
Stk36	10.1954	9.66E-05
Strada	10.1954	9.66E-05
Strada	10.1954	9.66E-05
Strada	10.7126	7.93E-05
Strn3	10.1954	9.66E-05
Strn3	10.1954	9.66E-05
Styx	10.1954	9.66E-05
Styx	10.1954	9.66E-05
Styx	10.7126	7.93E-05
Sumf1	10.1954	9.66E-05
Sumf1	10.1954	9.66E-05

Sumf1	10.7126	7.93E-05
Sycp1	10.1954	9.66E-05
Sycp1	10.1954	9.66E-05
Sycp1	10.1954	9.66E-05
Sycp1	10.1954	9.66E-05
Sycp1	10.1954	9.66E-05
Sycp1	10.1954	9.66E-05
Sycp1	10.1954	9.66E-05
Sycp1	10.1954	9.66E-05
Sycp1	10.1954	9.66E-05
Sycp1	10.7126	7.93E-05
Sycp2l	10.1954	9.66E-05
Sycp2l	10.7126	7.93E-05
Sycp2l	10.7126	7.93E-05
Sycp2l	10.1954	9.66E-05
Sycp2l	10.7126	7.93E-05
Sycp2l	10.7126	7.93E-05
Szrd1	10.7126	7.93E-05
Taf1b	10.7126	7.93E-05
Taf1b	10.1954	9.66E-05
Taf1b	10.7126	7.93E-05
Tango2	10.1954	9.66E-05
Tango2	10.1954	9.66E-05
Tango2	10.7126	7.93E-05
Tango2	10.7126	7.93E-05
Tank	10.1954	9.66E-05
Tank	10.1954	9.66E-05
Tank	10.1954	9.66E-05
Tank	10.1954	9.66E-05
Tank	10.1954	9.66E-05
Tank	10.1954	9.66E-05
Tank	10.1954	9.66E-05
Tank	10.1954	9.66E-05
Tank	10.1954	9.66E-05
Tank	10.1954	9.66E-05
Tank	10.1954	9.66E-05
Tank	10.7126	7.93E-05
Tank	10.7126	7.93E-05
Tarbp2	10.7126	7.93E-05
Tarbp2	10.7126	7.93E-05
Tarbp2	10.7126	7.93E-05
Tarbp2	10.7126	7.93E-05
Tarbp2	10.7126	7.93E-05
Tardbp	10.7126	7.93E-05
Tardbp	10.7126	7.93E-05
Tars2	10.7126	7.93E-05
Tars2	10.7126	7.93E-05
Tatdn3	10.7126	7.93E-05
Tatdn3	10.7126	7.93E-05
Tatdn3	10.7126	7.93E-05
Tatdn3	10.7126	7.93E-05
Tatdn3	10.7126	7.93E-05
Tbp	10.1954	9.66E-05
Tbp	10.1954	9.66E-05
Tcf4	10.1954	9.66E-05
Tcf4	10.1954	9.66E-05
Tcf4	10.1954	9.66E-05
Tchp	10.7126	7.93E-05

Tdh	10.1954	9.66E-05
Tdh	10.1954	9.66E-05
Tdh	10.7126	7.93E-05
Tdp2	10.1954	9.66E-05
Tdp2	10.1954	9.66E-05
Tdp2	10.1954	9.66E-05
Tdp2	10.7126	7.93E-05
Tdp2	10.7126	7.93E-05
Tdp2	10.7126	7.93E-05
Tdp2	10.7126	7.93E-05
Tdp2	10.7126	7.93E-05
Tdp2	10.7126	7.93E-05
Tdp2	10.7126	7.93E-05
Tdp2	10.7126	7.93E-05
Tdp2	10.7126	7.93E-05
Tdrd5	10.1954	9.66E-05
Tdrd5	10.7126	7.93E-05
Tdrd5	10.7126	7.93E-05
Tesk2	10.1954	9.66E-05
Tet1	10.1954	9.66E-05
Tet1	10.1954	9.66E-05
Tet1	10.1954	9.66E-05
Tet1	10.1954	9.66E-05
Tet1	10.1954	9.66E-05
Tet1	10.7126	7.93E-05
Tex2	10.1954	9.66E-05
Tex30	10.7126	7.93E-05
Tex30	10.7126	7.93E-05
Tex43	10.7126	7.93E-05
Tex9	10.1954	9.66E-05
Tex9	10.1954	9.66E-05
Tex9	10.7126	7.93E-05
Tex9	10.7126	7.93E-05
Tex9	10.7126	7.93E-05
Tex9	10.7126	7.93E-05
Tex9	10.1954	9.66E-05
Tex9	10.1954	9.66E-05
Tex9	10.1954	9.66E-05
Tex9	10.1954	9.66E-05
Tex9	10.7126	7.93E-05
Tex9	10.7126	7.93E-05
Tex9	10.7126	7.93E-05
Tfb2m	10.1954	9.66E-05
Tfb2m	10.1954	9.66E-05
Tfb2m	10.1954	9.66E-05
Tfb2m	10.7126	7.93E-05
Tfb2m	10.7126	7.93E-05
Tfdp2	10.1954	9.66E-05
Tfdp2	10.1954	9.66E-05
Tfdp2	10.1954	9.66E-05
Tfdp2	10.7126	7.93E-05
Tfdp2	10.7126	7.93E-05
Tfdp2	10.7126	7.93E-05
Tfdp2	10.7126	7.93E-05
Tfdp2	10.7126	7.93E-05
Thoc2	10.1954	9.66E-05
Thoc2	10.1954	9.66E-05
Thoc2	10.7126	7.93E-05

Thoc2	10.7126	7.93E-05
Thoc2	10.1954	9.66E-05
Thoc2	10.7126	7.93E-05
Thrap3	10.1954	9.66E-05
Thrap3	10.1954	9.66E-05
Thrap3	10.1954	9.66E-05
Tial1	10.1954	9.66E-05
Tiam1	10.1954	9.66E-05
Tiam1	10.1954	9.66E-05
Tiam1	10.1954	9.66E-05
Ticrr	10.1954	9.66E-05
Ticrr	10.1954	9.66E-05
Ticrr	10.1954	9.66E-05
Ticrr	10.1954	9.66E-05
Ticrr	10.1954	9.66E-05
Ticrr	10.7126	7.93E-05
Ticrr	10.7126	7.93E-05
Timd2	10.1954	9.66E-05
Timd2	10.7126	7.93E-05
Timm23	10.1954	9.66E-05
Timm23	10.7126	7.93E-05
Timm23	10.7126	7.93E-05
Timm44	10.1954	9.66E-05
Tktl1	10.1954	9.66E-05
Tktl1	10.1954	9.66E-05
Tktl1	10.1954	9.66E-05
Tktl1	10.1954	9.66E-05
Tktl1	10.1954	9.66E-05
Tktl1	10.1954	9.66E-05
Tktl1	10.1954	9.66E-05
Tktl1	10.7126	7.93E-05
Tktl1	10.7126	7.93E-05
Tle1	10.1954	9.66E-05
Tle6	10.1954	9.66E-05
Tle6	10.1954	9.66E-05
Tm9sf1	10.1954	9.66E-05
Tm9sf1	10.7126	7.93E-05
Tmco3	10.1954	9.66E-05
Tmco3	10.7126	7.93E-05
Tmem114	10.7126	7.93E-05
Tmem14c	10.1954	9.66E-05
Tmem175	10.1954	9.66E-05
Tmem175	10.7126	7.93E-05
Tmem175	10.7126	7.93E-05
Tmem190	10.1954	9.66E-05
Tmem190	10.1954	9.66E-05
Tmem190	10.1954	9.66E-05
Tmem209	10.1954	9.66E-05
Tmem209	10.7126	7.93E-05
Tmem209	10.7126	7.93E-05
Tmem230	10.1954	9.66E-05
Tmem230	10.7126	7.93E-05
Tmem234	10.7126	7.93E-05
Tmem234	10.7126	7.93E-05
Tmem234	10.7126	7.93E-05
Tmem234	10.7126	7.93E-05
Tmem234	10.7126	7.93E-05
Tmem234	10.7126	7.93E-05

Tmem234	10.7126	7.93E-05
Tmem234	10.7126	7.93E-05
Tmem234	10.7126	7.93E-05
Tmem234	10.7126	7.93E-05
Tmem234	10.7126	7.93E-05
Tmem38b	10.7126	7.93E-05
Tmem62	10.7126	7.93E-05
Tmf1	10.7126	7.93E-05
Tnfrsf12a	10.7126	7.93E-05
Tnfrsf12a	10.7126	7.93E-05
Tnfrsf12a	10.7126	7.93E-05
Tnfrsf12a	10.7126	7.93E-05
Tnfrsf13b	10.1954	9.66E-05
Tnfrsf13b	10.1954	9.66E-05
Tnfrsf19	10.1954	9.66E-05
Tnfrsf19	10.1954	9.66E-05
Tnfrsf19	10.1954	9.66E-05
Tnfrsf19	10.1954	9.66E-05
Tnfrsf19	10.1954	9.66E-05
Tnfrsf19	10.1954	9.66E-05
Tnfrsf19	10.1954	9.66E-05
Tnfrsf19	10.1954	9.66E-05
Tnfrsf19	10.1954	9.66E-05
Tnfrsf19	10.1954	9.66E-05
Tnfrsf19	10.1954	9.66E-05
Tnfrsf19	10.1954	9.66E-05
Tnfrsf19	10.1954	9.66E-05
Tnfrsf19	10.1954	9.66E-05
Tnfrsf19	10.1954	9.66E-05
Tnfrsf19	10.1954	9.66E-05
Tnfrsf19	10.1954	9.66E-05
Tnfrsf19	10.1954	9.66E-05
Tnfrsf19	10.7126	7.93E-05
Tnfrsf19	10.7126	7.93E-05
Tollip	10.1954	9.66E-05
Tollip	10.1954	9.66E-05
Tollip	10.7126	7.93E-05
Tomm70a	10.1954	9.66E-05
Tomm70a	10.1954	9.66E-05
Tomm70a	10.1954	9.66E-05
Tomm70a	10.1954	9.66E-05
Tomm70a	10.1954	9.66E-05
Tomm70a	10.1954	9.66E-05
Tomm70a	10.1954	9.66E-05
Tomm70a	10.7126	7.93E-05
Tomm70a	10.7126	7.93E-05
Tomm70a	10.7126	7.93E-05
Tomm70a	10.7126	7.93E-05
Tpd52	10.1954	9.66E-05
Tpd52	10.7126	7.93E-05
Tpd52	10.7126	7.93E-05
Tpd52l2	10.1954	9.66E-05
Tpd52l2	10.1954	9.66E-05
Tpd52l2	10.7126	7.93E-05
Tpd52l2	10.7126	7.93E-05
Tpd52l2	10.7126	7.93E-05
Tpd52l2	10.7126	7.93E-05
Tpd52l2	10.7126	7.93E-05

Tph2	10.1954	9.66E-05
Tph2	10.7126	7.93E-05
Tpra1	10.7126	7.93E-05
Tpra1	10.7126	7.93E-05
Tra2a	10.1954	9.66E-05
Tra2a	10.7126	7.93E-05
Traf2	10.7126	7.93E-05
Traf2	10.7126	7.93E-05
Trappc6b	10.7126	7.93E-05
Trappc8	10.1954	9.66E-05
Trappc8	10.1954	9.66E-05
Trappc8	10.7126	7.93E-05
Trappc8	10.7126	7.93E-05
Trip4	10.1954	9.66E-05
Trip4	10.1954	9.66E-05
Trip4	10.1954	9.66E-05
Trip4	10.1954	9.66E-05
Trip4	10.1954	9.66E-05
Trip4	10.1954	9.66E-05
Trmt11	10.1954	9.66E-05
Trmt11	10.7126	7.93E-05
Trmt1l	10.1954	9.66E-05
Trmt1l	10.1954	9.66E-05
Trmt1l	10.1954	9.66E-05
Trmt1l	10.7126	7.93E-05
Troap	10.7126	7.93E-05
Troap	10.7126	7.93E-05
Troap	10.7126	7.93E-05
Troap	10.7126	7.93E-05
Trpc1	10.1954	9.66E-05
Trpc1	10.1954	9.66E-05
Trpc1	10.1954	9.66E-05
Tsr3	10.1954	9.66E-05
Tsr3	10.1954	9.66E-05
Tsr3	10.1954	9.66E-05
Tsr3	10.1954	9.66E-05
Tsr3	10.1954	9.66E-05
Tsr3	10.1954	9.66E-05
Tsr3	10.7126	7.93E-05
Tsr3	10.7126	7.93E-05
Tsta3	10.1954	9.66E-05
Ttc19	10.1954	9.66E-05
Ttc19	10.1954	9.66E-05
Ttc19	10.1954	9.66E-05
Ttc19	10.1954	9.66E-05
Ttc19	10.7126	7.93E-05
Tti2	10.1954	9.66E-05
Tti2	10.7126	7.93E-05
Tti2	10.7126	7.93E-05
Tti2	10.7126	7.93E-05
Tulp2	10.7126	7.93E-05
Tulp2	10.7126	7.93E-05
Txndc11	10.7126	7.93E-05
Txndc11	10.7126	7.93E-05
Tyms	10.1954	9.66E-05
Tyms	10.1954	9.66E-05
Tyms	10.1954	9.66E-05
Tyms	10.7126	7.93E-05

Uap1l1	10.7126	7.93E-05
Uap1l1	10.7126	7.93E-05
Ubac2	10.7126	7.93E-05
Ubac2	10.7126	7.93E-05
Ubac2	10.7126	7.93E-05
Ubac2	10.1954	9.66E-05
Ubac2	10.1954	9.66E-05
Ubac2	10.7126	7.93E-05
Ubac2	10.7126	7.93E-05
Ubac2	10.7126	7.93E-05
Ube2d3	10.7126	7.93E-05
Ube2d3	10.7126	7.93E-05
Ube2d3	10.7126	7.93E-05
Ube2d3	10.7126	7.93E-05
Ube2d-ps	10.7126	7.93E-05
Ube2f	10.7126	7.93E-05
Ube2h	10.1954	9.66E-05
Ube2w	10.1954	9.66E-05
Ube2w	10.1954	9.66E-05
Ube2w	10.1954	9.66E-05
Ube2w	10.1954	9.66E-05
Ube2w	10.1954	9.66E-05
Ubp1	10.1954	9.66E-05
Ubp1	10.1954	9.66E-05
Ubp1	10.7126	7.93E-05
Ubp1	10.7126	7.93E-05
Uchl3	10.1954	9.66E-05
Uchl3	10.1954	9.66E-05
Uchl3	10.1954	9.66E-05
Uchl3	10.7126	7.93E-05
Uchl3	10.7126	7.93E-05
Uchl5	10.1954	9.66E-05
Uchl5	10.1954	9.66E-05
Uchl5	10.1954	9.66E-05
Uchl5	10.1954	9.66E-05
Uchl5	10.1954	9.66E-05
Uevld	10.1954	9.66E-05
Uevld	10.1954	9.66E-05
Uevld	10.7126	7.93E-05
Uevld	10.7126	7.93E-05
Uevld	10.7126	7.93E-05
Ufsp2	10.1954	9.66E-05
Ufsp2	10.1954	9.66E-05
Ufsp2	10.1954	9.66E-05
Uhrf1bp1l	10.1954	9.66E-05
Uhrf1bp1l	10.7126	7.93E-05
Unc45b	10.7126	7.93E-05
Unkl	10.1954	9.66E-05
Unkl	10.1954	9.66E-05
Unkl	10.1954	9.66E-05
Unkl	10.1954	9.66E-05
Usp20	10.7126	7.93E-05
Usp48	10.1954	9.66E-05
Usp48	10.1954	9.66E-05
Usp48	10.1954	9.66E-05
Usp48	10.1954	9.66E-05
Usp48	10.1954	9.66E-05
Usp48	10.7126	7.93E-05

Usp48	10.7126	7.93E-05
Usp48	10.7126	7.93E-05
Usp48	10.1954	9.66E-05
Usp48	10.1954	9.66E-05
Usp48	10.7126	7.93E-05
Usp48	10.7126	7.93E-05
Utp20	10.1954	9.66E-05
Utp20	10.1954	9.66E-05
Utp20	10.7126	7.93E-05
Vmn1r69	10.7126	7.93E-05
Vps16	10.7126	7.93E-05
Vps16	10.7126	7.93E-05
Vps26a	10.1954	9.66E-05
Vrk1	10.1954	9.66E-05
Vta1	10.1954	9.66E-05
Wdr18	10.1954	9.66E-05
Wdr18	10.1954	9.66E-05
Wdr46	10.1954	9.66E-05
Wdr46	10.1954	9.66E-05
Wdr46	10.7126	7.93E-05
Wdr46	10.7126	7.93E-05
Xiap	10.1954	9.66E-05
Xiap	10.1954	9.66E-05
Xiap	10.1954	9.66E-05
Xiap	10.7126	7.93E-05
Xlr3a	10.1954	9.66E-05
Xlr3a	10.1954	9.66E-05
Xlr3a	10.1954	9.66E-05
Xlr3a	10.1954	9.66E-05
Xlr3a	10.1954	9.66E-05
Xlr3a	10.1954	9.66E-05
Xlr3a	10.1954	9.66E-05
Xlr3a	10.1954	9.66E-05
Xlr3a	10.1954	9.66E-05
Xlr3a	10.7126	7.93E-05
Xlr3a	10.7126	7.93E-05
Xlr3a	10.7126	7.93E-05
Xlr3a	10.7126	7.93E-05
Xlr3a	10.7126	7.93E-05
Xlr3a	10.7126	7.93E-05
Xlr3a	10.7126	7.93E-05
Xlr3a	10.7126	7.93E-05
Xlr3a	10.7126	7.93E-05
Xlr3a	10.7126	7.93E-05
Xlr3a	10.7126	7.93E-05
Xlr3a	10.1954	9.66E-05
Xlr3a	10.1954	9.66E-05
Xlr3a	10.7126	7.93E-05
Xlr3a	10.7126	7.93E-05
Xlr3b	10.1954	9.66E-05
Xlr3b	10.7126	7.93E-05
Xlr4b	10.1954	9.66E-05
Xlr4b	10.1954	9.66E-05
Xlr4b	10.1954	9.66E-05
Xlr4b	10.1954	9.66E-05
Xlr4b	10.1954	9.66E-05
Xlr4b	10.1954	9.66E-05
Xlr4b	10.1954	9.66E-05

Xlr4b	10.1954	9.66E-05
Xlr4b	10.1954	9.66E-05
Xlr4b	10.1954	9.66E-05
Xlr4c	10.1954	9.66E-05
Xlr4c	10.1954	9.66E-05
Xpnpep1	10.1954	9.66E-05
Xpnpep1	10.1954	9.66E-05
Xpnpep3	10.1954	9.66E-05
Yap1	10.7126	7.93E-05
Yap1	10.7126	7.93E-05
Yif1a	10.7126	7.93E-05
Yif1a	10.7126	7.93E-05
Yif1a	10.7126	7.93E-05
Yif1a	10.7126	7.93E-05
Yif1a	10.7126	7.93E-05
Ylpm1	10.1954	9.66E-05
Ythdc2	10.1954	9.66E-05
Ythdc2	10.1954	9.66E-05
Ythdf3	10.1954	9.66E-05
Ythdf3	10.1954	9.66E-05
Ythdf3	10.7126	7.93E-05
Zbtb18	10.7126	7.93E-05
Zc3h11a	10.1954	9.66E-05
Zc3h11a	10.1954	9.66E-05
Zc3h11a	10.7126	7.93E-05
Zc3h14	10.1954	9.66E-05
Zc3h14	10.1954	9.66E-05
Zc3h7a	10.1954	9.66E-05
Zc3h7a	10.1954	9.66E-05
Zc3h7a	10.1954	9.66E-05
Zdhhc20	10.1954	9.66E-05
Zfand2b	10.1954	9.66E-05
Zfand2b	10.1954	9.66E-05
Zfas1	10.1954	9.66E-05
Zfas1	10.1954	9.66E-05
Zfas1	10.1954	9.66E-05
Zfas1	10.1954	9.66E-05
Zfas1	10.1954	9.66E-05
Zfml	10.1954	9.66E-05
Zfml	10.7126	7.93E-05
Zfp120	10.1954	9.66E-05
Zfp120	10.7126	7.93E-05
Zfp120	10.7126	7.93E-05
Zfp229	10.1954	9.66E-05
Zfp229	10.1954	9.66E-05
Zfp229	10.1954	9.66E-05
Zfp229	10.1954	9.66E-05
Zfp229	10.7126	7.93E-05
Zfp280d	10.1954	9.66E-05
Zfp280d	10.1954	9.66E-05
Zfp280d	10.1954	9.66E-05
Zfp280d	10.7126	7.93E-05
Zfp346	10.7126	7.93E-05
Zfp410	10.7126	7.93E-05
Zfp655	10.1954	9.66E-05
Zfp655	10.1954	9.66E-05
Zfp672	10.1954	9.66E-05
Zfp672	10.1954	9.66E-05

Zfp672	10.7126	7.93E-05
Zfp708	10.1954	9.66E-05
Zfp708	10.1954	9.66E-05
Zfp708	10.1954	9.66E-05
Zfp708	10.1954	9.66E-05
Zfp708	10.7126	7.93E-05
Zfp708	10.7126	7.93E-05
Zfp708	10.7126	7.93E-05
Zfp708	10.7126	7.93E-05
Zfp708	10.7126	7.93E-05
Zfp708	10.7126	7.93E-05
Zfp708	10.1954	9.66E-05
Zfp708	10.1954	9.66E-05
Zfp708	10.1954	9.66E-05
Zfp708	10.1954	9.66E-05
Zfp708	10.7126	7.93E-05
Zfp708	10.7126	7.93E-05
Zfp708	10.7126	7.93E-05
Zfp708	10.7126	7.93E-05
Zfp708	10.7126	7.93E-05
Zfp708	10.7126	7.93E-05
Zfp715	10.1954	9.66E-05
Zfp715	10.1954	9.66E-05
Zfp715	10.7126	7.93E-05
Zfp810	10.7126	7.93E-05
Zfp90	10.1954	9.66E-05
Zfp90	10.1954	9.66E-05
Zfp90	10.1954	9.66E-05
Zfp90	10.7126	7.93E-05
Zfp90	10.7126	7.93E-05
Zfp932	10.1954	9.66E-05
Zfp932	10.1954	9.66E-05
Zfp932	10.7126	7.93E-05
Zfp951	10.1954	9.66E-05
Zfp951	10.7126	7.93E-05
Zfy1	10.1954	9.66E-05
Zfy1	10.1954	9.66E-05
Zfy1	10.7126	7.93E-05
ZNF654	10.1954	9.66E-05
ZNF654	10.7126	7.93E-05
ZNF654	10.7126	7.93E-05
Zscan29	10.1954	9.66E-05
Zscan29	10.1954	9.66E-05
Zscan29	10.1954	9.66E-05
Zscan29	10.7126	7.93E-05
Zscan29	10.7126	7.93E-05
Zwilch	10.1954	9.66E-05
Zwilch	10.1954	9.66E-05
Zwilch	10.1954	9.66E-05
Zwilch	10.1954	9.66E-05
Zwilch	10.1954	9.66E-05
Zwilch	10.7126	7.93E-05
Zwilch	10.7126	7.93E-05
Zwilch	10.7126	7.93E-05
Zwilch	10.7126	7.93E-05
Zwilch	10.7126	7.93E-05
Zwilch	10.1954	9.66E-05
Zwilch	10.1954	9.66E-05

Zwilch	10.1954	9.66E-05
Zwilch	10.1954	9.66E-05
Zwilch	10.1954	9.66E-05
Zwilch	10.7126	7.93E-05
Zwilch	10.7126	7.93E-05
Zwilch	10.7126	7.93E-05
