# Supplementary material for: Integrative proteome analysis implicates aberrant RNA splicing in impaired developmental potential of aged mouse oocytes
Source: Aging Cell. 2021 Sep 28;20(10):e13482. doi: 10.1111/acel.13482 (PMC8520726; doi:10.1111/acel.13482)
Supplement: Supplementary file 11 — Table S7 [file ACEL-20-e13482-s010.pdf]

Table S7. Lists of differentially expressed genes among MII oocytes from 8-10w, 6-8m, 10-12m mice (8-10w versus 6-8m, 8-10w versus 10-12m and 6-8m versus 10-12m).

Table S7-1. List of differentially expressed genes between MII oocytes from 8-10w and 6-8m mice.

gene name	baseMean	log2FoldChange	lfcSE	stat	pvalue	padj
Prl8a2	384.4715713	-1.88874338	0.134873193	-14.0038456	1.48E-44	2.32E-40
Gm29106	663.0293312	-1.055552813	0.1121412	-9.41271198	4.83E-21	2.53E-17
Lipo3	87.49966271	-2.075086756	0.2306247	-8.99767784	2.31E-19	9.06E-16
Ddx60	457.6483759	-1.038907635	0.134627904	-7.71688188	1.19E-14	2.08E-11
Gca	133.2638081	-1.243882073	0.177011877	-7.02711079	2.11E-12	1.66E-09
Tlr8	143.1083724	-1.364313548	0.19505047	-6.99466938	2.66E-12	1.99E-09
Pet2	108.2281413	-1.113935273	0.201431395	-5.53009758	3.20E-08	8.11E-06
Nyap2	161.3049201	-1.023173296	0.196436654	-5.20866792	1.90E-07	3.56E-05
Car1	48.63771006	-1.883388859	0.367757408	-5.12128054	3.03E-07	5.36E-05
Vmn2r26	40.51295187	-1.531210876	0.315584873	-4.85197805	1.22E-06	0.000176222
Cox4i2	54.13339538	1.214184433	0.278254361	4.363577374	1.28E-05	0.001136622
Chrdl1	50.58720338	-1.526271336	0.352738735	-4.32691731	1.51E-05	0.001305563
Frmd3	69.661485	-1.103124928	0.257884617	-4.27759105	1.89E-05	0.001596126
Ldlr	28.62780108	-1.485282227	0.392807657	-3.78119469	0.000156078	0.008457613
Ppic	15.77914015	1.890825665	0.530955219	3.561177285	0.000369196	0.015307496
Pcsk9	23.54383472	1.368463378	0.40856909	3.349405065	0.000809853	0.02694165
H2-T23	13.63800218	1.793689324	0.550250605	3.259768016	0.001115034	0.03382556
Tcaf1	32.68495203	-1.082260973	0.339361736	-3.18910726	0.001427129	0.039024096
Trim29	2.796251169	4.899225743	1.554665638	3.151305092	0.001625426	0.042148424
Tmprss11g	10.09943479	-2.234942141	0.719828099	-3.10482759	0.0019039	0.047488698
Sh3gl3	27.35296791	1.122212644	0.368424666	3.045975877	0.002319264	0.053501937
Evc	12.29107622	1.717957526	0.577731163	2.973627937	0.002943017	0.062579929
Pcnx2	28.6158793	1.046100738	0.359530575	2.909629422	0.003618575	0.07188659
Loxl2	99.23512667	-1.940618824	0.667612378	-2.90680474	0.00365141	0.072173917
Ntrk3	18.4363971	-1.392764948	0.494098491	-2.81880025	0.004820351	0.086174048
Slc16a6	19.21911128	1.301112234	0.464874771	2.798844583	0.005128582	0.089844516
Wnt2b	5.898516719	2.57416907	0.929120487	2.77054387	0.005596276	0.09486503
Slc25a37	2.147799071	-4.584191098	1.661555092	-2.75897629	0.005798274	0.096960912

Table S7-2. List of differentially expressed genes between MII oocytes from 8-10w and 10-12m mice.

gene name	baseMean	log2FoldChange	lfcSE	stat	pvalue	padj
Nyap2	110.6962945	-2.395084528	0.23794339	-10.0657746	7.83E-24	1.22E-19
Fth1	1059.302369	1.484179555	0.154958383	9.5779236	9.90E-22	7.75E-18
Cttnbp2	169.8914866	-1.614189946	0.177749991	-9.08123787	1.07E-19	4.58E-16
Ddx60	348.7999566	-1.663942332	0.183418415	-9.07183903	1.17E-19	4.58E-16
Tlr8	112.0769857	-1.936003929	0.214605115	-9.02123853	1.86E-19	5.82E-16
Enpp2	579.4765494	-1.065723049	0.122675745	-8.68731671	3.71E-18	8.30E-15
Slc5a12	406.049359	-1.04028643	0.133095046	-7.81611684	5.45E-15	1.07E-11
Lipo3	76.61900018	-1.975011359	0.282125881	-7.00046146	2.55E-12	3.33E-09
Sult1c1	169.7883577	1.317629809	0.19793252	6.656964742	2.80E-11	2.43E-08
Mdga2	172.3020759	-1.030713796	0.166037644	-6.20771152	5.38E-10	3.66E-07
Wls	237.7900824	-1.060385247	0.176280162	-6.01534077	1.80E-09	9.69E-07
Gca	115.5990401	-1.224238497	0.219474469	-5.57804515	2.43E-08	1.03E-05
Vmn2r26	31.58442388	-2.20173834	0.397913227	-5.53321225	3.14E-08	1.26E-05
Tex16	131.5135844	-1.133373807	0.205194543	-5.52341105	3.32E-08	1.28E-05
Chrdl1	39.52908836	-2.211634853	0.411822586	-5.37035833	7.86E-08	2.62E-05
Yipf1	382.6965295	1.067499041	0.201672449	5.293231901	1.20E-07	3.76E-05

Trpc5	49.63634952	-1.567139956	0.296550414	-5.28456506	1.26E-07	3.87E-05
Cdh8	53.16447017	-1.588668355	0.30272425	-5.24790583	1.54E-07	4.63E-05
Cox4i2	57.90442968	1.639356118	0.316258333	5.183598175	2.18E-07	5.77E-05
Sh3gl3	32.73873938	1.773438306	0.359632557	4.931250721	8.17E-07	0.000157872
Trpc5os	81.8689219	-1.34994287	0.285639824	-4.72603173	2.29E-06	0.000341266
Frmd3	56.25637914	-1.442764097	0.307141455	-4.69739292	2.64E-06	0.00038186
Trappc2l	184.0891883	1.192040036	0.255381701	4.667679923	3.05E-06	0.000437395
Ppef2	63.72977021	-1.186492453	0.256617611	-4.62358155	3.77E-06	0.000500262
Spon2	148.3476272	1.042512239	0.225451264	4.624113524	3.76E-06	0.000500262
Isl1	91.53508395	-1.02304089	0.231765933	-4.41411245	1.01E-05	0.001079868
Prl8a2	333.4098891	-1.829620499	0.430240706	-4.2525509	2.11E-05	0.001923154
Car1	44.81293733	-1.518350748	0.371276719	-4.08953934	4.32E-05	0.003221354
Pet100	28.87067465	1.794668788	0.4507298	3.981695439	6.84E-05	0.004523696
Tcaf1	25.49314168	-1.555117079	0.40082341	-3.87980602	0.00010454	0.006101852
Tekt2	45.67025636	1.115054724	0.292112958	3.817203904	0.000134973	0.007438227
Lage3	65.42910364	1.03932344	0.276842412	3.754205983	0.000173892	0.008923216
Vwa5a	7.77057133	-4.298971475	1.1723615	-3.66693334	0.000245477	0.011537407
Tnc	16.71751701	-2.126199833	0.588494035	-3.61295052	0.000302732	0.013270557
Gadl1	13.4600762	2.338868038	0.656690313	3.561599726	0.000368602	0.01510207
Krt27	52.43736906	1.105000129	0.311759989	3.544393659	0.000393517	0.015873551
Uqcr10	636.4232451	1.180209038	0.368407418	3.203543092	0.001357477	0.036876365
Gm10332	41.32801863	1.098978905	0.348576326	3.152764035	0.001617325	0.041312884
Colca2	3.522414864	4.431340817	1.467815562	3.019003839	0.002536073	0.056062262
Mid1ip1	14.07201917	-1.793627114	0.595536629	-3.01178303	0.002597182	0.056767551
Lrrc51	8.935683416	2.028175047	0.68692071	2.952560635	0.003151502	0.065071442
Try4	24.59874773	1.148325551	0.396357209	2.897198598	0.003765113	0.073659735
Pcnx2	26.9282857	1.212429141	0.424956224	2.853068321	0.004329931	0.080621041
Slit3	26.23655291	-1.144340663	0.406424182	-2.81563134	0.004868149	0.086581143
Nts	13.57757091	1.513080093	0.538225341	2.811239042	0.004935111	0.087249846
Snhg11	16.93336326	1.768504773	0.645289339	2.740638449	0.006131994	0.098231157

Table S7-3. List of differentially expressed genes between MII oocytes from 6-8m and 10-12m mice.

gene name	baseMean	log2FoldChange	lfcSE	stat	pvalue	padj
Ccdc12	274.1339718	1.136495241	0.175470474	6.476846026	9.37E-11	1.47E-06
Loxl2	70.46286446	1.530738592	0.324211893	4.721414067	2.34E-06	0.003055667
Nyap2	64.45527234	-1.362439748	0.307749685	-4.42710363	9.55E-06	0.008301048
Olfr1369-ps1	71.09033415	1.336789836	0.339797623	3.934076485	8.35E-05	0.023862027
Ldlr	49.96022593	2.711403337	0.751990463	3.605635269	0.00031139	0.053566986
Fth1	1074.653395	1.515285127	0.427131354	3.547585801	0.000388779	0.063582753
Tbc1d25	52.43515179	-1.025087278	0.294190228	-3.48443688	0.000493174	0.071491931
Pet100	30.58795874	1.56038084	0.450430412	3.464199567	0.000531812	0.075691325
