# Supplementary material for: Integrative proteome analysis implicates aberrant RNA splicing in impaired developmental potential of aged mouse oocytes
Source: Aging Cell. 2021 Sep 28;20(10):e13482. doi: 10.1111/acel.13482 (PMC8520726; doi:10.1111/acel.13482)
Supplement: Supplementary file 12 — Supplementary Material [file ACEL-20-e13482-s008.docx]

**Supplementary Material**

**Supplementary Experimental Procedures**

**Oocyte retrieval and classification**

The female mice in the three age groups were superovulated by injecting 5 IU pregnant mare serum gonadotropin (PMSG) (Ningbo Sansheng Pharmaceutical Corporation, Zhejiang, China) followed by 5 IU human chorionic gonadotropin (hCG) (Ningbo Sansheng Pharmaceutical Corporation) 46–48 h after PMSG priming. Oocytes were collected 14–15 h after hCG injection. Cumulus-oocyte complexes (COCs) were isolated from oviduct ampullae, and denuded MII oocytes were obtained by removing the cumulus mass in M2 medium (M7167, Sigma) containing 0.5 mg/mL hyaluronidase at 37°C. Oocytes were counted and classified as MII (presence of first polar body in the perivitelline space) or dead (condensed, fragmented cytoplasm or vacuoles).

**IVF and embryo culture**

Sperms were isolated from dissected epididymides of adult C57BL/6 mice and capacitated for 1.5 h at 37°C in human tubal fluid fertilization medium (Fujifilm Irvine Scientific, Santa Ana, CA, USA) supplemented with 10 mg/mL bovine serum albumin. Dispersed spermatozoa were then added to drops of human tubal fluid medium containing COCs. After coincubation for 4–5 h in a 37°C incubator, the inseminated oocytes were washed to remove cumulus cells and excess sperm. The number of embryos that formed pronuclei was used as a measure of fertilization rate. For the culture of preimplantation embryos, zygotes were transferred to KSOM medium (Millipore Sigma) and cultured to the 2-cell, morula, or blastocyst stage.

**Immunofluorescence staining**

Oocytes and preimplantation embryos were fixed with 4% paraformaldehyde overnight at 4°C and incubated in 0.5% TritonX-100 for 30 min at 37°C. Samples were then blocked in 1% bovine serum albumin in PBS for 1 h and incubated overnight at 4°C with primary antibodies. The following antibodies were used at the following dilutions: anti-α-tubulin (1:250) (T6074; Sigma) and anti-γH2AX (1:500) (ab22551; Abcam). After three washes with PBS, the samples were incubated with secondary antibodies for 1 h at room temperature, washed three times with PBS, and stained with Hoechst 33342 (Invitrogen) for 10 min. The samples were mounted on glass slides and observed by confocal microscopy (LSM 800; Carl Zeiss, Germany).

**Mitochondrial staining**

MII oocytes were incubated in M2 medium supplemented with 200 nm MitoTracker™ Orange CMTMRos (Invitrogen) for 60 min at 37°C. Oocytes were then washed and fixed with 4% paraformaldehyde for 30 min at room temperature and washed again. Then, the oocytes were mounted on glass slides and observed using a confocal laser scanning microscope (LSM 800; Carl Zeiss, Germany).

**Chromosome spread**

Oocytes were exposed to Tyrode’s buffer (pH 2.5) for 30 s at 37°C to remove the zona pellucida. After recovery in M2 medium for 10 min, the oocytes were fixed in a drop of 1% paraformaldehyde and 0.15% Triton X-100 on a glass slide. After air drying, the oocytes were incubated with anti-centromere antibody (1:500) (15–234; Antibodies Incorporated) overnight at 4°C, and then incubated with secondary antibody for 1 h for kinetochore labelling. Chromosomes were stained with Hoechst 33342, and the samples were examined using a confocal laser scanning microscope (LSM 800).

**TUNEL assay**

To analyse apoptosis at the blastocyst stage, a terminal deoxynucleotidyl transferase dUTP nick-end labelling (TUNEL) assay was performed on blastocysts from three age groups using the In Situ Cell Death Detection Kit (11684795910; Roche). The procedures were carried out according to the instructions provided by the manufacturer. Fluorescence was detected using a confocal laser scanning microscope (LSM 800).

**EU incorporation assay**

The 2-cell embryos at different time points (24, 26, 28, 30, and 32 hpi) were cultured in KSOM medium with 1mM 5-ethynyl uridine (EU) for 2 h. Fixation, permeabilization, and staining were performed using the Click-iT RNA Alexa Fluor 594 Imaging Kit (C10330; Invitrogen) according to the manufacturer’s instructions. The 2-cells were detected and imaged using a confocal laser scanning microscope (LSM 800).

**RNA isolation, cDNA synthesis and RT-PCR analysis**

Total RNA was extracted from 2-cell embryos of 28 hpi from the three age groups using the RNeasy Plus Micro Kit (74034; Qiagen, Hilden, Germany). The reverse transcription enzyme (DRR036A; Takara) was used for cDNA synthesis. Semi-quantitative RT-PCR was performed using Premix Taq (RR901; Takara). Primers were designed flanking the skipped exons of DSGs. *Cdk9* primers were designed as 5’-CTGAACGGCCTCTACTACATC-3’ and 5’-GTCAATGAGGTCCAGAGCAT-3’, *Ube2d3* primers were designed as 5’- CCTTTGAGTACACCTCGGGA-3’ and 5’-GGTCGTCTGGGTTTGGATCA-3’, *Mnat1* primers were designed as 5’-AAGCATTGGAGGTAGAACGC-3’ and 5’- GCAAGATCCTGTGGAGATGC-3’, *Fanca* primers were designed as 5’- AGTGTCCCGTGATTCTGACT-3’ and 5’- TCTGTCAACTGGAAGAGCAC-3’, 18S primers were designed as 5’-GTAACCCGTTGAACCCCATT-3’ and 5’-CCATCCAATCGGTAGTAGCG-3’, *Actin* primers were designed as 5’- AGATCAAGATCATTGCTCCTCCTGA-3’ and 5’- GCAGCTCAGTAACAGTCCGC-3’. 18S rRNA and *Actin* were used as internal control.

**Western blotting**

A total of 50 oocytes were collected in RIPA lysis buffer (CW2333; CWBIO) supplemented with 1% protease inhibitor cocktail (CW2200; CWBIO) and 1% phosphatase inhibitor cocktail (CW2383; CWBIO). After boiling for 5 min, the total lysates of each group were subjected to a 4‒20% SDS-PAGE and then transferred to a polyvinyl fluoride membrane. Non-specific binding sites were blocked with 5% non-fat milk in Tris-buffered saline for 2 h at room temperature and then incubated overnight with primary antibodies: PUF60 (MA5-27821; Thermo Fisher Scientific) and Actin (AC026; Abclonal) at 4°C. After washing three times in Tris-buffered saline containing 0.05% Tween-20 (TBST), the membranes were incubated with the appropriate secondary antibody for 2 h at room temperature, followed by three washes with TBST. The protein bands were then visualised using a Bio-Rad gel imaging system.

**Morpholino microinjection**

Approximately 10 pL of morpholino (Gene Tools, USA) was injected into zygotes at a concentration of 1 mM. The zygotes were then cultured in KSOM medium, and after culture, 2-cell embryos of 28 hpi were collected for assessment of the changes in alternative splicing events by RT-PCR. The sequences of *Cdk9* morpholinos are 5’-CTGCCCGCTCGGGAGTCCTCACC-3’ and 5’-ACACCCAGTTCGGTTACTCACCTCT-3’, and the sequences of the control morpholino are 5’- CCTCTTACCTCAGTTACAATTTATA-3’.

**Supplementary Figure Legends**

**Figure S1.** PCA and pathway mapping of 187 DE proteins. (a) PCA of 187 DE proteins among three age groups. The first two principal components are plotted. There were 4 replicates for each stage. (b) Spliceosome assembly pathway mapping of the identified splicing-related DE proteins. Splicing-related DE proteins are listed in boxes near the spliceosome assembly steps in red.

**Figure S2.** Time point determination of splicing analysis. (a) Overview of transcription dynamics from oocyte to preimplantation development. (b) The 2-cell embryos of 24-34hpi were cultured with 5-ethynyl uridine (EU). The signals of nascent RNA are detected as red fluorescence, whereas the nuclei are blue (Hoechst). Scale bar, 10 μm. (c) Quantification of EU fluorescence intensity in 2-cell embryos of 24-34 hpi (24-26 hpi, n=21; 26-28 hpi, n= 22; 28-30 hpi, n=27; 30-32 hpi, n=30; 32-34 hpi, n=25). Data are presented as the mean ± SEM.

**Figure S3.** Analysis and validation of splicing events of DNA damage repair/response related DSGs. Sashimi plot and RT-PCR validation of *Ube2d3* (a), *Mnat1* (b), and *Fanca* (c) in 2-cell embryos from three age groups. Sashimi plot indicating the average RNA-seq read density and splice junction counts. The exon inclusion level (Inclusion%) was predicted by RNA-Seq data.

**Figure S4.** Validation of *Cdk9* morpholino’s effectiveness and preimplantation embryo development in control and *Cdk9* morpholinos-injected 2-cell embryos. (a) RT-PCR validation of splicing events of *Cdk9* morpholino-injected 2-cell embryos. (b) Blastocyst rate in control and *Cdk9* morpholinos-injected 2-cell embryos (from three independent experiments). Data are presented as the mean ± SEM.

**Supplementary Table Legends**

**Table S1.** List of 187 proteins that were differentially expressed in MII oocytes of three different age groups.

**Table S2.** GO analysis of 187 DE proteins according to biological process.

**Table S3.** KEGG pathway analysis of 187 DE proteins.

**Table S4.** List of alternative splicing events and genes at the 2-cell stage (10-12m versus 8-10w).

**Table S5.** List of alternative splicing events and genes at the 2-cell stage (6-8m versus 8-10w).

**Table S6.** List of PUF60-binding DSGs in the two reproductively aging groups versus the younger group.

**Table S7.** Lists of differentially expressed genes among MII oocytes from 8-10w, 6-8m, 10-12m mice (8-10w versus 6-8m, 8-10w versus 10-12m and 6-8m versus 10-12m).
